# Enantioselective Miyaura Reaction by Desymmetrizing C(sp^2^)–B Cross‐Coupling of 1,1′‐Biaryl‐2,6‐diyl Bis(nonaflates)

**DOI:** 10.1002/anie.202515234

**Published:** 2025-10-13

**Authors:** Yao Xiao, Annika L. Bartelt, Elisabeth Irran, Martin Oestreich

**Affiliations:** ^1^ Institut für Chemie Technische Universität Berlin Strasse des 17. Juni 115 10623 Berlin Germany

**Keywords:** Boron, Copper, Cross‐coupling, Desymmetrization, Palladium

## Abstract

An efficient protocol for a desymmetrizing C(sp^2^)–B cross‐coupling of achiral 1,1′‐biaryl‐2,6‐diyl bis(nonaflates) and B–B reagents is disclosed. An in situ‐formed palladium(0)–(*S*,*S*)‐f‐Binaphane complex discriminates between the enantiotopic nonaflate groups, and the subsequent transmetalation of the B–B reagent is enhanced by a copper co‐catalyst. The enantiomeric excess of the chiral monoborylated product after reductive elimination is further improved by a downstream kinetic resolution, thereby converting the minor enantiomer into the corresponding achiral bisborylated biaryl byproduct. This enantioselective Miyaura reaction enables the synthesis of highly valuable, axially chiral boron compounds with superb enantiomeric ratios (up to e.r. = 99:1) and exhibits broad substrate scope and good functional‐group tolerance.

## Graphical abstract

A desymmetrization strategy for an enantioselective Miyaura‐type C(sp^2^)–B cross‐coupling to access highly enantioenriched, axially chiral biaryl boronic esters is disclosed. The enantiomeric excess of the chiral monoborylated product after reductive elimination is further improved by a downstream kinetic resolution, thereby converting the minor enantiomer into the corresponding achiral bisborylated biaryl byproduct.

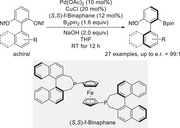

Arylboronic acids and arylboronate esters are central to synthetic organic chemistry, especially due the enormous success of the Suzuki–Miyaura cross‐coupling^[^ ^1^, ^2^ ^]^ with applications in academic and industrial settings.^[^ ^3^, ^4^, ^5^ ^]^ Consequently, extensive efforts have been devoted to develop reliable methods for the preparation of those boron pronucleophiles. Traditional approaches typically involve the reaction of aryl metal reagents with boron halides or trialkylborates to produce arylboron compounds.^[^ ^6^, ^7^, ^8^, ^9^, ^10^ ^]^ An alternative strategy was introduced by Miyaura and co‐workers, that is the palladium‐catalyzed cross‐coupling of aryl electrophiles and diboron reagents to forge a C(sp^2^)–B bond (Scheme 1a).^[^ ^11^, ^12^, ^13^, ^14^ ^]^ Other transition‐metal‐catalyzed protocols were subsequently developed.^[^ ^15^, ^16^, ^17^, ^18^, ^19^, ^20^, ^21^, ^22^, ^23^, ^24^, ^25^, ^26^ ^]^ Yet all of these methods remain limited to the preparation of achiral arylboronic esters. Enantioselective variants of such C(sp^2^)–B cross‐coupling reactions are not known to date. Of note, Xu and co‐workers accomplished iridium‐catalyzed atroposelective C(sp^2^)–H bond borylation in biaryls remote from the *ortho*,*ortho*’ positions.^[^ ^27^, ^28^ ^]^

**Scheme 1:**
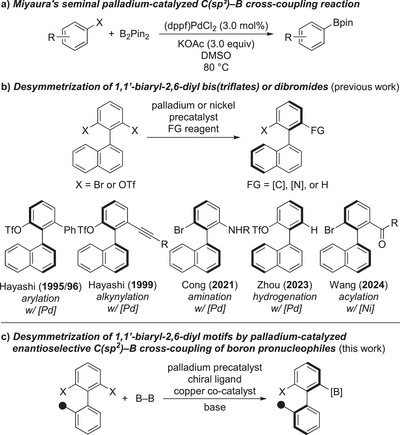
Background (a and b) and planned work (c) for the desymmetrization of 1,1′‐biaryl‐2,6‐diyl di(pseudo)halides. R group = any type of substituent, Tf = trifluoromethanesulfonyl.

Desymmetrization^[^ ^29^, ^30^, ^31^ ^]^ is a powerful way to convert achiral biaryl derivatives into axially chiral molecules^[^ ^32^, ^33^ ^]^ (Scheme 1b). Hayashi and co‐workers were first to demonstrate the desymmetrization of 1,1′‐biaryl‐2,6‐diyl bis(triflates) by an atroposelective arylation.^[^ ^34^ ^]^ The same laboratory also achieved alkynylation^[^ ^35^ ^]^ and again arylation^[^ ^36^ ^]^ of this class of compounds by using Grignard reagents as nucleophiles. Cong and co‐workers accomplished a palladium‐catalyzed amination reaction of the dibromide congeners,^[^ ^37^ ^]^ and just recently, a desymmetrizing hydrogenolysis of the bis(triflates) was realized by Zhou and co‐workers.^[^ ^38^ ^]^ Wang and co‐workers also showed that the dibromides engage in a nickel‐catalyzed acylation.^[^ ^39^ ^]^ Given this literature precedent, we decided to elaborate a desymmetrizing borylation of 1,1′‐biaryl‐2,6‐diyl di(pseudo)halides, corresponding to an enantioselective Miyaura C(sp^2^)–B cross‐coupling (Scheme 1c).

We started our investigation with a preliminary screening of various commonly used chiral ligands to achieve the enantioselective cross‐coupling of 2‐(naphthalen‐1‐yl)‐1,3‐phenylene bis(trifluoromethanesulfonate) (**1a**) and B_2_pin_2_ (**5a**) (see Table S1). With Pd(OAc)_2_ as the precatalyst and K_3_PO_4_ as the base in the presence of a copper co‐catalyst, (*S*,*S*)‐f‐Binaphane was found to be a viable ligand, yielding the product **6aa** in 56% yield with an enantiomeric ratio of 89:11 (Table 1, entry 1). Two blank experiments without a palladium precatalyst or a copper salt were conducted. There was no reaction without a palladium precatalyst (entry 2), and when there was no copper salt, the monoborylation only proceeded in 43% yield and with an e.r. = 76:24 (entry 3). As documented by ample evidence, the copper co‐catalyst or, to be precise, an in situ‐formed copper alkoxide is facilitating B–B bond activation, thereby improving the efficiency of the transmetalation.^[^ ^21^, ^40^ ^]^ Screening of other palladium precatalysts revealed that Pd(OAc)_2_ seemed to be optimal (entries 4 and 5). Then, the effect of different copper salts was explored (entries 6 and 7), and CuCl did give the best enantiomeric ratios. To our delight, a further improvement in both yield and enantioselectivity was achieved when using NaOH or KOH as the base (entries 8 and 9). Notably, the use of NaOH resulted in an 80% yield and an e.r. of 92:8 (entry 8). Nonaflate, serving as a cost‐effective alternative to the triflate group,^[^ ^41^ ^]^ delivered an even better outcome, affording the product **7aa** from **2a** in 86% yield with an e.r. of 94:6 (entry 10). When bis(tosylate) **3a** or dibromide **4a** were used as reactants, neither product **8aa** nor **9aa** was observed (entries 11 and 12). Assuming the stereochemical outcome is further enhanced by a downstream kinetic resolution process, 1.6 equiv. of B_2_pin_2_ were used, furnishing the monoborylated product **7aa** in 82% yield with e.r. = 95: 5 along with the achiral bisborylated product **10aa** in 12% yield (entry 13). The optimized reaction conditions were established as 10 mol% of Pd(OAc)_2_, 12 mol% of the chiral ligand, and 20 mol% of CuCl, which gave **7aa** with an isolated yield of 67% with an e.r. of 97:3 (entry 14).

**Table 1:** Optimization of the reaction conditions.^a)^

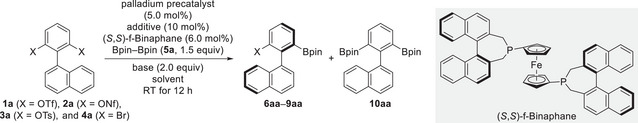								
Entry	X	Precatalyst	Additive	Base	Solvent	Yield (%)^b)^	e.r.^c)^	Yield of **10aa** (%)^b)^
1	OTf (**1a**)	Pd(OAc)_2_	CuCl	K_3_PO_4_	THF	56 (**6aa**)	89:11 (**6aa**)	<10
2	OTf (**1a**)	–	CuCl	K_3_PO_4_	THF	–	–	–
3	OTf (**1a**)	Pd(OAc)_2_	–	K_3_PO_4_	THF	43 (**6aa**)	76:24 (**6aa**)	<10
4	OTf (**1a**)	Pd(acac)_2_	CuCl	K_3_PO_4_	THF	50 (**6aa**)	84:16 (**6aa**)	<10
5	OTf (**1a**)	Pd_2_(dba)_3_	CuCl	K_3_PO_4_	THF	38 (**6aa**)	86:14 (**6aa**)	<10
6	OTf (**1a**)	Pd(OAc)_2_	CuTc	K_3_PO_4_	THF	60 (**6aa**)	80:20 (**6aa**)	<10
7	OTf (**1a**)	Pd(OAc)_2_	CuI	K_3_PO_4_	THF	55 (**6aa**)	85:15 (**6aa**)	<10
8	OTf (**1a**)	Pd(OAc)_2_	CuCl	NaOH	THF	80 (**6aa**)	92:8 (**6aa**)	<10
9	OTf (**1a**)	Pd(OAc)_2_	CuCl	KOH	THF	77 (**6aa**)	92:8 (**6aa**)	<10
10	ONf (**2a**)	Pd(OAc)_2_	CuCl	NaOH	THF	86 (**7aa**)	94:6 (**7aa**)	<10
11	OTs (**3a**)	Pd(OAc)_2_	CuCl	NaOH	THF	— (**8aa**)	–	–
12	Br (**4a**)	Pd(OAc)_2_	CuCl	NaOH	THF	— (**9aa**)	–	–
13^d)^	ONf (**2a**)	Pd(OAc)_2_	CuCl	NaOH	THF	82 (**7aa**)	95:5 (**7aa**)	12
14^d)^, ^e)^	ONf (**2a**)	Pd(OAc)_2_	CuCl	NaOH	THF	77 (67)^f)^ (**7aa**)	97:3 (**7aa**)	16

^a)^ Unless otherwise noted, all reactions were performed on a 0.10 mmol scale at room temperature under nitrogen atmosphere.

^b)^ Yields were estimated by ^1^H NMR spectroscopy.

^c)^ Enantiomeric ratios were determined by HPLC analysis on chiral stationary phases.

^d)^ 1.6 equiv. of **5 a** was used.

^e)^ 10 mol% of Pd(OAc)_2_, 12 mol% of (*S*,*S*)‐f‐Binaphane, and 20 mol% of CuCl were used.

^f)^ 67 % isolated yield on a 0.10‐mmol scale after purification on silica gel. Nf = 1,1,2,2,3,3,4,4,4‐nonafluorobutane‐1‐sulfonyl, Ts = *p*‐toluenesulfonyl, acac = acetylacetonate, dba = dibenzylideneacetone, Tc = thiophene‐2‐carboxylate.

With the optimized conditions in hand, we evaluated the substrate scope of naphth‐1′‐yl group. The absolute configuration of **7aa** was determined as *R* by X‐ray diffraction analysis.^[^ ^42^ ^]^ As illustrated in Scheme 2, the reaction easily extended to substrates **2b**–**e** bearing an additional electron‐withdrawing or ‐donating group such as a methyl group, a fluorine atom or a cyano group at C4’ of the naphthalene ring. The products **7ba**–**ea** were obtained in moderate to good yields and with moderate to high enantiomeric ratios, e.g. e.r. = 90:10 for **7da** with a bromine at C4’ and e.r. = 99:1 for **7ea** with a cyano group at C4’. Moreover, substrates bearing a phenyl (**2f**) or an NHTs (**2g**) group at C5’ were compatible with the reaction conditions, yielding the products **7fa** and **7ga** with excellent results. In contrast, a substrate bearing a methyl group at C2’ yielded product **7ha** in 72% yield and in an almost racemic form. As for other positions on naphthalene ring, the product **7ia** with methyl group at C8′ was also formed in 67% yield with e.r. = 99:1. Moreover, the methodology was successfully applied the more complicated biaryl bis(nonaflates) **2j** and **2k** to bring about the formation of **7ja** and **7ka** in good yields and with excellent enantiomeric ratios.

**Scheme 2:**
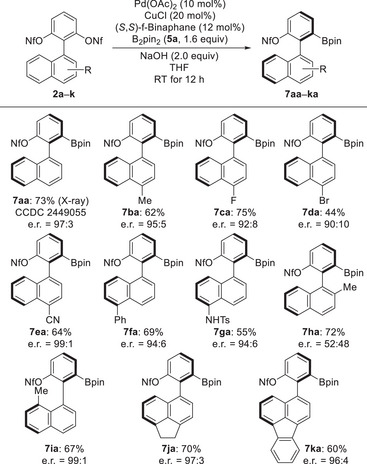
Substrate scope I: variation of the substituents on the naphthyl ring. All reactions were performed on a 0.10‐mmol scale using bis(nonaflates) **2**, 1.6 equiv. of the B–B reagent **5a**, 10 mol% of Pd(OAc)_2_, 20 mol% of CuCl, 12 mol% of (*S*,*S*)‐f‐Binaphane, and 2.0 equiv. of KOH in THF at RT. Yields were determined after purification by flash chromatography on silica gel or preparative thin‐layer chromatography. Enantiomeric ratios were determined by HPLC analysis on chiral stationary phases.

Encouraged by the above results, we next applied our protocol to the 2′‐substituted 1,1'‐biphenyl‐2,6‐diyl bis(nonaflates), which can be used to synthesize chiral biphenyl borate compounds with different backbones (Scheme 3). Substrate **2l** bearing a phenyl group was evaluated, and the corresponding product **7la** was efficiently obtained in 71% yield with e.r. = 98:2. Different functional groups at the 2′‐phenyl‐substituted position including *ortho*‐F, *meta*‐Cl, *para*‐*t*Bu, *para*‐C(O)Me, and *para*‐NO_2_ were well tolerated, giving products **7ma–qa** in yields ranging from 47% to 80% and with good to excellent enantioselectivity. The reactivity was not significantly affected for **7ra** with two substituents. Conversely, a 2′‐phenyl and a 5′‐methyl group was highly detrimental, furnishing **7sa** in an almost racemic form. This did not change when using various amounts of B_2_pin_2_ (**5a**), and we can only speculate at this stage but this position seems to be incompatible with the chiral catalyst due steric interactions (see also the ineffective borylation of **2h** in Scheme 2). The scope of this desymmetrization strategy could be further expanded other 2′‐substituted bis(nonaflates). Products **7ta** and **7ua** originating from **2t** and **2u**, respectively did form in moderate to good yields and with high enantioselection. It is worthy of note that substrates with a cyano or an isopropyl group at C2′ were equally compatible with the reaction conditions, yielding the product **7va** in 66% yield and with e.r. = 98:2 and **7wa** in 75% yield and with e.r. = 98:2. For comparison, the more challenging substrates **2x** (R = Me) and **2y** (R = Cl) bearing small groups at C2′ gave products **7xa** and **7ya** in good yields and with moderate enantiomeric ratios; potential configurational lability is not an issue here.

**Scheme 3:**
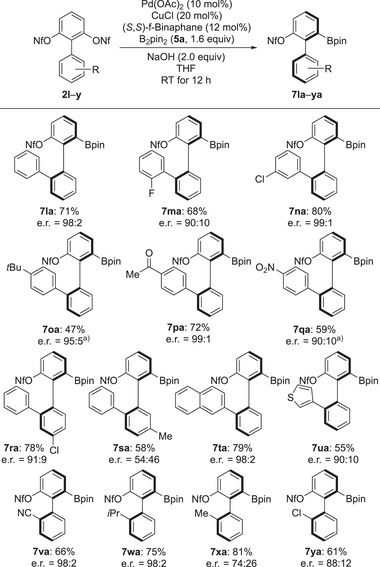
Substrate scope II: variation of the substituents on the phenyl ring. All reactions were performed on a 0.10‐mmol scale using bis(nonaflates) **2**, 1.6 equiv. of the B–B reagent **5a**, 10 mol% of Pd(OAc)_2_, 20 mol% of CuCl, 12 mol% of (*S*,*S*)‐f‐Binaphane, and 2.0 equiv. of KOH in THF at RT. Yields were determined after purification by flash chromatography on silica gel or preparative thin‐layer chromatography. Enantiomeric ratios were determined by HPLC analysis on chiral stationary phases. ^a)^ The reaction time was 36 h.

Our survey of the substrate scope also included other diboron pronucleophiles (Figure 1). We subjected B_2_(neop)_2_ (**5b**) and B_2_(dmpd)_2_ (**5c**) to the standard procedure with bis(nonaflate) **2l** as the electrophile. The monoborylated products **7lb** and **7lc** did form in good yield and with good enantiomeric ratios. In turn, **7ld** was only found in trace amounts with B_2_(cat)_2_ (**5d**), and the starting material **2l** remained unreacted.

**Figure 1:**
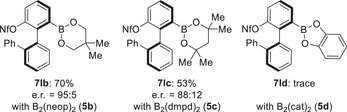
Scope III: variation of the B–B reagent.

To highlight the practicality of this methodology, synthetic applications were surveyed (Scheme 4). The model reaction of **2a** was run on a 1.0‐mmol scale and demonstrated good scalability. Product **7aa** was isolated in a 63% yield and with e.r. = 97:3. The boryl group in **7aa** can be reacted with NaN_3_ in the presence of Cu(OAc)_2_, affording the azide **11** in 72% yield and with e.r. = 97:3. The boryl group could also be transformed into an imidazole as in **12**. The oxidation of **7aa** with H_2_O_2_/NaOH gave the phenol derivative **13**. A Suzuki–Miyaura coupling was successfully performed by the reaction with iodobenzene to yield the chiral *ortho*‐terphenyl derivative **14** in 68% yield and with e.r. = 97:3.

**Scheme 4:**
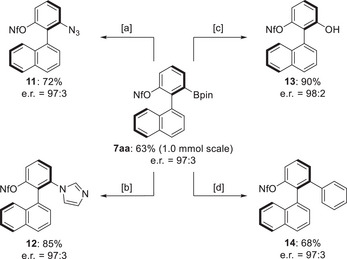
Derivatization of a desymmetrized product. Reaction conditions for **7aa** (0.10 mmol): [a] Cu(OAc)_2_ (20 mol%), NaN_3_ (5.0 equiv.), MeOH (1 mL), 50 °C, 8 h. [b] Cu_2_O (10 mol%), imidazole (1.5 equiv.), MeOH (1 mL), 40 °C, 12 h. [c] aq. H_2_O_2_ (30 wt%, 1.0 mL), aq. NaOH (2.0 M, 1.0 mL), THF (1.0 mL), RT, 12 h. [d] (Ph_3_P)_4_Pd (10 mol%), PhI (3.0 equiv.), K_2_CO_3_ (3.0 equiv.), 1,4‐dioxane (1.0 mL), H_2_O (0.20 mL), 60 °C, 24 h.

To distinguish between the contributions of the two different steps (desymmetrization followed by kinetic resolution) to the level of enantioselection, we performed two control experiments (Scheme 5). When the reaction of the model bis(nonaflate) **2a** was run with the B–B compound B_2_pin_2_ (**5a**) as the limiting reagent (0.6 instead of 1.6 equiv.) under the optimized conditions, the desired product (*R*)‐**7aa** was obtained in 42 % yield and an e.r. of 85:15 (Scheme 5a); the achiral bisborylation product **10aa** was formed in less than 5%. The enantiomeric ratio is therefore lower than that for the standard protocol with 1.6 equiv. of **5a** where an e.r. of 97:3 was obtained. This result implies that the desymmetrization may not be the sole factor in controlling the enantioselectivity (*k*_R_:*k*_S_ ≈ 5.7:1). To verify this further, the racemic mixture of **7aa** was subjected to a reaction with 0.6 equiv. of B_2_pin_2_ (**5a**) under identical reaction conditions (Scheme 5b). This led to its kinetic resolution with (*R*)‐**7aa** being formed in 66% yield with e.r. = 72:28 along with achiral byproduct **10aa** in 18% yield; other byproducts are protodeborylated **7aa** and **10aa** in minute amounts as well as the fully defunctionalized biaryl in traces (not shown). Clearly, (*S*)‐**7aa** reacts faster than (*R*)‐**7aa**, and the observed selectivity factor is 16. Based on these findings, we conclude that a matched downstream kinetic resolution further enhances the stereochemical outcome of the desymmetrization (Scheme 5c).

**Scheme 5:**
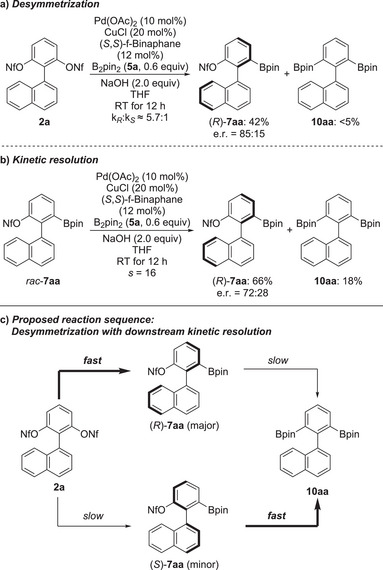
Control experiments (a and b) and proposed two‐step reaction sequence (c). *s* = selectivity factor.

We disclosed here a desymmetrization strategy for an enantioselective Miyaura‐type C(sp^2^)–B cross‐coupling to access highly enantioenriched, axially chiral biaryl boronic esters.^[^ ^43^ ^]^ This methodology hinges on palladium catalysis for the actual cross‐coupling event and copper catalysis for the activation of the B–B reagent to facilitate the transmetalation step. The overall enantioselectivity of the reaction is further enhanced by a downstream kinetic resolution process, in which the minor enantiomer of the chiral monoborylated product from the desymmetrization step is preferentially converted into the achiral bisborylated byproduct. The borylation reaction features a broad substrate scope and good functional‐group tolerance. The resulting axially chiral boronic esters can be readily transformed into other valuable axially chiral biaryl building blocks with virtually no loss of enantiopurity.

## Conflict of Interests

The authors declare no conflict of interest.

## Supporting information



Supporting Information

Supporting Information

## Data Availability

The data that support the findings of this study are available from the corresponding author upon reasonable request.
