# Supplementary material for: Enantioselective Miyaura Reaction by Desymmetrizing C(sp2)–B Cross‐Coupling of 1,1′‐Biaryl‐2,6‐diyl Bis(nonaflates)
Source: Angew Chem Int Ed Engl. 2025 Oct 13;64(47):e202515234. doi: 10.1002/anie.202515234 (PMC12624315; doi:10.1002/anie.202515234)
Supplement: Supplementary file 1 — Supporting Information [file ANIE-64-e202515234-s001.pdf]

Enantioselective Miyaura Reaction by Desymmetrizing C(sp^2)–B Cross-Coupling of 1,1’-Biaryl-2,6-diyl Bis(nonaflates)

Yao Xiao, Annika L. Bartelt, Elisabeth Irran, and Martin Oestreich*

Institut für Chemie, Technische Universität Berlin

Straße des 17. Juni 115, 10623 Berlin, Germany

martin.oestreich@tu-berlin.de

Supporting Information

Table of Contents

**1**	**General Information**	**S3**
**2**	**Optimization Study**	**S5**
**3**	**General Procedures**	**S7**
3.1	General Procedure for the Preparation of 1 (GP 1)	S7
3.2	General Procedure for the Synthesis of Racemic Products as HPLC Reference Compounds (GP 2)	S7
3.3	Typical Procedure for the Synthesis of Enantioenriched Products (GP 3)	S8
**4**	**Characterization Data**	**S9**
4.1	Characterization Data of Starting Materials	S9
4.2	Characterization Data of Chiral Products	S22
**5**	**1.0-mmol Scale Synthesis of (*R*)-7aa**	**S40**
**6**	**Transformations of (*R*)-7aa**	**S41**
6.1	Reaction with NaN_3	S41
6.2	Reaction with Imidazole	S42
6.3	Reaction with H_2O_2	S43
6.4	Reaction with PhI	S44
6.5	Control Experiments	S45
**7**	**Determination of the Absolute Configuration**	**S47**
**8**	**HPLC Traces**	**S57**
**9**	**NMR Spectra**	**S88**
**10**	**References**	**S283**

1 General Information

All reactions were performed in flame-dried glassware using conventional Schlenk techniques under a static pressure of nitrogen unless stated otherwise. Liquids and solutions were transferred with syringes. All metal salts were purchased from commercial suppliers and used as received. All solvents (CH_2Cl_2, toluene, Et_2O, MeOH, and THF *et al*.) were dried and purified following standard procedures. Technical grade solvents for extraction or chromatography (*n*-hexane, CH_2Cl_2, ethanol, ethyl acetate, and *n*-pentane *et al*.) were distilled prior to use. Analytical thin layer chromatography (TLC) was performed on ALUGRAM® Xtra SIL G/UV_{254} TLC-Sheets by Macherey-Nagel. Flash column chromatography was performed on silica gel 60 (40-63 µm, 230-400 mesh, ASTM) by Grace using the indicated solvents. ^1H, ^{13}C, ^{19}F, and ^{11}B NMR spectra were recorded in $CDCl_3$ on Bruker AV400 or AV500 instruments. Chemical shifts were reported in parts per million (ppm) and were referenced to the residual solvent resonance as the internal standard ($CHCl_3$: δ = 7.26 ppm for ^1H NMR and $CDCl_3$: δ = 77.00 ppm for ^{13}C NMR). All other nuclei (^{19}F and ^{11}B) were referenced in compliance with the unified scale for NMR chemical shifts as recommended by the IUPAC stating the chemical shift relative to $BF_3{\cdot}Et_2O$, CCl_3F, and Me_4Si.[S1] Data were reported as follows: chemical shift, multiplicity (s = singlet, d = doublet, t = triplet, q = quartet, sept = septet, m = multiplet), coupling constants (Hz), and integration. Gas liquid chromatography (GLC) was performed on an *Agilent Technologies 7820A* gas chromatograph equipped with a HP-5 capillary column (30 m × 0.32 mm, 0.25 µm film thickness) by *Agilent Technologies/CS-Chromatographie Service* using the following program: N_2 carrier gas, injection temperature 250 °C, detector temperature 300 °C, flow rate: 1.7 mL/min; temperature program: start temperature 40 °C, heating rate 10 °C/min, end temperature 280 °C for 10 min. Infrared (IR) spectra were recorded on an *Agilent Technologies Cary* 630 FT-IR spectrometer equipped with an ATR unit and the signals were reported in wave-numbers (cm^{-1}). Melting points (m.p.) were determined with a Stuart Scientific SMP20 melting point apparatus and were not corrected. Enantiomeric excesses were determined by analytical high performance liquid

chromatography (HPLC) analysis on an *Agilent Technologies* 1290 Infinity instrument with a chiral stationary phase using a *Daicel* Chiralcel AD-H column, *Daicel* Chiralcel OD-H column, *Daicel* Chiralcel IA, or a *Daicel* Chiralcel IC column (*n*heptane/*iso*propanol mixtures as solvent). Data for the single crystal structure determination were collected with an *Agilent SuperNova* diffractometer equipped with a CCD area Atlas detector and a mirror monochromator by utilizing Cu-K_{α} radiation (λ = 1.5418 Å). Software packages used: CrysAlis PRO for data collection, cell refinement, and data reduction,[S2] SHELXS-97 for structure solution,[S3] SHELXL-97 for structure refinement[S4], and Mercury 3.1.1[S5] for graphic. Single crystals are presented by Olex2-1.5. High-resolution mass spectra (HRMS) were obtained from the Analytical Facility at the *Institut für Chemie, Technische Universität Berlin* on a Thermo Fisher Scientific LTQ Orbitrap XL apparatus using ESI/LIFDI techniques with a linear ion trap analyzer. Optical rotations were measured on a *Schmidt & Haensch Polartronic* H532 Polarimeter with $[\alpha]_{\lambda}$values reported in 10^{-1} ($^{\circ}$ cm^2 g^{-1}); with the concentration *c* in g/100 mL and λ indicated.

2 Optimization Study

Table S1. Optimization of the reaction conditions.[a]

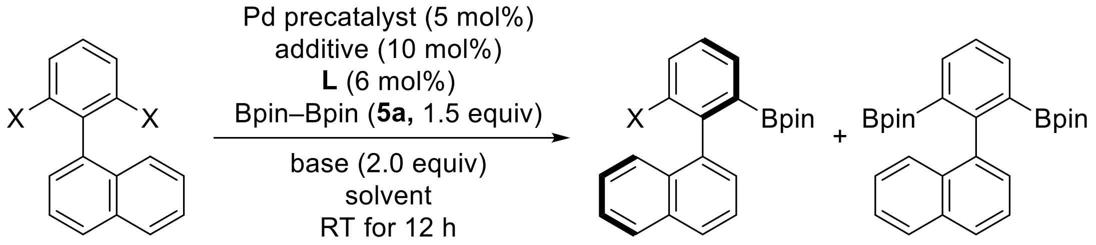

1a (X = OTf), **2a** (X = ONf), **3a** (X = OTs), and **4a** (X=Br)

6aa–9aa

10aa

Entry	X	ligand	precatalyst	additive	base	solvent	yield (%)[b]	e.r.[c]	**10aa** yield (%)[b]
1	OTf (**1a**)	**L1**	$Pd(OAc)_2$	—	K_3PO_4	THF	trace	—	—
2	OTf (**1a**)	**L2**	$Pd(OAc)_2$	—	K_3PO_4	THF	21 (**6aa**)	69:31 (**6aa**)	<10
3	OTf (**1a**)	**L3**	$Pd(OAc)_2$	—	K_3PO_4	THF	35 (**6aa**)	57:43 (**6aa**)	<10
4	OTf (**1a**)	**L4**	$Pd(OAc)_2$	—	K_3PO_4	THF	24 (**6aa**)	76:24 (**6aa**)	<10
5	OTf (**1a**)	**L5**	$Pd(OAc)_2$	—	K_3PO_4	THF	43 (**6aa**)	76:24 (**6aa**)	<10
6	OTf (**1a**)	**L5**	$Pd(OAc)_2$	CuCl	K_3PO_4	THF	56 (**6aa**)	89:11 (**6aa**)	<10
7	OTf (**1a**)	**L5**	—	CuCl	K_3PO_4	THF	— (**6aa**)	—	—
8	OTf (**1a**)	**L5**	$Pd(OAc)_2$	—	K_3PO_4	THF	43 (**6aa**)	76:24 (**6aa**)	<10
9	OTf (**1a**)	**L5**	$Pd(acac)_2$	CuCl	K_3PO_4	THF	50 (**6aa**)	84:16 (**6aa**)	<10
10	OTf (**1a**)	**L5**	$Pd_2(dba)_3$	CuCl	K_3PO_4	THF	38 (**6aa**)	86:14 (**6aa**)	<10
11	OTf (**1a**)	**L5**	$Pd(OAc)_2$	CuTc	K_3PO_4	THF	60 (**6aa**)	80:20 (**6aa**)	<10
12	OTf (**1a**)	**L5**	$Pd(OAc)_2$	CuI	K_3PO_4	THF	55 (**6aa**)	85:15 (**6aa**)	<10
13	OTf (**1a**)	**L5**	$Pd(OAc)_2$	CuCl	NaOH	THF	80 (**6aa**)	92:8 (**6aa**)	<10
14	OTf (**1a**)	**L5**	$Pd(OAc)_2$	CuCl	KOH	THF	77 (**6aa**)	92:8 (**6aa**)	<10
15	ONf (**2a**)	**L5**	$Pd(OAc)_2$	CuCl	NaOH	THF	86 (**7aa**)	94:6 (**7aa**)	<10
16	OTs (**3a**)	**L5**	$Pd(OAc)_2$	CuCl	NaOH	THF	— (**8aa**)	—	—
17	Br (**4a**)	**L5**	$Pd(OAc)_2$	CuCl	NaOH	THF	— (**9aa**)	—	—
18[d]	ONf (**2a**)	**L5**	$Pd(OAc)_2$	CuCl	NaOH	THF	82 (**7aa**)	95:5 (**7aa**)	12
19[d,e]	ONf (**2a**)	**L5**	$Pd(OAc)_2$	CuCl	NaOH	THF	77 (67)[f] (**7aa**)	97:3 (**7aa**)	16

[a] Reactions were performed on a 0.10 mmol scale under argon atmosphere. [b] Yields were determined by ^1H NMR spectroscopy. [c] Enantiomeric ratio was determined by HPLC analysis on a chiral stationary phase. [d] 1.6 equiv of **5 a** was used. [e] 10 mol% of $Pd(OAc)_2$, 12 mol% of (*S*,*S*)-f-Binaphane and 20 mol% of CuCl were used. [f] 67 % isolated yield on a 0.10 mmol scale after purification on silica gel.

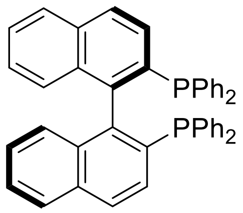

L1

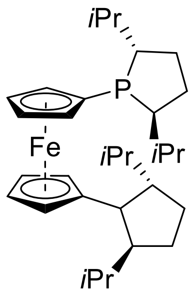

L2

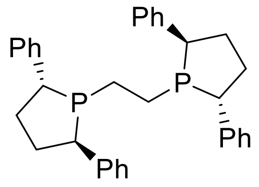

L3

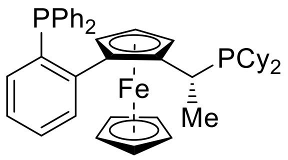

L4

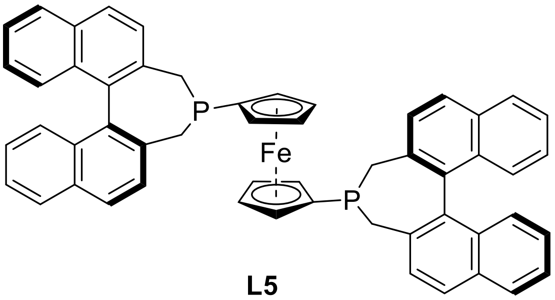

L5

3 General Procedures

3.1 General Procedure for the Preparation of 1 (GP 1)

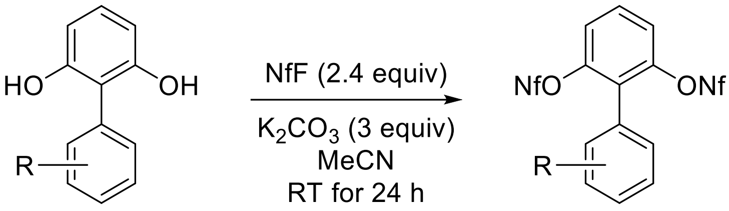

1'

2

According to a reported procedure[S6]: Perfluorobutanesulfonyl fluoride (7.2 mmol, 2.4 equiv) was added dropwise to a solution of **1'** (3.0 mmol, 1.0 equiv) and K_2CO_3 (9.0 mmol, 3.0 equiv) in dry acetonitrile. After stirring at room temperature for 24 hours (monitored by TLC), the reaction mixture was filtered through a celite pad. The organic layer was concentrated. The residue was purified by silica gel on column chromatography (ethyl acetate/cyclohexane = 1/100) to give compound **2**.

3.2 General Procedure for the Synthesis of Racemic Products as HPLC Reference Compounds (GP 2)

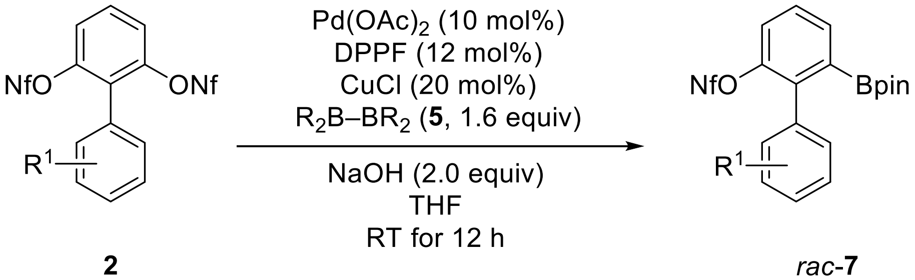

2

rac-**7**

To a flame-dried Schlenk tube equipped with a septum and a magnetic stir bar were added $Pd(OAc)_2$ (10 mol%), CuCl (20 mol%) and *rac*-DPPF (12 mol%). The tube was evacuated under high vacuum and backfilled with nitrogen gas (3 times). THF (1 mL) was added to the tube, and the resulting mixture was stirred under room temperature for 30 minutes. Then nonaflates **2** (0.10 mmol, 1.0 equiv), B–B reagent **5** (1.6 equiv) and NaOH (2.0 equiv) were successively added. The reaction was stirred at room temperature for 12 h (monitored by TLC). Then, the reaction mixture was filtered through a thin silicon pad. The organic layer was concentrated. The reaction mixture was purified by flash column chromatography on silica gel or preparative thin-layer chromatography with ethyl acetate and hexane (or cyclohexane) as the eluent to afford the product *rac*-**7**.

3.3 Typical Procedure for the Synthesis of Enantioenriched Products (GP 3)

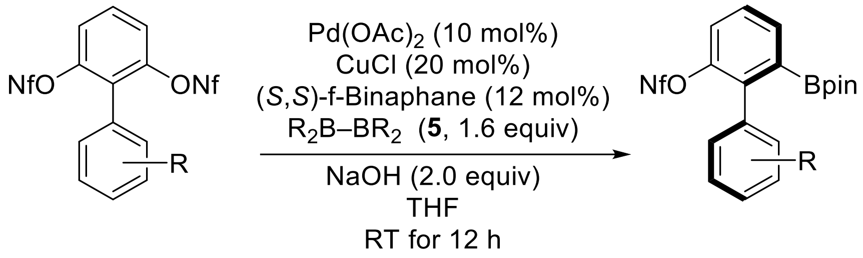

2 **7**

To a flame-dried Schlenk tube equipped with a septum and a magnetic stir bar were added $Pd(OAc)_2$ (10 mol%), CuCl (20 mol%) and (*S*,*S*)-f-Binaphane (12 mol%). The tube was evacuated under high vacuum and backfilled with nitrogen gas (3 times). THF (1 mL) was added to the tube, and the resulting mixture was stirred under room temperature for 30 minutes. Then nonaflates **2** (0.10 mmol, 1.0 equiv), B–B reagent **5** (1.6 equiv) and NaOH (2.0 equiv) were successively added. The reaction was stirred at room temperature for 12 h to 36 h (monitored by TLC). Then, the reaction mixture was filtered through a thin silicon pad. The organic layer was concentrated. The reaction mixture was purified by flash column chromatography on silica gel or preparative thin-layer chromatography with ethyl acetate and hexane (or pentane) as the eluent to afford the product **7**.

4 Characterization Data

4.1 Characterization Data of Starting Materials

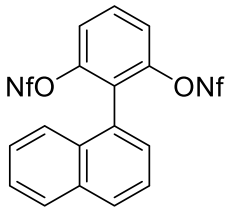

2a
$C_{24}H_{10}F_{18}O_6S_2$
M = 799.96 g/mol

2-(naphthalen-1-yl)-1,3-phenylene bis(1,1,2,2,3,3,4,4,4-nonafluorobutane-1-sulfonate) (**2a**): Prepared from 2-(naphthalen-1-yl)benzene-1,3-diol (708 mg, 3.00 mmol, 1.00 equiv) according to **GP 1**. **2a** was afforded as colorless oil (1.72 g, 73% yield). **^{1}H NMR** (500 MHz, $CDCl_3$, 298 K) δ = 8.00 (d, *J* = 8.3 Hz, 1H), 7.92 (d, *J* = 8.2 Hz, 1H), 7.68–7.64 (m, 1H), 7.60–7.56 (m, 3H), 7.53–7.49 (m, 2H), 7.47–7.44 (m, 1H), 7.33 (d, *J* = 8.4 Hz, 1H) ppm. **^{13}C{^{1}H} NMR** (126 MHz, $CDCl_3$, 298 K) δ = 148.5, 133.5, 131.5, 130.3, 130.3, 129.6, 129.5, 128.4, 126.8, 126.2, 125.8, 124.8, 124.6, 121.8 (nonaflate group not listed) ppm. **^{19}F NMR** (471 MHz, $CDCl_3$, 298 K) δ = −80.72–(−80.86) (m), −109.78–(−109.85) (m), −121.14–(−121.18) (m), −126.00–(−126.12) (m) ppm. **HRMS** (APCI) m/z: $[M]^+$ calcd for $C_{24}H_{10}F_{18}O_6S_2^+$ 799.9626; found 799.9619.

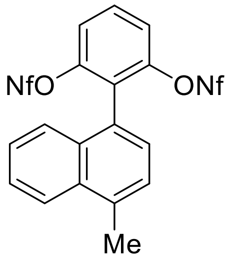

2b
$C_{25}H_{12}F_{18}O_6S_2$
M = 813.98 g/mol

2-(4-methylnaphthalen-1-yl)-1,3-phenylene bis(1,1,2,2,3,3,4,4,4-nonafluorobutane-1-sulfonate) (**2b**): Prepared from 2-(4-methylnaphthalen-1-yl)benzene-1,3-diol (750 mg, 3.00 mmol, 1.00 equiv) according to **GP 1**. **2b** was afforded as white solid (1.93 g, 79% yield). **M.P.** 46–49 °C. **^{1}H NMR** (400 MHz, $CDCl_3$, 298 K) δ = 8.10 (d, *J* = 8.5 Hz, 1H), 7.64–7.52 (m, 4H), 7.48–7.40 (m, 3H), 7.36 (d, *J* = 8.4 Hz, 1H), 2.77 (s, 3H) ppm. **^{13}C{^{1}H} NMR** (101 MHz, $CDCl_3$, 298 K) δ = 148.6, 137.0, 132.7, 131.5, 130.1, 130.0, 129.2, 126.4, 126.0, 125.6, 125.2, 124.6, 124.0, 121.8, 19.5 (nonaflate group not listed) ppm. **^{19}F NMR** (471 MHz, $CDCl_3$, 298 K) δ =

−80.81–(−80.00) (m), −109.85 (t, *J* = 14.3 Hz), −121.21–(−121.24) (m), −126.10–(−126.19) (m) ppm. **HRMS** (APCI) m/z: [M]$^+$ calcd for $C_{25}H_{12}F_{18}O_6S_2^+$ 813.9782; found 813.9779.

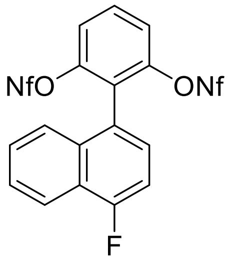

2c
$C_{24}H_9F_{19}O_6S_2$
M = 817.95 g/mol

2-(4-fluoronaphthalen-1-yl)-1,3-phenylene bis(1,1,2,2,3,3,4,4,4-nonafluorobutane-1-sulfonate) (**2c**): Prepared from 2-(4-fluoronaphthalen-1-yl)benzene-1,3-diol (762 mg, 3.00 mmol, 1.00 equiv) according to **GP 1**. **2c** was afforded as white solid (1.82 g, 74% yield). **M.P.** 38–39 °C. **^{1}H NMR** (500 MHz, $CDCl_3$, 298 K) δ = 8.12 (dd, *J* = 8.5, 2.6 Hz, 1H), 7.61–7.57 (m, 1H), 7.52–7.42 (m, 4H), 7.36 (s, 1H), 7.24 (d, *J* = 8.6 Hz, 1H), 7.19–7.16 (m, 1H) ppm. **^{13}C{^{1}H} NMR** (126 MHz, $CDCl_3$, 298 K) δ = 160.0 (d, *J* = 255.8 Hz), 148.5, 133.1 (d, *J* = 4.9 Hz), 130.5, 129.7 (d, *J* = 9.1 Hz), 129.0, 127.8, 126.6, 124.7, 123.8 (d, *J* = 16.5 Hz), 121.9, 121.8 (d, *J* = 3.8 Hz), 121.0 (d, *J* = 5.4 Hz), 108.8 (d, *J* = 21.0 Hz) (nonaflate group not listed) ppm. **^{19}F NMR** (471 MHz, $CDCl_3$, 298 K) δ = −80.66–(−80.85) (m), −109.77 (t, *J* = 14.2 Hz), −119.70, −121.16, −126.02–(−126.09) (m) ppm. **HRMS** (APCI) m/z: [M]$^+$ calcd for $C_{24}H_9F_{19}O_6S_2^+$ 817.9532; found 817.9532.

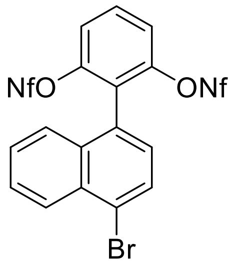

2d
$C_{24}H_9BrF_{18}O_6S_2$
M = 877.87 g/mol

2-(4-bromonaphthalen-1-yl)-1,3-phenylene bis(1,1,2,2,3,3,4,4,4-nonafluorobutane-1-sulfonate) (**2d**)**:** Prepared from 2-(4-bromonaphthalen-1-yl)benzene-1,3-diol (942 mg, 3.00 mmol, 1.00 equiv) according to **GP 1**. **2d** was afforded as white solid (1.98 g, 75% yield). **M.P.** 67–69 °C. **^{1}H NMR** (500 MHz, $CDCl_3$, 298 K) δ = 8.35 (d, *J* = 8.5 Hz, 1H), 7.90 (dd, *J* = 7.6, 1.2 Hz, 1H), 7.69 (ddd, *J* = 9.0, 7.8, 1.3 Hz, 1H), 7.63 (ddt, *J* = 8.2, 6.8, 1.2 Hz, 1H), 7.58–7.57 (m, 2H), 7.50 (ddt, *J* = 8.3, 6.8, 1.3 Hz, 1H), 7.33 (td, *J* = 5.1, 2.7 Hz, 2H) ppm. **^{13}C{^{1}H} NMR** (126 MHz, $CDCl_3$, 298 K) δ = 148.3, 132.6, 132.1, 130.7, 129.6, 129.0, 128.7, 127.8, 127.7, 127.7,

125.9, 125.5, 125.2, 121.9 (nonaflate group not observed) ppm. **^{19}F NMR** (471 MHz, $CDCl_3$, 298 K) δ = −80.77–(−80.81) (m), −109.60–(−109.67) (m), −121.12–(−121.15) (m), −125.95–(−126.07) (m) ppm. **HRMS** (APCI) m/z: $[M]^+$ calcd for $C_{24}H_9BrF_{18}O_6S_2^+$ 877.8731; found 877.8723.

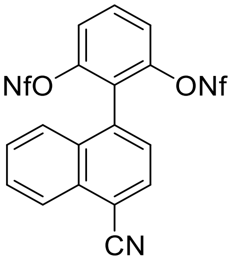

2e
$C_{25}H_9F_{18}NO_6S_2$
M = 825.96 g/mol

2-(4-cyanonaphthalen-1-yl)-1,3-phenylene bis(1,1,2,2,3,3,4,4,4-nonafluorobutane-1-sulfonate) (**2e**): Prepared from 4-(2,6-dihydroxyphenyl)-1-naphthonitrile (783 mg, 3.00 mmol, 1.00 equiv) according to **GP 1**. **2e** was afforded as white solid (1.51 g, 61% yield). **M.P.** 94–96 °C. **^{1}H NMR** (500 MHz, $CDCl_3$, 298 K) δ = 8.37–8.35 (m, 1H), 8.02 (dd, J = 7.4, 1.7 Hz, 1H), 7.76–7.72 (m, 2H), 7.62–7.56 (m, 4H), 7.45 (dd, J = 8.6, 3.5 Hz, 1H) ppm. **^{13}C{^{1}H} NMR** (126 MHz, $CDCl_3$, 298 K) δ = 147.9, 132.4, 131.6, 131.4, 131.3, 129.0, 128.5, 127.9, 125.7, 125.6, 122.1, 117.1, 112.5 (nonaflate group not listed) ppm. **^{19}F NMR** (471 MHz, $CDCl_3$, 298 K) δ = −80.84–(−80.94) (m), −109.65, −121.20, −126.09–(−126.11) (m) ppm. **HRMS** (APCI) m/z: $[M]^+$ calcd for $C_{25}H_9F_{18}NO_6S_2^+$ 824.9578; found 824.9577.

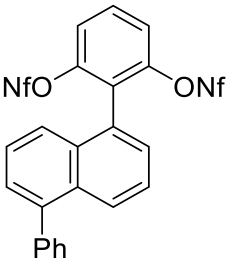

2f
$C_{30}H_{14}F_{18}O_6S_2$
M = 875.99 g/mol

2-(5-phenylnaphthalen-1-yl)-1,3-phenylene bis(1,1,2,2,3,3,4,4,4-nonafluorobutane-1-sulfonate) (2f): Prepared from 2-(5-phenylnaphthalen-1-yl)benzene-1,3-diol (936 mg, 3.00 mmol, 1.00 equiv) according to **GP 1**. **2f** was afforded as white solid (1.87 g, 71% yield). **M.P.** 37–41 °C. **^{1}H NMR** (500 MHz, $CDCl_3$, 298 K) δ = 8.12 (dd, J = 8.6, 3.9 Hz, 1H), 7.66–7.51 (m, 12H), 7.41–7.40 (m, 1H) ppm. **^{13}C{^{1}H} NMR** (126 MHz, $CDCl_3$, 298 K) δ = 148.6, 140.9, 140.7, 131.9, 131.9, 130.3, 130.2, 129.9, 129.5, 128.6, 128.3, 127.4, 127.3, 126.2, 126.0, 124.9, 124.3, 121.9 (nonaflate group not listed) ppm. **^{19}F NMR** (471 MHz, $CDCl_3$, 298 K) δ = −80.93

(t, *J* = 10.0 Hz), −109.79 (t, *J* = 14.3 Hz), −121.16–(−121.17) (m), −126.10 (t, *J* = 13.9 Hz) ppm. **HRMS** (APCI) m/z: [M]$^+$ calcd for $C_{30}H_{14}F_{18}O_6S_2^+$ 875.9939; found 875.9935.

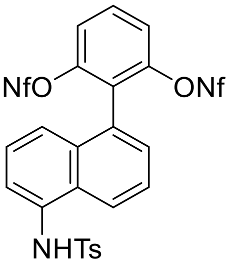

2g
$C_{31}H_{17}F_{18}NO_8S_3$
M = 968.98 g/mol

2-(5-((4-methylphenyl)sulfonamido)naphthalen-1-yl)-1,3-phenylene bis(1,1,2,2,3,3,4,4,4-nonafluorobutane-1-sulfonate) (2g): Prepared from *N*-(5-(2,6-dihydroxyphenyl)naphthalen-1-yl)-4-methylbenzenesulfonamide (1.22 g, 3.00 mmol, 1.00 equiv) according to **GP 1**. **2g** was afforded as white solid (1.86 g, 64% yield). **M.P.** 110–112 °C. **^{1}H NMR** (500 MHz, $CDCl_3$, 298 K) δ = 7.87–7.86 (m, 1H), 7.69–7.66 (m, 1H), 7.55–7.54 (m, 5H), 7.44–7.43 (m, 2H), 7.39–7.36 (m, 1H), 7.26–7.22 (m, 1H), 7.10–7.05 (m, 3H), 2.29 (s, 3H) ppm. **^{13}C{^{1}H} NMR** (126 MHz, $CDCl_3$, 298 K) δ = 148.4, 143.8, 135.9, 132.2, 131.9, 130.6, 130.0, 129.5, 129.5, 129.1, 127.1, 126.5, 126.4, 125.4, 124.3, 124.1, 123.8, 121.9, 21.2 (nonaflate group not observed) ppm. **^{19}F NMR** (471 MHz, $CDCl_3$, 298 K) δ = −80.73–(−80.75) (m), −109.66–(−109.73) (m), −121.06–(−121.07) (m), −125.91–(−125.98) (m) ppm. **HRMS** (APCI) m/z: [M+H]$^+$ calcd for $C_{31}H_{18}F_{18}NO_8S_3^+$ 969.9902; found 969.9897.

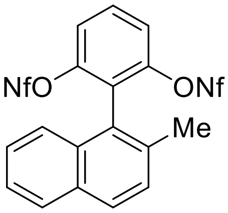

2h
$C_{25}H_{12}F_{18}O_6S_2$
M = 813.98 g/mol

2-(2-methylnaphthalen-1-yl)-1,3-phenylene bis(1,1,2,2,3,3,4,4,4-nonafluorobutane-1-sulfonate) (2h): Prepared from 2-(2-methylnaphthalen-1-yl)benzene-1,3-diol (750 mg, 3.00 mmol, 1.00 equiv) according to **GP 1**. **2h** was afforded as colorless oil (2.12 g, 87% yield). **^{1}H NMR** (500 MHz, $CDCl_3$, 298 K) δ = 7.90 (d, *J* = 8.5 Hz, 1H), 7.85 (d, *J* = 8.0 Hz, 1H), 7.68 (dd, *J* = 9.1, 7.6 Hz, 1H), 7.58 (d, *J* = 8.3 Hz, 2H), 7.45–7.38 (m, 3H), 7.19 (d, *J* = 8.3 Hz, 1H), 2.28 (s, 3H) ppm. **^{13}C{^{1}H} NMR** (126 MHz, $CDCl_3$, 298 K) δ = 148.5, 136.3, 132.0, 131.9, 130.4, 130.1, 128.7, 128.1, 128.1, 126.7, 125.3, 124.5, 123.8, 121.9, 20.1 (nonaflate group not observed) ppm. **^{19}F NMR** (471 MHz, $CDCl_3$, 298 K) δ = −80.83–(−80.87) (m), −109.93–

(−109.99) (m), −121.17–(−121.21) (m), −126.00–(−126.10) (m) ppm. **HRMS** (APCI) m/z: $[M]^+$ calcd for $C_{25}H_{12}F_{18}O_6S_2^+$ 813.9782; found 813.9775.

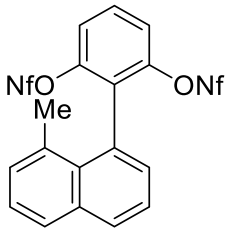

2i
$C_{25}H_{12}F_{18}O_6S_2$
M = 813.98 g/mol

2-(8-methylnaphthalen-1-yl)-1,3-phenylene bis(1,1,2,2,3,3,4,4,4-nonafluorobutane-1-sulfonate) (2i): Prepared from 2-(8-methylnaphthalen-1-yl)benzene-1,3-diol (750 mg, 3.00 mmol, 1.00 equiv) according to **GP 1**. **2i** was afforded as colorless oil (2.00 g, 82% yield). **^{1}H NMR** (500 MHz, $CDCl_3$, 298 K) δ = 8.01 (dd, *J* = 8.3, 1.4 Hz, 1H), 7.82 (d, *J* = 8.2 Hz, 1H), 7.60–7.50 (m, 4H), 7.42–7.39 (m, 2H), 7.30 (d, *J* = 6.9 Hz, 1H), 2.09 (s, 3H) ppm. **^{13}C{^{1}H} NMR** (126 MHz, $CDCl_3$, 298 K) δ = 148.4, 135.1, 133.6, 133.4, 131.7, 131.3, 131.1, 130.5, 130.0, 128.1, 125.8, 124.8, 124.0, 121.3, 22.7 (nonaflate group not listed) ppm. **^{19}F NMR** (471 MHz, $CDCl_3$, 298 K) δ = −81.00 (t, *J* = 9.8 Hz), −109.83–(−109.98) (m), −121.20–(−121.25) (m), −126.08–(−126.20) (m) ppm. **HRMS** (APCI) m/z: $[M]^+$ calcd for $C_{25}H_{12}F_{18}O_6S_2^+$ 813.9782; found 813.9781.

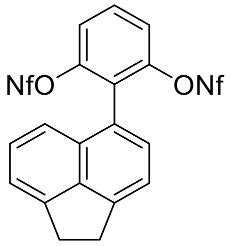

2j
$C_{26}H_{12}F_{18}O_6S_2$
M = 825.98 g/mol

2-(1,2-dihydroacenaphthylen-5-yl)-1,3-phenylene bis(1,1,2,2,3,3,4,4,4-nonafluorobutane-1-sulfonate) (2j): Prepared from 2-(1,2-dihydroacenaphthylen-5-yl)benzene-1,3-diol (786 mg, 3.00 mmol, 1.00 equiv) according to **GP 1**. **2j** was afforded as white solid (1.49 g, 60% yield). **M.P**. 58–60 °C. **^{1}H NMR** (500 MHz, $CDCl_3$, 298 K) δ = 7.61–7.41 (m, 6H), 7.34–7.31 (m, 1H), 7.15–7.11 (m, 1H), 3.46 (s, 4H) ppm. **^{13}C{^{1}H} NMR** (126 MHz, $CDCl_3$, 298 K) δ = 148.8, 146.3, 139.2, 131.3, 130.1, 129.9, 129.7, 128.7, 121.8, 121.1, 119.8, 119.8, 118.5, 30.4, 30.3 (nonaflate group not listed) ppm. **^{19}F NMR** (471 MHz, $CDCl_3$, 298 K) δ = −80.85–(−81.11) (m), −109.82 (t, *J* = 14.6 Hz), −121.19, −126.10–(−126.17) (m) ppm. **HRMS** (APCI) m/z: $[M]^+$ calcd for $C_{26}H_{12}F_{18}O_6S_2^+$ 825.9782; found 825.9777.

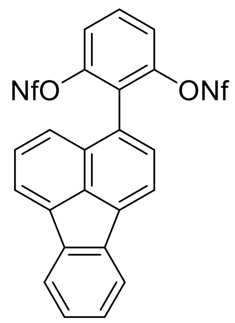

2k
$C_{30}H_{12}F_{18}O_6S_2$
M = 873.98 g/mol

2-(fluoranthen-3-yl)-1,3-phenylene bis(1,1,2,2,3,3,4,4,4-nonafluorobutane-1-sulfonate) (2k): Prepared from 2-(fluoranthen-3-yl)benzene-1,3-diol (930 mg, 3.00 mmol, 1.00 equiv) according to **GP 1**. **2k** was afforded as white solid (1.89 g, 72% yield). **M.P**. 91–94 °C. **^{1}H NMR** (500 MHz, $CDCl_3$, 298 K) δ = 8.02 (dd, *J* = 7.1, 2.5 Hz, 1H), 7.94–7.90 (m, 3H), 7.69–7.63 (m, 2H), 7.58 (dd, *J* = 8.3, 2.5 Hz, 3H), 7.41 (dd, *J* = 6.1, 2.9 Hz, 2H), 7.34 (dd, *J* = 8.4, 2.4 Hz, 1H) ppm. **^{13}C{^{1}H} NMR** (126 MHz, $CDCl_3$, 298 K) δ = 148.5, 139.9, 139.1, 139.0, 137.4, 132.5, 131.4, 130.4, 129.0, 128.8, 128.7, 128.2, 127.8, 125.4, 124.3, 122.0, 121.8, 121.6, 120.5, 119.1 (nonaflate group not observed) ppm. **^{19}F NMR** (471 MHz, $CDCl_3$, 298 K) δ = −80.64–(−80.83) (m), −109.63 (t, *J* = 14.1 Hz), −121.05, −125.97–(−126.04) (m) ppm. **HRMS** (APCI) m/z: $[M]^+$ calcd for $C_{30}H_{12}F_{18}O_6S_2^+$ 873.9782; found 873.9775.

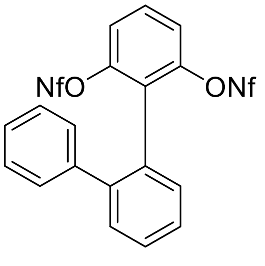

2l
$C_{26}H_{12}F_{18}O_6S_2$
M = 825.98 g/mol

[1,1':2',1''-terphenyl]-2,6-diyl bis(1,1,2,2,3,3,4,4,4-nonafluorobutane-1-sulfonate) (2l): Prepared from [1,1':2',1''-terphenyl]-2,6-diol (786 mg, 3.00 mmol, 1.00 equiv) according to **GP 1**. **2l** was afforded as colorless oil (2.08 g, 84% yield). **^{1}H NMR** (500 MHz, $CDCl_3$, 298 K) δ = 7.59–7.56 (m, 1H), 7.55–7.41 (m, 4H), 7.32–7.26 (m, 2H), 7.23–7.22 (m, 3H), 7.18–7.16 (m, 2H) ppm. **^{13}C{^{1}H} NMR** (126 MHz, $CDCl_3$, 298 K) δ = 147.9, 142.7, 140.4, 131.8, 130.6, 130.4, 130.1, 129.7, 128.9, 127.9, 127.2, 126.8, 121.4 (nonaflate group not listed) ppm. **^{19}F NMR** (471 MHz, $CDCl_3$, 298 K) δ = −80.71–(−81.35) (m), −109.16–(−110.50) (m), −120.99–(−121.43) (m), −125.95–(−126.02) (m) ppm. **HRMS** (APCI) m/z: $[M]^+$ calcd for $C_{26}H_{12}F_{18}O_6S_2^+$ 825.9782; found 825.9781.

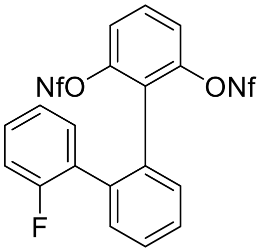

2m
$C_{26}H_{11}F_{19}O_6S_2$
M = 843.97 g/mol

2''-fluoro-[1,1':2',1''-terphenyl]-2,6-diyl bis(1,1,2,2,3,3,4,4,4-nonafluorobutane-1-sulfonate) (2m): Prepared from 2''-fluoro-[1,1':2',1''-terphenyl]-2,6-diol (840 mg, 3.00 mmol, 1.00 equiv) according to **GP 1**. **2m** was afforded as colorless oil (1.69 g, 67% yield). **^{1}H NMR** (500 MHz, $CDCl_3$, 298 K) δ = 7.61–7.57 (m, 4H), 7.43–7.38 (m, 3H), 7.33–7.26 (m, 2H), 7.12–7.09 (m, 1H), 6.99 (t, *J* = 9.2 Hz, 1H) ppm. **^{13}C{^{1}H} NMR** (126 MHz, $CDCl_3$, 298 K) δ = 159.6 (d, *J* = 247.5 Hz), 148.1, 136.7, 131.8, 131.2, 130.0, 130.0, 129.9, 129.7 (d, *J* = 8.0 Hz), 128.4, 127.9, 127.8 (d, *J* = 15.5 Hz), 123.7 (d, *J* = 3.6 Hz), 121.1, 115.4 (d, *J* = 22.5 Hz) (nonaflate group not listed) ppm. **^{19}F NMR** (471 MHz, $CDCl_3$, 298 K) δ = −81.40 (t, *J* = 9.9 Hz), −109.38–(−110.75) (m), −115.14, −121.24, −126.28–(−126.35) (m) ppm. **HRMS** (APCI) m/z: $[M]^+$ calcd for $C_{26}H_{11}F_{19}O_6S_2^+$ 843.9688; found 843.9686.

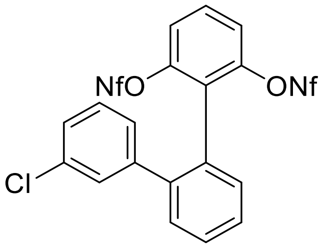

2n
$C_{26}H_{11}ClF_{18}O_6S_2$
M = 859.94 g/mol

3''-chloro-[1,1':2',1''-terphenyl]-2,6-diyl bis(1,1,2,2,3,3,4,4,4-nonafluorobutane-1-sulfonate) (2n): Prepared from 3''-chloro-[1,1':2',1''-terphenyl]-2,6-diol (888 mg, 3.00 mmol, 1.00 equiv) according to **GP 1**. **2n** was afforded as white solid (2.14 g, 83% yield). **M.P**. 31–35 °C. **^{1}H NMR** (500 MHz, $CDCl_3$, 298 K) δ = 7.57 (td, *J* = 6.9, 2.6 Hz, 1H), 7.52–7.42 (m, 4H), 7.32 (d, *J* = 8.5 Hz, 2H), 7.20–7.17 (m, 2H), 7.12 (t, *J* = 7.8 Hz, 1H), 7.00–6.99 (m, 1H) ppm. **^{13}C{^{1}H} NMR** (126 MHz, $CDCl_3$, 298 K) δ = 147.7, 142.0, 141.1, 133.9, 131.9, 130.2, 130.2, 130.1, 130.0, 129.1, 127.7, 127.3, 126.9, 126.8, 121.6 (nonaflate group not listed) ppm. **^{19}F NMR** (471 MHz, $CDCl_3$, 298 K) δ = −80.53–(−81.14) (m), −109.05–(−110.43) (m), −120.94, −125.85–(−125.89) (m) ppm. **HRMS** (APCI) m/z: $[M]^+$ calcd for $C_{26}H_{11}ClF_{18}O_6S_2^+$ 859.9393; found 859.9391.

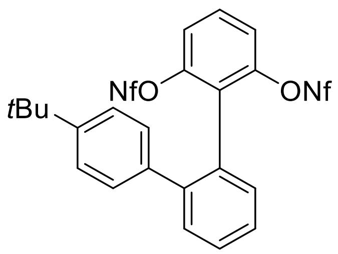

2o
$C_{30}H_{20}F_{18}O_6S_2$
M = 882.04 g/mol

4''-(*tert*-butyl)-[1,1':2',1''-terphenyl]-2,6-diyl bis(1,1,2,2,3,3,4,4,4-nonafluorobutane-1-sulfonate) (2o): Prepared from 4''-(*tert*-butyl)-[1,1':2',1''-terphenyl]-2,6-diol (954 mg, 3.00 mmol, 1.00 equiv) according to **GP 1**. **2o** was afforded as white solid (1.79 g, 68% yield). **M**.**P**. 83–84 °C. **^{1}H NMR** (500 MHz, $CDCl_3$, 298 K) δ = 7.55–7.51 (m, 2H), 7.47–7.40 (m, 3H), 7.31–7.30 (m, 2H), 7.24–7.23 (m, 2H), 7.08–7.07 (m, 2H), 1.28 (s, 9H) ppm. **^{13}C{^{1}H} NMR** (126 MHz, $CDCl_3$, 298 K) δ = 150.1, 147.9, 142.5, 137.3, 131.8, 130.7, 130.4, 130.0, 129.6, 128.5, 126.9, 126.7, 124.8, 121.4, 34.4, 31.2 (nonaflate group not listed) ppm. **^{19}F NMR** (471 MHz, $CDCl_3$, 298 K) δ = −80.82 (t, *J* = 9.9 Hz), −109.19–(−110.57) (m), −120.98, −125.92–(−125.98) (m) ppm. **HRMS** (APCI) m/z: $[M]^+$ calcd for $C_{30}H_{20}F_{18}NO_6S_2^+$ 882.0408; found 882.0409.

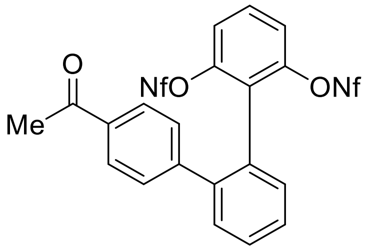

2p
$C_{28}H_{14}F_{18}O_7S_2$
M = 867.99 g/mol

4''-acetyl-[1,1':2',1''-terphenyl]-2,6-diyl bis(1,1,2,2,3,3,4,4,4-nonafluorobutane-1-sulfonate) (2p): Prepared from 1-(2'',6''-dihydroxy-[1,1':2',1''-terphenyl]-4-yl)ethan-1-one (912 mg, 3.00 mmol, 1.00 equiv) according to **GP 1**. **2p** was afforded as white solid (625 mg, 24% yield). **M**.**P**. 52–56 °C. **^{1}H NMR** (500 MHz, $CDCl_3$, 298 K) δ = 7.73 (dt, *J* = 8.4, 2.1 Hz, 2H), 7.52–7.35 (m, 5H), 7.23 (dd, *J* = 8.3, 2.5 Hz, 2H), 7.17 (dt, *J* = 8.2, 2.2 Hz, 2H), 2.48 (s, 3H) ppm. **^{13}C{^{1}H} NMR** (126 MHz, $CDCl_3$, 298 K) δ = 197.7, 147.7, 145.2, 141.4, 135.8, 132.1, 130.3, 130.2, 130.1, 129.1, 128.0, 127.9, 126.7, 121.6, 26.5 (nonaflate group not listed) ppm. **^{19}F NMR** (471 MHz, $CDCl_3$, 298 K) δ = −80.72 (t, *J* = 10.0 Hz), −109.56–(−109.79) (m), −120.93–(−120.94) (m), −125.88 (t, *J* = 10.0 Hz) ppm. **HRMS** (APCI) m/z: $[M+H]^+$ calcd for $C_{28}H_{15}F_{18}O_7S_2^+$ 868.9966; found 868.9964.

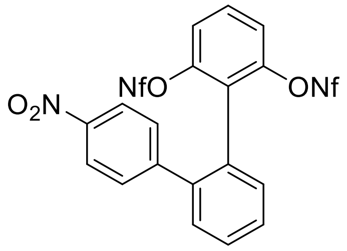

2q
$C_{26}H_{11}F_{18}NO_8S_2$
M = 870.96 g/mol

4''-nitro-[1,1':2',1''-terphenyl]-2,6-diyl bis(1,1,2,2,3,3,4,4,4-nonafluorobutane-1-sulfonate) (2q): Prepared from 4''-nitro-[1,1':2',1''-terphenyl]-2,6-diol (921 mg, 3.00 mmol, 1.00 equiv) according to **GP 1**. **2q** was afforded as white solid (1.80 g, 69% yield). **M.P**. 50–52 °C. **^{1}H NMR** (500 MHz, $CDCl_3$, 298 K) δ = 8.10–8.08 (m, 2H), 7.62 (t, *J* = 7.5 Hz, 1H), 7.56 (dd, *J* = 8.6, 6.7 Hz, 1H), 7.52–7.47 (m, 3H), 7.35–7.33 (m, 4H) ppm. **^{13}C{^{1}H} NMR** (126 MHz, $CDCl_3$, 298 K) δ = 147.7, 147.1, 147.0, 140.3, 132.4, 130.5, 130.5, 130.3, 129.8, 129.8, 128.5, 126.8, 123.2, 121.9 (nonaflate group not listed) ppm. **^{19}F NMR** (471 MHz, $CDCl_3$, 298 K) δ = −80.83 (t, *J* = 9.8 Hz), −108.97–(−110.31) (m), −120.97, −125.91–(−125.98) (m) ppm. **HRMS** (APCI) m/z: $[M-O_2+H_3]^+$ calcd for $C_{26}H_{14}F_{18}NO_6S_2^+$ 841.9970; found 841.9966.

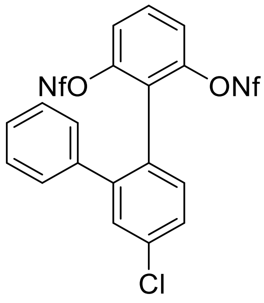

2r
$C_{26}H_{11}ClF_{18}O_6S_2$
M = 859.94 g/mol

4'-chloro-[1,1':2',1''-terphenyl]-2,6-diyl bis(1,1,2,2,3,3,4,4,4-nonafluorobutane-1-sulfonate) (2r): Prepared from 4'-chloro-[1,1':2',1''-terphenyl]-2,6-diol (888 mg, 3.00 mmol, 1.00 equiv) according to **GP 1**. **2r** was afforded as white solid (1.80 g, 69% yield). **M.P**. 54–55 °C. **^{1}H NMR** (500 MHz, $CDCl_3$, 298 K) δ = 7.40–7.39 (m, 1H), 7.34 (dt, *J* = 8.4, 1.9 Hz, 1H), 7.29 (t, *J* = 8.4 Hz, 1H), 7.25–7.23 (m, 1H), 7.17 (dd, *J* = 8.4, 1.9 Hz, 2H), 7.10–7.06 (m, 3H), 7.02–6.99 (m, 2H) ppm. **^{13}C{^{1}H} NMR** (126 MHz, $CDCl_3$, 298 K) δ = 147.8, 144.4, 139.1, 136.3, 133.1, 130.6, 130.1, 129.6, 128.7, 128.1, 127.8, 127.4, 125.4, 121.5 (nonaflate group not listed) ppm. **^{19}F NMR** (471 MHz, $CDCl_3$, 298 K) δ = −80.92–(−81.02) (m), −109.61–(−109.80) (m), −121.01, −125.99–(−126.02) (m) ppm. **HRMS** (APCI) m/z: $[M]^+$ calcd for $C_{26}H_{11}ClF_{18}O_6S_2^+$ 859.9393; found 859.9393.

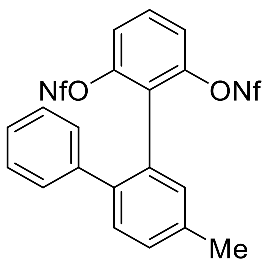

2s

$C_{27}H_{14}F_{18}O_6S_2$

M = 839.99 g/mol

5'-methyl-[1,1':2',1''-terphenyl]-2,6-diyl bis(1,1,2,2,3,3,4,4,4-nonafluorobutane-1-sulfonate) (2s): Prepared from 5'-methyl-[1,1':2',1''-terphenyl]-2,6-diol (600 mg, 3.00 mmol, 1.00 equiv) according to **GP 1**. **2s** was afforded as white solid (1.66 g, 66% yield). **M.P**. 35–37 °C. **^{1}H NMR** (500 MHz, $CDCl_3$, 298 K) δ = 7.44–7.35 (m, 3H), 7.29 (dd, *J* = 8.4, 2.3 Hz, 2H), 7.21–7.18 (m, 4H), 7.11– 7.10 (m, 2H), 2.42 (s, 3H) ppm. **^{13}C{^{1}H} NMR** (126 MHz, $CDCl_3$, 298 K) δ = 147.8, 140.3, 139.6, 137.0, 132.2, 130.7, 130.6, 130.2, 129.5, 128.8, 127.8, 126.9, 126.4, 121.3, 20.7 (nonaflate group not listed) ppm. **^{19}F NMR** (471 MHz, $CDCl_3$, 298 K) δ = −81.73 (t, *J* = 9.8 Hz), −109.73–(−110.00) (m), −121.00 (m), −125.92 (t, *J* = 9.8 Hz) ppm. **HRMS** (APCI) m/z: $[M]^+$ calcd for $C_{27}H_{14}F_{18}O_6S_2^+$ 839.9939; found 839.9935.

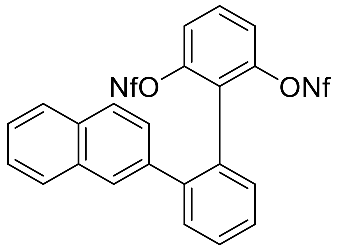

2t

$C_{30}H_{14}F_{18}O_6S_2$

M = 875.99 g/mol

2'-(naphthalen-2-yl)-[1,1'-biphenyl]-2,6-diyl bis(1,1,2,2,3,3,4,4,4-nonafluorobutane-1-sulfonate) (2t): Prepared from 2'-(naphthalen-2-yl)-[1,1'-biphenyl]-2,6-diol (936 mg, 3.00 mmol, 1.00 equiv) according to **GP 1**. **2t** was afforded as colorless oil (2.23 g, 85% yield). **^{1}H NMR** (500 MHz, $CDCl_3$, 298 K) δ = 7.65–7.64 (m, 1H), 7.59–7.55 (m, 2H), 7.51 (s, 1H), 7.47–7.45 (m, 2H), 7.40–7.34 (m, 2H), 7.32–7.28 (m, 2H), 7.21–7.18 (m, 2H), 7.11 (d, *J* = 8.5 Hz, 2H) ppm. **^{13}C{^{1}H} NMR** (126 MHz, $CDCl_3$, 298 K) δ = 147.8, 142.6, 137.8, 133.1, 132.4, 131.9, 130.7, 130.6, 130.1, 129.7, 128.2, 128.1, 127.6, 127.5, 127.3, 127.0, 126.9, 126.1, 121.5 (nonaflate group not listed) ppm. **^{19}F NMR** (471 MHz, $CDCl_3$, 298 K) δ = −80.62–(−81.28) (m), −109.13–(−110.47) (m), −120.93–(−120.95) (m), −125.90–(−125.97) (m) ppm. **HRMS** (APCI) m/z: $[M]^+$ calcd for $C_{30}H_{14}F_{18}O_6S_2^+$ 875.9939; found 875.9944.

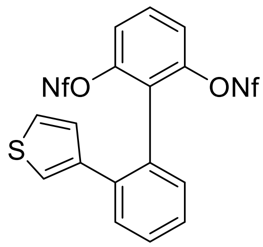

2u
$C_{24}H_{10}F_{18}O_6S_3$
M = 831.94 g/mol

2'-(thiophen-3-yl)-[1,1'-biphenyl]-2,6-diyl bis(1,1,2,2,3,3,4,4,4-nonafluorobutane-1-sulfonate) (2u): Prepared from 2'-(thiophen-3-yl)-[1,1'-biphenyl]-2,6-diol (804 mg, 3.00 mmol, 1.00 equiv) according to **GP 1**. **2u** was afforded as colorless oil (1.35 g, 54% yield). **^{1}H NMR** (500 MHz, $CDCl_3$, 298 K) δ = 7.57–7.48 (m, 3H), 7.45–7.43 (m, 1H), 7.39–7.34 (m, 3H), 7.17 (s, 1H), 6.95 (s, 1H), 6.95–6.88 (m, 1H) ppm. **^{13}C{^{1}H} NMR** (126 MHz, $CDCl_3$, 298 K) δ = 147.9, 140.6, 137.3, 131.9, 130.7, 130.1, 130.0, 129.8, 128.2, 127.2, 126.6, 125.0, 123.2, 121.7 (nonaflate group not listed) ppm. **^{19}F NMR** (471 MHz, $CDCl_3$, 298 K) δ = −80.71–(−81.17) (m), −109.70–(−109.81) (m), −120.94–(−121.38) (m), −125.85–(−125.90) (m) ppm. **HRMS** (APCI) m/z: $[M]^+$ calcd for $C_{24}H_{10}F_3NO_3S^+$ 831.9347; found 831.9349.

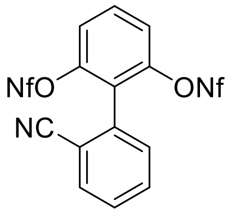

2v
$C_{21}H_7F_{18}NO_6S_2$
M = 774.94 g/mol

2'-cyano-[1,1'-biphenyl]-2,6-diyl bis(1,1,2,2,3,3,4,4,4-nonafluorobutane-1-sulfonate) (2v): Prepared from 2'-cyano-[1,1'-biphenyl]-2,6-diol (633 mg, 3.00 mmol, 1.00 equiv) according to **GP 1**. **2v** was afforded as white solid (1.26 g, 54% yield). **M.P**. 58–59 °C. **^{1}H NMR** (500 MHz, $CDCl_3$, 298 K) δ = 7.84 (d, J = 7.8 Hz, 1H), 7.75 (t, J = 7.7 Hz, 1H), 7.69 (t, J = 8.5 Hz, 1H), 7.63 (t, J = 7.7 Hz, 1H), 7.56 (d, J = 8.5 Hz, 2H), 7.52 (d, J = 7.8 Hz, 1H) ppm. **^{13}C{^{1}H} NMR** (126 MHz, $CDCl_3$, 298 K) δ = 147.5, 133.1, 132.6, 132.4, 132.2, 131.7, 130.2, 127.4, 122.2, 116.5, 114.5 (nonaflate group not listed) ppm. **^{19}F NMR** (471 MHz, $CDCl_3$, 298 K) δ = −80.70 (t, J = 9.8 Hz), −109.33 (t, J = 14.1 Hz), −120.87–(−120.91) (m), −125.81–(−125.88) (m) ppm. **HRMS** (APCI) m/z: $[M+H]^+$ calcd for $C_{21}H_8F_{18}NO_6S_2^+$ 775.9500; found 775.9498.

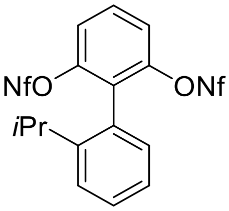

2w

$C_{23}H_{14}F_{18}O_6S_2$

M = 791.99 g/mol

2'-isopropyl-[1,1'-biphenyl]-2,6-diyl bis(1,1,2,2,3,3,4,4,4-nonafluorobutane-1-sulfonate) (2w): Prepared from 2'-isopropyl-[1,1'-biphenyl]-2,6-diol (684 mg, 3.00 mmol, 1.00 equiv) according to **GP 1**. **2w** was afforded as colorless oil (1.40 g, 59% yield). **^{1}H NMR** (500 MHz, $CDCl_3$, 298 K) δ = 7.58–7.55 (m, 1H), 7.51–7.46 (m, 4H), 7.31–7.29 (m, 1H), 7.20 (d, *J* = 7.8 Hz, 1H), 2.55–2.49 (m, 1H), 1.20 (s, 3H), 1.19 (s, 3H) ppm. **^{13}C{^{1}H} NMR** (126 MHz, $CDCl_3$, 298 K) δ = 148.2, 147.8, 131.1, 130.9, 130.3, 129.9, 126.5, 126.1, 125.6, 121.6, 31.0, 23.7 (nonaflate group not listed) ppm. **^{19}F NMR** (471 MHz, $CDCl_3$, 298 K) δ = −81.04–(−81.11) (m), −109.90–(−109.98) (m), −120.99–(−121.13) (m), −126.12–(−126.19) (m) ppm. **HRMS** (APCI) m/z: $[M+H]^+$ calcd for $C_{23}H_{15}F_{18}O_6S_2^+$ 793.0017; found 793.0017.

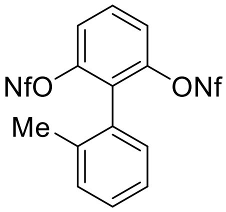

2x

$C_{21}H_{10}F_{18}O_6S_2$

M = 763.96 g/mol

2'-methyl-[1,1'-biphenyl]-2,6-diyl bis(1,1,2,2,3,3,4,4,4-nonafluorobutane-1-sulfonate) (2x): Prepared from 2'-methyl-[1,1'-biphenyl]-2,6-diol (600 mg, 3.00 mmol, 1.00 equiv) according to **GP 1**. **2x** was afforded as colorless oil (1.74 g, 76% yield). **^{1}H NMR** (500 MHz, $CDCl_3$, 298 K) δ = 7.58–7.55 (m, 1H), 7.50–7.48 (m, 2H), 7.41–7.38 (m, 1H), 7.35 (d, *J* = 7.6 Hz, 1H), 7.31 (t, *J* = 7.4 Hz, 1H), 7.24 (dd, *J* = 7.7, 1.6 Hz, 1H), 2.16 (s, 3H) ppm. **^{13}C{^{1}H} NMR** (126 MHz, $CDCl_3$, 298 K) δ = 148.0, 137.5, 131.1, 130.4, 129.9, 127.9, 125.8, 121.9, 19.3 (nonaflate group not listed) ppm. **^{19}F NMR** (471 MHz, $CDCl_3$, 298 K) δ = −81.08 (t, *J* = 9.8 Hz), −109.95 (t, *J* = 14.5 Hz), −121.14–(−121.18) (m), −126.10–(−126.19) (m) ppm. **HRMS** (APCI) m/z: $[M+H+N]^+$ calcd for $C_{21}H_{11}F_{18}NO_6S_2^+$ 778.9735; found 778.9729.

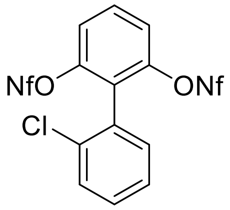

2y
$C_{20}H_7ClF_{18}O_6S_2$
M = 783.91 g/mol

2'-chloro-[1,1'-biphenyl]-2,6-diyl bis(1,1,2,2,3,3,4,4,4-nonafluorobutane-1-sulfonate) (2y): Prepared from 2'-chloro-[1,1'-biphenyl]-2,6-diol (660 mg, 3.00 mmol, 1.00 equiv) according to **GP 1**. **2y** was afforded as colorless oil (627 g, 80% yield). ^{1}**H NMR** (500 MHz, $CDCl_3$, 298 K) δ = 7.64–7.61 (m, 1H), 7.55–7.50 (m, 3H), 7.46–7.43 (m, 1H), 7.41–7.38 (m, 1H), 7.36–7.35 (m, 1H) ppm. ^{13}**C{**^{1}**H} NMR** (126 MHz, $CDCl_3$, 298 K) δ = 147.8, 134.5, 132.5, 131.2, 130.6, 129.8, 128.3, 127.8, 126.7, 121.7 (nonaflate group not listed) ppm. ^{19}**F NMR** (471 MHz, $CDCl_3$, 298 K) δ = −80.75 (t, *J* = 10.0 Hz), −109.64 (t, *J* = 14.1 Hz), −120.98 (d, *J* = 4.5 Hz), −125.88–(−125.95) (m) ppm. **HRMS** (APCI) m/z: $[M]^+$ calcd for $C_{20}H_7ClF_{18}O_6S_2^+$ 783.9080; found 783.9075.

4.2 Characterization Data of Chiral Products

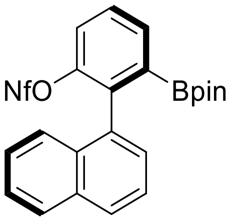

7aa
$C_{26}H_{22}BF_9O_5S$
M = 628.11 g/mol

(*R*)-2-(naphthalen-1-yl)-3-(4,4,5,5-tetramethyl-1,3,2-dioxaborolan-2-yl)phenyl 1,1,2,2,3,3,4,4,4-nonafluorobutane-1-sulfonate (**7aa**): Prepared from **2a** (80.0 mg, 0.100 mmol, 1.00 equiv) according to **GP 3**. The residue was purified by flash column chromatography on silica gel using ethyl acetate and hexane as the eluent to afford **7aa** as white solid (42.1 mg, 67% yield). **M.P.** 32–34 °C. **^{1}H NMR** (500 MHz, $CDCl_3$, 298 K) δ = 7.88 (dd, *J* = 13.5, 8.2 Hz, 2H), 7.82 (dd, *J* = 6.1, 2.6 Hz, 1H), 7.53–7.50 (m, 3H), 7.46–7.43 (m, 2H), 7.37 (d, *J* = 5.2 Hz, 2H), 0.89 (s, 6H), 0.73 (s, 6H) ppm. **^{13}C{^{1}H} NMR** (126 MHz, $CDCl_3$, 298 K) δ = 147.8, 138.8, 133.9, 133.4, 133.4, 132.9, 128.9, 128.4, 128.2, 128.0, 126.0, 125.6, 125.5, 124.7, 123.3, 83.7, 24.2, 24.0 (nonaflate group not observed) ppm. **^{19}F NMR** (471 MHz, $CDCl_3$, 298 K) δ = −80.80 (t, *J* = 9.8 Hz), −110.23 (t, *J* = 14.2 Hz), −121.15–(−121.18) (m), −125.99–(−126.06) (m) ppm. **^{11}B NMR** (160 MHz, $CDCl_3$, 298 K) δ = 30.16 ppm. **HRMS** (APCI) m/z: $[M]^+$ calcd for $C_{26}H_{21}BF_9O_5S^+$ 628.1132; found 628.1130. Optical rotation: $[\alpha]_D^{20}$ = 20.6 (*c* 1.0, CH_2Cl_2, e.r. = 97:3). The enantiomeric excess of **7aa** was determined by HPLC analysis on a chiral stationary phase (Daicel Chiralcel ID column, column temperature 20 °C, solvent heptane:*i*PrOH = 99.8:0.2, flow rate 0.5 mL/min): t_R = 9.8 min (minor), t_R = 10.4 min (major).

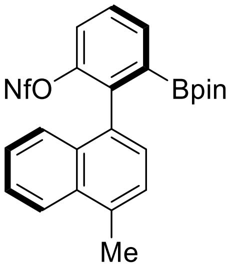

7ba
$C_{27}H_{24}BF_9O_5S$
M = 642.13 g/mol

(*R*)-2-(4-methylnaphthalen-1-yl)-3-(4,4,5,5-tetramethyl-1,3,2-dioxaborolan-2-yl)phenyl 1,1,2,2,3,3,4,4,4-nonafluorobutane-1-sulfonate (**7ba**): Prepared from **2b** (81.4 mg, 0.100 mmol, 1.00 equiv) according to **GP 3**. The residue was purified by flash column chromatography on silica gel using ethyl acetate and hexane as the eluent to afford **7ba** as colorless oil (39.8 mg, 62% yield). **^{1}H NMR** (400 MHz, $CDCl_3$, 298 K) δ = 8.03 (d, *J* = 8.4 Hz,

1H), 7.80–7.78 (m, 1H), 7.50–7.46 (m, 3H), 7.35 (dd, *J* = 16.3, 8.0 Hz, 4H), 2.75 (s, 3H), 0.88 (s, 6H), 0.72 (s, 6H) ppm. **^{13}C{^{1}H} NMR** (101 MHz, $CDCl_3$, 298 K) δ = 148.0, 139.0, 134.7, 133.8, 132.9, 132.5, 131.6, 128.8, 127.9, 126.2, 125.6, 125.4, 125.3, 124.1, 123.2, 83.7, 24.2, 24.0, 19.5 (nonaflate group not observed) ppm. **^{19}F NMR** (471 MHz, $CDCl_3$, 298 K) δ = −80.80 (t, *J* = 9.8 Hz), −110.19 (t, *J* = 14.1 Hz), −121.15–(−121.18) (m), −125.99–(−126.06) (m) ppm. **^{11}B NMR** (160 MHz, $CDCl_3$, 298 K) δ = 30.23 ppm. **HRMS** (APCI) m/z: $[M]^+$ calcd for $C_{27}H_{24}BF_9O_5S^+$ 642.1288; found 642.1285. Optical rotation: $[\alpha]_D^{20}$ = 15.0 (*c* 1.0, CH_2Cl_2, e.r. = 95:5). The enantiomeric excess of **7ba** was determined by HPLC analysis on a chiral stationary phase (Daicel Chiralcel ID column, column temperature 20 °C, solvent heptane:*i*PrOH = 99.9:0.1, flow rate 0.6 mL/min): t_R = 8.9 min (minor), t_R = 10.3 min (major).

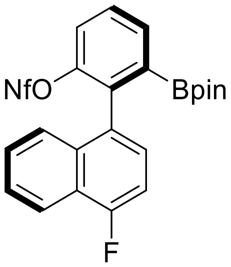

7ca
$C_{26}H_{21}BF_{10}O_5S$
M = 646.10 g/mol

(*R*)-2-(4-fluoronaphthalen-1-yl)-3-(4,4,5,5-tetramethyl-1,3,2-dioxaborolan-2-yl)phenyl 1,1,2,2,3,3,4,4,4-nonafluorobutane-1-sulfonate (**7ca**): Prepared from **2c** (81.8 mg, 0.100 mmol, 1.00 equiv) according to **GP 3**. The residue was purified by flash column chromatography on silica gel using ethyl acetate and hexane as the eluent to afford **7ca** as colorless oil (48.5 mg, 75% yield). **^{1}H NMR** (500 MHz, $CDCl_3$, 298 K) δ = 8.15 (d, *J* = 8.4 Hz, 1H), 7.84 (dd, *J* = 6.6, 2.7 Hz, 1H), 7.55–7.52 (dt, *J* = 10.2, 5.6 Hz, 3H), 7.42 (t, *J* = 7.7 Hz, 1H), 7.36 (d, *J* = 8.8 Hz, 2H), 7.26–7.18 (m, 1H), 0.91 (s, 6H), 0.76 (s, 6H) ppm. **^{13}C{^{1}H} NMR** (101 MHz, $CDCl_3$, 298 K) δ = 158.9 (d, *J* = 252.5 Hz), 147.9, 138.2, 134.3 (d, *J* = 5.0 Hz), 134.2, 129.5 (d, *J* = 4.5 Hz), 129.1, 128.0 (d, *J* = 8.5 Hz), 126.9, 125.9, 125.6 (d, *J* = 2.7 Hz), 123.5 (d, *J* = 16.4 Hz), 123.5, 120.5 (d, *J* = 5.4 Hz), 108.3 (d, *J* = 20.4 Hz), 83.8, 24.2, 24.1 (nonaflate group not observed) ppm. **^{19}F NMR** (471 MHz, $CDCl_3$, 298 K) δ = −80.64–(−80.83) (m), −110.18 (t, *J* = 14.2 Hz), −121.15, −123.28, −125.97–(−126.04) (m) ppm. **^{11}B NMR** (160 MHz, $CDCl_3$, 298 K) δ = 30.48 ppm. **HRMS** (APCI) m/z: $[M]^+$ calcd for $C_{26}H_{21}BF_{10}O_5S^+$ 646.1038; found 646.1039. Optical rotation: $[\alpha]_D^{20}$ = 15.8 (*c* 1.0, CH_2Cl_2, e.r. = 92:8). The enantiomeric excess of **7ca** was determined by HPLC analysis on a chiral stationary phase (Daicel Chiralcel ADH column, column temperature 20 °C, solvent heptane:*i*PrOH = 99.8:0.2, flow rate 0.5 mL/min): t_R = 10.0 min (minor), t_R = 10.6 min (major).

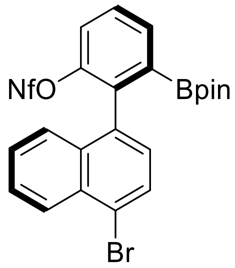

7da

$C_{26}H_{21}BBrF_9O_5S$

M = 706.02 g/mol

(*R*)-2-(4-bromonaphthalen-1-yl)-3-(4,4,5,5-tetramethyl-1,3,2-dioxaborolan-2-yl)phenyl 1,1,2,2,3,3,4,4,4-nonafluorobutane-1-sulfonate (**7da**): Prepared from **2d** (87.8 mg, 0.100 mmol, 1.00 equiv) according to **GP 3**. The residue was purified by flash column chromatography on silica gel using ethyl acetate and hexane as the eluent to afford **7da** as white solid (31.0 mg, 44% yield). **M.P**. 84–86 °C. ^{1}**H NMR** (500 MHz, $CDCl_3$, 298 K) δ = 8.30 (d, *J* = 8.5 Hz, 1H), 7.84 (dd, *J* = 6.8, 2.2 Hz, 2H), 7.55 (dd, *J* = 15.3, 6.9 Hz, 3H), 7.42 (t, *J* = 7.6 Hz, 1H), 7.36 (d, *J* = 8.5 Hz, 1H), 7.28 (d, *J* = 7.6 Hz, 1H), 0.90 (s, 6H), 0.74 (s, 6H) ppm. ^{13}**C{^{1}H} NMR** (126 MHz, $CDCl_3$, 298 K) δ = 147.6, 138.0, 134.3, 134.2, 133.7, 131.8, 129.3, 128.8, 128.4, 127.3, 127.0, 126.8, 126.2, 123.5, 123.2, 83.8, 24.2, 24.1 (nonaflate group not observed) ppm. ^{19}**F NMR** (471 MHz, $CDCl_3$, 298 K) δ = −80.68 (t, *J* = 9.8 Hz), −110.04–(−110.25) (m), −121.13–(−121.16) (m), −125.96–(−126.03) (m) ppm. ^{11}**B NMR** (160 MHz, $CDCl_3$, 298 K) δ = 30.16 ppm. **HRMS** (APCI) m/z: $[M]^+$ calcd for $C_{26}H_{24}BBrF_9O_5S^+$ 706.0237; found 706.0242. Optical rotation: $[\alpha]_D^{20}$ = 10.5 (*c* 1.0, CH_2Cl_2, e.r. = 90:10). The enantiomeric excess of **7da** was determined by HPLC analysis on a chiral stationary phase (Daicel Chiralcel ADH column, column temperature 20 °C, solvent heptane:*i*PrOH = 99.9:0.1, flow rate 0.4 mL/min): t_R = 11.8 min (minor), t_R = 12.5 min (major).

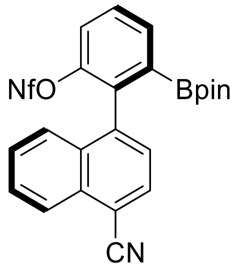

7ea

$C_{27}H_{21}BF_9NO_5S$

M = 653.11 g/mol

(*R*)-2-(4-cyanonaphthalen-1-yl)-3-(4,4,5,5-tetramethyl-1,3,2-dioxaborolan-2-yl)phenyl 1,1,2,2,3,3,4,4,4-nonafluorobutane-1-sulfonate (**7ea**): Prepared from **2e** (82.5 mg, 0.100 mmol, 1.00 equiv) according to **GP 3**. The residue was purified by flash column chromatography on silica gel using ethyl acetate and hexane as the eluent to afford **7ea** as

colorless oil (41.8 mg, 64% yield). **^{1}H NMR** (500 MHz, $CDCl_3$, 298 K) δ = 8.30 (d, *J* = 8.4 Hz, 1H), 7.96 (d, *J* = 7.3 Hz, 1H), 7.90 (d, *J* = 7.2 Hz, 1H), 7.67 (t, *J* = 7.6 Hz, 1H), 7.58 (dt, *J* = 14.8, 8.1 Hz, 2H), 7.49 (q, *J* = 7.7 Hz, 2H), 7.43 (d, *J* = 8.5 Hz, 1H), 0.90 (s, 6H), 0.72 (s, 6H) ppm. **^{13}C{^{1}H} NMR** (101 MHz, $CDCl_3$, 298 K) δ = 147.2, 139.5, 137.4, 134.7, 132.8, 132.2, 131.4, 129.8, 128.3, 127.6, 127.2, 126.5, 125.3, 123.8, 118.0, 110.2, 83.9, 24.2, 24.0 (nonaflate group not observed) ppm. **^{19}F NMR** (471 MHz, $CDCl_3$, 298 K) δ = −80.75 (t, *J* = 9.9 Hz), −110.02 (t, *J* = 14.2 Hz), −121.13–(−121.16) (m), −125.95–(−126.03) (m) ppm. **^{11}B NMR** (160 MHz, $CDCl_3$, 298 K) δ = 29.95 ppm. **HRMS** (APCI) m/z: $[M+H]^+$ calcd for $C_{27}H_{22}BF_9NO_5S^+$ 654.1163; found 654.1155. Optical rotation: $[\alpha]_D^{20}$ = 19.8 (*c* 1.0, CH_2Cl_2, e.r. = 99:1). The enantiomeric excess of **7ea** was determined by HPLC analysis on a chiral stationary phase (Daicel Chiralcel ADH column, column temperature 20 °C, solvent heptane:*i*PrOH = 99.8:0.2, flow rate 0.6 mL/min): t_R = 16.1 min (major), t_R = 18.0 min (minor).

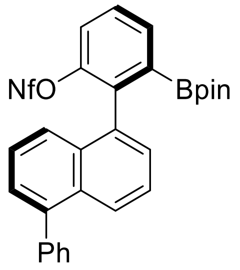

7fa
$C_{32}H_{26}BF_9O_5S$
M = 704.15 g/mol

(*R*)-2-(5-phenylnaphthalen-1-yl)-3-(4,4,5,5-tetramethyl-1,3,2-dioxaborolan-2-yl)phenyl 1,1,2,2,3,3,4,4,4-nonafluorobutane-1-sulfonate (**7fa**): Prepared from **2f** (87.6 mg, 0.100 mmol, 1.00 equiv) according to **GP 3**. The residue was purified by flash column chromatography on silica gel using ethyl acetate and hexane as the eluent to afford **7fa** as colorless oil (38.6 mg, 69% yield). **^{1}H NMR** (400 MHz, $CDCl_3$, 298 K) δ = 7.95–7.93 (m, 1H), 7.83 (t, *J* = 4.4 Hz, 1H), 7.54 (d, *J* = 4.3 Hz, 2H), 7.51 (d, *J* = 6.1 Hz, 4H), 7.47–7.45 (m, 3H), 7.40 (s, 3H), 0.91 (s, 6H), 0.74 (s, 6H) ppm. **^{13}C{^{1}H} NMR** (101 MHz, $CDCl_3$, 298 K) δ = 147.8, 141.1, 140.3, 139.0, 133.9, 133.6, 133.3, 131.7, 130.1, 129.0, 128.2, 128.1, 127.2, 126.6, 126.5, 125.3, 124.7, 123.4, 83.7, 24.3, 24.0 (nonaflate group not observed) ppm. **^{19}F NMR** (471 MHz, $CDCl_3$, 298 K) δ = −80.75 (t, *J* = 10.0 Hz), −110.09 (t, *J* = 14.3 Hz), −121.11, −125.95–(−126.02) (m) ppm. **^{11}B NMR** (160 MHz, $CDCl_3$, 298 K) δ = 30.08 ppm. **HRMS** (APCI) m/z: $[M]^+$ calcd for $C_{32}H_{26}BF_9O_5S^+$ 704.1445; found 704.1451. Optical rotation: $[\alpha]_D^{20}$ = 23.5 (*c* 1.0, CH_2Cl_2, e.r. = 94:6). The enantiomeric excess of **7fa** was determined by HPLC analysis on a chiral stationary phase (Daicel Chiralcel ID column, column temperature 20 °C, solvent heptane:*i*PrOH = 99.8:0.2, flow rate 0.5 mL/min): t_R = 8.5 min (minor), t_R = 9.3 min (major).

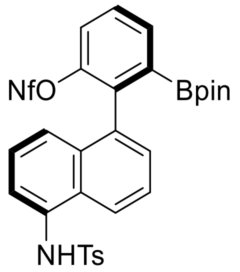

7ga
$C_{33}H_{29}BF_9NO_7S_2$
M = 797.13 g/mol

(*R*)-2-(5-((4-methylphenyl)sulfonamido)naphthalen-1-yl)-3-(4,4,5,5-tetramethyl-1,3,2-dioxaborolan-2-yl)phenyl 1,1,2,2,3,3,4,4,4-nonafluorobutane-1-sulfonate (**7ga**): Prepared from **2g** (96.9 mg, 0.100 mmol, 1.00 equiv) according to **GP 3**. The residue was purified by flash column chromatography on silica gel using ethyl acetate and hexane as the eluent to afford **7ga** as colorless oil (43.8 mg, 55% yield). **^{1}H NMR** (500 MHz, $CDCl_3$, 298 K) δ = 7.83 (dd, *J* = 18.8, 7.8 Hz, 2H), 7.63–7.61 (m, 2H), 7.54–7.40 (m, 5H), 7.26–7.25 (m, 1H), 7.15 (d, *J* = 8.0 Hz, 2H), 6.88 (s, 1H), 2.33 (s, 3H), 0.86 (s, 6H), 0.72 (s, 6H) ppm. **^{13}C{^{1}H} NMR** (126 MHz, $CDCl_3$, 298 K) δ = 147.8, 143.7, 138.6, 136.4, 134.2, 134.1, 133.7, 131.4, 129.5, 129.2, 128.9, 128.7, 127.3, 125.5, 125.4, 125.1, 123.4, 122.9, 121.7, 83.7, 24.2, 24.0, 21.4 (nonaflate group not observed) ppm. **^{19}F NMR** (471 MHz, $CDCl_3$, 298 K) δ = −80.72 (t, *J* = 10.0 Hz), −110.17 (t, *J* = 14.1 Hz), −121.11, −125.91–(−125.98) (m) ppm. **^{11}B NMR** (160 MHz, $CDCl_3$, 298 K) δ = 30.11 ppm. **HRMS** (APCI) m/z: $[M+H]^+$ calcd for $C_{33}H_{30}BF_9NO_7S_2^+$ 798.1408; found 798.1411. Optical rotation: $[\alpha]_D^{20}$ = 19.9 (*c* 1.0, CH_2Cl_2, e.r. = 94:6). The enantiomeric excess of **7ga** was determined by HPLC analysis on a chiral stationary phase (Daicel Chiralcel IC column, column temperature 20 °C, solvent heptane:*i*PrOH = 90:10, flow rate 0.5 mL/min): t_R = 13.9 min (major), t_R = 15.9 min (minor).

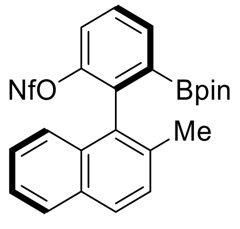

7ha
$C_{27}H_{24}BF_9O_5S$
M = 642.13 g/mol

(*R*)-2-(2-methylnaphthalen-1-yl)-3-(4,4,5,5-tetramethyl-1,3,2-dioxaborolan-2-yl)phenyl 1,1,2,2,3,3,4,4,4-nonafluorobutane-1-sulfonate (**7ha**): Prepared from **2h** (81.4 mg, 0.100 mmol, 1.00 equiv) according to **GP 3**. The residue was purified by flash column chromatography on silica gel using ethyl acetate and hexane as the eluent to afford **7ha** as colorless oil (46.2 mg, 72% yield). **^{1}H NMR** (500 MHz, $CDCl_3$, 298 K) δ = 7.85–7.83 (m, 1H),

7.79 (d, *J* = 8.3 Hz, 2H), 7.54–7.51 (m, 2H), 7.38–7.33 (m, 2H), 7.29 (ddd, *J* = 8.3, 6.7, 1.4 Hz, 1H), 7.15 (d, *J* = 8.6 Hz, 1H), 2.20 (s, 3H), 0.86 (s, 6H), 0.72 (s, 6H) ppm. **^{13}C{^{1}H} NMR** (126 MHz, $CDCl_3$, 298 K) δ = 147.9, 138.1, 134.8, 134.2, 133.3, 131.8, 131.3, 128.9, 128.0, 128.0, 127.5, 125.7, 125.4, 124.4, 123.3, 83.6, 24.2, 24.0, 20.4 (nonaflate group not observed) ppm. **^{19}F NMR** (471 MHz, $CDCl_3$, 298 K) δ = −80.73 (t, *J* = 9.7 Hz), −110.42–(−110.51) (m), −121.14–(−121.25) (m), −125.97–(−126.07) (m) ppm. **^{11}B NMR** (160 MHz, $CDCl_3$, 298 K) δ = 30.47 ppm. **HRMS** (APCI) m/z: $[M]^+$ calcd for $C_{27}H_{24}BF_9O_5S^+$ 642.1288; found 642.1283. e.r. = 52:48. The enantiomeric excess of **7ha** was determined by HPLC analysis on a chiral stationary phase (Daicel Chiralcel ODH column, column temperature 20 °C, solvent heptane:*i*PrOH = 99.9:0.1, flow rate 0.5 mL/min): t_R = 11.9 min (minor), t_R = 12.9 min (major).

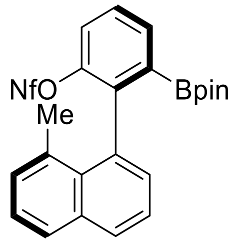

7ia
$C_{27}H_{24}BF_9O_5S$
M = 642.13 g/mol

(*R*)-2-(8-methylnaphthalen-1-yl)-3-(4,4,5,5-tetramethyl-1,3,2-dioxaborolan-2-yl)phenyl 1,1,2,2,3,3,4,4,4-nonafluorobutane-1-sulfonate (**7ia**): Prepared from **2i** (81.4 mg, 0.100 mmol, 1.00 equiv) according to **GP 3**. The residue was purified by flash column chromatography on silica gel using ethyl acetate and hexane as the eluent to afford **7ia** as colorless oil (43.0 mg, 67% yield). **^{1}H NMR** (500 MHz, $CDCl_3$, 298 K) δ = 7.79 (d, *J* = 8.2 Hz, 1H), 7.67–7.64 (m, 2H), 7.41–7.32 (m, 3H), 7.26–7.23 (m, 1H), 7.20–7.18 (m, 1H), 7.11 (d, *J* = 7.1 Hz, 1H), 1.90 (s, 3H), 0.77 (s, 6H), 0.75 (s, 6H) ppm. **^{13}C{^{1}H} NMR** (126 MHz, $CDCl_3$, 298 K) δ = 147.6, 142.9, 134.8, 134.7, 133.2, 133.0, 131.8, 130.3, 129.8, 129.4, 128.6, 127.5, 125.1, 123.7, 122.7, 83.7, 24.2, 24.1, 23.8 (nonaflate group not observed) ppm. **^{19}F NMR** (471 MHz, $CDCl_3$, 298 K) δ = −80.65–(−80.81) (m), −110.22–(−110.45) (m), −121.19, −125.99–(−126.03) (m) ppm. **^{11}B NMR** (160 MHz, $CDCl_3$, 298 K) δ = 30.53 ppm. **HRMS** (APCI) m/z: $[M]^+$ calcd for $C_{27}H_{24}BF_9O_5S^+$ 642.1288; found 642.1293. Optical rotation: $[\alpha]_D^{20}$ = 1.7 (*c* 1.0, CH_2Cl_2, e.r. = 99:1). The enantiomeric excess of **7ia** was determined by HPLC analysis on a chiral stationary phase (Daicel Chiralcel IC column, column temperature 20 °C, solvent heptane:*i*PrOH = 99.8:0.2, flow rate 0.6 mL/min): t_R = 13.5 min (major), t_R = 27.5 min (minor).

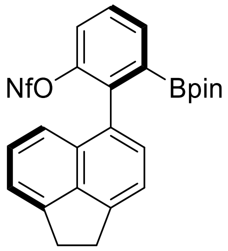

7ja
$C_{28}H_{24}BF_9O_5S$
M = 654.13 g/mol

(*R*)-2-(1,2-dihydroacenaphthylen-5-yl)-3-(4,4,5,5-tetramethyl-1,3,2-dioxaborolan-2-yl)phenyl 1,1,2,2,3,3,4,4,4-nonafluorobutane-1-sulfonate (**7ja**): Prepared from **2j** (82.6 mg, 0.100 mmol, 1.00 equiv) according to **GP 3**. The residue was purified by flash column chromatography on silica gel using ethyl acetate and hexane as the eluent to afford **7ja** as colorless oil (45.9 mg, 70% yield). **^{1}H NMR** (500 MHz, $CDCl_3$, 298 K) δ = 7.68 (t, *J* = 4.5 Hz, 1H), 7.39 (d, *J* = 4.4 Hz, 2H), 7.30 (d, *J* = 7.0 Hz, 1H), 7.23 (t, *J* = 6.6 Hz, 2H), 7.15 (d, *J* = 6.9 Hz, 1H), 7.04 (d, *J* = 8.3 Hz, 1H), 3.33 (s, 4H), 0.82 (s, 6H), 0.63 (s, 6H) ppm. **^{13}C{^{1}H} NMR** (126 MHz, $CDCl_3$, 298 K) δ = 148.1, 146.3, 145.7, 139.1, 138.5, 133.8, 131.3, 129.9, 128.8, 128.6, 127.9, 123.2, 120.7, 119.0, 118.3, 83.7, 30.5, 30.1, 24.3, 24.1 (nonaflate group not observed) ppm. **^{19}F NMR** (471 MHz, $CDCl_3$, 298 K) δ = −80.79 (t, *J* = 9.8 Hz), −110.16 (t, *J* = 14.3 Hz), −121.12–(−121.14) (m), −125.98–(−126.05) (m) ppm. **^{11}B NMR** (160 MHz, $CDCl_3$, 298 K) δ = 30.43 ppm. **HRMS** (APCI) m/z: $[M]^+$ calcd for $C_{28}H_{24}BF_9O_5S^+$ 654.1288; found 654.1284. Optical rotation: $[\alpha]_D^{20}$ = 9.7 (*c* 1.0, CH_2Cl_2, e.r. = 97:3). The enantiomeric excess of **7ja** was determined by HPLC analysis on a chiral stationary phase (Daicel Chiralcel ID column, column temperature 20 °C, solvent heptane:*i*PrOH = 99.8:0.2, flow rate 0.5 mL/min): t_R = 10.4 min (major), t_R = 11.7 min (minor).

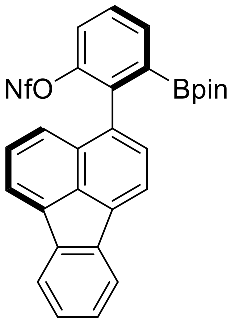

7ka
$C_{32}H_{24}BF_9O_5S$
M = 702.13 g/mol

(*R*)-2-(fluoranthen-3-yl)-3-(4,4,5,5-tetramethyl-1,3,2-dioxaborolan-2-yl)phenyl 1,1,2,2,3,3,4,4,4-nonafluorobutane-1-sulfonate (**7ka**): Prepared from **2k** (87.3 mg, 0.100 mmol, 1.00 equiv) according to **GP 3**. The residue was purified by flash column

chromatography on silica gel using ethyl acetate and hexane as the eluent to afford **7ka** as colorless oil (42.1 mg, 60% yield). **^{1}H NMR** (500 MHz, $CDCl_3$, 298 K) δ = 7.99–7.92 (m, 4H), 7.85–7.83 (m, 1H), 7.61 (dd, *J* = 7.2, 2.4 Hz, 1H), 7.54 (d, *J* = 6.8 Hz, 3H), 7.41–7.40 (m, 3H), 0.90 (s, 6H), 0.69 (s, 6H) ppm. **^{13}C{^{1}H} NMR** (126 MHz, $CDCl_3$, 298 K) δ = 147.9, 139.8, 139.4, 137.8, 137.2, 136.9, 133.9, 133.6, 132.4, 130.1, 130.0, 129.0, 128.0, 127.5, 127.5, 125.2, 123.3, 121.6, 121.5, 119.9, 119.2, 83.8, 24.3, 24.0 (nonaflate group not observed) ppm. **^{19}F NMR** (471 MHz, $CDCl_3$, 298 K) δ = −80.79 (t, *J* = 10.0 Hz), −110.06 (t, *J* = 14.1 Hz), −121.08, −125.98 (t, *J* = 17.3 Hz) ppm. **^{11}B NMR** (160 MHz, $CDCl_3$, 298 K) δ = 30.54 ppm. **HRMS** (APCI) m/z: $[M-C_6H_{10}BO]^+$ calcd for $C_{26}H_{14}F_9O_4S^+$ 593.0464; found 593.0457. Optical rotation: $[\alpha]_D^{20}$ = 19.2 (*c* 1.0, CH_2Cl_2, e.r. = 96:4). The enantiomeric excess of **7ka** was determined by HPLC analysis on a chiral stationary phase (Daicel Chiralcel ID column, column temperature 20 °C, solvent heptane:*i*PrOH = 99.8:0.2, flow rate 0.5 mL/min): t_R = 23.4 min (minor), t_R = 31.6 min (major).

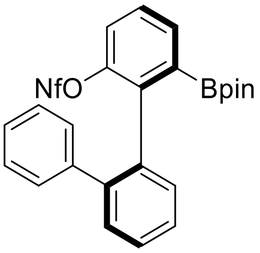

7la
$C_{28}H_{24}BF_9O_5S$
M = 654.13 g/mol

(*R*)-6-(4,4,5,5-tetramethyl-1,3,2-dioxaborolan-2-yl)-[1,1':2',1''-terphenyl]-2-yl 1,1,2,2,3,3,4,4,4-nonafluorobutane-1-sulfonate (**7la**): Prepared from **2l** (82.6 mg, 0.100 mmol, 1.00 equiv) according to **GP 3**. The residue was purified by flash column chromatography on silica gel using ethyl acetate and hexane as the eluent to afford **7la** as colorless oil (46.4 mg, 71% yield). **^{1}H NMR** (400 MHz, $CDCl_3$, 298 K) δ = 7.68 (d, *J* = 7.4 Hz, 1H), 7.47–7.43 (m, 1H), 7.39–7.26 (m, 4H), 7.18–7.15 (m, 5H), 7.08 (d, *J* = 8.2 Hz, 1H), 1.18 (s, 6H), 1.09 (s, 6H) ppm. **^{13}C{^{1}H} NMR** (101 MHz, $CDCl_3$, 298 K) δ = 146.9, 141.8, 141.3, 140.3, 134.6, 133.6, 131.7, 129.4, 128.4, 127.5, 126.5, 126.4, 122.6, 84.0, 24.8, 24.3 (nonaflate group not observed) ppm. **^{19}F NMR** (471 MHz, $CDCl_3$, 298 K) δ = −80.66–(−80.75) (m), −109.67–(−111.26) (m), −121.06–(−121.09) (m), −125.87–(−125.94) (m) ppm. **^{11}B NMR** (160 MHz, $CDCl_3$, 298 K) δ = 30.70 ppm. **HRMS** (APCI) m/z: $[M]^+$ calcd for $C_{28}H_{24}BF_9O_5S^+$ 654.1288; found 654.1293. Optical rotation: $[\alpha]_D^{20}$ = 1.2 (*c* 1.0, CH_2Cl_2, e.r. = 98:2). The enantiomeric excess of **7la** was determined by HPLC analysis on a chiral stationary phase (Daicel Chiralcel IC column, column temperature 20 °C, solvent heptane:*i*PrOH = 99.8:0.2, flow rate 0.5 mL/min): t_R = 7.9 min (major), t_R = 9.7 min (minor).

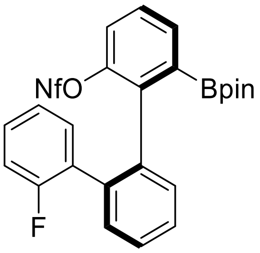

7ma
$C_{28}H_{23}BF_{10}O_5S$
M = 672.12 g/mol

(*R*)-2''-fluoro-6-(4,4,5,5-tetramethyl-1,3,2-dioxaborolan-2-yl)-[1,1':2',1''-terphenyl]-2-yl 1,1,2,2,3,3,4,4,4-nonafluorobutane-1-sulfonate (**7ma**): Prepared from **2m** (84.4 mg, 0.100 mmol, 1.00 equiv) according to **GP 3**. The residue was purified by flash column chromatography on silica gel using ethyl acetate and hexane as the eluent to afford **7ma** as colorless oil (45.7 mg, 68% yield). ^{1}**H NMR** (500 MHz, $CDCl_3$, 298 K) δ = 7.69 (d, *J* = 7.4 Hz, 1H), 7.47–7.44 (m, 1H), 7.40–7.34 (m, 3H), 7.29–7.26 (m, 1H), 7.13–7.09 (m, 3H), 6.93–7.87 (m, 2H), 1.16 (s, 6H), 1.13 (s, 6H) ppm. ^{13}**C{^{1}H} NMR** (126 MHz, $CDCl_3$, 298 K) δ = 159.5 (d, *J* = 247.6 Hz), 147.0, 139.8, 135.6, 135.3, 133.9, 132.2 (d, *J* = 3.4 Hz), 132.0, 130.0, 128.8 (d, *J* = 8.1 Hz), 128.5, 128.0, 126.8, 123.0 (d, *J* = 3.5 Hz), 122.5, 115.2 (d, *J* = 22.4 Hz), 84.0, 24.7, 24.4 (nonaflate group not observed) ppm. ^{19}**F NMR** (471 MHz, $CDCl_3$, 298 K) δ = −80.73 (t, *J* = 9.8 Hz), −110.58–(−111.17) (m), −114.25, −121.10, −125.90 (t, *J* = 13.5 Hz) ppm. ^{11}**B NMR** (160 MHz, $CDCl_3$, 298 K) δ = 30.63 ppm. **HRMS** (APCI) m/z: $[M]^+$ calcd for $C_{28}H_{23}BF_{10}O_5S^+$ 672.1194; found 672.1193. Optical rotation: $[\alpha]_D^{20}$ = −7.4 (*c* 1.0, CH_2Cl_2, e.r. = 90:10). The enantiomeric excess of **7ma** was determined by HPLC analysis on a chiral stationary phase (Daicel Chiralcel IC column, column temperature 20 °C, solvent heptane:*i*PrOH = 99.8:0.2, flow rate 0.5 mL/min): t_R = 8.3 min (major), t_R = 9.4 min (minor).

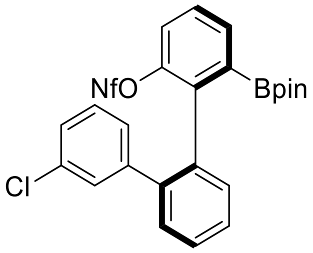

7na
$C_{28}H_{23}BClF_9O_5S$
M = 688.09 g/mol

(*R*)-3''-chloro-6-(4,4,5,5-tetramethyl-1,3,2-dioxaborolan-2-yl)-[1,1':2',1''-terphenyl]-2-yl 1,1,2,2,3,3,4,4,4-nonafluorobutane-1-sulfonate (**7na**): Prepared from **2n** (85.9 mg, 0.100 mmol, 1.00 equiv) according to **GP 3**. The residue was purified by flash column chromatography on silica gel using ethyl acetate and hexane as the eluent to afford **7na** as colorless oil (55.1 mg, 80% yield). ^{1}**H NMR** (500 MHz, $CDCl_3$, 298 K) δ = 7.63 (dd, *J* = 7.4, 1.2

Hz, 1H), 7.38 (td, *J* = 7.6, 1.4 Hz, 1H), 7.31–7.23 (m, 3H), 7.19–7.18 (m, 2H), 7.07–7.04 (m, 2H), 7.00–6.97 (m, 2H), 1.11 (s, 6H), 1.02 (s, 6H) ppm. **^{13}C{^{1}H} NMR** (101 MHz, $CDCl_3$, 298 K) δ = 146.8, 143.1, 140.4, 139.9, 134.7, 133.9, 133.3, 131.7, 129.7, 129.2, 128.7, 128.7, 128.5, 127.4, 126.9, 126.6, 122.7, 84.0, 24.8, 24.3 (nonaflate group not observed) ppm. **^{19}F NMR** (471 MHz, $CDCl_3$, 298 K) δ = −80.53–(−80.73) (m), −109.62–(−111.29) (m), −121.05–(−121.08) (m), −125.83–(−125.93) (m) ppm. **^{11}B NMR** (160 MHz, $CDCl_3$, 298 K) δ = 31.20 ppm. **HRMS** (APCI) m/z: $[M]^+$ calcd for $C_{28}H_{23}BClF_9O_5S^+$ 688.0899; found 688.0894. Optical rotation: $[\alpha]_D^{20}$ = 1.9 (*c* 1.0, CH_2Cl_2, e.r. = 99:1). The enantiomeric excess of **7na** was determined by HPLC analysis on a chiral stationary phase (Daicel Chiralcel IC column, column temperature 20 °C, solvent heptane:*i*PrOH = 99.9:0.1, flow rate 0.4 mL/min): t_R = 11.6 min (major), t_R = 13.1 min (minor).

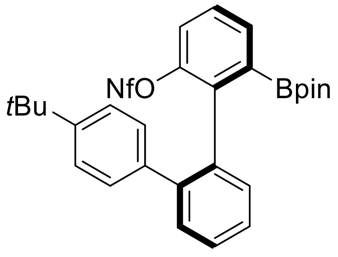

7oa
$C_{32}H_{32}BF_9O_5S$
M = 710.19 g/mol

(*R*)-4''-(*tert*-butyl)-6-(4,4,5,5-tetramethyl-1,3,2-dioxaborolan-2-yl)-[1,1':2',1''-terphenyl]-2-yl 1,1,2,2,3,3,4,4,4-nonafluorobutane-1-sulfonate (**7oa**): Prepared from **2o** (88.2 mg, 0.100 mmol, 1.00 equiv) according to **GP 3**. The residue was purified by flash column chromatography on silica gel using ethyl acetate and hexane as the eluent to afford **7oa** as colorless oil (33.4 mg, 47% yield). **^{1}H NMR** (500 MHz, $CDCl_3$, 298 K) δ = 7.68 (d, *J* = 7.4 Hz, 1H), 7.44–7.38 (m, 2H), 7.33–7.29 (m, 2H), 7.26–7.23 (m, 1H), 7.16 (d, *J* = 8.2 Hz, 2H), 7.10 (dd, *J* = 8.1, 5.4 Hz, 3H), 1.25 (s, 9H), 1.17 (s, 6H), 1.08 (s, 6H) ppm. **^{13}C{^{1}H} NMR** (126 MHz, $CDCl_3$, 298 K) δ = 149.2, 146.9, 141.7, 140.5, 138.2, 134.6, 133.6, 131.7, 129.5, 129.0, 128.3, 128.3, 126.1, 124.4, 122.5, 84.0, 34.3, 31.3, 24.8, 24.3 (nonaflate group not observed) ppm. **^{19}F NMR** (471 MHz, $CDCl_3$, 298 K) δ = −80.55–(−80.75) (m), −109.71–(−111.39) (m), −121.08–(−121.10) (m), −125.89–(−125.95) (m) ppm. **^{11}B NMR** (160 MHz, $CDCl_3$, 298 K) δ = 30.56 ppm. **HRMS** (APCI) m/z: $[M]^+$ calcd for $C_{32}H_{32}BF_9O_5S^+$ 710.1914; found 710.1909. Optical rotation: $[\alpha]_D^{20}$ = 5.0 (*c* 1.0, CH_2Cl_2, e.r. = 95:5). The enantiomeric excess of **7oa** was determined by HPLC analysis on a chiral stationary phase (Daicel Chiralcel IC column, column temperature 20 °C, solvent heptane:*i*PrOH = 99.8:0.2, flow rate 0.5 mL/min): t_R = 8.8 min (major), t_R = 10.6 min (minor).

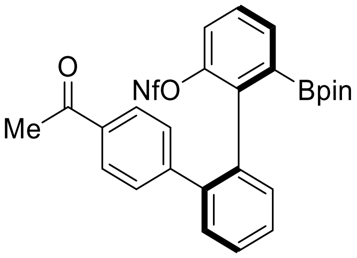

7pa
$C_{30}H_{26}BF_9O_6S$
M = 696.14 g/mol

(*R*)-4''-acetyl-6-(4,4,5,5-tetramethyl-1,3,2-dioxaborolan-2-yl)-[1,1':2',1''-terphenyl]-2-yl 1,1,2,2,3,3,4,4,4-nonafluorobutane-1-sulfonate (**7pa**): Prepared from **2p** (86.8 mg, 0.100 mmol, 1.00 equiv) according to **GP 3**. The residue was purified by flash column chromatography on silica gel using ethyl acetate and hexane as the eluent to afford **7pa** as colorless oil (50.1 mg, 72% yield). **^{1}H NMR** (500 MHz, $CDCl_3$, 298 K) δ = 7.70–7.68 (m, 2H), 7.63–7.61 (m, 1H), 7.42–7.39 (m, 1H), 7.32–7.30 (m, 2H), 7.25–719 (m, 4H), 7.02 (dd, *J* = 7.6, 2.6 Hz, 1H), 2.47 (d, *J* = 2.9 Hz, 3H), 1.11 (s, 6H), 1.02 (s, 6H) ppm. **^{13}C{^{1}H} NMR** (126 MHz, $CDCl_3$, 298 K) δ = 198.0, 146.9, 146.4, 140.7, 139.8, 135.3, 134.6, 133.9, 131.9, 129.6, 129.2, 128.7, 128.5, 127.7, 127.1, 122.8, 84.1, 26.5, 24.8, 24.3 (nonaflate group not observed) ppm. **^{19}F NMR** (471 MHz, $CDCl_3$, 298 K) δ = −80.53–(−81.17) (m), −109.56–(−111.16) (m), −121.07–(−121.48) (m), −125.86–(−125.93) (m) ppm. **^{11}B NMR** (160 MHz, $CDCl_3$, 298 K) δ = 30.69 ppm. **HRMS** (APCI) m/z: $[M+H]^+$ calcd for $C_{30}H_{27}BF_9O_6S^+$ 697.1472; found 697.1470. Optical rotation: $[\alpha]_D^{20}$ = 16.4 (*c* 1.0, CH_2Cl_2, e.r. = 99:1). The enantiomeric excess of **7pa** was determined by HPLC analysis on a chiral stationary phase (Daicel Chiralcel IC column, column temperature 20 °C, solvent heptane:*i*PrOH = 99.5:0.5, flow rate 0.5 mL/min): t_R = 24.7 min (major), t_R = 31.2 min (minor).

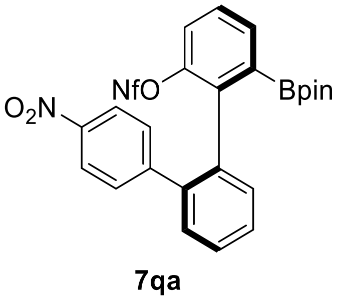

7qa
$C_{28}H_{23}BF_9NO_7S$
M = 699.11 g/mol

(*R*)-4''-nitro-6-(4,4,5,5-tetramethyl-1,3,2-dioxaborolan-2-yl)-[1,1':2',1''-terphenyl]-2-yl 1,1,2,2,3,3,4,4,4-nonafluorobutane-1-sulfonate (**7qa**): Prepared from **2q** (87.1 mg, 0.100 mmol, 1.00 equiv) according to **GP 3**. The residue was purified by flash column chromatography on silica gel using ethyl acetate and hexane as the eluent to afford **7qa** as

colorless oil (41.2 mg, 59% yield). **^{1}H NMR** (500 MHz, $CDCl_3$, 298 K) δ = 7.97–7.96 (m, 2H), 7.65–7.63 (m, 1H), 7.44–7.42 (m, 1H), 7.36 (s, 1H), 7.32–7.26 (m, 3H), 7.23 (d, *J* = 7.1 Hz, 1H), 7.19–7.18 (m, 1H), 7.04–7.03 (m, 1H), 1.12 (s, 6H), 1.02 (s, 6H) ppm. **^{13}C{^{1}H} NMR** (101 MHz, $CDCl_3$, 298 K) δ = 148.2, 146.7, 146.6, 139.6, 139.3, 138.2, 134.7, 134.0, 132.1, 130.2, 129.1, 128.7, 127.8, 122.9, 84.2, 24.9, 24.2 (nonaflate group not observed) ppm. **^{19}F NMR** (471 MHz, $CDCl_3$, 298 K) δ = −80.69 (t, *J* = 9.9 Hz), −109.45–(−111.08) (m), −121.05, −125.88 (t, *J* = 15.1 Hz) ppm. **^{11}B NMR** (160 MHz, $CDCl_3$, 298 K) δ = 30.78 ppm. **HRMS** (APCI) m/z: $[M]^+$ calcd for $C_{28}H_{23}BF_9NO_7S^+$ 699.1145; found 699.1132. Optical rotation: $[\alpha]_D^{20}$ = 18.6 (*c* 1.0, CH_2Cl_2, e.r. = 90:10). The enantiomeric excess of **7qa** was determined by HPLC analysis on a chiral stationary phase (Daicel Chiralcel IA column, column temperature 20 °C, solvent heptane:*i*PrOH = 99.8:0.2, flow rate 0.5 mL/min): t_R = 12.4 min (minor), t_R = 17.0 min (major).

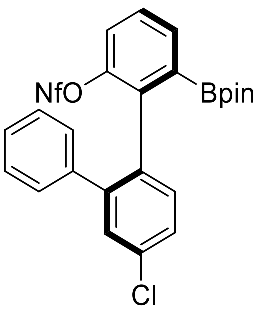

7ra
$C_{28}H_{23}BClF_9O_5S$
M = 688.09 g/mol

(*R*)-4'-chloro-6-(4,4,5,5-tetramethyl-1,3,2-dioxaborolan-2-yl)-[1,1':2',1''-terphenyl]-2-yl 1,1,2,2,3,3,4,4,4-nonafluorobutane-1-sulfonate (**7ra**): Prepared from **2r** (86.0 mg, 0.100 mmol, 1.00 equiv) according to **GP 3**. The residue was purified by flash column chromatography on silica gel using ethyl acetate and hexane as the eluent to afford **7ra** as colorless oil (53.7 mg, 78% yield). **^{1}H NMR** (500 MHz, $CDCl_3$, 298 K) δ = 7.71 (dd, *J* = 7.3, 1.2 Hz, 1H), 7.40 (d, *J* = 2.2 Hz, 1H), 7.34 (dd, *J* = 8.2, 2.2 Hz, 1H), 7.31 (t, *J* = 7.8 Hz, 1H), 7.21 (d, *J* = 8.2 Hz, 1H), 7.16 (s, 5H), 7.11 (d, *J* = 8.3 Hz, 1H), 1.20 (s, 6H), 1.13 (s, 6H) ppm. **^{13}C{^{1}H} NMR** (101 MHz, $CDCl_3$, 298 K) δ = 146.8, 143.5, 139.9, 139.2, 134.2, 134.1, 133.2, 133.0, 129.3, 129.2, 128.8, 127.6, 127.0, 126.3, 122.8, 84.1, 24.8, 24.3 (nonaflate group not observed) ppm. **^{19}F NMR** (471 MHz, $CDCl_3$, 298 K) δ = −80.53–(−80.73) (m), −109.51–(−111.07) (m), −121.04–(−121.07) (m), −125.88 (t, *J* = 11.4 Hz) ppm. **^{11}B NMR** (160 MHz, $CDCl_3$, 298 K) δ = 31.04 ppm. **HRMS** (APCI) m/z: $[M]^+$ calcd for $C_{28}H_{23}BClF_9O_5S^+$ 688.0899; found 688.0901. Optical rotation: $[\alpha]_D^{20}$ = 3.4 (*c* 1.0, CH_2Cl_2, e.r. = 91:9). The enantiomeric excess of **7ra** was determined by HPLC analysis on a chiral stationary phase (Daicel Chiralcel IC column, column temperature 20 °C, solvent heptane:*i*PrOH = 99.8:0.2, flow rate 0.6 mL/min): t_R = 9.4 min (major), t_R = 11.4 min (minor).

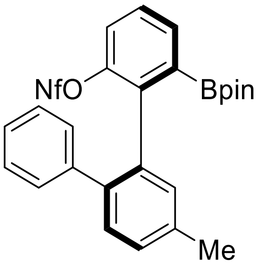

7sa
$C_{29}H_{26}BF_9O_5S$
M = 668.15 g/mol

(*R*)-5'-methyl-6-(4,4,5,5-tetramethyl-1,3,2-dioxaborolan-2-yl)-[1,1':2',1''-terphenyl]-2-yl 1,1,2,2,3,3,4,4,4-nonafluorobutane-1-sulfonate (**7sa**): Prepared from **2s** (84.0 mg, 0.100 mmol, 1.00 equiv) according to **GP 3**. The residue was purified by flash column chromatography on silica gel using ethyl acetate and hexane as the eluent to afford **7sa** as colorless oil (38.4 mg, 58% yield). **^{1}H NMR** (500 MHz, $CDCl_3$, 298 K) δ = 7.68 (dd, *J* = 7.4, 1.2 Hz, 1H), 7.32–7.28 (m, 3H), 7.20–7.15 (m, 5H), 7.10 (t, *J* = 4.2 Hz, 2H), 2.40 (s, 3H), 1.21 (s, 6H), 1.12 (s, 6H) ppm. **^{13}C{^{1}H} NMR** (126 MHz, $CDCl_3$, 298 K) δ = 146.9, 141.3, 140.3, 139.0, 135.8, 134.3, 133.4, 132.5, 129.4, 129.3, 129.0, 128.3, 127.4, 126.3, 122.5, 84.0, 24.8, 24.2, 20.8 (nonaflate group not observed) ppm. **^{19}F NMR** (471 MHz, $CDCl_3$, 298 K) δ = −80.57–(−81.21) (m), −109.72–(−111.34) (m), −121.09–(−121.54) (m), −125.89–(−126.00) (m) ppm. **^{11}B NMR** (160 MHz, $CDCl_3$, 298 K) δ = 30.80 ppm. **HRMS** (APCI) m/z: $[M]^+$ calcd for $C_{29}H_{26}BF_9O_5S^+$ 668.1445; found 668.1445. Optical rotation: $[\alpha]_D^{20}$ = 5.4 (*c* 1.0, CH_2Cl_2, e.r. = 54:46). The enantiomeric excess of **7sa** was determined by HPLC analysis on a chiral stationary phase (Daicel Chiralcel IC column, column temperature 20 °C, solvent heptane:*i*PrOH = 99.8:0.2, flow rate 0.6 mL/min): t_R = 11.5 min (minor), t_R = 16.3 min (major).

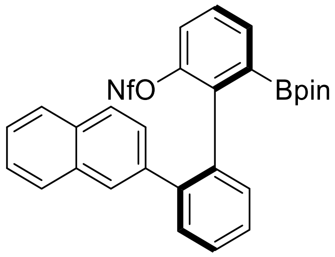

7ta
$C_{32}H_{26}BF_9O_5S$
M = 704.15 g/mol

(*R*)-2'-(naphthalen-2-yl)-6-(4,4,5,5-tetramethyl-1,3,2-dioxaborolan-2-yl)-[1,1'-biphenyl]-2-yl 1,1,2,2,3,3,4,4,4-nonafluorobutane-1-sulfonate (**7ta**): Prepared from **2t** (87.6 mg, 0.100 mmol, 1.00 equiv) according to **GP 3**. The residue was purified by flash column chromatography on silica gel using ethyl acetate and hexane as the eluent to afford **7ta** as colorless oil (55.6 mg, 79% yield). **^{1}H NMR** (500 MHz, $CDCl_3$, 298 K) δ = 7.75–7.64 (m, 4H),

7.61 (d, *J* = 8.5 Hz, 1H), 7.48 (d, *J* = 6.7 Hz, 2H), 7.41–7.38 (m, 3H), 7.35–7.30 (m, 2H), 7.24 (t, *J* = 7.8 Hz, 1H), 7.03 (d, *J* = 8.2 Hz, 1H), 1.21 (s, 6H), 1.12 (s, 6H) ppm. **^{13}C{^{1}H} NMR** (101 MHz, $CDCl_3$, 298 K) δ = 146.9, 141.7, 140.3, 138.9, 134.8, 133.8, 133.0, 132.1, 131.7, 129.7, 128.4, 128.0, 127.7, 127.4, 126.9, 126.5, 125.7, 125.6, 122.7, 84.0, 24.8, 24.3 (nonaflate group not observed) ppm. **^{19}F NMR** (471 MHz, $CDCl_3$, 298 K) δ = −80.54–(−81.17) (m), −109.67–(−111.29) (m), −121.03–(−121.06) (m), −125.86–(−125.93) (m) ppm. **^{11}B NMR** (160 MHz, $CDCl_3$, 298 K) δ = 30.33 ppm. **HRMS** (APCI) m/z: $[M]^+$ calcd for $C_{32}H_{26}BF_9O_5S^+$ 704.1445; found 704.1438. Optical rotation: $[\alpha]_D^{20}$ = 9.9 (*c* 1.0, CH_2Cl_2, e.r. = 98:2). The enantiomeric excess of **7ta** was determined by HPLC analysis on a chiral stationary phase (Daicel Chiralcel IC column, column temperature 20 °C, solvent heptane:*i*PrOH = 99.8:0.2, flow rate 0.5 mL/min): t_R = 9.3 min (major), t_R = 11.5 min (minor).

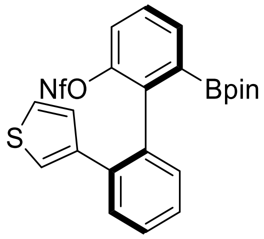

7ua
$C_{26}H_{22}BF_9O_5S_2$
M = 660.09 g/mol

(*R*)-6-(4,4,5,5-tetramethyl-1,3,2-dioxaborolan-2-yl)-2'-(thiophen-3-yl)-[1,1'-biphenyl]-2-yl 1,1,2,2,3,3,4,4,4-nonafluorobutane-1-sulfonate (**7ua**): Prepared from **2u** (87.6 mg, 0.100 mmol, 1.00 equiv) according to **GP 3**. The residue was purified by flash column chromatography on silica gel using ethyl acetate and hexane as the eluent to afford **7ua** as colorless oil (36.3 mg, 55% yield). **^{1}H NMR** (500 MHz, $CDCl_3$, 298 K) δ = 7.70–7.68 (m, 1H), 7.43 (d, *J* = 14.4 Hz, 2H), 7.37–7.31 (m, 2H), 7.26–7.24 (m, 1H), 7.20 (d, *J* = 7.8 Hz, 1H), 7.10–7.09 (m, 1H), 6.92 (s, 1H), 6.87 (d, *J* = 4.4 Hz, 1H), 1.14 (s, 6H), 1.06 (s, 6H) ppm. **^{13}C{^{1}H} NMR** (126 MHz, $CDCl_3$, 298 K) δ = 147.1, 141.5, 140.3, 136.6, 134.5, 133.7, 131.6, 128.9, 128.7, 128.6, 128.4, 126.4, 124.2, 123.1, 122.7, 84.0, 24.7, 24.3 (nonaflate group not observed) ppm. **^{19}F NMR** (471 MHz, $CDCl_3$, 298 K) δ = −80.69–(−81.15) (m), −109.70–(−111.13) (m), −121.09, −125.90 (t, *J* = 14.4 Hz) ppm. **^{11}B NMR** (160 MHz, $CDCl_3$, 298 K) δ = 30.82 ppm. **HRMS** (APCI) m/z: $[M+H]^+$ calcd for $C_{26}H_{23}BF_9O_5S_2^+$ 661.0931; found 661.0935. Optical rotation: $[\alpha]_D^{20}$ = 3.3 (*c* 1.0, CH_2Cl_2, e.r. = 90:10). The enantiomeric excess of **7ua** was determined by HPLC analysis on a chiral stationary phase (Daicel Chiralcel IC column, column temperature 20 °C, solvent heptane:*i*PrOH = 99.5:0.5, flow rate 0.4 mL/min): t_R = 9.1 min (major), t_R = 9.7 min (minor).

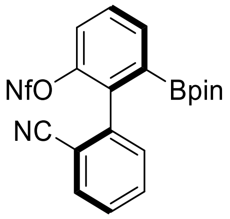

7va
$C_{23}H_{19}BF_9NO_5S$
M = 603.09 g/mol

(*R*)-2'-cyano-6-(4,4,5,5-tetramethyl-1,3,2-dioxaborolan-2-yl)-[1,1'-biphenyl]-2-yl 1,1,2,2,3,3,4,4,4-nonafluorobutane-1-sulfonate (**7va**): Prepared from **2v** (77.5 mg, 0.100 mmol, 1.00 equiv) according to **GP 3**. The residue was purified by flash column chromatography on silica gel using ethyl acetate and hexane as the eluent to afford **7va** as colorless oil (39.8 mg, 66% yield). **^{1}H NMR** (500 MHz, $CDCl_3$, 298 K) δ = 7.93 (dd, *J* = 7.2, 1.4 Hz, 1H), 7.72 (d, *J* = 7.8 Hz, 1H), 7.63–7.60 (m, 1H), 7.56–7.48 (m, 3H), 7.40 (d, *J* = 7.8 Hz, 1H), 1.13 (s, 6H), 1.10 (s, 6H) ppm. **^{13}C{^{1}H} NMR** (126 MHz, $CDCl_3$, 298 K) δ = 146.9, 139.9, 137.3, 135.5, 132.0, 131.6, 131.4, 130.1, 128.3, 123.9, 117.7, 114.3, 84.1, 24.6, 24.5 (nonaflate group not observed) ppm. **^{19}F NMR** (471 MHz, $CDCl_3$, 298 K) δ = −80.69 (t, *J* = 9.8 Hz), −108.28–(−111.39) (m), −108.50–(−122.41) (m), −124.77–(−127.14) (m) ppm. **^{11}B NMR** (160 MHz, $CDCl_3$, 298 K) δ = 30.17 ppm. **HRMS** (APCI) m/z: $[M+H]^+$ calcd for $C_{23}H_{20}BF_9NO_5S^+$ 604.1006; found 604.1006. Optical rotation: $[\alpha]_D^{20}$ = 12.3 (*c* 1.0, CH_2Cl_2, e.r. = 98:2). The enantiomeric excess of **7va** was determined by HPLC analysis on a chiral stationary phase (Daicel Chiralcel ID column, column temperature 20 °C, solvent heptane:*i*PrOH = 99:1, flow rate 0.6 mL/min): t_R = 8.1 min (minor), t_R = 11.6 min (major).

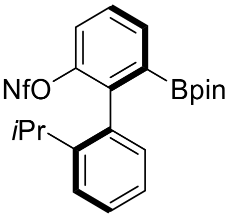

7wa
$C_{25}H_{26}BF_9O_5S$
M = 620.15 g/mol

(*R*)-2'-isopropyl-6-(4,4,5,5-tetramethyl-1,3,2-dioxaborolan-2-yl)-[1,1'-biphenyl]-2-yl 1,1,2,2,3,3,4,4,4-nonafluorobutane-1-sulfonate (**7wa**): Prepared from **2w** (87.6 mg, 0.100 mmol, 1.00 equiv) according to **GP 3**. The residue was purified by flash column chromatography on silica gel using ethyl acetate and hexane as the eluent to afford **7wa** as colorless oil (46.5 mg, 75% yield). **^{1}H NMR** (500 MHz, $CDCl_3$, 298 K) δ = 7.72 (dd, *J* = 7.1, 1.4 Hz, 1H), 7.45–7.37 (m, 2H), 7.35–7.31 (m, 2H), 7.16–7.13 (m, 1H), 7.07 (dd, *J* = 7.6, 1.4 Hz, 1H), 2.57–2.51 (m, 1H), 1.19 (d, *J* = 6.9 Hz, 3H), 1.07 (d, *J* = 7.1 Hz, 9H), 1.02 (s, 6H) ppm.

$^{13}C\{^1H\}$ NMR (126 MHz, $CDCl_3$, 298 K) δ = 147.5, 147.1, 140.2, 134.1, 133.7, 130.8, 128.6, 128.5, 124.8, 124.6, 122.5, 83.8, 30.6, 24.6, 24.2, 24.0, 23.2 (nonaflate group not observed) ppm. **^{19}F NMR** (471 MHz, $CDCl_3$, 298 K) δ = −80.74 (t, *J* = 9.8 Hz), −109.48–(−111.05) (m), −121.04–(−121.06) (m), −125.91–(−125.97) (m) ppm. **^{11}B NMR** (160 MHz, $CDCl_3$, 298 K) δ = 30.78 ppm. **HRMS** (APCI) m/z: $[M+H]^+$ calcd for $C_{25}H_{27}BF_9O_5S^+$ 621.1523; found 621.1519. Optical rotation: $[\alpha]_D^{20}$ = −37.2 (*c* 1.0, CH_2Cl_2, e.r. = 98:2). The enantiomeric excess of **7wa** was determined by HPLC analysis on a chiral stationary phase (Daicel Chiralcel IC column, column temperature 20 °C, solvent heptane:*i*PrOH = 99.8:0.2, flow rate 0.5 mL/min): t_R = 9.1 min (major), t_R = 10.4 min (minor).

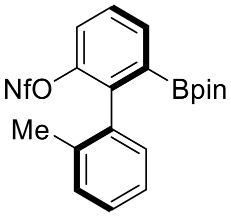

7xa
$C_{23}H_{22}BF_9O_5S$
M = 592.11 g/mol

(*R*)-2'-methyl-6-(4,4,5,5-tetramethyl-1,3,2-dioxaborolan-2-yl)-[1,1'-biphenyl]-2-yl 1,1,2,2,3,3,4,4,4-nonafluorobutane-1-sulfonate (**7xa**): Prepared from **2x** (76.4 mg, 0.100 mmol, 1.00 equiv) according to **GP 3**. The residue was purified by flash column chromatography on silica gel using ethyl acetate and hexane as the eluent to afford **7xa** as colorless oil (47.9 mg, 81% yield). **^1H NMR** (500 MHz, $CDCl_3$, 298 K) δ = 7.69 (d, *J* = 6.8 Hz, 1H), 7.39 (dt, *J* = 11.0, 7.8 Hz, 2H), 7.24 (dd, *J* = 8.8, 6.1 Hz, 1H), 7.16 (dd, *J* = 14.5, 7.2 Hz, 2H), 7.09 (d, *J* = 7.4 Hz, 1H), 2.04 (s, 3H), 1.05 (s, 6H), 1.02 (s, 6H) ppm. **$^{13}C\{^1H\}$ NMR** (101 MHz, $CDCl_3$, 298 K) δ = 147.2, 140.1, 136.7, 135.4, 133.7, 130.4, 129.3, 128.6, 128.1, 124.9, 122.9, 83.8, 24.4, 24.3, 19.8 (nonaflate group not observed) ppm. **^{19}F NMR** (471 MHz, $CDCl_3$, 298 K) δ = −80.73 (t, *J* = 9.8 Hz), −110.24–(−110.35) (m), −121.06–(−121.08) (m), −125.88–(−126.96) (m) ppm. **^{11}B NMR** (160 MHz, $CDCl_3$, 298 K) δ = 30.85 ppm. **HRMS** (APCI) m/z: $[M]^+$ calcd for $C_{23}H_{22}BF_9O_5S^+$ 592.1132; found 592.1238. Optical rotation: $[\alpha]_D^{20}$ = −2.1 (*c* 1.0, CH_2Cl_2, e.r. = 74:26). The enantiomeric excess of **7xa** was determined by HPLC analysis on a chiral stationary phase (Daicel Chiralcel IC column, column temperature 20 °C, solvent heptane:*i*PrOH = 99.9:0.1, flow rate 0.5 mL/min): t_R = 9.6 min (major), t_R = 11.3 min (minor).

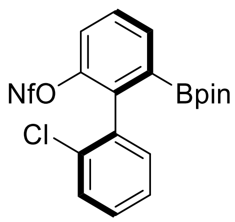

7ya
$C_{22}H_{19}BClF_9O_5S$
M = 612.06 g/mol

(*R*)-2'-chloro-6-(4,4,5,5-tetramethyl-1,3,2-dioxaborolan-2-yl)-[1,1'-biphenyl]-2-yl 1,1,2,2,3,3,4,4,4-nonafluorobutane-1-sulfonate (**7ya**): Prepared from **2y** (78.4 mg, 0.100 mmol, 1.00 equiv) according to **GP 3**. The residue was purified by flash column chromatography on silica gel using ethyl acetate and hexane as the eluent to afford **7ya** as colorless oil (37.3 mg, 61% yield). **^{1}H NMR** (400 MHz, $CDCl_3$, 298 K) δ = 7.82 (dd, *J* = 6.9, 1.8 Hz, 1H), 7.50–7.42 (m, 3H), 7.35–7.28 (m, 3H), 1.12 (s, 6H), 1.10 (s, 6H) ppm. **^{13}C{^{1}H} NMR** (101 MHz, $CDCl_3$, 298 K) δ = 147.1, 138.0, 134.9, 134.5, 134.0, 132.0, 129.4, 129.2, 128.8, 125.9, 123.3, 83.9, 24.5, 24.4 (nonaflate group not observed) ppm. **^{19}F NMR** (471 MHz, $CDCl_3$, 298 K) δ = −80.71 (t, *J* = 10.1 Hz), −110.13 (t, *J* = 14.1 Hz), −121.02–(−121.03) (m), −125.85–(−125.92) (m) ppm. **^{11}B NMR** (160 MHz, $CDCl_3$, 298 K) δ = 30.20 ppm. **HRMS** (APCI) m/z: $[M]^+$ calcd for $C_{22}H_{19}BClF_9O_5S^+$ 612.0586; found 612.0579. Optical rotation: $[\alpha]_D^{20}$ = 3.3 (*c* 1.0, CH_2Cl_2, e.r. = 88:12). The enantiomeric excess of **7ya** was determined by HPLC analysis on a chiral stationary phase (Daicel Chiralcel IA column, column temperature 20 °C, solvent heptane:*i*PrOH = 99.8:0.2, flow rate 0.5 mL/min): t_R = 10.2 min (minor), t_R = 10.7 min (major).

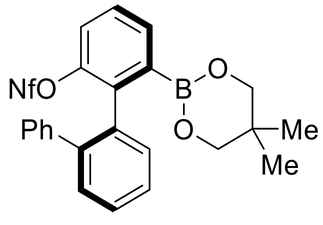

7lb
$C_{27}H_{22}BF_9O_5S$
M = 640.11 g/mol

(*R*)-6-(5,5-dimethyl-1,3,2-dioxaborinan-2-yl)-[1,1':2',1''-terphenyl]-2-yl 1,1,2,2,3,3,4,4,4-nonafluorobutane-1-sulfonate (**7lb**): Prepared from **2l** (82.6 mg, 0.100 mmol, 1.00 equiv) according to **GP 3**. The residue was purified by flash column chromatography on silica gel using ethyl acetate and hexane as the eluent to afford **7lb** as colorless oil (44.8 mg, 70% yield). **^{1}H NMR** (500 MHz, $CDCl_3$, 298 K) δ = 7.67 (d, *J* = 7.4 Hz, 1H), 7.42–7.39 (m, 2H), 7.36–7.35 (m, 1H), 7.30–7.24 (m, 2H), 7.19–7.15 (m, 5H), 7.07 (d, *J* = 8.4 Hz, 1H), 3.56 (d, *J* = 10.9 Hz, 2H), 3.47 (d, *J* = 10.9 Hz, 2H), 0.88 (s, 6H) ppm. **^{13}C{^{1}H} NMR** (101 MHz, $CDCl_3$, 298 K) δ = 147.0, 141.5, 141.3, 139.6, 135.1, 133.3, 131.4, 129.4, 129.4, 128.3, 128.2, 127.5, 126.5,

126.4, 122.1, 72.3, 31.6, 21.8 (nonaflate group not observed) ppm. **^{19}F NMR** (471 MHz, $CDCl_3$, 298 K) δ = −80.73–(−81.21) (m), −109.76–(−111.30) (m), −121.08–(−121.50) (m), −125.92 (t, *J* = 14.1 Hz) ppm. **^{11}B NMR** (160 MHz, $CDCl_3$, 298 K) δ = 27.05 ppm. **HRMS** (APCI) m/z: $[M]^+$ calcd for $C_{27}H_{22}BF_9O_5S^+$ 640.1132; found 640.1128. Optical rotation: $[\alpha]_D^{20}$ = 9.8 (*c* 1.0, CH_2Cl_2, e.r. = 95:5). The enantiomeric excess of **7lb** was determined by HPLC analysis on a chiral stationary phase (Daicel Chiralcel IA column, column temperature 20 °C, solvent heptane:*i*PrOH = 99.8:0.2, flow rate 0.6 mL/min): t_R = 11.4 min (minor), t_R = 15.8 min (major).

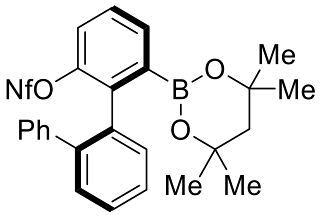

7lc
$C_{29}H_{26}BF_9O_5S$
M = 668.15 g/mol

(*R*)-6-(4,4,6,6-tetramethyl-1,3,2-dioxaborinan-2-yl)-[1,1':2',1''-terphenyl]-2-yl 1,1,2,2,3,3,4,4,4-nonafluorobutane-1-sulfonate (**7lc**): Prepared from **2l** (82.6 mg, 0.100 mmol, 1.00 equiv) according to **GP 3**. The residue was purified by flash column chromatography on silica gel using ethyl acetate and hexane as the eluent to afford **7lc** as colorless oil (35.4 mg, 53% yield). **^{1}H NMR** (500 MHz, $CDCl_3$, 298 K) δ = 7.64 (d, *J* = 7.4 Hz, 1H), 7.43–7.37 (m, 2H), 7.33–7.31 (m, 1H), 7.26–7.24 (m, 4H), 7.15 (s, 3H), 7.00 (d, *J* = 8.3 Hz, 1H), 1.75 (s, 2H), 1.24 (s, 6H), 1.07 (s, 6H) ppm. **^{13}C{^{1}H} NMR** (101 MHz, $CDCl_3$, 298 K) δ = 146.8, 141.7, 141.5, 139.4, 135.6, 132.8, 131.4, 129.4, 128.2, 128.1, 127.4, 126.5, 126.4, 121.5, 71.3, 48.6, 31.4, 31.2 (nonaflate group not observed) ppm. **^{19}F NMR** (471 MHz, $CDCl_3$, 298 K) δ = −80.74 (t, *J* = 9.9 Hz), −109.78–(−111.40) (m), −121.10, −125.92 (t, *J* = 14.1 Hz) ppm. **^{11}B NMR** (160 MHz, $CDCl_3$, 298 K) δ = 26.59 ppm. **HRMS** (APCI) m/z: $[M]^+$ calcd for $C_{29}H_{26}BF_9O_5S^+$ 668.1445; found 668.1439. Optical rotation: $[\alpha]_D^{20}$ = 4.6 (*c* 1.0, CH_2Cl_2, e.r. = 88:12). The enantiomeric excess of **7lc** was determined by HPLC analysis on a chiral stationary phase (Daicel Chiralcel IC column, column temperature 20 °C, solvent heptane:*i*PrOH = 99.8:0.2, flow rate 0.5 mL/min): t_R = 7.8 min (major), t_R = 9.0 min (minor).

5 1.0-mmol Scale Synthesis of (*R*)-7aa

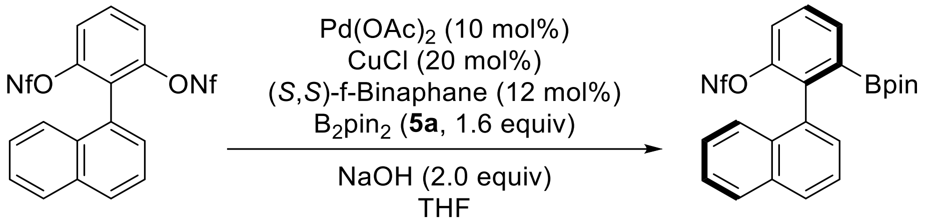

2a **7aa**

To a flame-dried Schlenk tube equipped with a septum and a magnetic stir bar were added $Pd(OAc)_2$ (10 mol%), CuCl (20 mol%) and (*S*,*S*)-f-Binaphane (12 mol%). The tube was evacuated under high vacuum and backfilled with nitrogen gas (3 times). THF (10 mL) was added to the tube, and the resulting mixture was stirred under room temperature for 30 minutes. Then nonaflates **2a** (1.0 mmol, 1.0 equiv), B_2pin_2 reagent **5a** (1.6 mmol, 1.6 equiv) and NaOH (2.0 mmol, 2.0 equiv) were successively added. The reaction was stirred at room temperature for 12 h (monitored by TLC). Then, the reaction mixture was filtered through a thin silicon pad. The organic layer was concentrated. The reaction mixture was purified by flash column chromatography on silica gel with ethyl acetate and hexane as the eluent to afford the product **7aa**.

6 Transformations of (*R*)-7aa

6.1 Reaction with NaN_3

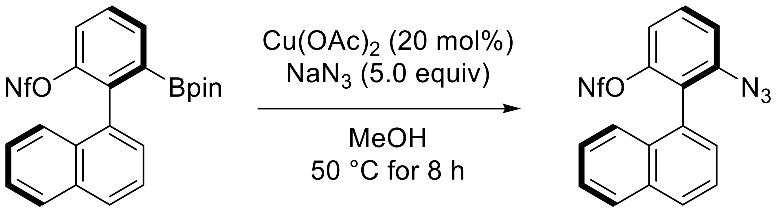

7aa, e.r. = 97:3 **11**, 72%, e.r. = 97:3

According to a reported procedure[S7]: To an oven dried sealed tube, **7aa** (0.10 mmol), NaN_3 (0.50 mmol), $Cu(OAc)_2$ (0.020 mmol) and MeOH (1 mL) was added. The flask was sealed under air, the reaction mixture was heated at 50 °C in an oil bath for 8 h (monitored by TLC), and the resulting mixture was evaporated under reduced pressure and the crude product was purified by flash column chromatography on silica gel with ethyl acetate and hexane as the eluent to afford the product **11** as colorless oil.

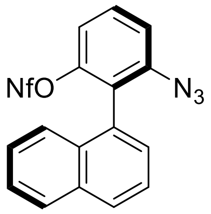

11
$C_{20}H_{10}F_9N_3O_3S$
M = 543.03 g/mol

(*R*)-3-azido-2-(naphthalen-1-yl)phenyl 1,1,2,2,3,3,4,4,4-nonafluorobutane-1-sulfonate (**11**): colorless oil. **^{1}H NMR** (500 MHz, $CDCl_3$, 298 K) δ = 7.98 (d, *J* = 8.4 Hz, 1H), 7.93 (d, *J* = 8.4 Hz, 1H), 7.59–7.56 (m, 2H), 7.52 (t, *J* = 7.7 Hz, 1H), 7.46–7.41 (m, 2H), 7.36–7.30 (m, 2H), 7.27 (d, *J* = 8.6 Hz, 1H) ppm. **^{13}C{^{1}H} NMR** (126 MHz, $CDCl_3$, 298 K) δ = 148.7, 141.9, 133.4, 131.7, 130.1, 129.6, 128.7, 128.5, 128.5, 126.6, 126.4, 126.1, 125.0, 124.9, 118.4, 117.6 (nonaflate group not observed) ppm. **^{19}F NMR** (471 MHz, $CDCl_3$, 298 K) δ = −80.78 (t, *J* = 9.9 Hz), −110.01 (t, *J* = 14.3 Hz), −121.16, −125.97–(−126.04) (m) ppm. **HRMS** (APCI) m/z: $[M-N_2+H_3]^+$ calcd for $C_{20}H_{13}F_9NO_3S^+$ 518.0467; found 518.0468. Optical rotation: $[\alpha]_D^{20}$ = 40.5 (*c* 1.0, CH_2Cl_2, e.r. = 97:3). The enantiomeric excess of **11** was determined by HPLC analysis on a chiral stationary phase (Daicel Chiralcel ODH column, column temperature 20 °C, solvent heptane:*i*PrOH = 99.9:0.1, flow rate 0.5 mL/min): t_R = 29.2 min (major), t_R = 35.1 min (minor).

6.2 Reaction with Imidazole

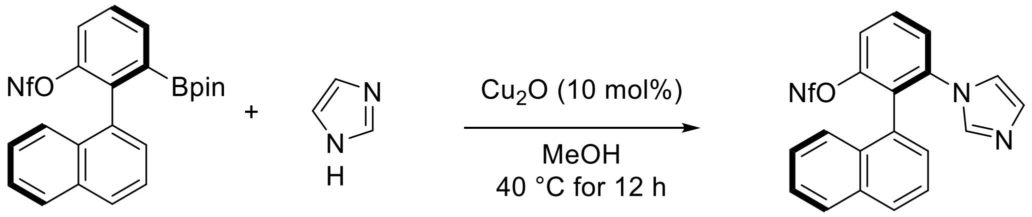

7aa, e.r. = 97:3 **12**, 85%, e.r. = 97:3

According to a reported procedure[S8]: A 10 mL Schlenk tube equipped with a magnetic stirrer bar was charged with **7aa** (0.10 mmol), Cu_2O (10 mol%, 0.010 mmol) and imidazole (0.15 mmol). MeOH (1 mL) were added via syringe under air, and the tube was sealed under air and heated at 40 °C for 12 h. After cooling to room temperature, the organic layers were concentrated under reduced pressure. The crude mixture was purified by column chromatography on silica gel to give the product **12** as colorless oil.

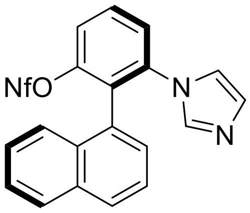

12
$C_{23}H_{13}F_9N_2O_3S$
M = 568.05 g/mol

(*R*)-3-(1H-imidazol-1-yl)-2-(naphthalen-1-yl)phenyl 1,1,2,2,3,3,4,4,4-nonafluorobutane-1-sulfonate (**12**): colorless oil. **^{1}H NMR** (500 MHz, $CDCl_3$, 298 K) δ = 7.88 (t, *J* = 9.7 Hz, 2H), 7.69–7.67 (m, 1H), 7.56 (dd, *J* = 15.7, 7.8 Hz, 2H), 7.47 (d, *J* = 7.7 Hz, 1H), 7.42–7.41 (m, 2H), 7.35 (d, *J* = 8.8 Hz, 1H), 7.26–7.23 (m, 2H), 6.75 (s, 1H), 6.62 (s, 1H) ppm. **^{13}C{^{1}H} NMR** (126 MHz, $CDCl_3$, 298 K) δ = 148.4, 138.9, 137.1, 133.4, 131.6, 130.6, 130.2, 129.9, 129.3, 128.7, 128.4, 127.9, 127.0, 126.3, 125.8, 125.0, 124.1, 121.5, 120.0 (nonaflate group not observed) ppm. **^{19}F NMR** (471 MHz, $CDCl_3$, 298 K) δ = −80.76 (t, *J* = 9.8 Hz), −109.89–(−110.00) (m), −121.15–(−121.16) (m), −125.97–(−126.04) (m) ppm. **HRMS** (APCI) m/z: $[M+H]^+$ calcd for $C_{23}H_{14}F_9N_2O_3S^+$ 569.0576; found 569.0574. Optical rotation: $[\alpha]_D^{20}$ = 36.3 (*c* 1.0, CH_2Cl_2, e.r. = 97:3). The enantiomeric excess of **12** was determined by HPLC analysis on a chiral stationary phase (Daicel Chiralcel ODH column, column temperature 20 °C, solvent heptane:*i*PrOH = 85:15, flow rate 0.5 mL/min): t_R = 16.0 min (major), t_R = 18.2 min (minor).

6.3 Reaction with H_2O_2

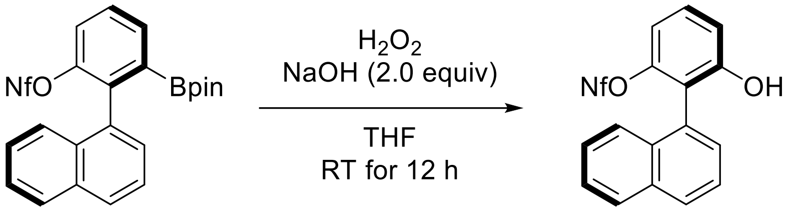

7aa, e.r. = 97:3 **13**, 90%, e.r. = 98:2

According to a reported procedure[S9]: To a flame-dried Schlenk tube equipped with a septum and a magnetic stir bar were added **7aa** (0.10 mmol) and THF (1.0 mL) under air. Then, NaOH aq. (2.0 M, 1.0 mL) and H_2O_2 aq. (30 wt%, 1.0 mL) were added. Then, the reaction stirred at room temperature for 12 h. After full conversion, the reaction mixture was diluted with water, then extracted with EtOAc for three times. The combined organic layer was dried over Na_2SO_4, filtered and concentrated. The residue was purified by silica gel on column chromatography to give compound **13** as colorless oil.

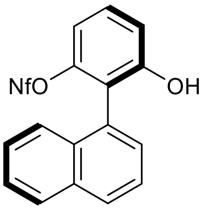

13
$C_{20}H_{11}F_9O_4S$
M = 518.02 g/mol

(*R*)-3-hydroxy-2-(naphthalen-1-yl)phenyl 1,1,2,2,3,3,4,4,4-nonafluorobutane-1-sulfonate (**13**): colorless oil. **^{1}H NMR** (500 MHz, $CDCl_3$, 298 K) δ = 8.01 (d, *J* = 8.3 Hz, 1H), 7.94 (d, *J* = 8.4 Hz, 1H), 7.62–7.43 (m, 6H), 7.13 (dd, *J* = 8.4, 2.5 Hz, 1H), 7.09 (d, *J* = 8.5 Hz, 1H), 4.98 (s, 1H) ppm. **^{13}C{^{1}H} NMR** (126 MHz, $CDCl_3$, 298 K) δ = 155.1, 147.9, 134.0, 131.8, 130.3, 130.1, 129.7, 128.6, 127.2, 126.7, 126.4, 125.6, 124.8, 120.6, 115.7, 113.5 (nonaflate group not observed) ppm. **^{19}F NMR** (471 MHz, $CDCl_3$, 298 K) δ = −80.81 (t, *J* = 10.0 Hz), −110.14 (t, *J* = 14.2 Hz), −121.20, −126.00–(−126.07) (m) ppm. **HRMS** (APCI) m/z: $[M]^+$ calcd for $C_{20}H_{11}F_9O_4S^+$ 518.0229; found 518.0228. Optical rotation: $[\alpha]_D^{20}$ = −30.0 (*c* 1.0, CH_2Cl_2, e.r. = 98:2). The enantiomeric excess of **13** was determined by HPLC analysis on a chiral stationary phase (Daicel Chiralcel ODH column, column temperature 20 °C, solvent heptane:*i*PrOH = 90:10, flow rate 0.7 mL/min): t_R = 8.3 min (major), t_R = 10.1 min (minor).

6.4 Reaction with PhI

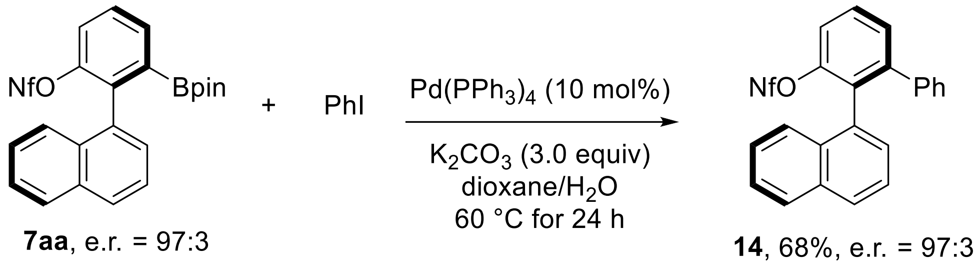

According to a reported procedure[S9]: To a flame-dried Schlenk tube equipped with a septum and a magnetic stir bar were added **7aa** (0.10 mmol), $Pd(PPh_3)_4$ (0.010 mol), and K_2CO_3 (0.30 mmol). The tube was evacuated under high vacuum and backfilled with nitrogen gas (3 times). Then, dioxane (1.0 mL), H_2O (0.20 mL) and PhI (0.30 mmol) were added to the tube. The mixture was stirred at 60 °C for 24 h. After cooling to room temperature, water was added and the mixture was extracted with EtOAc three times. The combined organic layer was dried over Na_2SO_4. After removal of the solvent under reduced pressure, the residue was purified by silica gel on column chromatography to give compound **14** as colorless oil.

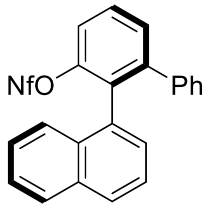

14
$C_{26}H_{15}F_9O_3S$
M = 578.06 g/mol

(*R*)-2-(naphthalen-1-yl)-[1,1'-biphenyl]-3-yl 1,1,2,2,3,3,4,4,4-nonafluorobutane-1-sulfonate (**14**): colorless oil. **^{1}H NMR** (500 MHz, $CDCl_3$, 298 K) δ = 7.80 (dd, *J* = 16.4, 8.2 Hz, 2H), 7.62–7.56 (m, 2H), 7.48–7.33 (m, 5H), 7.22–7.20 (m, 1H), 7.05–7.01 (m, 5H) ppm. **^{13}C{^{1}H} NMR** (126 MHz, $CDCl_3$, 298 K) δ = 148.2, 145.4, 139.6, 133.2, 132.9, 132.3, 131.5, 130.2, 129.5, 129.3, 128.8, 128.6, 128.3, 127.7, 127.1, 126.3, 125.7, 125.3, 124.8, 120.2 (nonaflate group not observed) ppm. **^{19}F NMR** (471 MHz, $CDCl_3$, 298 K) δ = −80.78 (t, *J* = 10.0 Hz), −110.26–(−110.38) (m), −121.20–(−121.22) (m), −125.98–(−126.05) (m) ppm. **HRMS** (APCI) m/z: $[M]^+$ calcd for $C_{26}H_{15}F_9O_3S^+$ 578.0593; found 578.0593. Optical rotation: $[\alpha]_D^{20}$ = 47.7 (*c* 1.0, CH_2Cl_2, e.r. = 97:3). The enantiomeric excess of **14** was determined by HPLC analysis on a chiral stationary phase (Daicel Chiralcel ODH column, column temperature 20 °C, solvent heptane:*i*PrOH = 99.9:0.1, flow rate 0.5 mL/min): t_R = 14.7 min (minor), t_R = 20.5 min (major).

6.5 Control Experiments

A Desymmetrization

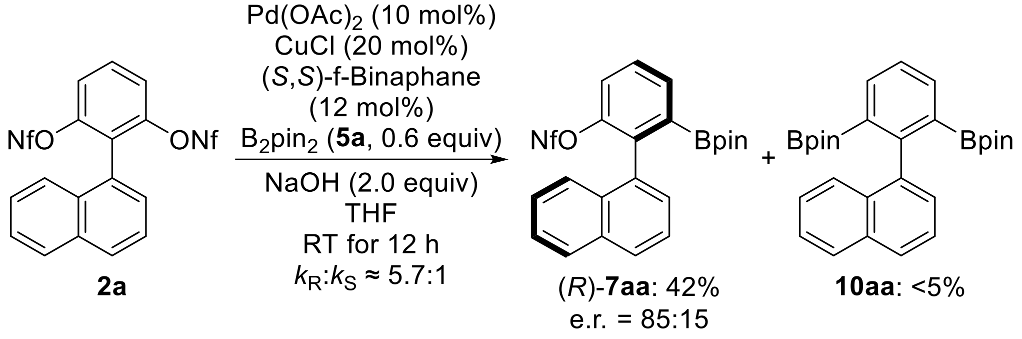

To a flame-dried Schlenk tube equipped with a septum and a magnetic stir bar were added $Pd(OAc)_2$ (10 mol%), CuCl (20 mol%) and (*S*,*S*)-f-Binaphane (12 mol%). The tube was evacuated under high vacuum and backfilled with nitrogen gas (3 times). THF (1 mL) was added to the tube, and the resulting mixture was stirred under room temperature for 30 minutes. Then nonaflates **2a** (0.10 mmol, 1.0 equiv), B_2pin_2 (0.6 equiv) and NaOH (2.0 equiv) were successively added. The reaction was stirred at room temperature for 12 h. Then, the reaction mixture was filtered through a thin silicon pad. The organic layer was concentrated. The reaction mixture was purified by flash column chromatography on silica gel with ethyl acetate and hexane as the eluent to afford the product **7aa** in 42% yield with e.r. = 85:15 and trace amount **10aa**.

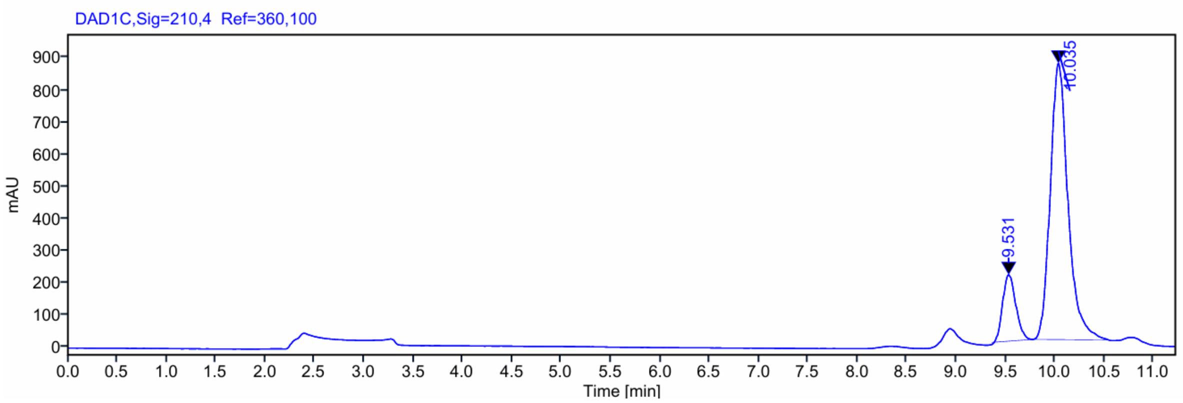

Signal: DAD1C,Sig=210,4 Ref=360,100

RT [min]	Width [min]	Area	Height	Area%
9.531	0.42	1907.52	206.26	15.05
10.035	0.76	10766.33	858.71	84.95
	**Sum**	**12673.85**		

B Kinetic resolution

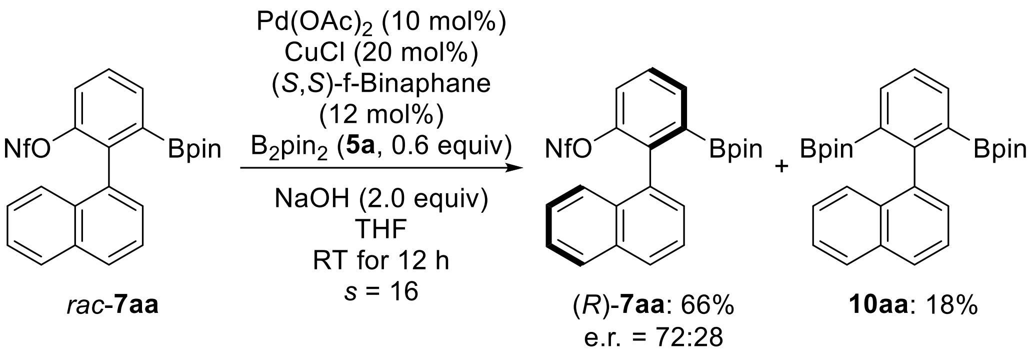

To a flame-dried Schlenk tube equipped with a septum and a magnetic stir bar were added $Pd(OAc)_2$ (10 mol%), CuCl (20 mol%) and (*S*,*S*)-f-Binaphane (12 mol%). The tube was evacuated under high vacuum and backfilled with nitrogen gas (3 times). THF (1 mL) was added to the tube, and the resulting mixture was stirred under room temperature for 30 minutes. Then *rac*-**7aa** (0.10 mmol, 1.0 equiv), B_2pin_2 (0.6 equiv) and NaOH (2.0 equiv) were successively added. The reaction was stirred at room temperature for 12 h. Then, the reaction mixture was filtered through a thin silicon pad. The organic layer was concentrated. The reaction mixture was purified by flash column chromatography on silica gel with ethyl acetate and hexane as the eluent to afford the product **7aa** in 66% yield with e.r. = 72:28 and **10aa** in 18% yield.

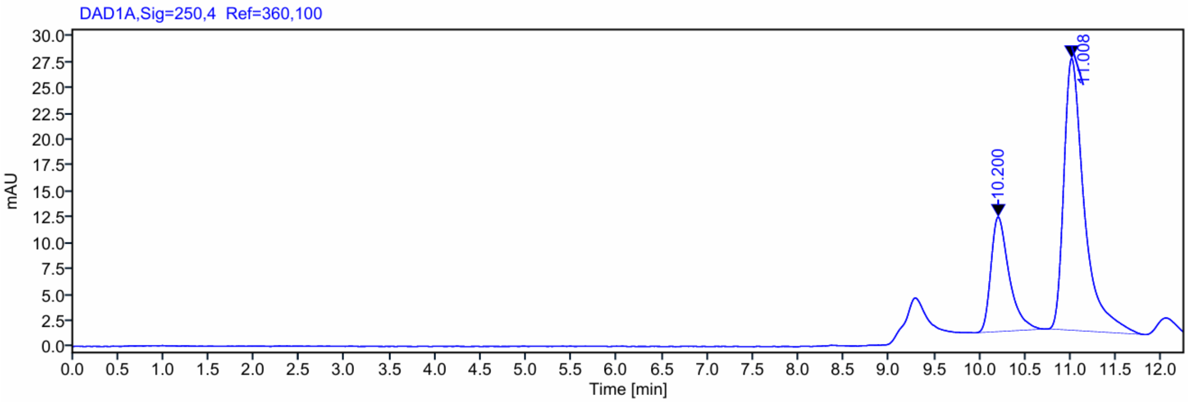

Signal: DAD1A,Sig=250,4 Ref=360,100

RT [min]	Width [min]	Area	Height	Area%
10.200	0.81	155.51	11.08	27.31
11.008	1.07	414.01	26.26	72.69
	**Sum**	**569.52**		

7 Determination of the Absolute Configuration

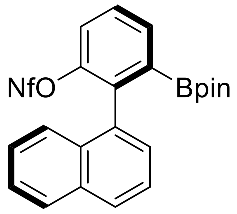

7aa
$C_{26}H_{22}BF_9O_5S$
M = 628.11 g/mol

Crystal (*R*)- **7aa** was obtained through recrystallization in the solution of pentane at room temperature. The absolute configuration of **7aa** was confirmed unambiguously by X-ray diffraction analysis, and other compounds were assigned by analogy. CCDC 2449055 contains the supplementary crystallographic data for this compound.

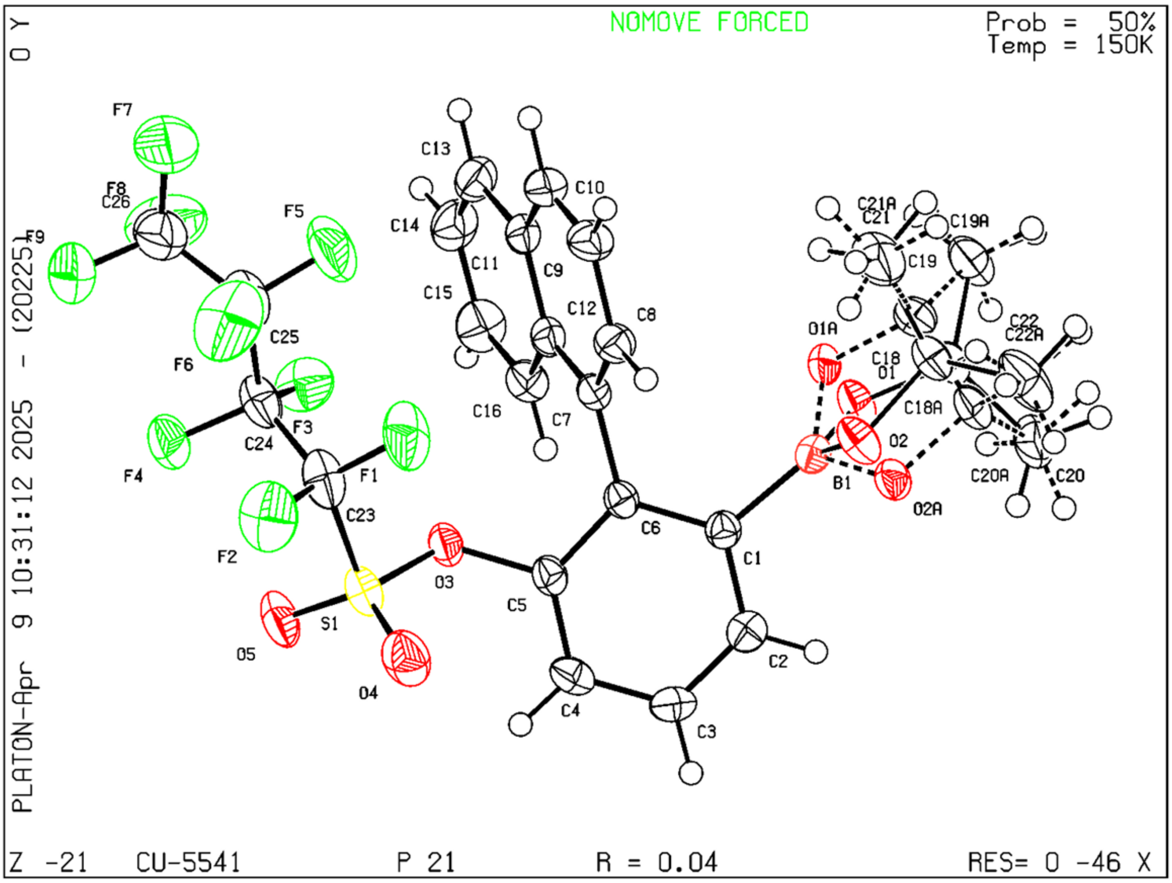

Figure S1. Molecular structure of (*R*)- **7aa**.

Table S2. Crystal data and structure refinement for cu-5541.

Identification code	CU-5541	
Empirical formula	C26 H22 B F9 O5 S	
Formula weight	628.30	
Temperature	150.01(10) K	
Wavelength	1.54184 Å	
Crystal system	Monoclinic	
Space group	$P2_1$ (No. 4)	
Unit cell dimensions	a = 13.4176(3) Å	α= 90°.
	b = 7.5006(2) Å	β= 101.951(2)°.
	c = 13.8439(3) Å	γ = 90°.
Volume	1363.05(6) Å^3	
Z	2	
Density (calculated)	1.531 Mg/m^3	
Absorption coefficient	1.961 mm^{-1}	
F(000)	640	
Crystal size	0.356 x 0.280 x 0.042 mm^3	
Theta range for data collection	3.263 to 72.647°.	
Index ranges	-14<=h<=16, -9<=k<=8, -16<=l<=17	
Reflections collected	9454	
Independent reflections	4144 [R(int) = 0.0237]	
Completeness to theta = 67.684°	99.9 %	
Absorption correction	Semi-empirical from equivalents	
Max. and min. transmission	1.00000 and 0.01129	
Refinement method	Full-matrix least-squares on F^2	
Data / restraints / parameters	4144 / 255 / 417	
Goodness-of-fit on F^2	1.040	
Final R indices [I>2sigma(I)]	R1 = 0.0354, wR2 = 0.0953	
R indices (all data)	R1 = 0.0376, wR2 = 0.0976	
Absolute structure parameter	0.40(2)	
Extinction coefficient	n/a	
Largest diff. peak and hole	0.195 and -0.376 e.Å^{-3}	

Table S3. Atomic coordinates (x 10^4) and equivalent isotropic displacement parameters ($Å^2$x 10^3) for cu-5541. U(eq) is defined as one third of the trace of the orthogonalized U^{ij} tensor.

	x	y	z	U(eq)
S(1)	1951(1)	3369(1)	8429(1)	36(1)
F(1)	2860(1)	6364(3)	8278(2)	61(1)
F(2)	1393(2)	6517(4)	8713(2)	64(1)
F(3)	1706(2)	5057(3)	6409(2)	53(1)
F(4)	364(1)	5913(3)	6938(2)	53(1)
F(5)	2468(2)	8253(4)	6541(3)	98(1)
F(6)	1454(2)	9189(4)	7463(2)	86(1)
F(7)	1088(2)	10322(5)	5510(3)	99(1)
F(8)	712(3)	7628(5)	5060(2)	93(1)
F(9)	-137(2)	9005(5)	5975(2)	81(1)
O(3)	2749(1)	2650(3)	7850(1)	31(1)
O(4)	2320(2)	3306(5)	9464(2)	50(1)
O(5)	985(2)	2662(4)	7994(2)	45(1)
C(1)	5510(2)	1769(4)	8550(2)	25(1)
C(2)	5505(2)	343(4)	9197(2)	31(1)
C(3)	4607(2)	-323(4)	9401(2)	32(1)
C(4)	3688(2)	464(4)	8967(2)	32(1)
C(5)	3702(2)	1875(4)	8338(2)	27(1)
C(6)	4582(2)	2573(4)	8096(2)	24(1)
C(7)	4563(2)	4093(4)	7408(2)	25(1)
C(8)	5001(2)	5694(4)	7754(2)	31(1)
C(9)	5004(2)	7161(4)	7116(2)	37(1)
C(10)	4576(2)	7027(5)	6139(2)	36(1)
C(11)	4117(2)	5408(4)	5749(2)	31(1)
C(12)	4117(2)	3913(4)	6378(2)	27(1)
C(13)	3660(2)	5247(5)	4728(2)	40(1)
C(14)	3221(2)	3684(6)	4362(2)	45(1)
C(15)	3238(3)	2196(5)	4974(2)	43(1)
C(16)	3680(2)	2290(4)	5961(2)	32(1)
C(23)	1904(2)	5744(5)	8088(3)	42(1)
C(24)	1372(2)	6173(5)	7016(3)	40(1)
C(25)	1527(3)	8117(5)	6704(3)	57(1)
C(26)	777(3)	8782(6)	5794(4)	60(1)
B(1)	6574(2)	2433(4)	8384(2)	26(1)

O(1)	6995(2)	1838(6)	7633(2)	33(1)
O(2)	7181(2)	3528(6)	9029(2)	35(1)
C(17)	8070(2)	2383(6)	7874(3)	30(1)
C(18)	8054(3)	3977(6)	8579(3)	33(1)
C(19)	8406(2)	2856(6)	6921(2)	47(1)
C(20)	8666(2)	795(6)	8424(4)	55(1)
C(21)	7784(3)	5714(6)	7988(4)	64(1)
C(22)	9013(3)	4114(7)	9405(3)	58(1)
O(1A)	6810(8)	2830(30)	7507(8)	32(3)
O(2A)	7386(8)	2560(20)	9172(8)	30(3)
C(17A)	7866(11)	3560(30)	7738(12)	34(3)
C(18A)	8292(11)	2760(30)	8750(12)	32(3)
C(19A)	8406(2)	2856(6)	6921(2)	47(1)
C(20A)	8666(2)	795(6)	8424(4)	55(1)
C(21A)	7784(3)	5714(6)	7988(4)	64(1)
C(22A)	9013(3)	4114(7)	9405(3)	58(1)

Table S4. Bond lengths [Å] and angles [°] for cu-5541.

S(1)-O(5)	1.414(2)
S(1)-O(4)	1.416(2)
S(1)-O(3)	1.561(2)
S(1)-C(23)	1.841(4)
F(1)-C(23)	1.338(4)
F(2)-C(23)	1.343(4)
F(3)-C(24)	1.328(4)
F(4)-C(24)	1.349(3)
F(5)-C(25)	1.333(4)
F(6)-C(25)	1.343(5)
F(7)-C(26)	1.316(5)
F(8)-C(26)	1.324(6)
F(9)-C(26)	1.313(5)
O(3)-C(5)	1.440(3)
C(1)-C(2)	1.396(4)
C(1)-C(6)	1.409(3)
C(1)-B(1)	1.573(4)
C(2)-C(3)	1.386(4)
C(3)-C(4)	1.387(4)
C(4)-C(5)	1.373(4)
C(5)-C(6)	1.394(3)
C(6)-C(7)	1.483(4)
C(7)-C(8)	1.379(4)
C(7)-C(12)	1.434(4)
C(8)-C(9)	1.412(4)
C(9)-C(10)	1.358(4)
C(10)-C(11)	1.417(5)
C(11)-C(12)	1.420(4)
C(11)-C(13)	1.425(4)
C(12)-C(16)	1.421(4)
C(13)-C(14)	1.362(6)
C(14)-C(15)	1.398(6)
C(15)-C(16)	1.375(4)
C(23)-C(24)	1.541(5)
C(24)-C(25)	1.547(5)
C(25)-C(26)	1.524(6)
B(1)-O(1A)	1.351(12)

B(1)-O(2)	1.355(4)
B(1)-O(1)	1.358(4)
B(1)-O(2A)	1.377(11)
O(1)-C(17)	1.469(4)
O(2)-C(18)	1.474(4)
C(17)-C(19)	1.522(5)
C(17)-C(20)	1.545(5)
C(17)-C(18)	1.547(6)
C(18)-C(22)	1.537(5)
C(18)-C(21)	1.542(6)
O(1A)-C(17A)	1.490(18)
O(2A)-C(18A)	1.461(18)
C(17A)-C(18A)	1.52(2)
C(17A)-C(19A)	1.557(17)
C(17A)-C(21A)	1.66(2)
C(18A)-C(22A)	1.559(16)
C(18A)-C(20A)	1.65(2)

O(5)-S(1)-O(4)	121.08(16)
O(5)-S(1)-O(3)	108.52(13)
O(4)-S(1)-O(3)	111.98(12)
O(5)-S(1)-C(23)	105.92(15)
O(4)-S(1)-C(23)	106.15(18)
O(3)-S(1)-C(23)	101.10(14)
C(5)-O(3)-S(1)	122.37(17)
C(2)-C(1)-C(6)	119.5(2)
C(2)-C(1)-B(1)	117.5(2)
C(6)-C(1)-B(1)	123.0(2)
C(3)-C(2)-C(1)	121.8(3)
C(2)-C(3)-C(4)	119.4(3)
C(5)-C(4)-C(3)	118.4(2)
C(4)-C(5)-C(6)	124.5(2)
C(4)-C(5)-O(3)	118.8(2)
C(6)-C(5)-O(3)	116.7(2)
C(5)-C(6)-C(1)	116.5(2)
C(5)-C(6)-C(7)	122.8(2)
C(1)-C(6)-C(7)	120.8(2)
C(8)-C(7)-C(12)	119.2(3)
C(8)-C(7)-C(6)	119.8(2)

C(12)-C(7)-C(6)	121.0(2)
C(7)-C(8)-C(9)	121.1(3)
C(10)-C(9)-C(8)	120.7(3)
C(9)-C(10)-C(11)	120.2(3)
C(10)-C(11)-C(12)	119.9(3)
C(10)-C(11)-C(13)	121.0(3)
C(12)-C(11)-C(13)	119.1(3)
C(11)-C(12)-C(16)	118.7(2)
C(11)-C(12)-C(7)	118.8(3)
C(16)-C(12)-C(7)	122.5(3)
C(14)-C(13)-C(11)	120.4(3)
C(13)-C(14)-C(15)	120.7(3)
C(16)-C(15)-C(14)	120.7(3)
C(15)-C(16)-C(12)	120.3(3)
F(1)-C(23)-F(2)	108.7(3)
F(1)-C(23)-C(24)	110.6(3)
F(2)-C(23)-C(24)	109.5(3)
F(1)-C(23)-S(1)	107.8(2)
F(2)-C(23)-S(1)	104.3(3)
C(24)-C(23)-S(1)	115.7(2)
F(3)-C(24)-F(4)	108.7(3)
F(3)-C(24)-C(23)	109.1(3)
F(4)-C(24)-C(23)	107.7(3)
F(3)-C(24)-C(25)	109.6(3)
F(4)-C(24)-C(25)	107.7(3)
C(23)-C(24)-C(25)	113.8(3)
F(5)-C(25)-F(6)	108.2(4)
F(5)-C(25)-C(26)	108.8(4)
F(6)-C(25)-C(26)	108.0(3)
F(5)-C(25)-C(24)	107.8(3)
F(6)-C(25)-C(24)	108.0(3)
C(26)-C(25)-C(24)	115.8(3)
F(9)-C(26)-F(7)	108.2(4)
F(9)-C(26)-F(8)	108.3(4)
F(7)-C(26)-F(8)	108.6(4)
F(9)-C(26)-C(25)	111.5(4)
F(7)-C(26)-C(25)	109.8(4)
F(8)-C(26)-C(25)	110.3(4)
O(2)-B(1)-O(1)	114.5(3)

O(1A)-B(1)-O(2A)	113.7(7)
O(1A)-B(1)-C(1)	126.2(5)
O(2)-B(1)-C(1)	122.4(3)
O(1)-B(1)-C(1)	122.8(3)
O(2A)-B(1)-C(1)	120.0(5)
B(1)-O(1)-C(17)	106.2(3)
B(1)-O(2)-C(18)	106.4(3)
O(1)-C(17)-C(19)	108.7(3)
O(1)-C(17)-C(20)	106.4(3)
C(19)-C(17)-C(20)	113.0(3)
O(1)-C(17)-C(18)	102.5(3)
C(19)-C(17)-C(18)	114.4(4)
C(20)-C(17)-C(18)	111.0(3)
O(2)-C(18)-C(22)	108.5(3)
O(2)-C(18)-C(21)	107.3(3)
C(22)-C(18)-C(21)	114.0(4)
O(2)-C(18)-C(17)	101.9(3)
C(22)-C(18)-C(17)	113.7(4)
C(21)-C(18)-C(17)	110.5(3)
B(1)-O(1A)-C(17A)	106.2(9)
B(1)-O(2A)-C(18A)	106.1(9)
O(1A)-C(17A)-C(18A)	102.1(12)
O(1A)-C(17A)-C(19A)	106.4(11)
C(18A)-C(17A)-C(19A)	112.9(14)
O(1A)-C(17A)-C(21A)	107.6(12)
C(18A)-C(17A)-C(21A)	102.8(12)
C(19A)-C(17A)-C(21A)	123.0(11)
O(2A)-C(18A)-C(17A)	102.9(12)
O(2A)-C(18A)-C(22A)	107.5(12)
C(17A)-C(18A)-C(22A)	110.2(14)
O(2A)-C(18A)-C(20A)	110.2(13)
C(17A)-C(18A)-C(20A)	100.4(12)
C(22A)-C(18A)-C(20A)	123.7(10)

Symmetry transformations used to generate equivalent atoms:

Table S5. Anisotropic displacement parameters ($Å^2$x 10^3) for cu-5541. The anisotropic displacement factor exponent takes the form: $-2\pi^2[h^2 a^{*2}U^{11} + ... + 2 h k a^* b^* U^{12}]$

	U^{11}	U^{22}	U^{33}	U^{23}	U^{13}	U^{12}
S(1)	24(1)	43(1)	42(1)	-7(1)	10(1)	-3(1)
F(1)	34(1)	44(1)	97(2)	-16(1)	-4(1)	-13(1)
F(2)	70(1)	62(2)	64(1)	-24(1)	20(1)	16(1)
F(3)	69(1)	40(1)	53(1)	-4(1)	19(1)	10(1)
F(4)	27(1)	56(1)	75(1)	7(1)	5(1)	-7(1)
F(5)	39(1)	61(2)	202(3)	42(2)	40(2)	-2(1)
F(6)	97(2)	37(1)	113(2)	-23(2)	-5(2)	6(1)
F(7)	77(2)	62(2)	161(3)	48(2)	35(2)	2(2)
F(8)	132(3)	85(2)	67(2)	16(2)	34(2)	10(2)
F(9)	46(1)	86(2)	114(2)	34(2)	22(1)	18(1)
O(3)	20(1)	38(1)	34(1)	0(1)	5(1)	-1(1)
O(4)	42(1)	70(2)	42(1)	-6(1)	16(1)	3(1)
O(5)	23(1)	45(1)	70(1)	-6(1)	12(1)	-5(1)
C(1)	24(1)	27(1)	24(1)	-1(1)	4(1)	0(1)
C(2)	31(1)	30(2)	30(1)	0(1)	4(1)	1(1)
C(3)	40(1)	30(2)	28(1)	6(1)	7(1)	-4(1)
C(4)	32(1)	34(2)	32(1)	-1(1)	10(1)	-10(1)
C(5)	21(1)	31(2)	28(1)	-2(1)	3(1)	-3(1)
C(6)	21(1)	25(1)	24(1)	-3(1)	4(1)	-2(1)
C(7)	20(1)	26(1)	30(1)	2(1)	7(1)	0(1)
C(8)	30(1)	29(2)	34(1)	1(1)	5(1)	-5(1)
C(9)	38(1)	28(2)	45(2)	3(1)	12(1)	-4(1)
C(10)	35(1)	33(2)	42(2)	10(1)	11(1)	2(1)
C(11)	24(1)	38(2)	32(1)	6(1)	9(1)	6(1)
C(12)	22(1)	30(1)	30(1)	0(1)	7(1)	3(1)
C(13)	37(2)	49(2)	33(1)	8(2)	7(1)	11(1)
C(14)	43(2)	61(3)	28(1)	1(2)	1(1)	6(2)
C(15)	44(2)	49(2)	35(2)	-9(2)	4(1)	-4(2)
C(16)	31(1)	35(2)	31(1)	-2(1)	8(1)	-3(1)
C(23)	30(1)	38(2)	59(2)	-19(2)	9(1)	-4(1)
C(24)	28(1)	36(2)	57(2)	-11(2)	13(1)	-6(1)
C(25)	39(2)	35(2)	98(3)	-4(2)	18(2)	-2(1)
C(26)	53(2)	49(2)	84(3)	14(2)	25(2)	2(2)
B(1)	24(1)	25(2)	26(1)	0(1)	2(1)	1(1)

O(1)	22(1)	46(2)	31(1)	-10(1)	7(1)	-7(1)
O(2)	28(1)	40(2)	38(2)	-12(2)	13(1)	-10(1)
C(17)	20(2)	36(2)	32(2)	-3(2)	6(1)	-4(1)
C(18)	25(2)	35(2)	40(2)	-5(2)	11(1)	-7(2)
C(19)	29(1)	75(3)	36(1)	0(2)	10(1)	-12(2)
C(20)	32(2)	40(2)	97(3)	15(2)	20(2)	7(1)
C(21)	51(2)	31(2)	118(4)	14(2)	32(2)	-2(2)
C(22)	35(2)	96(3)	45(2)	-21(2)	10(1)	-30(2)
O(1A)	18(5)	45(8)	32(5)	8(6)	1(4)	0(5)
O(2A)	21(5)	38(8)	29(5)	1(5)	2(4)	-5(5)
C(17A)	30(6)	30(7)	41(7)	3(6)	6(5)	-9(6)
C(18A)	21(6)	41(8)	36(6)	-4(6)	8(5)	-8(5)
C(19A)	29(1)	75(3)	36(1)	0(2)	10(1)	-12(2)
C(20A)	32(2)	40(2)	97(3)	15(2)	20(2)	7(1)
C(21A)	51(2)	31(2)	118(4)	14(2)	32(2)	-2(2)
C(22A)	35(2)	96(3)	45(2)	-21(2)	10(1)	-30(2)

8 HPLC Traces

(*R*)-2-(naphthalen-1-yl)-3-(4,4,5,5-tetramethyl-1,3,2-dioxaborolan-2-yl)phenyl 1,1,2,2,3,3,4,4,4-nonafluorobutane-1-sulfonate (7aa)

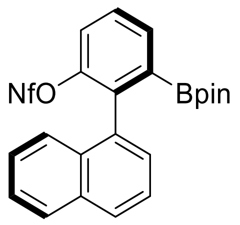

7aa

Figure S2. *rac*-**7aa**

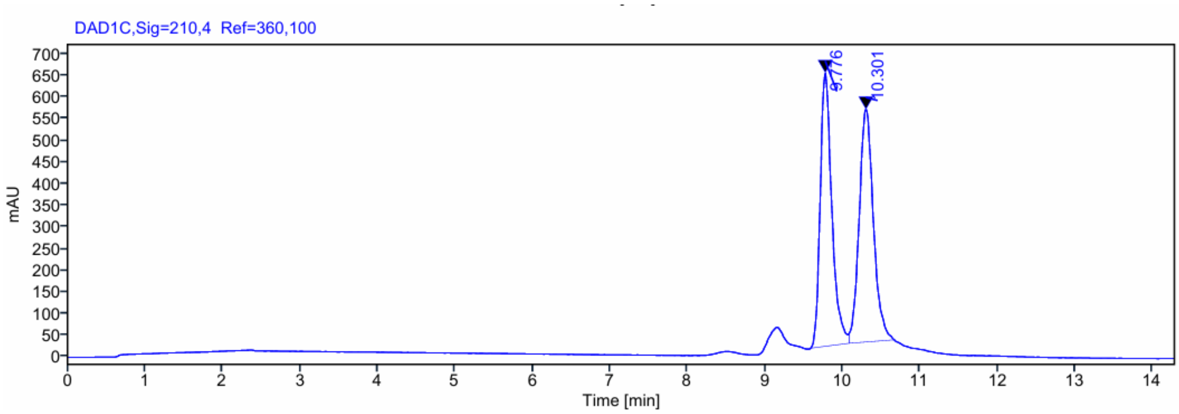

Signal: DAD1C,Sig=210,4 Ref=360,100

RT [min]	Width [min]	Area	Height	Area%
9.776	0.57	6570.75	631.80	49.01
10.301	0.58	6836.10	537.46	50.99
	**Sum**	**13406.85**		

Figure S3. (*R*)-**7aa** (e.r. = 97:3)

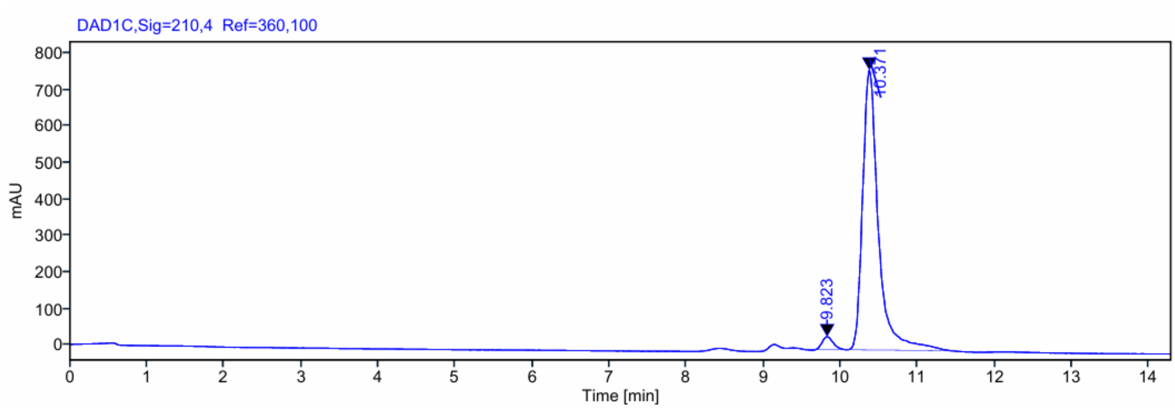

Signal: DAD1C,Sig=210,4 Ref=360,100

RT [min]	Width [min]	Area	Height	Area%
9.823	0.51	354.49	35.84	3.15
10.371	1.35	10907.06	766.67	96.85
	**Sum**	**11261.56**		

(*R*)-2-(4-methylnaphthalen-1-yl)-3-(4,4,5,5-tetramethyl-1,3,2-dioxaborolan-2-yl)phenyl 1,1,2,2,3,3,4,4,4-nonafluorobutane-1-sulfonate (**7ba**)

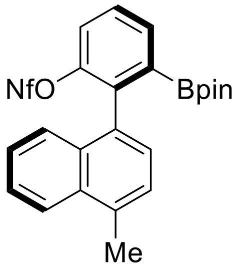

7ba

Figure S4. *rac*-**7ba**

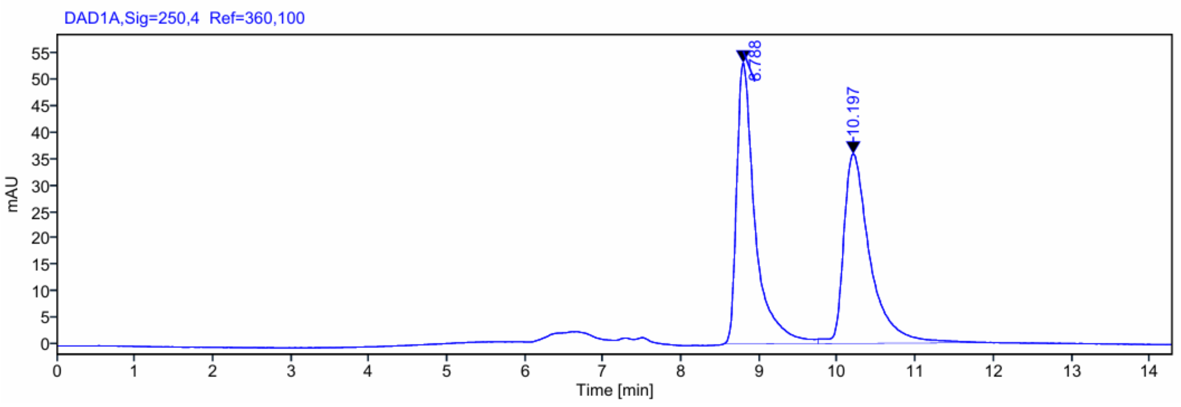

Signal: DAD1A,Sig=250,4 Ref=360,100

RT [min]	Width [min]	Area	Height	Area%
8.788	1.22	872.27	53.09	50.78
10.197	2.31	845.32	35.78	49.22
	**Sum**	**1717.59**		

Figure S5. (*R*)-**7ba** (e.r. = 95:5)

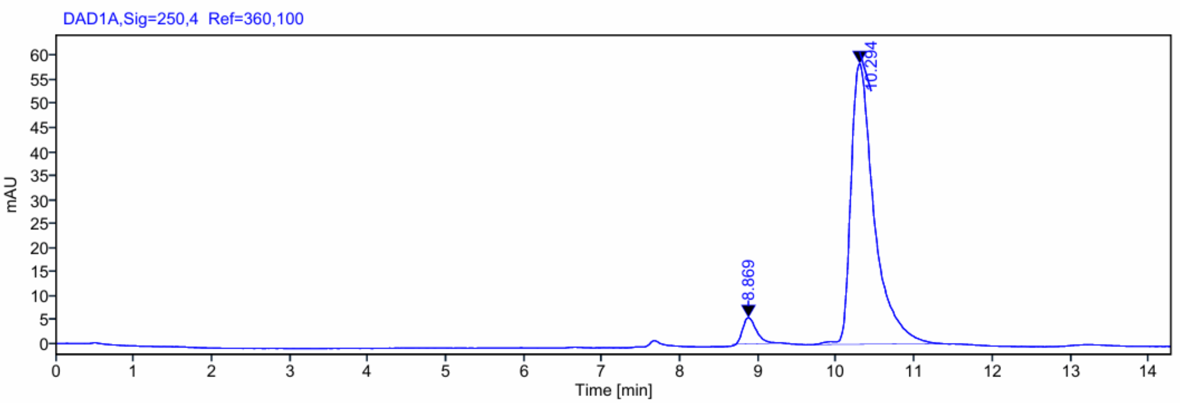

Signal: DAD1A,Sig=250,4 Ref=360,100

RT [min]	Width [min]	Area	Height	Area%
8.869	0.64	65.60	5.45	5.15
10.294	1.64	1207.93	58.42	94.85
	**Sum**	**1273.53**		

(*R*)-2-(4-fluoronaphthalen-1-yl)-3-(4,4,5,5-tetramethyl-1,3,2-dioxaborolan-2-yl)phenyl 1,1,2,2,3,3,4,4,4-nonafluorobutane-1-sulfonate (**7ca**)

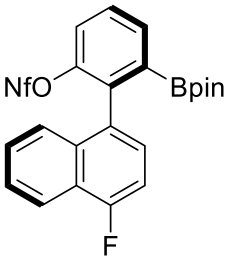

7ca

Figure S6. *rac*-**7ca**

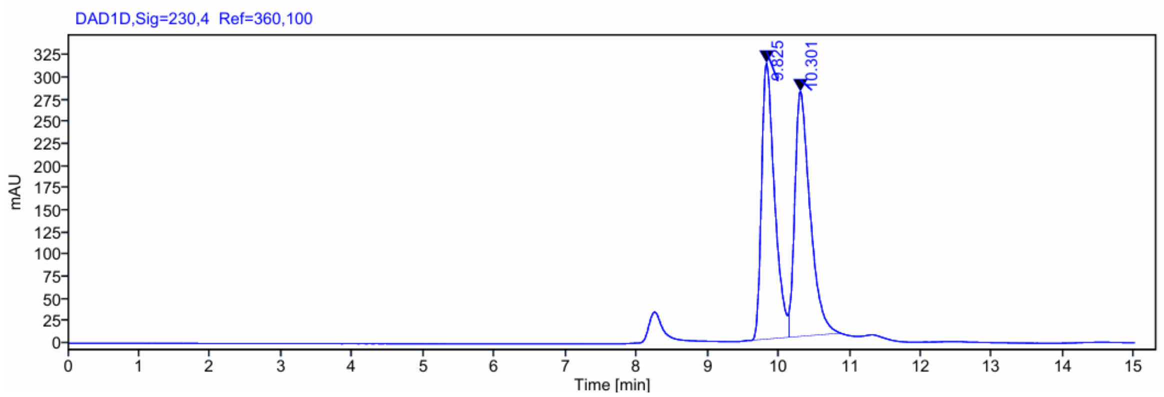

**Signal:**	DAD1D,Sig=230,4 Ref=360,100			
**RT [min]**	**Width [min]**	**Area**	**Height**	**Area%**
9.825	0.62	3989.02	312.38	49.08
10.301	0.72	4139.30	277.01	50.92
	**Sum**	**8128.33**		

Figure S7. (*R*)-**7ca** (e.r. = 92:8)

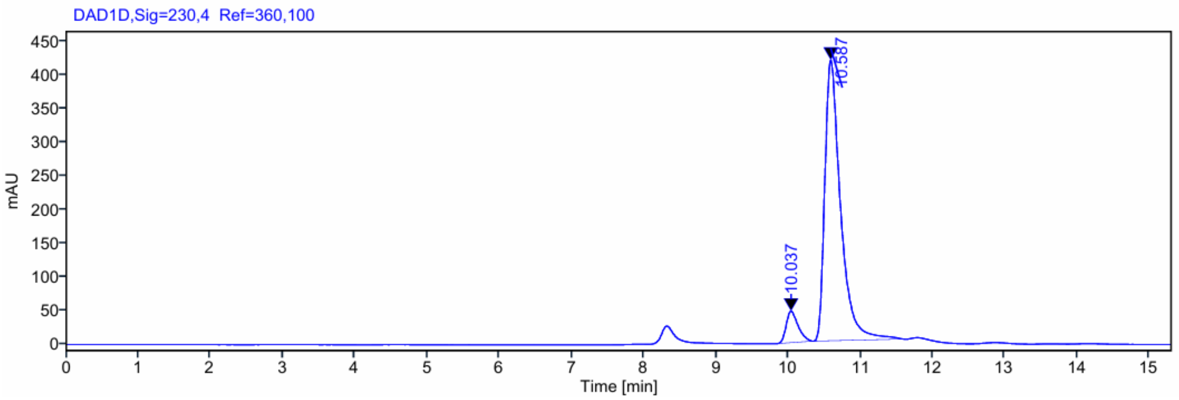

**Signal:**	DAD1D,Sig=230,4 Ref=360,100			
**RT [min]**	**Width [min]**	**Area**	**Height**	**Area%**
10.037	0.51	530.75	46.72	7.75
10.587	1.26	6315.46	415.92	92.25
	**Sum**	**6846.21**		

(*R*)-2-(4-bromonaphthalen-1-yl)-3-(4,4,5,5-tetramethyl-1,3,2-dioxaborolan-2-yl)phenyl 1,1,2,2,3,3,4,4,4-nonafluorobutane-1-sulfonate (**7da**)

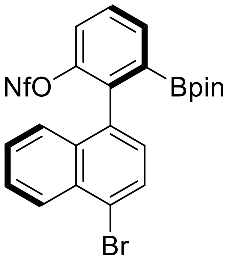

7da

Figure S8. *rac*-**7da**

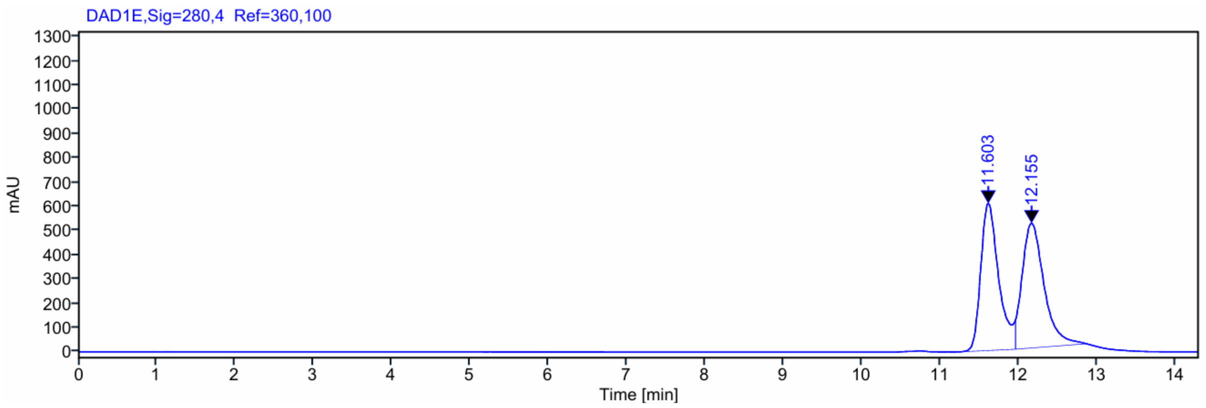

**Signal:**	DAD1E,Sig=280,4 Ref=360,100			
**RT [min]**	**Width [min]**	**Area**	**Height**	**Area%**
11.603	0.73	9702.74	606.57	49.24
12.155	0.90	10003.05	514.67	50.76
	**Sum**	**19705.80**		

Figure S9. (*R*)-**7da** (e.r. = 90:10)

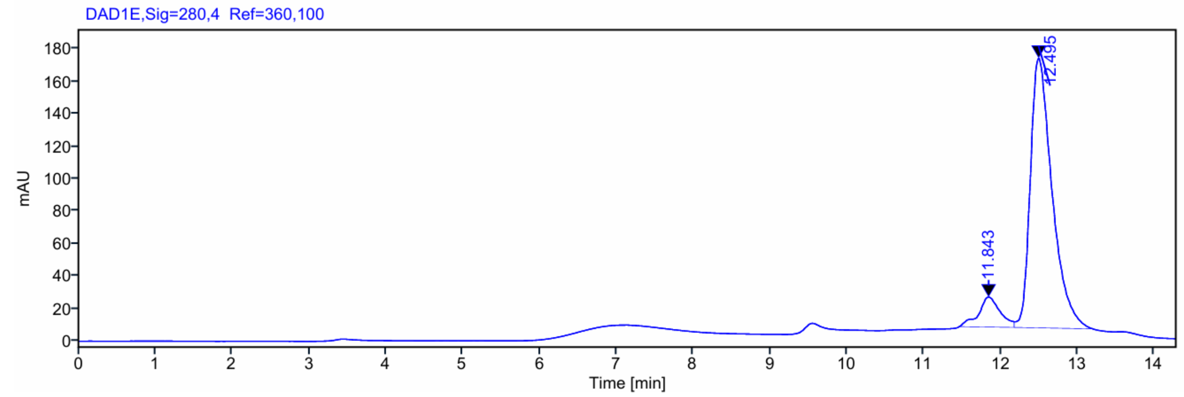

**Signal:**	DAD1E,Sig=280,4 Ref=360,100			
**RT [min]**	**Width [min]**	**Area**	**Height**	**Area%**
11.843	0.71	359.89	18.48	9.89
12.495	1.02	3279.03	166.10	90.11
	**Sum**	**3638.92**		

(*R*)-2-(4-cyanonaphthalen-1-yl)-3-(4,4,5,5-tetramethyl-1,3,2-dioxaborolan-2-yl)phenyl 1,1,2,2,3,3,4,4,4-nonafluorobutane-1-sulfonate (**7ea**)

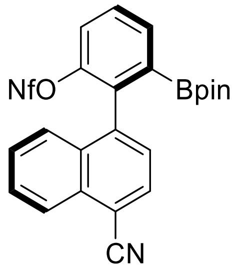

7ea

Figure S10. *rac*-**7ea**

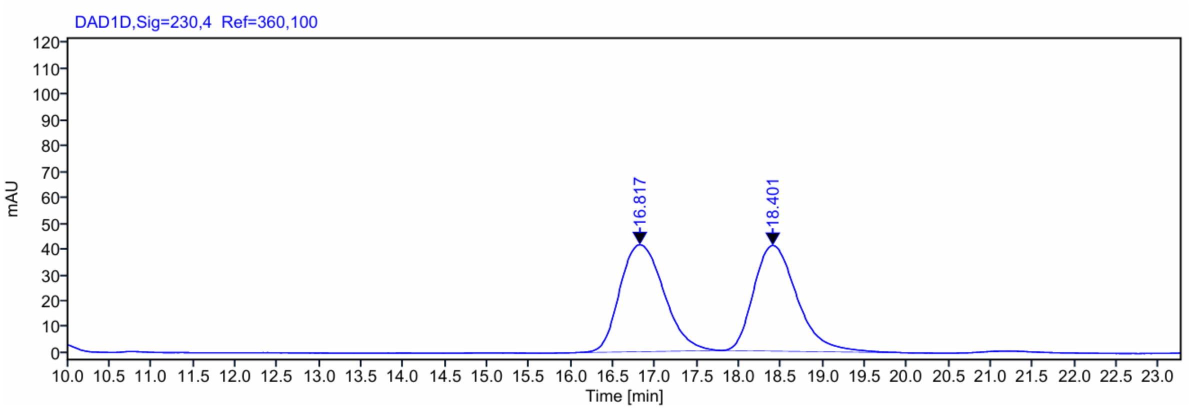

Signal:	DAD1D,Sig=230,4 Ref=360,100			
**RT [min]**	**Width [min]**	**Area**	**Height**	**Area%**
16.817	1.84	1505.42	41.33	50.68
18.401	2.30	1465.14	40.84	49.32
	**Sum**	**2970.56**		

Figure S11. (*R*)-**7ea** (e.r. = 99:1)

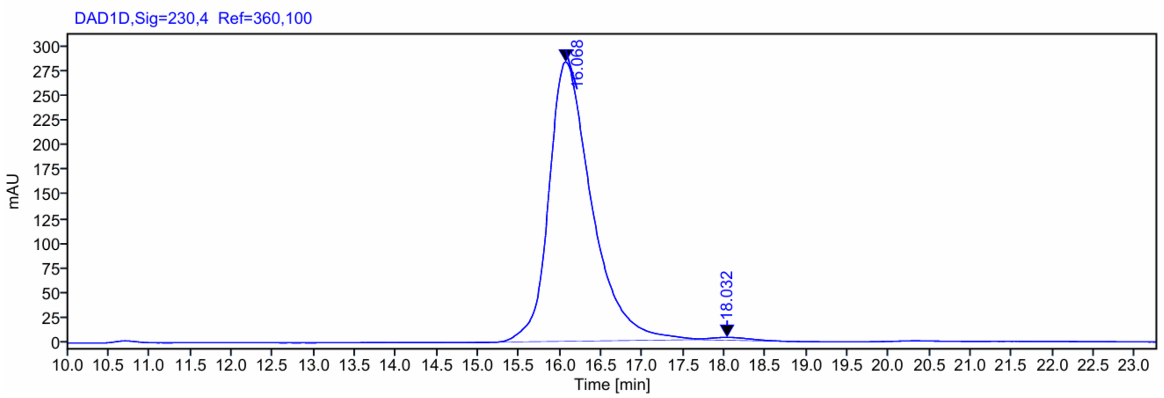

Signal:	DAD1D,Sig=230,4 Ref=360,100			
**RT [min]**	**Width [min]**	**Area**	**Height**	**Area%**
16.068	2.77	10285.48	283.05	99.02
18.032	1.16	101.78	2.88	0.98
	**Sum**	**10387.26**		

(*R*)-2-(5-phenylnaphthalen-1-yl)-3-(4,4,5,5-tetramethyl-1,3,2-dioxaborolan-2-yl)phenyl 1,1,2,2,3,3,4,4,4-nonafluorobutane-1-sulfonate (**7fa**)

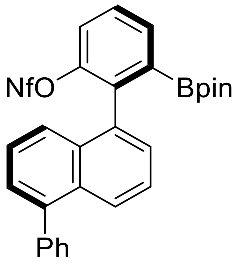

7fa

Figure S12. *rac*-**7fa**

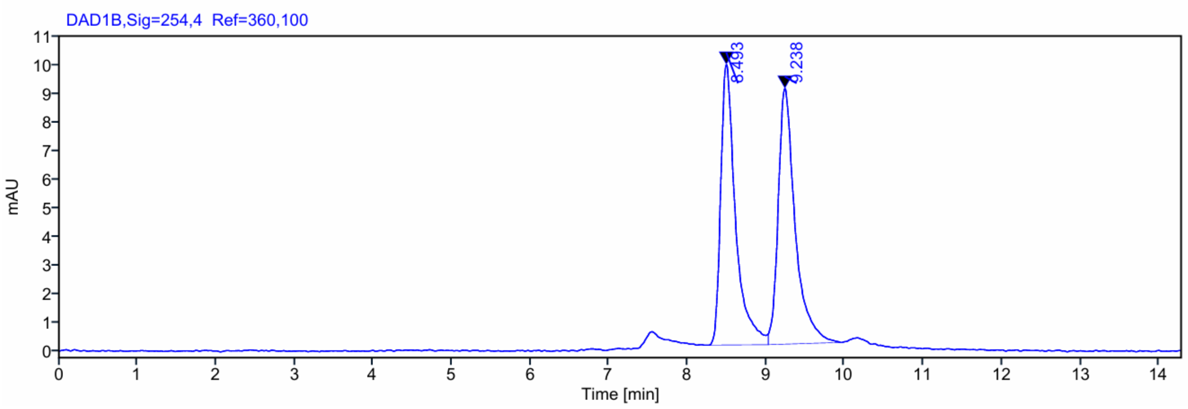

Signal:	DAD1B,Sig=254,4 Ref=360,100			
**RT [min]**	**Width [min]**	**Area**	**Height**	**Area%**
8.493	0.80	129.22	9.81	49.05
9.238	0.92	134.22	8.94	50.95
	**Sum**	**263.44**		

Figure S13. (*R*)-**7fa** (e.r. = 94:6)

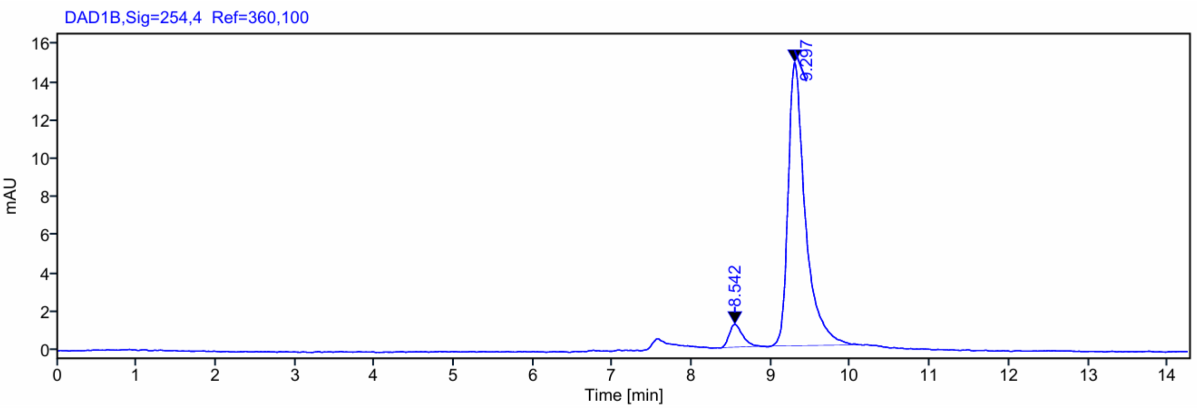

Signal:	DAD1B,Sig=254,4 Ref=360,100			
**RT [min]**	**Width [min]**	**Area**	**Height**	**Area%**
8.542	0.55	13.81	1.19	5.84
9.297	1.34	222.55	14.80	94.16
	**Sum**	**236.36**		

(*R*)-2-(5-((4-methylphenyl)sulfonamido)naphthalen-1-yl)-3-(4,4,5,5-tetramethyl-1,3,2-dioxaborolan-2-yl)phenyl 1,1,2,2,3,3,4,4,4-nonafluorobutane-1-sulfonate (7ga)

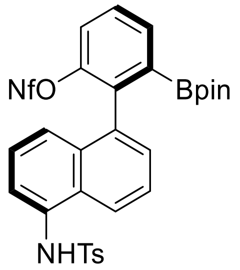

7ga

Figure S14. *rac*-**7ga**

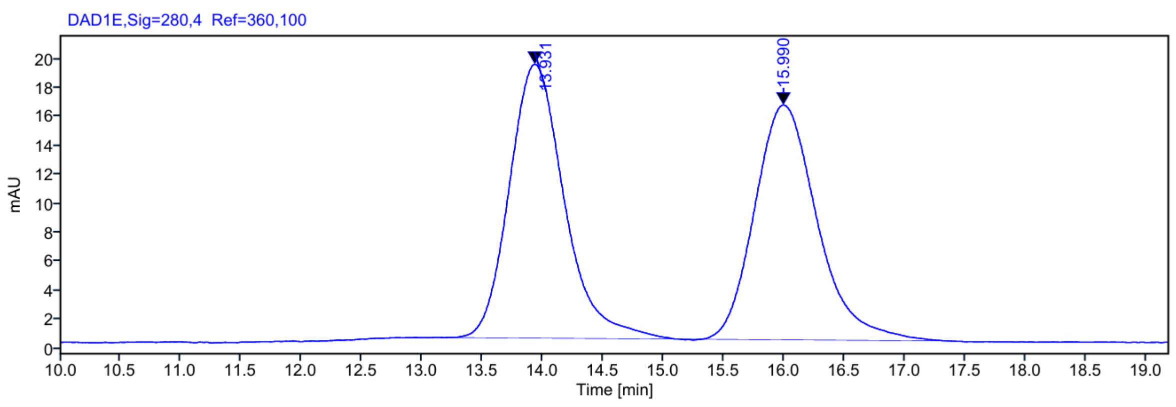

Signal: DAD1E,Sig=280,4 Ref=360,100

RT [min]	Width [min]	Area	Height	Area%
13.931	1.98	598.89	18.90	50.63
15.990	2.23	584.00	16.21	49.37
	**Sum**	**1182.89**		

Figure S15. (*R*)-**7ga** (e.r. = 94:6)

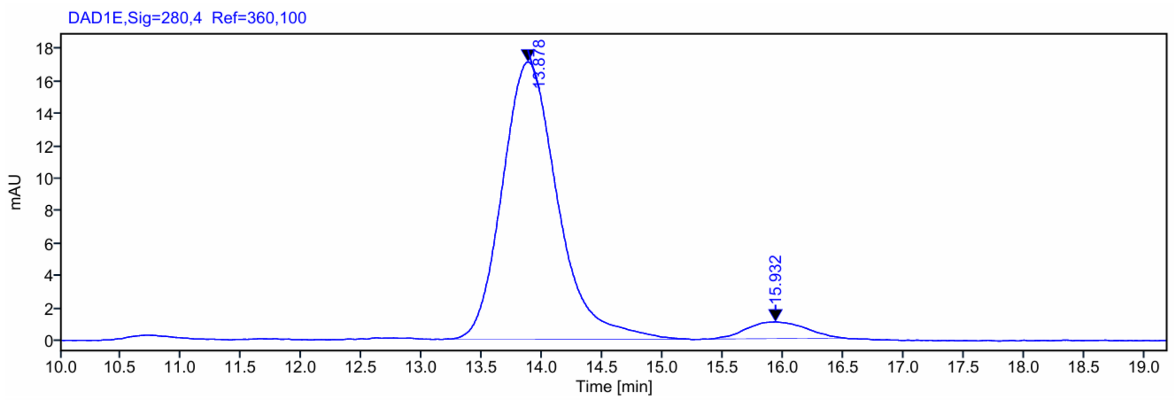

Signal: DAD1E,Sig=280,4 Ref=360,100

RT [min]	Width [min]	Area	Height	Area%
13.878	2.28	545.96	17.10	93.94
15.932	1.16	35.23	1.03	6.06
	**Sum**	**581.19**		

(*R*)-2-(2-methylnaphthalen-1-yl)-3-(4,4,5,5-tetramethyl-1,3,2-dioxaborolan-2-yl)phenyl 1,1,2,2,3,3,4,4,4-nonafluorobutane-1-sulfonate (**7ha**)

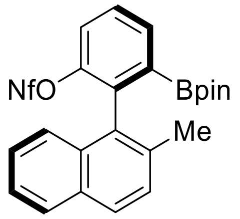

7ha

Figure S16. *rac*-**7ha**

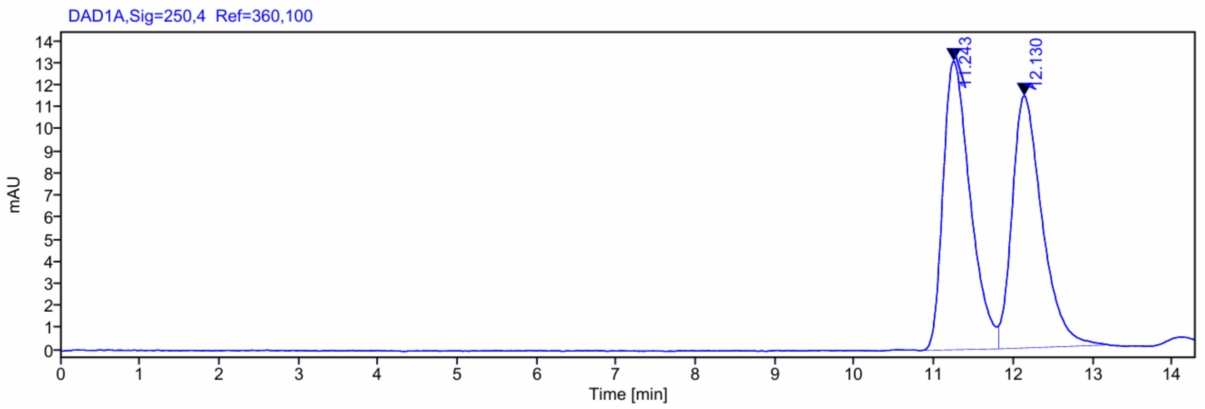

Signal:	DAD1A,Sig=250,4 Ref=360,100			
**RT [min]**	**Width [min]**	**Area**	**Height**	**Area%**
11.243	0.97	311.43	13.09	50.96
12.130	1.39	299.68	11.43	49.04
	**Sum**	**611.11**		

Figure S17. (*R*)-**7ha** (e.r. = 52:48)

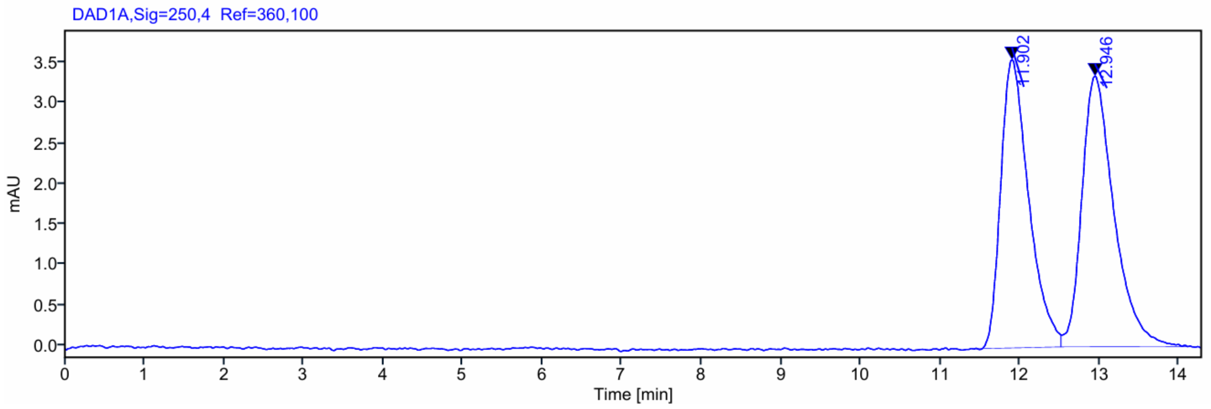

Signal:	DAD1A,Sig=250,4 Ref=360,100			
**RT [min]**	**Width [min]**	**Area**	**Height**	**Area%**
11.902	1.10	87.08	3.56	48.06
12.946	1.59	94.09	3.35	51.94
	**Sum**	**181.17**		

(*R*)-2-(8-methylnaphthalen-1-yl)-3-(4,4,5,5-tetramethyl-1,3,2-dioxaborolan-2-yl)phenyl 1,1,2,2,3,3,4,4,4-nonafluorobutane-1-sulfonate (**7ia**)

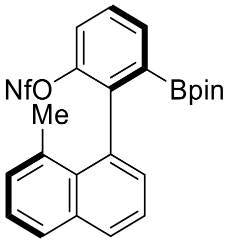

7ia

Figure S18. *rac*-**7ia**

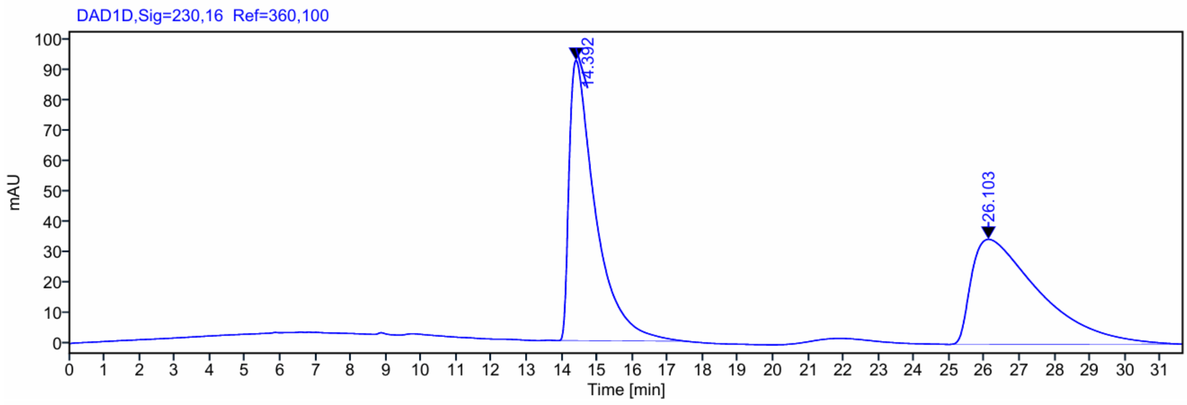

Signal: DAD1D,Sig=230,16 Ref=360,100

RT [min]	Width [min]	Area	Height	Area%
14.392	3.82	4763.51	91.97	50.77
26.103	6.52	4618.43	34.50	49.23
	**Sum**	**9381.94**		

Figure S19. (*R*)-**7ia** (e.r. = 99:1)

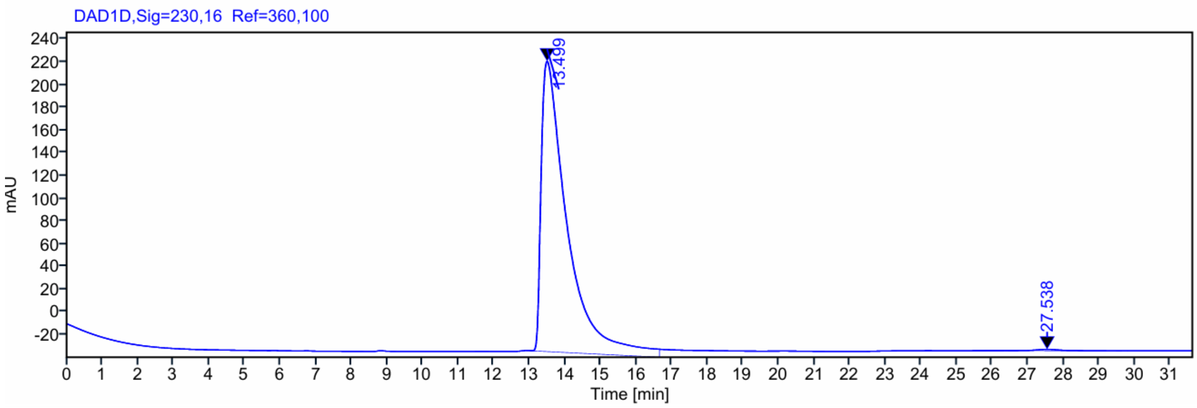

Signal: DAD1D,Sig=230,16 Ref=360,100

RT [min]	Width [min]	Area	Height	Area%
13.499	3.73	12925.88	255.75	99.58
27.538	2.69	54.40	1.15	0.42
	**Sum**	**12980.28**		

(*R*)-2-(1,2-dihydroacenaphthylen-5-yl)-3-(4,4,5,5-tetramethyl-1,3,2-dioxaborolan-2-yl)phenyl 1,1,2,2,3,3,4,4,4-nonafluorobutane-1-sulfonate (**7ja**)

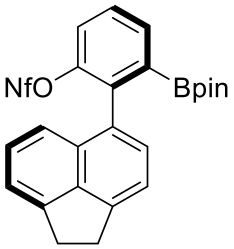

7ja

Figure S20. *rac*-**7ja**

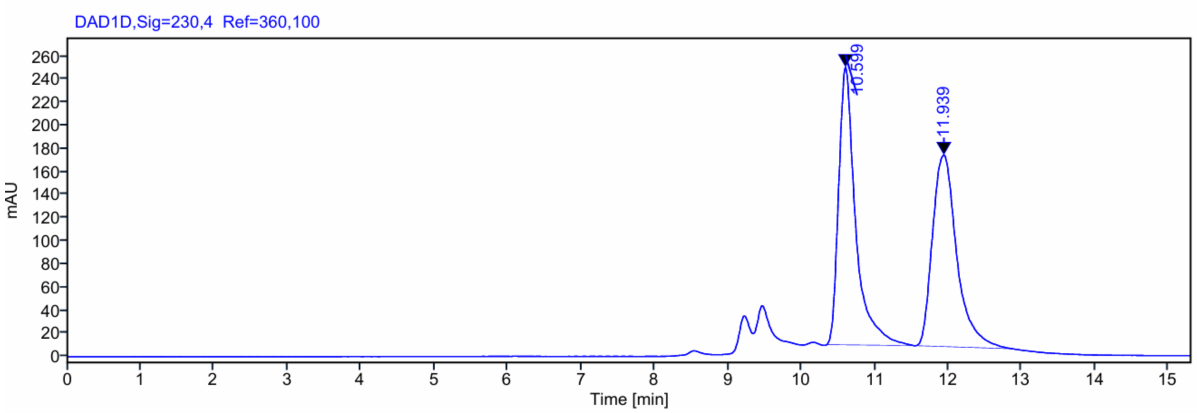

**Signal:**	DAD1D,Sig=230,4 Ref=360,100			
**RT [min]**	**Width [min]**	**Area**	**Height**	**Area%**
10.599	1.18	3863.00	240.28	50.28
11.939	1.35	3820.30	165.75	49.72
	**Sum**	**7683.30**		

Figure S21. (*R*)-**7ja** (e.r. = 97:3)

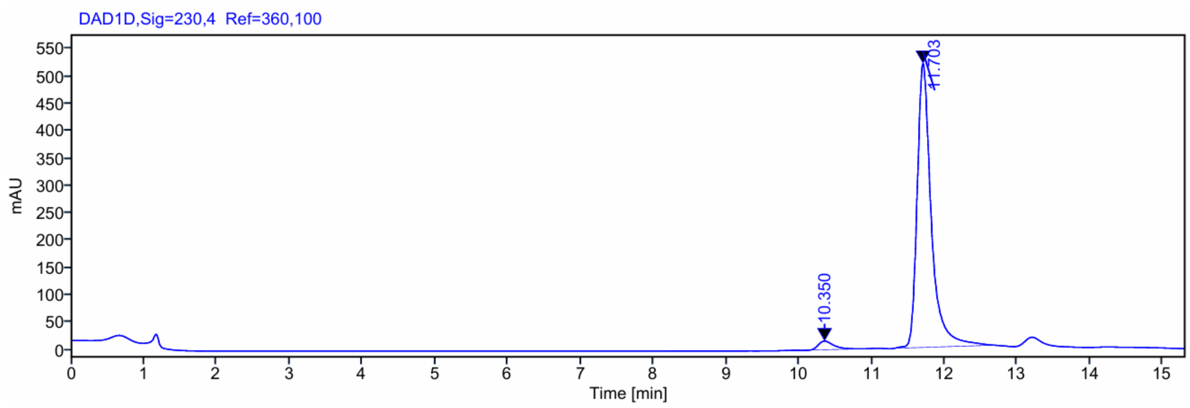

**Signal:**	DAD1D,Sig=230,4 Ref=360,100			
**RT [min]**	**Width [min]**	**Area**	**Height**	**Area%**
10.350	0.88	232.81	15.82	3.21
11.703	1.37	7013.56	517.99	96.79
	**Sum**	**7246.37**		

(*R*)-2-(fluoranthen-3-yl)-3-(4,4,5,5-tetramethyl-1,3,2-dioxaborolan-2-yl)phenyl 1,1,2,2,3,3,4,4,4-nonafluorobutane-1-sulfonate (7ka)

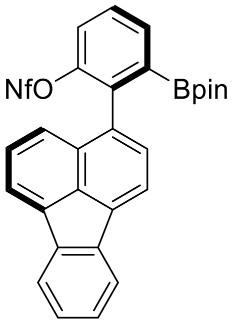

7ka

Figure S22. *rac*-**7ka**

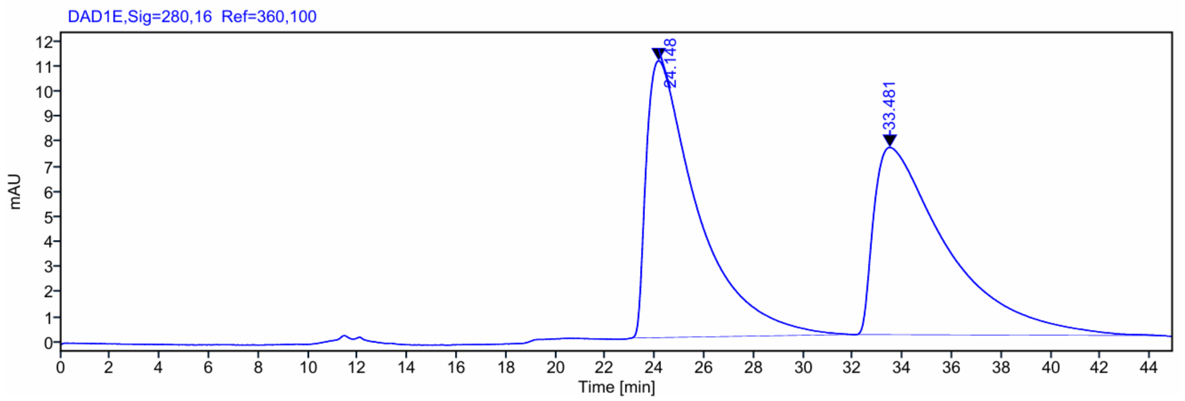

**Signal:**	DAD1E,Sig=280,16 Ref=360,100			
**RT [min]**	**Width [min]**	**Area**	**Height**	**Area%**
24.148	8.89	1589.41	11.02	50.91
33.481	12.51	1532.33	7.46	49.09
	**Sum**	**3121.74**		

Figure S23. (*R*)-**7ka** (e.r. = 96:4)

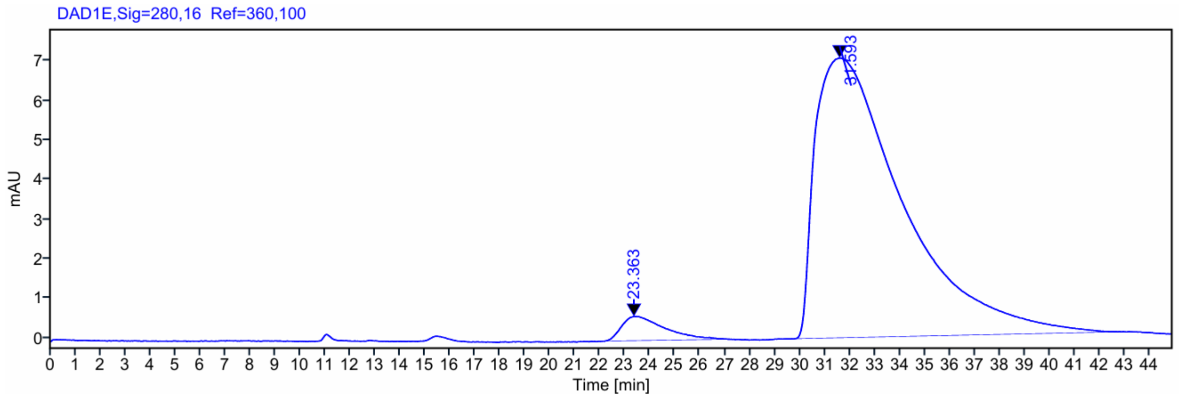

**Signal:**	DAD1E,Sig=280,16 Ref=360,100			
**RT [min]**	**Width [min]**	**Area**	**Height**	**Area%**
23.363	5.03	72.74	0.61	4.05
31.593	13.62	1724.40	7.07	95.95
	**Sum**	**1797.14**		

(*R*)-6-(4,4,5,5-tetramethyl-1,3,2-dioxaborolan-2-yl)-[1,1':2',1''-terphenyl]-2-yl 1,1,2,2,3,3,4,4,4-nonafluorobutane-1-sulfonate (**7la**)

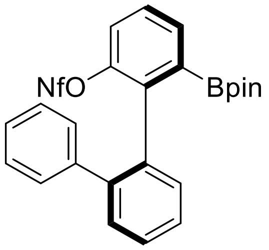

7la

Figure S24. *rac*-**7la**

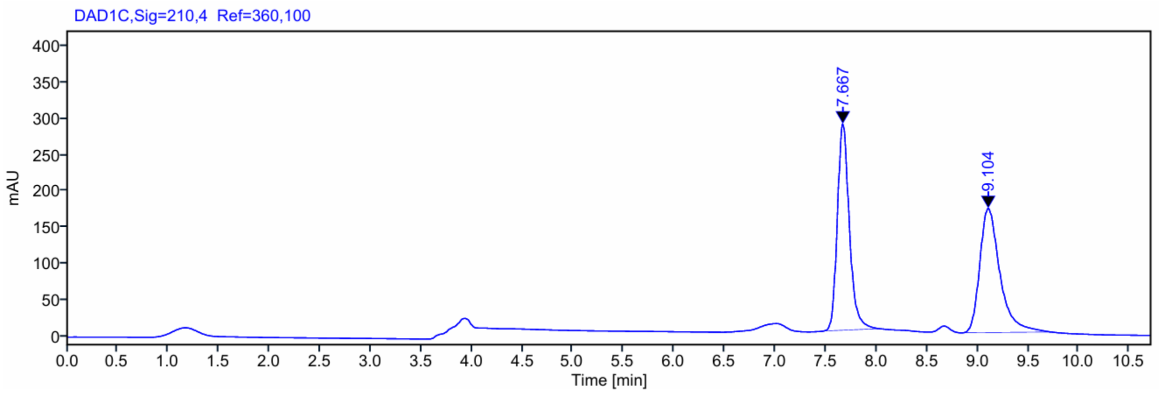

Signal: DAD1C,Sig=210,4 Ref=360,100

RT [min]	Width [min]	Area	Height	Area%
7.667	0.55	2350.78	284.95	49.71
9.104	0.90	2377.89	171.55	50.29
	**Sum**	**4728.67**		

Figure S25. (*R*)-**7la** (e.r. = 98:2)

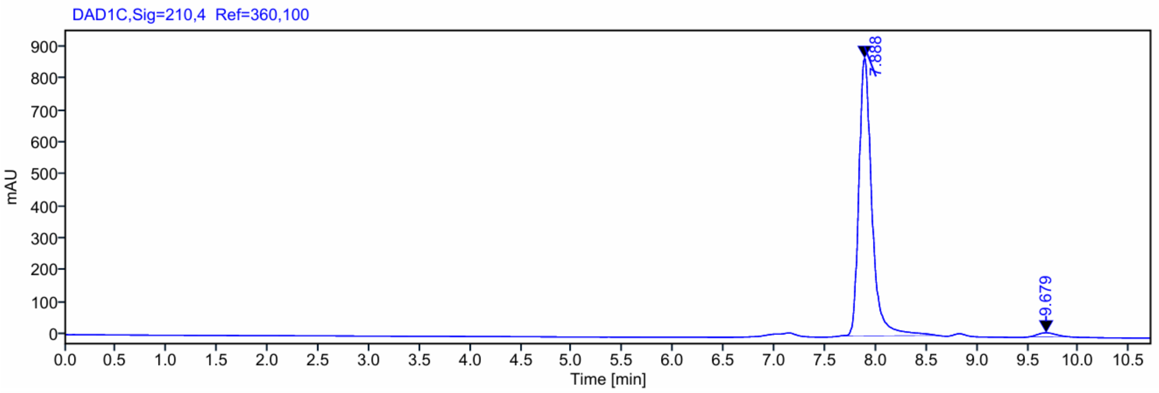

Signal: DAD1C,Sig=210,4 Ref=360,100

RT [min]	Width [min]	Area	Height	Area%
7.888	0.97	7764.67	868.67	97.96
9.679	0.41	161.76	12.86	2.04
	**Sum**	**7926.42**		

(*R*)-2''-fluoro-6-(4,4,5,5-tetramethyl-1,3,2-dioxaborolan-2-yl)-[1,1':2',1''-terphenyl]-2-yl 1,1,2,2,3,3,4,4,4-nonafluorobutane-1-sulfonate (**7ma**)

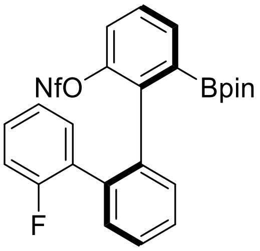

7ma

Figure S26. *rac*-**7ma**

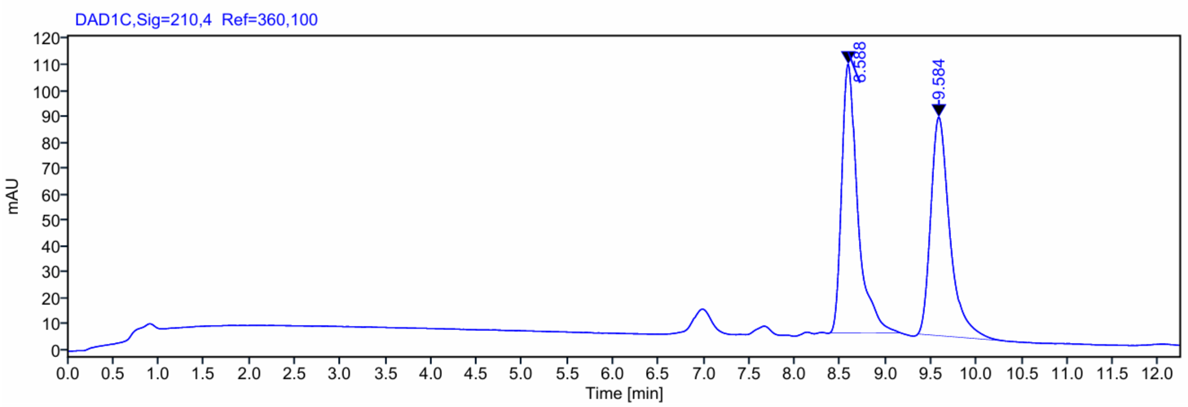

Signal: DAD1C,Sig=210,4 Ref=360,100

RT [min]	Width [min]	Area	Height	Area%
8.588	0.90	1265.00	103.56	50.47
9.584	0.99	1241.27	84.22	49.53
	**Sum**	**2506.27**		

Figure S27. (*R*)-**7ma** (e.r. = 90:10)

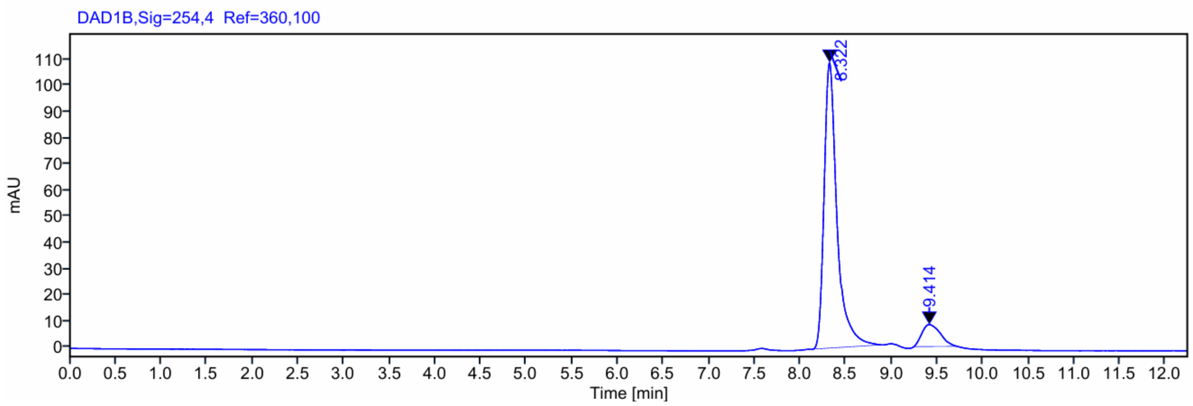

Signal: DAD1B,Sig=254,4 Ref=360,100

RT [min]	Width [min]	Area	Height	Area%
8.322	0.82	1073.00	109.00	90.06
9.414	0.47	118.38	8.37	9.94
	**Sum**	**1191.38**		

(*R*)-3''-chloro-6-(4,4,5,5-tetramethyl-1,3,2-dioxaborolan-2-yl)-[1,1':2',1''-terphenyl]-2-yl 1,1,2,2,3,3,4,4,4-nonafluorobutane-1-sulfonate (**7na**)

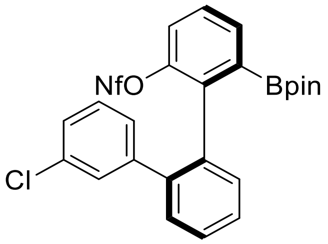

7na

Figure S28. *rac*-**7na**

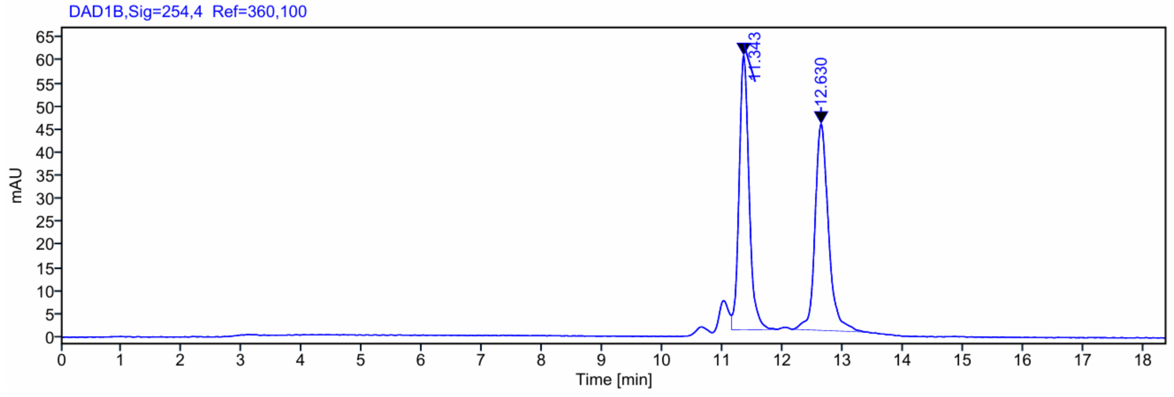

Signal: DAD1B,Sig=254,4 Ref=360,100

RT [min]	Width [min]	Area	Height	Area%
11.343	0.75	686.00	59.50	49.62
12.630	1.25	696.47	44.76	50.38
	**Sum**	**1382.47**		

Figure S29. (*R*)-**7na** (e.r. = 99:1)

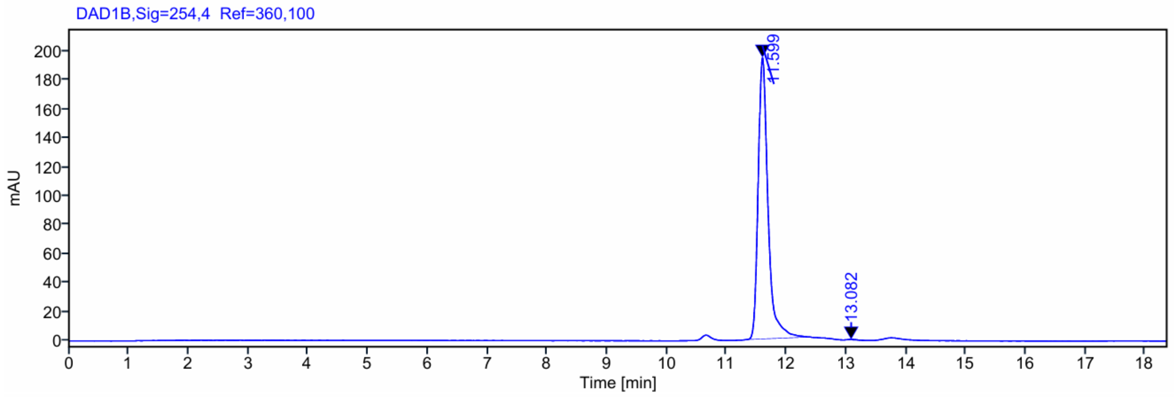

Signal: DAD1B,Sig=254,4 Ref=360,100

RT [min]	Width [min]	Area	Height	Area%
11.599	1.06	2218.60	194.24	99.76
13.082	0.41	5.28	0.51	0.24
	**Sum**	**2223.89**		

(*R*)-4''-(*tert*-butyl)-6-(4,4,5,5-tetramethyl-1,3,2-dioxaborolan-2-yl)-[1,1':2',1''-terphenyl]-2-yl 1,1,2,2,3,3,4,4,4-nonafluorobutane-1-sulfonate (**7oa**)

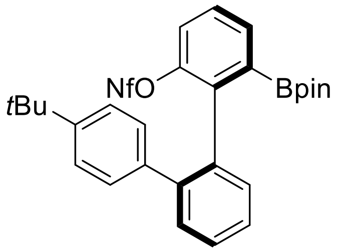

7oa

Figure S30. *rac*-**7oa**

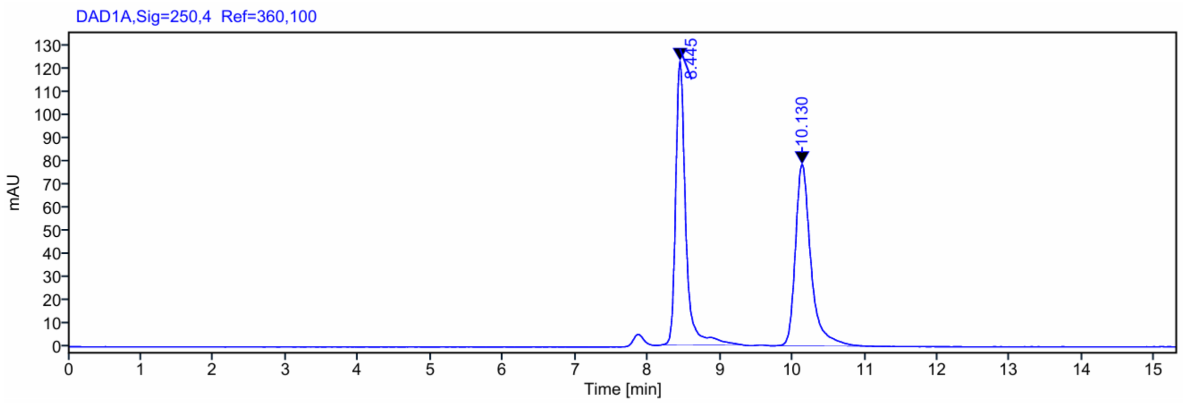

**Signal:**	DAD1A,Sig=250,4 Ref=360,100			
**RT [min]**	**Width [min]**	**Area**	**Height**	**Area%**
8.445	1.11	1186.97	122.96	50.40
10.130	1.30	1168.32	78.60	49.60
	**Sum**	**2355.29**		

Figure S31. (*R*)-**7oa** (e.r. = 95:5)

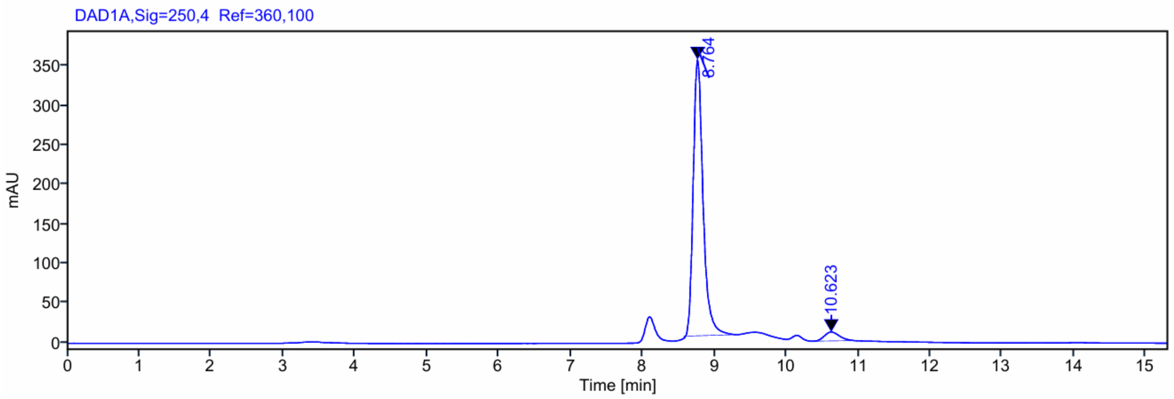

**Signal:**	DAD1A,Sig=250,4 Ref=360,100			
**RT [min]**	**Width [min]**	**Area**	**Height**	**Area%**
8.764	0.73	3327.04	349.82	95.32
10.623	0.58	163.28	11.28	4.68
	**Sum**	**3490.32**		

(*R*)-4''-acetyl-6-(4,4,5,5-tetramethyl-1,3,2-dioxaborolan-2-yl)-[1,1':2',1''-terphenyl]-2-yl 1,1,2,2,3,3,4,4,4-nonafluorobutane-1-sulfonate (**7pa**)

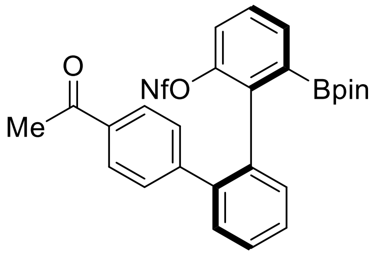

7pa

Figure S32. *rac*-**7pa**

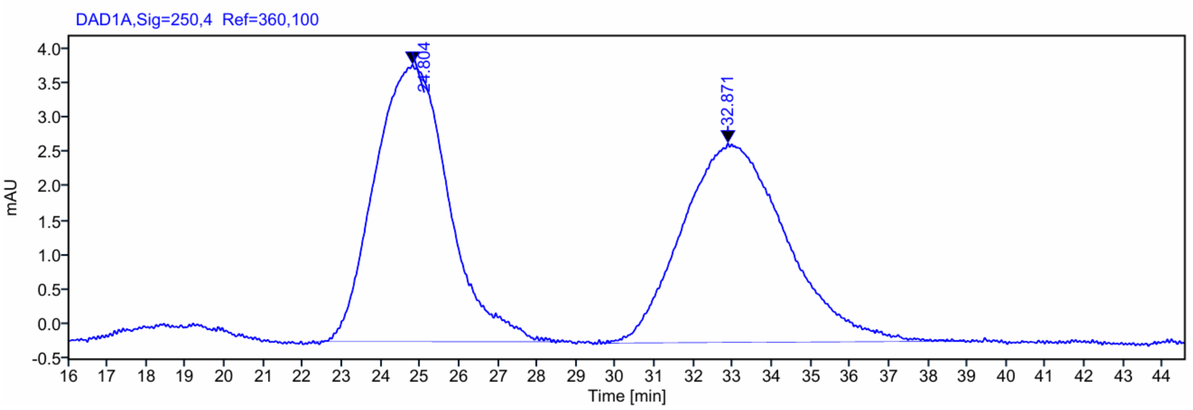

Signal: DAD1A,Sig=250,4 Ref=360,100

RT [min]	Width [min]	Area	Height	Area%
24.804	6.51	525.81	4.01	49.71
32.871	9.43	531.98	2.88	50.29
	**Sum**	**1057.79**		

Figure S33. (*R*)-**7pa** (e.r. = 99:1)

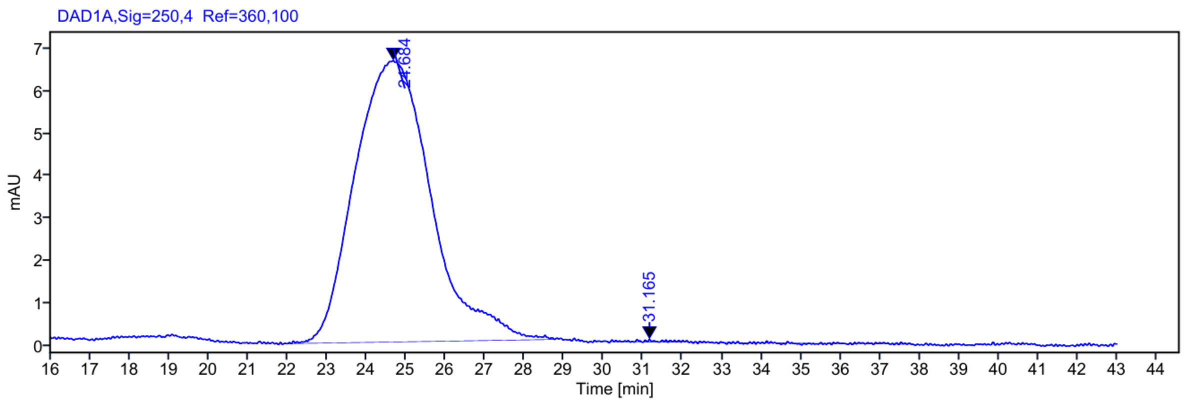

Signal: DAD1A,Sig=250,4 Ref=360,100

RT [min]	Width [min]	Area	Height	Area%
24.684	6.86	885.13	6.60	99.69
31.165	7.90	2.75	0.06	0.31
	**Sum**	**887.89**		

(*R*)-4''-nitro-6-(4,4,5,5-tetramethyl-1,3,2-dioxaborolan-2-yl)-[1,1':2',1''-terphenyl]-2-yl 1,1,2,2,3,3,4,4,4-nonafluorobutane-1-sulfonate (**7qa**)

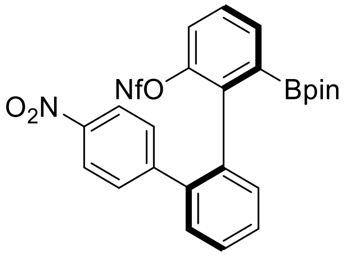

7qa

Figure S34. *rac*-**7qa**

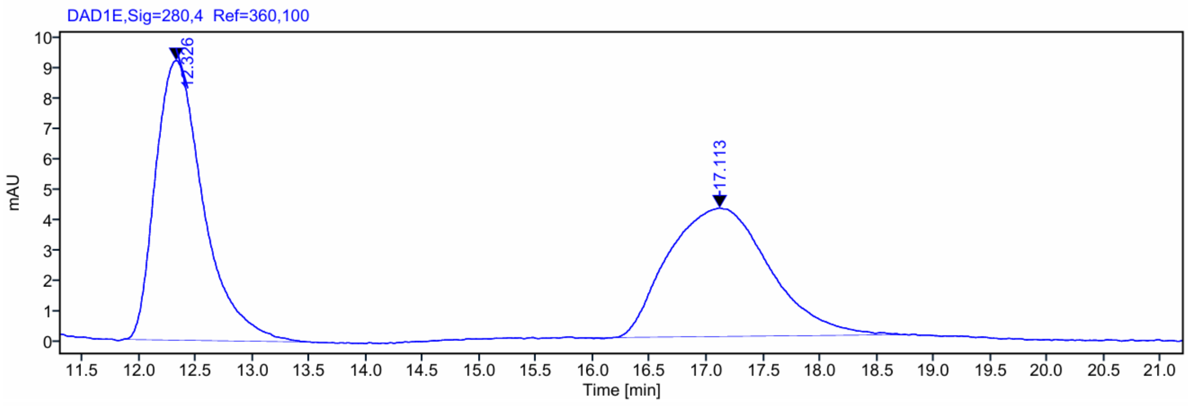

Signal: DAD1E,Sig=280,4 Ref=360,100

RT [min]	Width [min]	Area	Height	Area%
12.326	1.64	269.71	9.20	50.69
17.113	2.86	262.37	4.23	49.31
	**Sum**	**532.09**		

Figure S35. (*R*)-**7qa** (e.r. = 90:10)

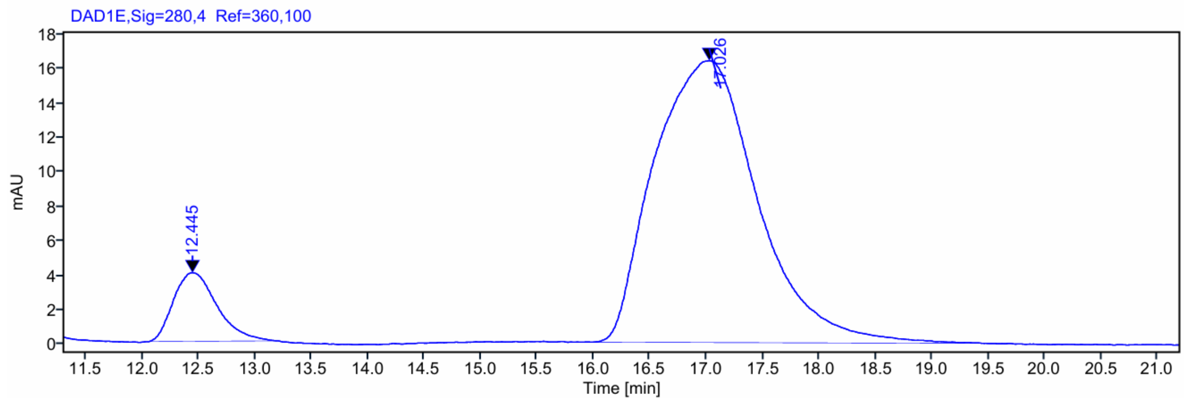

Signal: DAD1E,Sig=280,4 Ref=360,100

RT [min]	Width [min]	Area	Height	Area%
12.445	1.15	106.73	3.98	9.26
17.026	3.77	1046.40	16.36	90.74
	**Sum**	**1153.13**		

(*R*)-4'-chloro-6-(4,4,5,5-tetramethyl-1,3,2-dioxaborolan-2-yl)-[1,1':2',1''-terphenyl]-2-yl 1,1,2,2,3,3,4,4,4-nonafluorobutane-1-sulfonate (**7ra**)

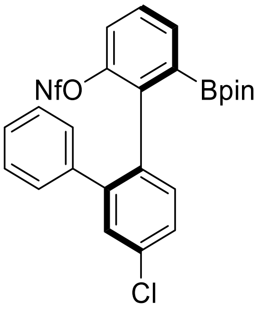

7ra

Figure S36. *rac*-**7ra**

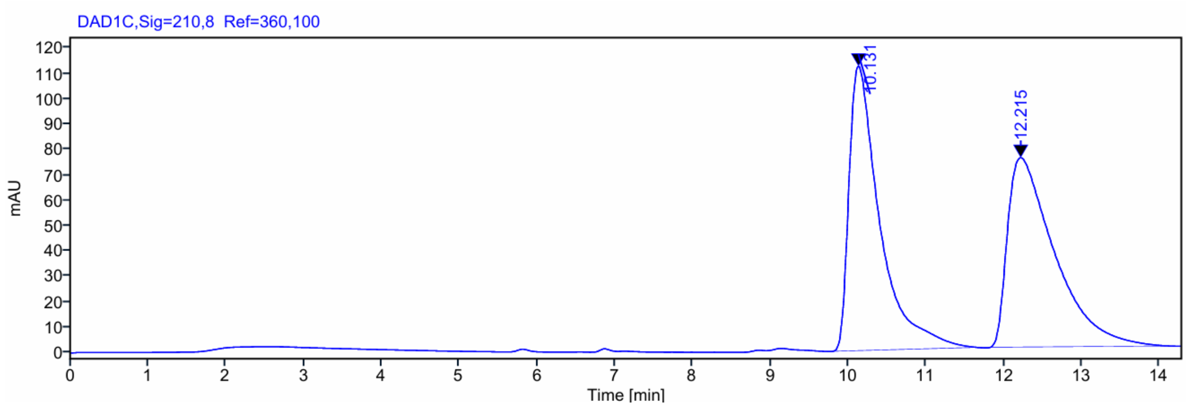

Signal:	DAD1C,Sig=210,8 Ref=360,100			
**RT [min]**	**Width [min]**	**Area**	**Height**	**Area%**
10.131	1.84	3087.34	112.17	49.57
12.215	2.47	3140.65	74.72	50.43
	**Sum**	**6227.99**		

Figure S37. (*R*)-**7ra** (e.r. = 91:9)

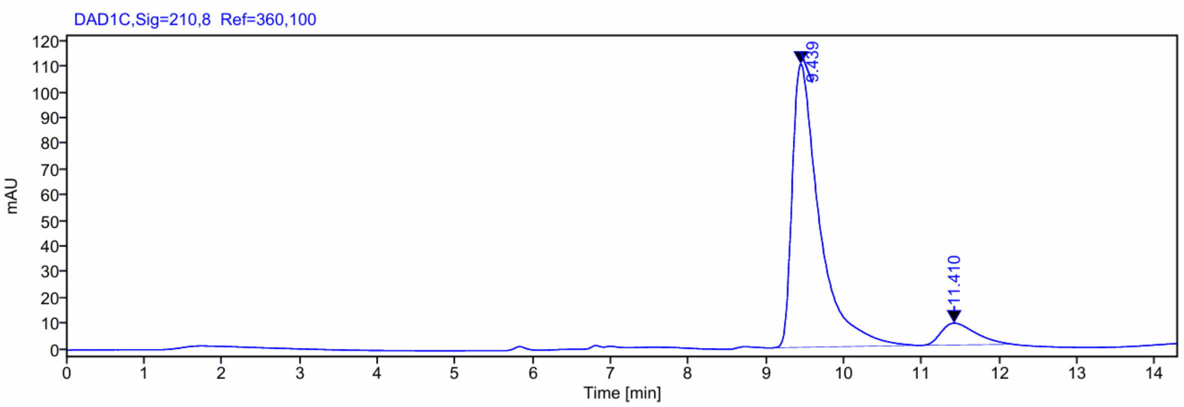

Signal:	DAD1C,Sig=210,8 Ref=360,100			
**RT [min]**	**Width [min]**	**Area**	**Height**	**Area%**
9.439	1.93	2667.98	110.25	91.03
11.410	1.23	263.04	8.51	8.97
	**Sum**	**2931.03**		

(*R*)-5'-methyl-6-(4,4,5,5-tetramethyl-1,3,2-dioxaborolan-2-yl)-[1,1':2',1''-terphenyl]-2-yl 1,1,2,2,3,3,4,4,4-nonafluorobutane-1-sulfonate (**7sa**)

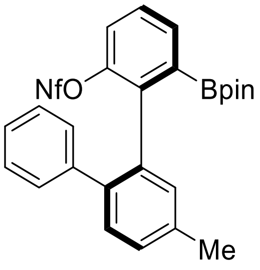

7sa

Figure S38. *rac*-**7sa**

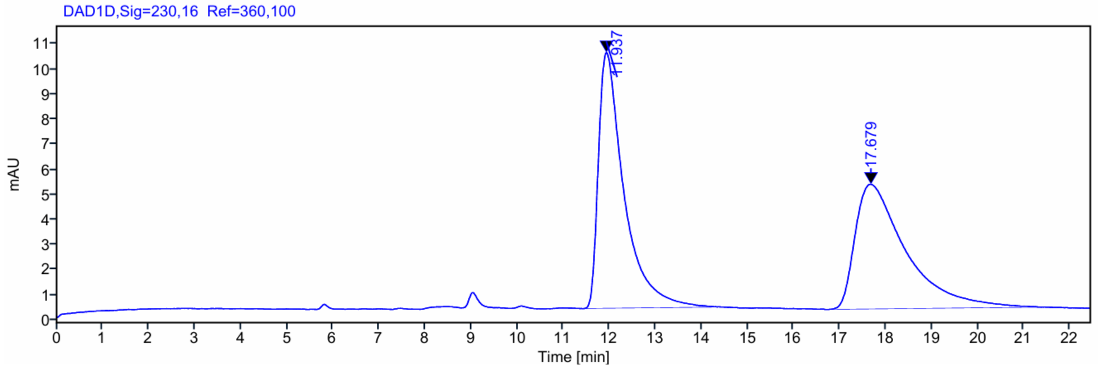

Signal: DAD1D,Sig=230,16 Ref=360,100

RT [min]	Width [min]	Area	Height	Area%
11.937	3.11	393.97	10.20	50.83
17.679	4.64	381.04	4.97	49.17
	**Sum**	**775.02**		

Figure S39. (*R*)-**7sa** (e.r. = 54:46)

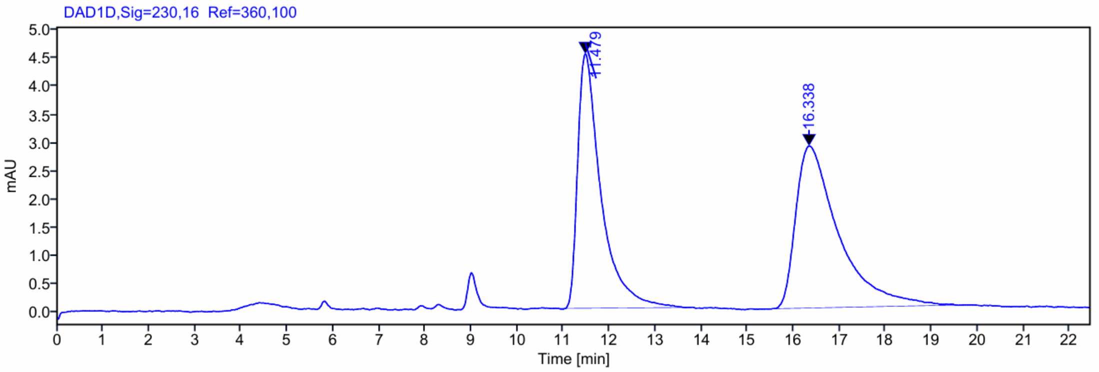

Signal: DAD1D,Sig=230,16 Ref=360,100

RT [min]	Width [min]	Area	Height	Area%
11.479	2.88	158.72	4.52	46.07
16.338	4.30	185.78	2.88	53.93
	**Sum**	**344.50**		

(*R*)-2'-(naphthalen-2-yl)-6-(4,4,5,5-tetramethyl-1,3,2-dioxaborolan-2-yl)-[1,1'-biphenyl]-2-yl 1,1,2,2,3,3,4,4,4-nonafluorobutane-1-sulfonate (**7ta**)

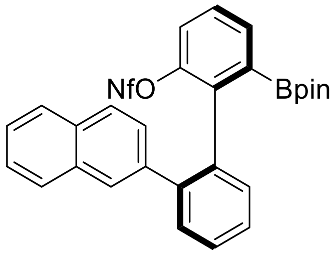

7ta

Figure S40. *rac*-**7ta**

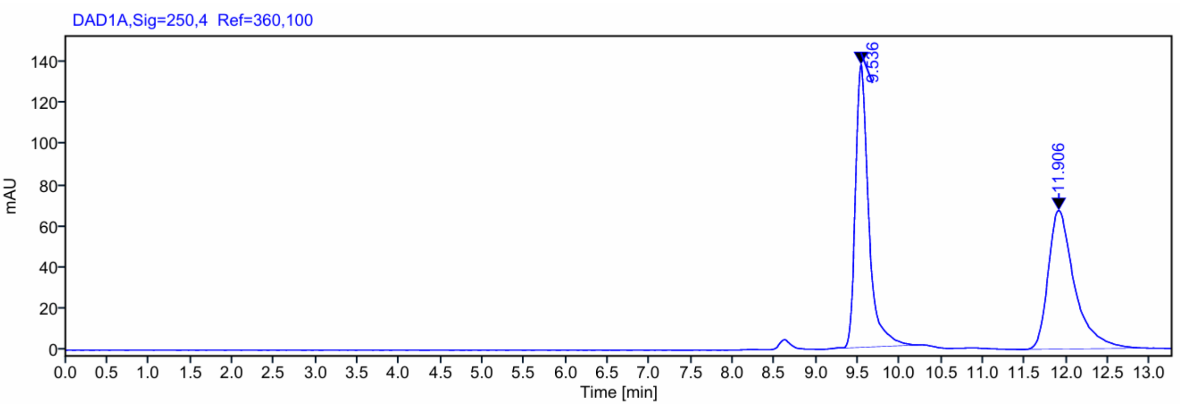

**Signal:**	DAD1A,Sig=250,4 Ref=360,100			
**RT [min]**	**Width [min]**	**Area**	**Height**	**Area%**
9.536	1.07	1516.87	137.37	50.70
11.906	1.52	1474.79	67.32	49.30
	**Sum**	**2991.66**		

Figure S41. (*R*)-**7ta** (e.r. = 98:2)

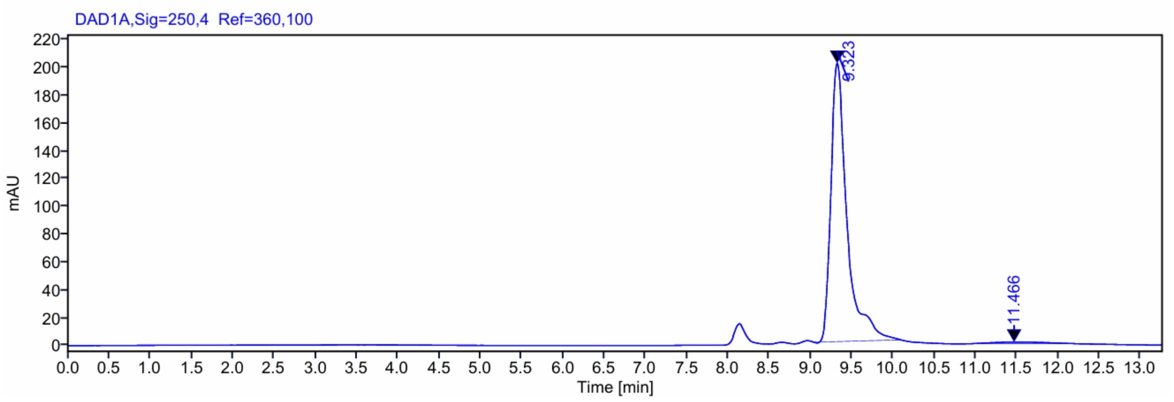

**Signal:**	DAD1A,Sig=250,4 Ref=360,100			
**RT [min]**	**Width [min]**	**Area**	**Height**	**Area%**
9.323	1.05	2724.49	200.37	97.94
11.466	1.34	57.26	1.23	2.06
	**Sum**	**2781.74**		

(*R*)-6-(4,4,5,5-tetramethyl-1,3,2-dioxaborolan-2-yl)-2'-(thiophen-3-yl)-[1,1'-biphenyl]-2-yl 1,1,2,2,3,3,4,4,4-nonafluorobutane-1-sulfonate (**7ua**)

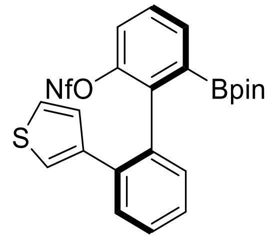

7ua

Figure S42. *rac*-**7ua**

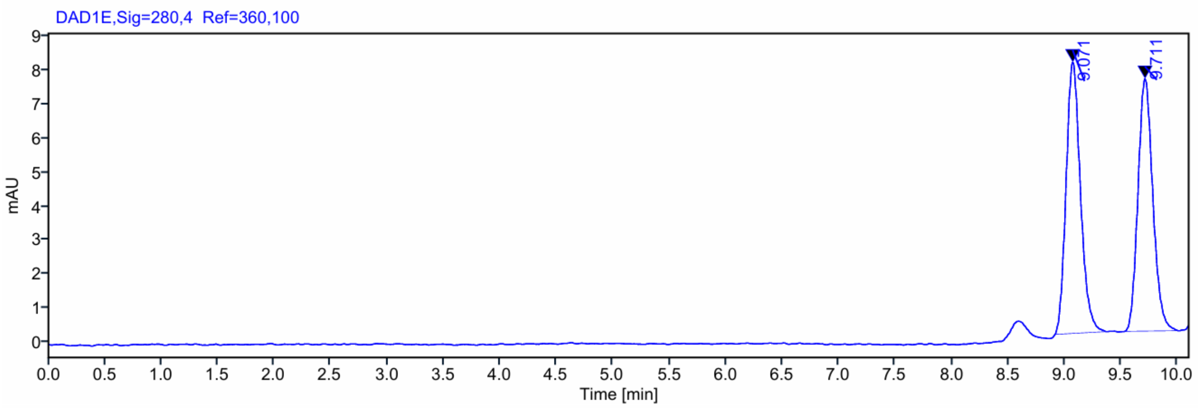

**Signal:**	DAD1E,Sig=280,4 Ref=360,100			
**RT [min]**	**Width [min]**	**Area**	**Height**	**Area%**
9.071	0.46	70.49	7.99	50.97
9.711	0.65	67.82	7.44	49.03
	**Sum**	**138.32**		

Figure S43. (*R*)-**7ua** (e.r. = 90:10)

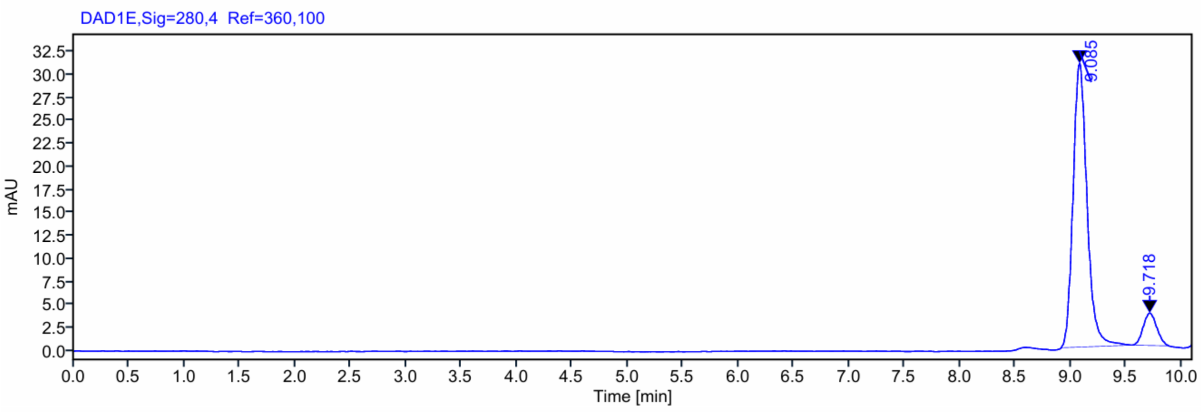

**Signal:**	DAD1E,Sig=280,4 Ref=360,100			
**RT [min]**	**Width [min]**	**Area**	**Height**	**Area%**
9.085	0.64	270.20	30.81	90.03
9.718	0.33	29.92	3.51	9.97
	**Sum**	**300.12**		

(*R*)-2'-cyano-6-(4,4,5,5-tetramethyl-1,3,2-dioxaborolan-2-yl)-[1,1'-biphenyl]-2-yl 1,1,2,2,3,3,4,4,4-nonafluorobutane-1-sulfonate (**7va**)

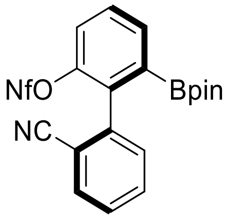

7va

Figure S44. *rac*-**7va**

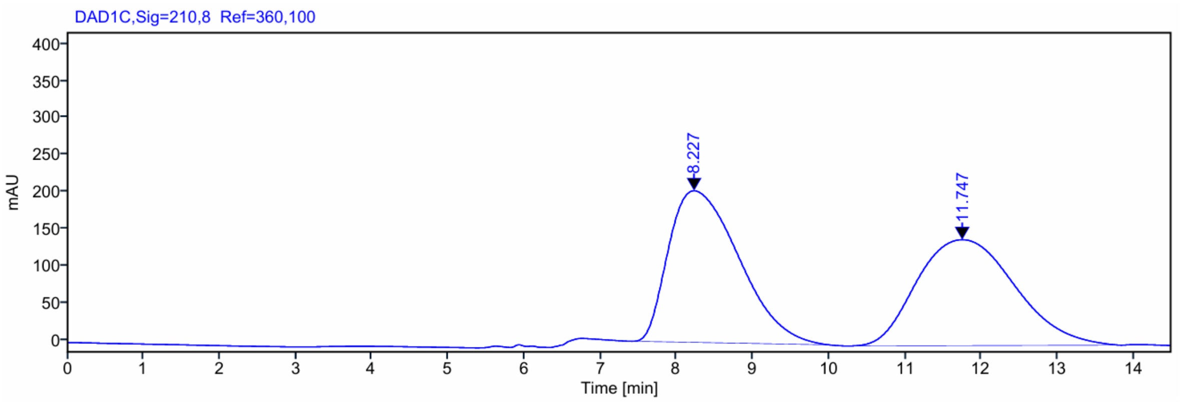

Signal: DAD1C,Sig=210,8 Ref=360,100

RT [min]	Width [min]	Area	Height	Area%
8.227	2.68	13128.60	204.12	50.67
11.747	4.03	12783.08	142.58	49.33
	**Sum**	**25911.68**		

Figure S45. (*R*)-**7va** (e.r. = 98:2)

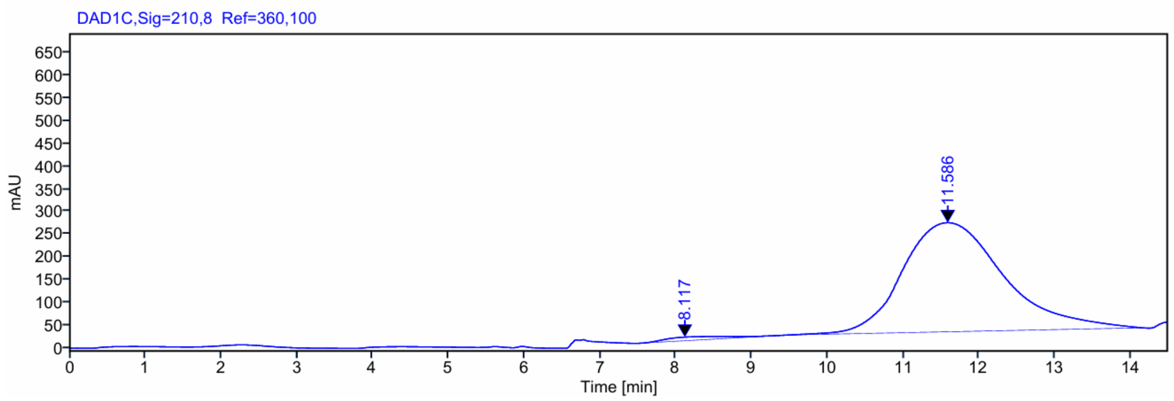

Signal: DAD1C,Sig=210,8 Ref=360,100

RT [min]	Width [min]	Area	Height	Area%
8.117	1.98	431.78	7.96	1.90
11.586	4.66	22286.68	240.95	98.10
	**Sum**	**22718.46**		

(*R*)-2'-isopropyl-6-(4,4,5,5-tetramethyl-1,3,2-dioxaborolan-2-yl)-[1,1'-biphenyl]-2-yl 1,1,2,2,3,3,4,4,4-nonafluorobutane-1-sulfonate (**7wa**)

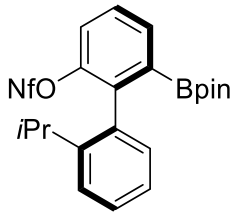

7wa

Figure S46. *rac*-**7wa**

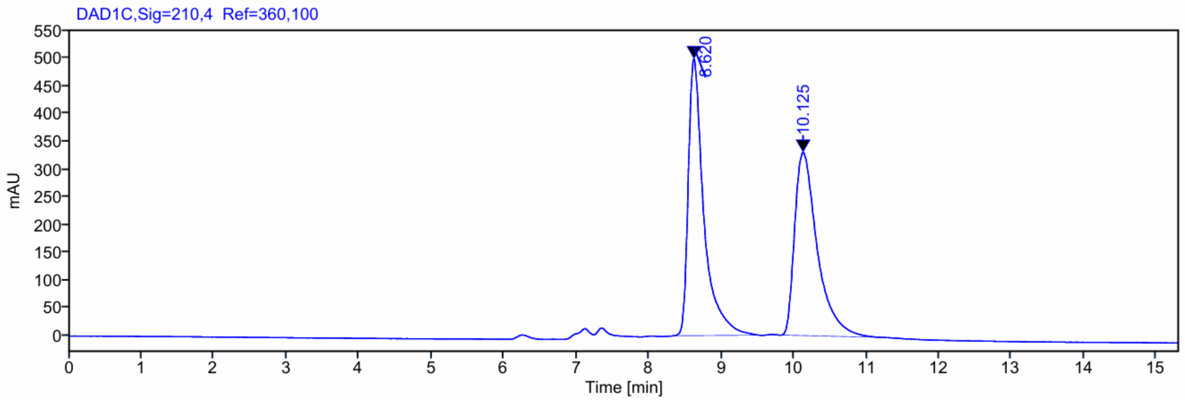

**Signal:**	DAD1C,Sig=210,4 Ref=360,100			
**RT [min]**	**Width [min]**	**Area**	**Height**	**Area%**
8.620	1.26	7469.10	499.59	51.01
10.125	1.42	7174.71	330.76	48.99
	**Sum**	**14643.82**		

Figure S47. (*R*)-**7wa** (e.r. = 98:2)

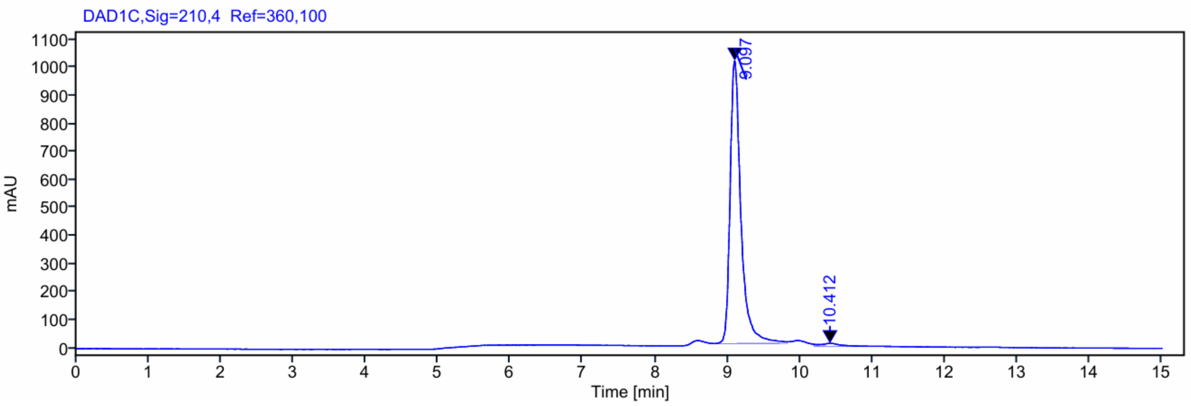

**Signal:**	DAD1C,Sig=210,4 Ref=360,100			
**RT [min]**	**Width [min]**	**Area**	**Height**	**Area%**
9.097	0.99	10459.29	1008.33	98.22
10.412	0.69	189.82	10.13	1.78
	**Sum**	**10649.12**		

(*R*)-2'-methyl-6-(4,4,5,5-tetramethyl-1,3,2-dioxaborolan-2-yl)-[1,1'-biphenyl]-2-yl 1,1,2,2,3,3,4,4,4-nonafluorobutane-1-sulfonate (**7xa**)

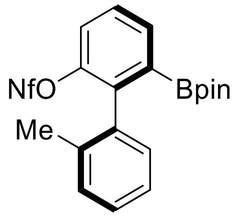

7xa

Figure S48. *rac*-**7xa**

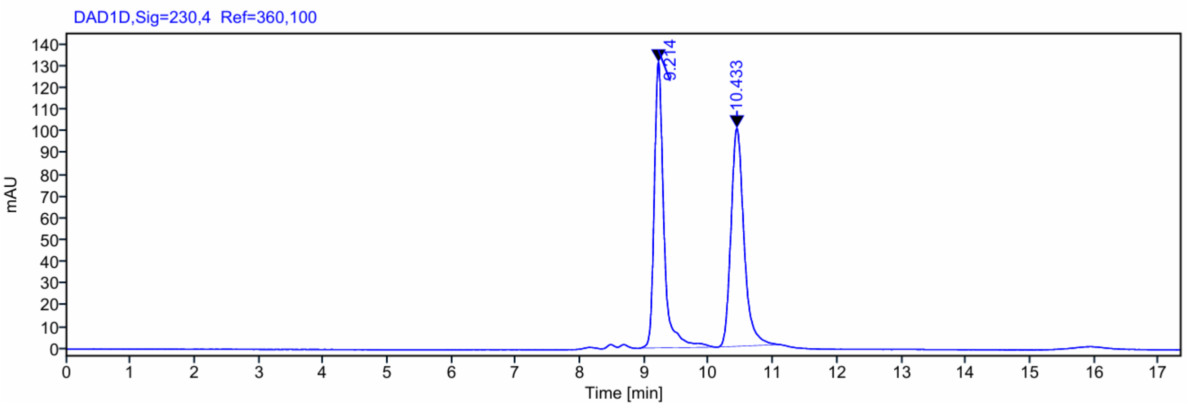

Signal: DAD1D,Sig=230,4 Ref=360,100

RT [min]	Width [min]	Area	Height	Area%
9.214	1.19	1373.28	131.32	49.33
10.433	0.98	1410.46	100.26	50.67
	**Sum**	**2783.75**		

Figure S49. (*R*)-**7xa** (e.r. = 74:26)

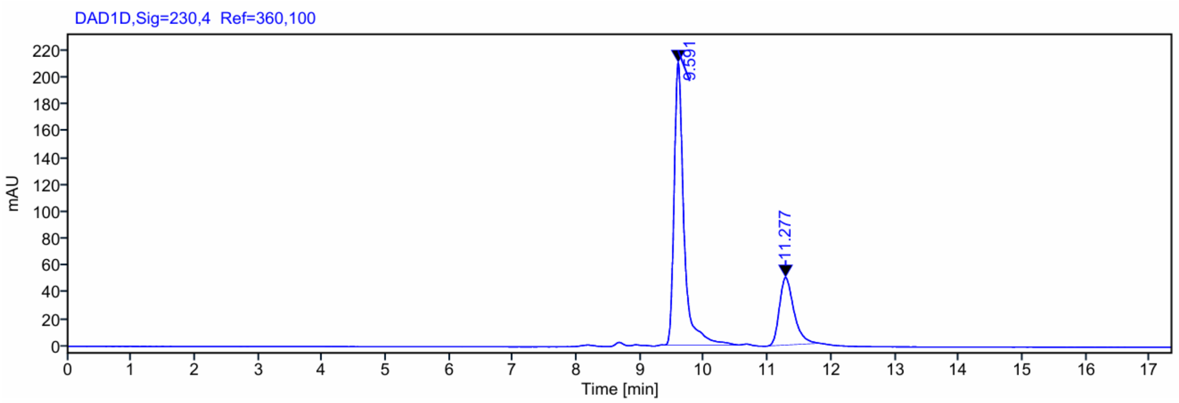

Signal: DAD1D,Sig=230,4 Ref=360,100

RT [min]	Width [min]	Area	Height	Area%
9.591	1.22	2257.28	210.11	73.93
11.277	0.89	796.10	50.29	26.07
	**Sum**	**3053.38**		

(*R*)-2'-chloro-6-(4,4,5,5-tetramethyl-1,3,2-dioxaborolan-2-yl)-[1,1'-biphenyl]-2-yl 1,1,2,2,3,3,4,4,4-nonafluorobutane-1-sulfonate (**7ya**)

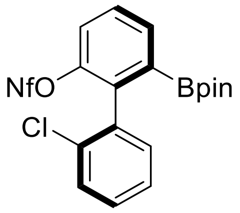

7ya

Figure S50. *rac*-**7ya**

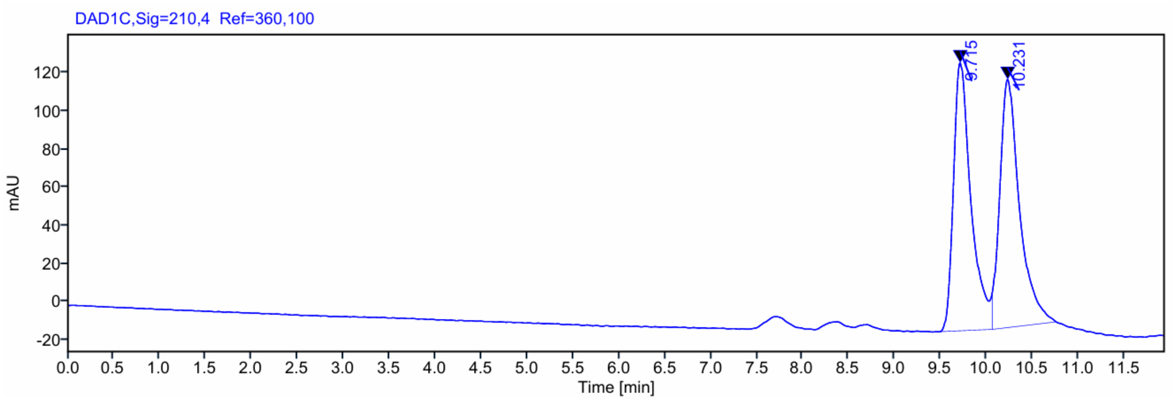

Signal: DAD1C,Sig=210,4 Ref=360,100

RT [min]	Width [min]	Area	Height	Area%
9.715	0.59	1871.70	140.25	49.01
10.231	0.70	1947.00	129.85	50.99
	**Sum**	**3818.70**		

Figure S51. (*R*)-**7ya** (e.r. = 88:12)

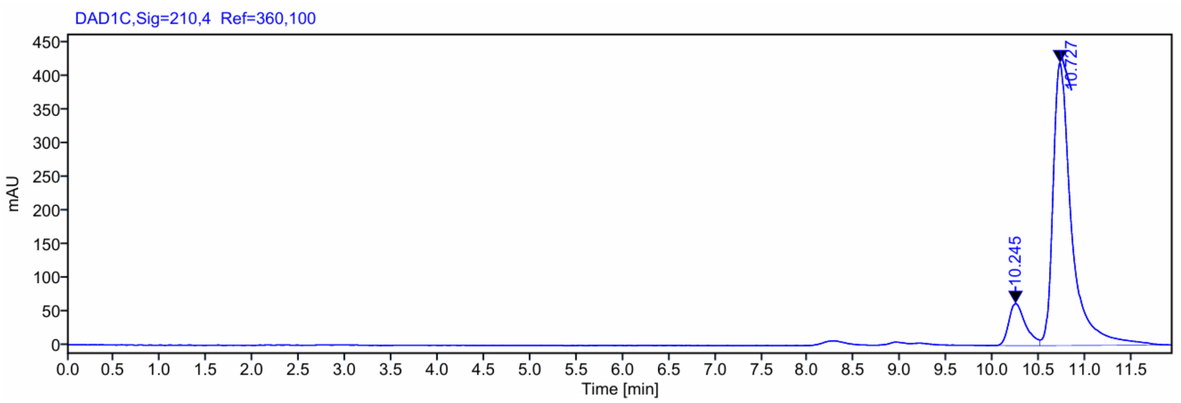

Signal: DAD1C,Sig=210,4 Ref=360,100

RT [min]	Width [min]	Area	Height	Area%
10.245	0.56	782.51	62.39	11.77
10.727	1.51	5868.62	419.90	88.23
	**Sum**	**6651.13**		

(*R*)-6-(5,5-dimethyl-1,3,2-dioxaborinan-2-yl)-[1,1':2',1''-terphenyl]-2-yl 1,1,2,2,3,3,4,4,4-nonafluorobutane-1-sulfonate (**7lb**)

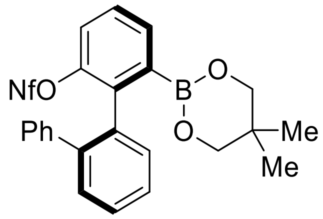

7lb

Figure S52. *rac*-**7lb**

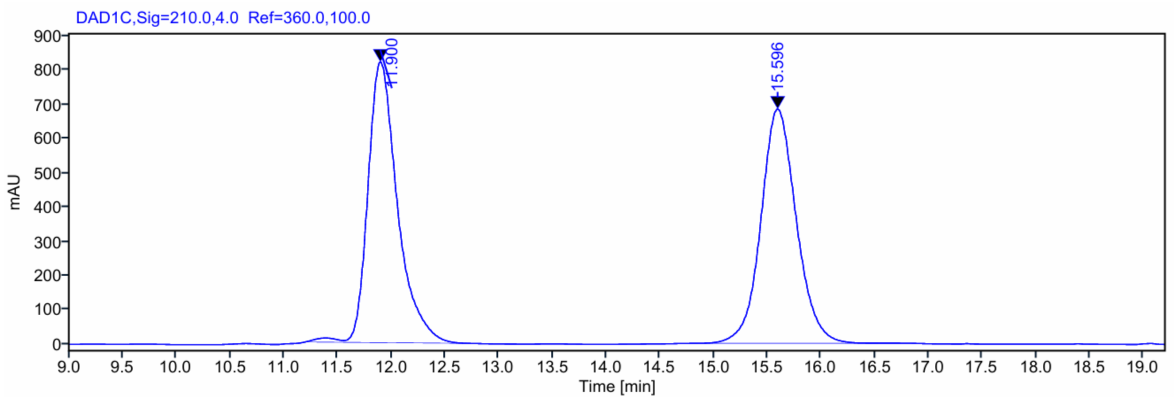

**Signal:**	DAD1C,Sig=210.0,4.0 Ref=360.0,100.0			
**RT [min]**	**Width [min]**	**Area**	**Height**	**Area%**
11.900	1.51	15734.99	818.89	50.07
15.596	1.69	15690.27	683.63	49.93
	**Sum**	**31425.26**		

Figure S53. (*R*)-**7lb** (e.r. = 95:5)

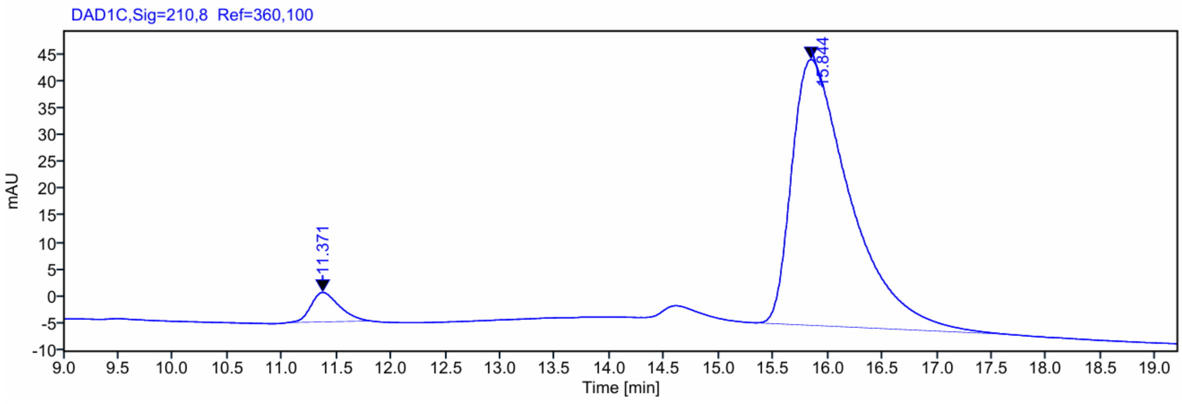

**Signal:**	DAD1C,Sig=210,8 Ref=360,100			
**RT [min]**	**Width [min]**	**Area**	**Height**	**Area%**
11.371	0.77	98.88	5.49	5.08
15.844	2.35	1846.59	49.45	94.92
	**Sum**	**1945.47**		

(*R*)-6-(4,4,6,6-tetramethyl-1,3,2-dioxaborinan-2-yl)-[1,1':2',1''-terphenyl]-2-yl 1,1,2,2,3,3,4,4,4-nonafluorobutane-1-sulfonate (**7lc**)

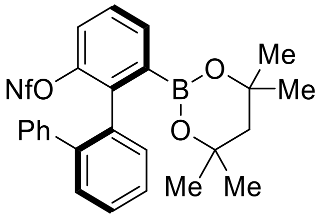

7lc

Figure S54. *rac*-**7lc**

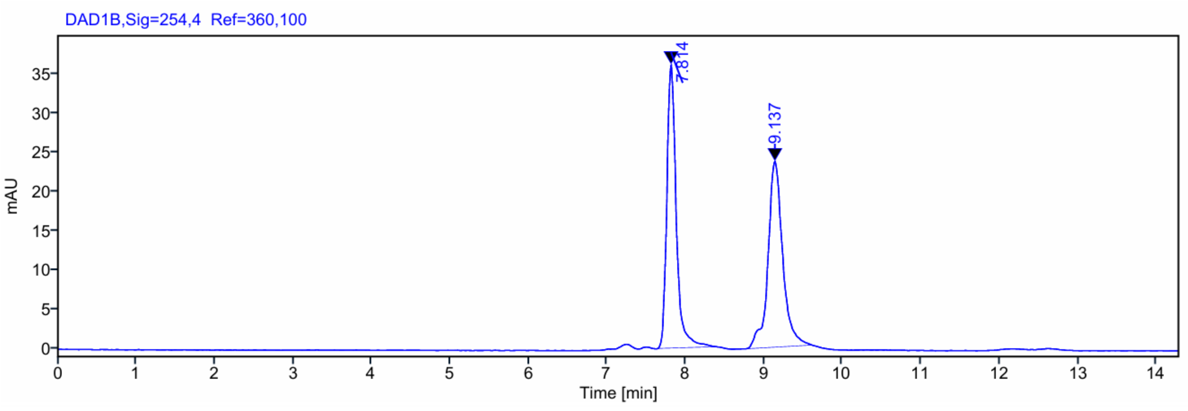

**Signal:**	DAD1B,Sig=254,4 Ref=360,100			
**RT [min]**	**Width [min]**	**Area**	**Height**	**Area%**
7.814	0.80	299.16	36.01	49.47
9.137	0.84	305.52	23.62	50.53
	**Sum**	**604.68**		

Figure S55. (*R*)-**7lc** (e.r. = 88:12)

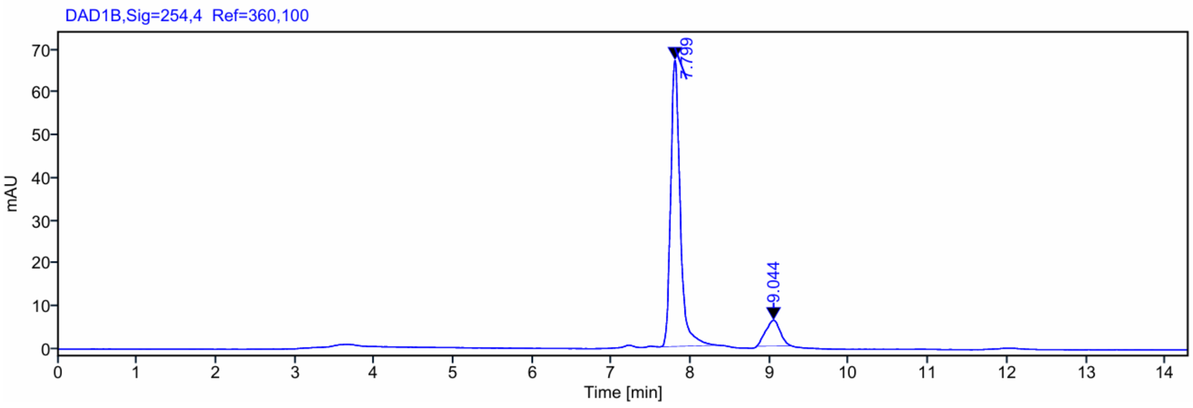

**Signal:**	DAD1B,Sig=254,4 Ref=360,100			
**RT [min]**	**Width [min]**	**Area**	**Height**	**Area%**
7.799	0.75	559.42	67.03	87.90
9.044	0.44	77.02	6.05	12.10
	**Sum**	**636.44**		

(*R*)-3-azido-2-(naphthalen-1-yl)phenyl 1,1,2,2,3,3,4,4,4-nonafluorobutane-1-sulfonate (11)

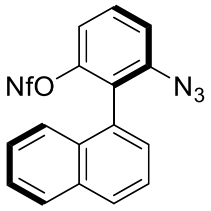

11

Figure S56. *rac*-**11**

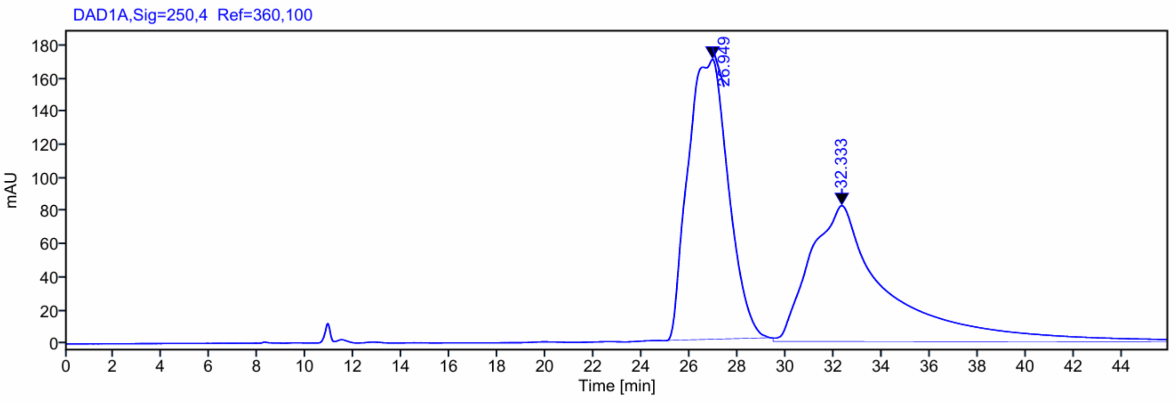

Signal: DAD1A,Sig=250,4 Ref=360,100

RT [min]	Width [min]	Area	Height	Area%
26.949	5.30	19706.95	168.86	50.90
32.333	19.93	19008.05	81.97	49.10
	**Sum**	**38715.00**		

Figure S57. (*R*)-**11** (e.r. = 97:3)

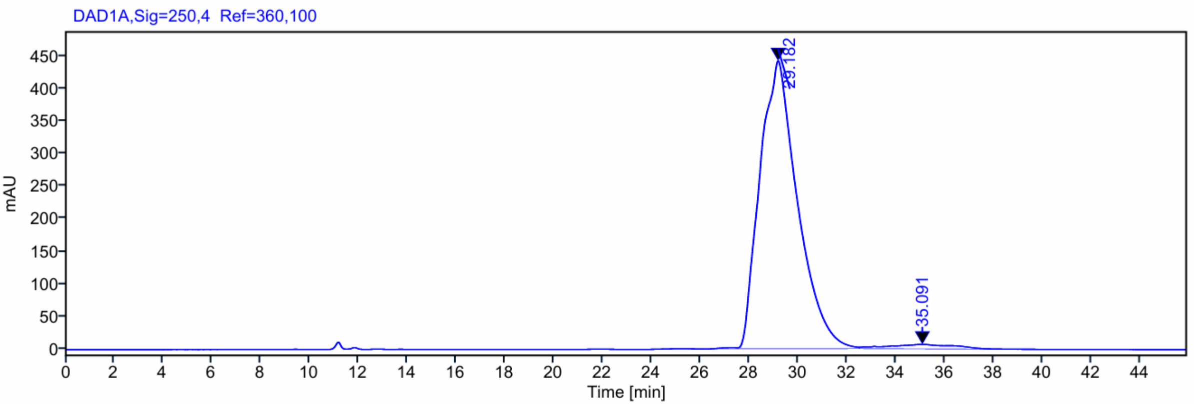

Signal: DAD1A,Sig=250,4 Ref=360,100

RT [min]	Width [min]	Area	Height	Area%
29.182	6.07	47305.39	440.40	97.32
35.091	8.67	1304.93	6.90	2.68
	**Sum**	**48610.32**		

(*R*)-3-(1H-imidazol-1-yl)-2-(naphthalen-1-yl)phenyl 1,1,2,2,3,3,4,4,4-nonafluorobutane-1-sulfonate (12)

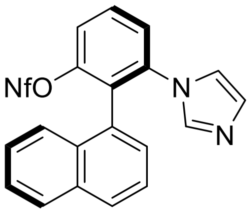

12

Figure S58. *rac*-**12**

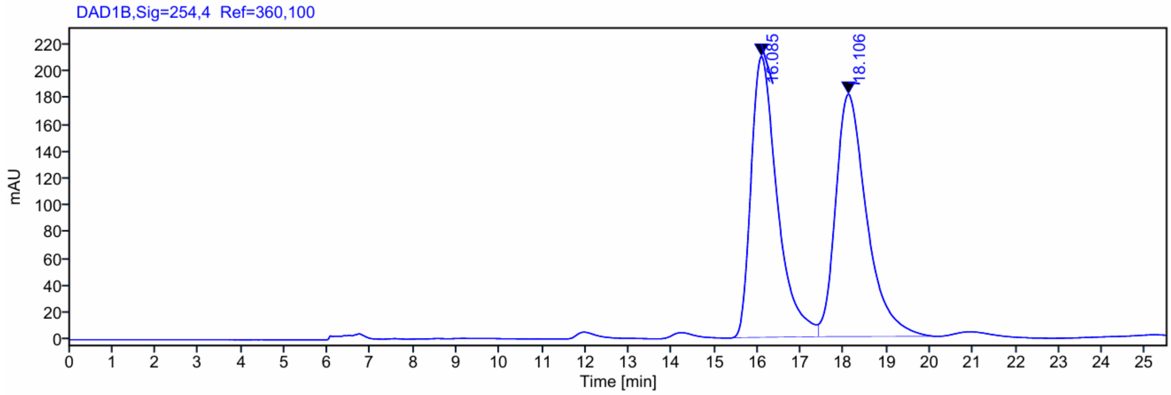

Signal:	DAD1B,Sig=254,4 Ref=360,100			
**RT [min]**	**Width [min]**	**Area**	**Height**	**Area%**
16.085	2.35	8867.32	209.63	49.44
18.106	2.71	9068.09	180.81	50.56
	**Sum**	**17935.41**		

Figure S59. (*R*)-**12** (e.r. = 97:3)

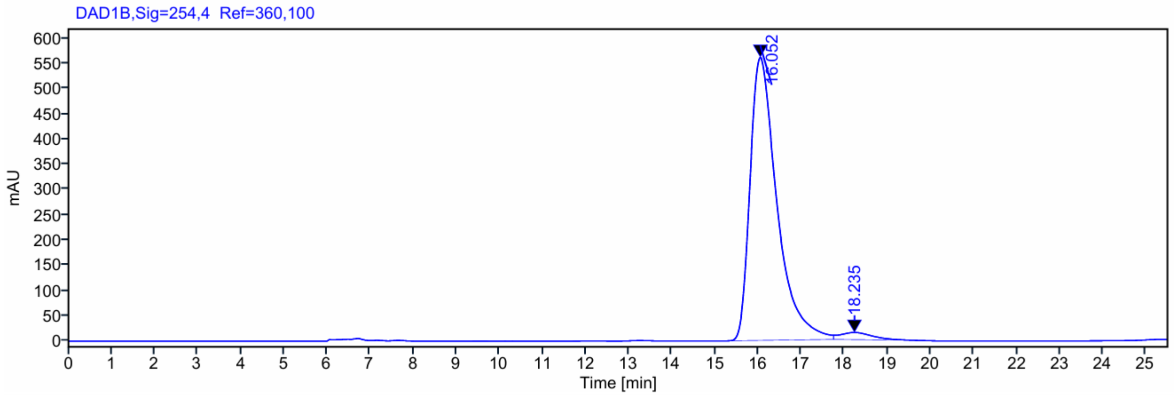

Signal:	DAD1B,Sig=254,4 Ref=360,100			
**RT [min]**	**Width [min]**	**Area**	**Height**	**Area%**
16.052	2.43	24268.24	560.16	97.09
18.235	1.76	726.71	13.96	2.91
	**Sum**	**24994.95**		

(*R*)-3-hydroxy-2-(naphthalen-1-yl)phenyl 1,1,2,2,3,3,4,4,4-nonafluorobutane-1-sulfonate (13)

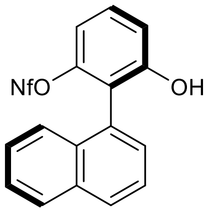

13

Figure S60. *rac*-**13**

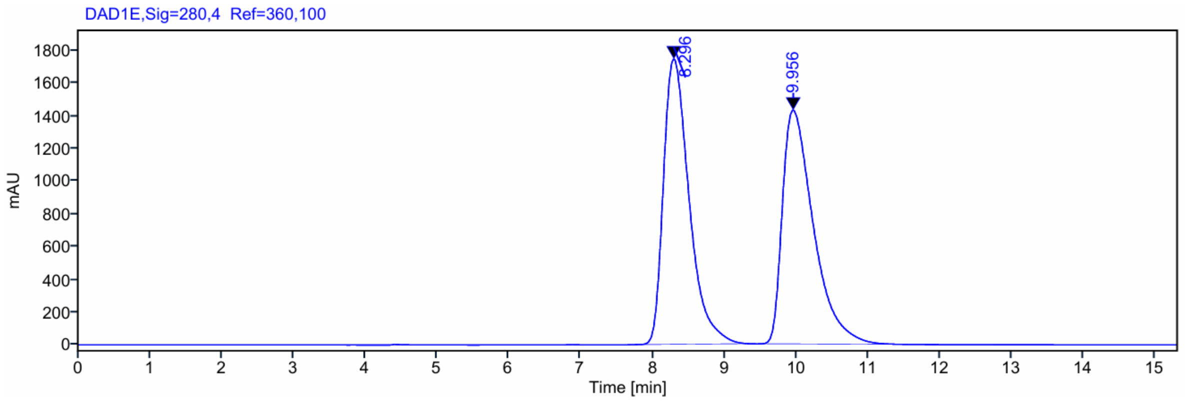

**Signal:**	DAD1E,Sig=280,4 Ref=360,100			
**RT [min]**	**Width [min]**	**Area**	**Height**	**Area%**
8.296	1.57	43375.43	1744.39	50.28
9.956	2.17	42899.79	1428.89	49.72
	**Sum**	**86275.21**		

Figure S61. (*R*)-**13** (e.r. = 98:2)

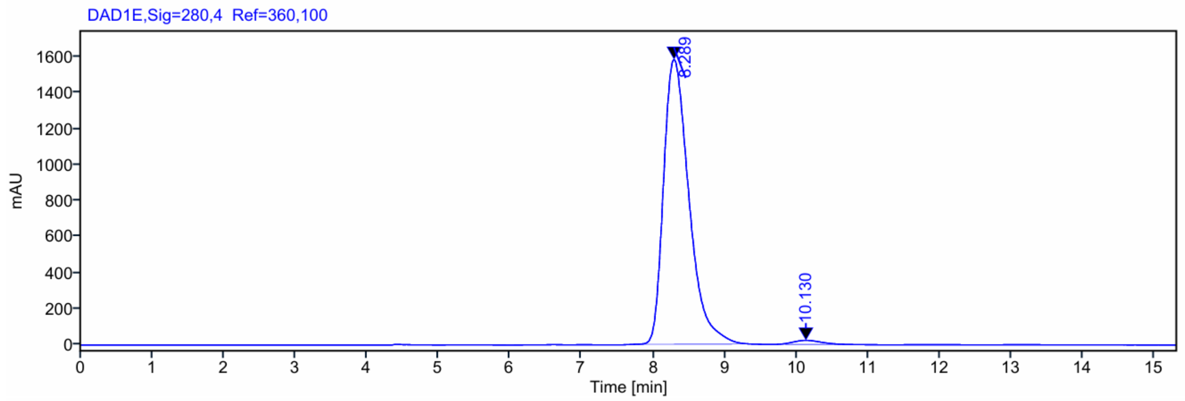

**Signal:**	DAD1E,Sig=280,4 Ref=360,100			
**RT [min]**	**Width [min]**	**Area**	**Height**	**Area%**
8.289	1.66	39130.38	1582.79	98.47
10.130	1.05	609.79	22.33	1.53
	**Sum**	**39740.16**		

(*R*)-2-(naphthalen-1-yl)-[1,1'-biphenyl]-3-yl 1,1,2,2,3,3,4,4,4-nonafluorobutane-1-sulfonate (14)

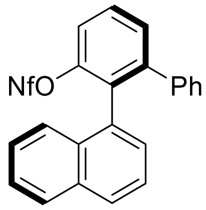

14

Figure S62. *rac*-**14**

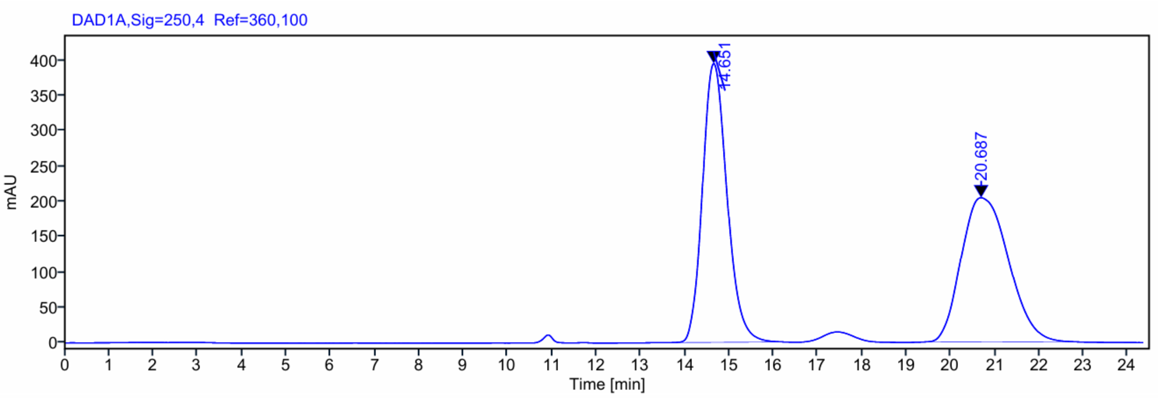

Signal: DAD1A,Sig=250,4 Ref=360,100

RT [min]	Width [min]	Area	Height	Area%
14.651	2.57	15276.64	395.53	50.11
20.687	3.44	15209.81	204.55	49.89
	**Sum**	**30486.45**		

Figure S63. (*R*)-**14** (e.r. = 97:3)

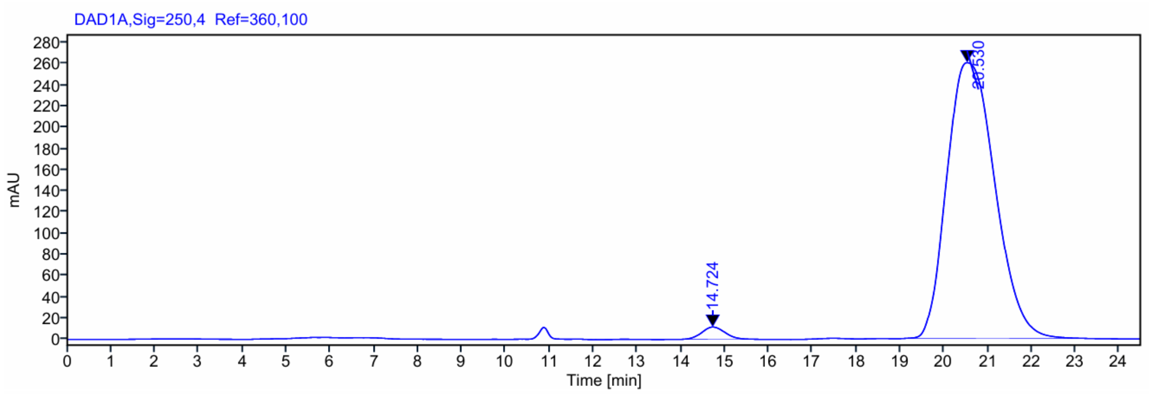

Signal: DAD1A,Sig=250,4 Ref=360,100

RT [min]	Width [min]	Area	Height	Area%
14.724	1.80	428.24	11.62	2.11
20.530	4.25	19891.42	260.69	97.89
	**Sum**	**20319.66**		

9 NMR Spectra

2-(naphthalen-1-yl)-1,3-phenylene bis(1,1,2,2,3,3,4,4,4-nonafluorobutane-1-sulfonate) (2a)

Figure S64. **^{1}H NMR** (500 MHz, $CDCl_3$, 298 K) of **2a**

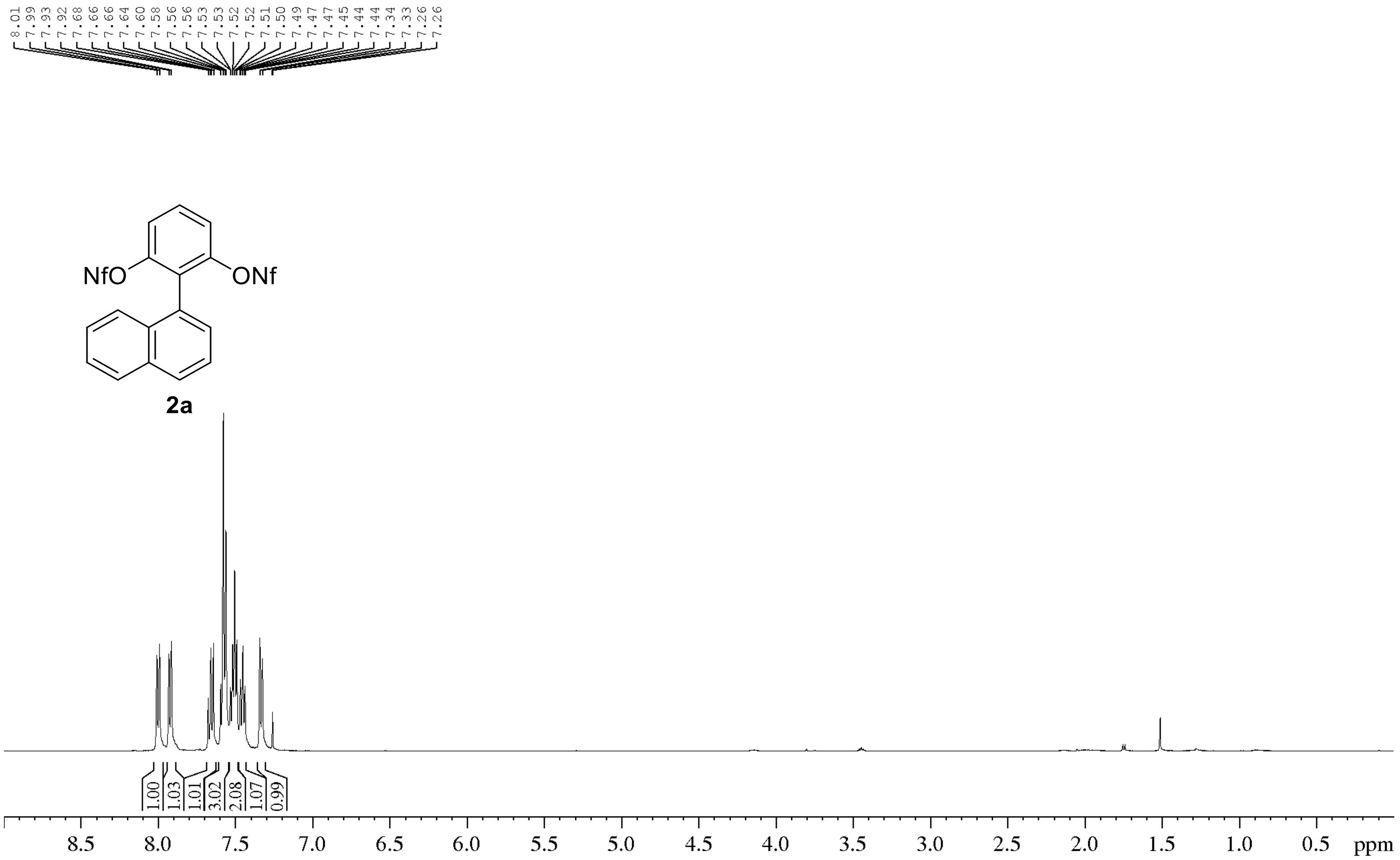

Figure S65. $^{13}C\{^1H\}$ NMR (126 MHz, $CDCl_3$, 298 K) of **2a**

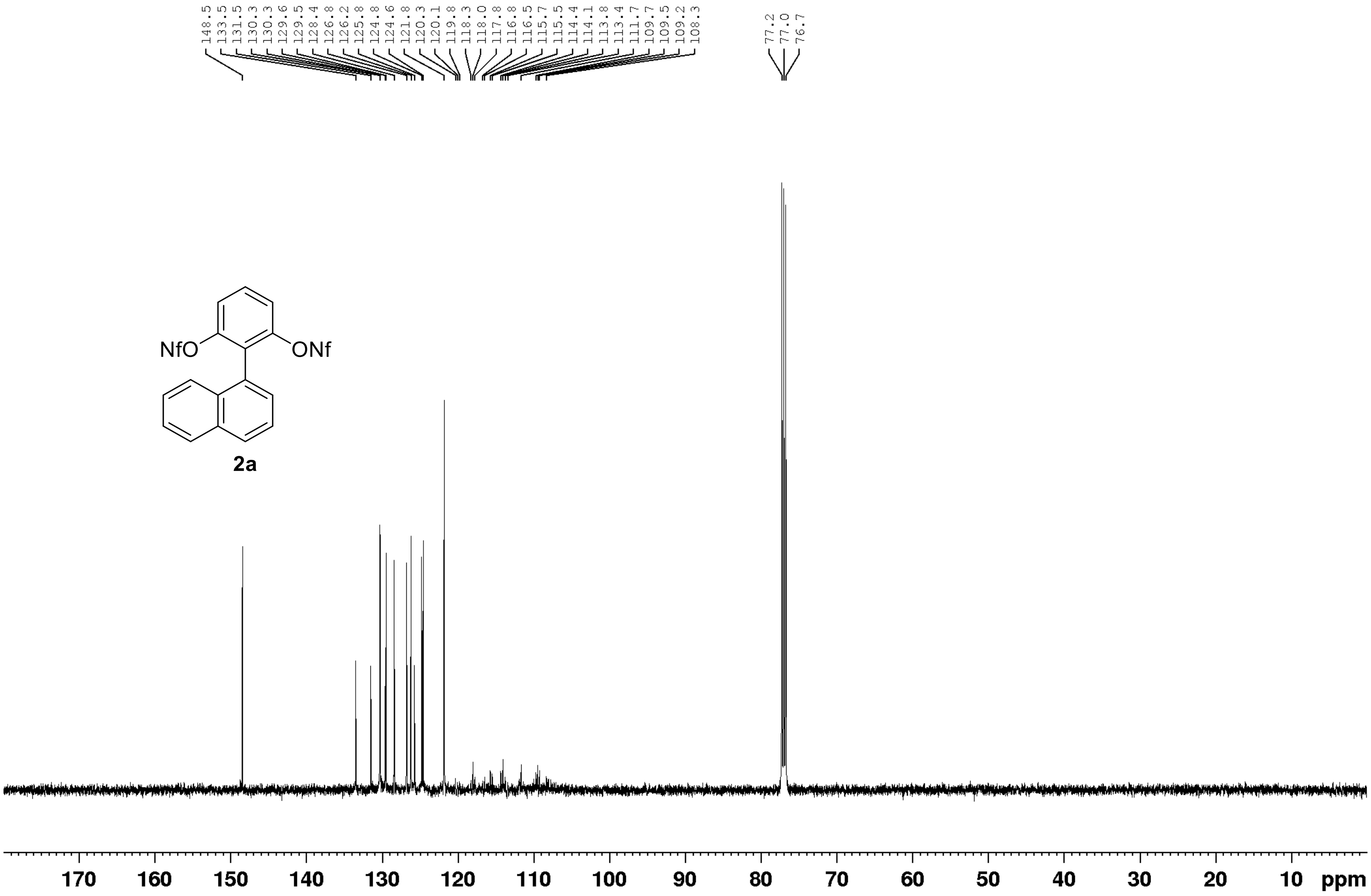

Figure S66. **^{19}F NMR** (471 MHz, $CDCl_3$, 298 K) of **2a**

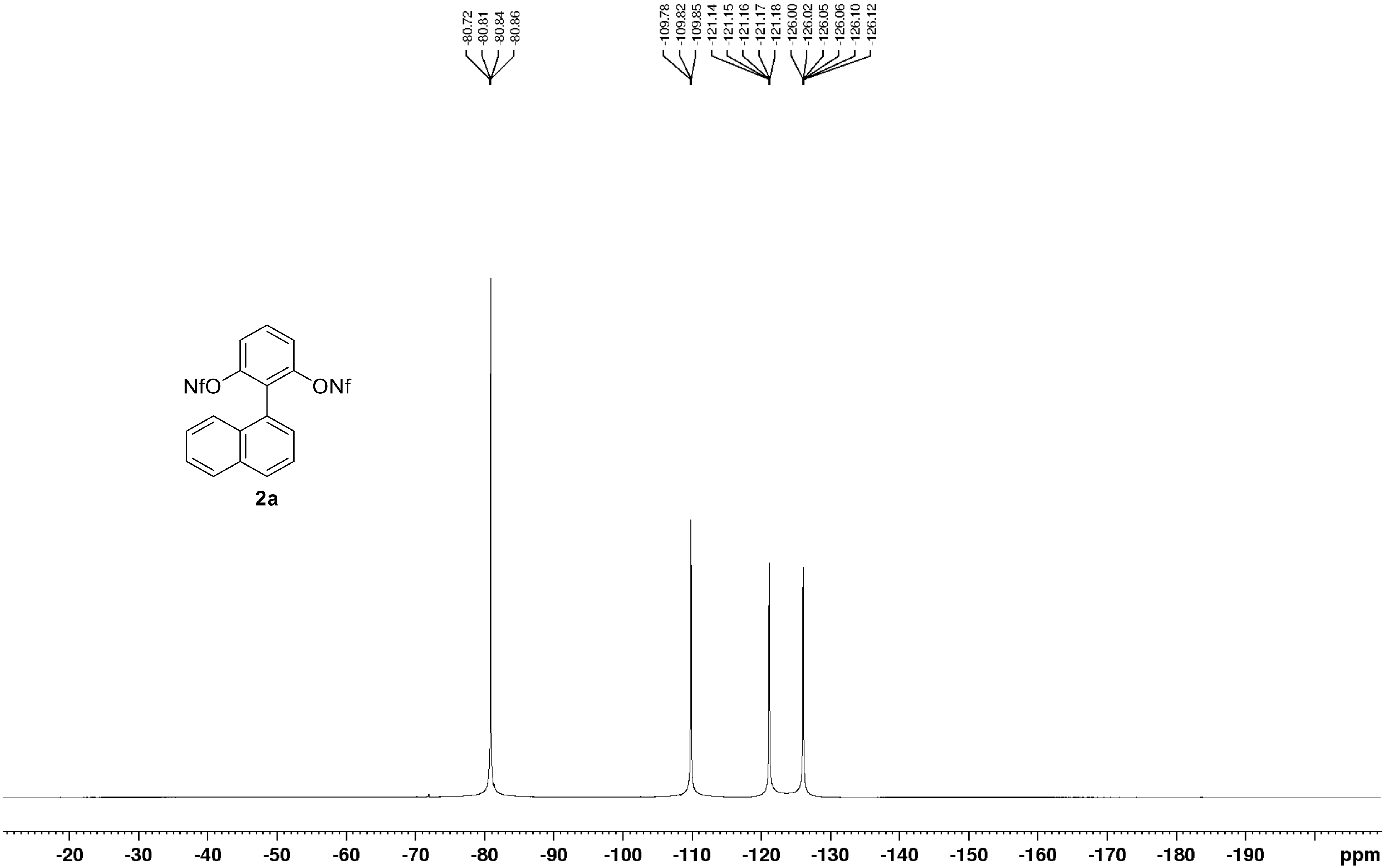

2a

2-(4-methylnaphthalen-1-yl)-1,3-phenylene bis(1,1,2,2,3,3,4,4,4-nonafluorobutane-1-sulfonate) (**2b**)

Figure S67. **^{1}H NMR** (400 MHz, $CDCl_3$, 298 K) of **2b**

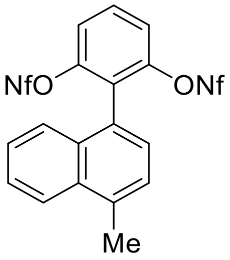

2b

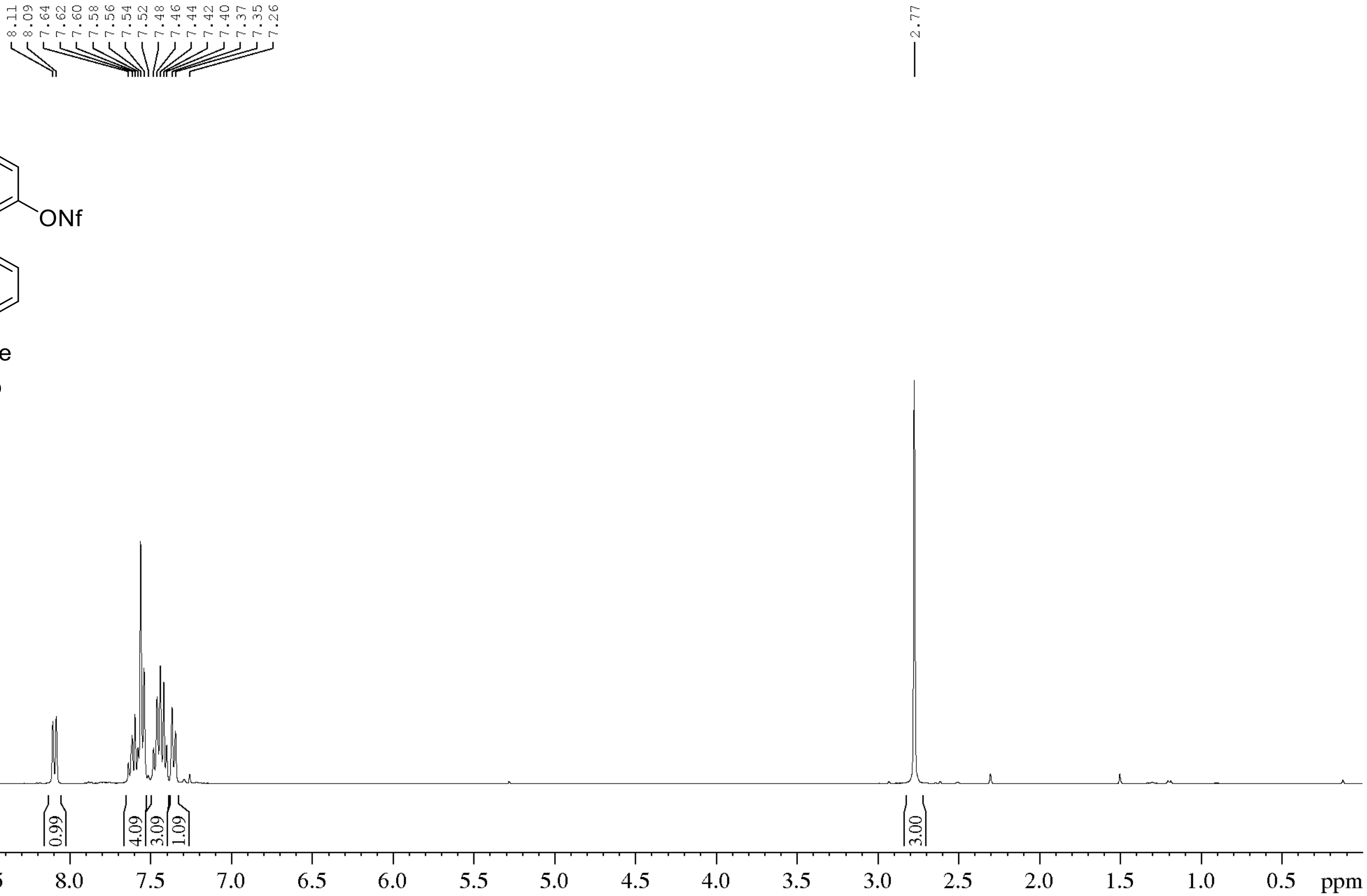

Figure S68. **^{13}C{^{1}H} NMR** (101 MHz, $CDCl_3$, 298 K) of **2b**

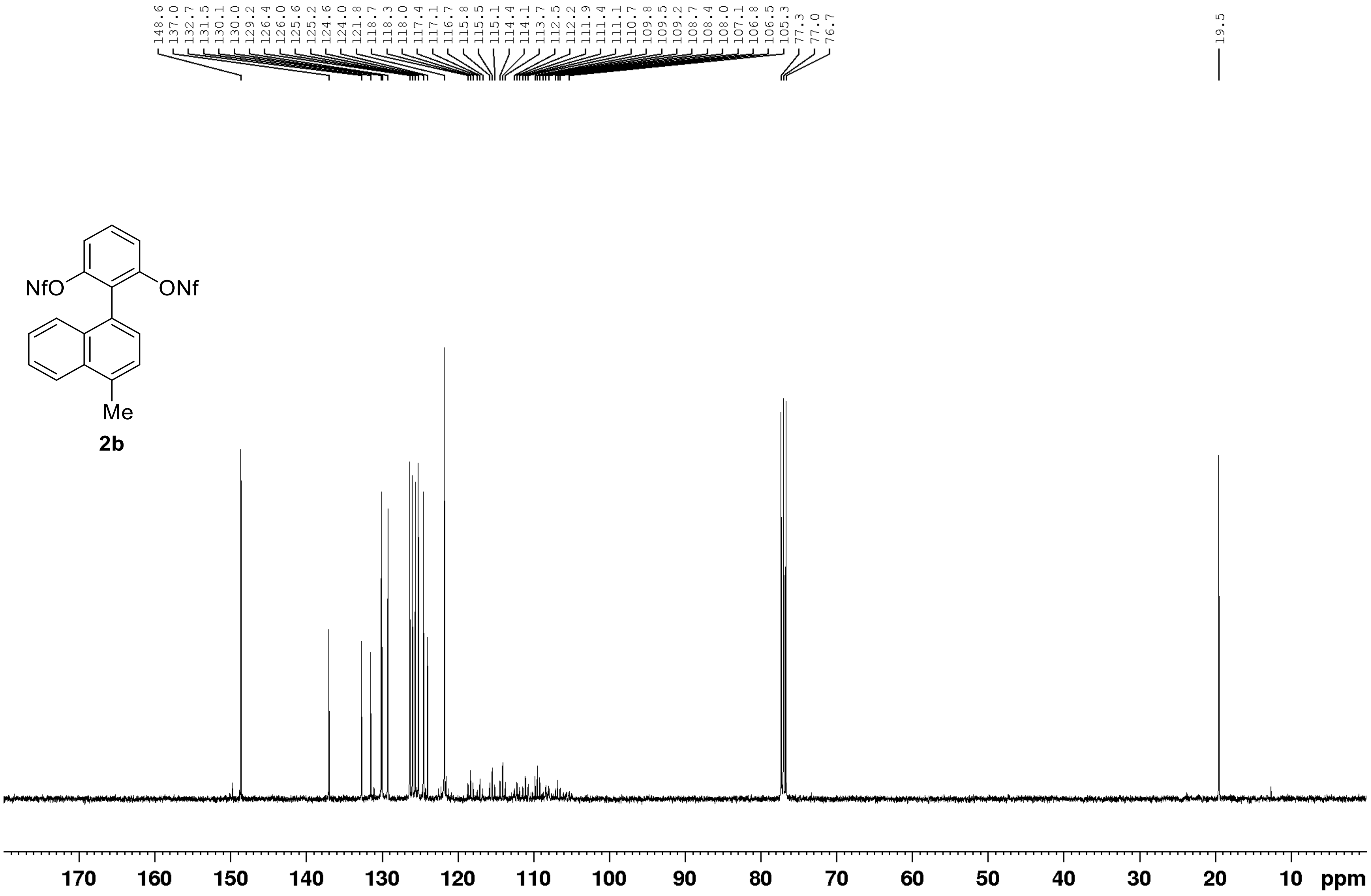

Figure S69. **^{19}F NMR** (471 MHz, $CDCl_3$, 298 K) of **2b**

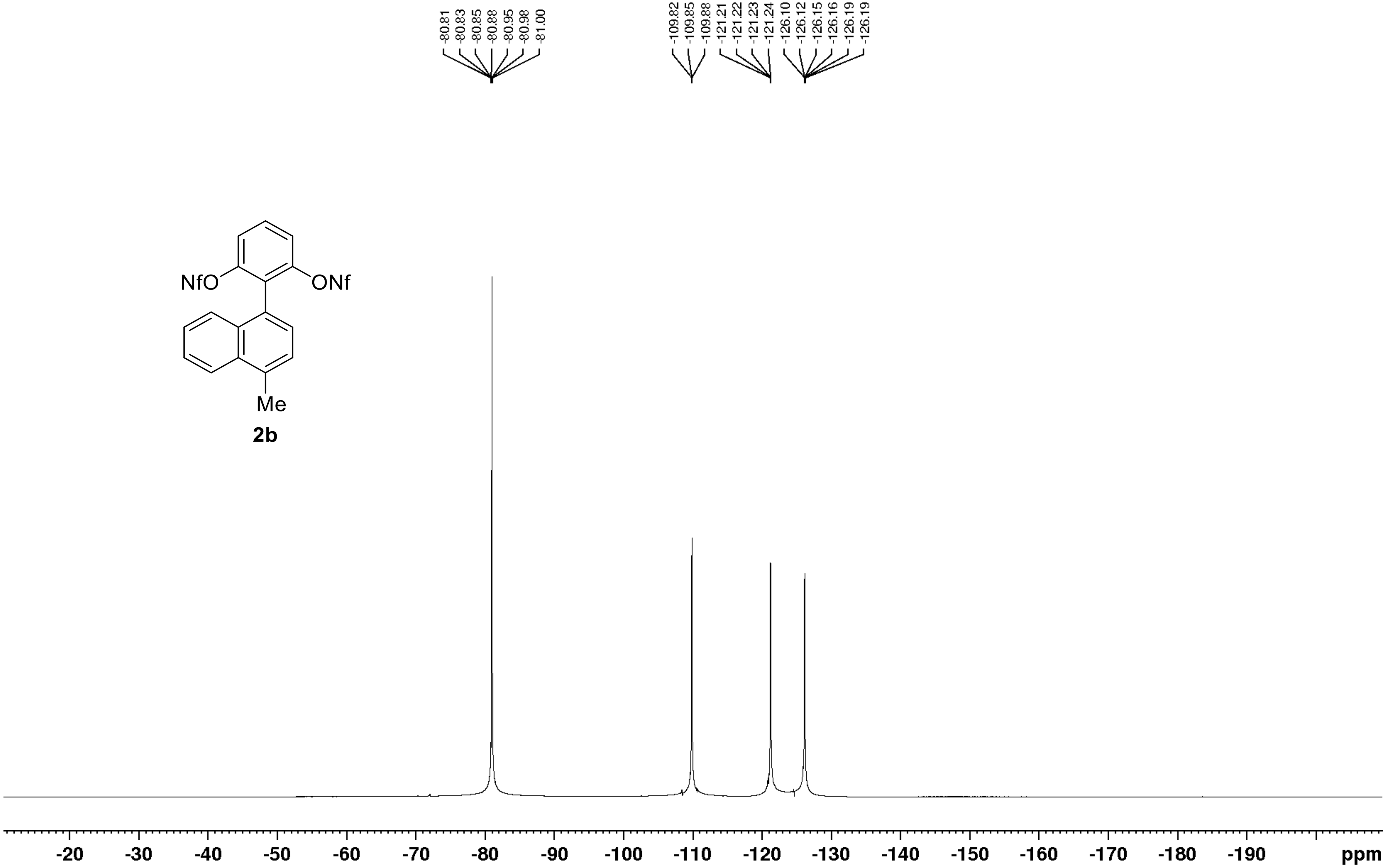

2-(4-fluoronaphthalen-1-yl)-1,3-phenylene bis(1,1,2,2,3,3,4,4,4-nonafluorobutane-1-sulfonate) (**2c**)

Figure S70. **^{1}H NMR** (500 MHz, $CDCl_3$, 298 K) of **2c**

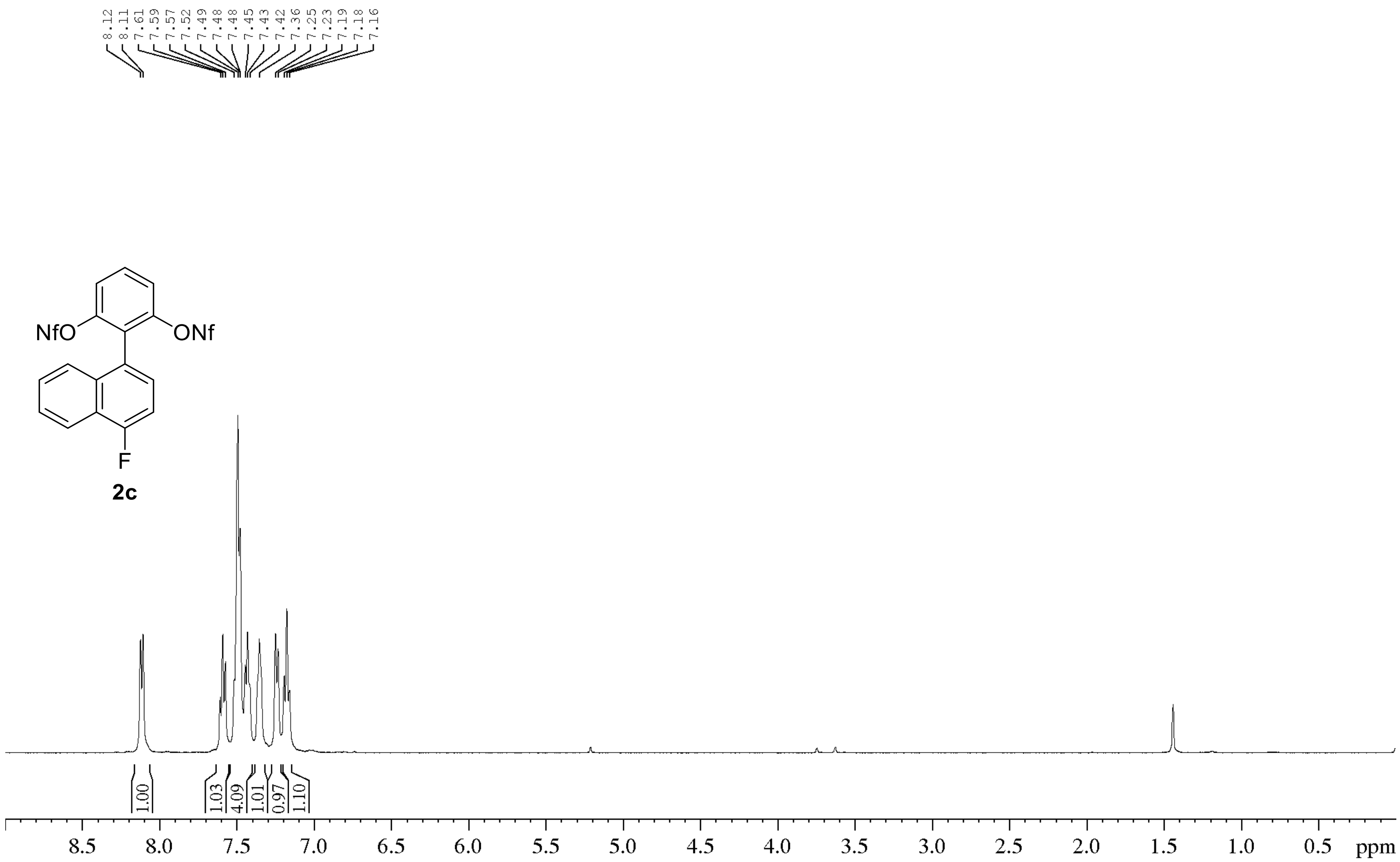

Figure S71. ^{13}**C{^{1}H} NMR** (126 MHz, $CDCl_3$, 298 K) of **2c**

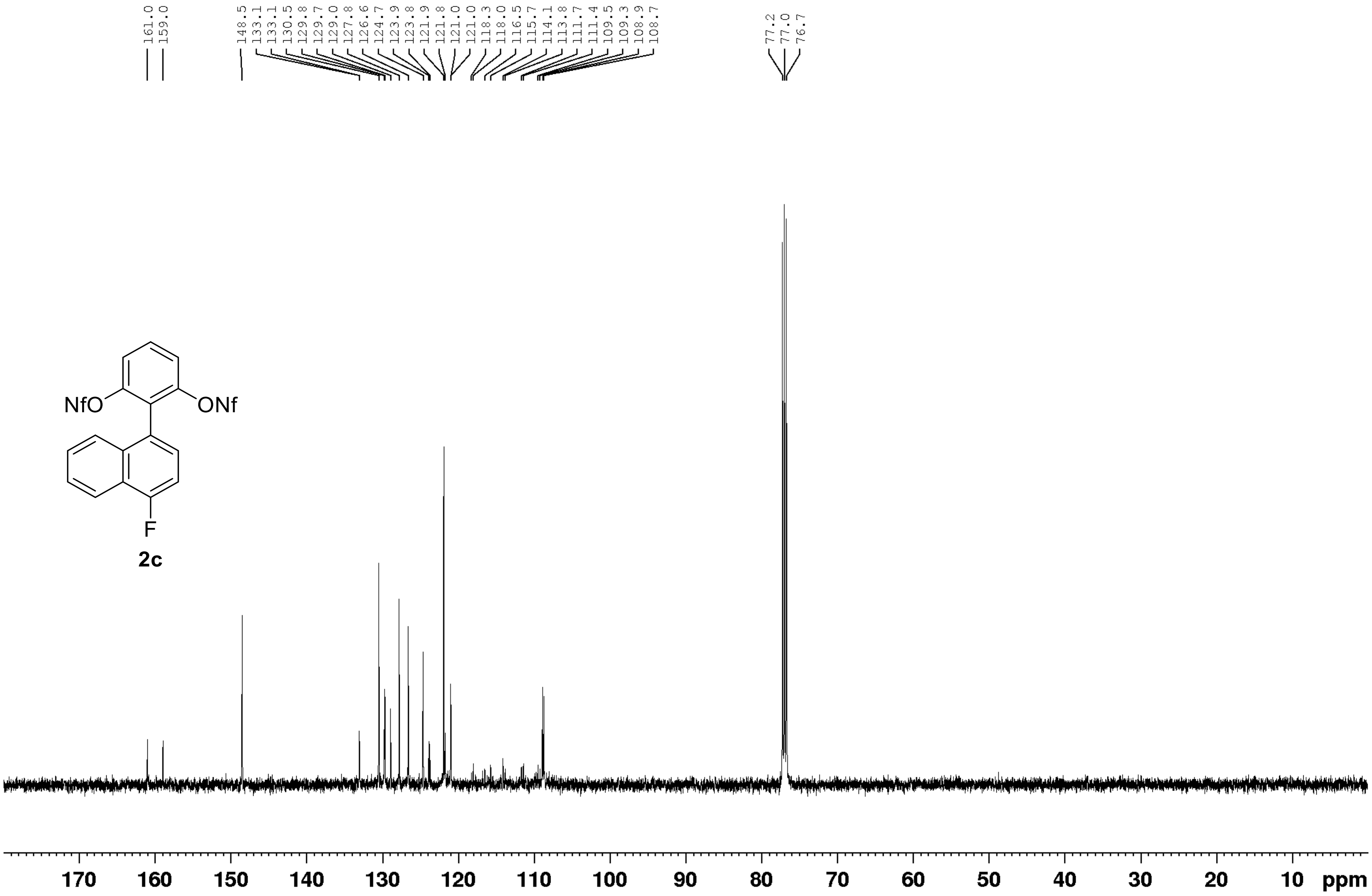

Figure S72. **^{19}F NMR** (471 MHz, $CDCl_3$, 298 K) of **2c**

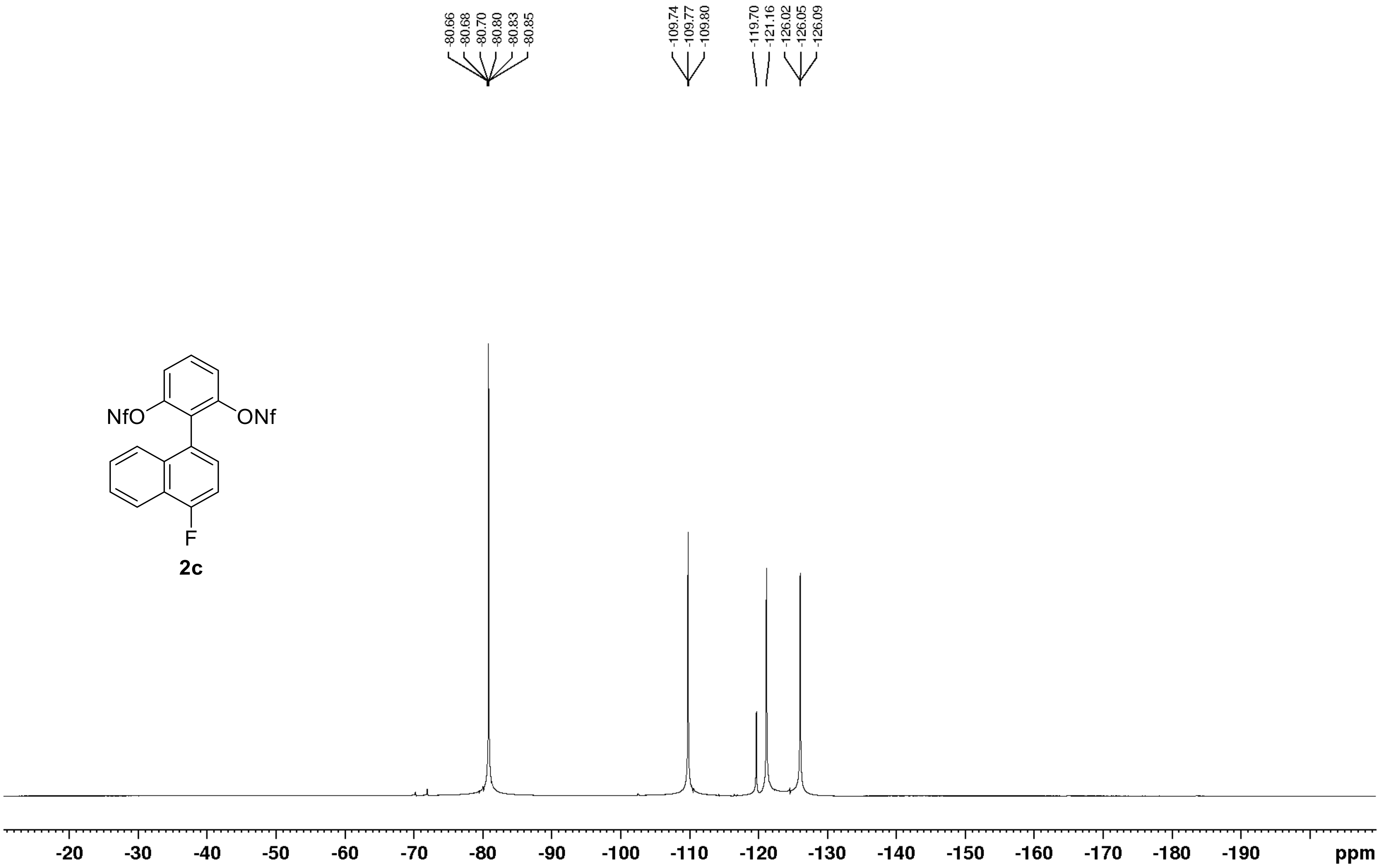

2-(4-bromonaphthalen-1-yl)-1,3-phenylene bis(1,1,2,2,3,3,4,4,4-nonafluorobutane-1-sulfonate) (2d)

Figure S73. ^{1}**H NMR** (500 MHz, $CDCl_3$, 298 K) of **2d**

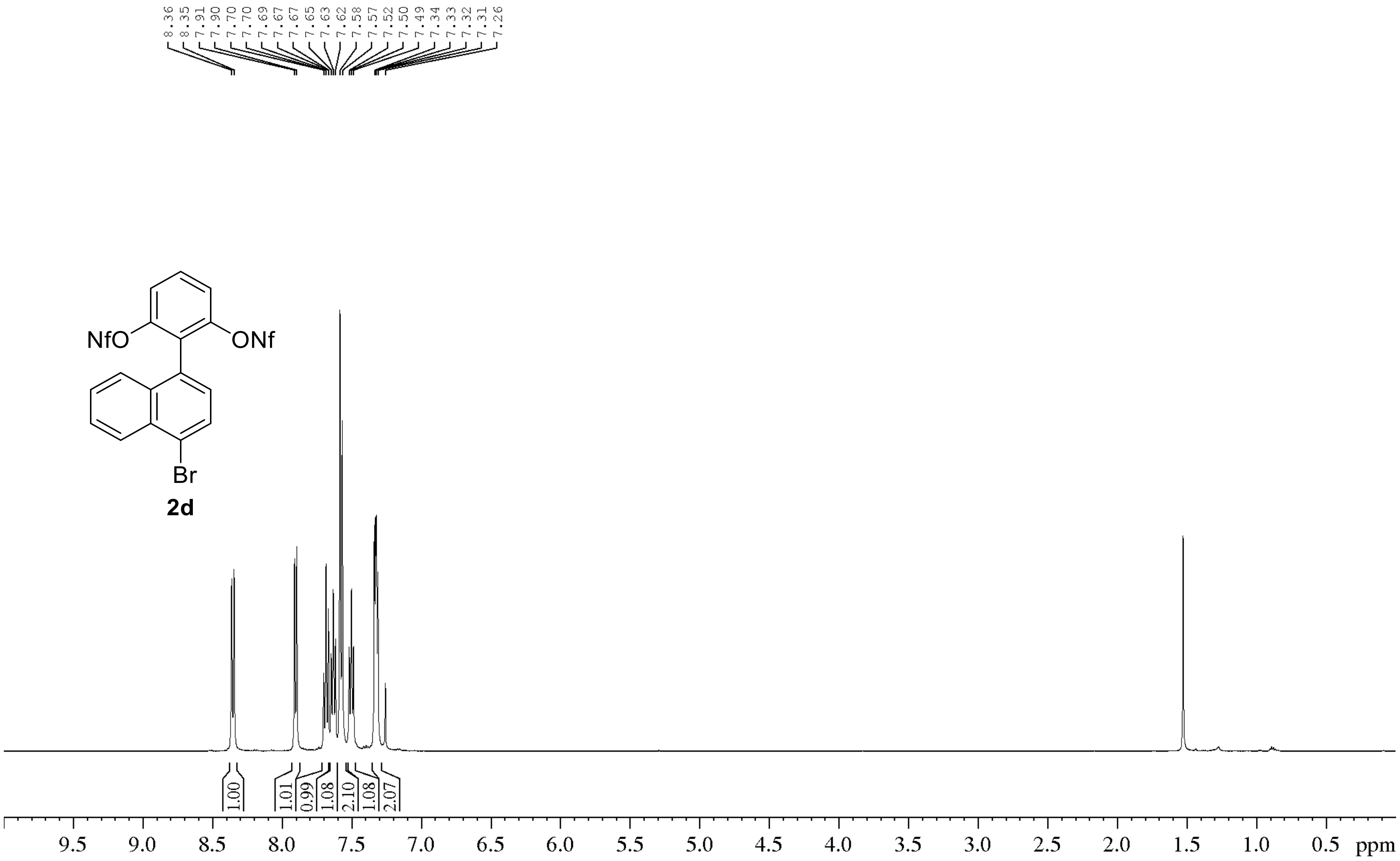

Figure S74. ^{13}**C{**^{1}**H} NMR** (126 MHz, $CDCl_3$, 298 K) of **2d**

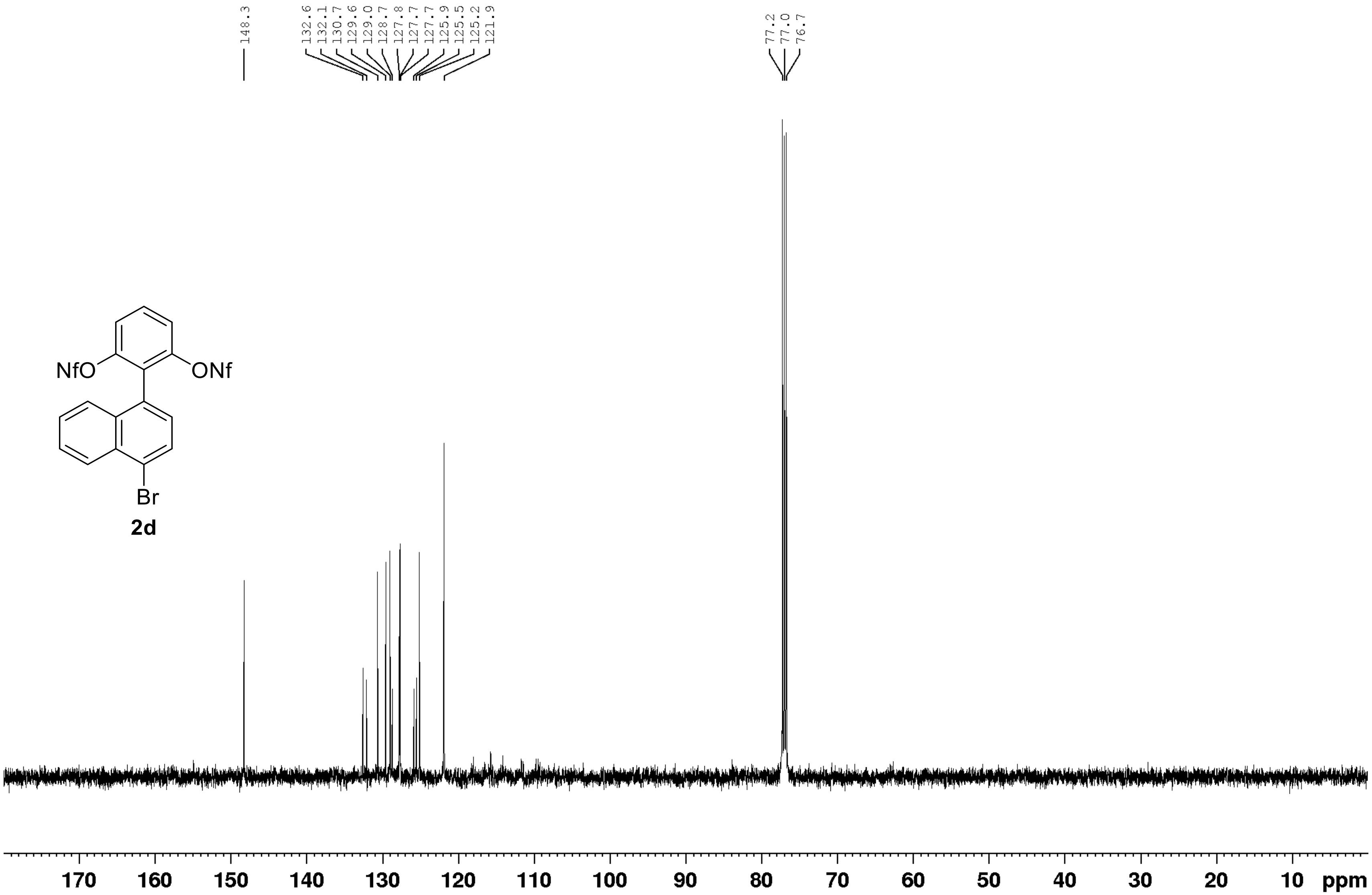

Figure S75. ^{19}**F NMR** (471 MHz, $CDCl_3$, 298 K) of **2d**

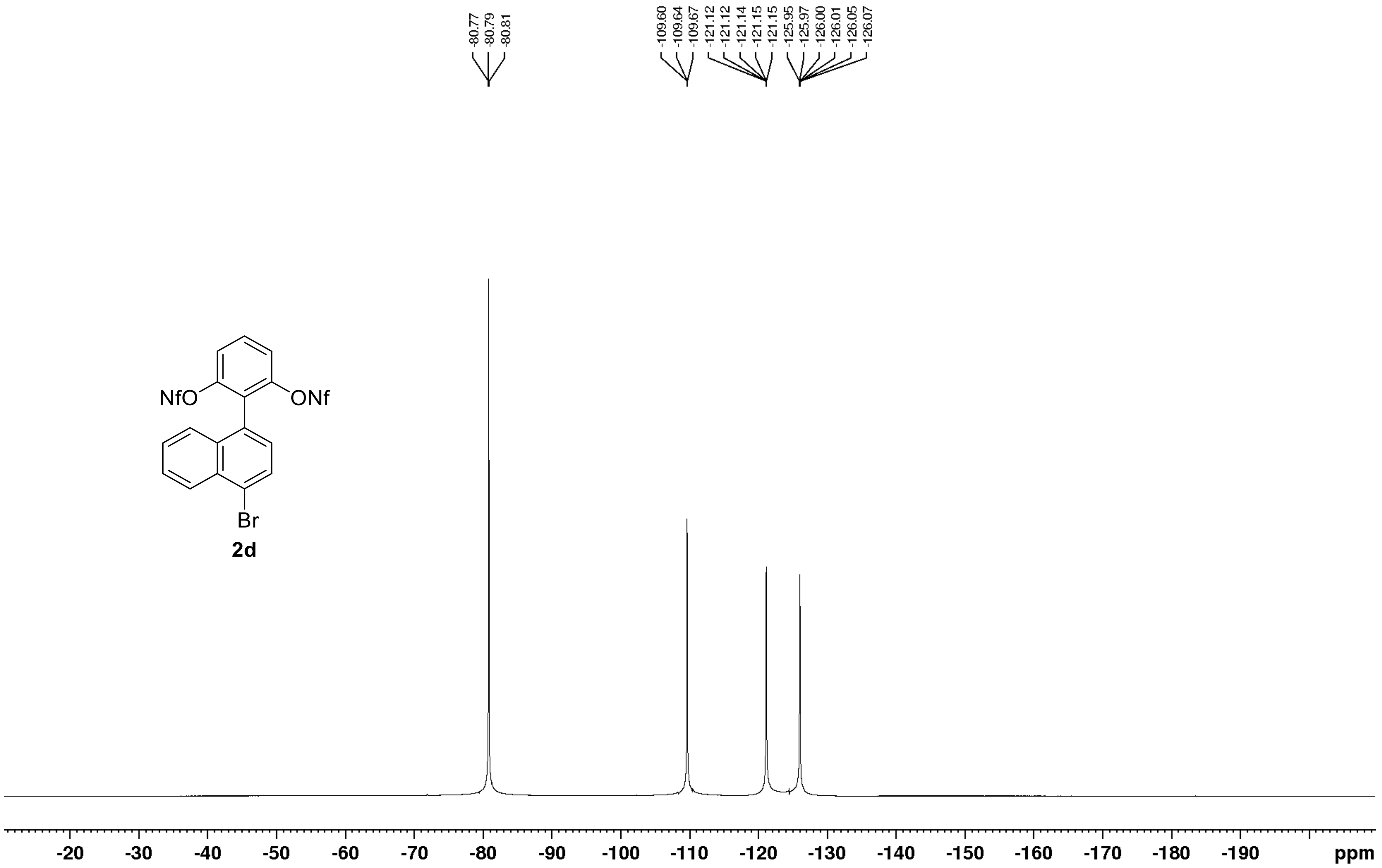

2-(4-cyanonaphthalen-1-yl)-1,3-phenylene bis(1,1,2,2,3,3,4,4,4-nonafluorobutane-1-sulfonate) (**2e**)

Figure S76. **^{1}H NMR** (500 MHz, $CDCl_3$, 298 K) of **2e**

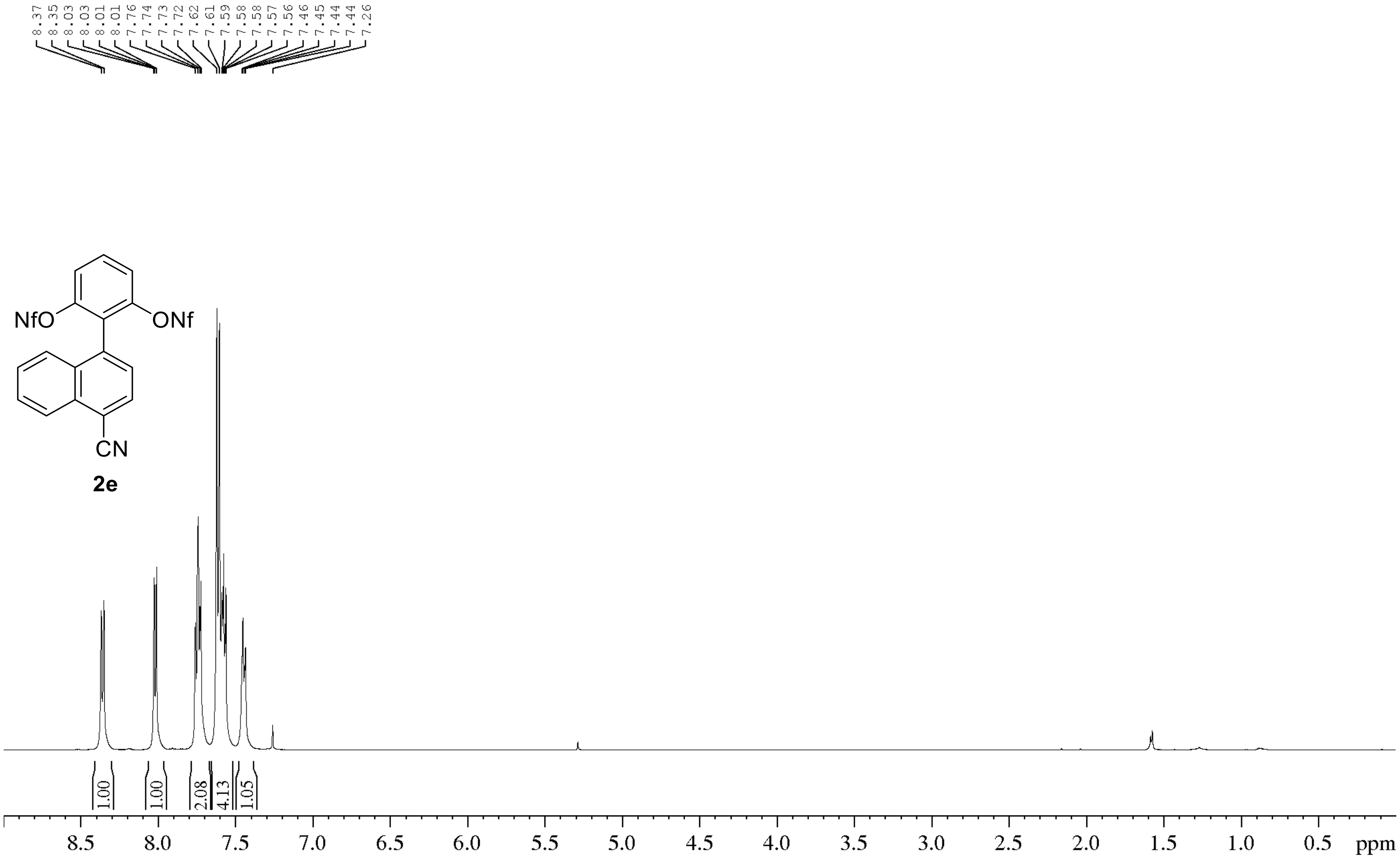

Figure S77. $^{13}C\{^{1}H\}$ **NMR** (126 MHz, $CDCl_3$, 298 K) of **2e**

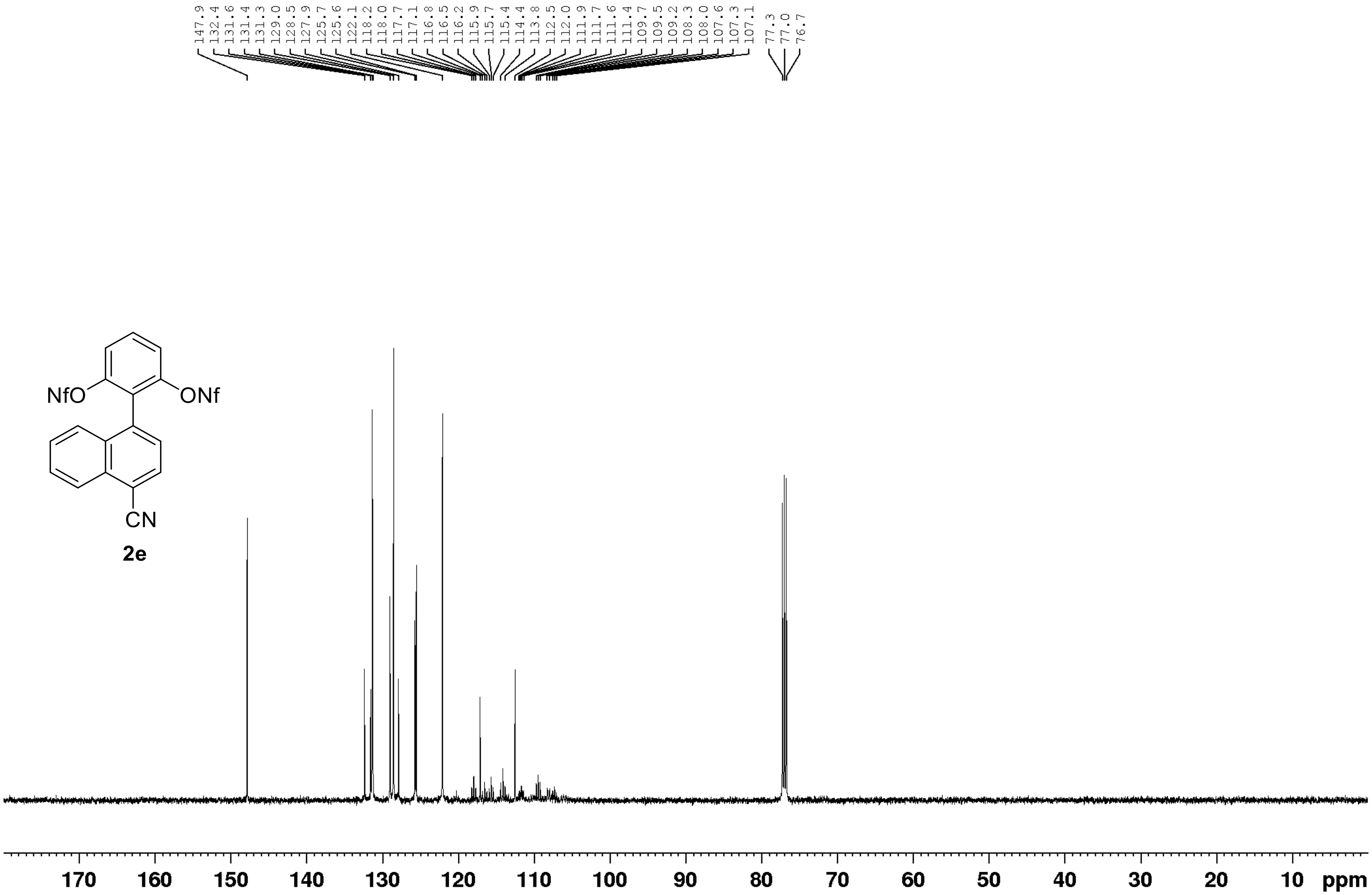

Figure S78. **^{19}F NMR** (471 MHz, $CDCl_3$, 298 K) of **2e**

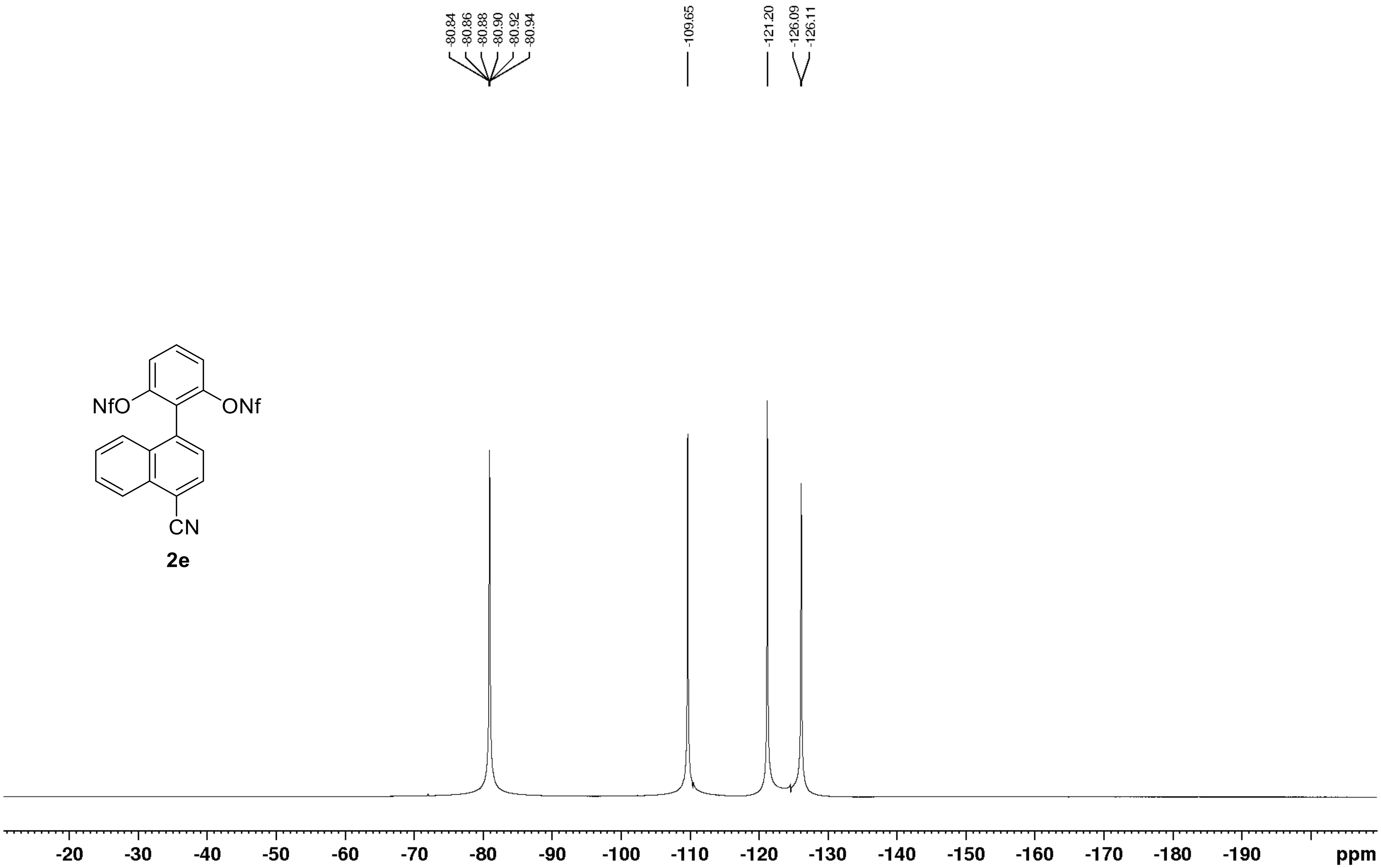

2-(5-phenylnaphthalen-1-yl)-1,3-phenylene bis(1,1,2,2,3,3,4,4,4-nonafluorobutane-1-sulfonate) (2f)

Figure S79. **^{1}H NMR** (500 MHz, $CDCl_3$, 298 K) of **2f**

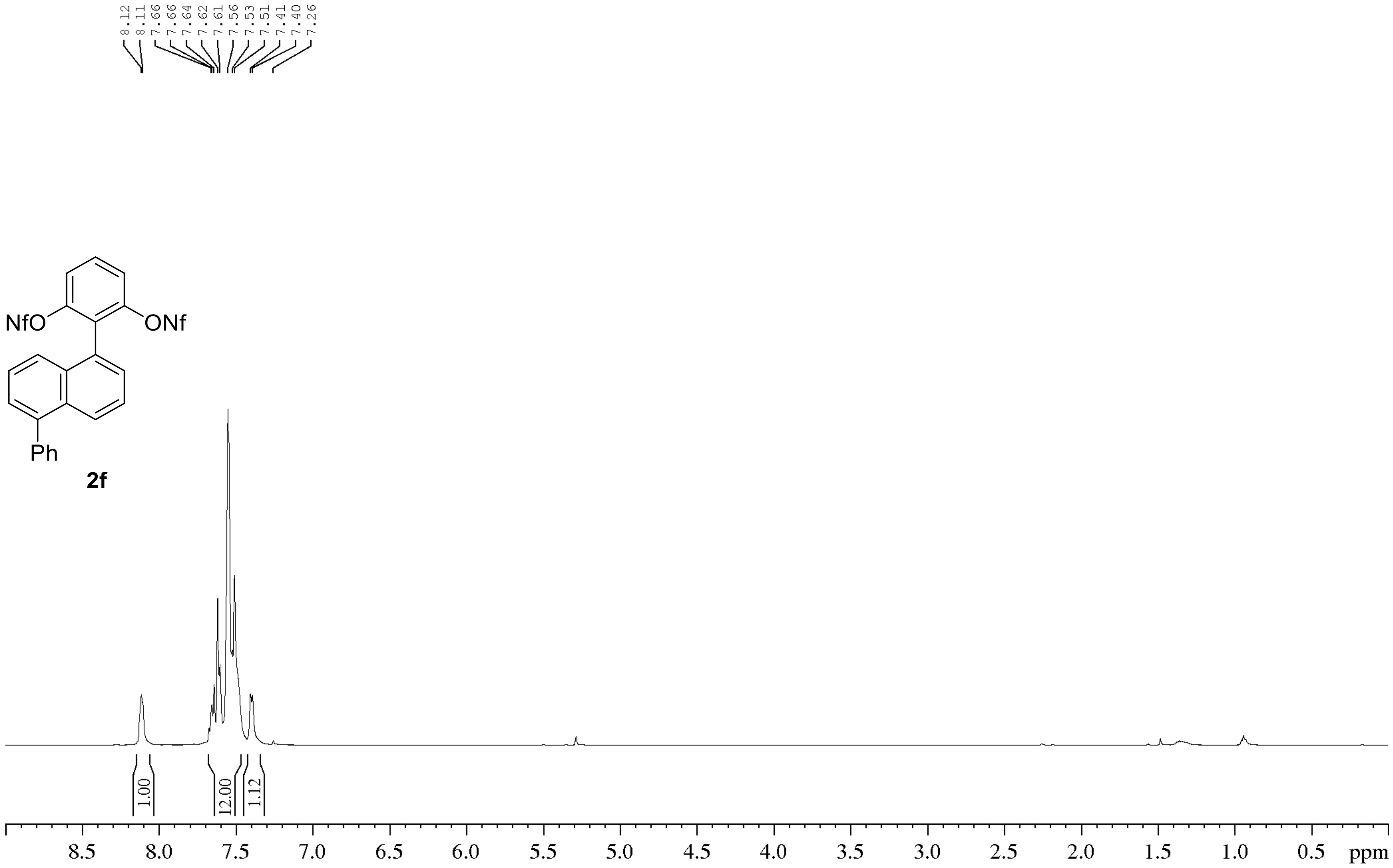

Figure S80. $^{13}C\{^{1}H\}$ **NMR** (126 MHz, $CDCl_3$, 298 K) of **2f**

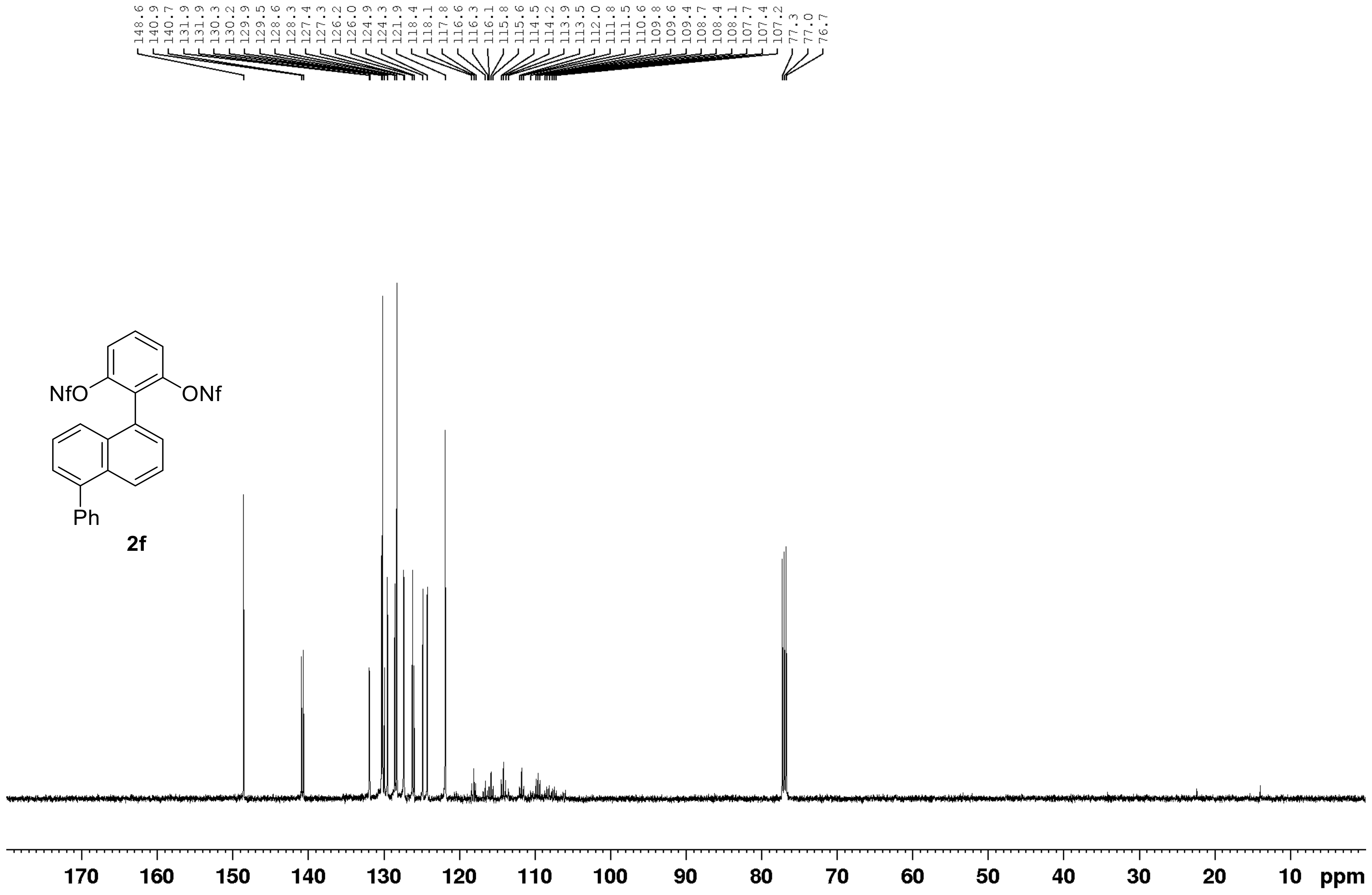

Figure S81. ^{19}**F NMR** (471 MHz, $CDCl_3$, 298 K) of **2f**

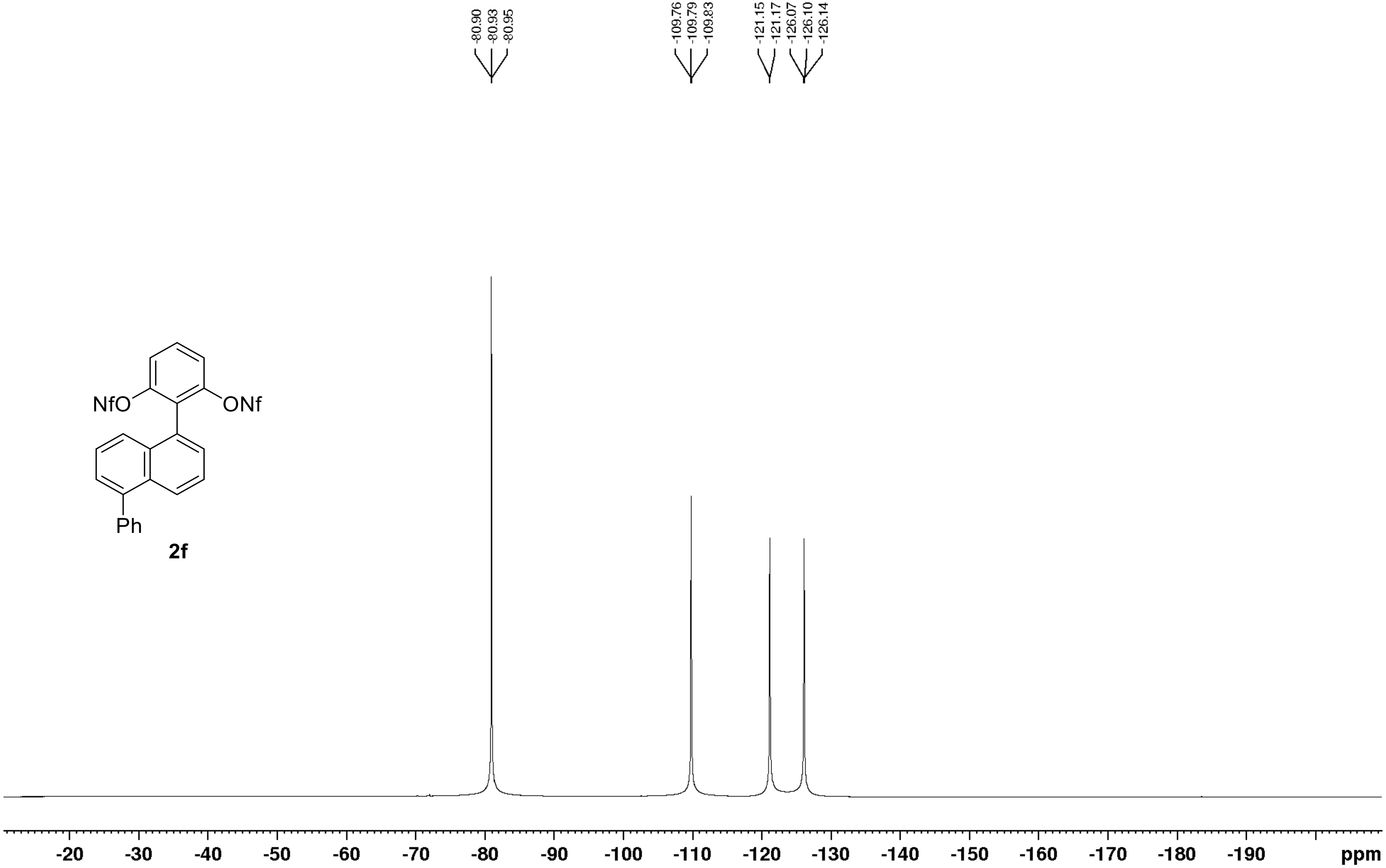

2-(5-((4-methylphenyl)sulfonamido)naphthalen-1-yl)-1,3-phenylene bis(1,1,2,2,3,3,4,4,4-nonafluorobutane-1-sulfonate) (2g)

Figure S82. ^{1}**H NMR** (500 MHz, $CDCl_3$, 298 K) of **2g**

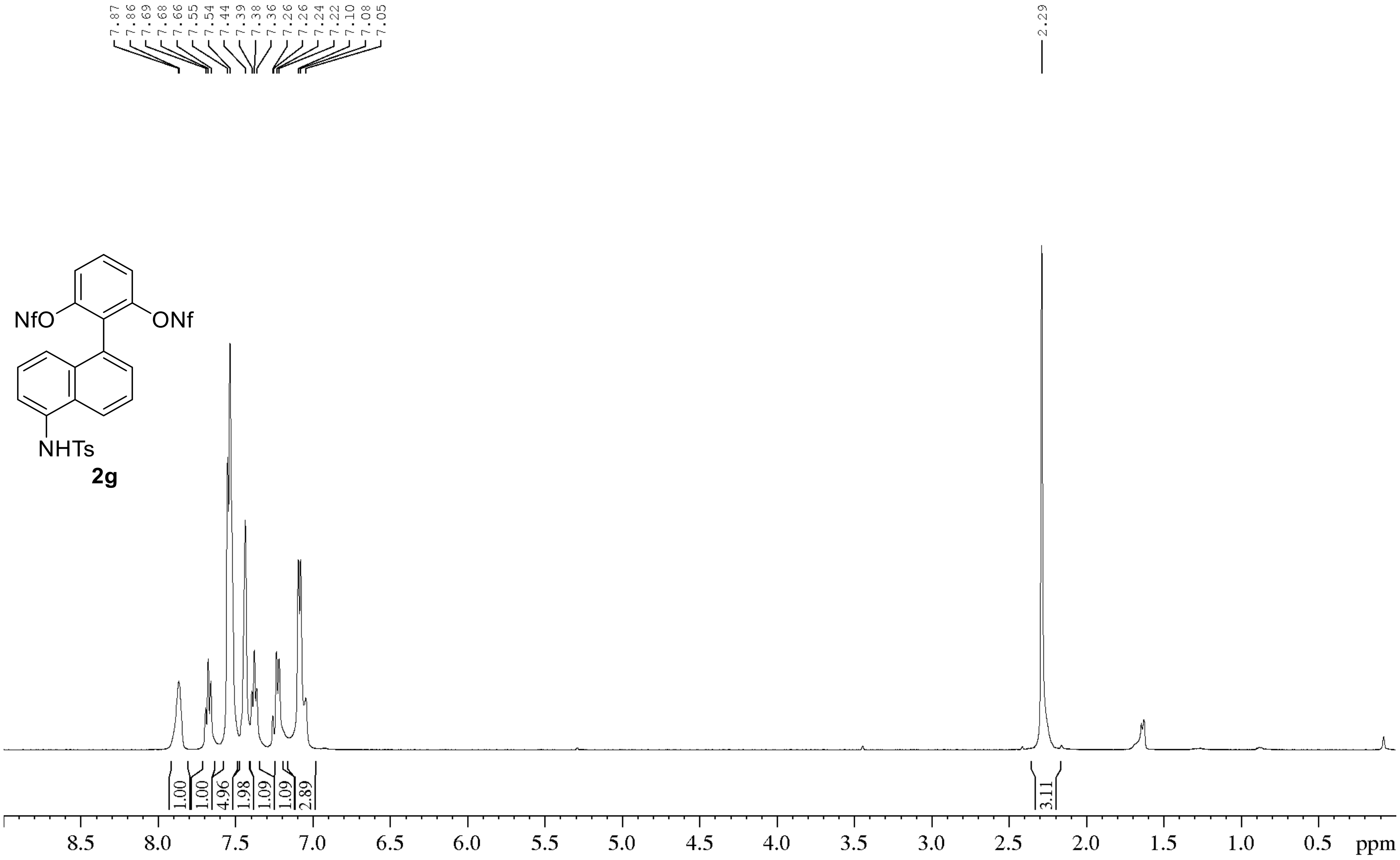

Figure S83. $^{13}C\{^1H\}$ **NMR** (126 MHz, $CDCl_3$, 298 K) of **2g**

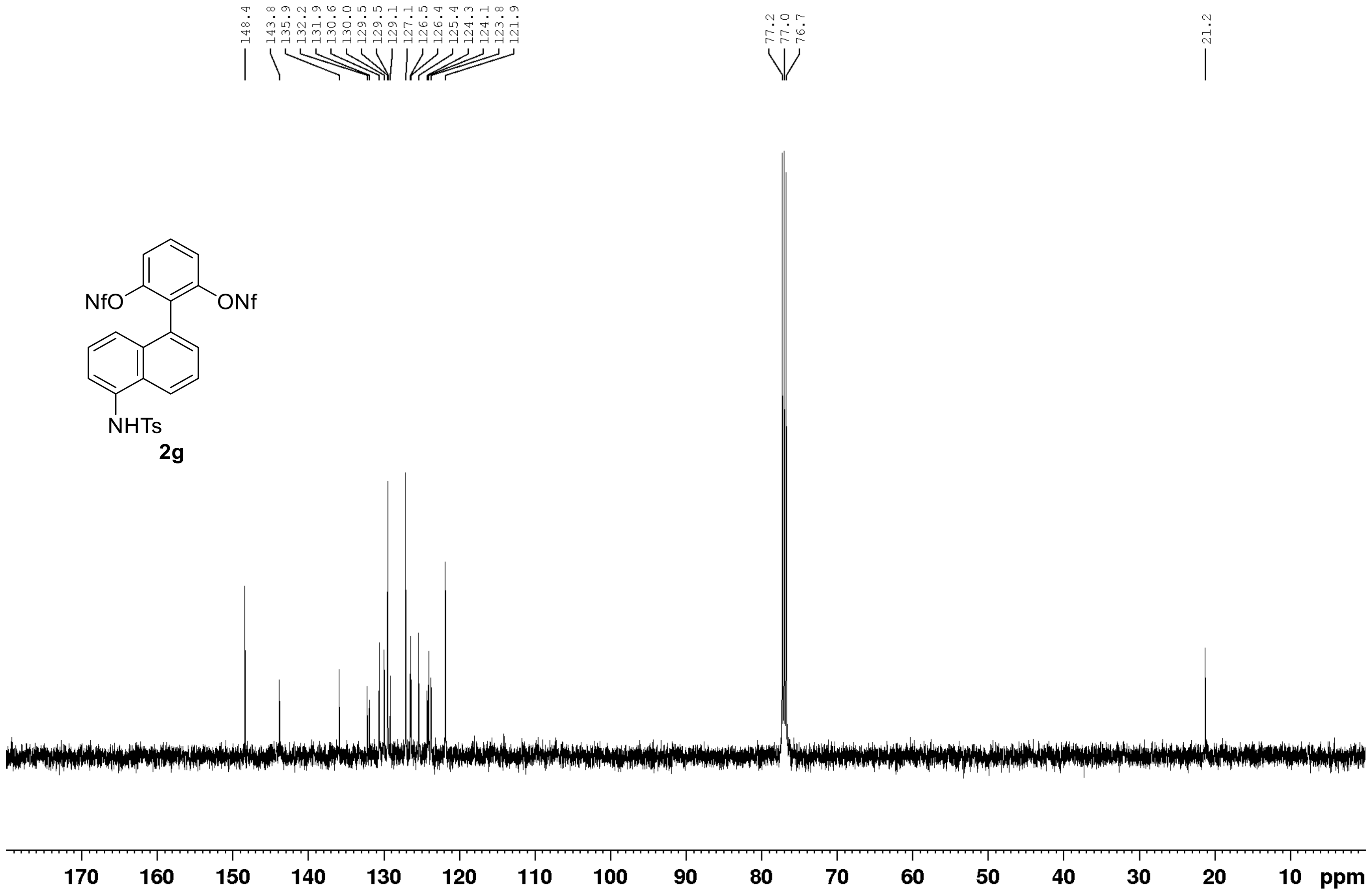

Figure S84. **^{19}F NMR** (471 MHz, $CDCl_3$, 298 K) of **2g**

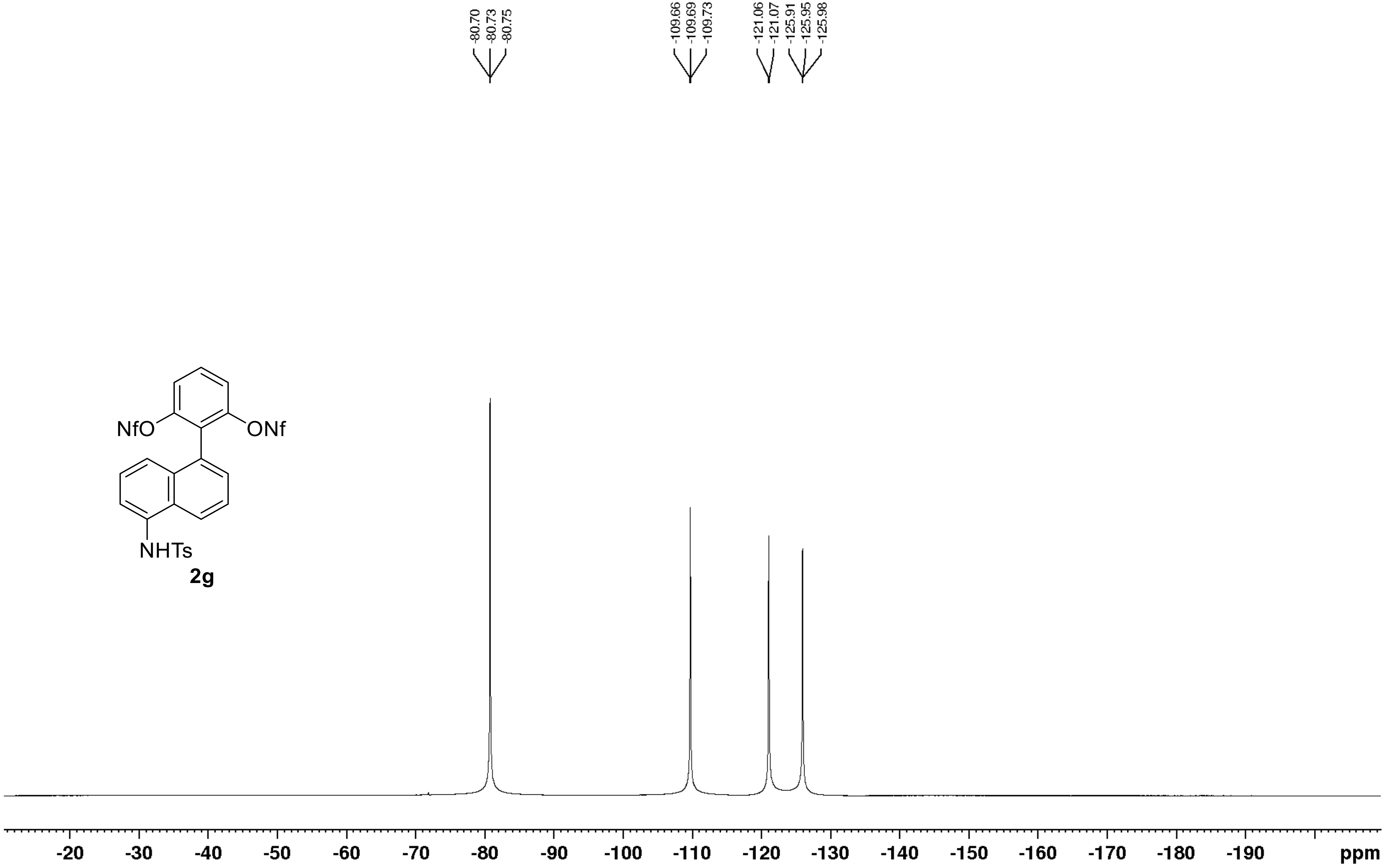

2-(2-methylnaphthalen-1-yl)-1,3-phenylene bis(1,1,2,2,3,3,4,4,4-nonafluorobutane-1-sulfonate) (2h)

Figure S85. ^{1}**H NMR** (500 MHz, $CDCl_3$, 298 K) of **2h**

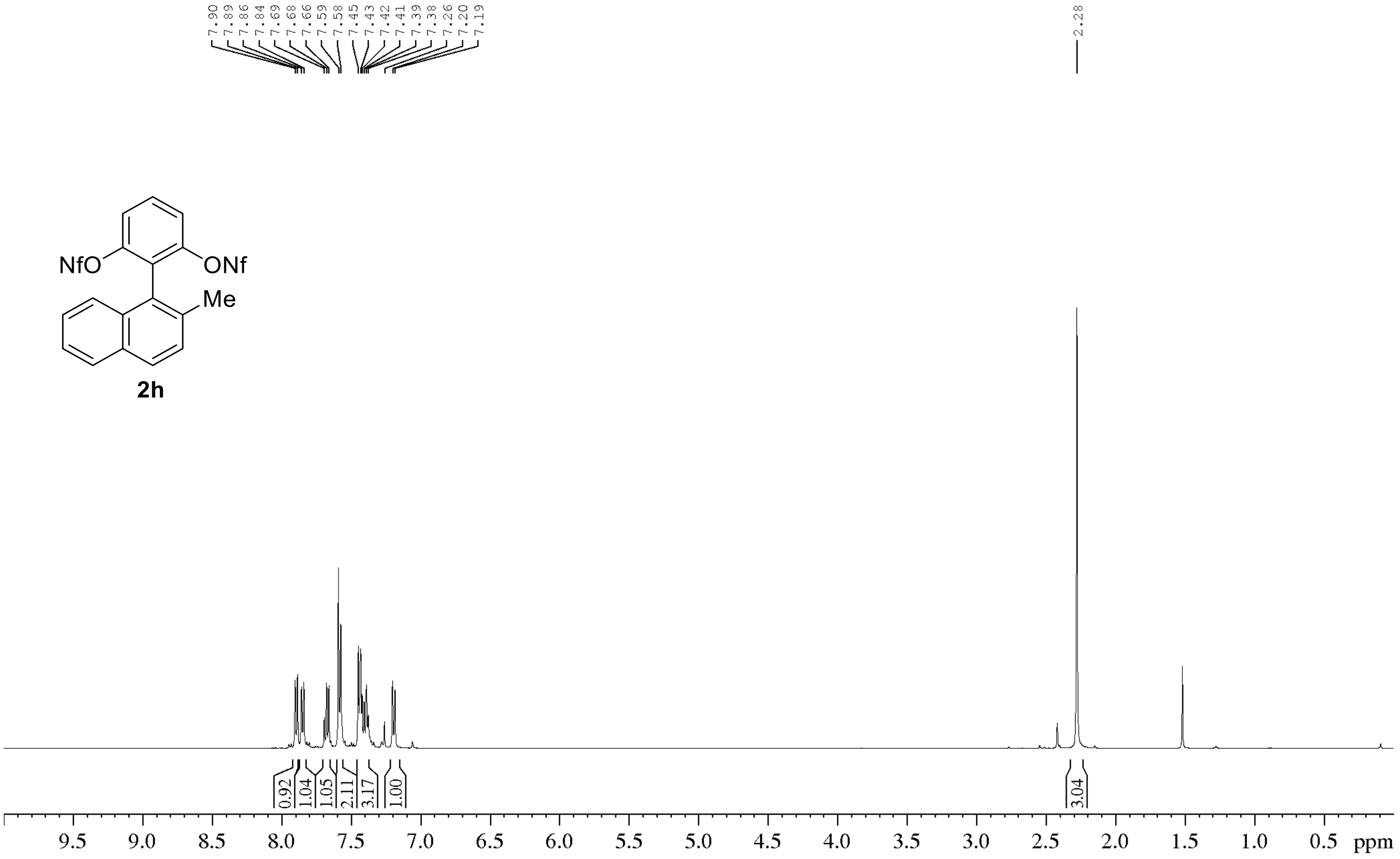

2h

Figure S86. ^{13}**C{^{1}H} NMR** (126 MHz, $CDCl_3$, 298 K) of **2h**

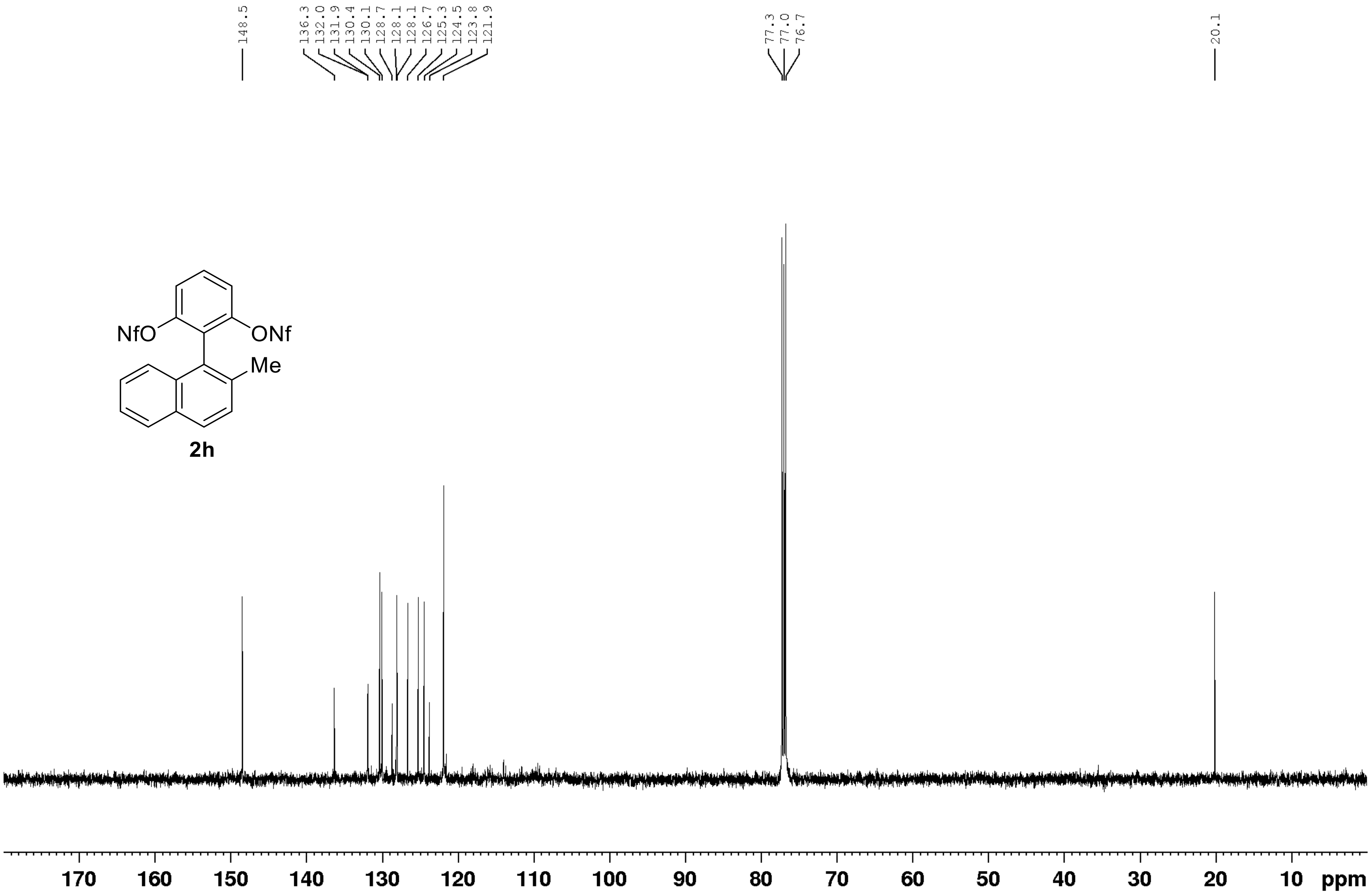

Figure S87. **^{19}F NMR** (471 MHz, $CDCl_3$, 298 K) of **2h**

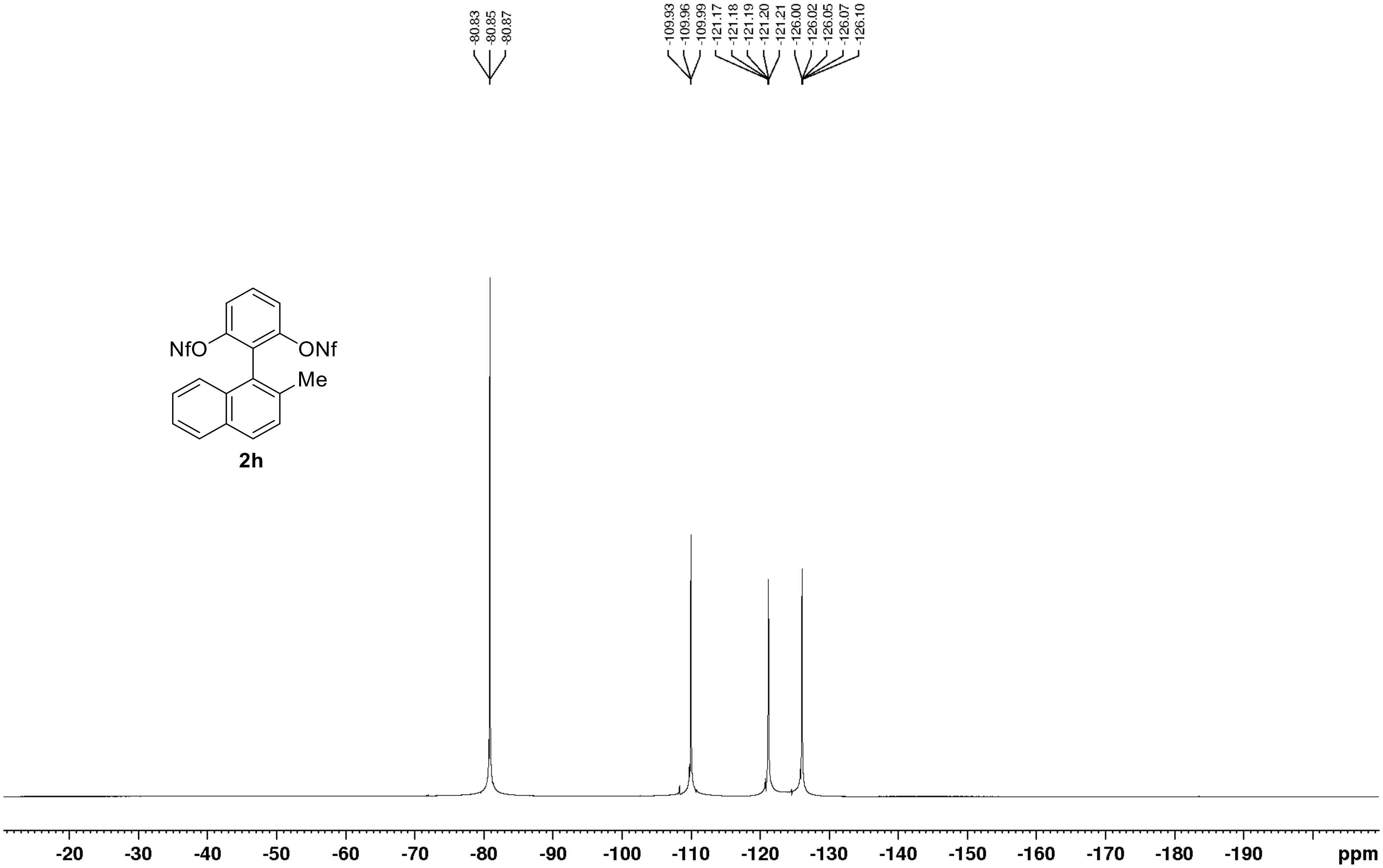

2-(8-methylnaphthalen-1-yl)-1,3-phenylene bis(1,1,2,2,3,3,4,4,4-nonafluorobutane-1-sulfonate) (2i)

Figure S88. **^{1}H NMR** (500 MHz, $CDCl_3$, 298 K) of **2i**

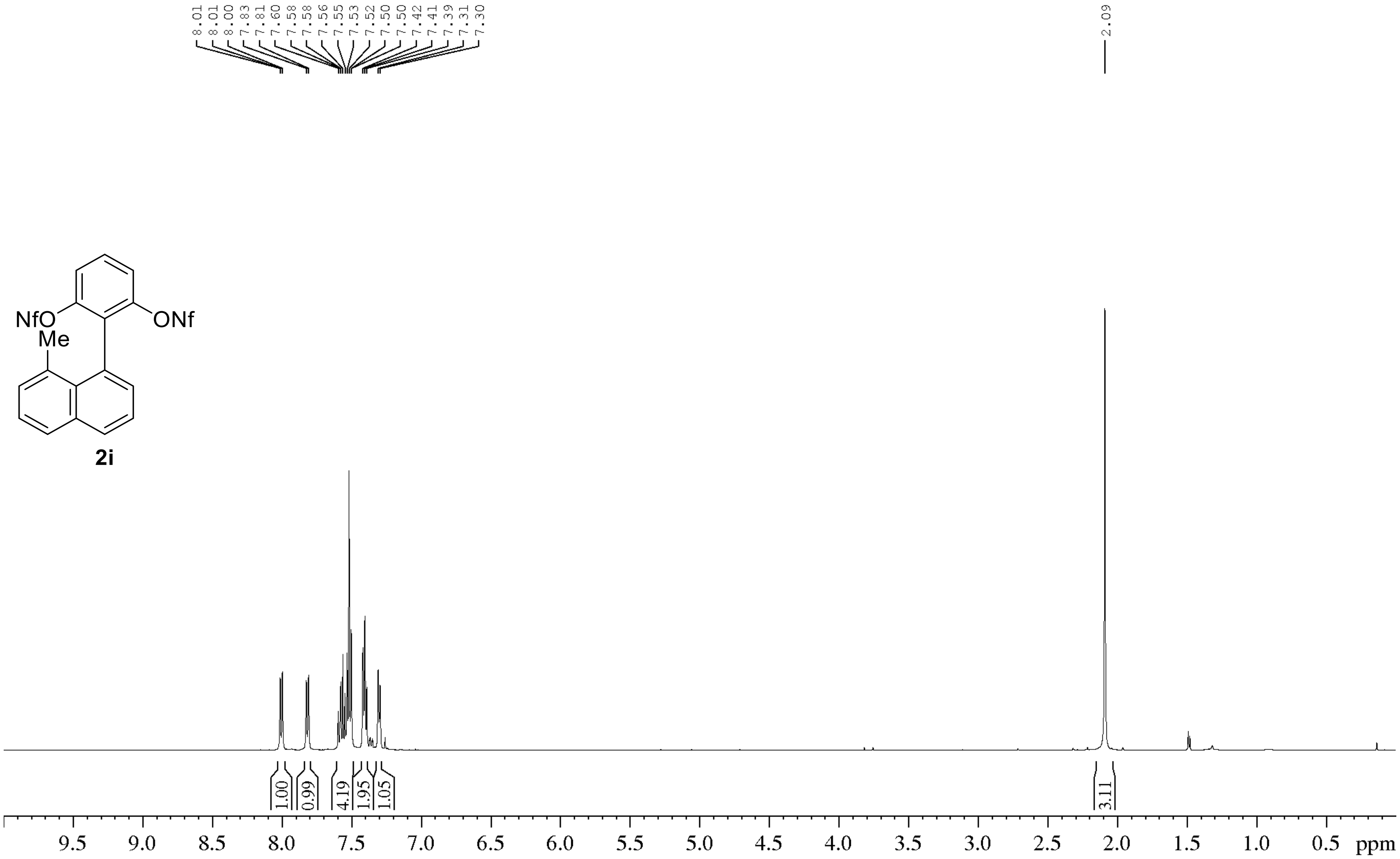

Figure S89. **^{13}C{^{1}H} NMR** (126 MHz, $CDCl_3$, 298 K) of **2i**

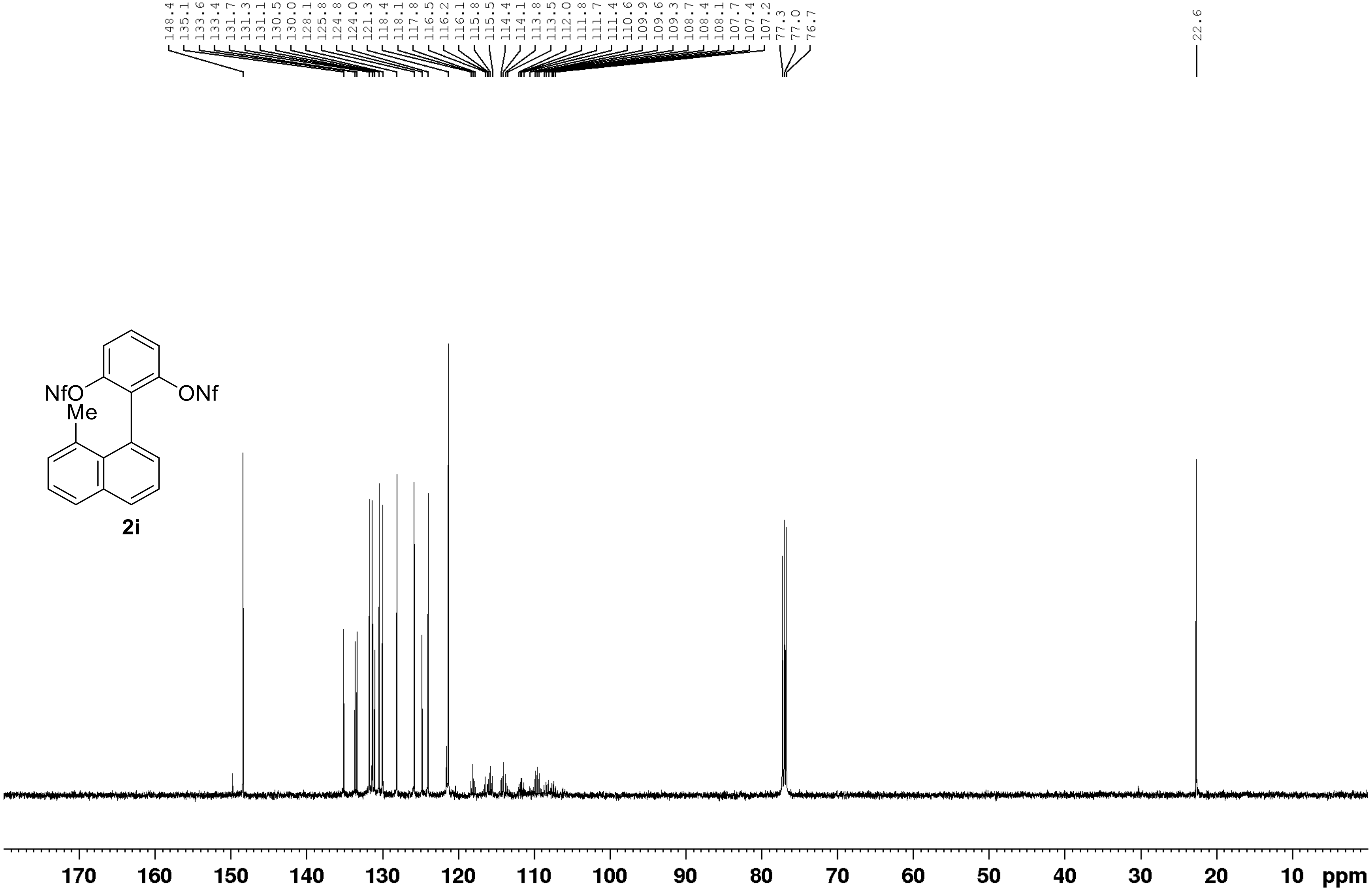

Figure S90. **^{19}F NMR** (471 MHz, $CDCl_3$, 298 K) of **2i**

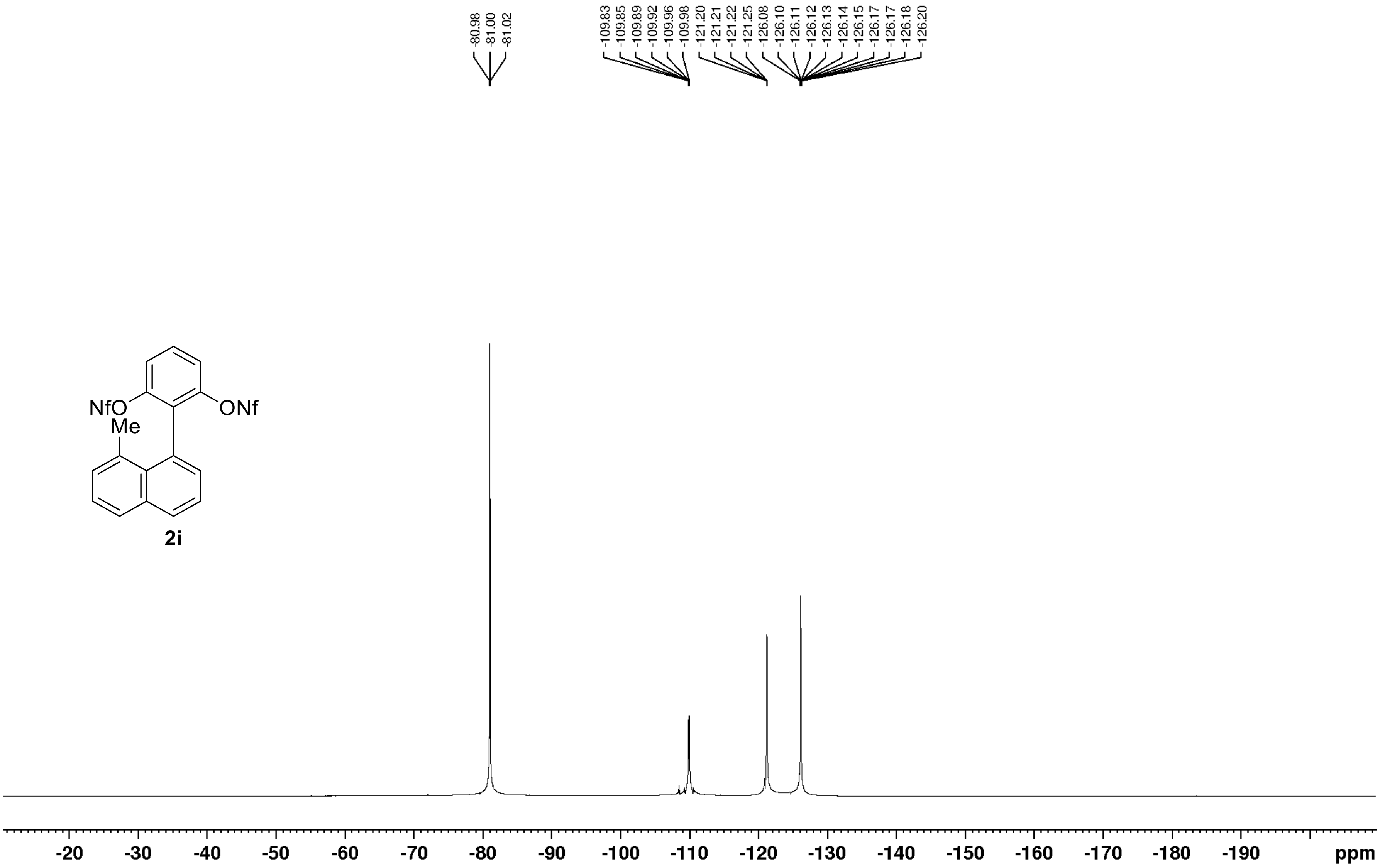

2-(1,2-dihydroacenaphthylen-5-yl)-1,3-phenylene bis(1,1,2,2,3,3,4,4,4-nonafluorobutane-1-sulfonate) (2j)

Figure S91. ^{1}**H NMR** (500 MHz, $CDCl_3$, 298 K) of **2j**

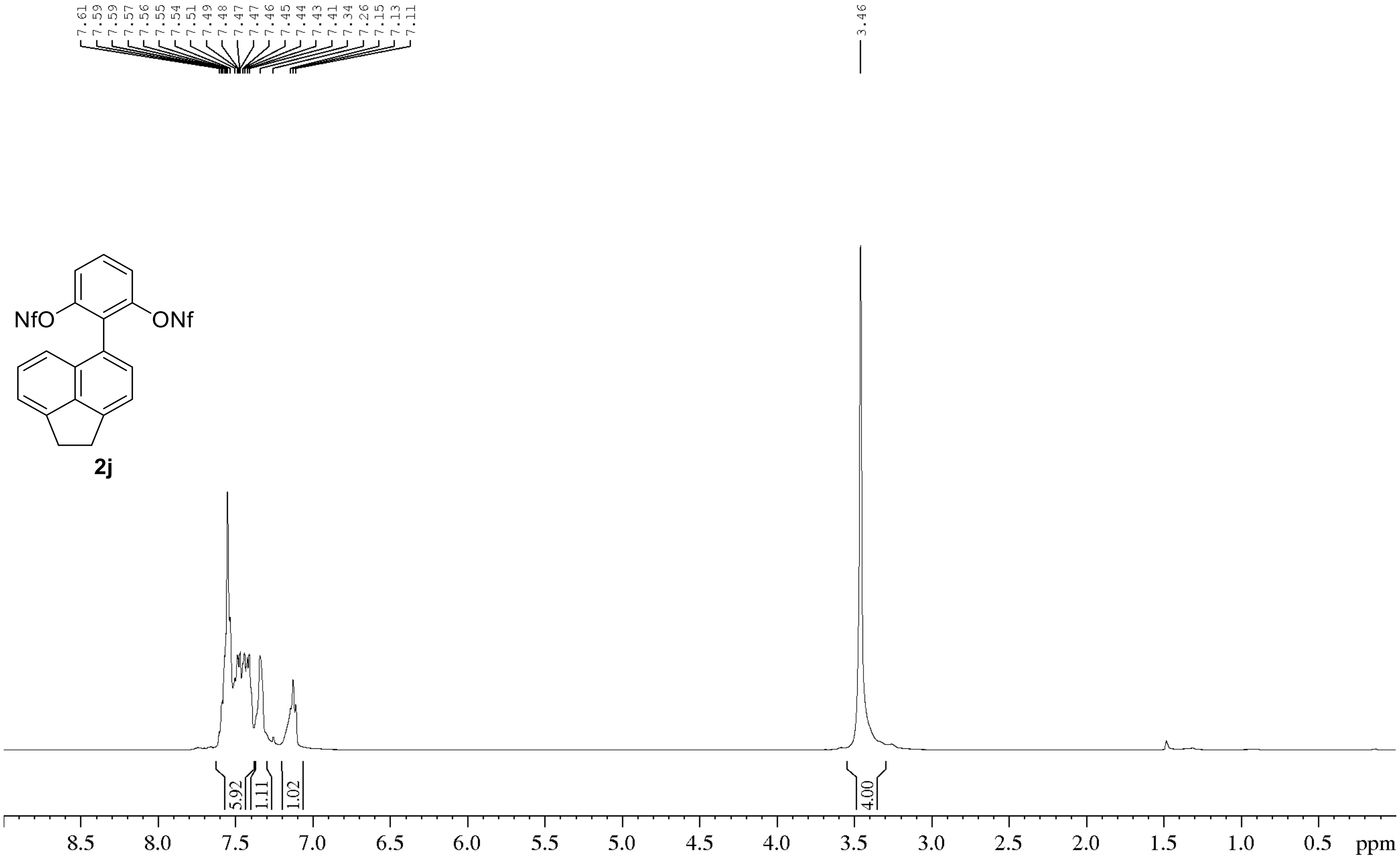

Figure S92. ^{13}C{^{1}H} NMR (126 MHz, $CDCl_3$, 298 K) of **2j**

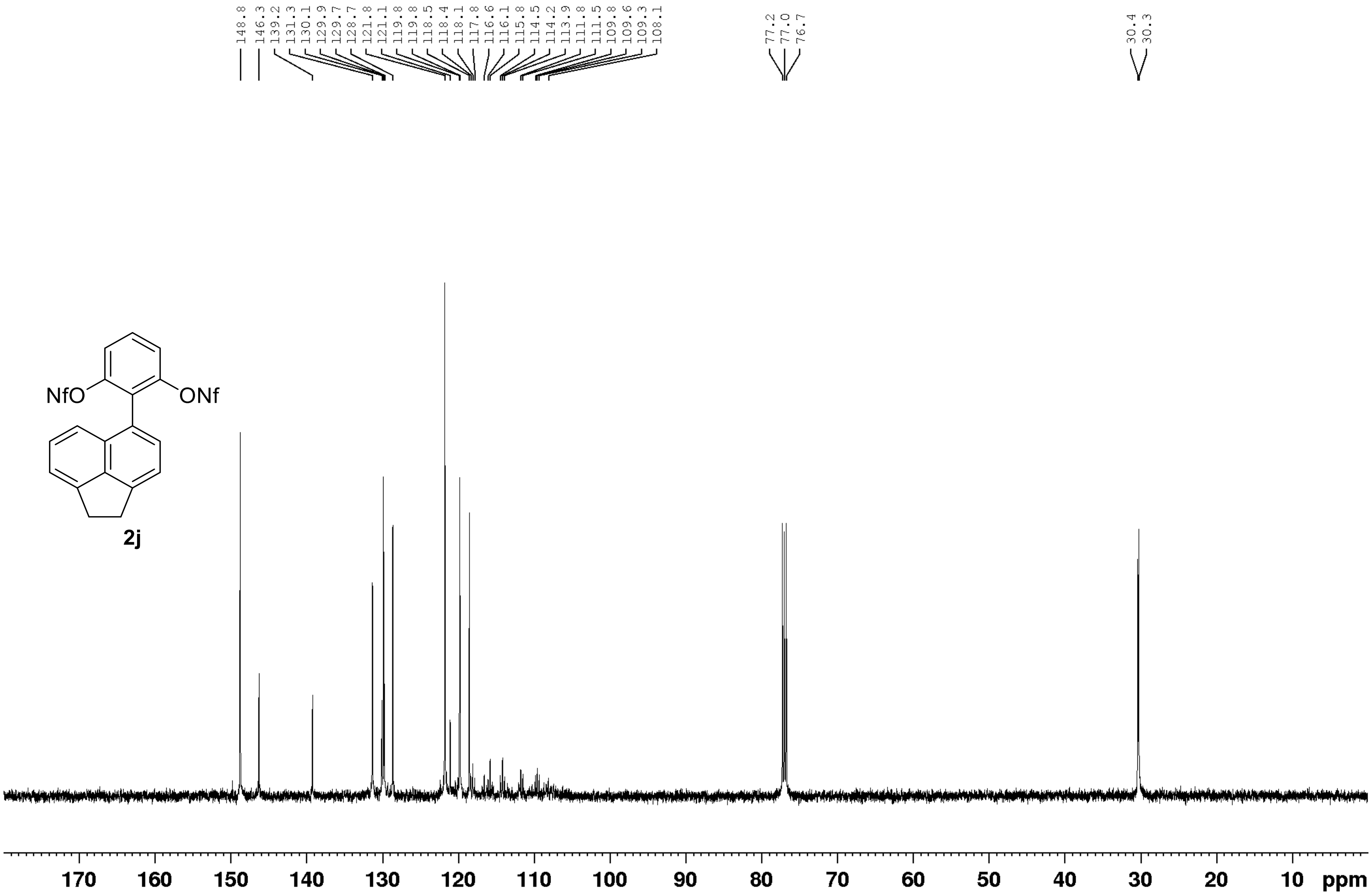

Figure S93. **^{19}F NMR** (471 MHz, $CDCl_3$, 298 K) of **2j**

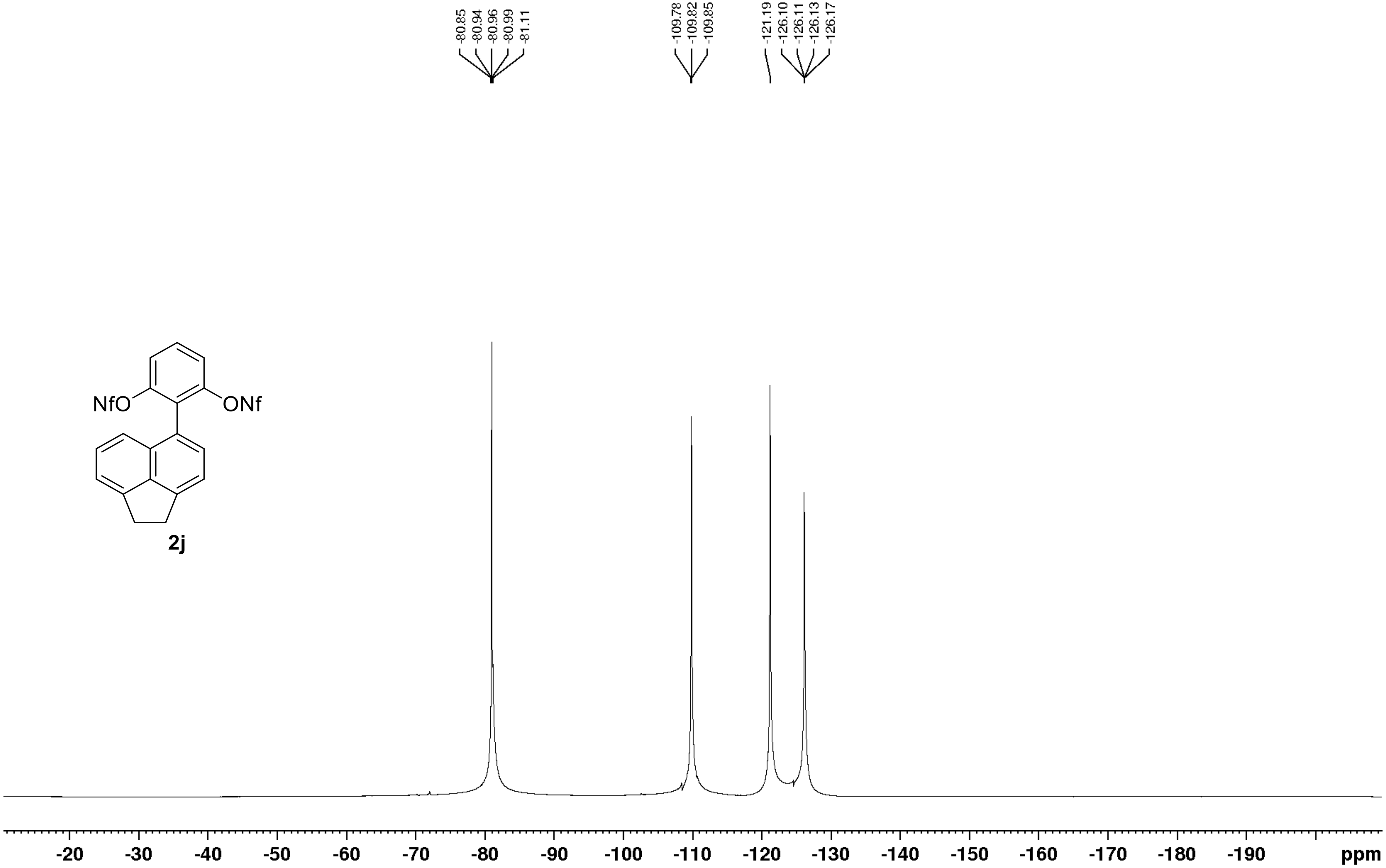

2-(fluoranthen-3-yl)-1,3-phenylene bis(1,1,2,2,3,3,4,4,4-nonafluorobutane-1-sulfonate) (2k)

Figure S94. **^{1}H NMR** (500 MHz, $CDCl_3$, 298 K) of **2k**

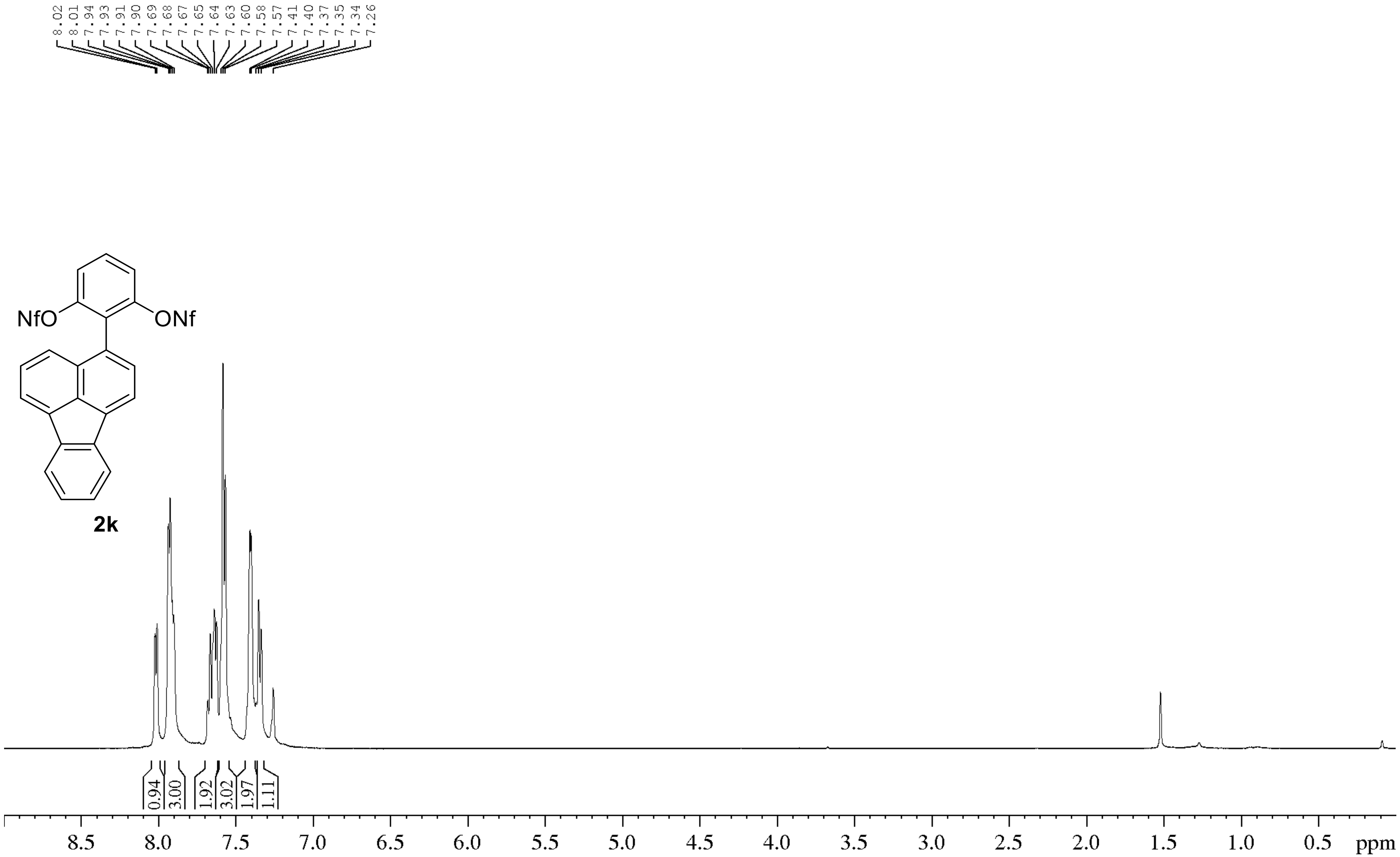

Figure S95. $^{13}C\{^1H\}$ NMR (126 MHz, $CDCl_3$, 298 K) of **2k**

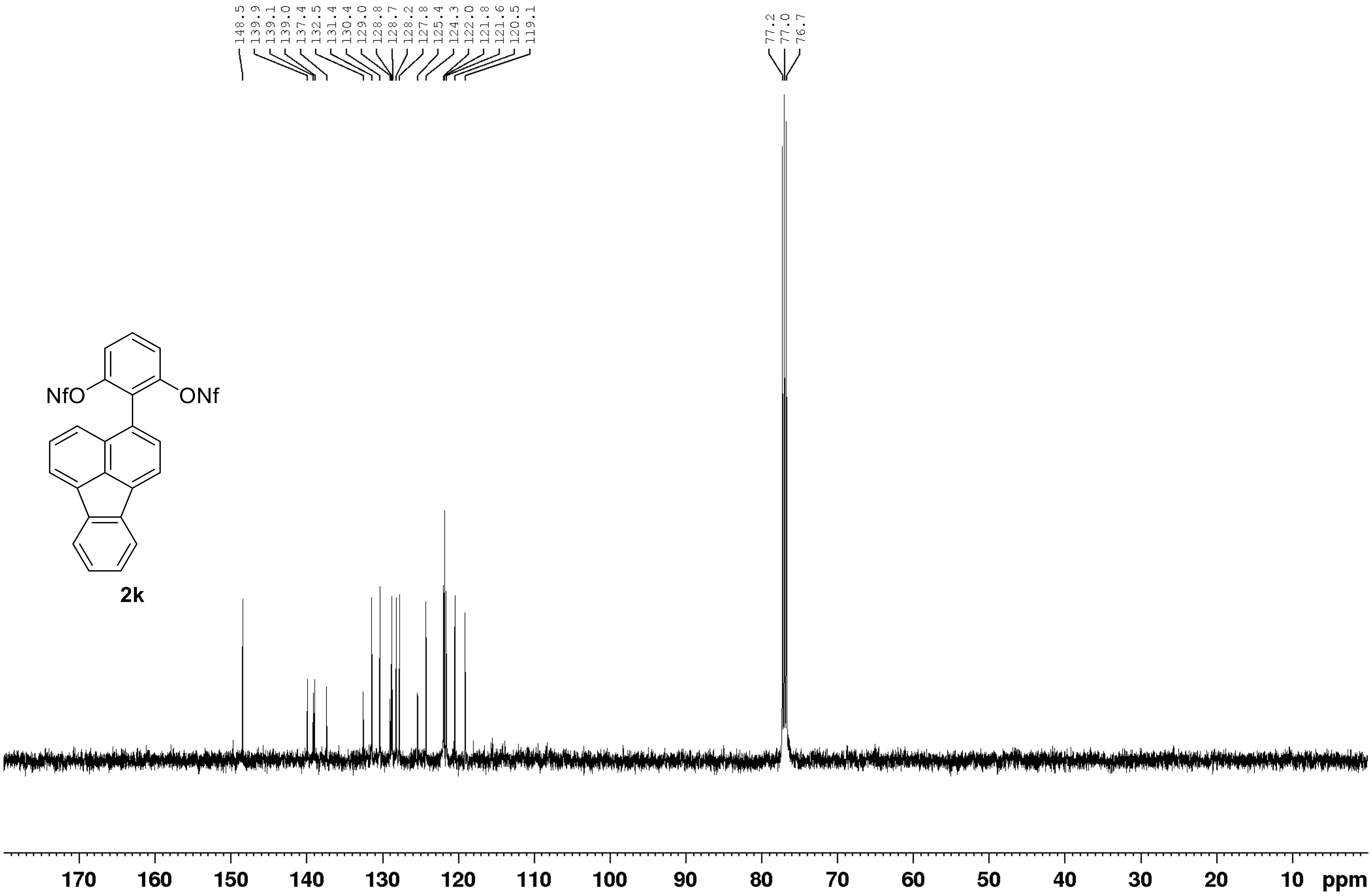

Figure S96. ^{19}F NMR (471 MHz, $CDCl_3$, 298 K) of **2k**

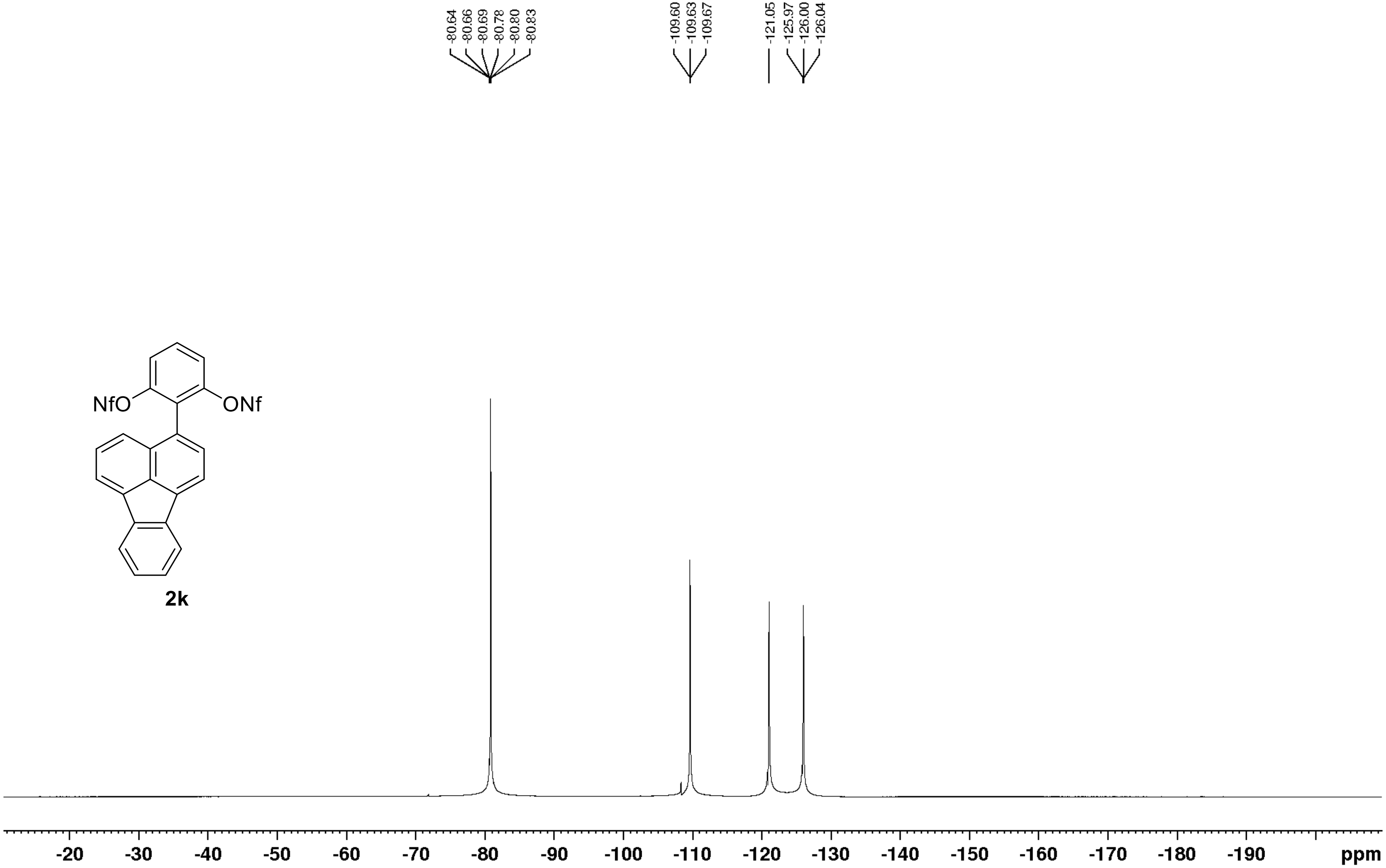

[1,1':2',1''-terphenyl]-2,6-diyl bis(1,1,2,2,3,3,4,4,4-nonafluorobutane-1-sulfonate) (2l)

Figure S97. ^{1}H NMR (500 MHz, $CDCl_3$, 298 K) of **2l**

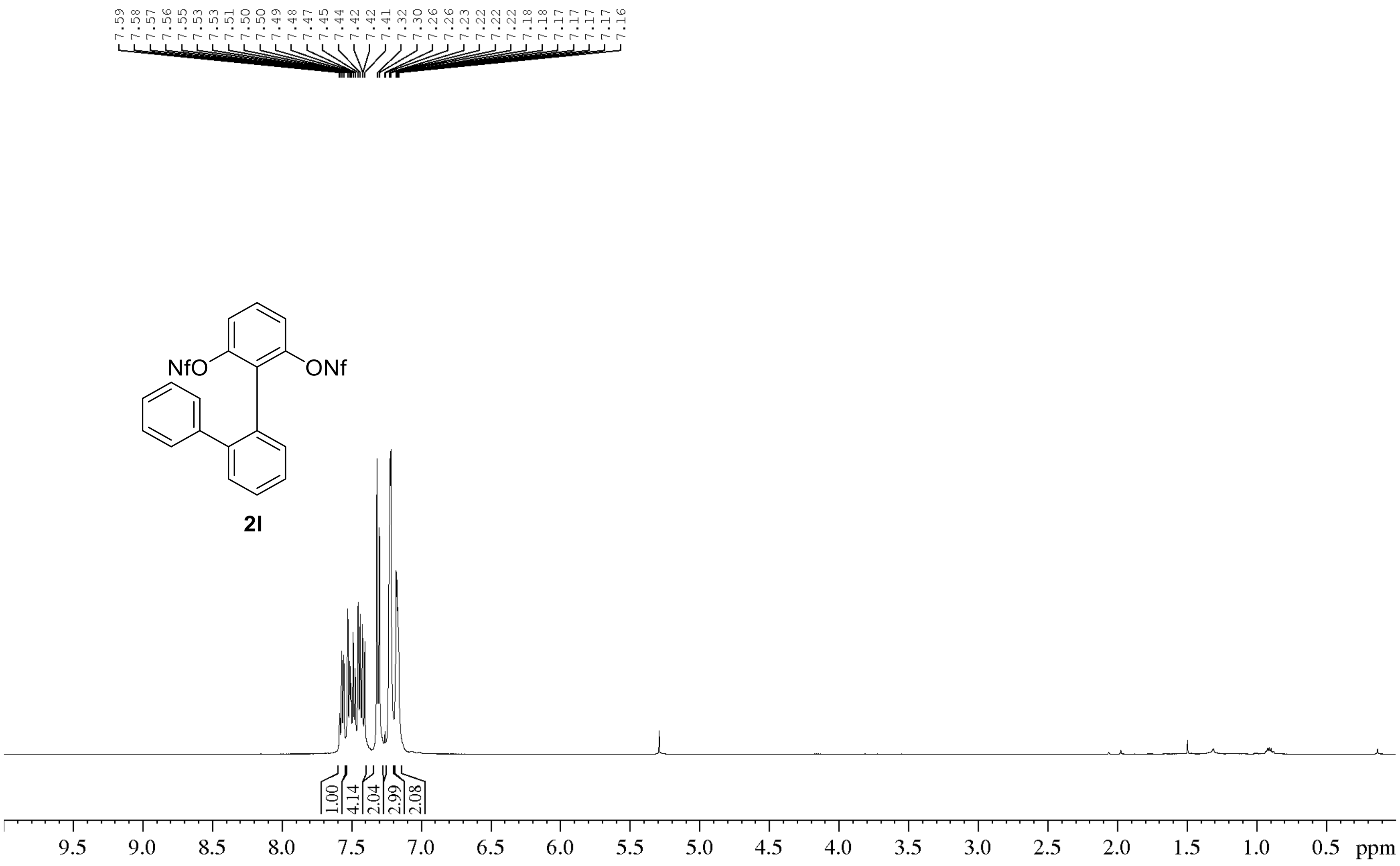

Figure S98. ^{13}C{^{1}H} NMR (126 MHz, $CDCl_3$, 298 K) of **2l**

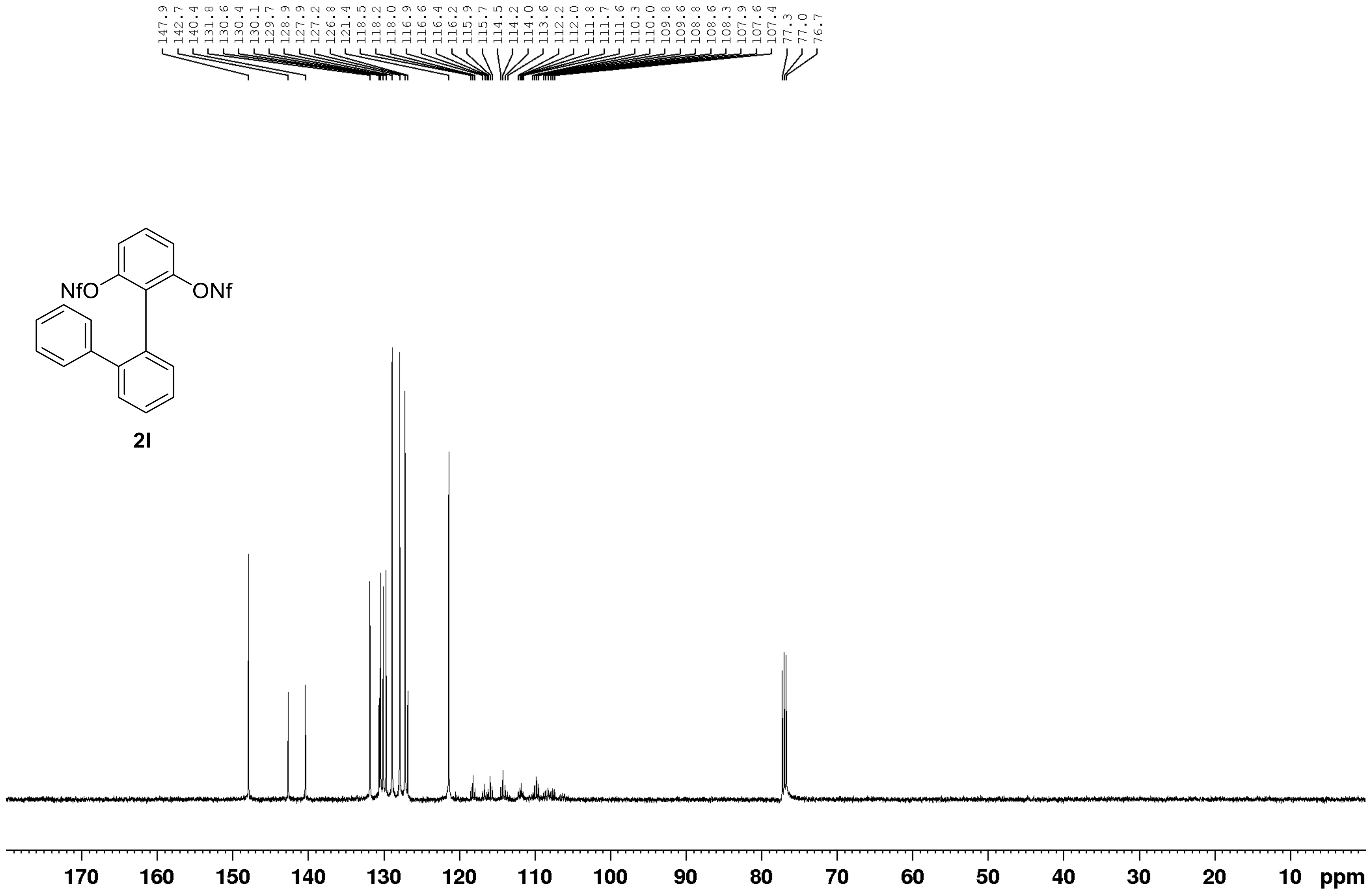

Figure S99. ^{19}**F NMR** (471 MHz, $CDCl_3$, 298 K) of **2l**

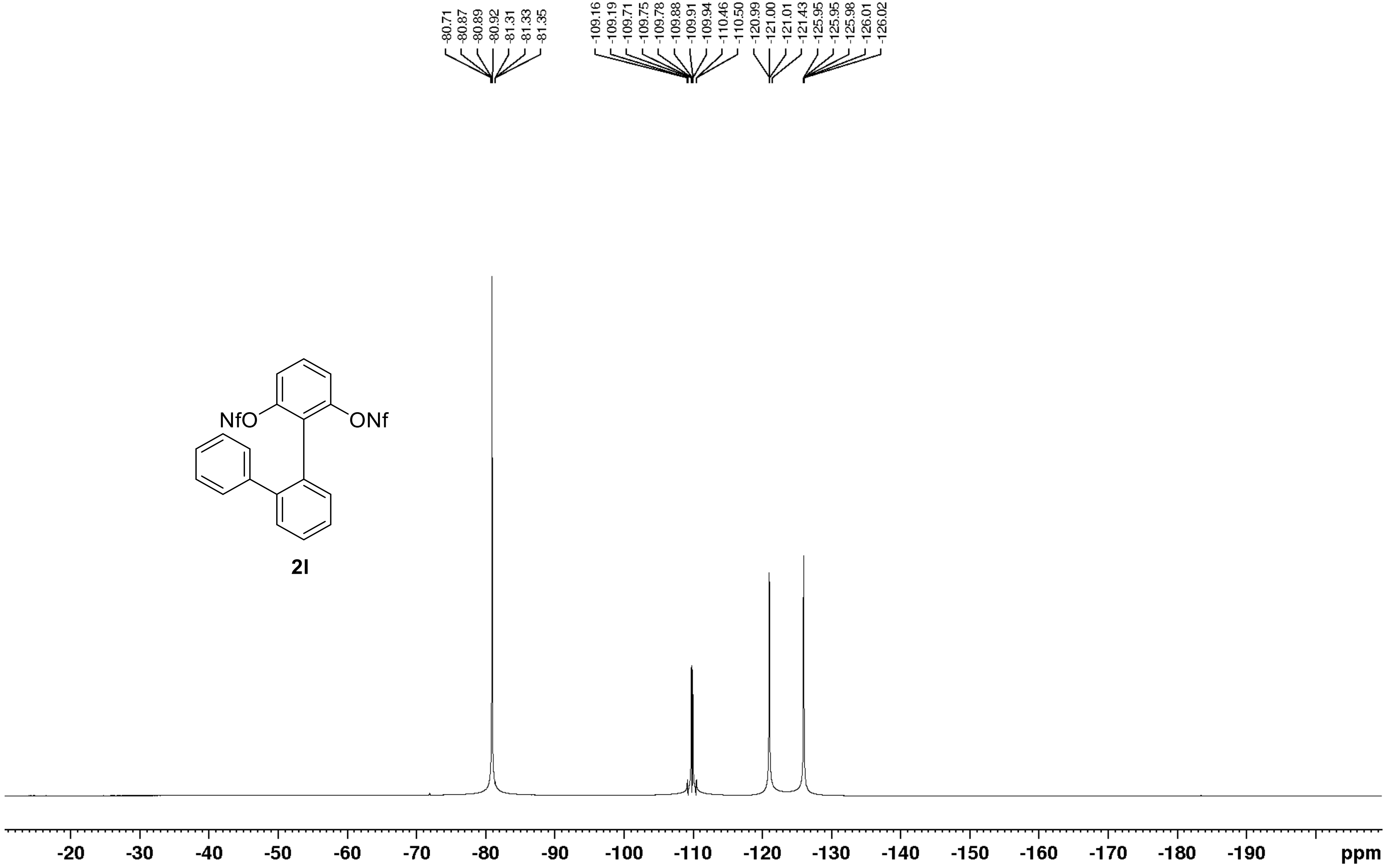

2''-fluoro-[1,1':2',1''-terphenyl]-2,6-diyl bis(1,1,2,2,3,3,4,4,4-nonafluorobutane-1-sulfonate) (2m)

Figure S100. **^{1}H NMR** (500 MHz, $CDCl_3$, 298 K) of **2m**

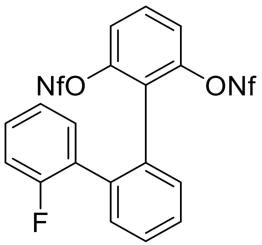

2m

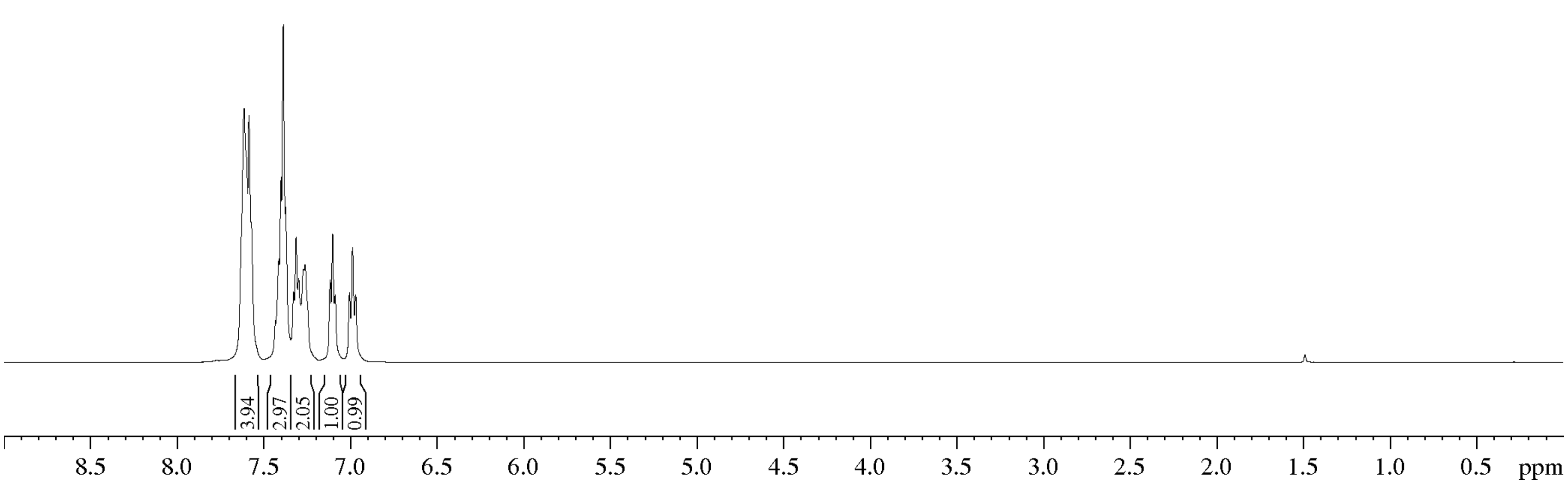

Figure S101. ^{13}C{^{1}H} NMR (126 MHz, $CDCl_3$, 298 K) of **2m**

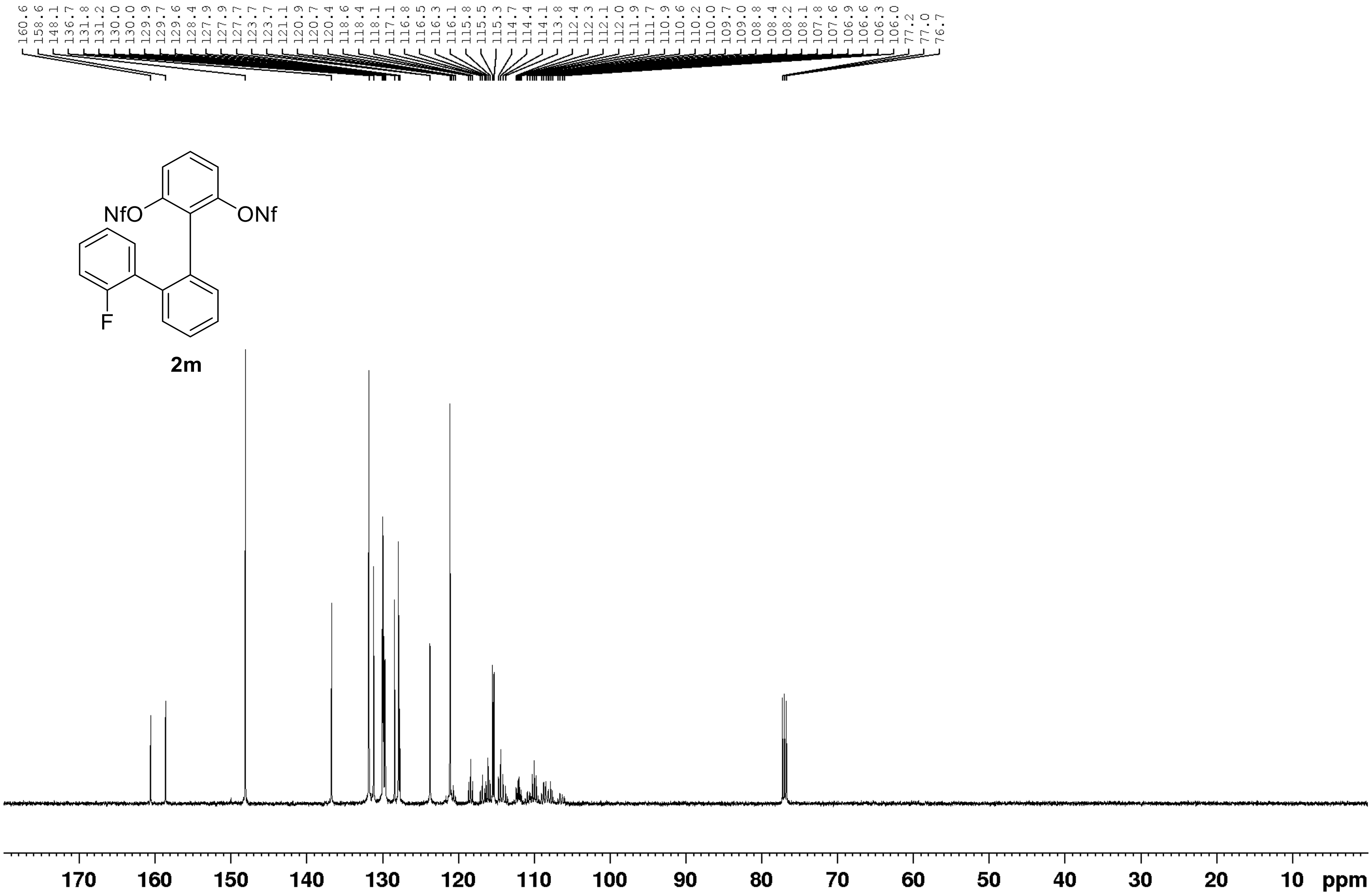

Figure S102. ^{19}F NMR (471 MHz, $CDCl_3$, 298 K) of **2m**

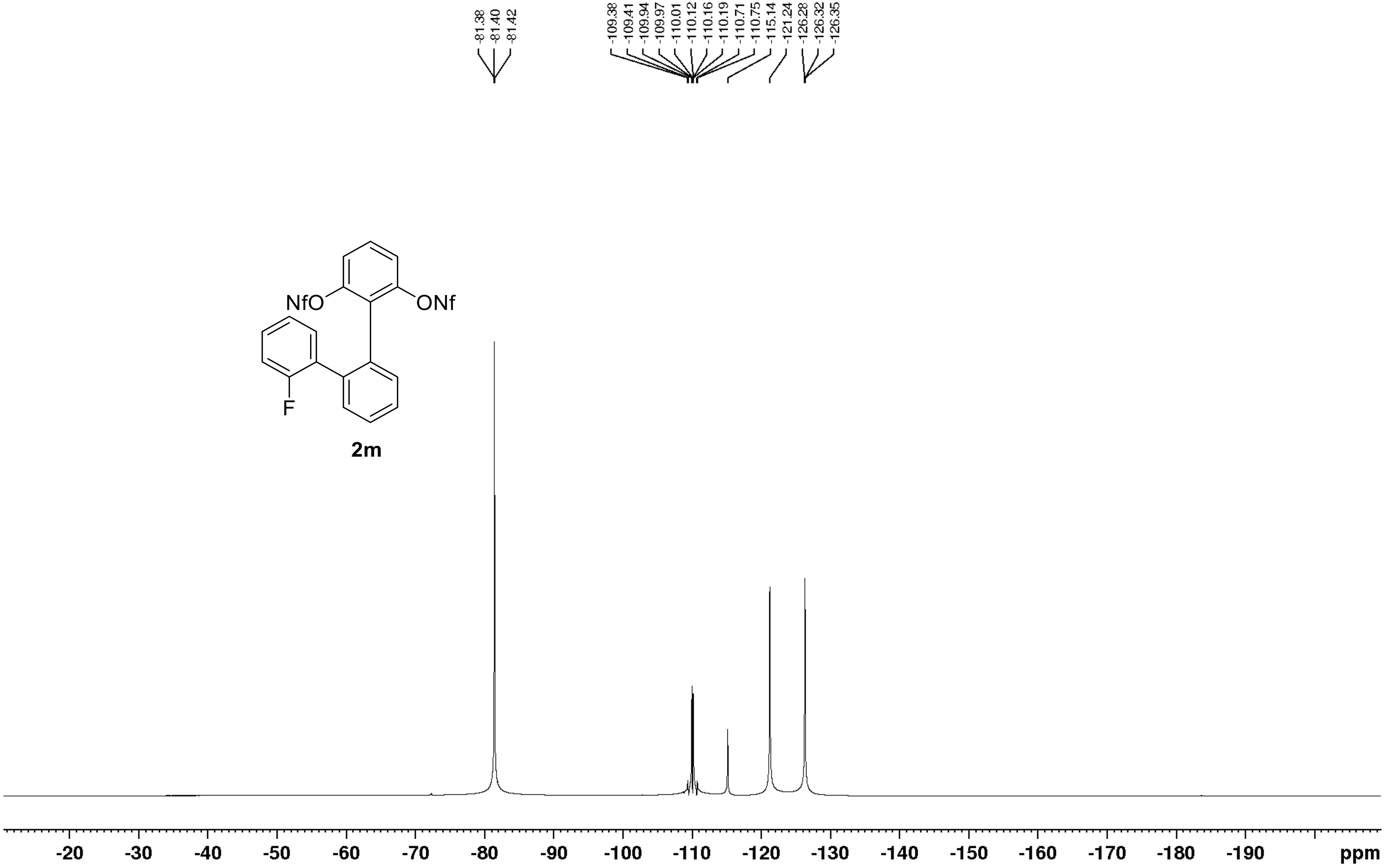

3''-chloro-[1,1':2',1''-terphenyl]-2,6-diyl bis(1,1,2,2,3,3,4,4,4-nonafluorobutane-1-sulfonate) (2n)

Figure S103. **^{1}H NMR** (500 MHz, $CDCl_3$, 298 K) of **2n**

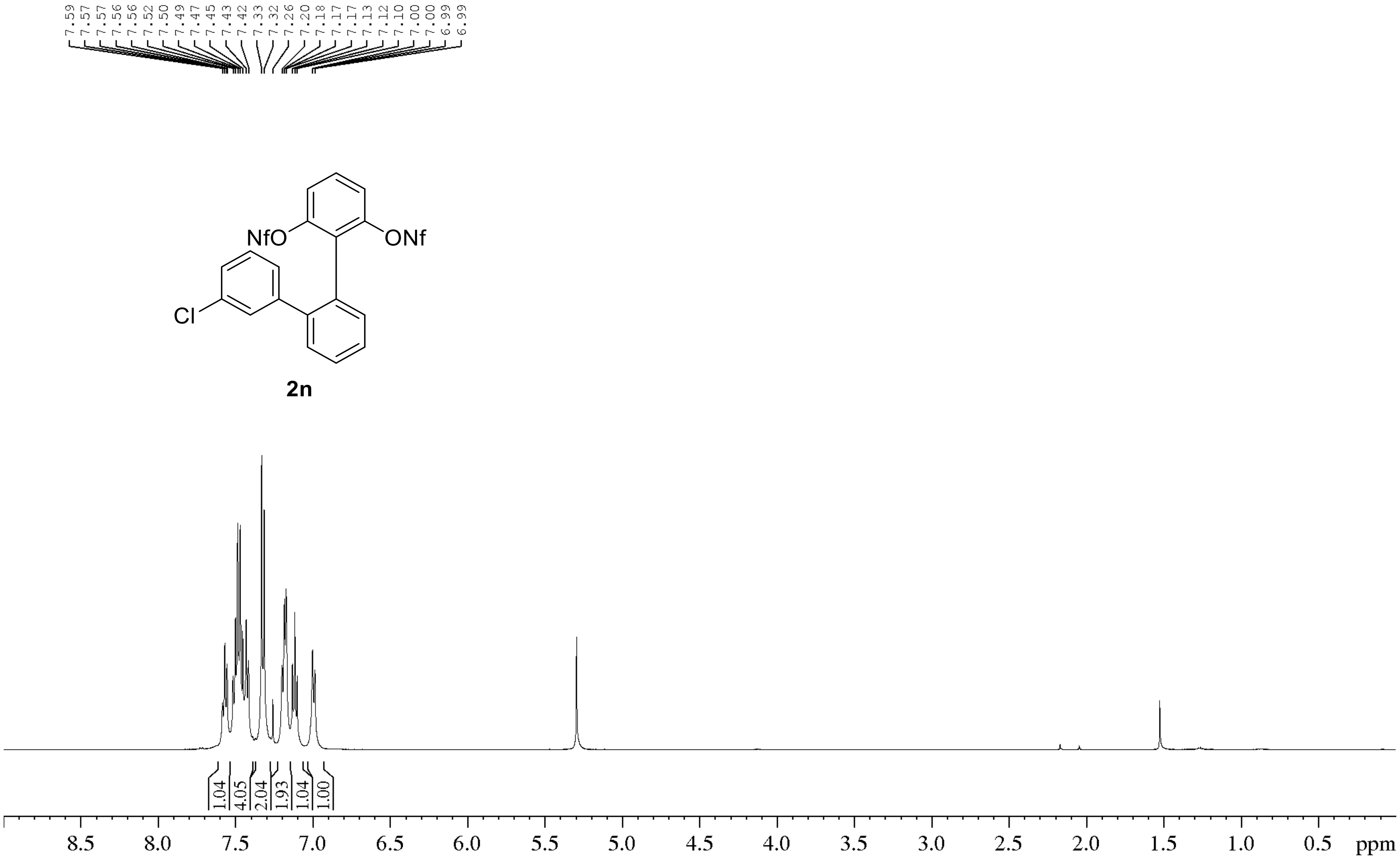

Figure S104. $^{13}C\{^{1}H\}$ NMR (126 MHz, $CDCl_3$, 298 K) of **2n**

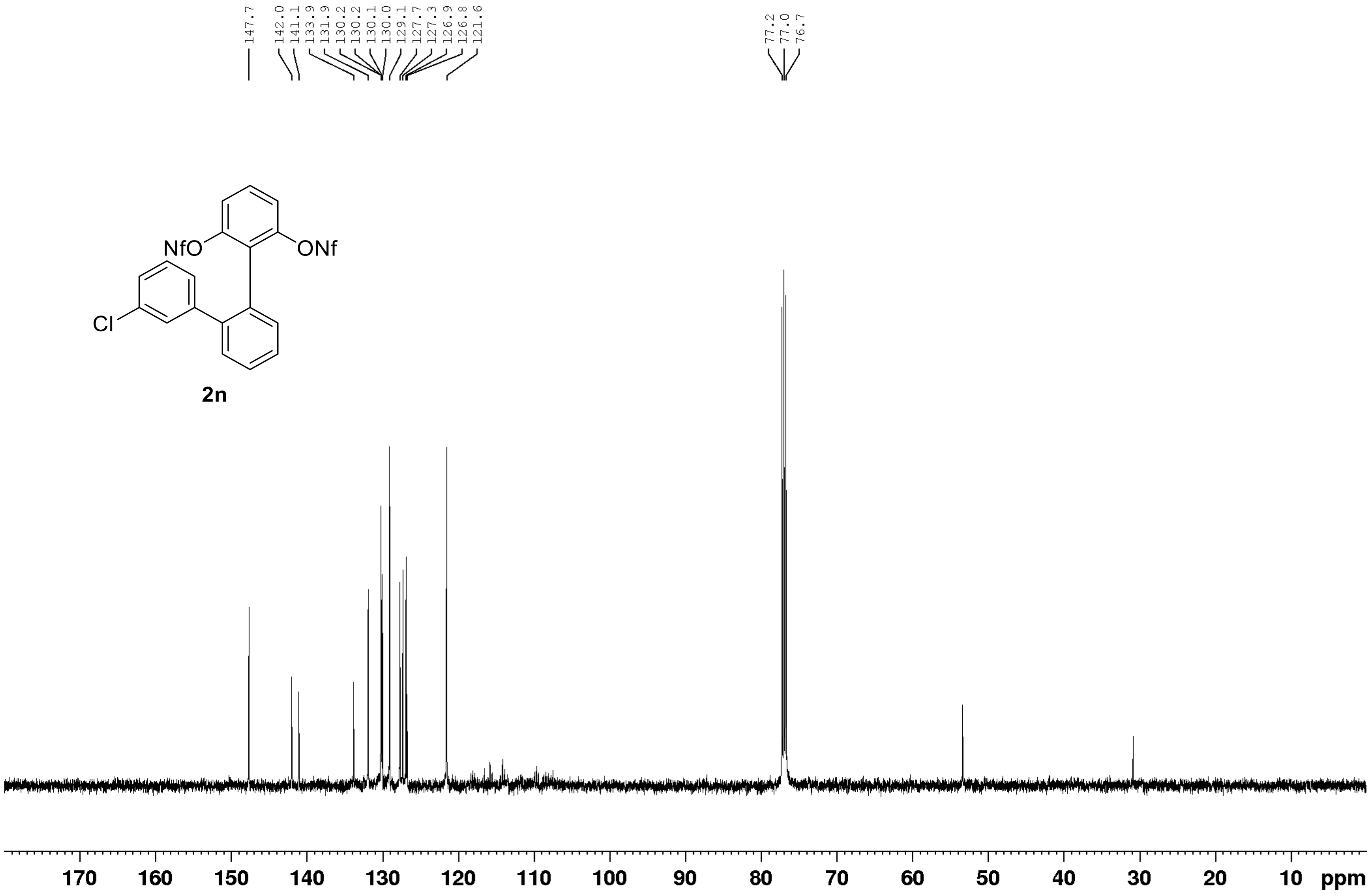

Figure S105. **^{19}F NMR** (471 MHz, $CDCl_3$, 298 K) of **2n**

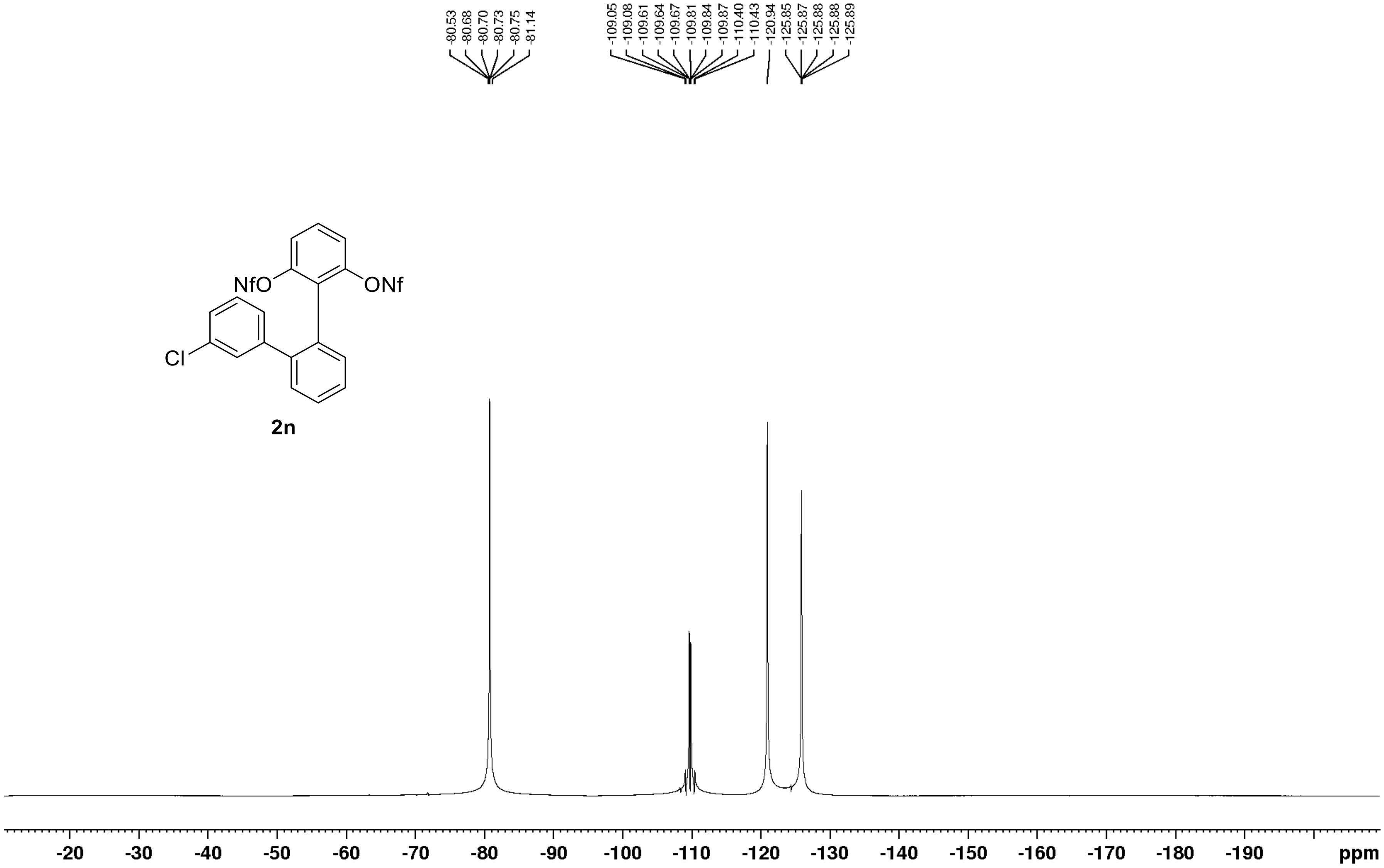

4''-(*tert*-butyl)-[1,1':2',1''-terphenyl]-2,6-diyl bis(1,1,2,2,3,3,4,4,4-nonafluorobutane-1-sulfonate) (2o)

Figure S106. ^{1}**H NMR** (500 MHz, $CDCl_3$, 298 K) of **2o**

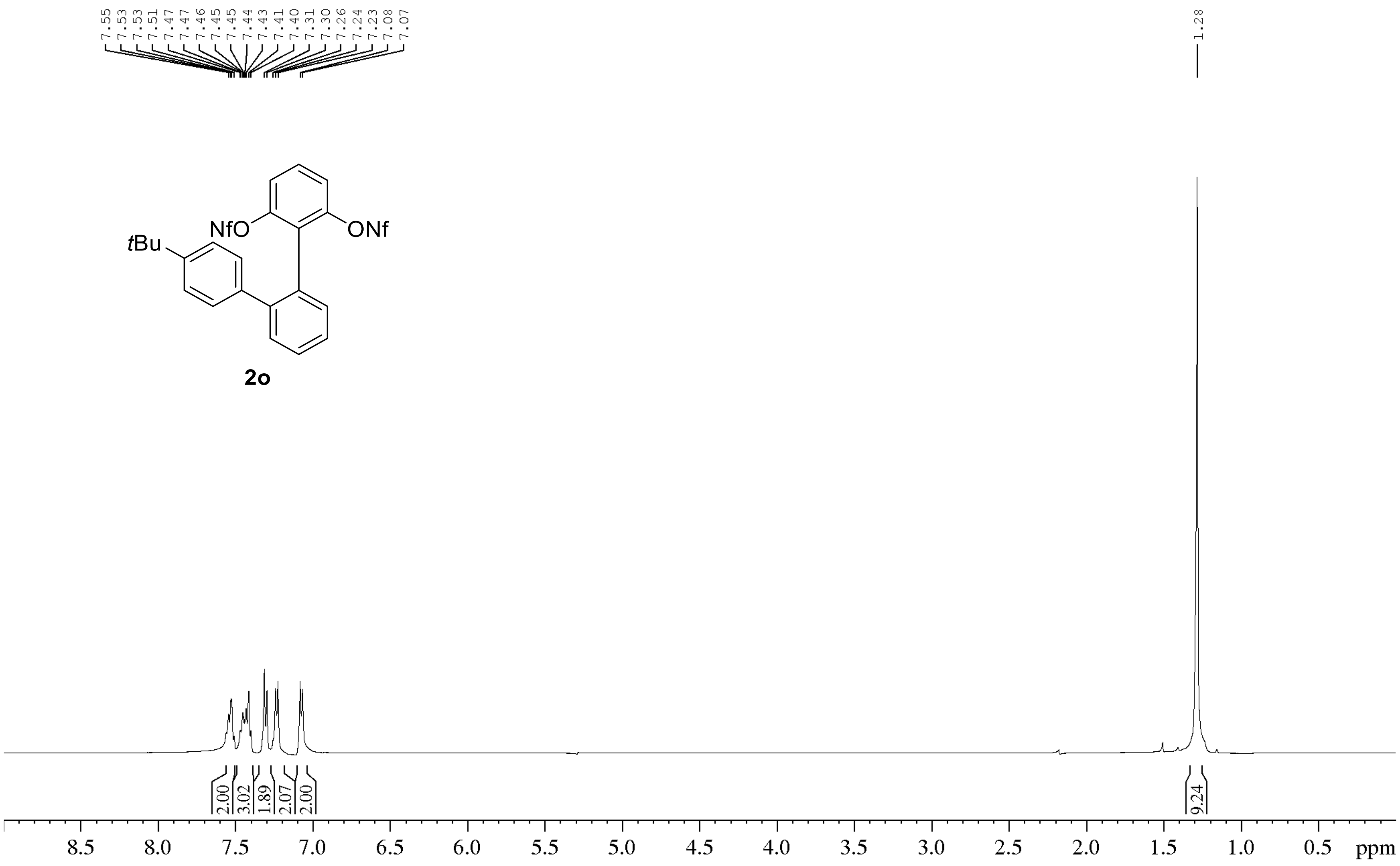

Figure S107. $^{13}C\{^1H\}$ NMR (126 MHz, $CDCl_3$, 298 K) of **2o**

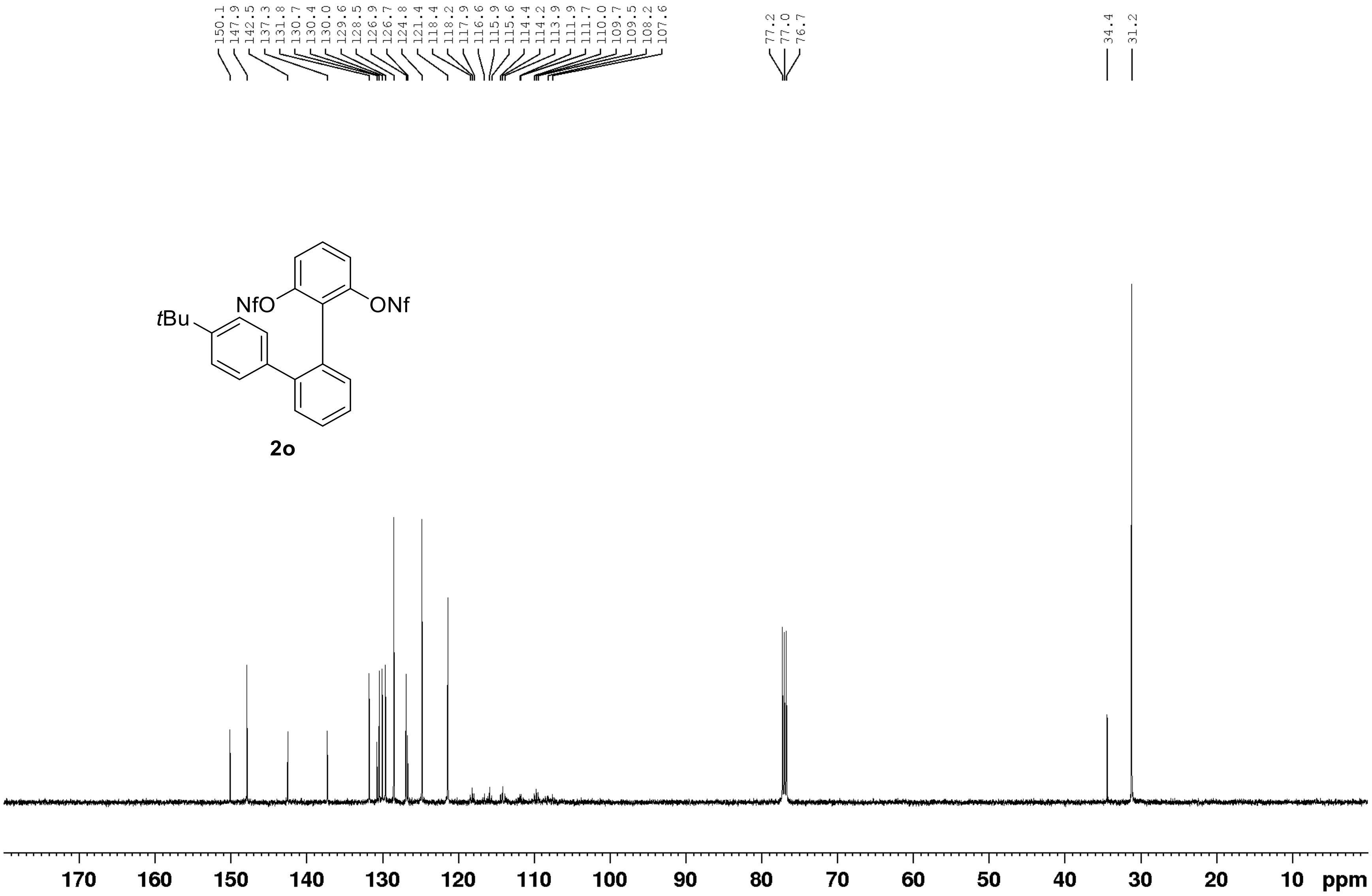

Figure S108. ^{19}**F NMR** (471 MHz, $CDCl_3$, 298 K) of **2o**

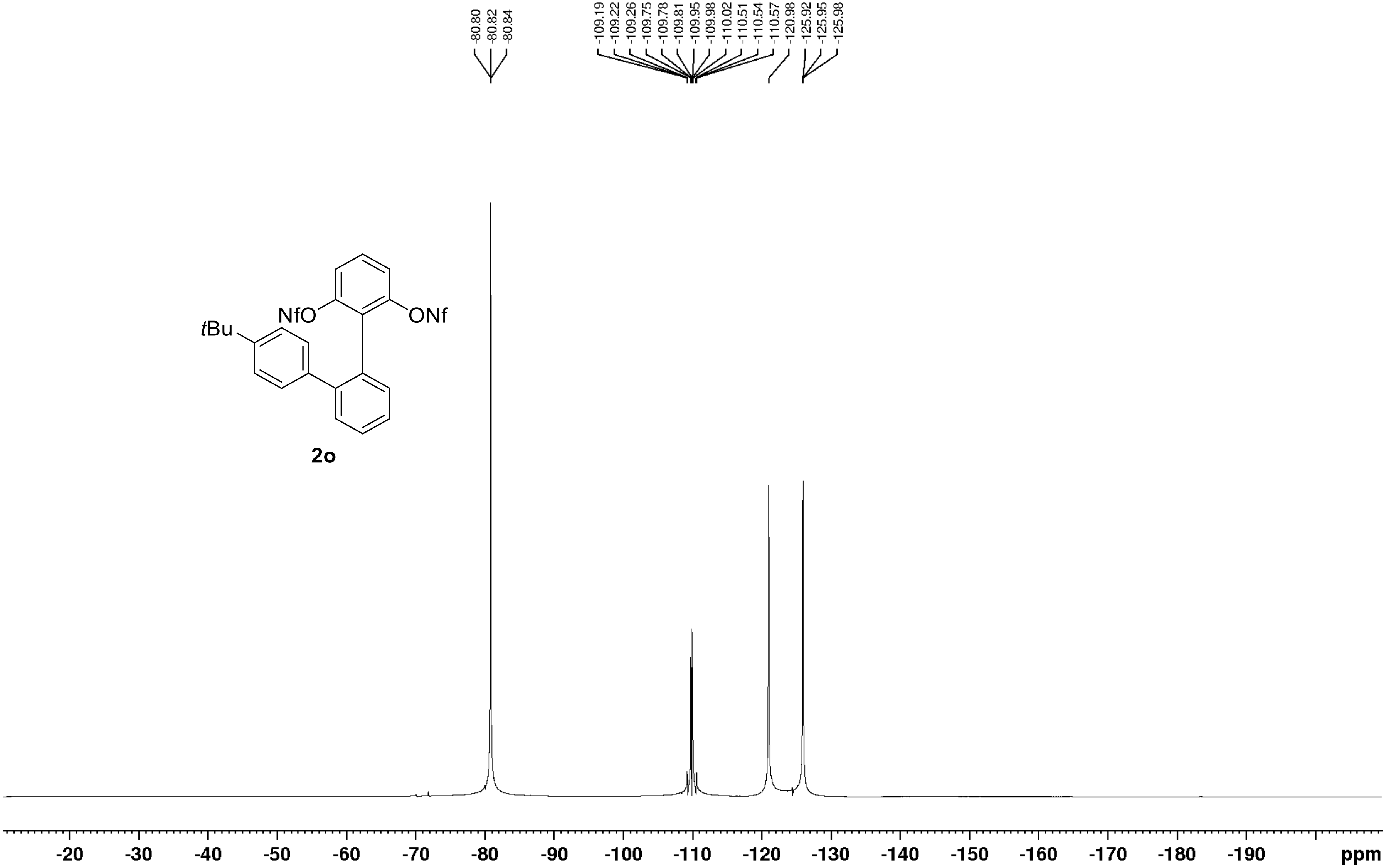

4''-acetyl-[1,1':2',1''-terphenyl]-2,6-diyl bis(1,1,2,2,3,3,4,4,4-nonafluorobutane-1-sulfonate) (2p)

Figure S109. **^{1}H NMR** (500 MHz, $CDCl_3$, 298 K) of **2p**

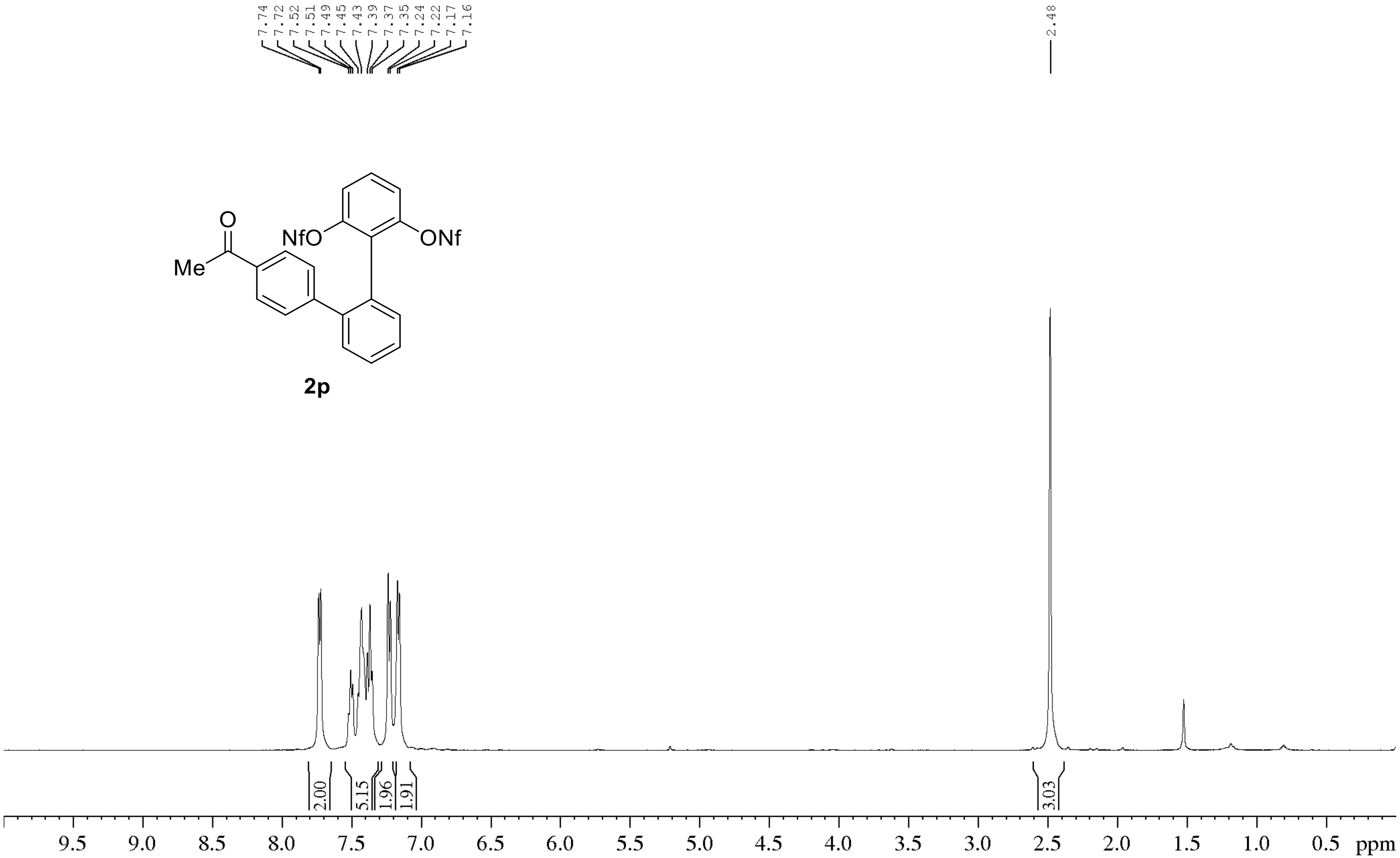

Figure S110. $^{13}C\{^{1}H\}$ NMR (126 MHz, $CDCl_3$, 298 K) of **2p**

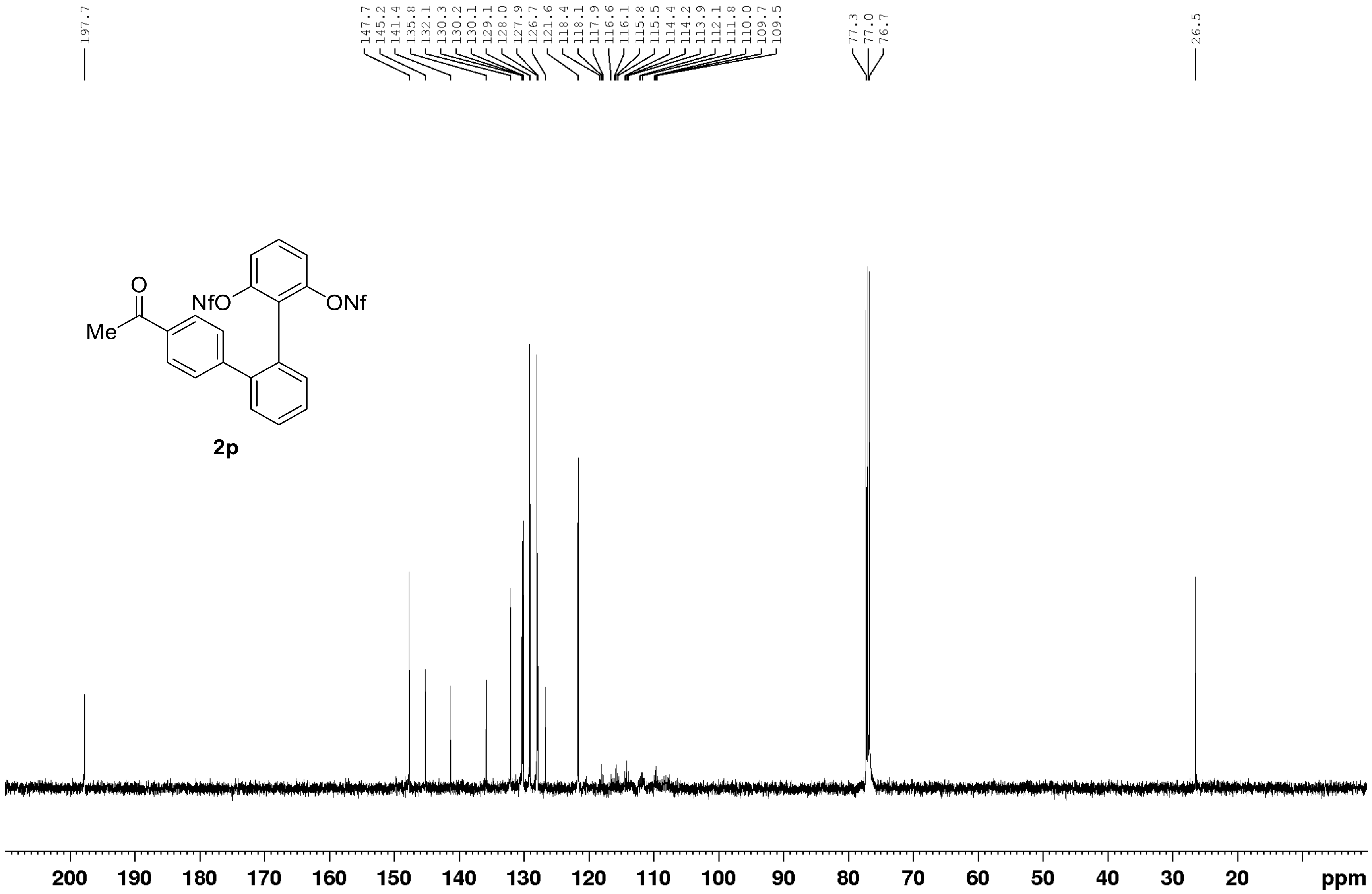

Figure S111. ^{19}F NMR (471 MHz, $CDCl_3$, 298 K) of **2p**

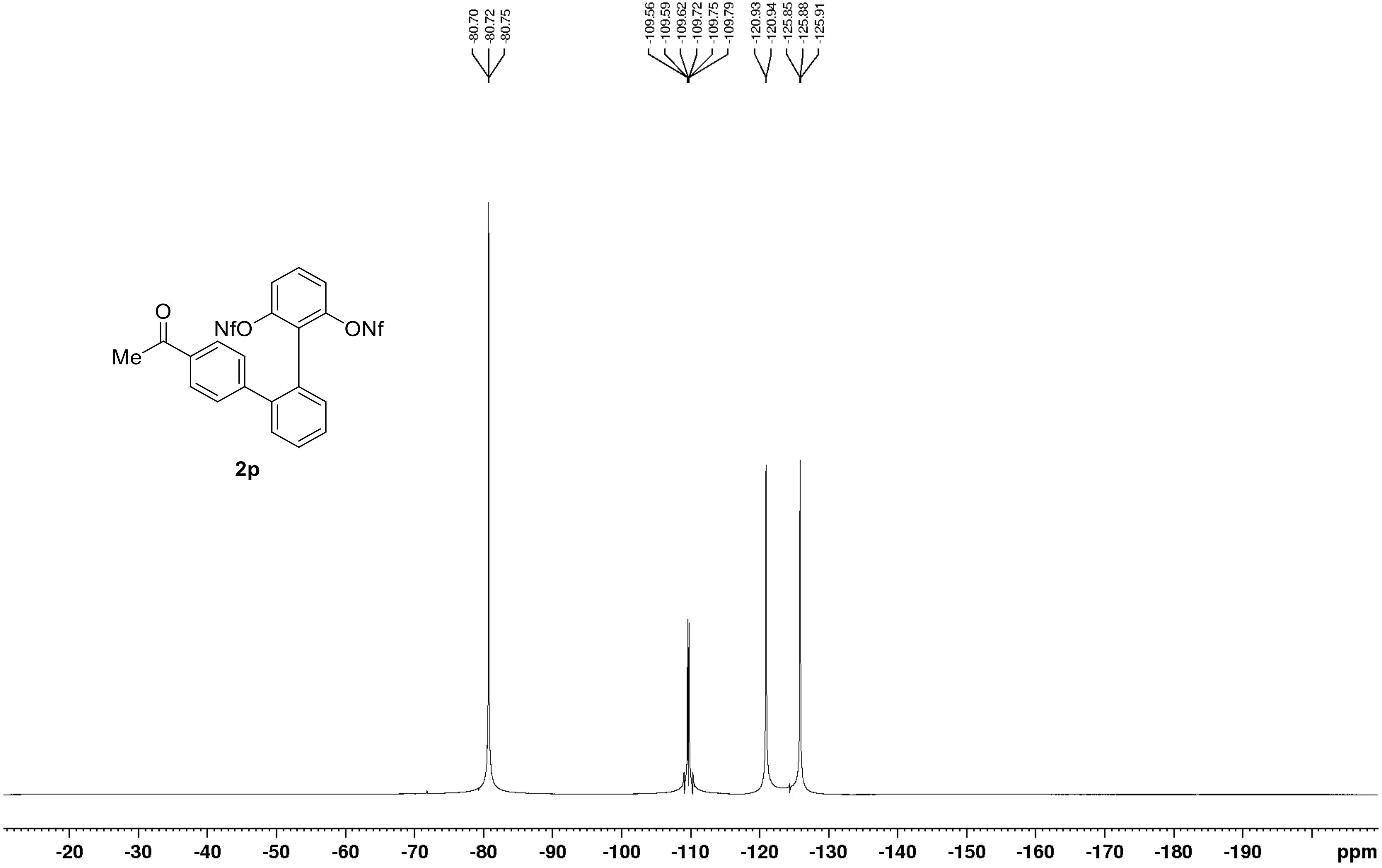

4''-nitro-[1,1':2',1''-terphenyl]-2,6-diyl bis(1,1,2,2,3,3,4,4,4-nonafluorobutane-1-sulfonate) (2q)

Figure S112. ^{1}**H NMR** (500 MHz, $CDCl_3$, 298 K) of **2q**

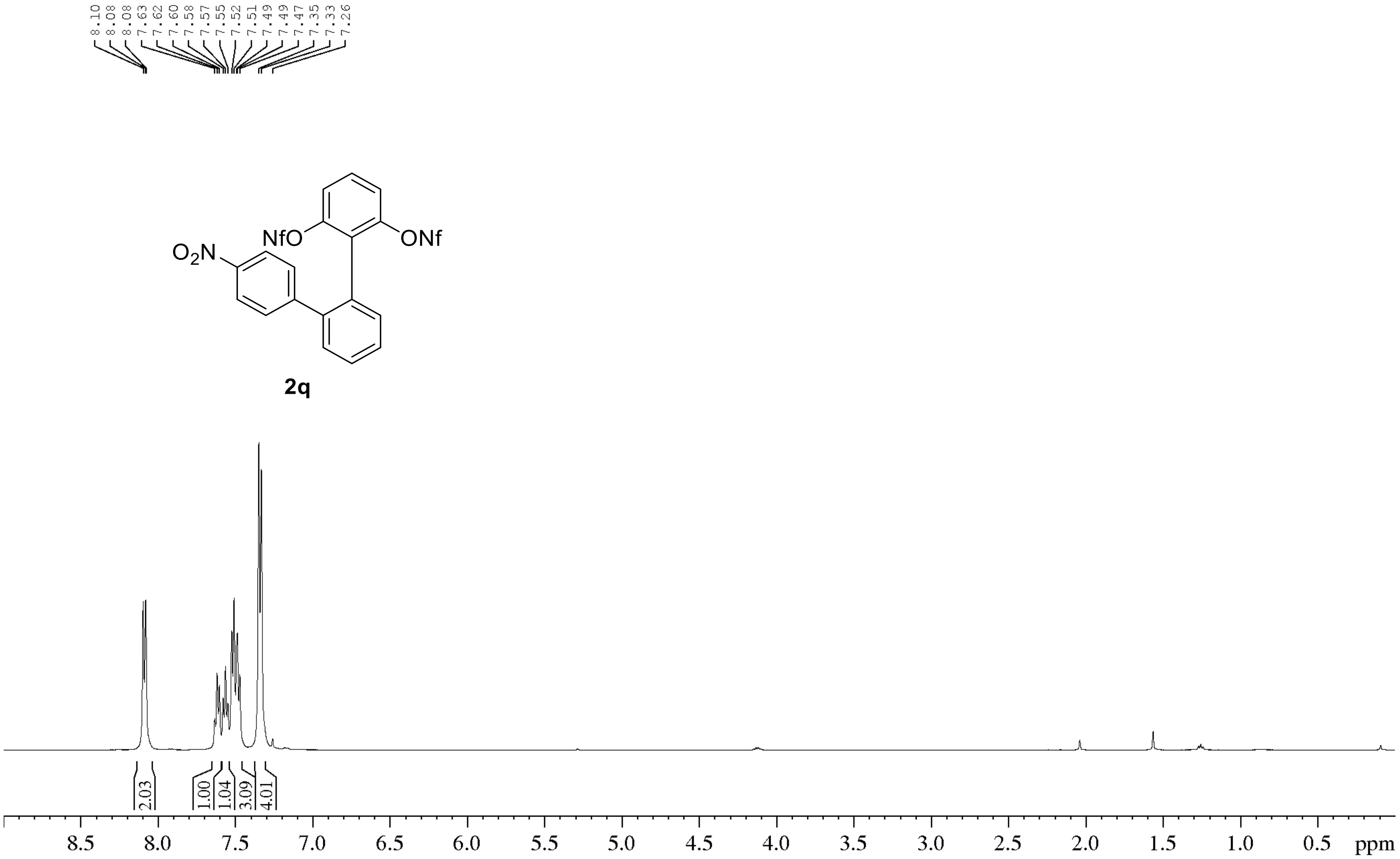

Figure S113. $^{13}C\{^1H\}$ NMR (126 MHz, $CDCl_3$, 298 K) of **2q**

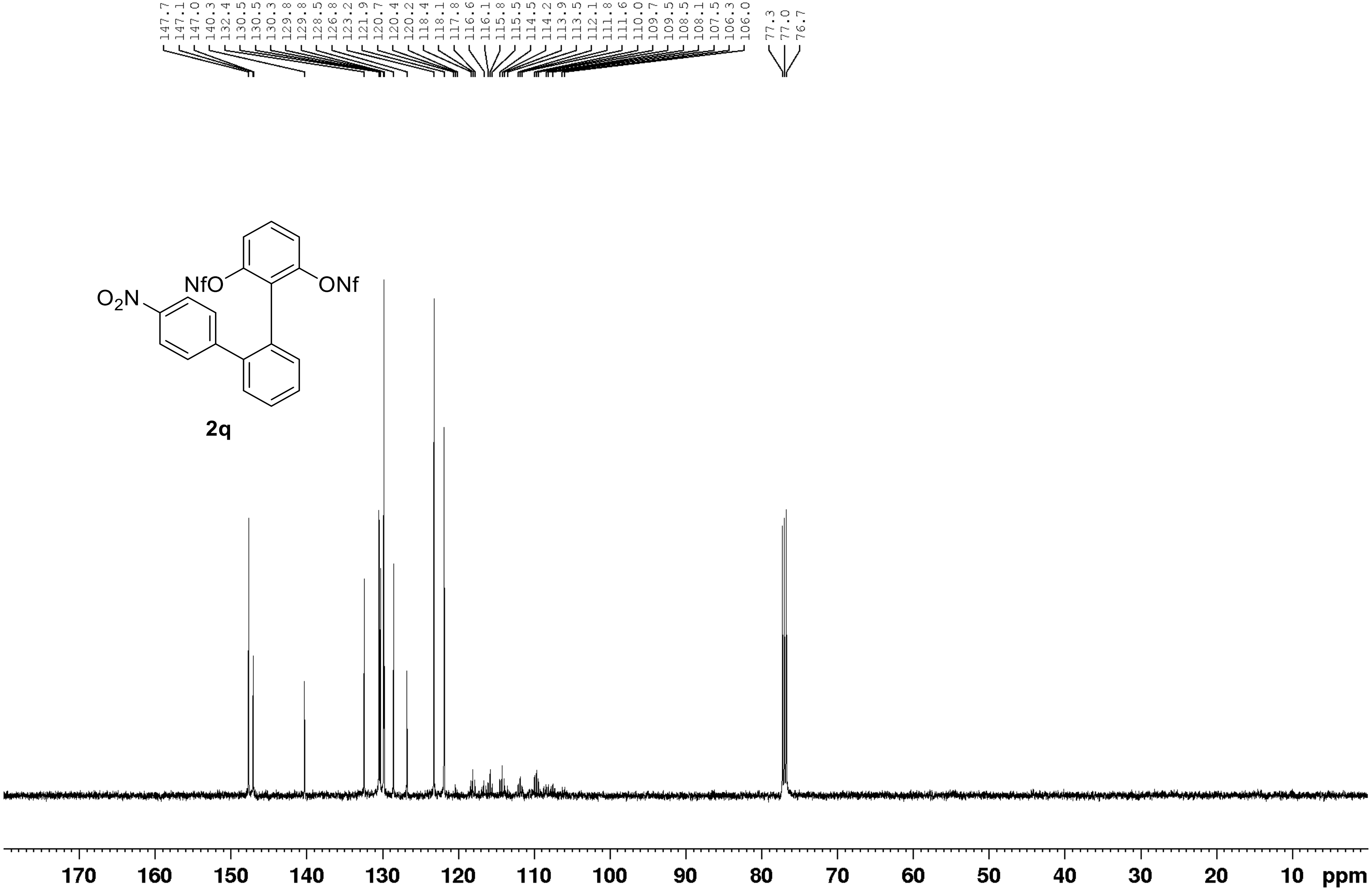

Figure S114. ^{19}**F NMR** (471 MHz, $CDCl_3$, 298 K) of **2q**

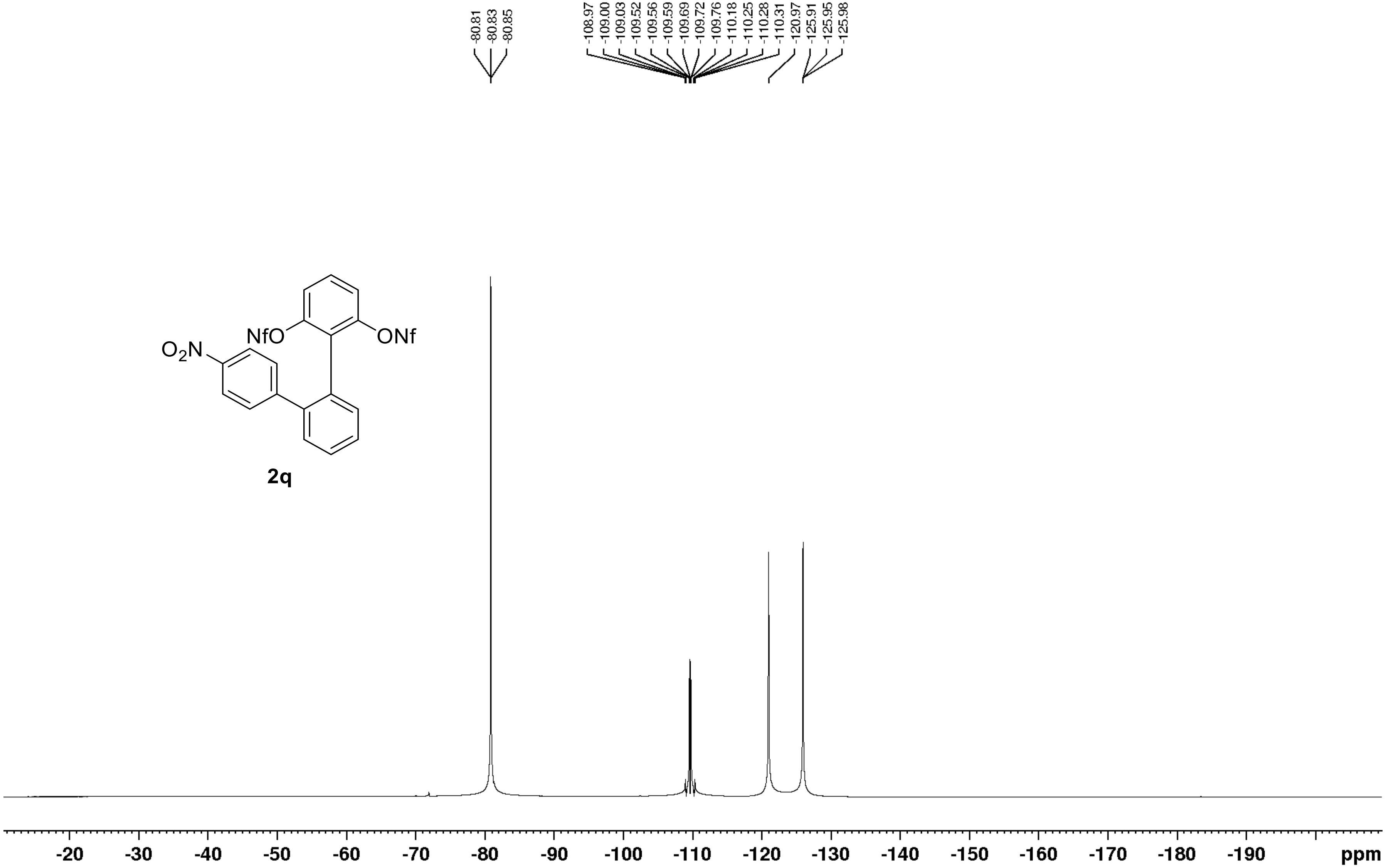

4'-chloro-[1,1':2',1''-terphenyl]-2,6-diyl bis(1,1,2,2,3,3,4,4,4-nonafluorobutane-1-sulfonate) (2r)

Figure S115. ^{1}**H NMR** (500 MHz, $CDCl_3$, 298 K) of **2r**

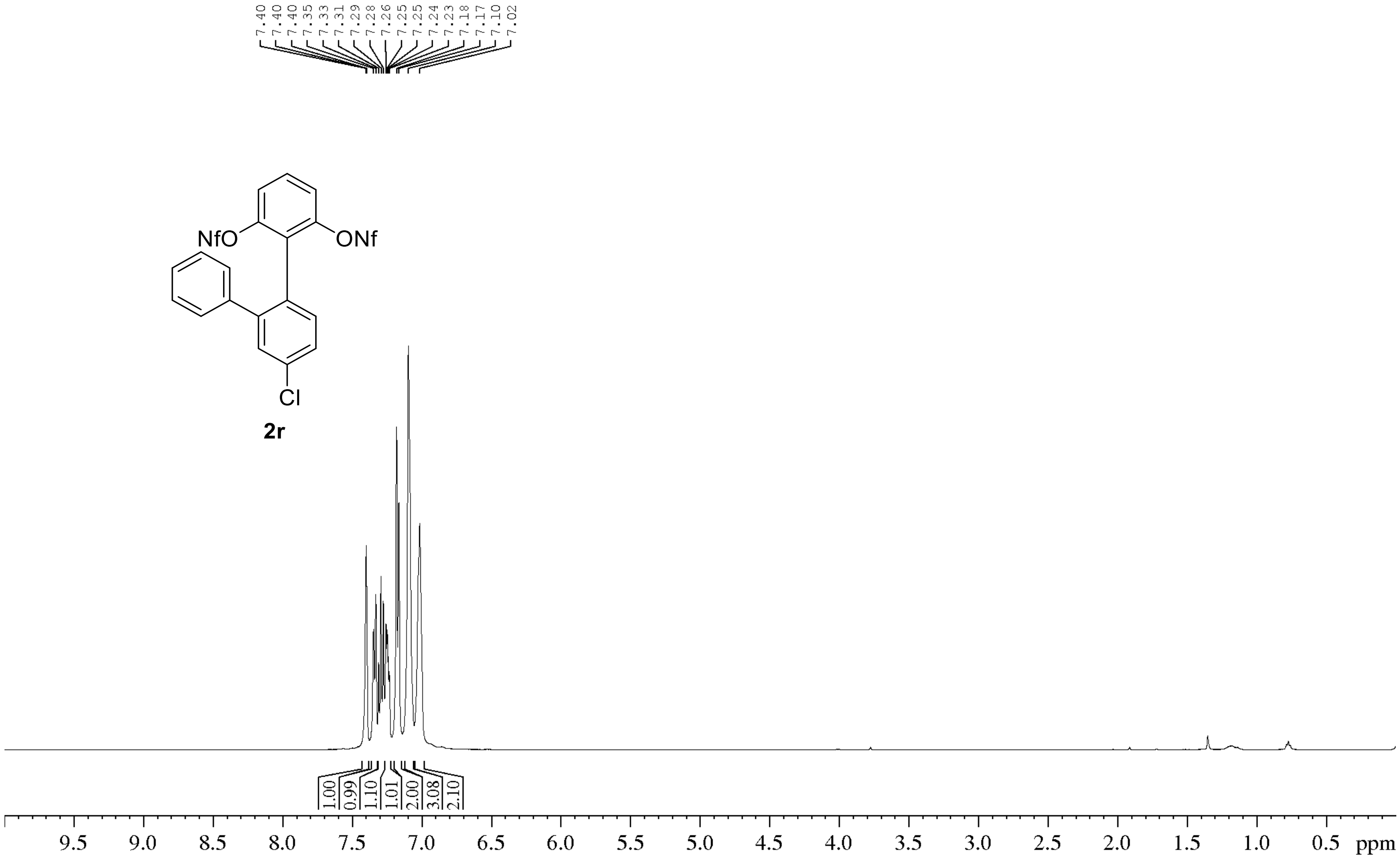

Figure S116. ^{13}**C{**^{1}**H} NMR** (126 MHz, $CDCl_3$, 298 K) of **2r**

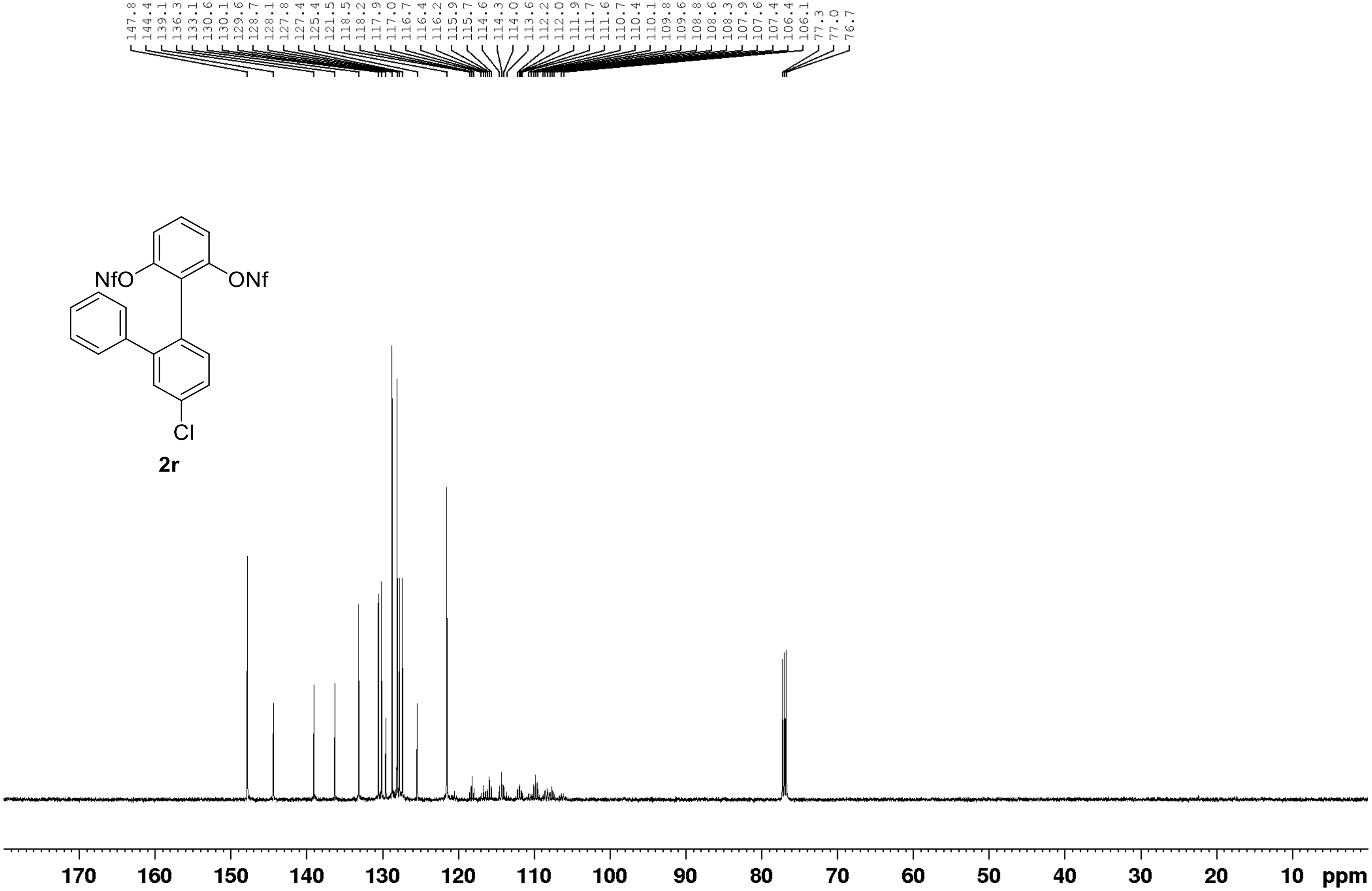

Figure S117. ^{19}**F NMR** (471 MHz, $CDCl_3$, 298 K) of **2r**

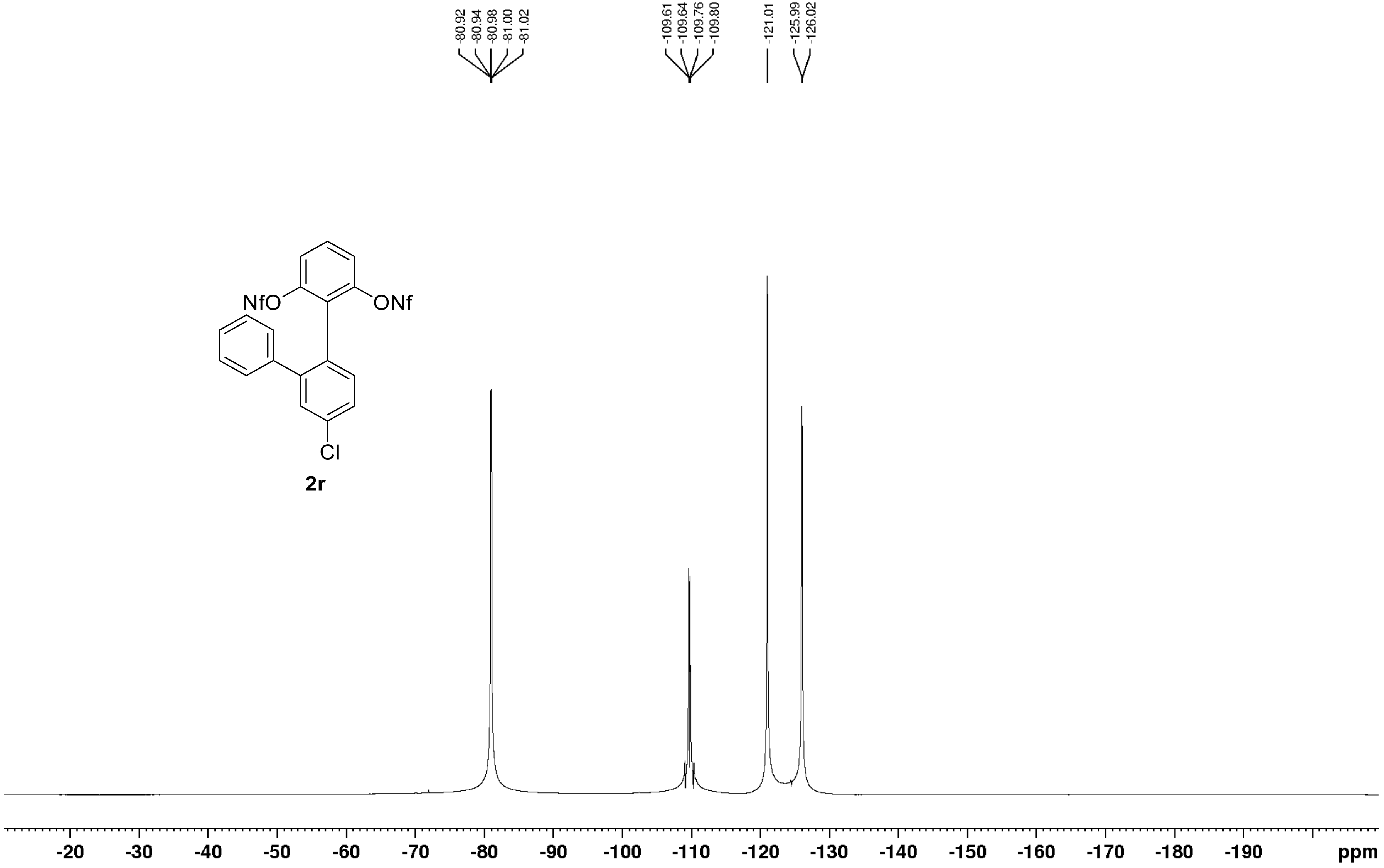

5'-methyl-[1,1':2',1''-terphenyl]-2,6-diyl bis(1,1,2,2,3,3,4,4,4-nonafluorobutane-1-sulfonate) (2s)

Figure S118. ^{1}H NMR (500 MHz, $CDCl_3$, 298 K) of **2s**

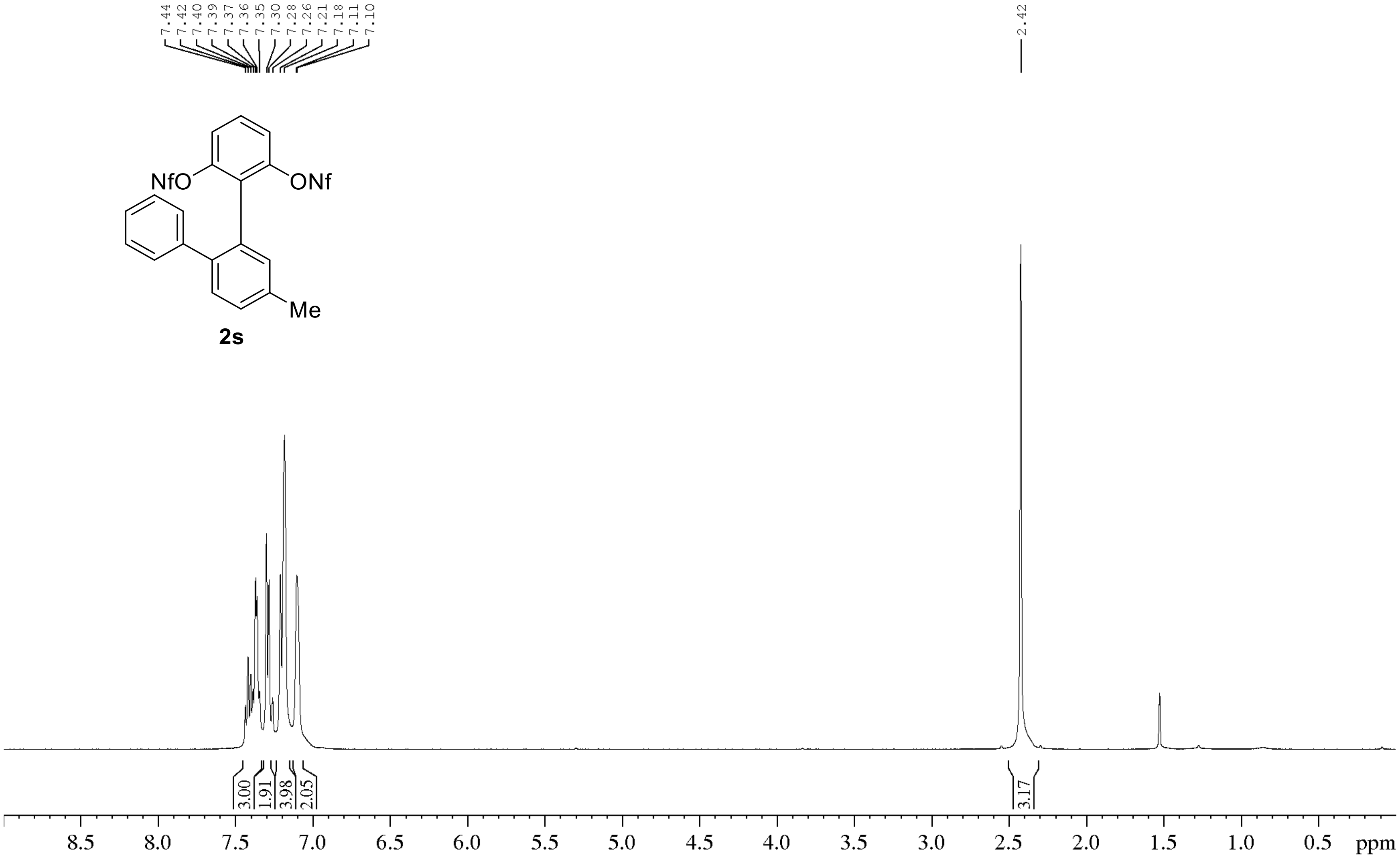

Figure S119. ^{13}**C{**^{1}**H} NMR** (126 MHz, $CDCl_3$, 298 K) of **2s**

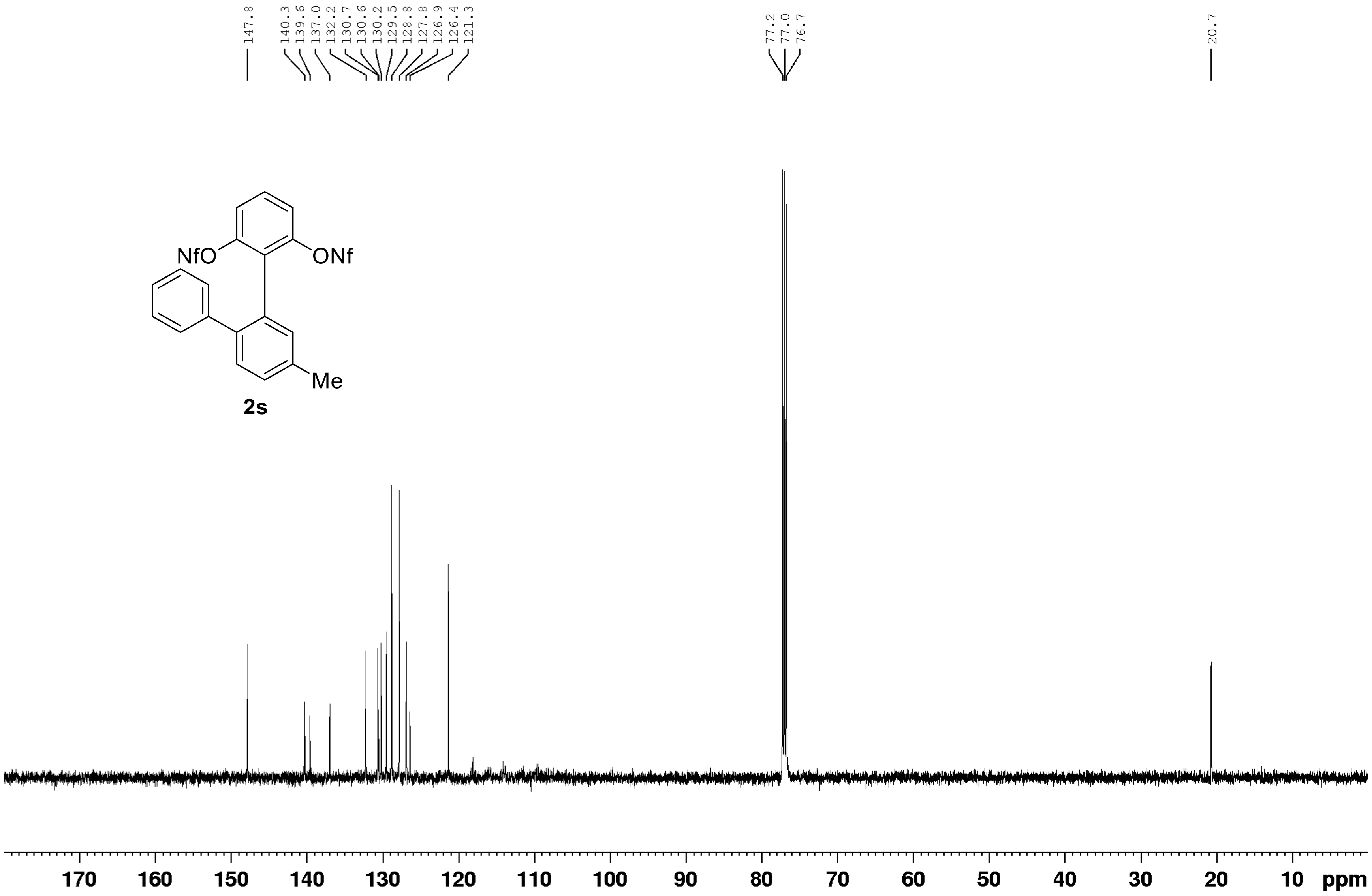

Figure S120. ^{19}**F NMR** (471 MHz, $CDCl_3$, 298 K) of **2s**

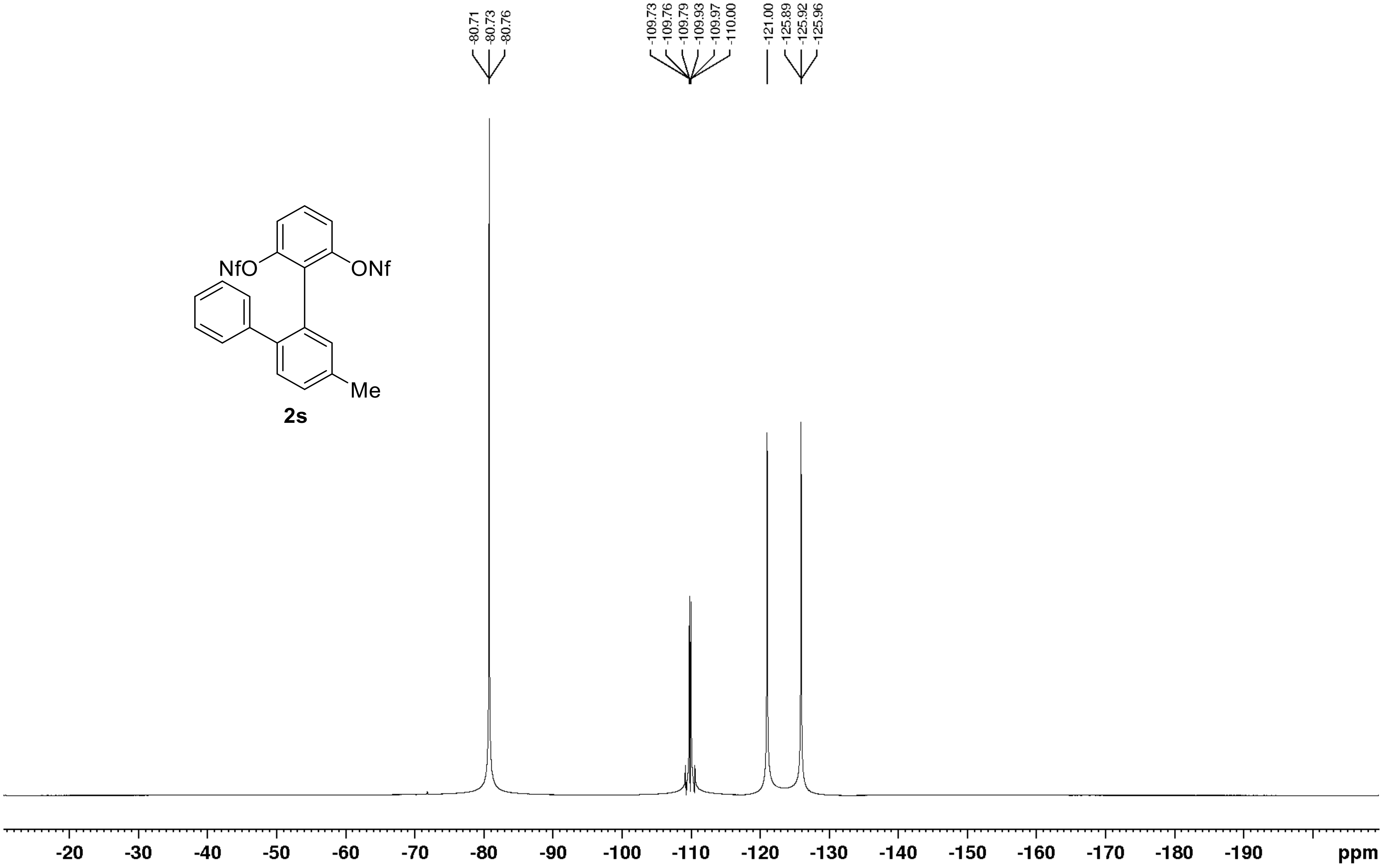

2'-(naphthalen-2-yl)-[1,1'-biphenyl]-2,6-diyl bis(1,1,2,2,3,3,4,4,4-nonafluorobutane-1-sulfonate) (2t)

Figure S121. **^{1}H NMR** (500 MHz, $CDCl_3$, 298 K) of **2t**

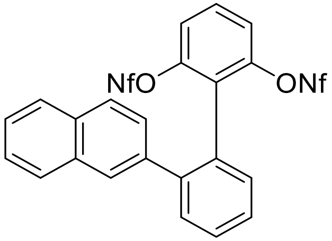

2t

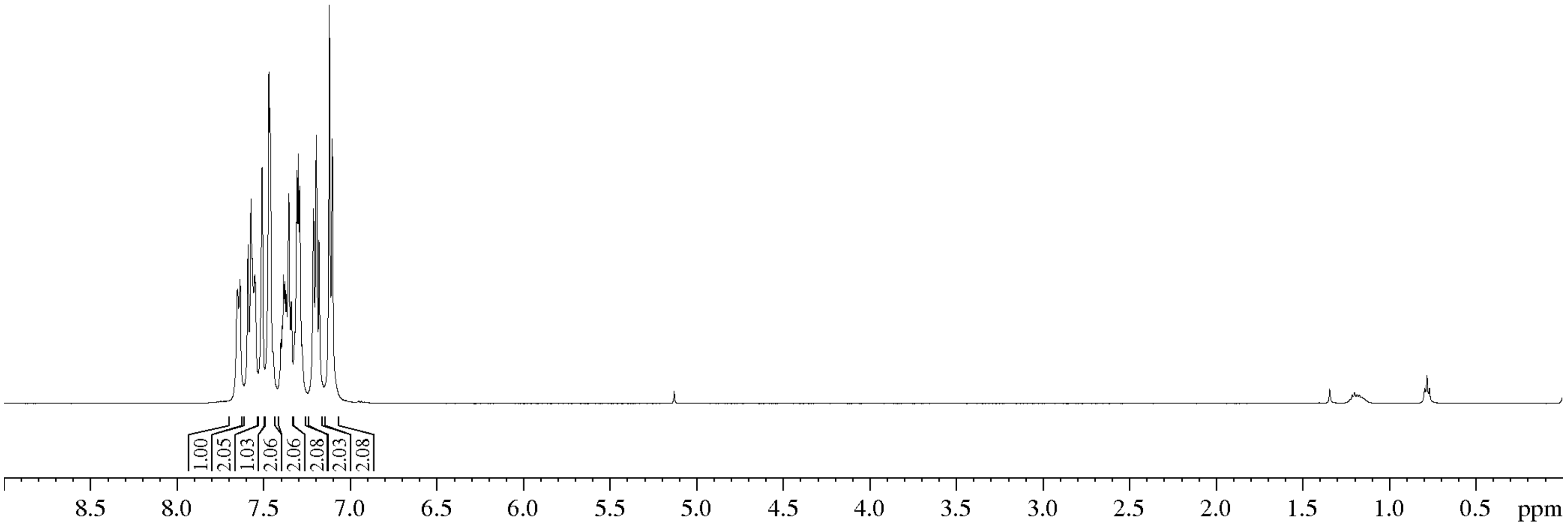

Figure S122. $^{13}C\{^{1}H\}$ NMR (126 MHz, $CDCl_3$, 298 K) of **2t**

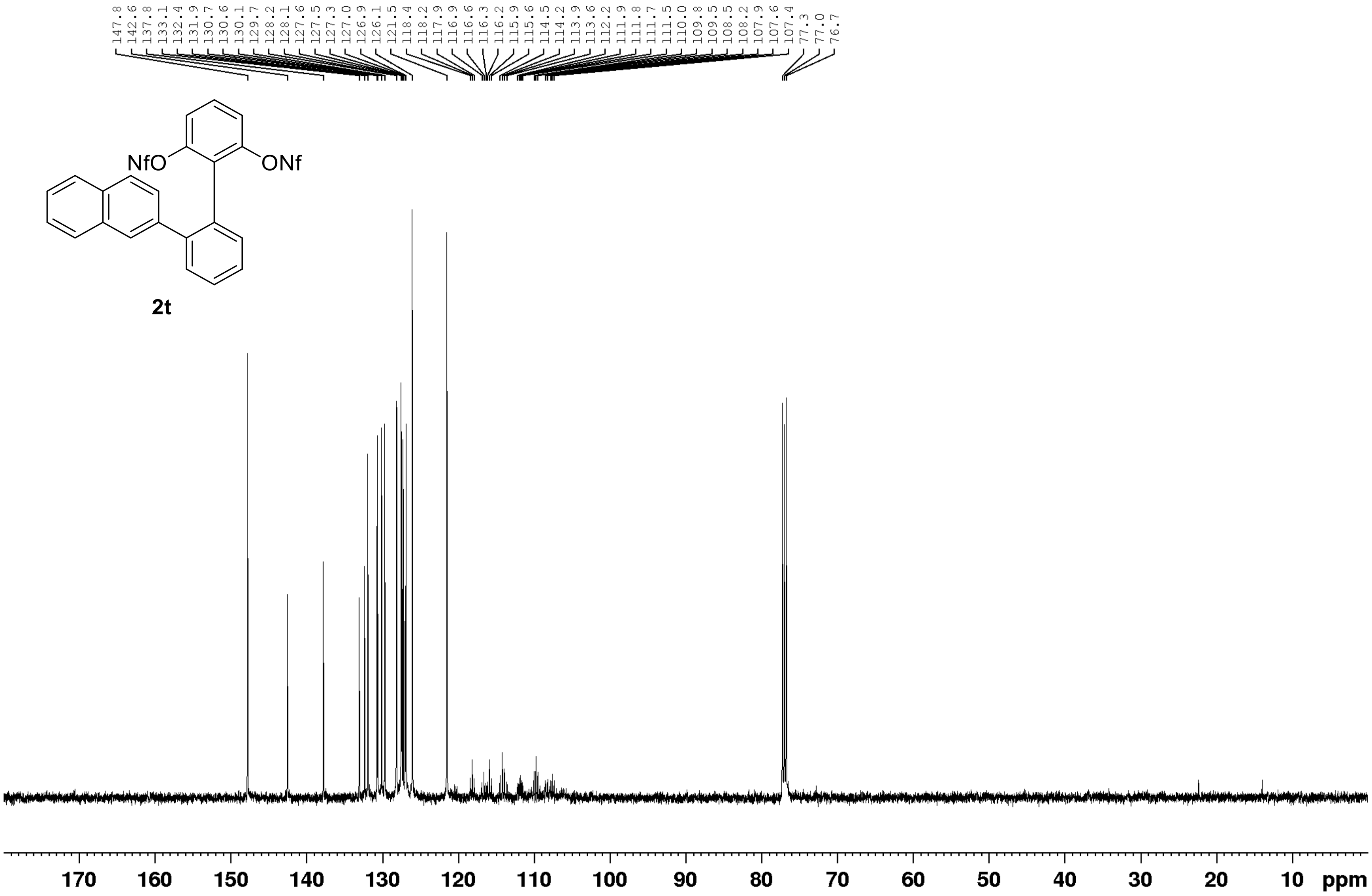

Figure S123. **^{19}F NMR** (471 MHz, $CDCl_3$, 298 K) of **2t**

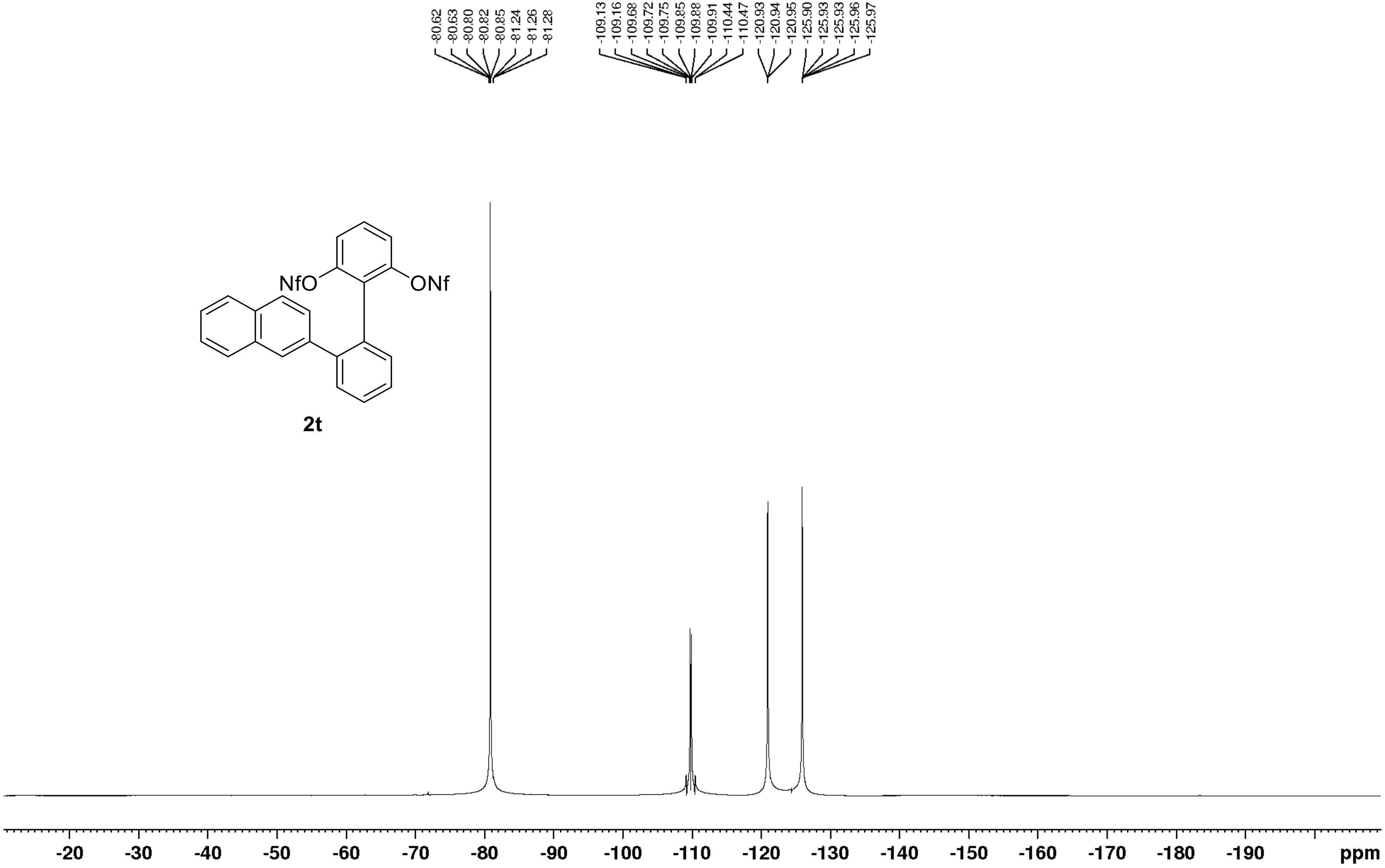

2'-(thiophen-3-yl)-[1,1'-biphenyl]-2,6-diyl bis(1,1,2,2,3,3,4,4,4-nonafluorobutane-1-sulfonate) (2u)

Figure S124. **^{1}H NMR** (500 MHz, $CDCl_3$, 298 K) of **2u**

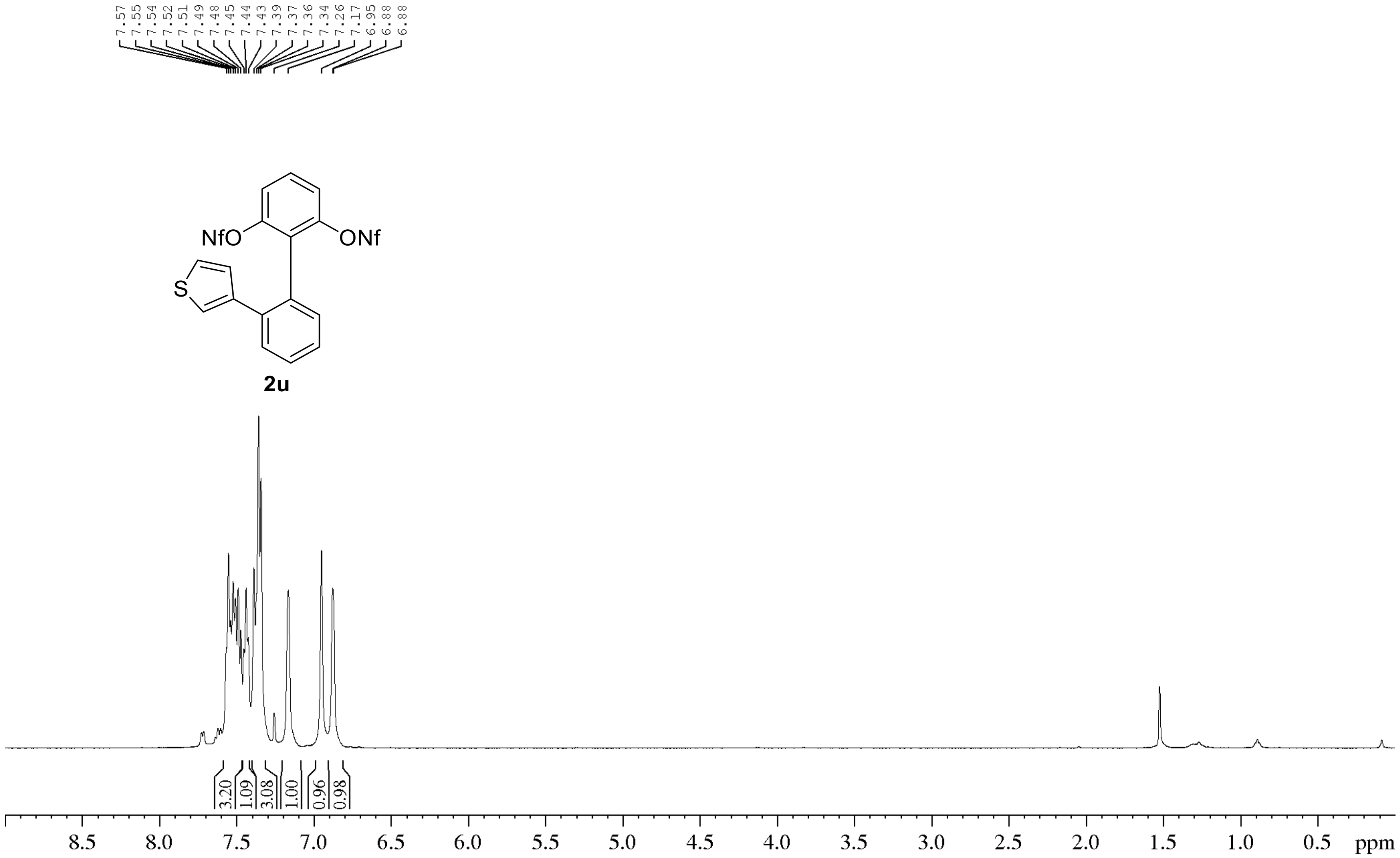

Figure S125. ^{13}**C{**^{1}**H} NMR** (126 MHz, $CDCl_3$, 298 K) of **2u**

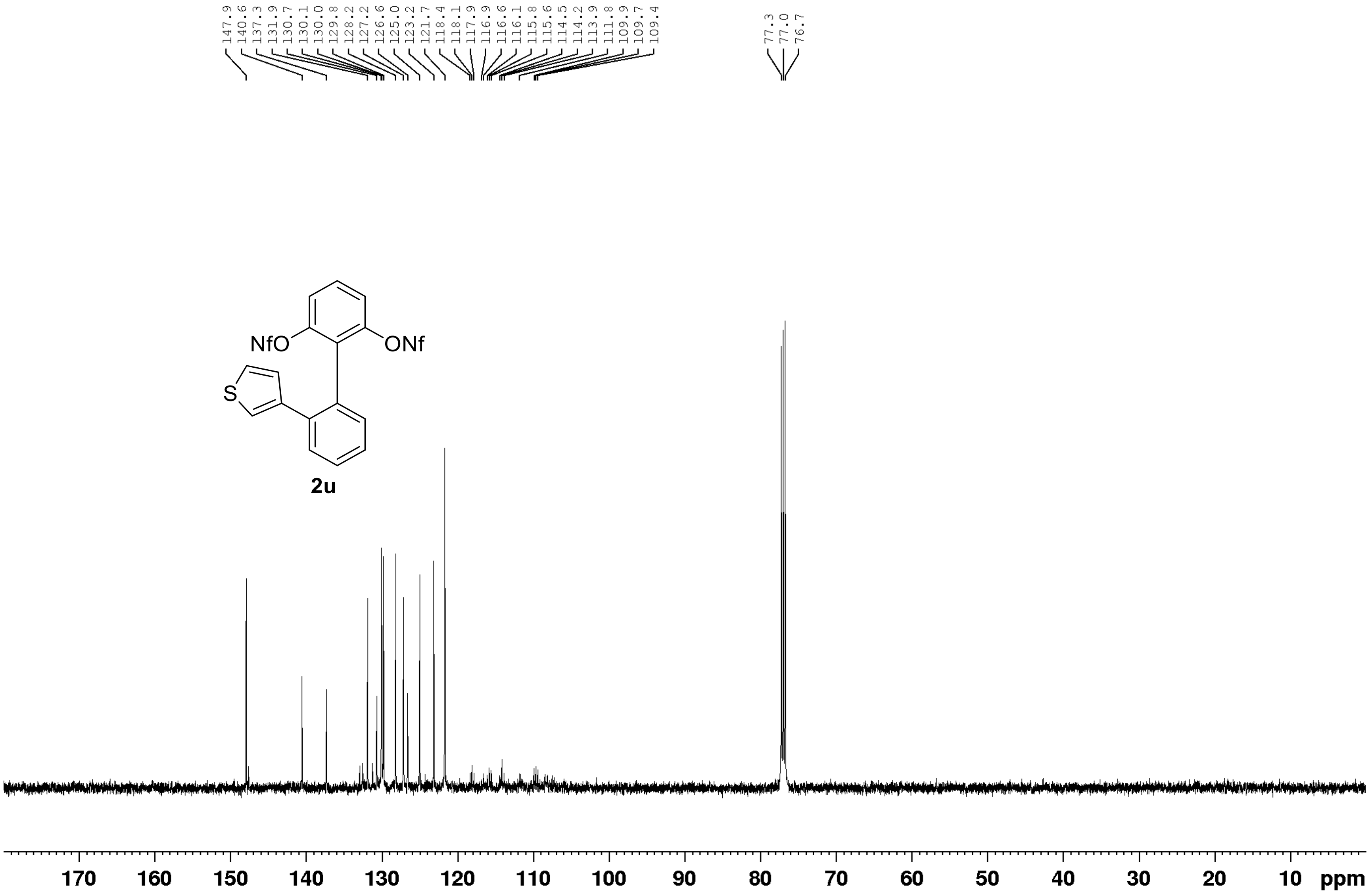

Figure S126. ^{19}F NMR (471 MHz, $CDCl_3$, 298 K) of **2u**

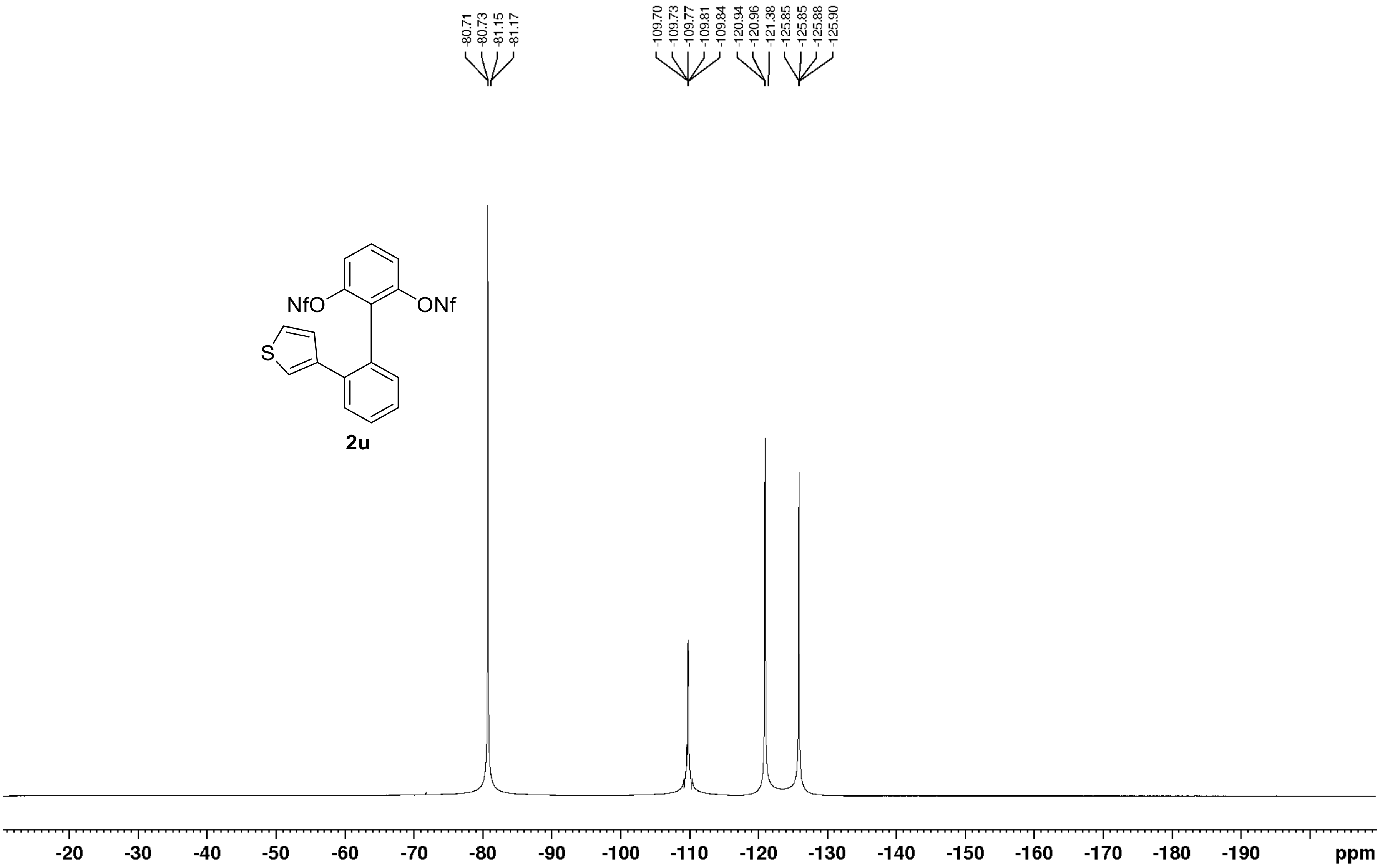

2'-cyano-[1,1'-biphenyl]-2,6-diyl bis(1,1,2,2,3,3,4,4,4-nonafluorobutane-1-sulfonate) (2v)

Figure S127. **^{1}H NMR** (500 MHz, $CDCl_3$, 298 K) of **2v**

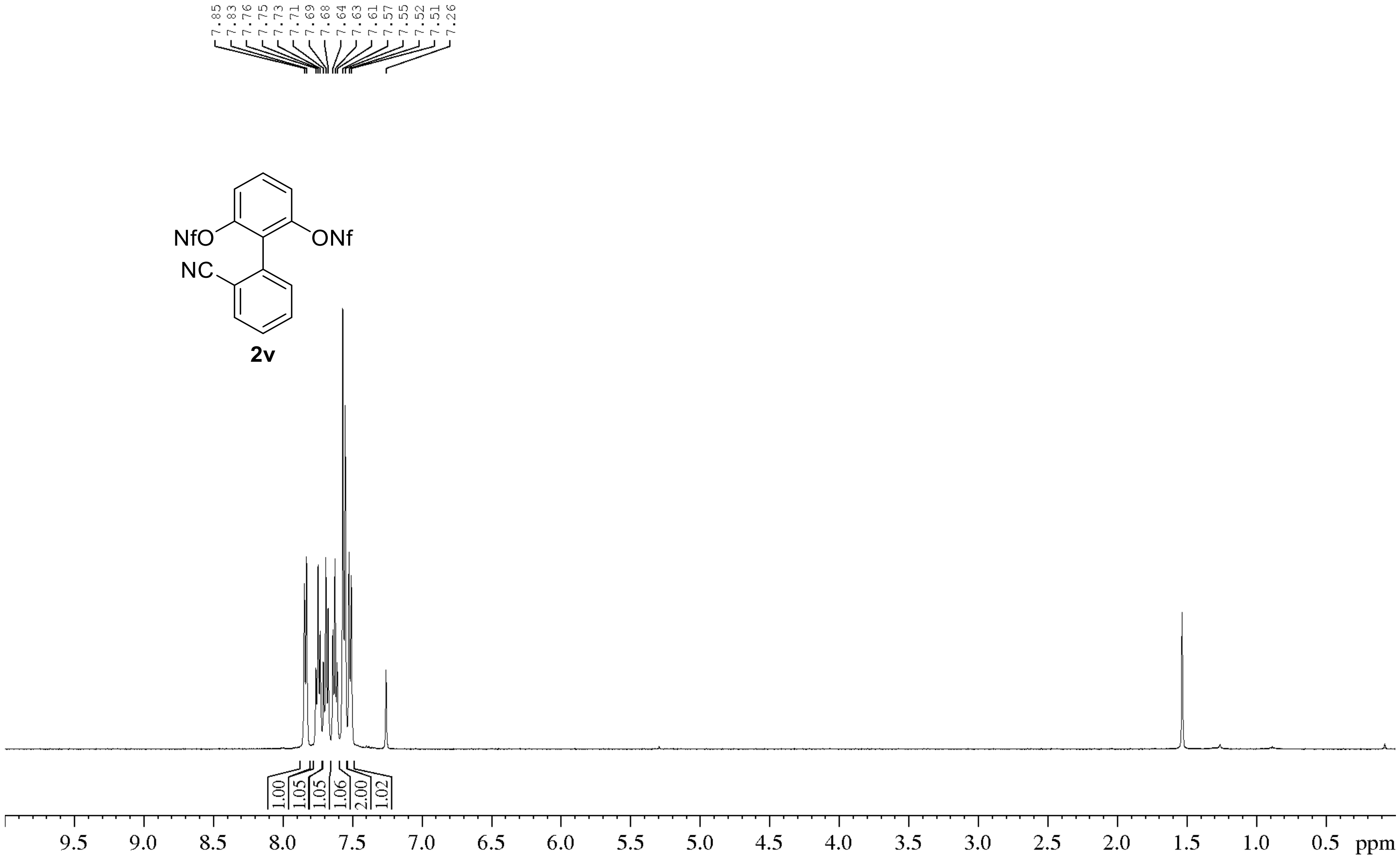

Figure S128. ^{13}**C{**^{1}**H} NMR** (126 MHz, $CDCl_3$, 298 K) of **2v**

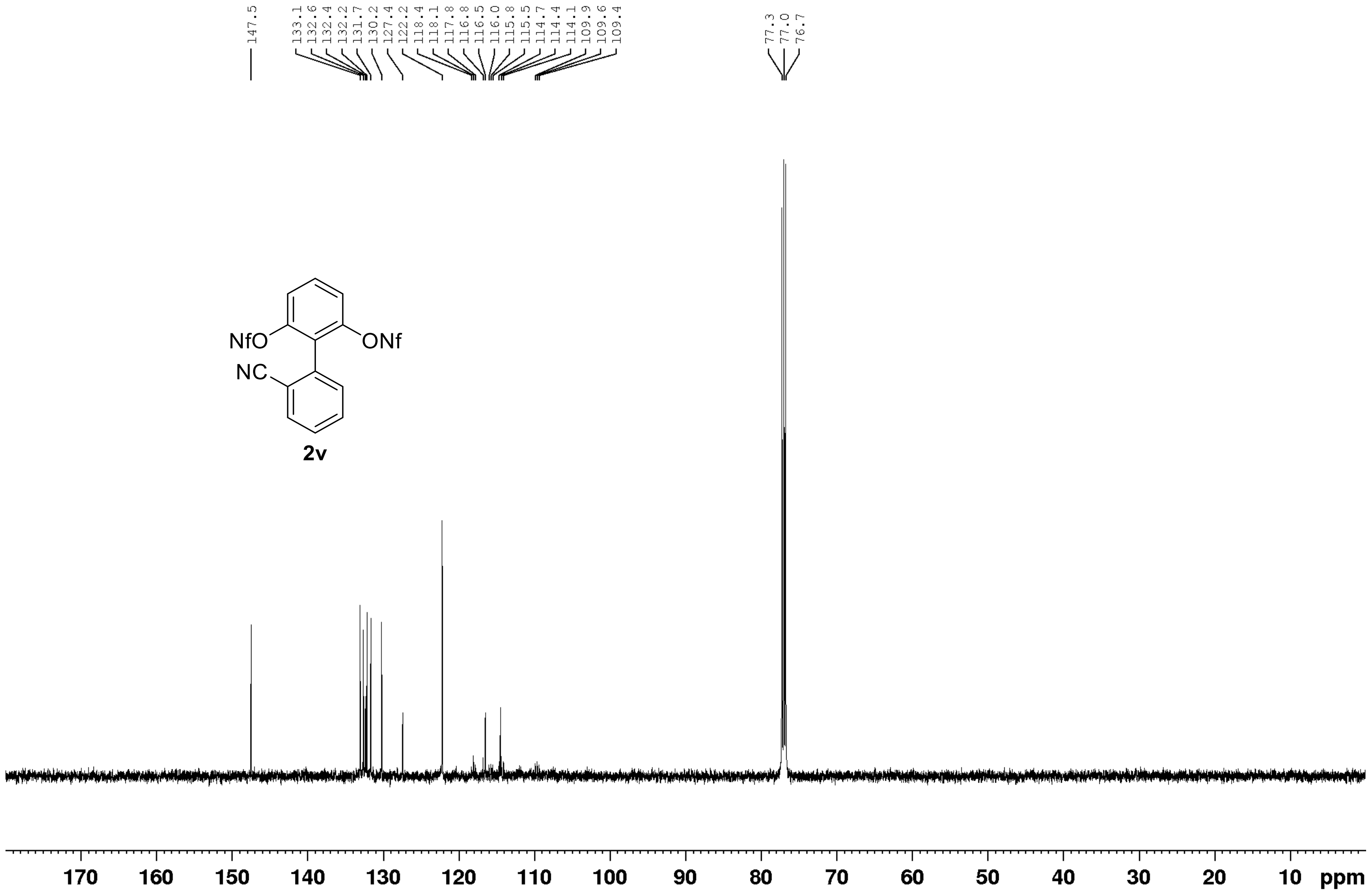

Figure S129. **^{19}F NMR** (471 MHz, $CDCl_3$, 298 K) of **2v**

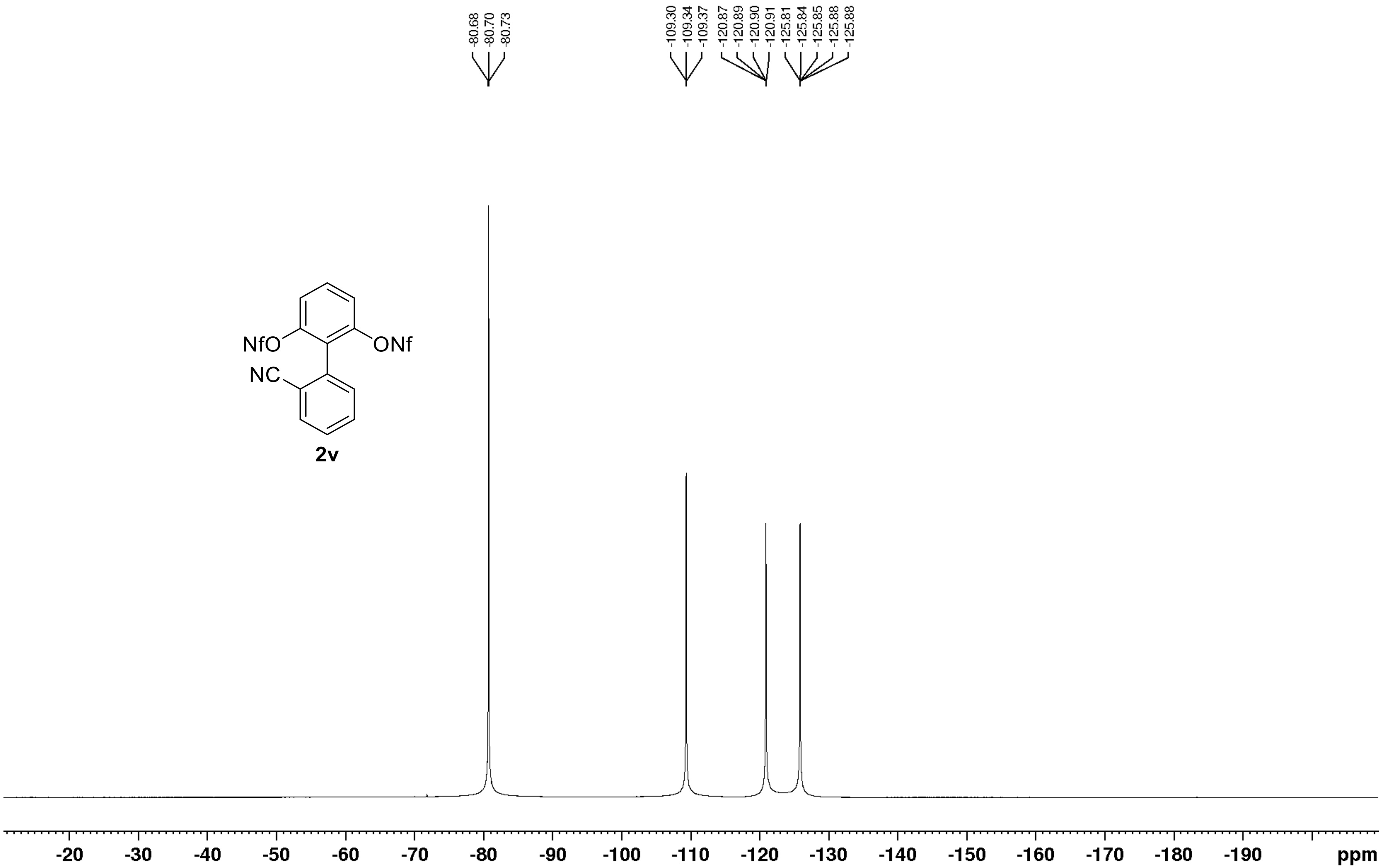

2'-isopropyl-[1,1'-biphenyl]-2,6-diyl bis(1,1,2,2,3,3,4,4,4-nonafluorobutane-1-sulfonate) (2w)

Figure S130. ^{1}**H NMR** (500 MHz, $CDCl_3$, 298 K) of **2w**

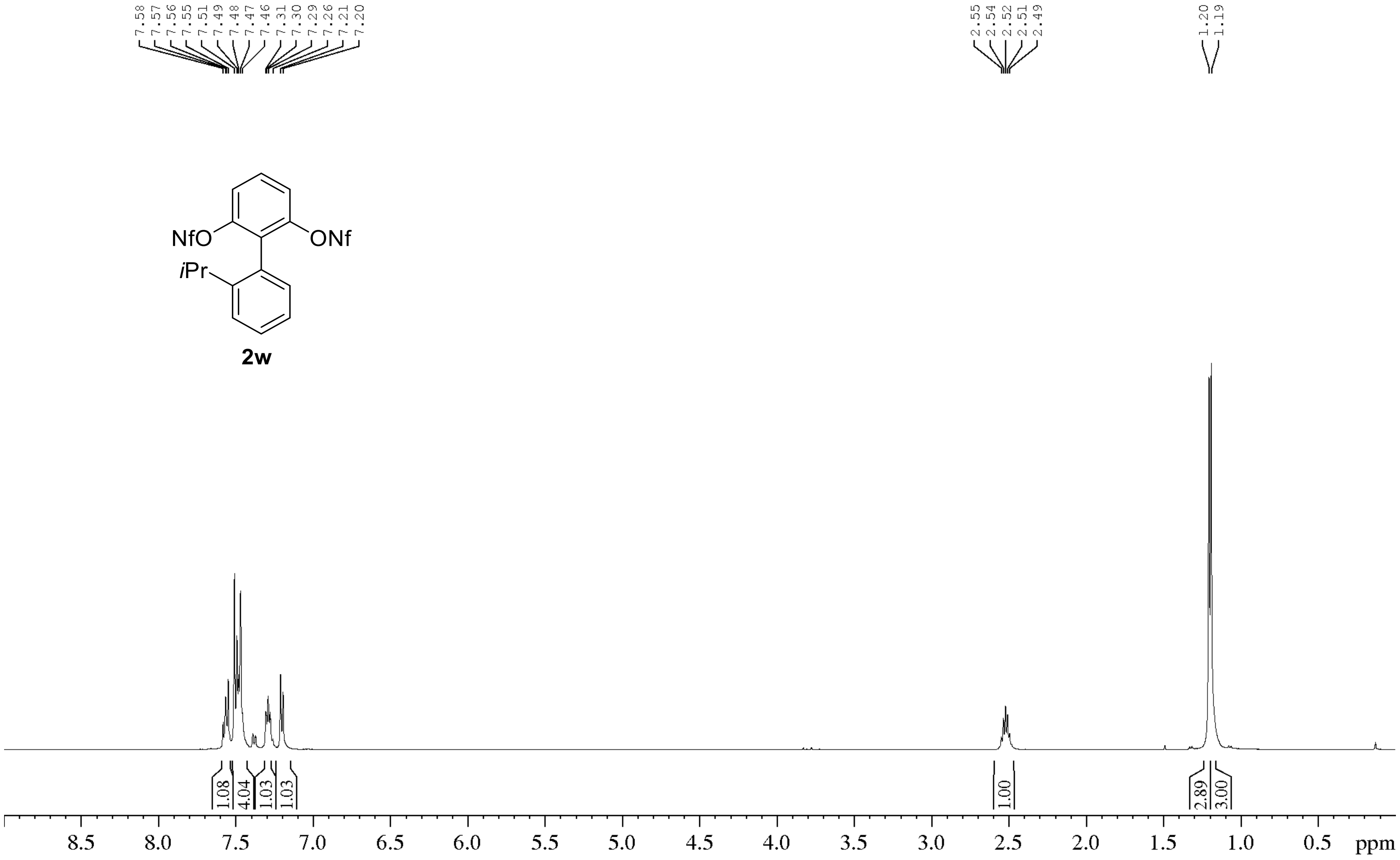

Figure S131. **^{13}C{^{1}H} NMR** (126 MHz, $CDCl_3$, 298 K) of **2w**

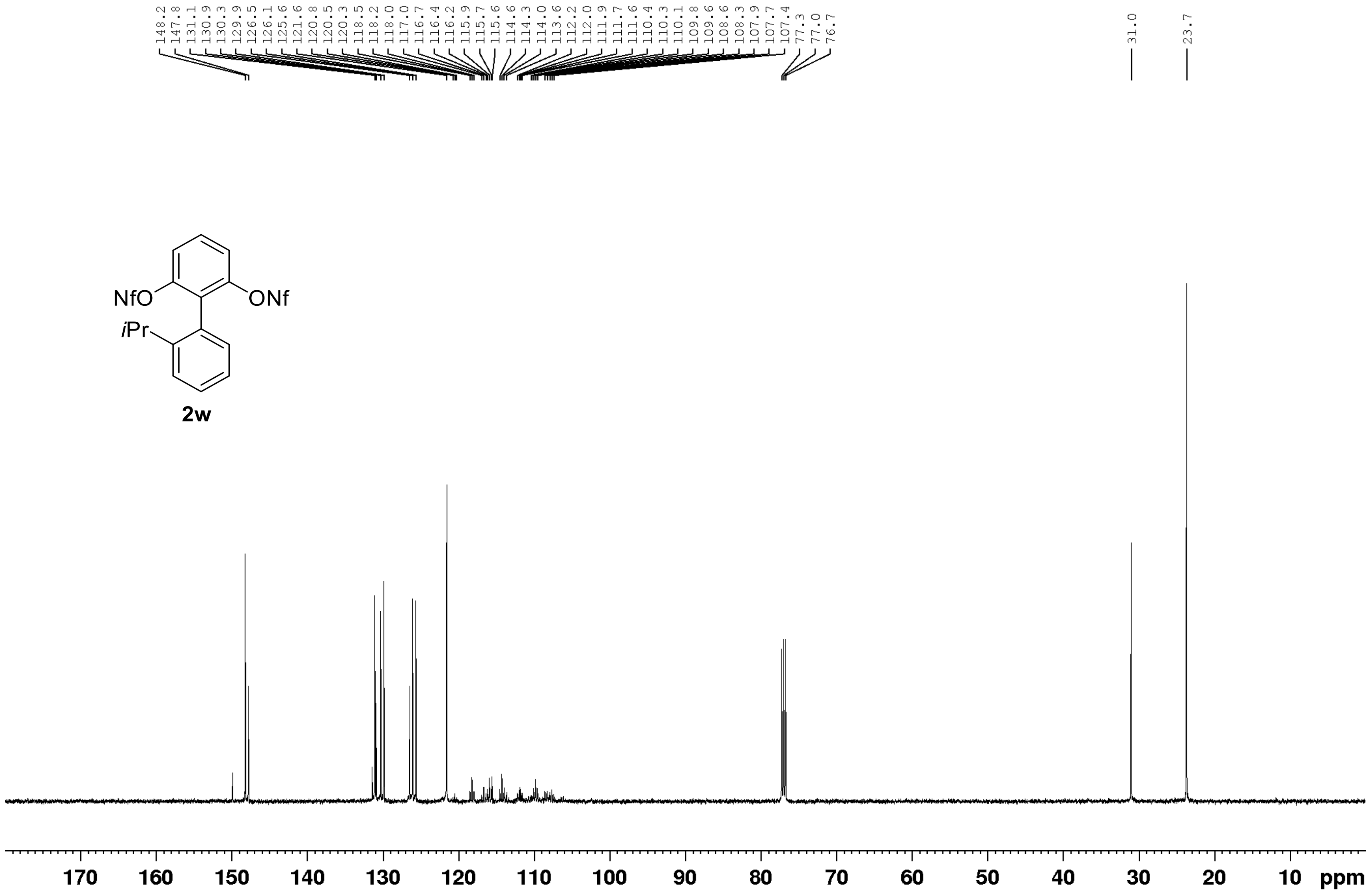

Figure S132. ^{19}**F NMR** (471 MHz, $CDCl_3$, 298 K) of **2w**

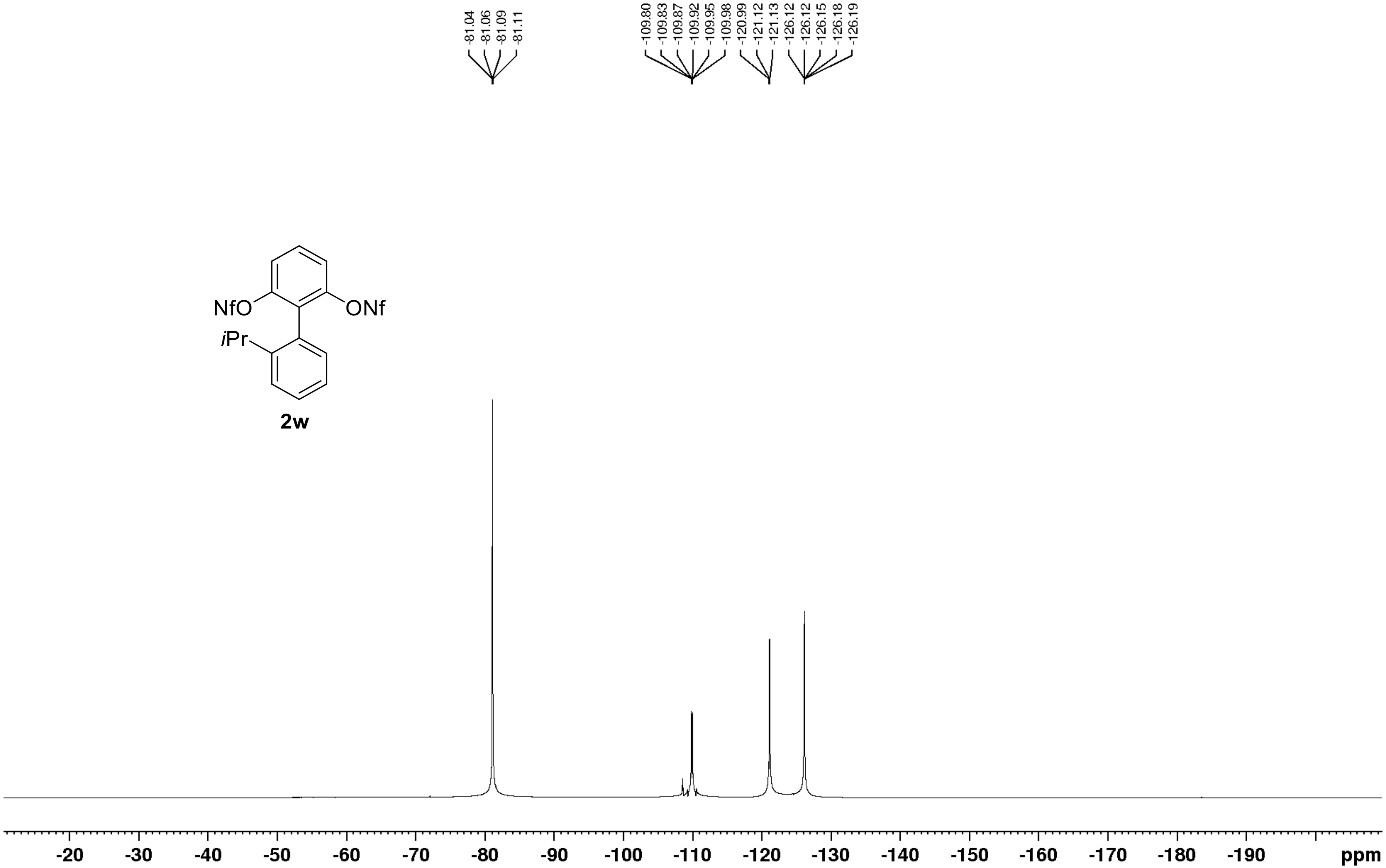

2'-methyl-[1,1'-biphenyl]-2,6-diyl bis(1,1,2,2,3,3,4,4,4-nonafluorobutane-1-sulfonate) (2x)

Figure S133. ^{1}H NMR (500 MHz, $CDCl_3$, 298 K) of **2x**

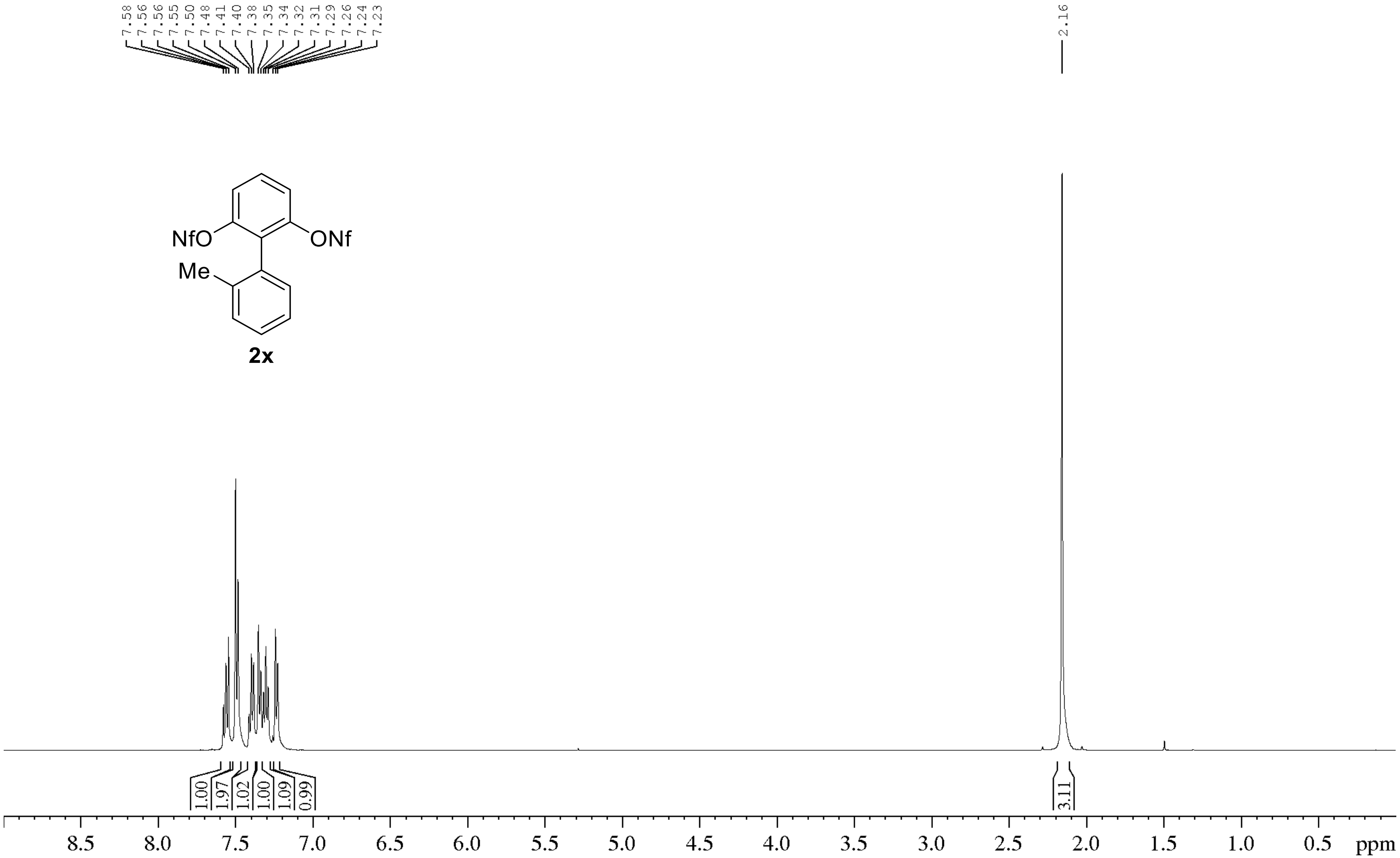

Figure S134. ^{13}C{^{1}H} NMR (126 MHz, $CDCl_3$, 298 K) of **2x**

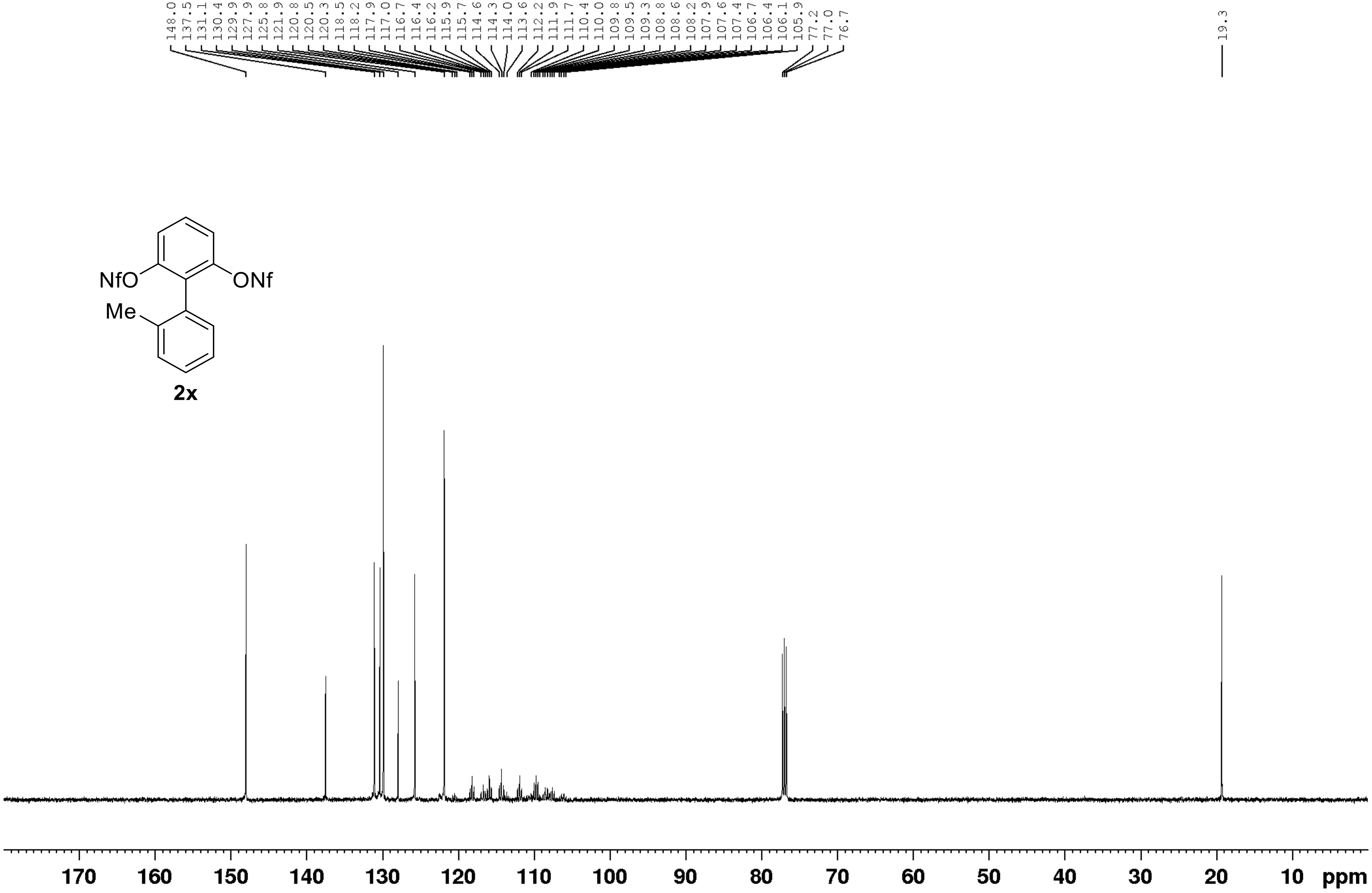

Figure S135. ^{19}**F NMR** (471 MHz, $CDCl_3$, 298 K) of **2x**

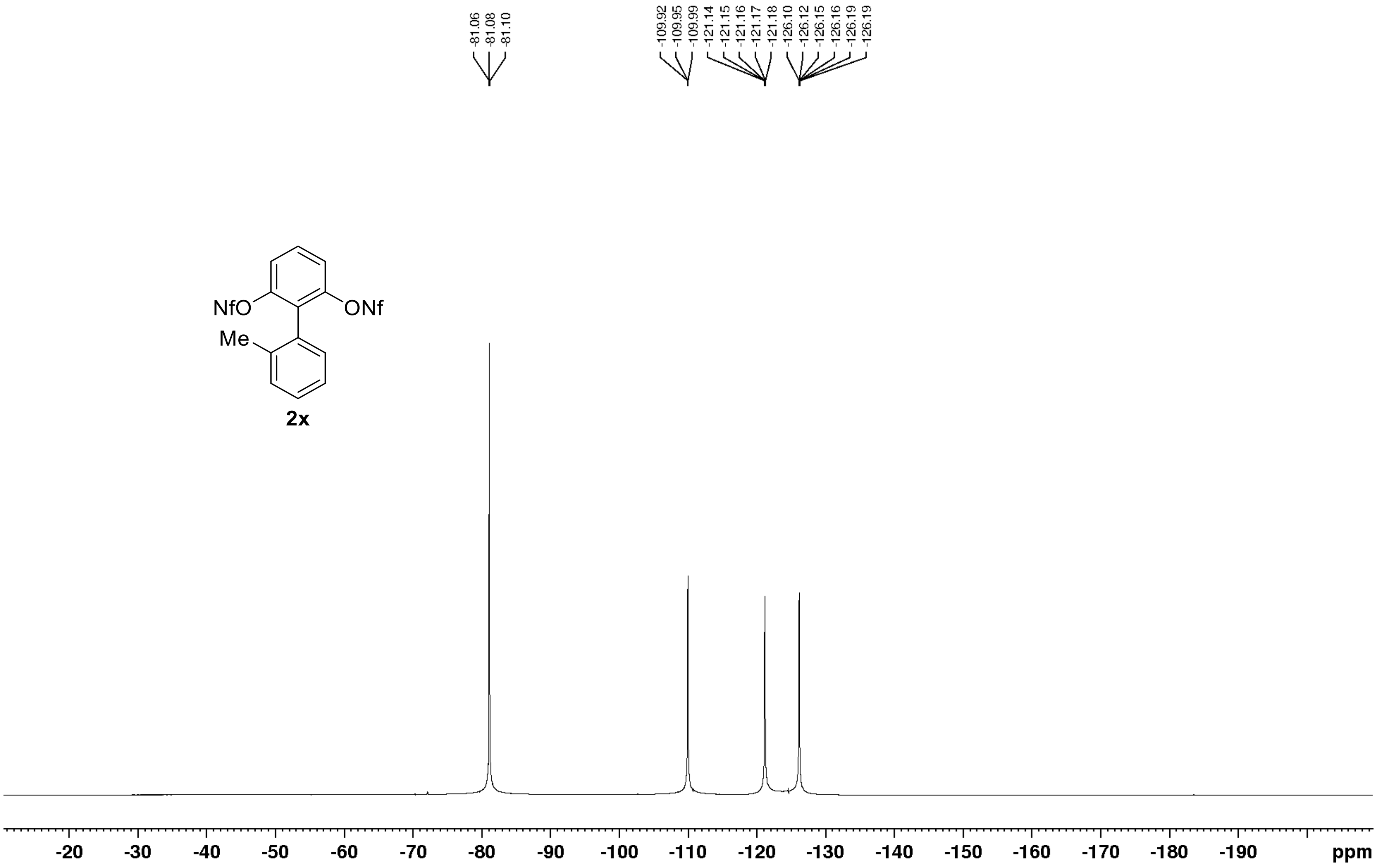

2'-chloro-[1,1'-biphenyl]-2,6-diyl bis(1,1,2,2,3,3,4,4,4-nonafluorobutane-1-sulfonate) (2y)

Figure S136. ^{1}H NMR (500 MHz, $CDCl_3$, 298 K) of **2y**

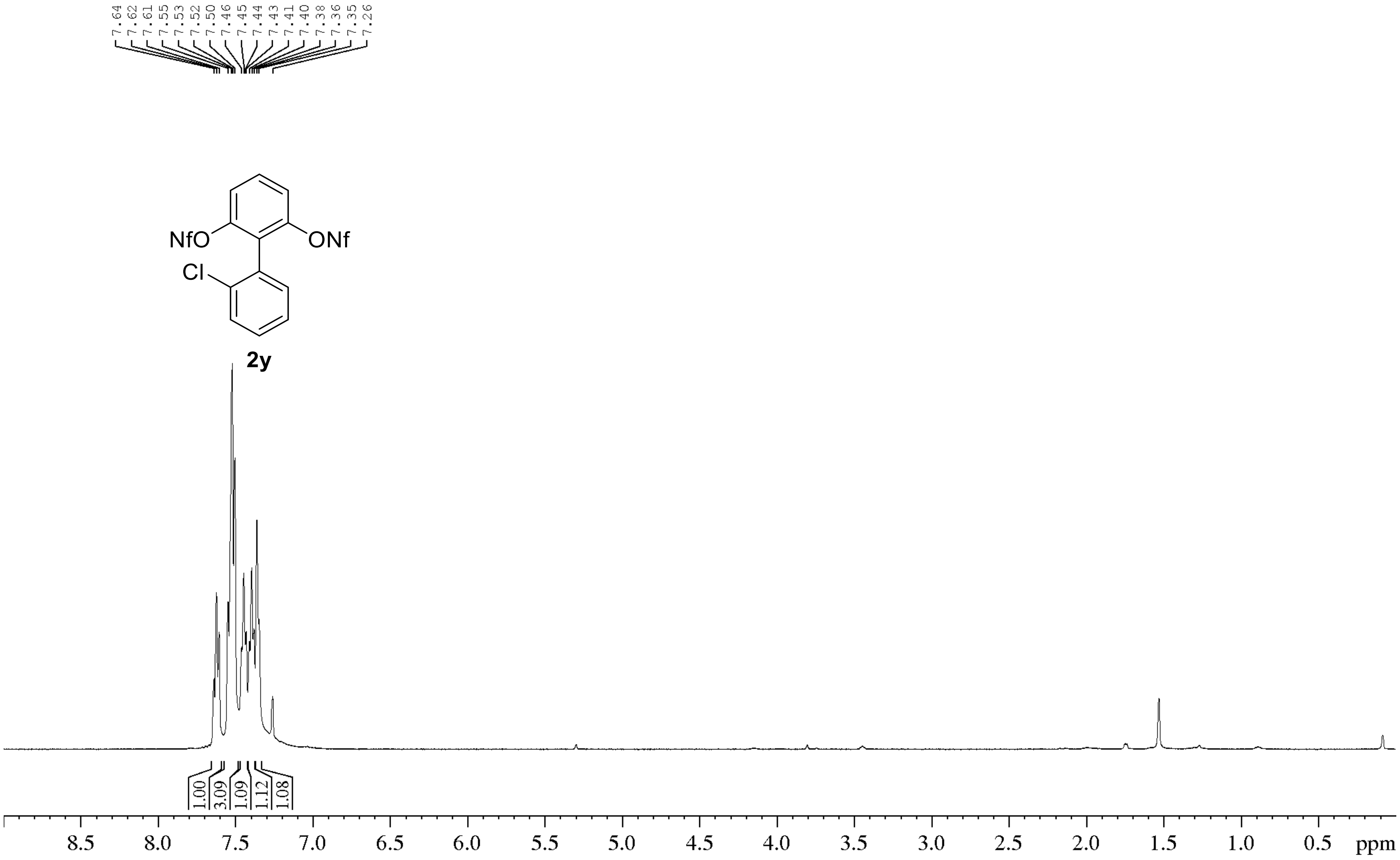

Figure S137. ^{13}**C{**^{1}**H} NMR** (126 MHz, $CDCl_3$, 298 K) of **2y**

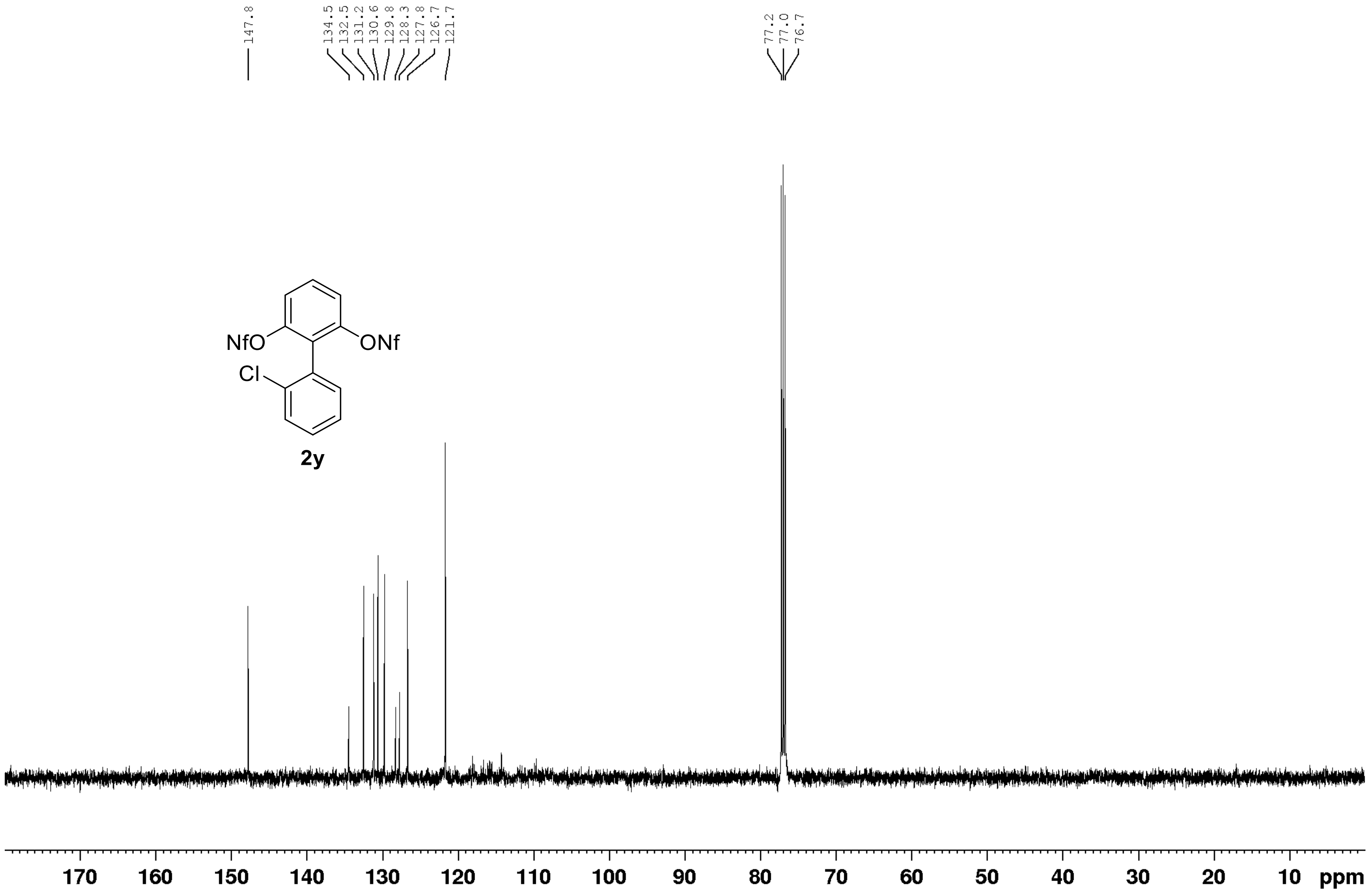

Figure S138. ^{19}**F NMR** (471 MHz, $CDCl_3$, 298 K) of **2y**

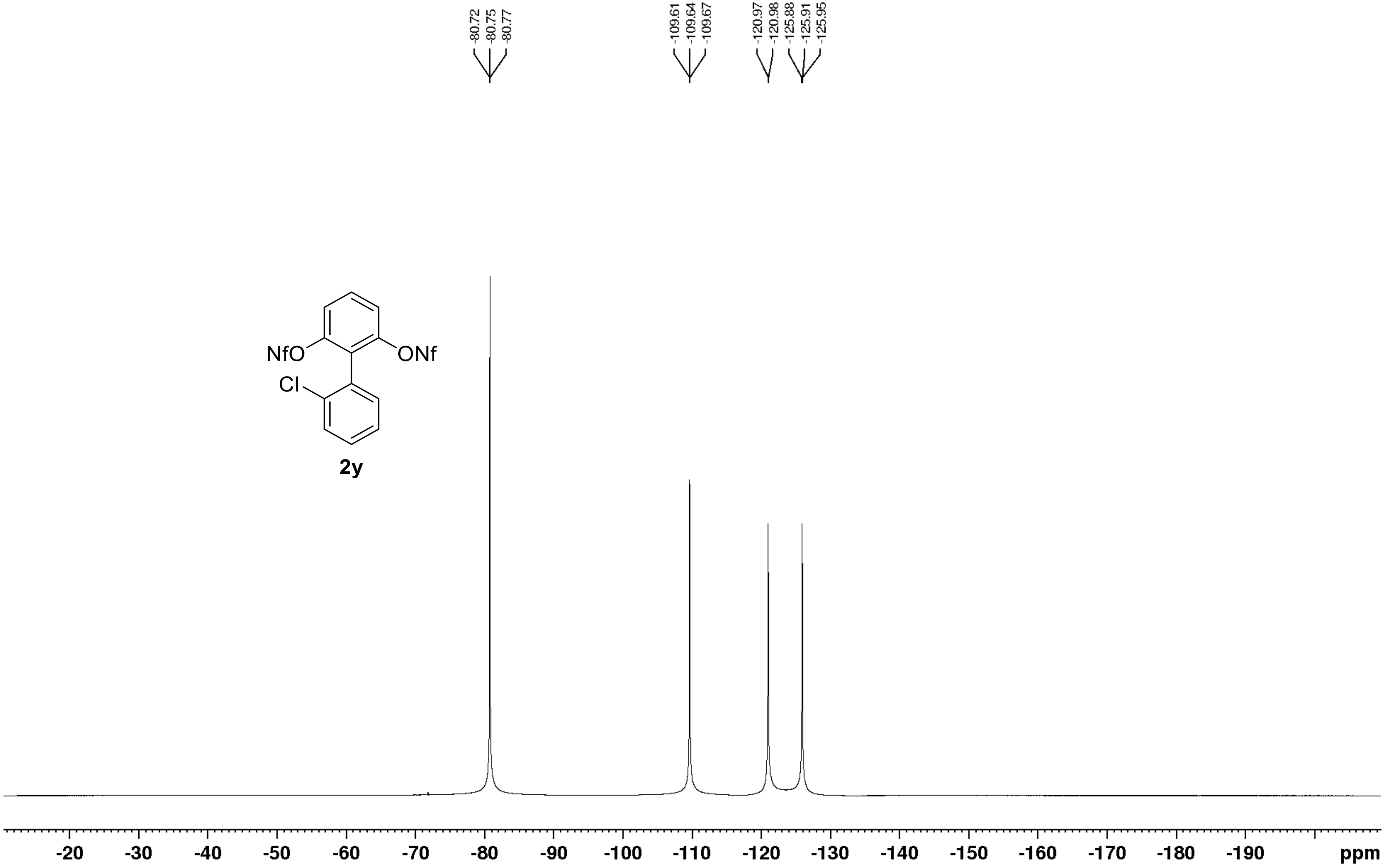

(*R*)-2-(naphthalen-1-yl)-3-(4,4,5,5-tetramethyl-1,3,2-dioxaborolan-2-yl)phenyl 1,1,2,2,3,3,4,4,4-nonafluorobutane-1-sulfonate (7aa)

Figure S139. **^{1}H NMR** (500 MHz, $CDCl_3$, 298 K) of **7aa**

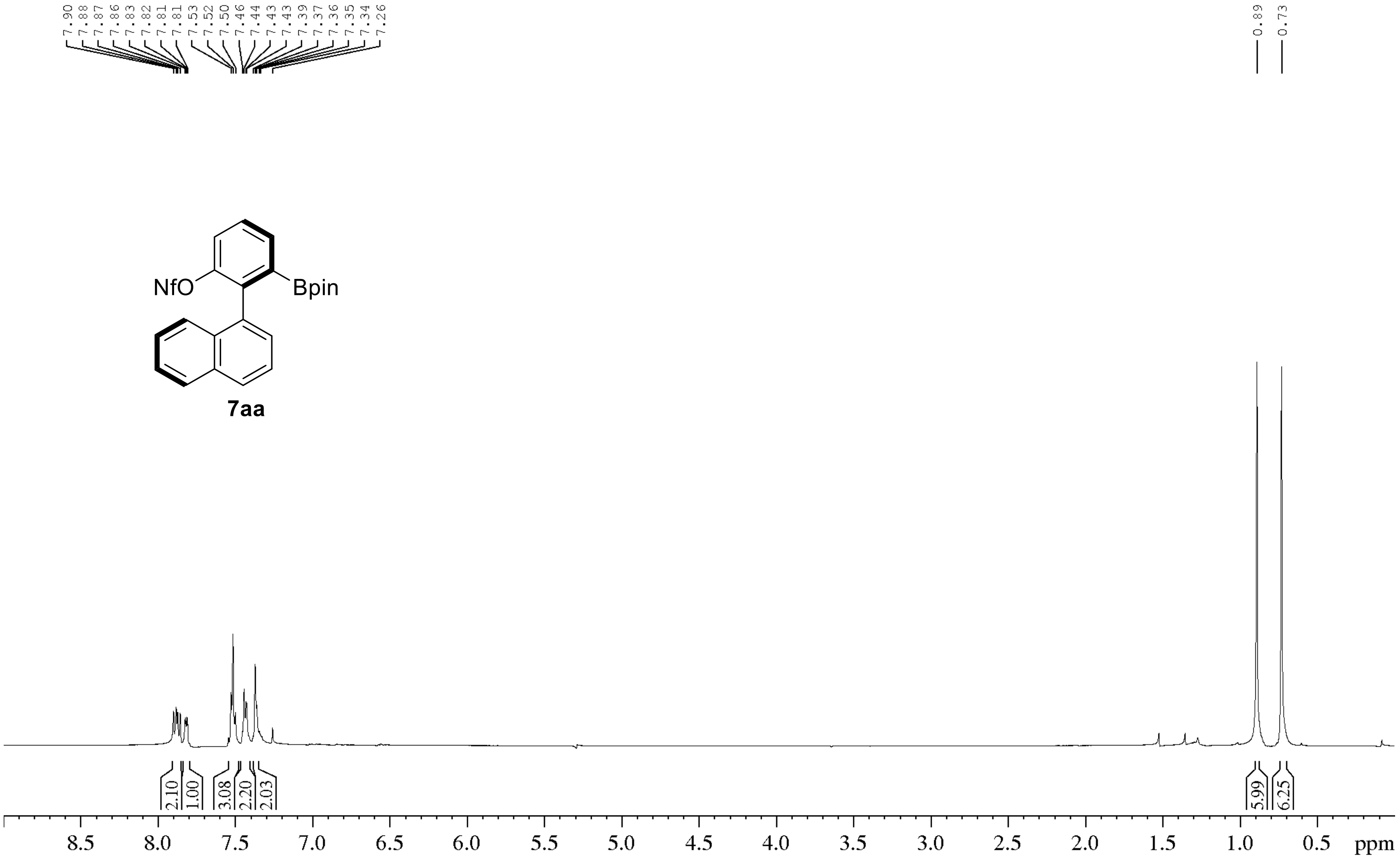

Figure S140. ^{13}**C{**^{1}**H} NMR** (126 MHz, $CDCl_3$, 298 K) of **7aa**

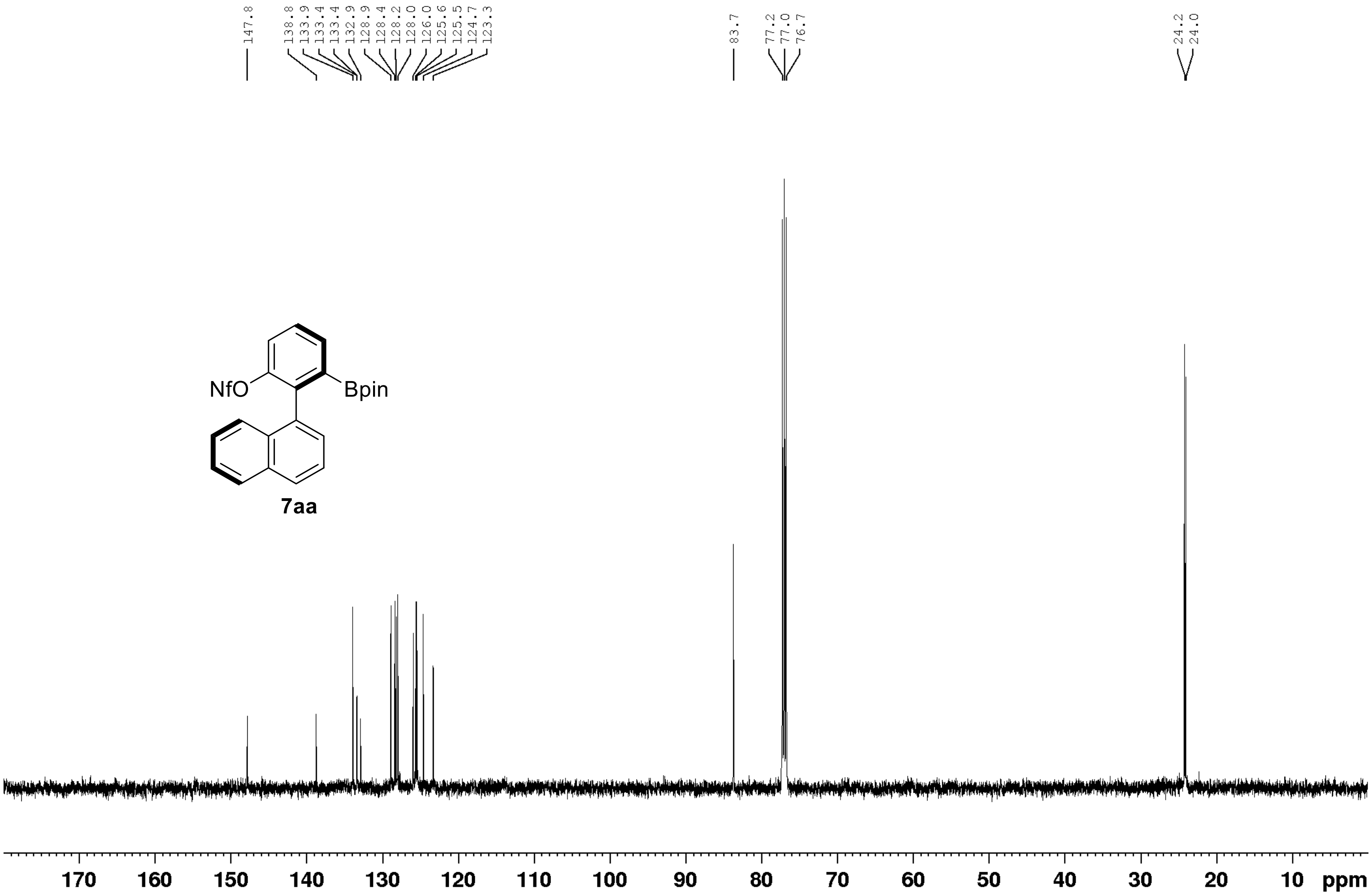

Figure S141. **^{19}F NMR** (471 MHz, $CDCl_3$, 298 K) of **7aa**

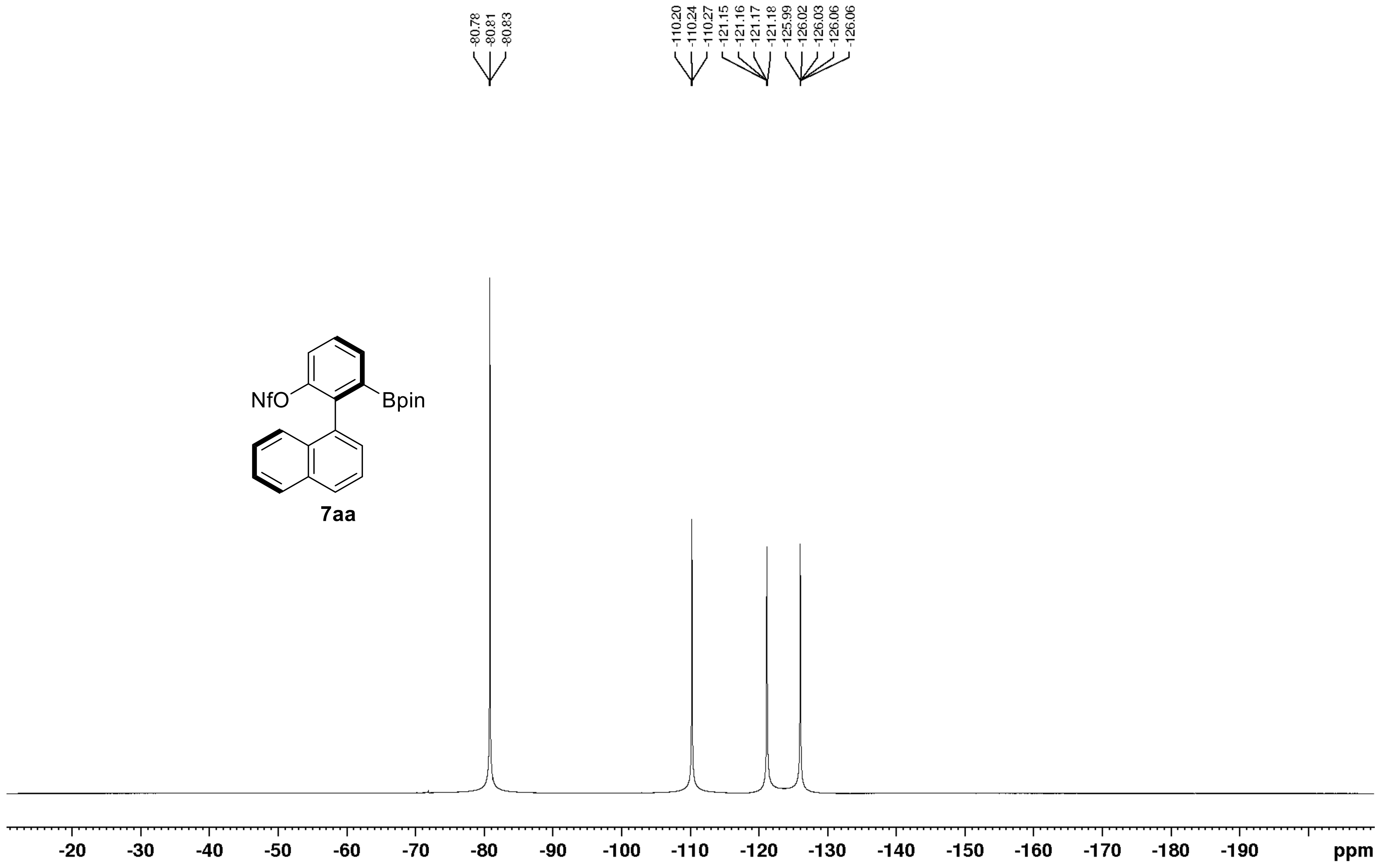

Figure S142. **^{11}B NMR** (160 MHz, $CDCl_3$, 298 K) of **7aa**

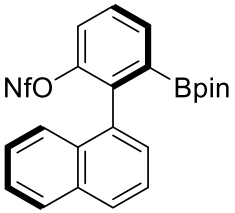

7aa

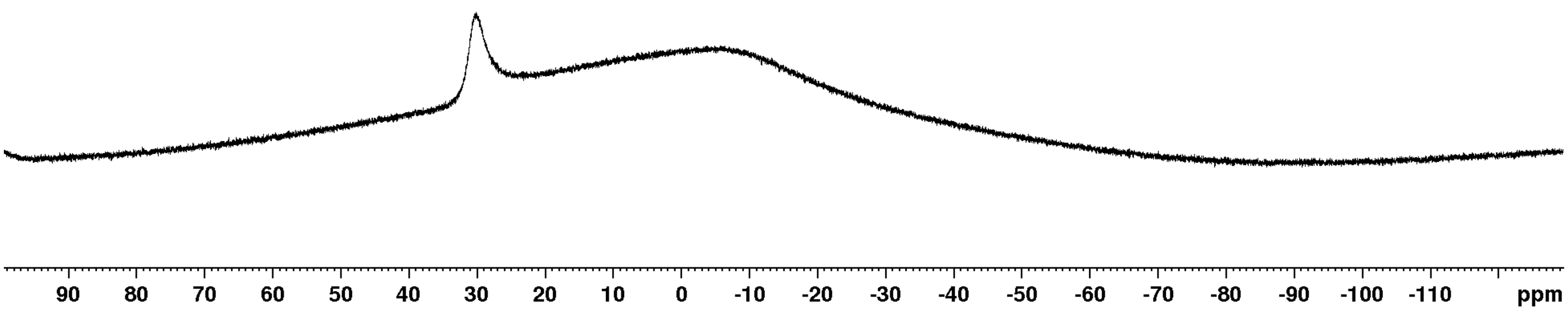

(*R*)-2-(4-methylnaphthalen-1-yl)-3-(4,4,5,5-tetramethyl-1,3,2-dioxaborolan-2-yl)phenyl 1,1,2,2,3,3,4,4,4-nonafluorobutane-1-sulfonate (7ba)

Figure S143. ^{1}H NMR (400 MHz, $CDCl_3$, 298 K) of **7ba**

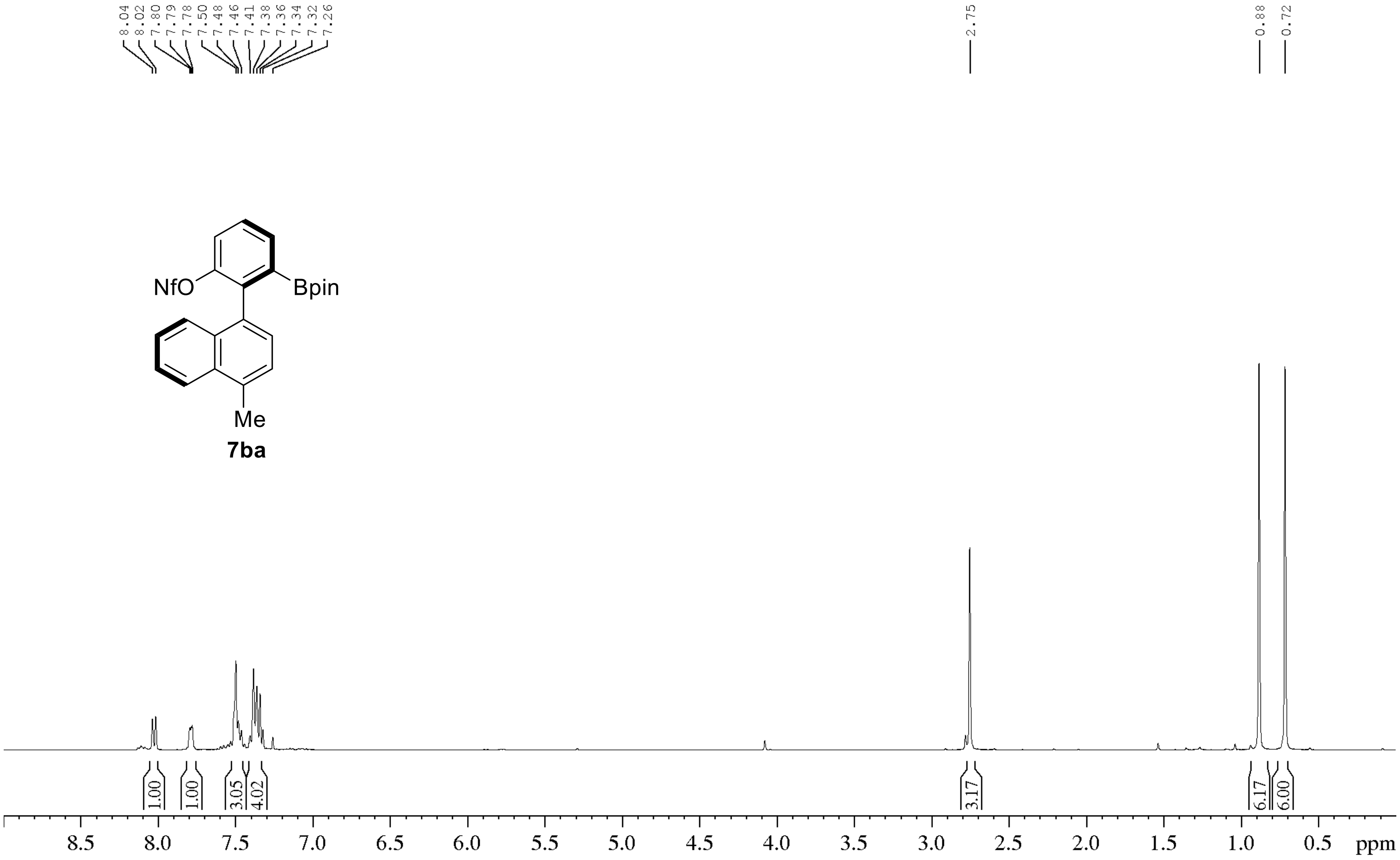

Figure S144. ^{13}C{^{1}H} NMR (101 MHz, $CDCl_3$, 298 K) of **7ba**

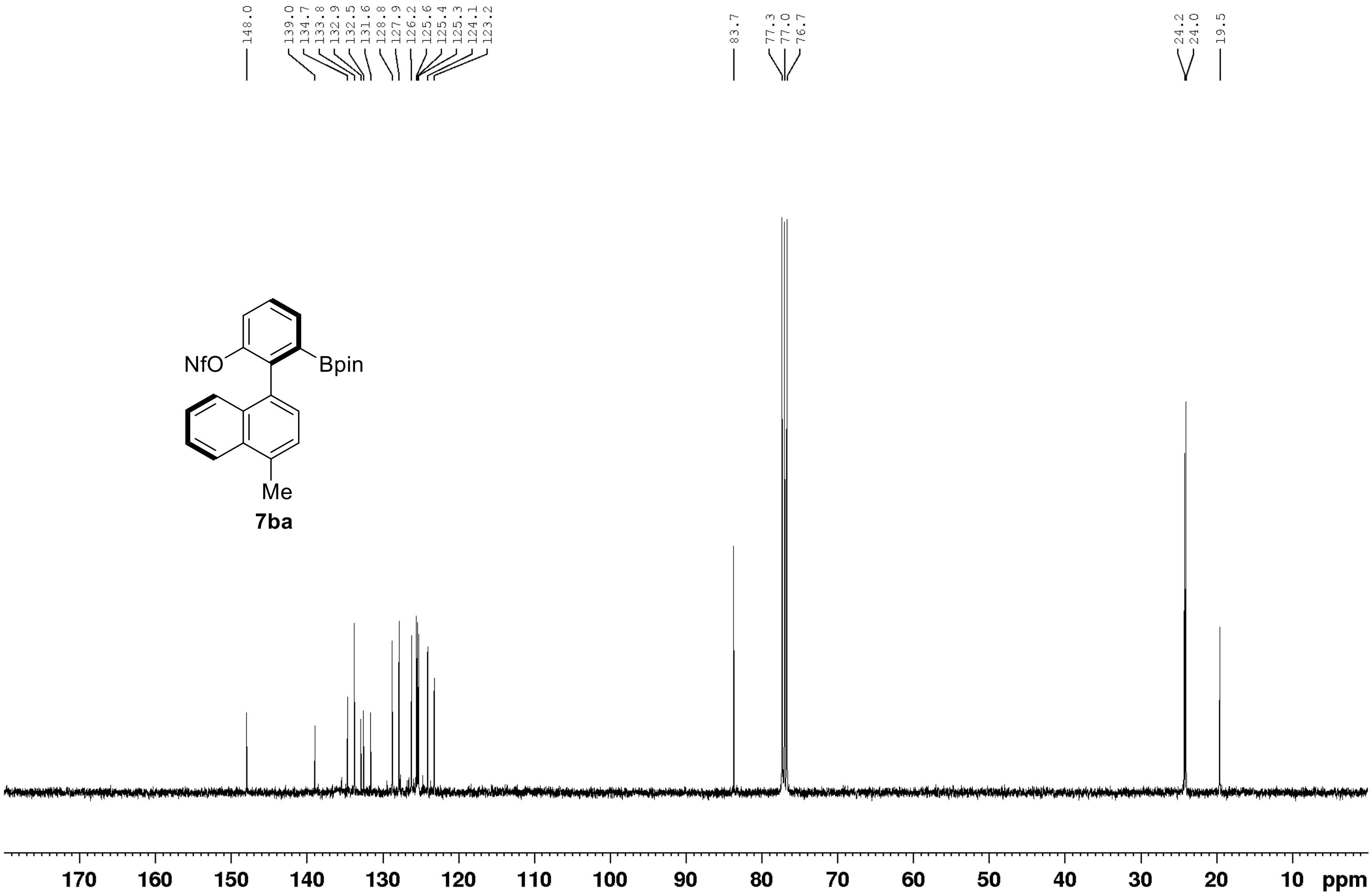

Figure S145. ^{19}**F NMR** (471 MHz, $CDCl_3$, 298 K) of **7ba**

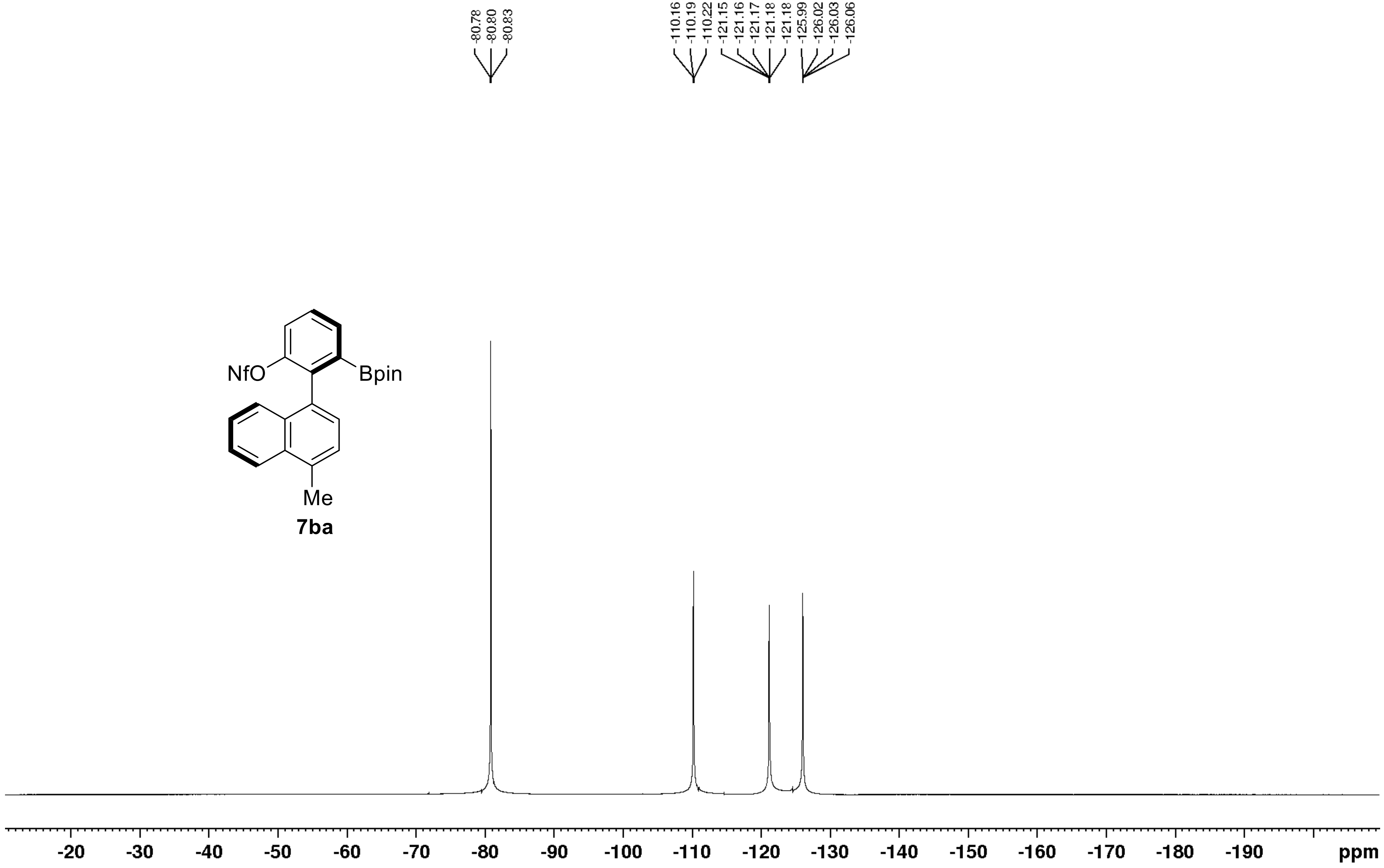

Figure S146. ^{11}B NMR (160 MHz, $CDCl_3$, 298 K) of **7ba**

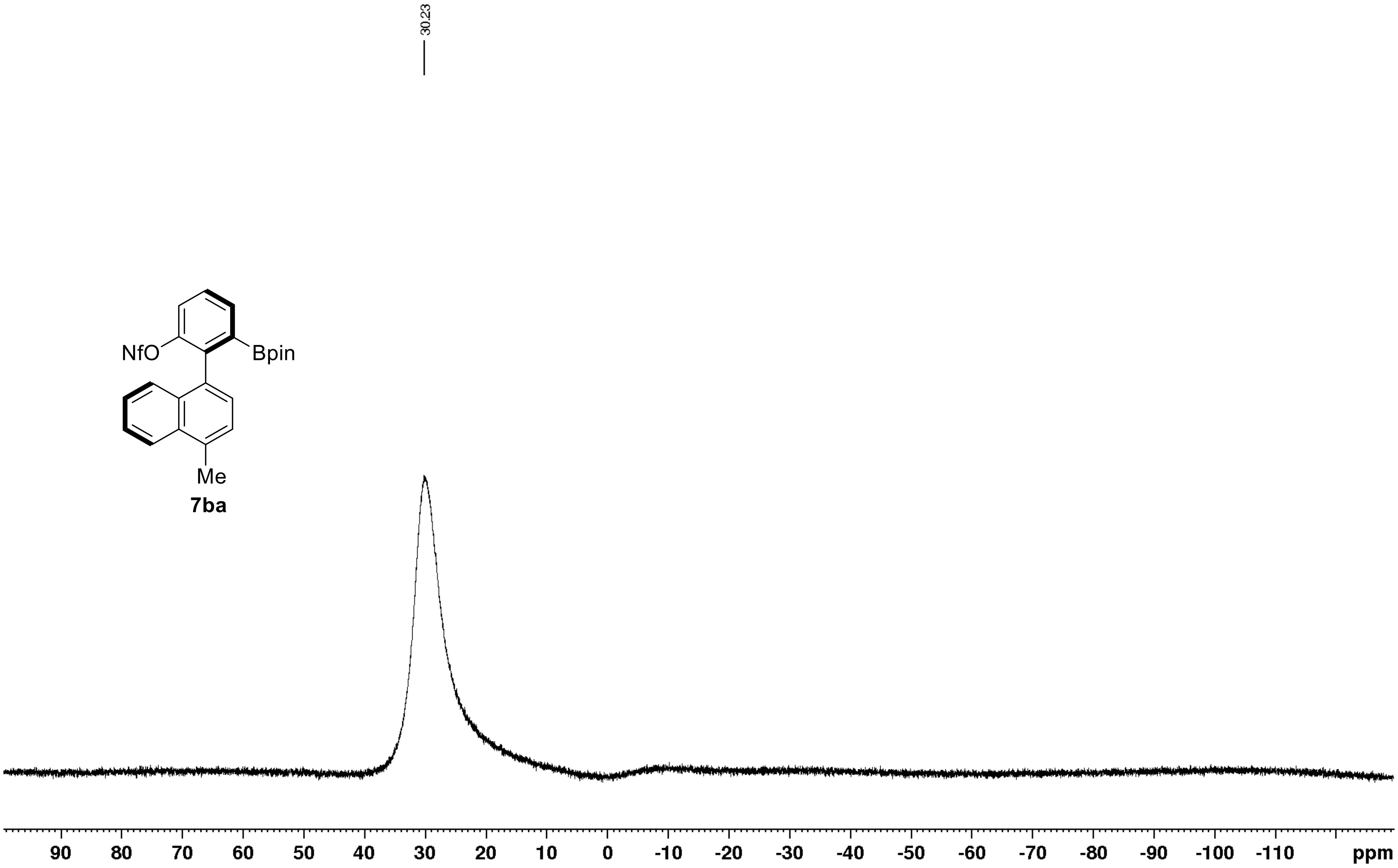

(*R*)-2-(4-fluoronaphthalen-1-yl)-3-(4,4,5,5-tetramethyl-1,3,2-dioxaborolan-2-yl)phenyl 1,1,2,2,3,3,4,4,4-nonafluorobutane-1-sulfonate (7ca)

Figure S147. ^{1}H NMR (500 MHz, $CDCl_3$, 298 K) of **7ca**

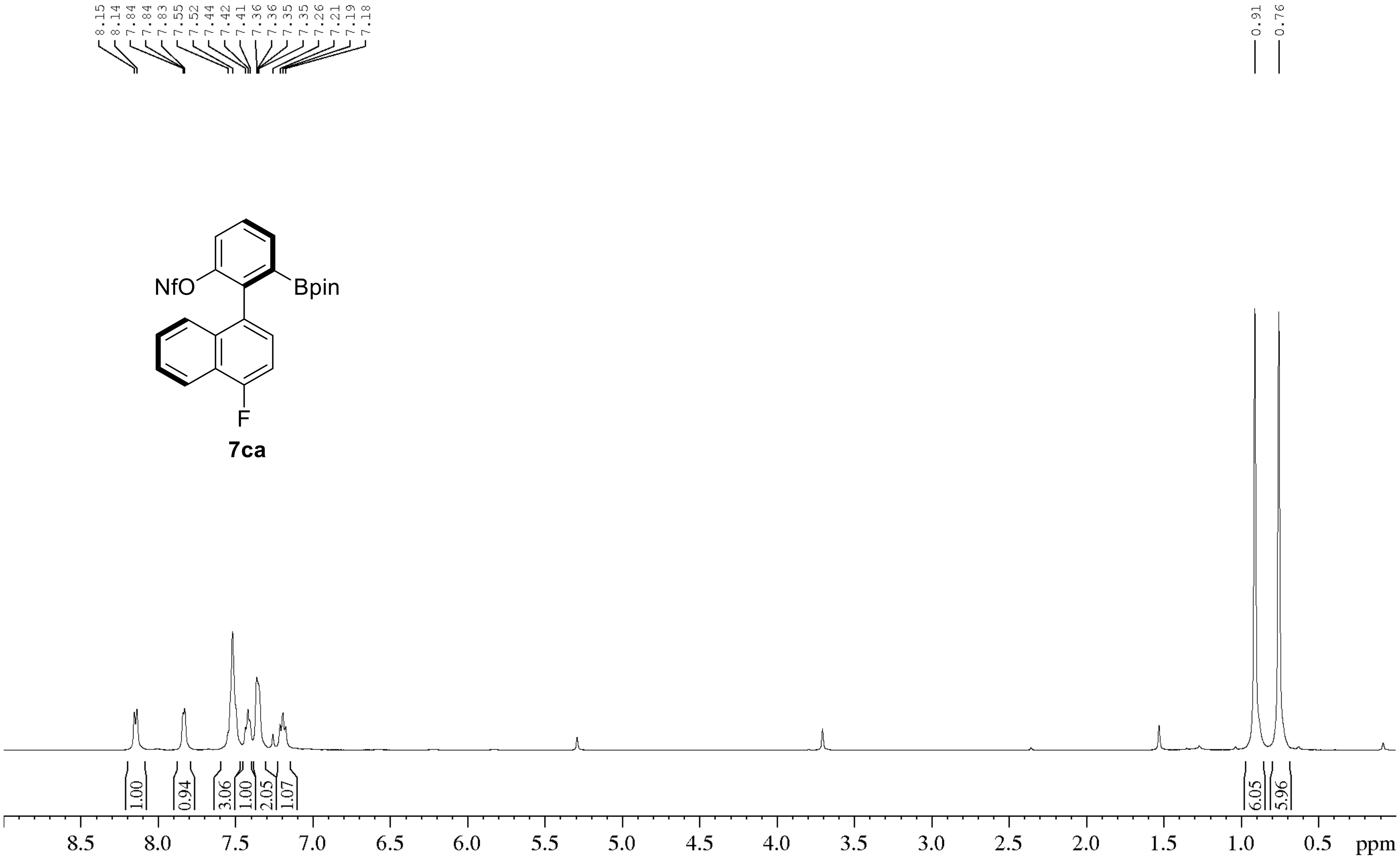

Figure S148. **$^{13}C\{^{1}H\}$ NMR** (101 MHz, $CDCl_3$, 298 K) of **7ca**

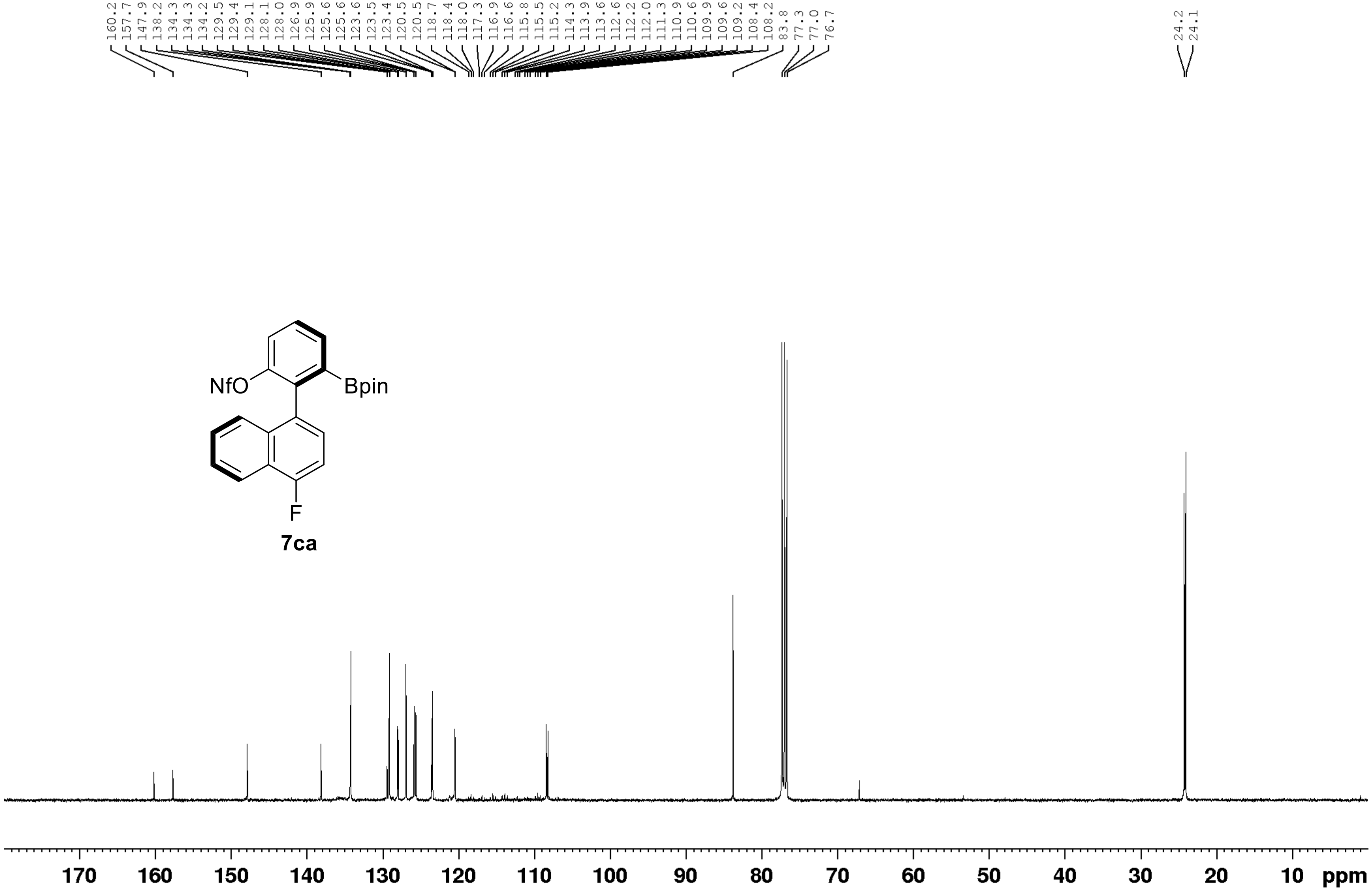

Figure S149. ^{19}F NMR (471 MHz, $CDCl_3$, 298 K) of **7ca**

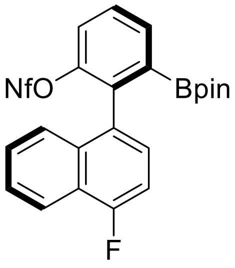

7ca

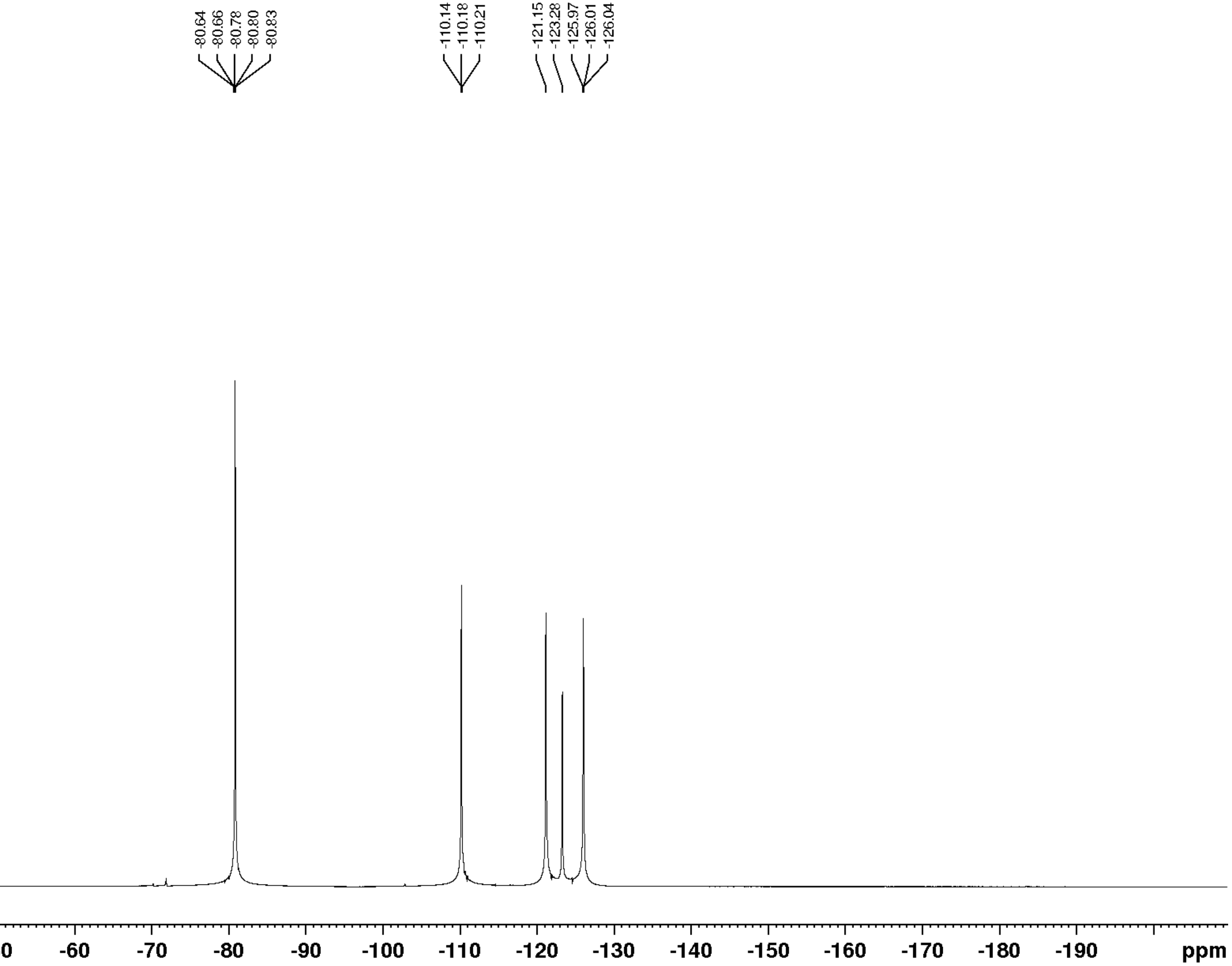

Figure S150. ^{11}**B NMR** (160 MHz, $CDCl_3$, 298 K) of **7ca**

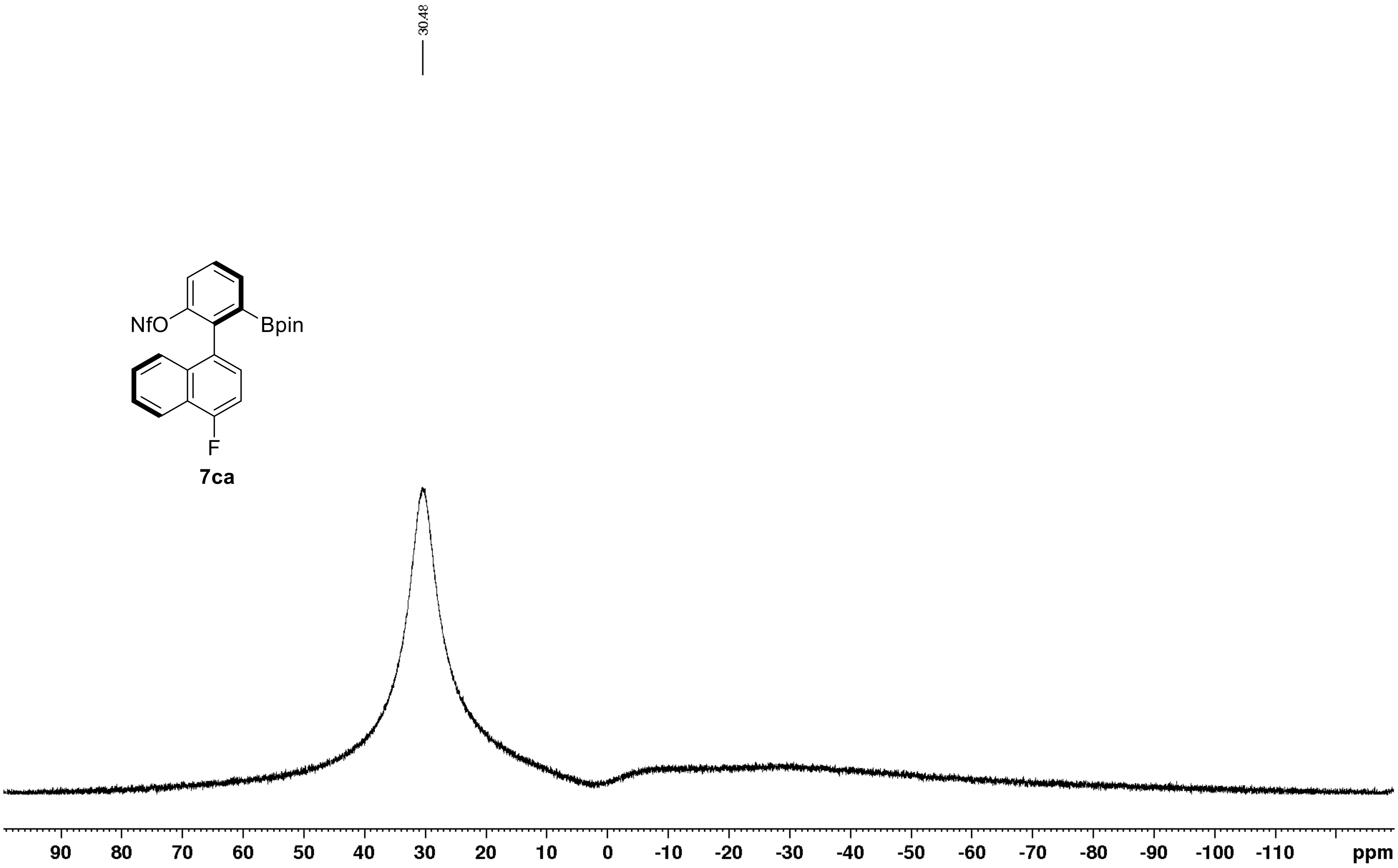

(*R*)-2-(4-bromonaphthalen-1-yl)-3-(4,4,5,5-tetramethyl-1,3,2-dioxaborolan-2-yl)phenyl 1,1,2,2,3,3,4,4,4-nonafluorobutane-1-sulfonate (7da)

Figure S151. ^{1}H NMR (500 MHz, $CDCl_3$, 298 K) of **7da**

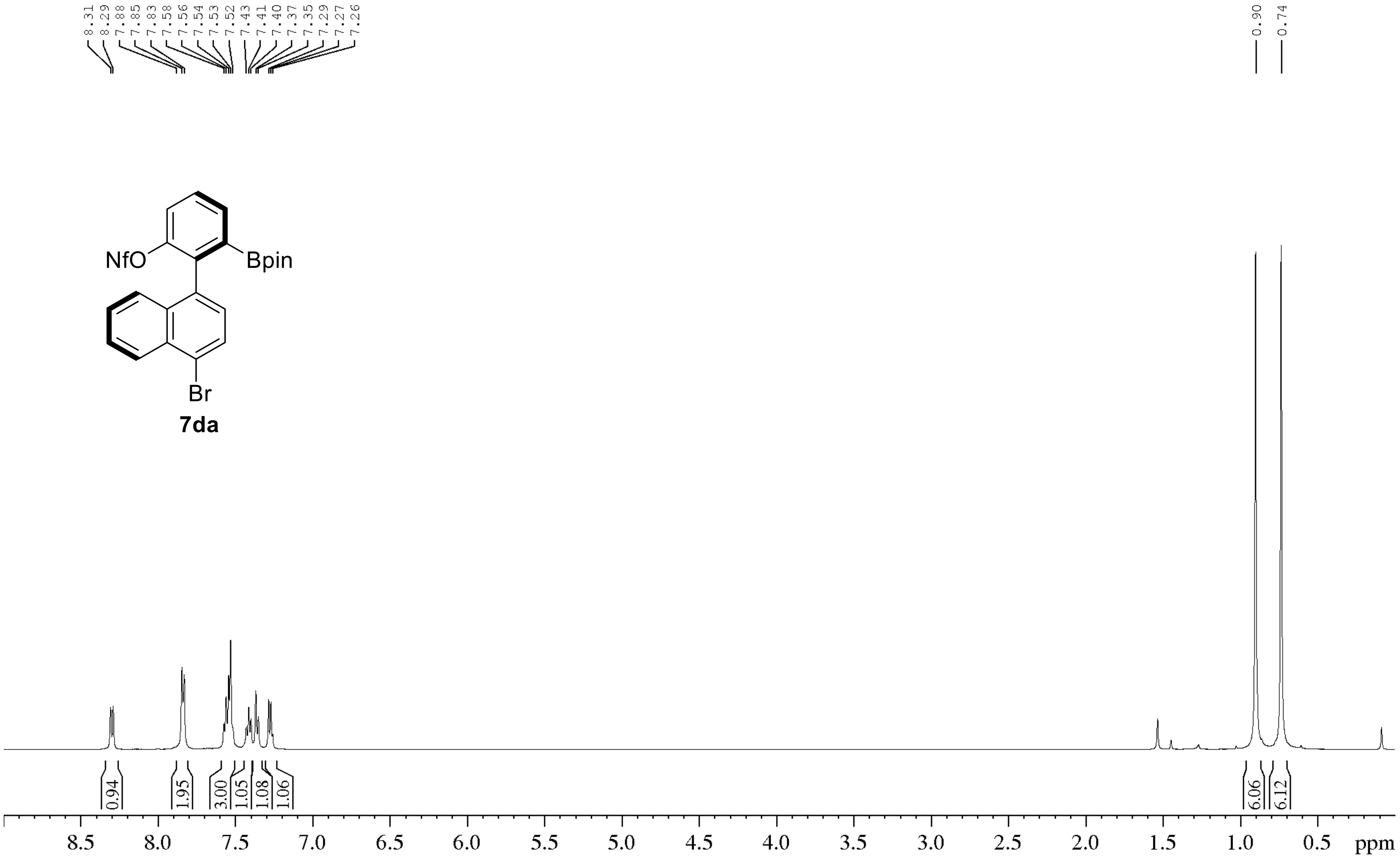

Figure S152. ^{13}**C{**^{1}**H} NMR** (126 MHz, $CDCl_3$, 298 K) of **7da**

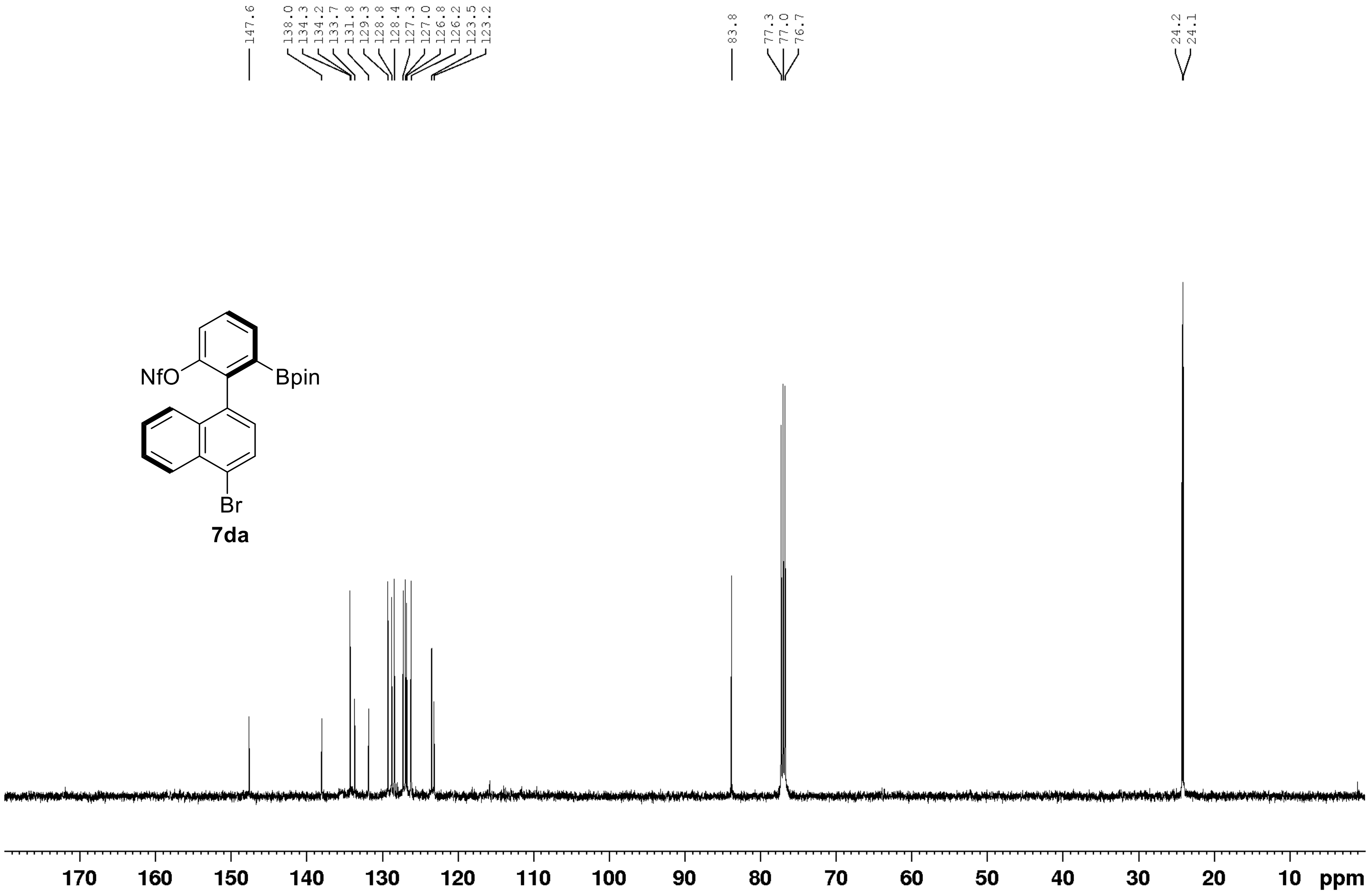

Figure S153. ^{19}**F NMR** (471 MHz, $CDCl_3$, 298 K) of **7da**

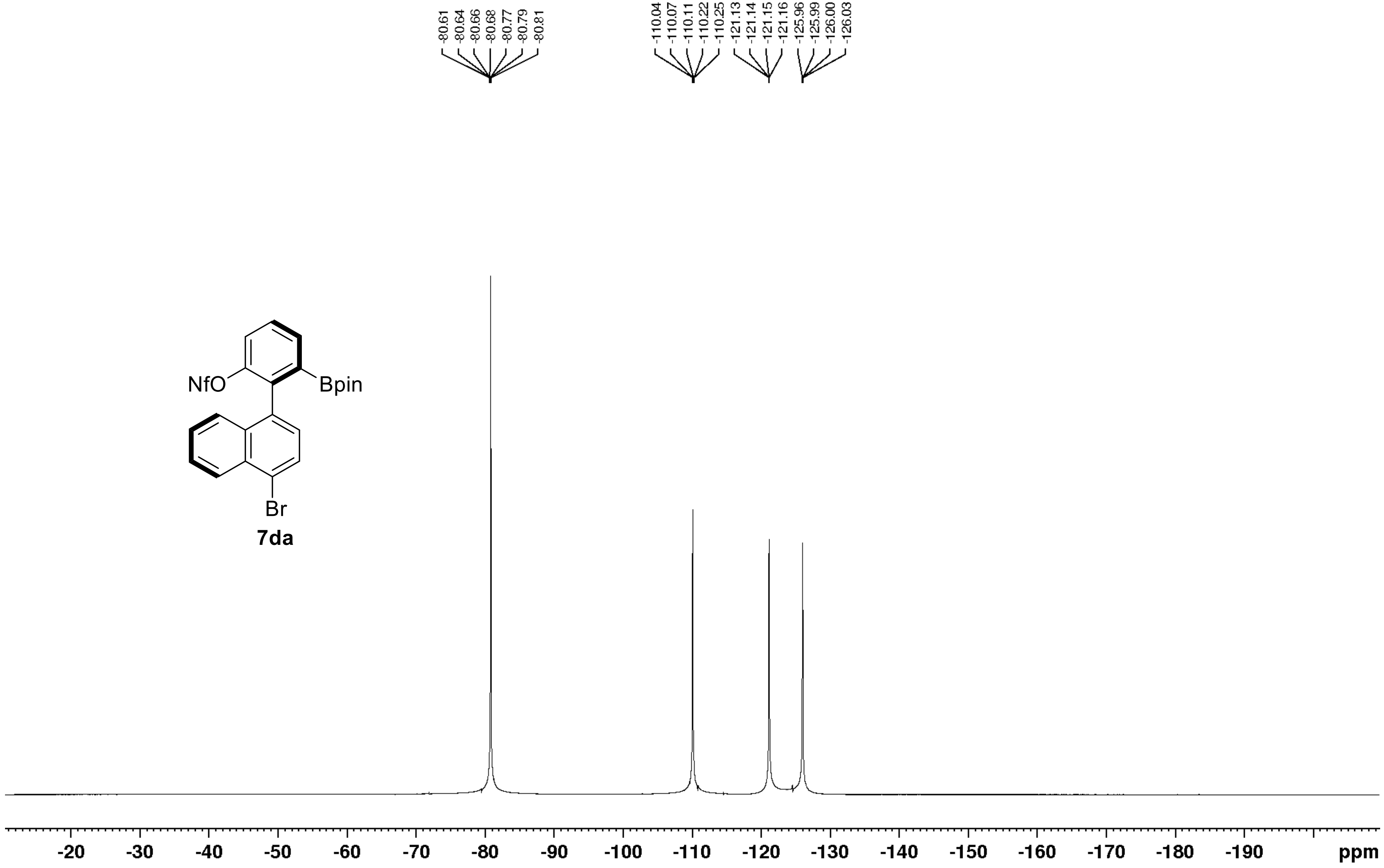

Figure S154. ^{11}**B NMR** (160 MHz, $CDCl_3$, 298 K) of **7da**

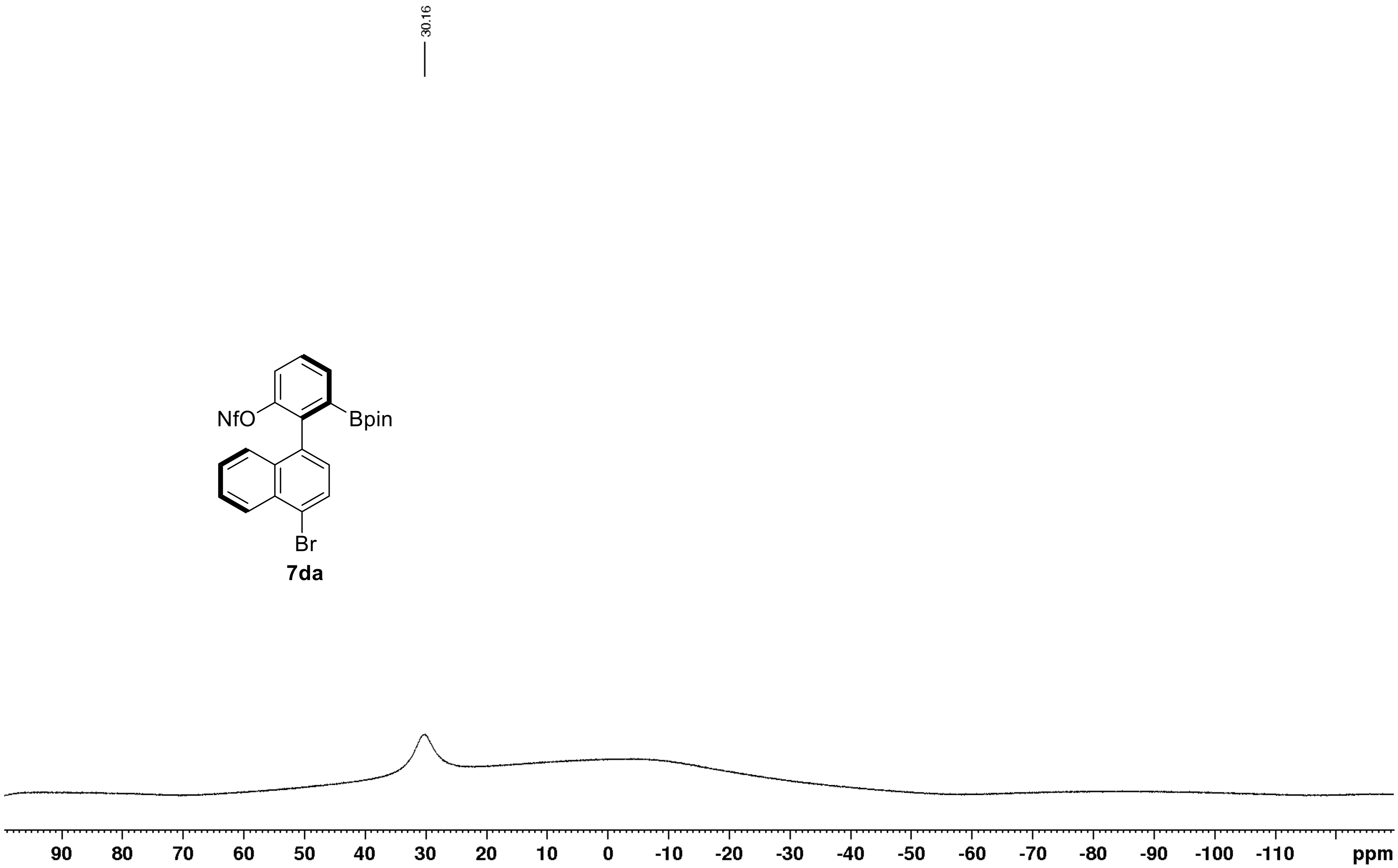

(*R*)-2-(4-cyanonaphthalen-1-yl)-3-(4,4,5,5-tetramethyl-1,3,2-dioxaborolan-2-yl)phenyl 1,1,2,2,3,3,4,4,4-nonafluorobutane-1-sulfonate (**7ea**)

Figure S155. ^{1}H NMR (500 MHz, $CDCl_3$, 298 K) of **7ea**

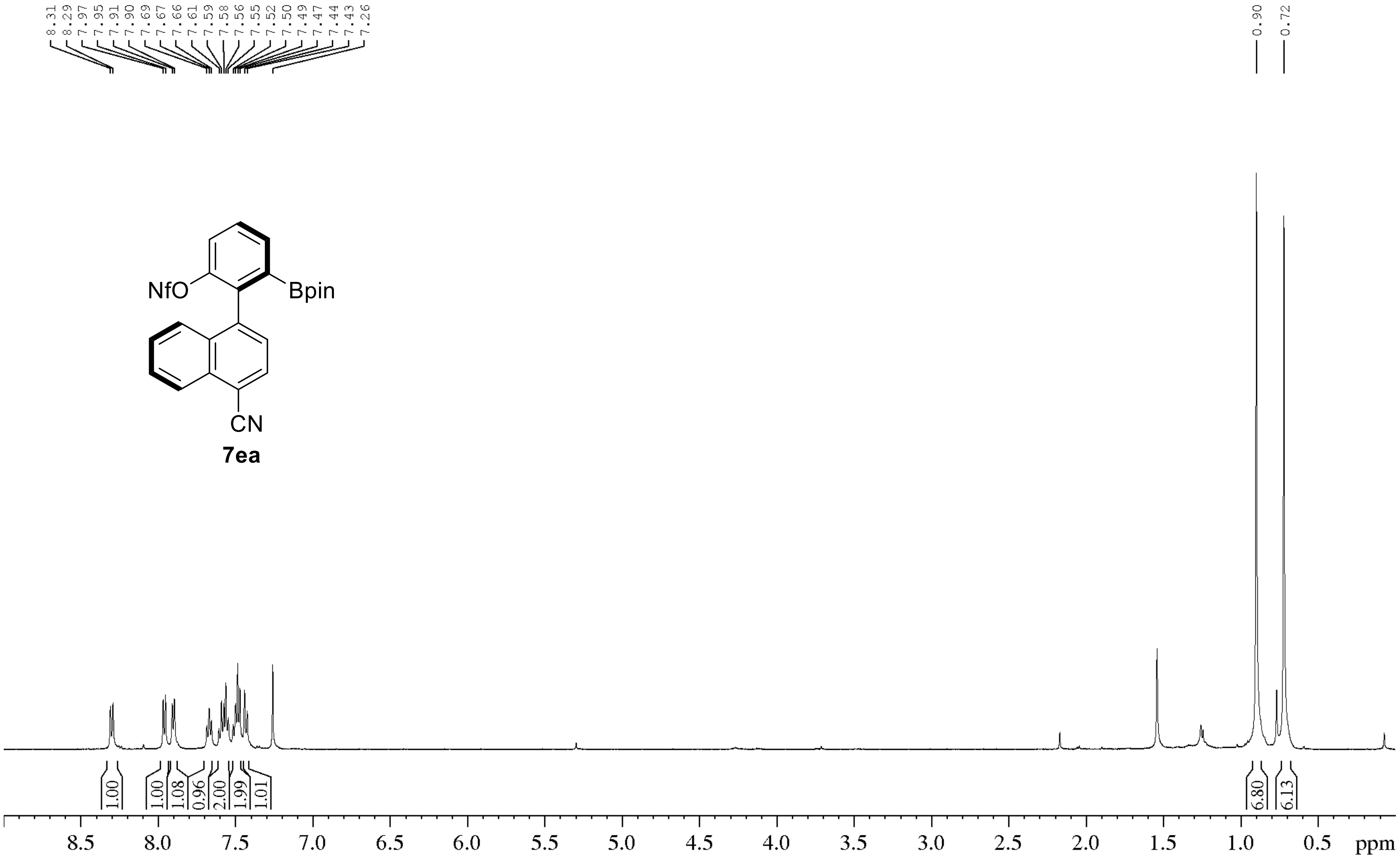

Figure S156. **$^{13}C\{^{1}H\}$ NMR** (101 MHz, $CDCl_3$, 298 K) of **7ea**

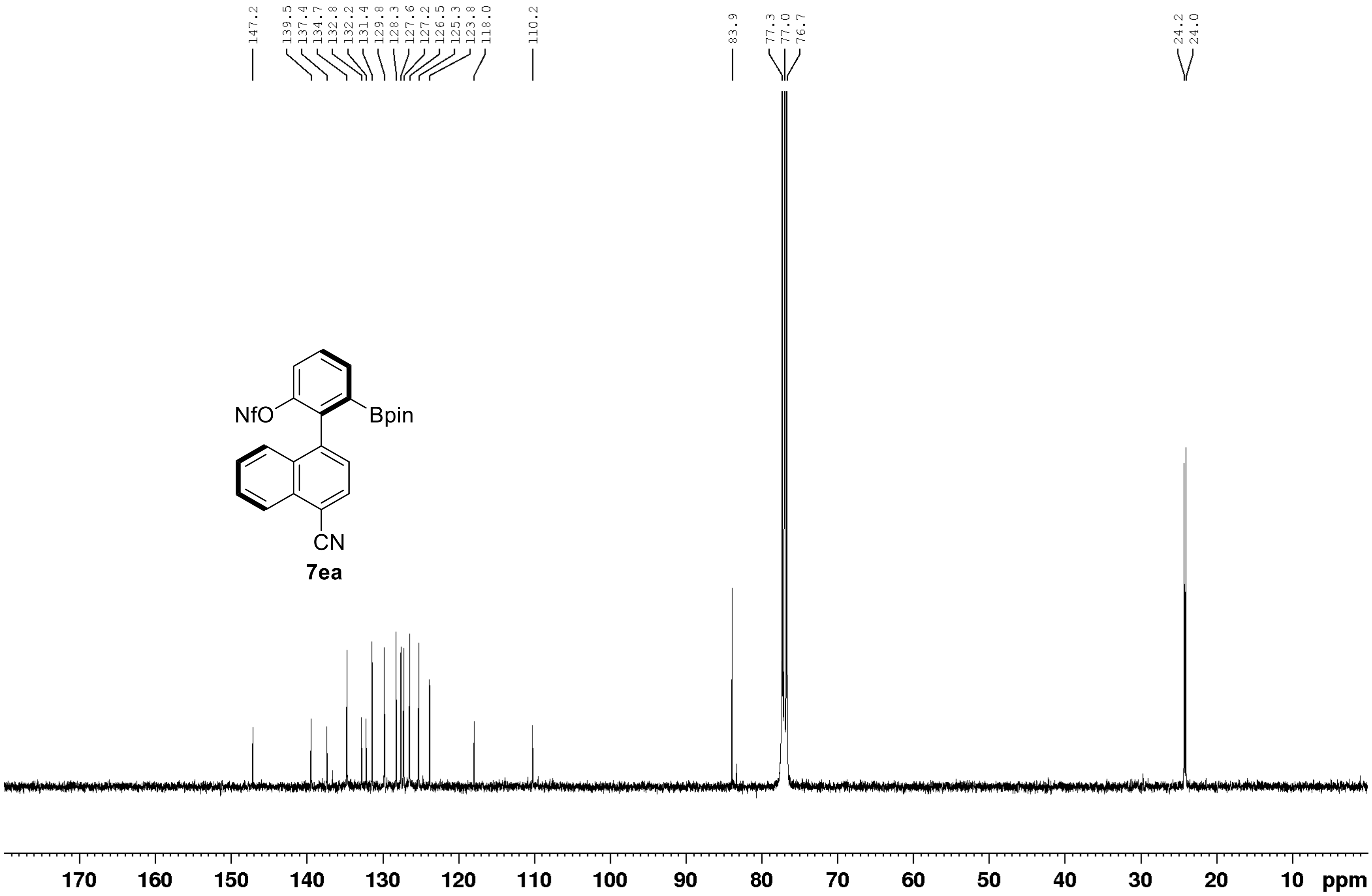

Figure S157. **^{19}F NMR** (471 MHz, $CDCl_3$, 298 K) of **7ea**

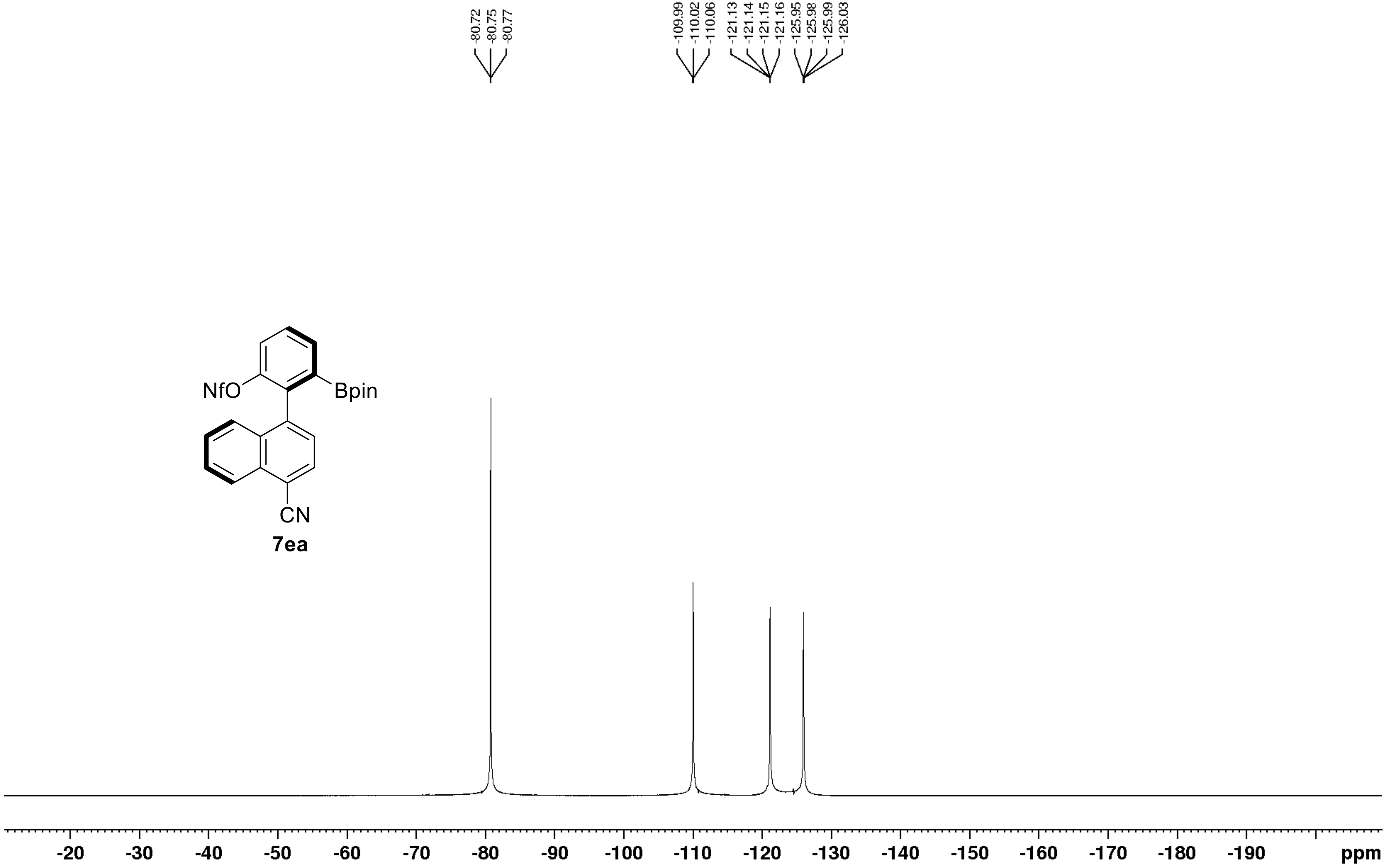

Figure S158. ^{11}B NMR (160 MHz, $CDCl_3$, 298 K) of **7ea**

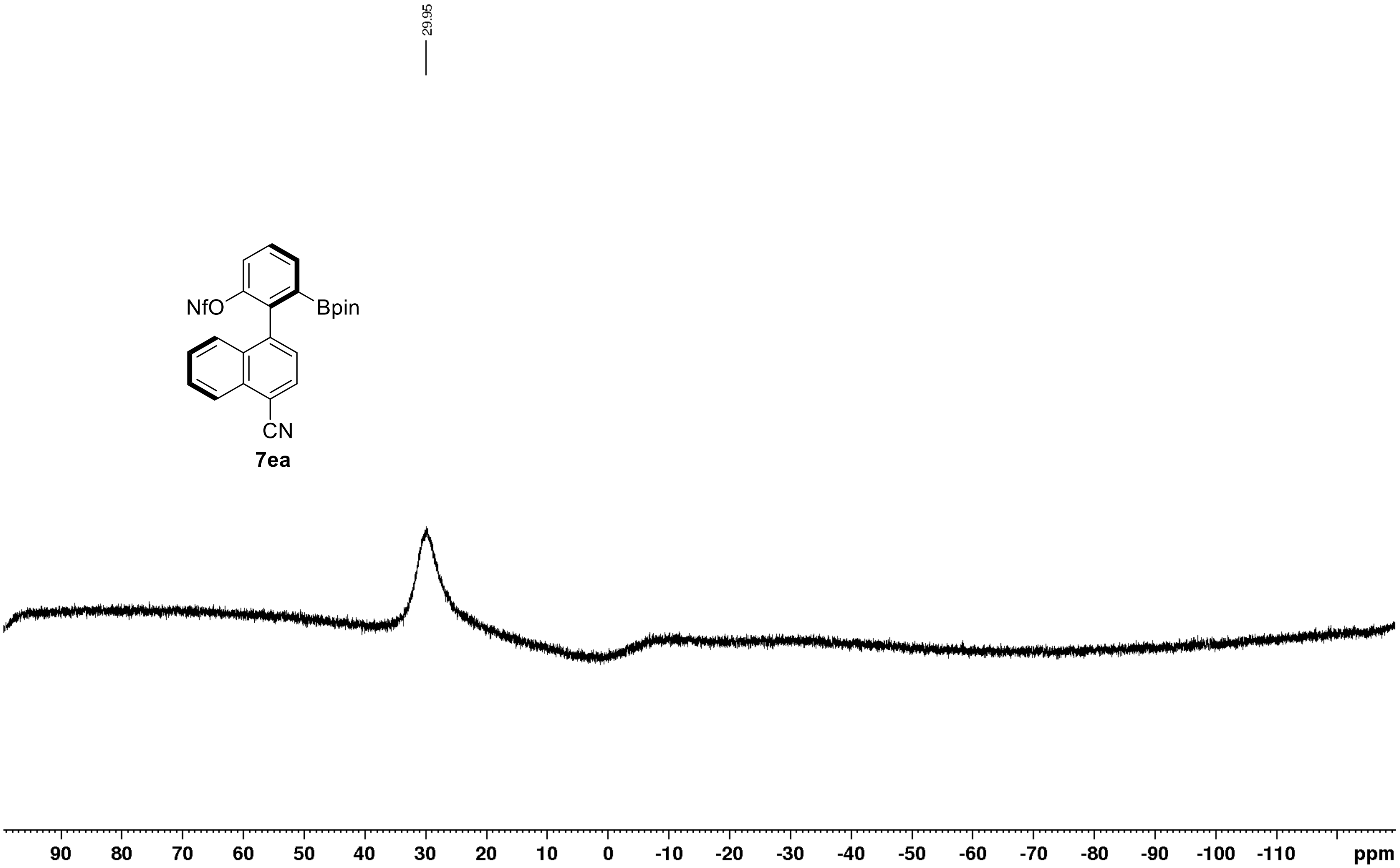

(*R*)-2-(5-phenylnaphthalen-1-yl)-3-(4,4,5,5-tetramethyl-1,3,2-dioxaborolan-2-yl)phenyl 1,1,2,2,3,3,4,4,4-nonafluorobutane-1-sulfonate (**7fa**)

Figure S159. **^{1}H NMR** (400 MHz, $CDCl_3$, 298 K) of **7fa**

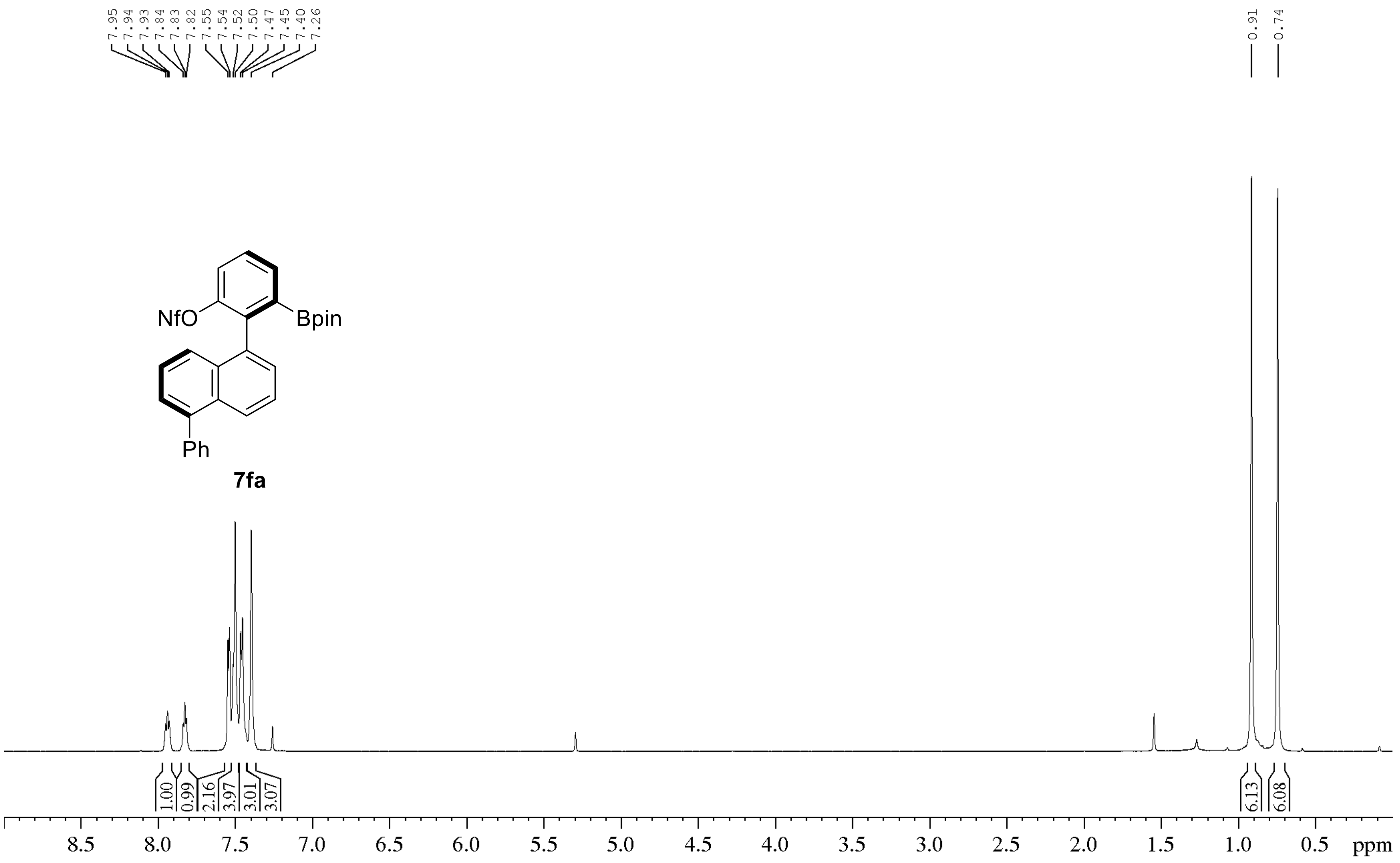

Figure S160. $^{13}C\{^1H\}$ **NMR** (101 MHz, $CDCl_3$, 298 K) of **7fa**

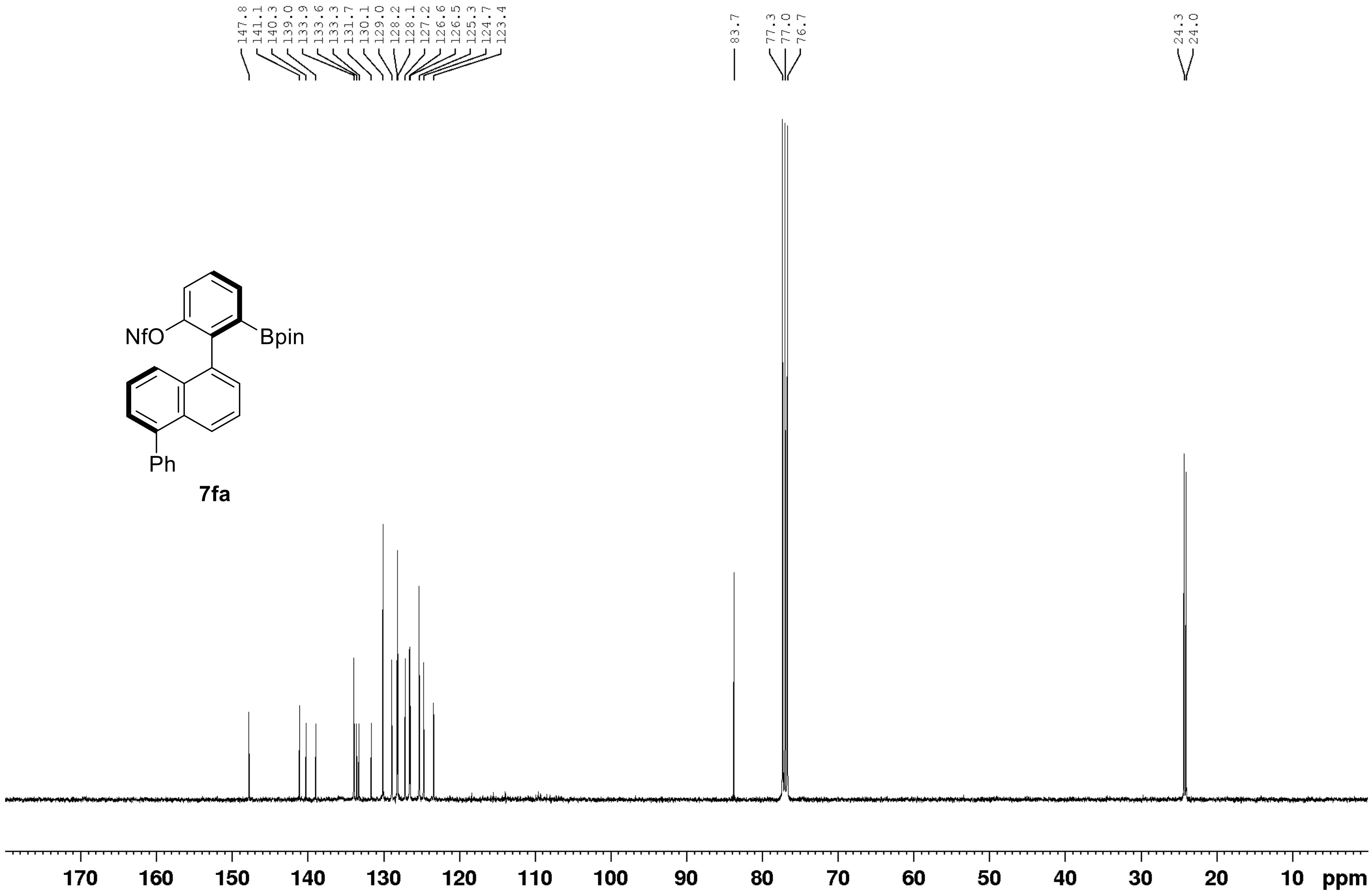

Figure S161. ^{19}F NMR (471 MHz, $CDCl_3$, 298 K) of **7fa**

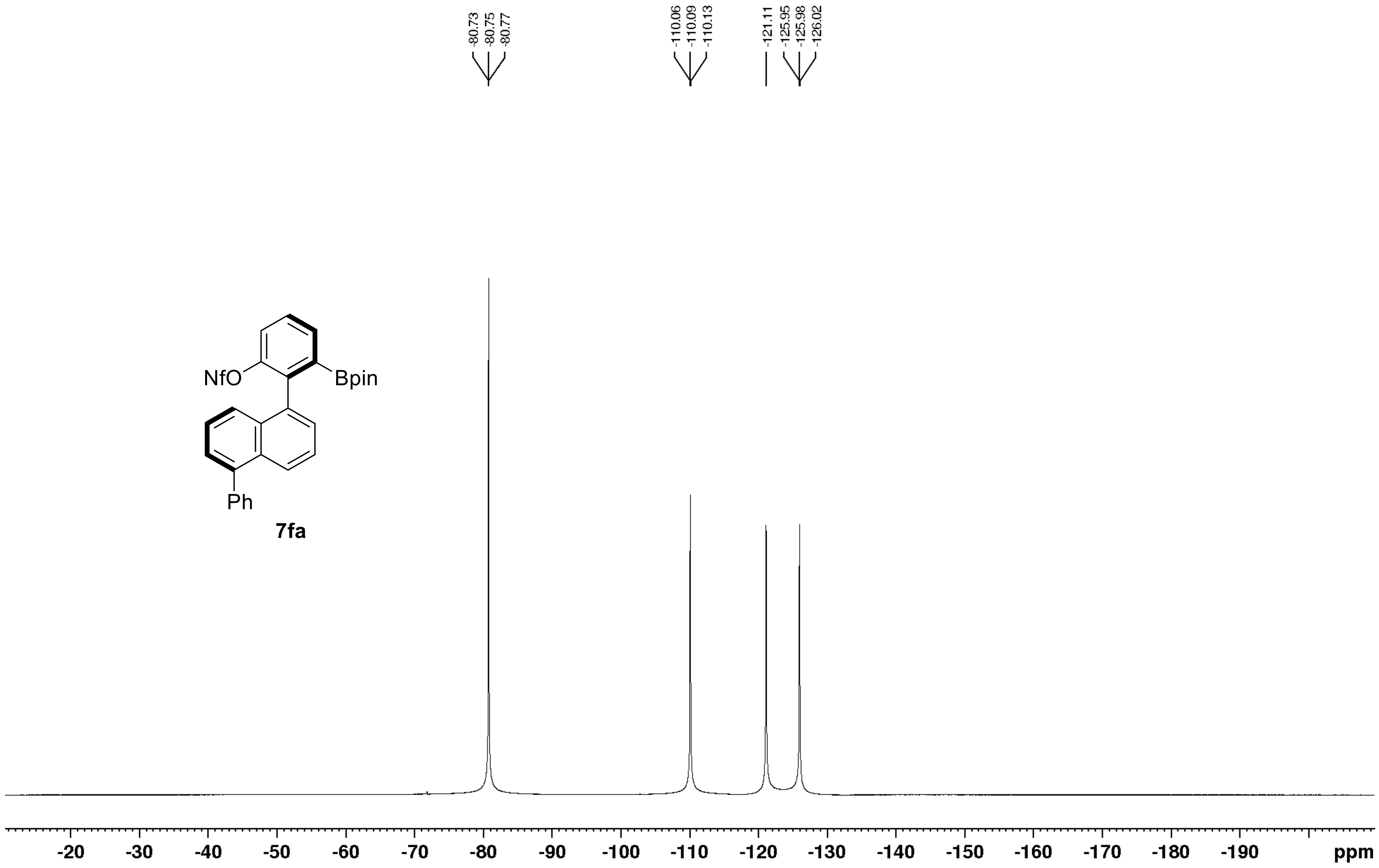

Figure S162. ^{11}B NMR (160 MHz, $CDCl_3$, 298 K) of **7fa**

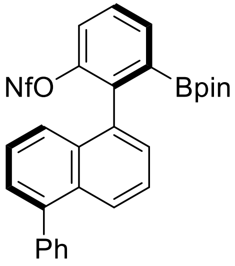

7fa

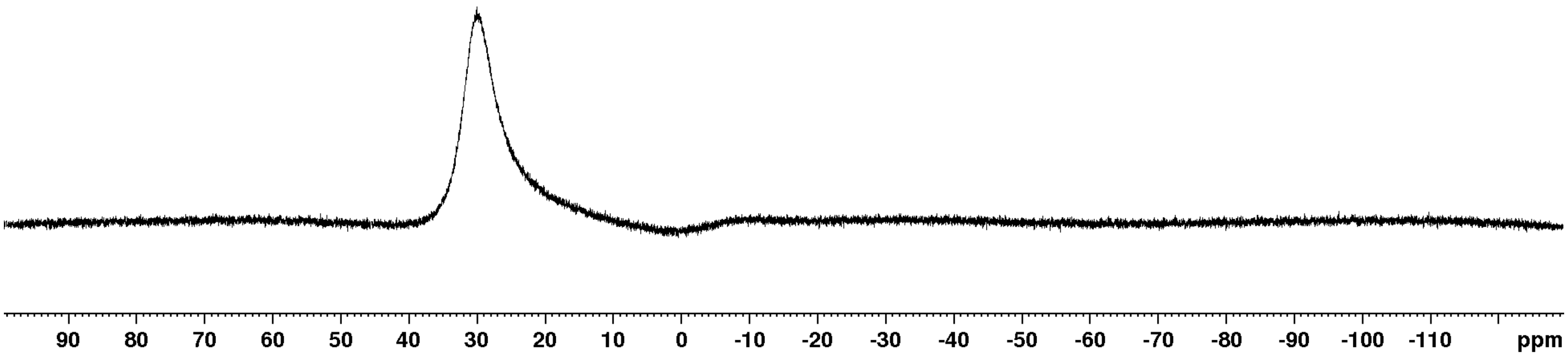

(*R*)-2-(5-((4-methylphenyl)sulfonamido)naphthalen-1-yl)-3-(4,4,5,5-tetramethyl-1,3,2-dioxaborolan-2-yl)phenyl 1,1,2,2,3,3,4,4,4-nonafluorobutane-1-sulfonate (**7ga**)

Figure S163. ^{1}H NMR (500 MHz, $CDCl_3$, 298 K) of **7ga**

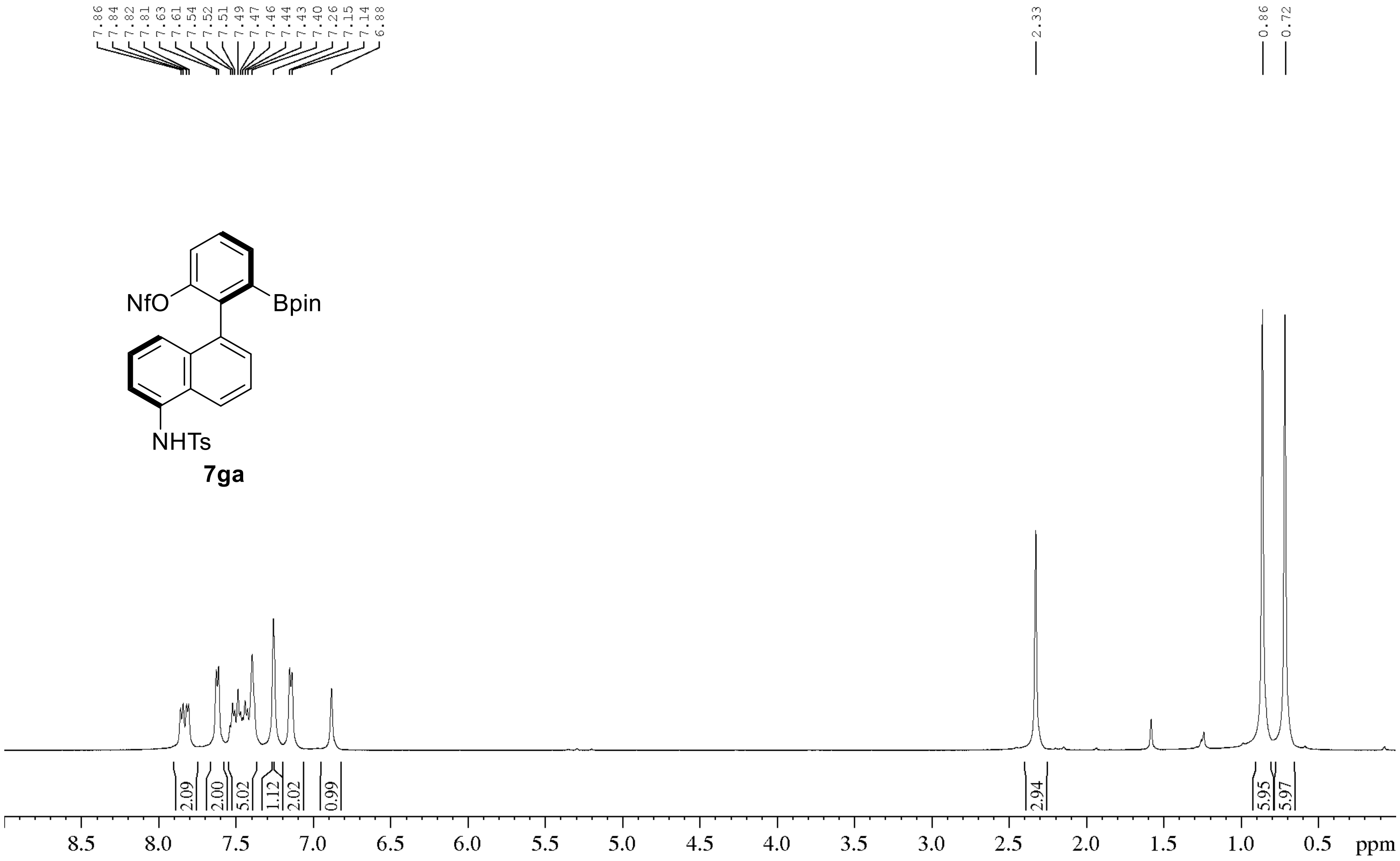

Figure S164. **^{13}C{^{1}H} NMR** (126 MHz, $CDCl_3$, 298 K) of **7ga**

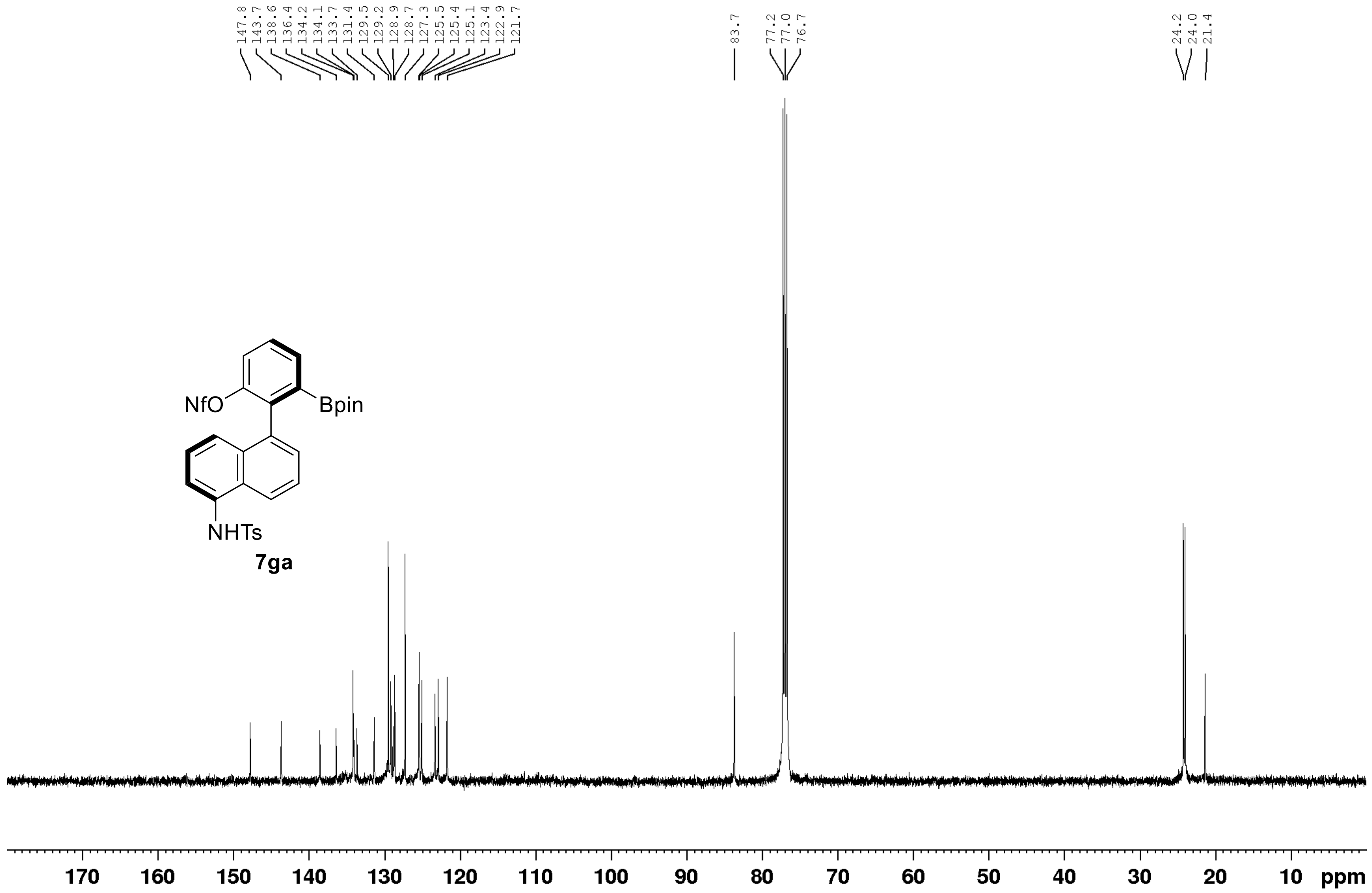

Figure S165. **^{19}F NMR** (471 MHz, $CDCl_3$, 298 K) of **7ga**

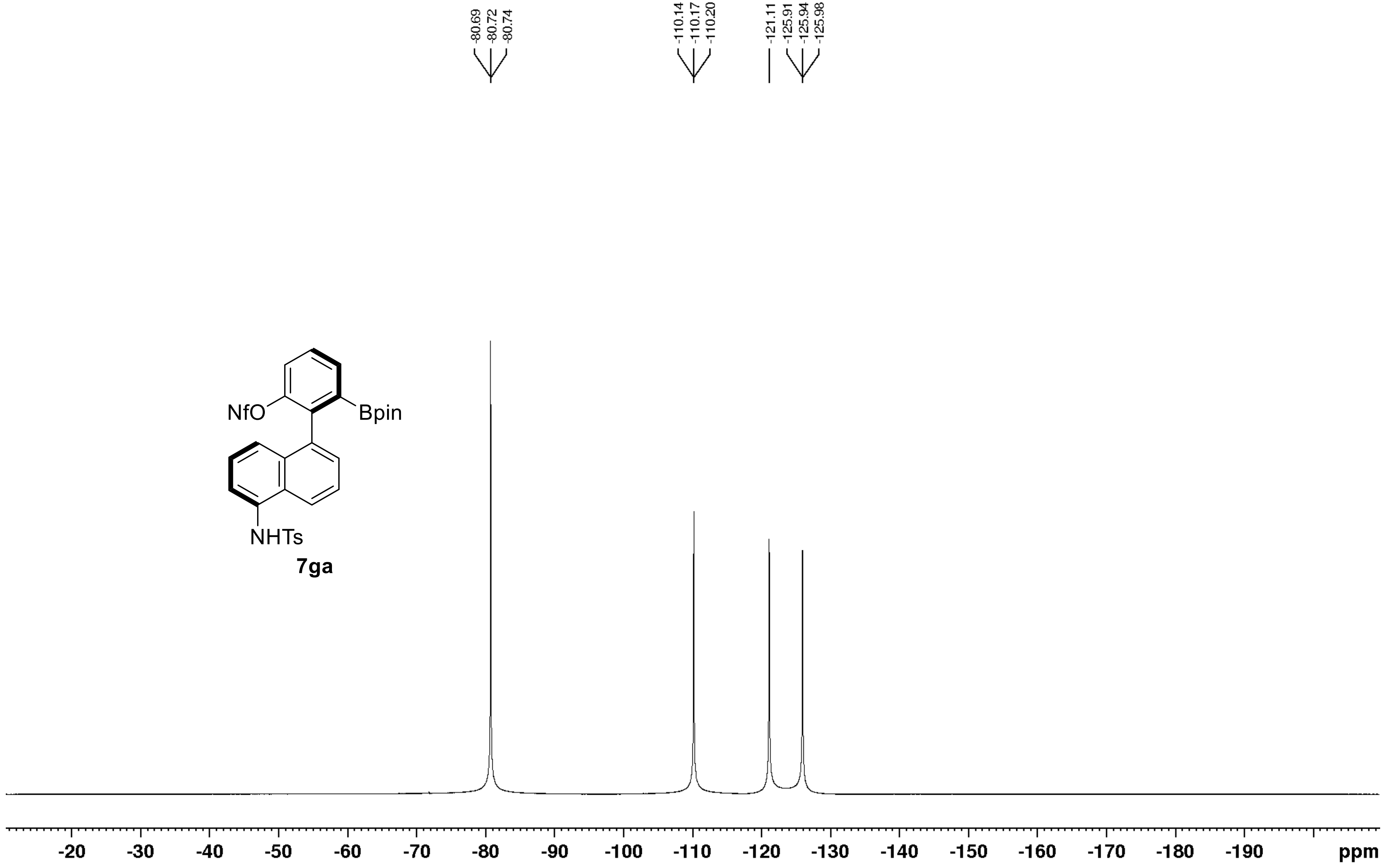

Figure S166. ^{11}B NMR (160 MHz, $CDCl_3$, 298 K) of **7ga**

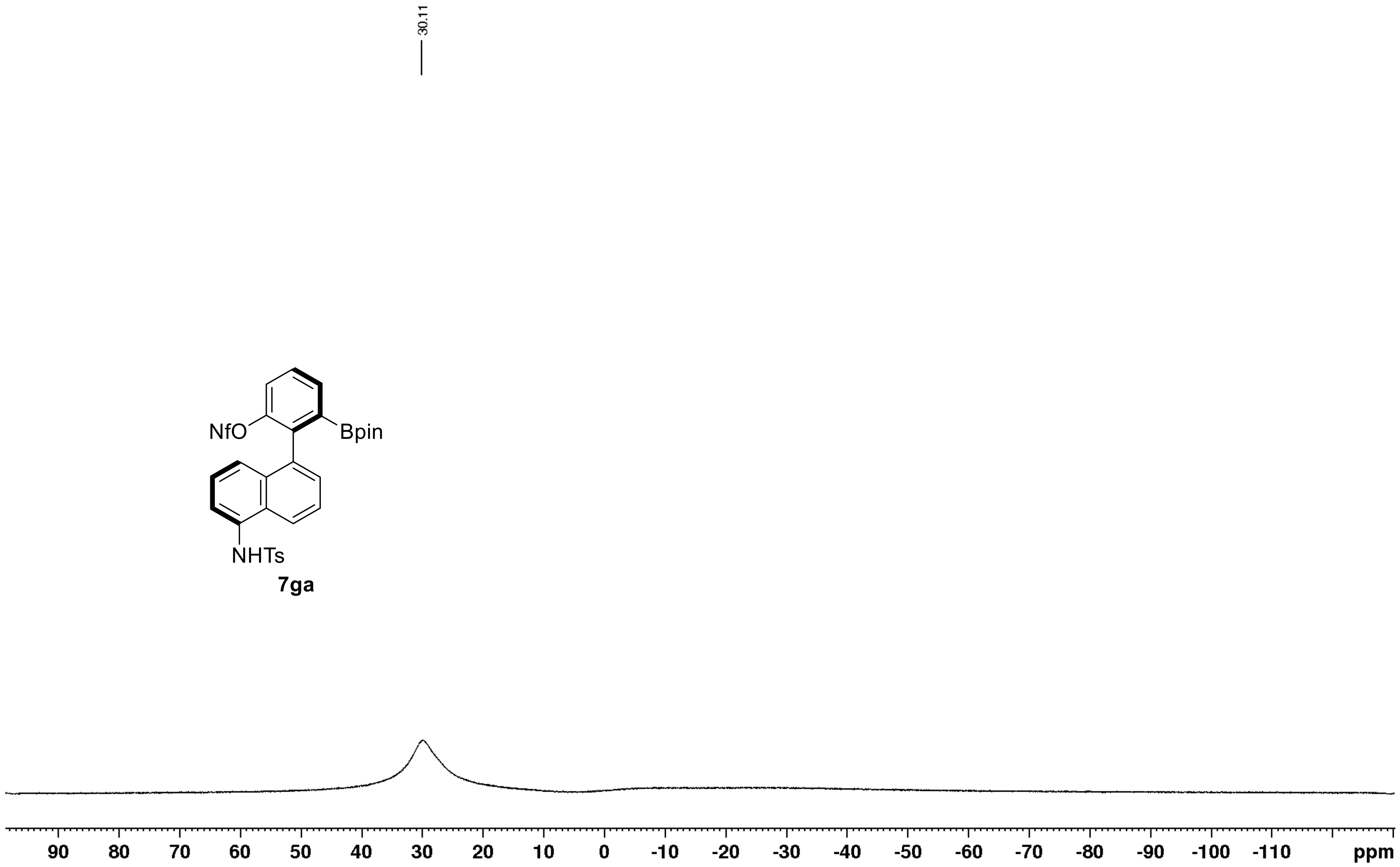

(*R*)-2-(2-methylnaphthalen-1-yl)-3-(4,4,5,5-tetramethyl-1,3,2-dioxaborolan-2-yl)phenyl 1,1,2,2,3,3,4,4,4-nonafluorobutane-1-sulfonate (**7ha**)

Figure S167. ^{1}H NMR (500 MHz, $CDCl_3$, 298 K) of **7ha**

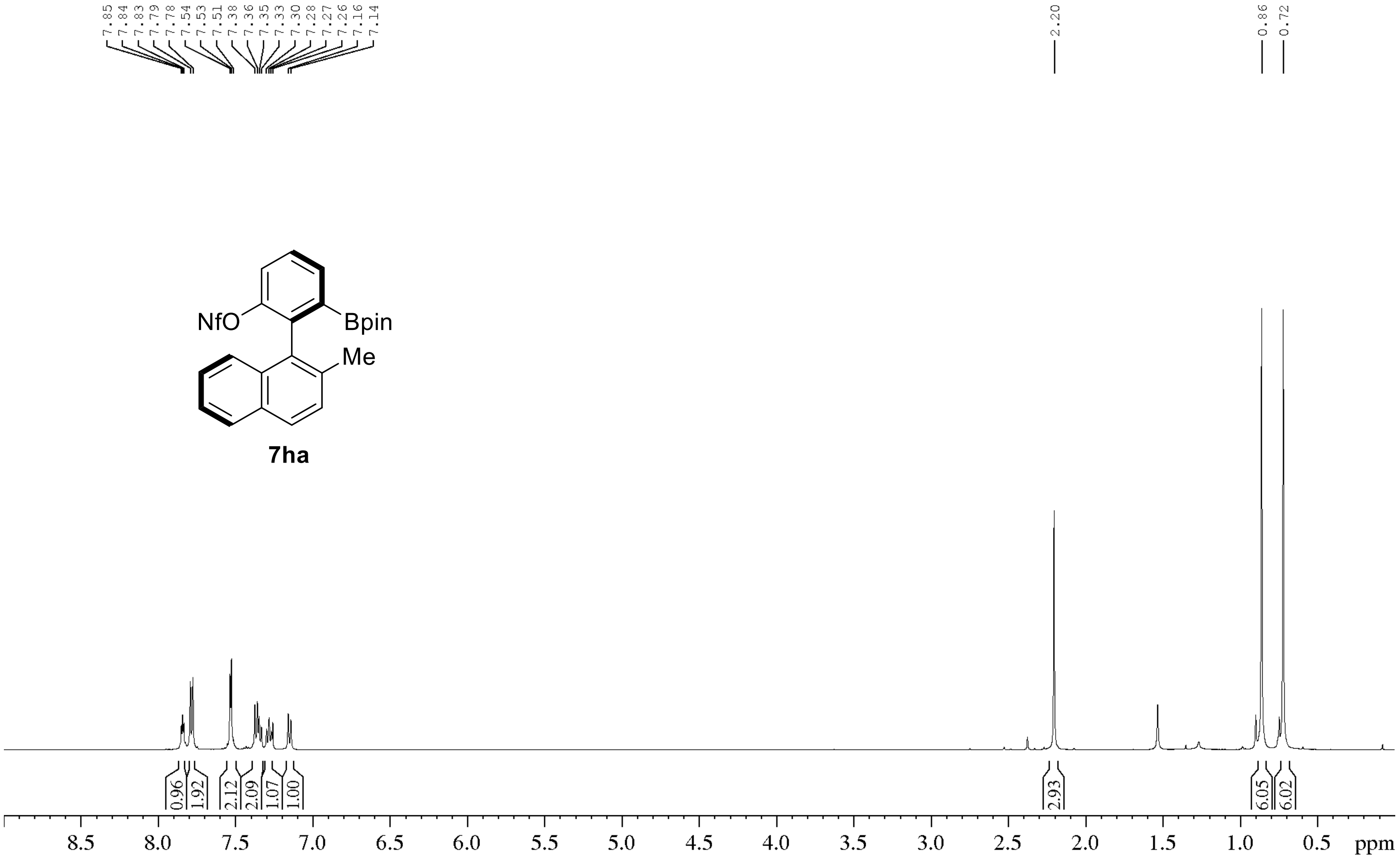

Figure S168. **^{13}C{^{1}H} NMR** (126 MHz, $CDCl_3$, 298 K) of **7ha**

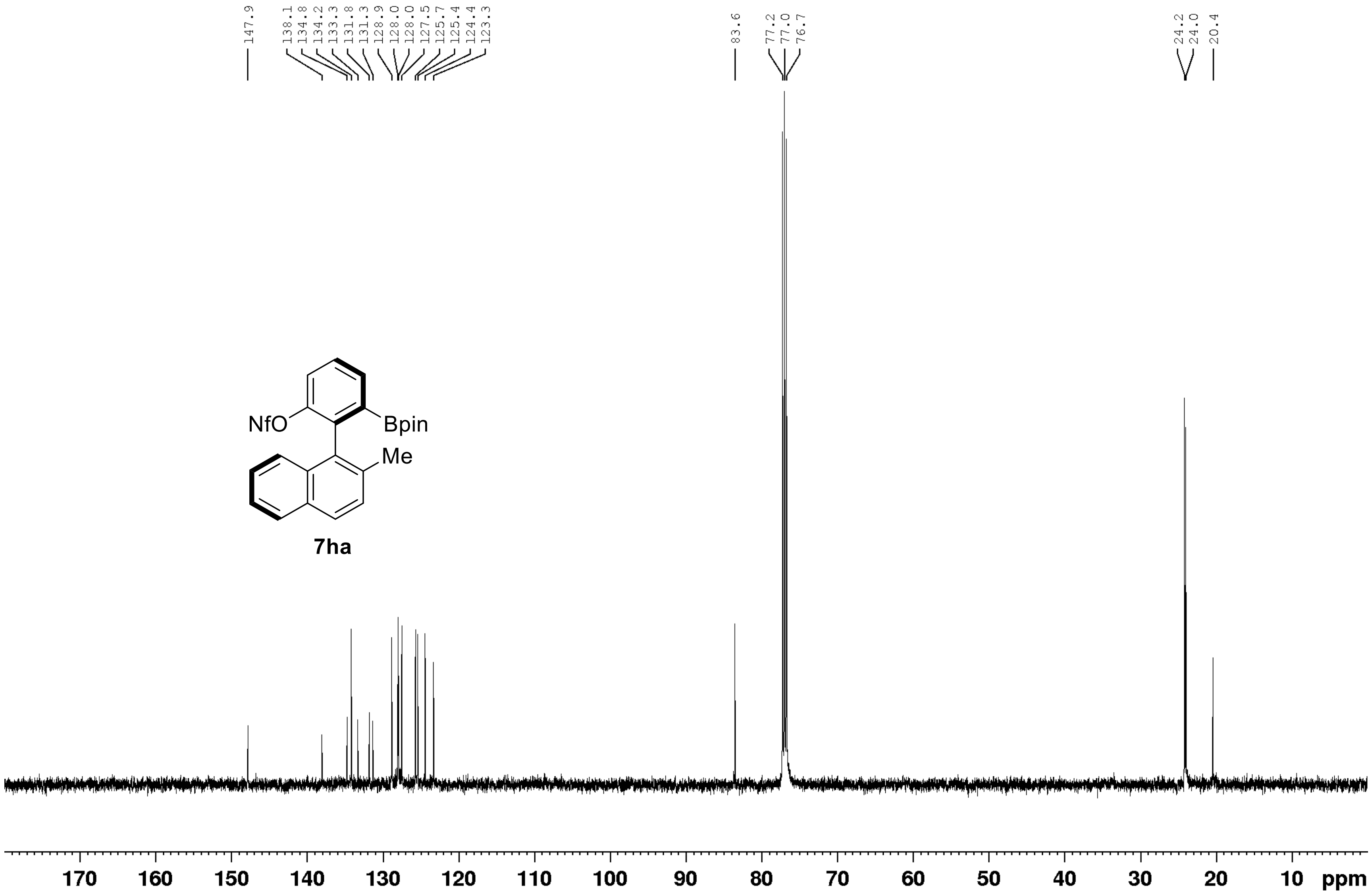

Figure S169. **^{19}F NMR** (471 MHz, $CDCl_3$, 298 K) of **7ha**

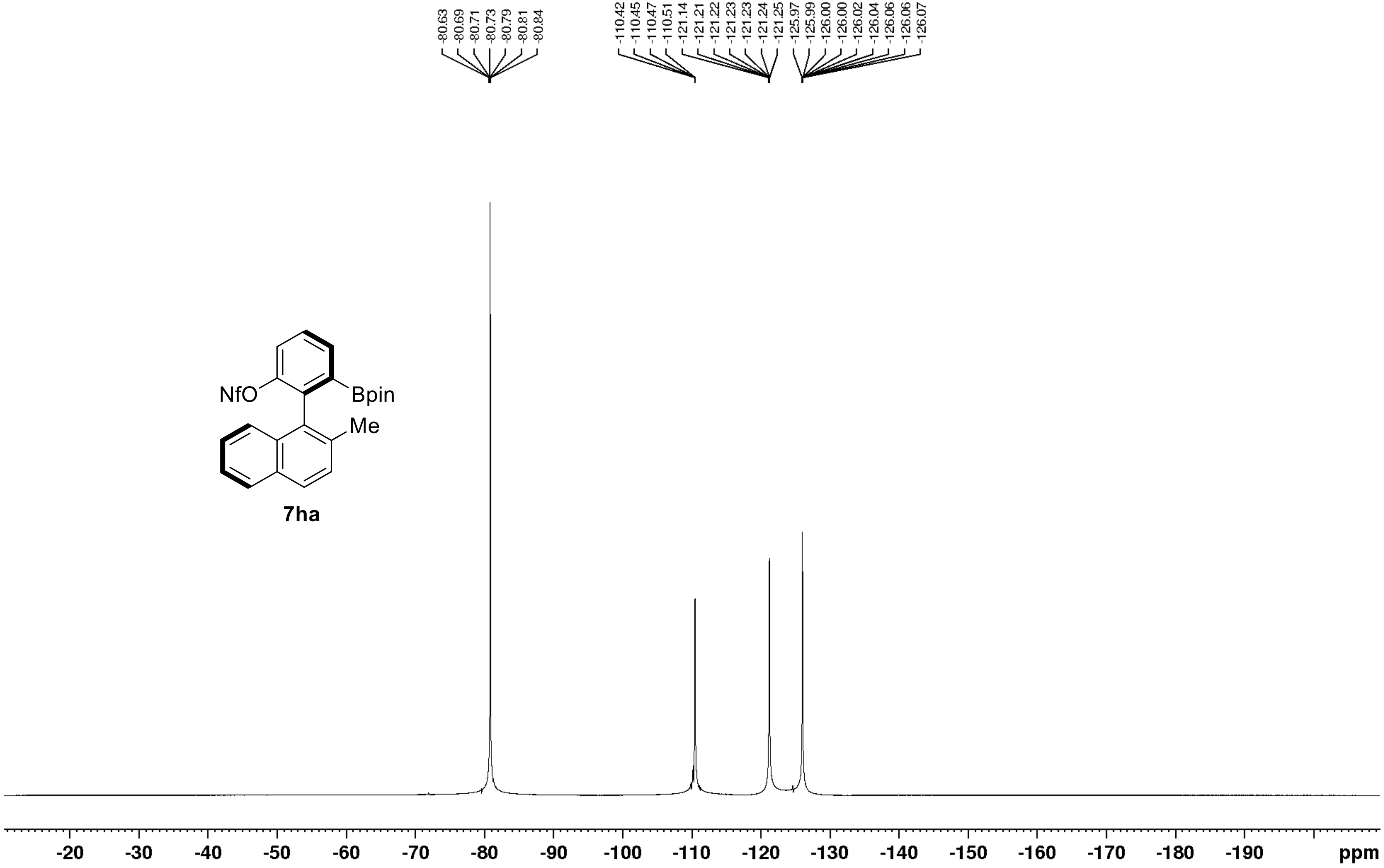

Figure S170. ^{11}**B NMR** (160 MHz, $CDCl_3$, 298 K) of **7ha**

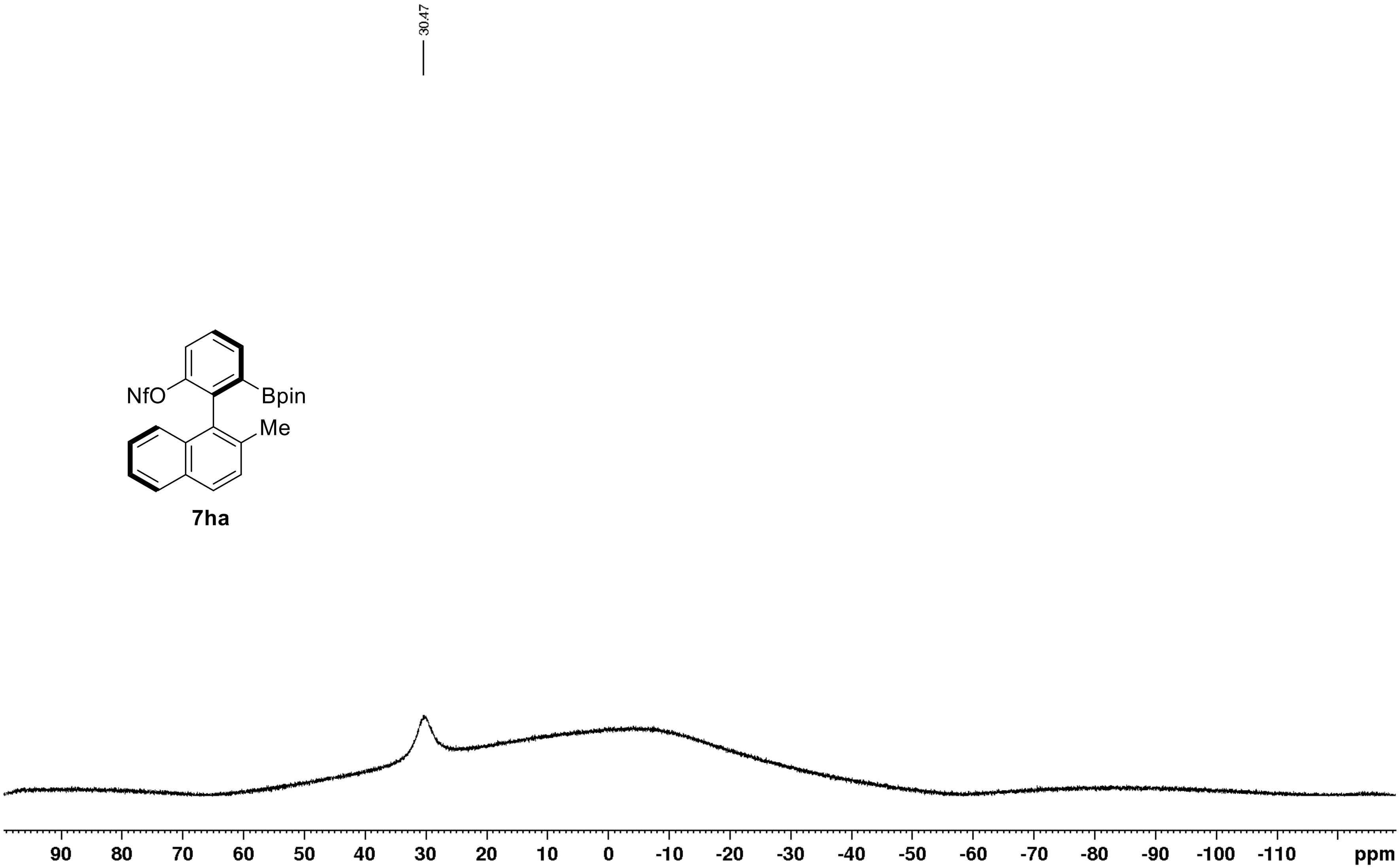

(*R*)-2-(8-methylnaphthalen-1-yl)-3-(4,4,5,5-tetramethyl-1,3,2-dioxaborolan-2-yl)phenyl 1,1,2,2,3,3,4,4,4-nonafluorobutane-1-sulfonate (7ia)

Figure S171. ^{1}**H NMR** (500 MHz, $CDCl_3$, 298 K) of **7ia**

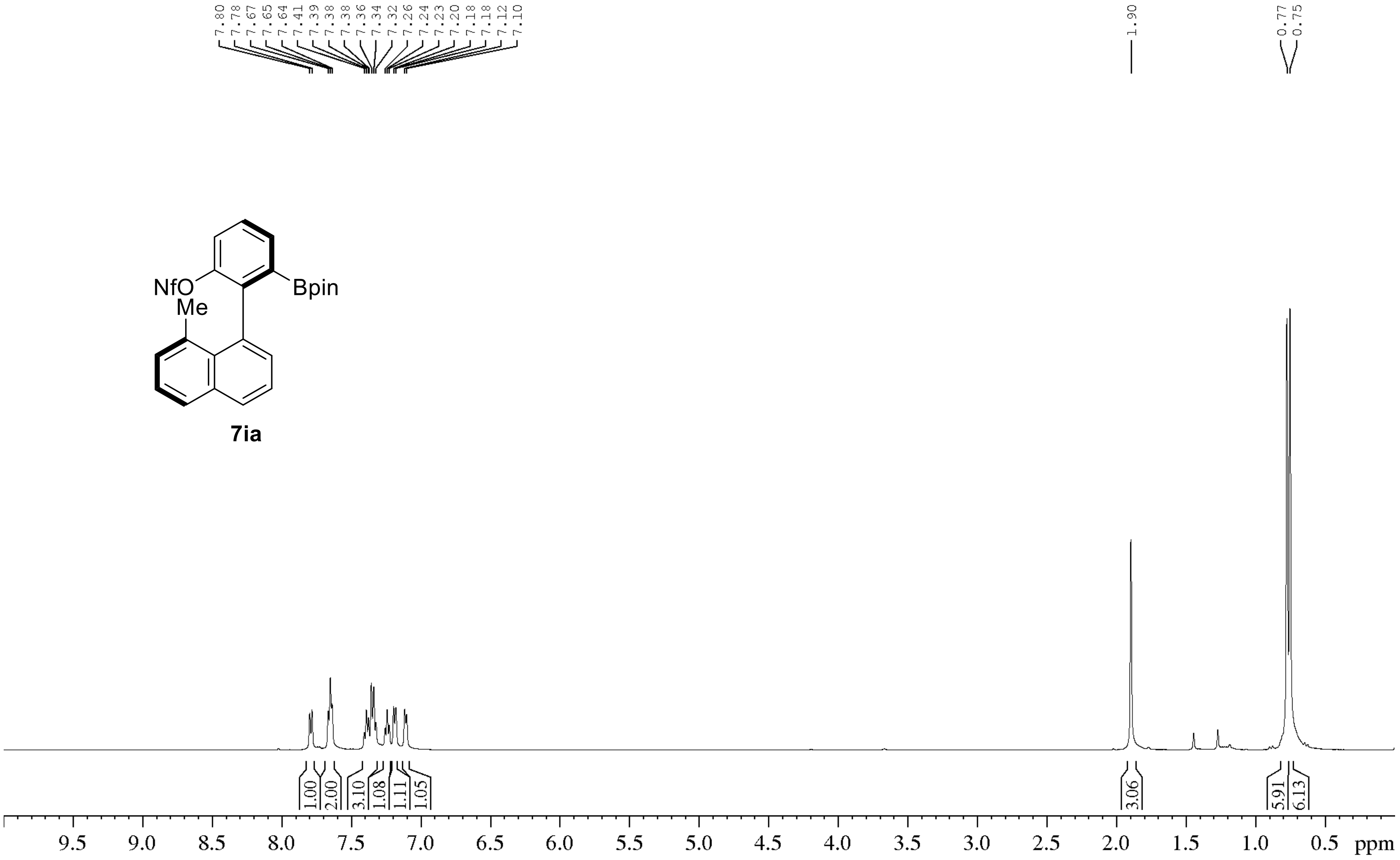

Figure S172. ^{13}**C{**^{1}**H} NMR** (126 MHz, $CDCl_3$, 298 K) of **7ia**

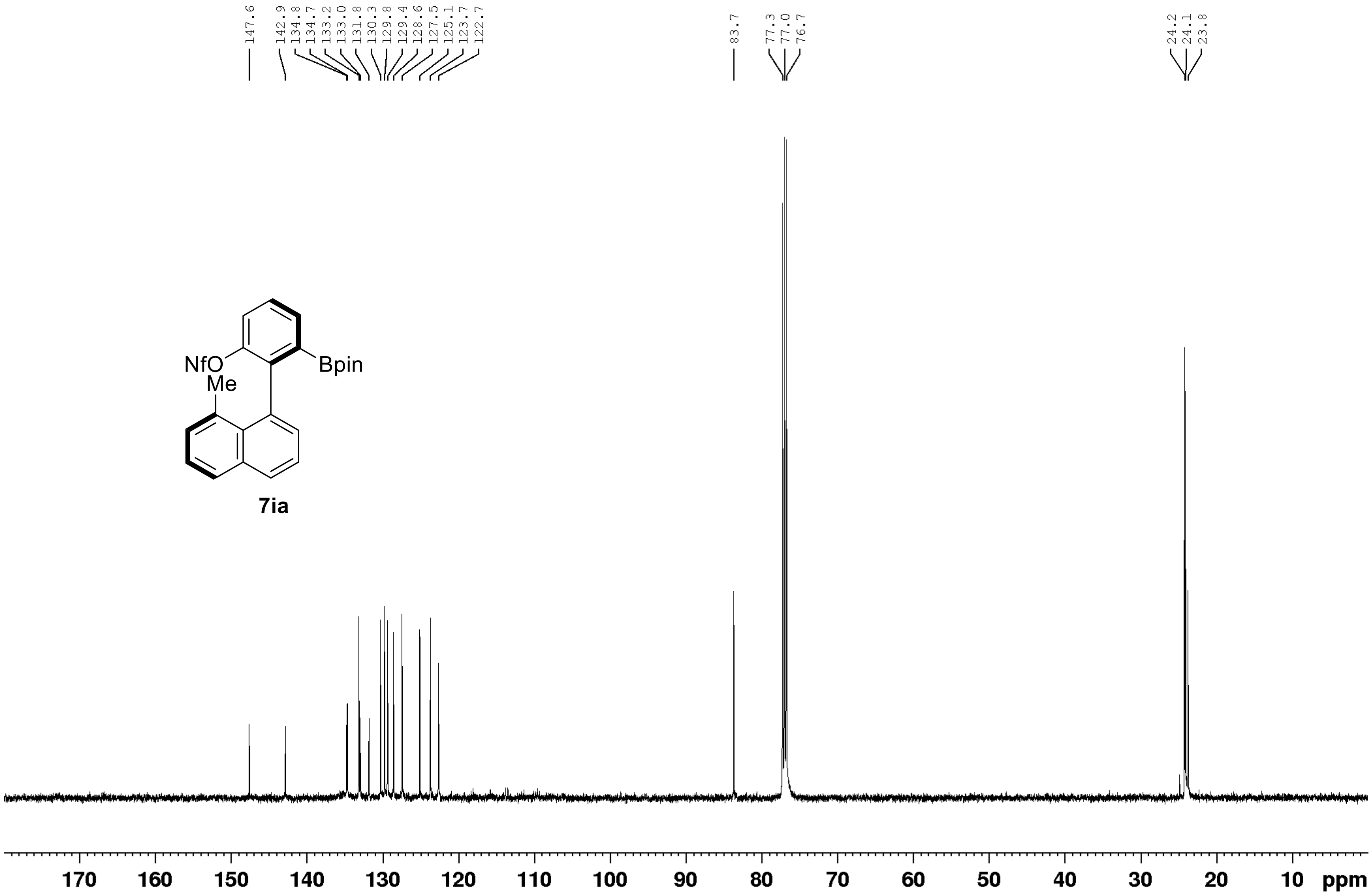

Figure S173. ^{19}**F NMR** (471 MHz, $CDCl_3$, 298 K) of **7ia**

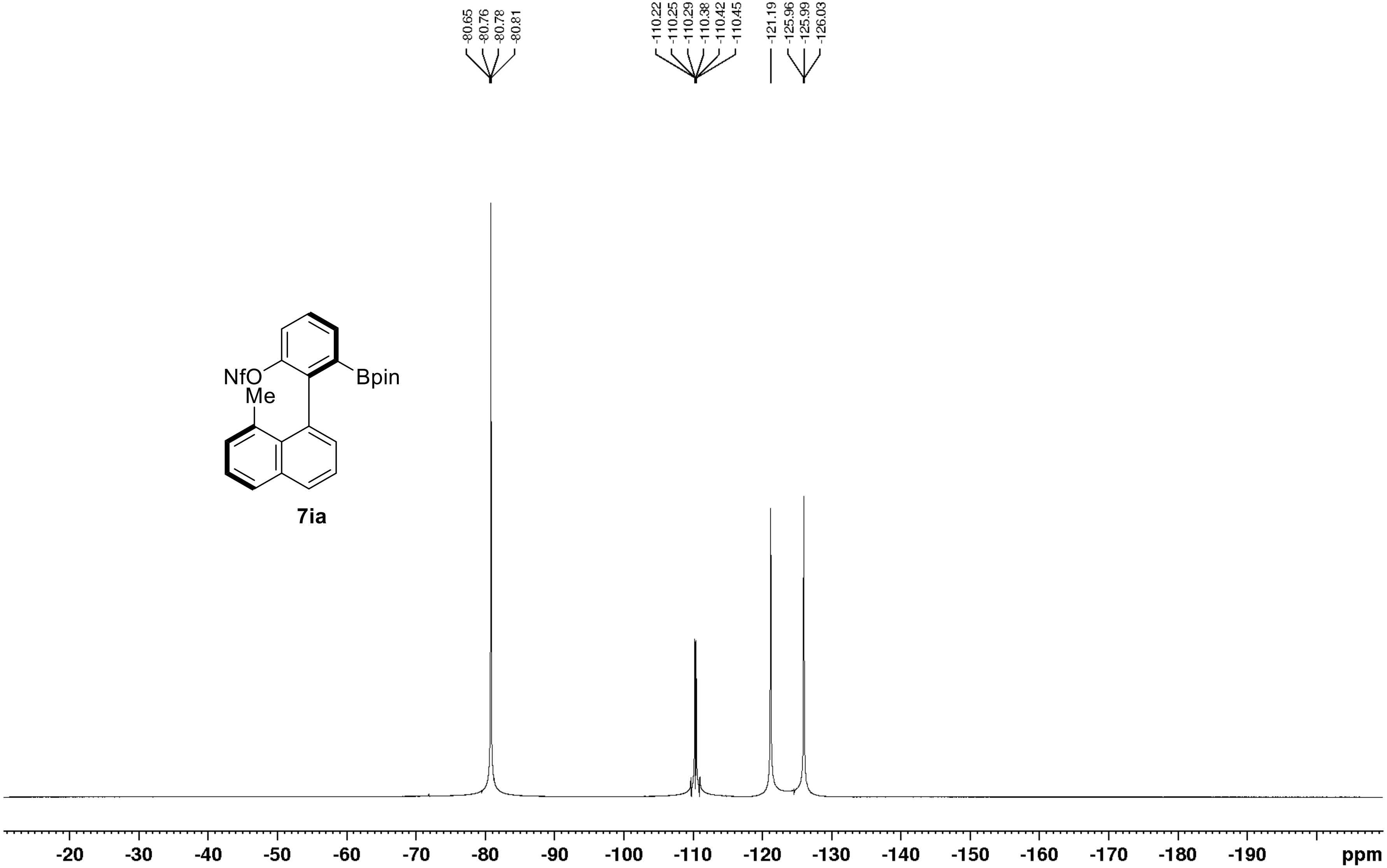

Figure S174. ^{11}B NMR (160 MHz, $CDCl_3$, 298 K) of **7ia**

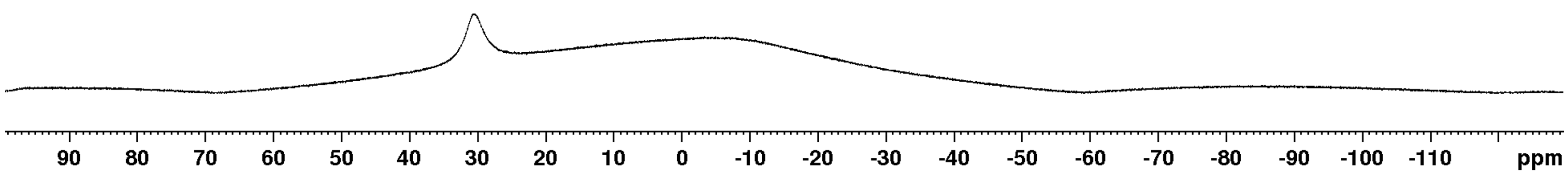

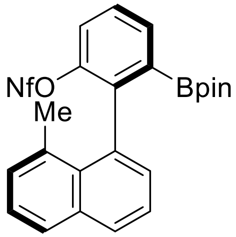

7ia

(*R*)-2-(1,2-dihydroacenaphthylen-5-yl)-3-(4,4,5,5-tetramethyl-1,3,2-dioxaborolan-2-yl)phenyl 1,1,2,2,3,3,4,4,4-nonafluorobutane-1-sulfonate (7ja)

Figure S175. **¹H NMR** (500 MHz, $CDCl_3$, 298 K) of **7ja**

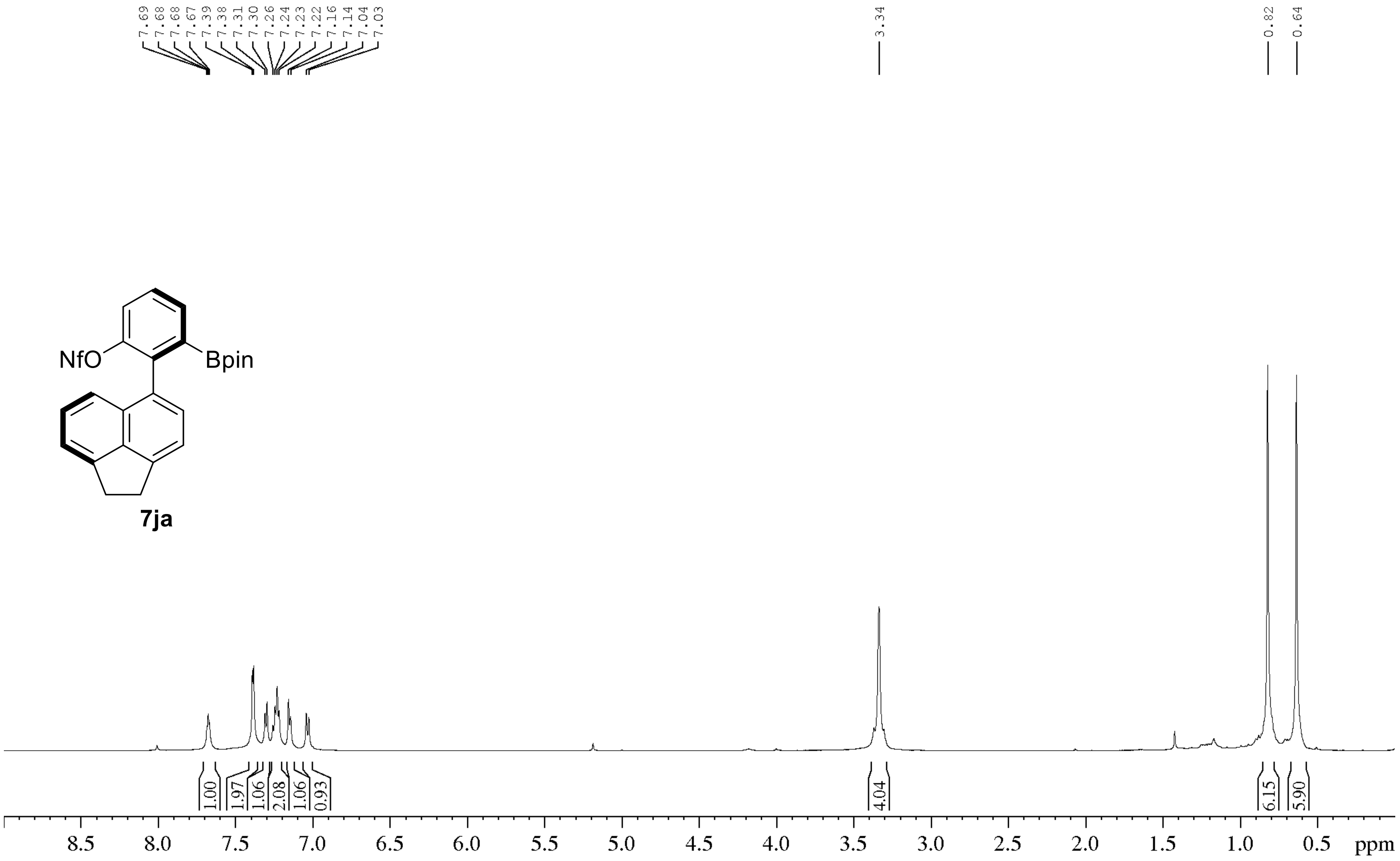

Figure S176. ^{13}C{^{1}H} NMR (126 MHz, $CDCl_3$, 298 K) of **7ja**

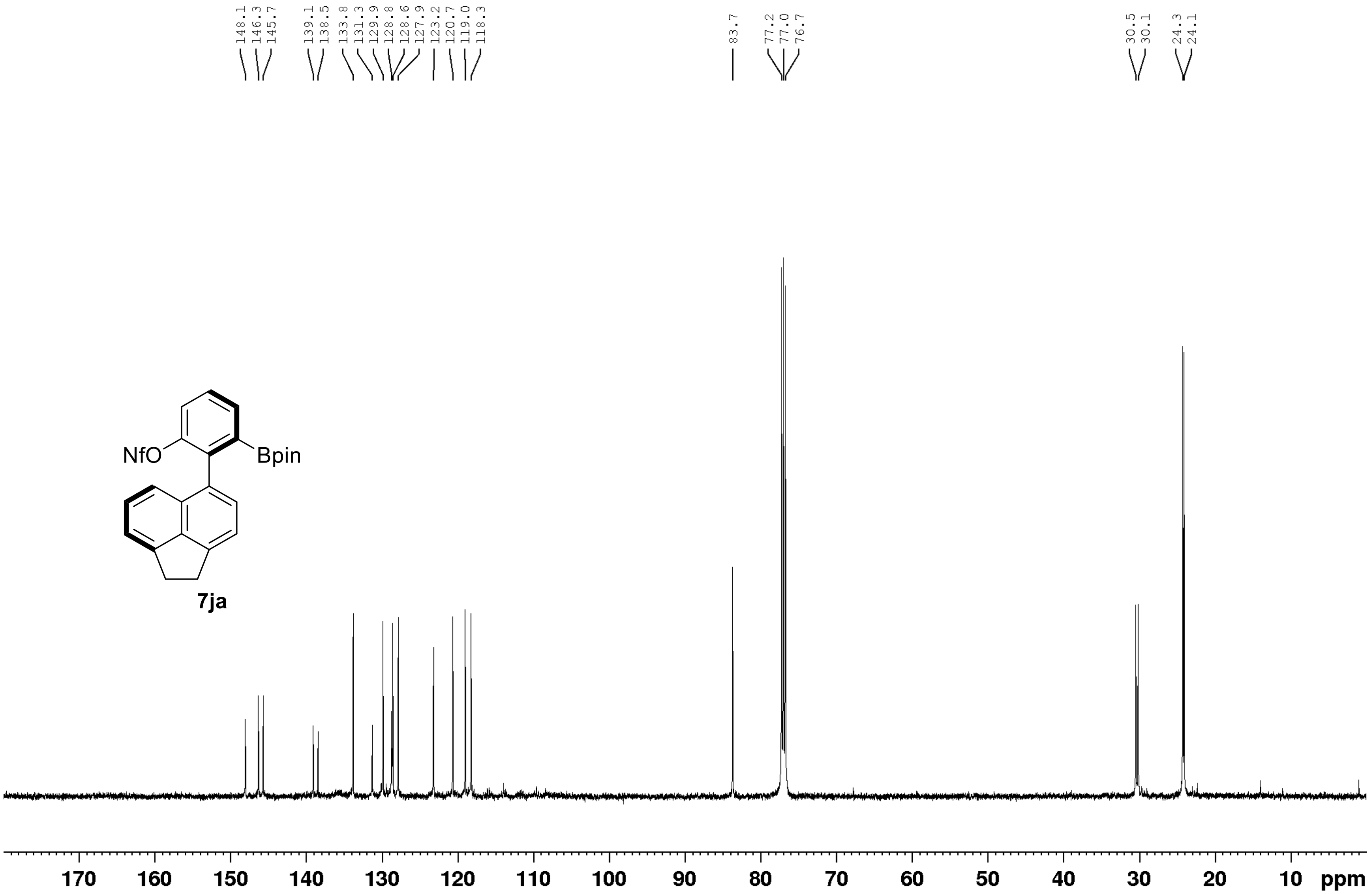

Figure S177. ^{19}F NMR (471 MHz, $CDCl_3$, 298 K) of **7ja**

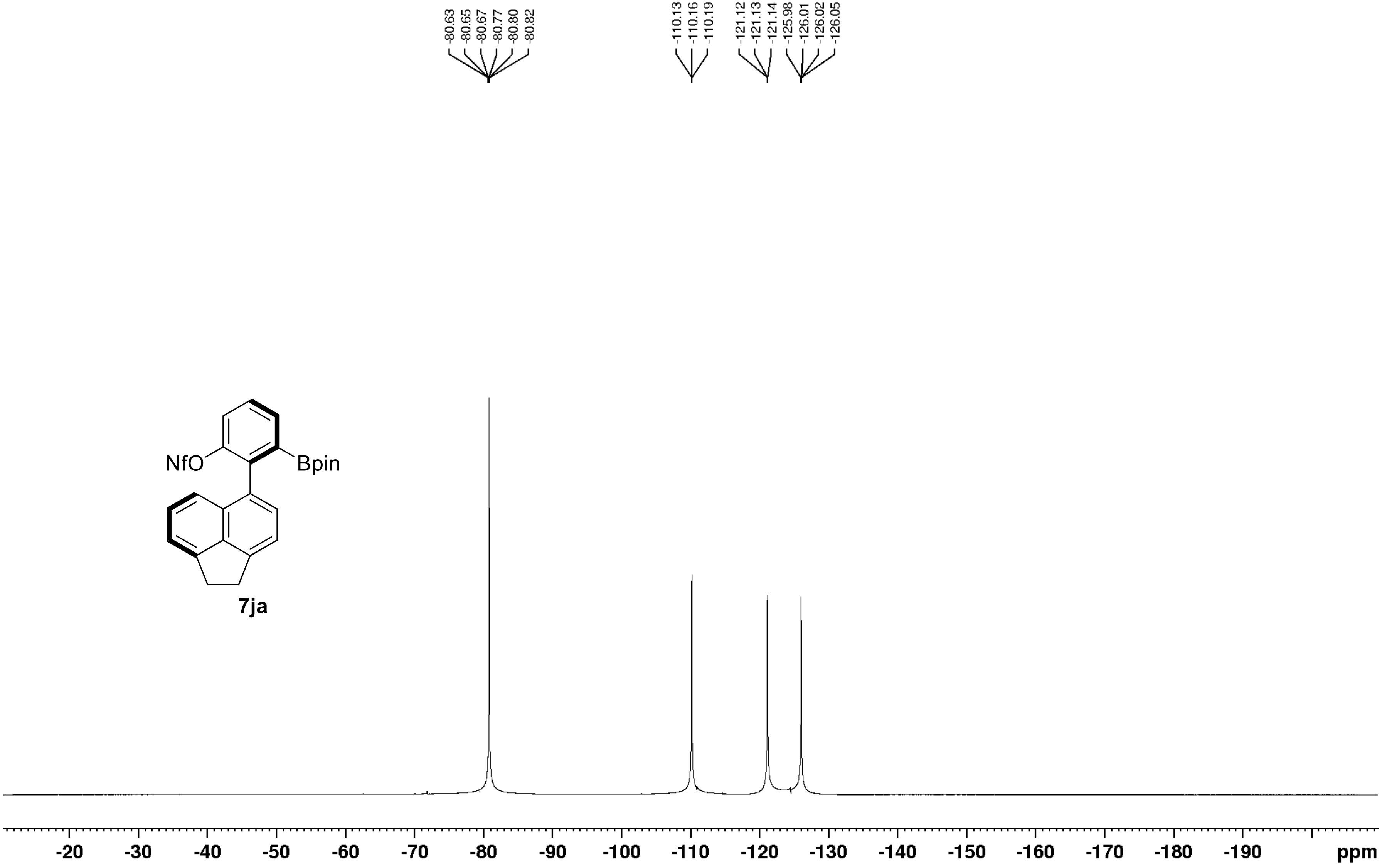

Figure S178. ^{11}**B NMR** (160 MHz, $CDCl_3$, 298 K) of **7ja**

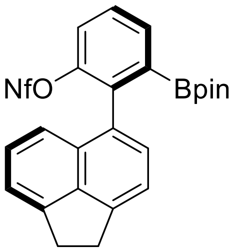

7ja

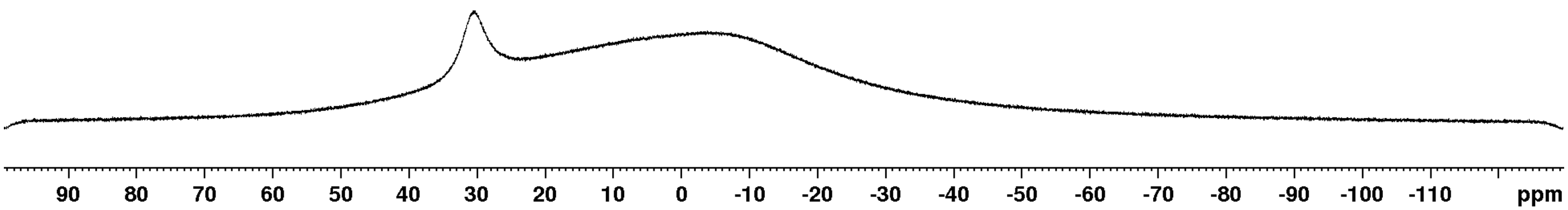

(*R*)-2-(fluoranthen-3-yl)-3-(4,4,5,5-tetramethyl-1,3,2-dioxaborolan-2-yl)phenyl 1,1,2,2,3,3,4,4,4-nonafluorobutane-1-sulfonate (7ka)

Figure S179. **^{1}H NMR** (500 MHz, $CDCl_3$, 298 K) of **7ka**

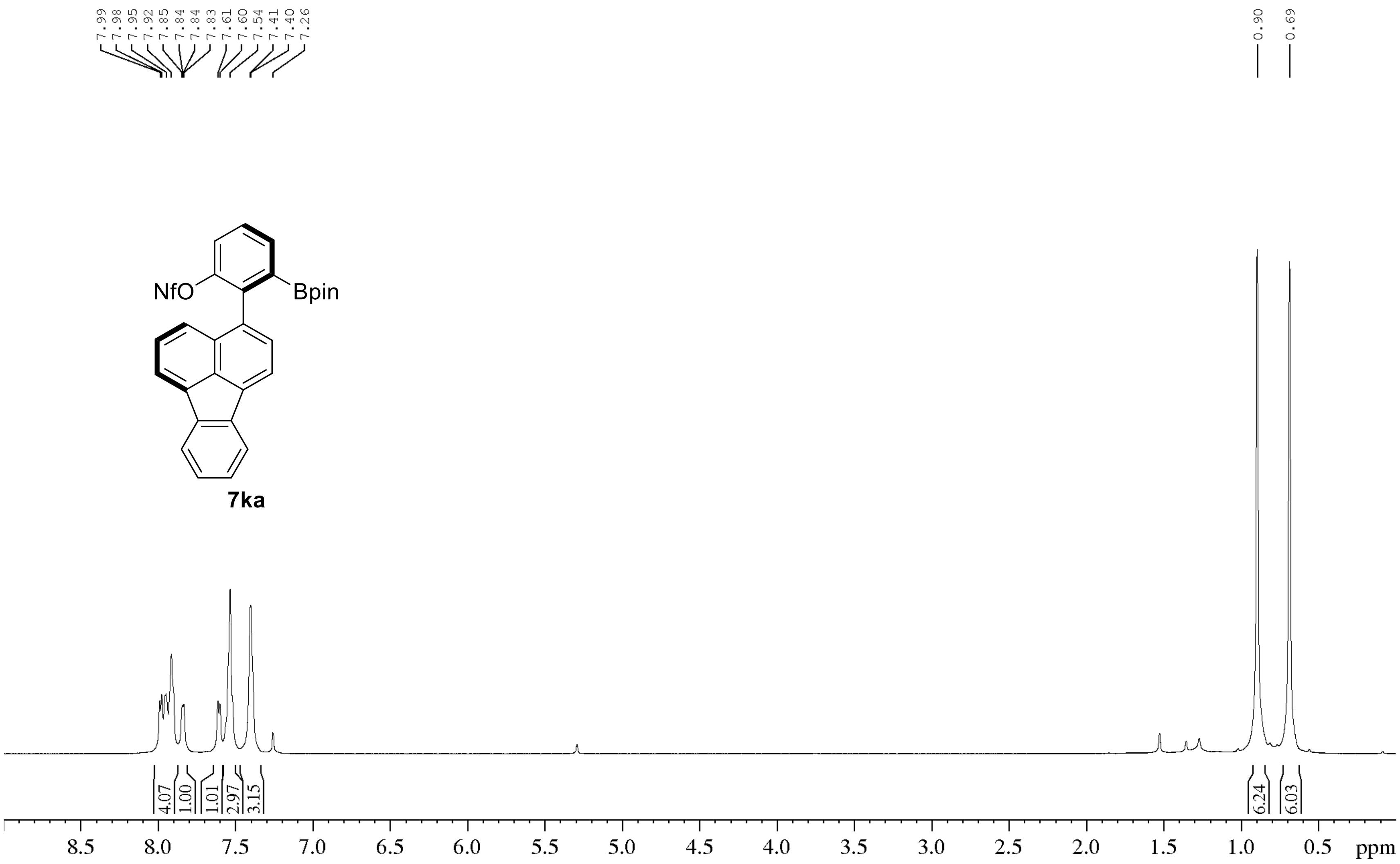

Figure S180. ^{13}**C{**^{1}**H} NMR** (126 MHz, $CDCl_3$, 298 K) of **7ka**

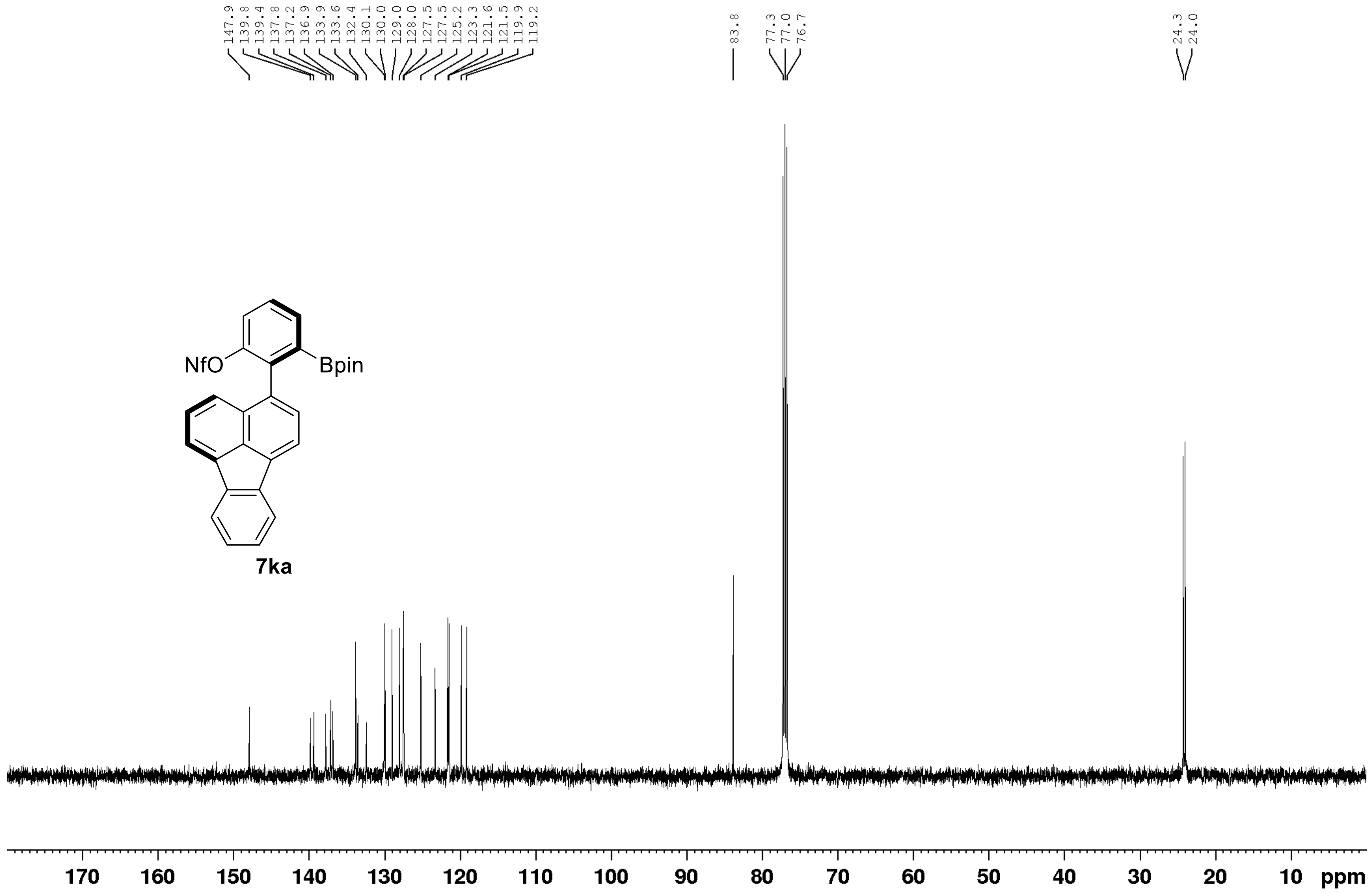

Figure S181. ^{19}**F NMR** (471 MHz, $CDCl_3$, 298 K) of **7ka**

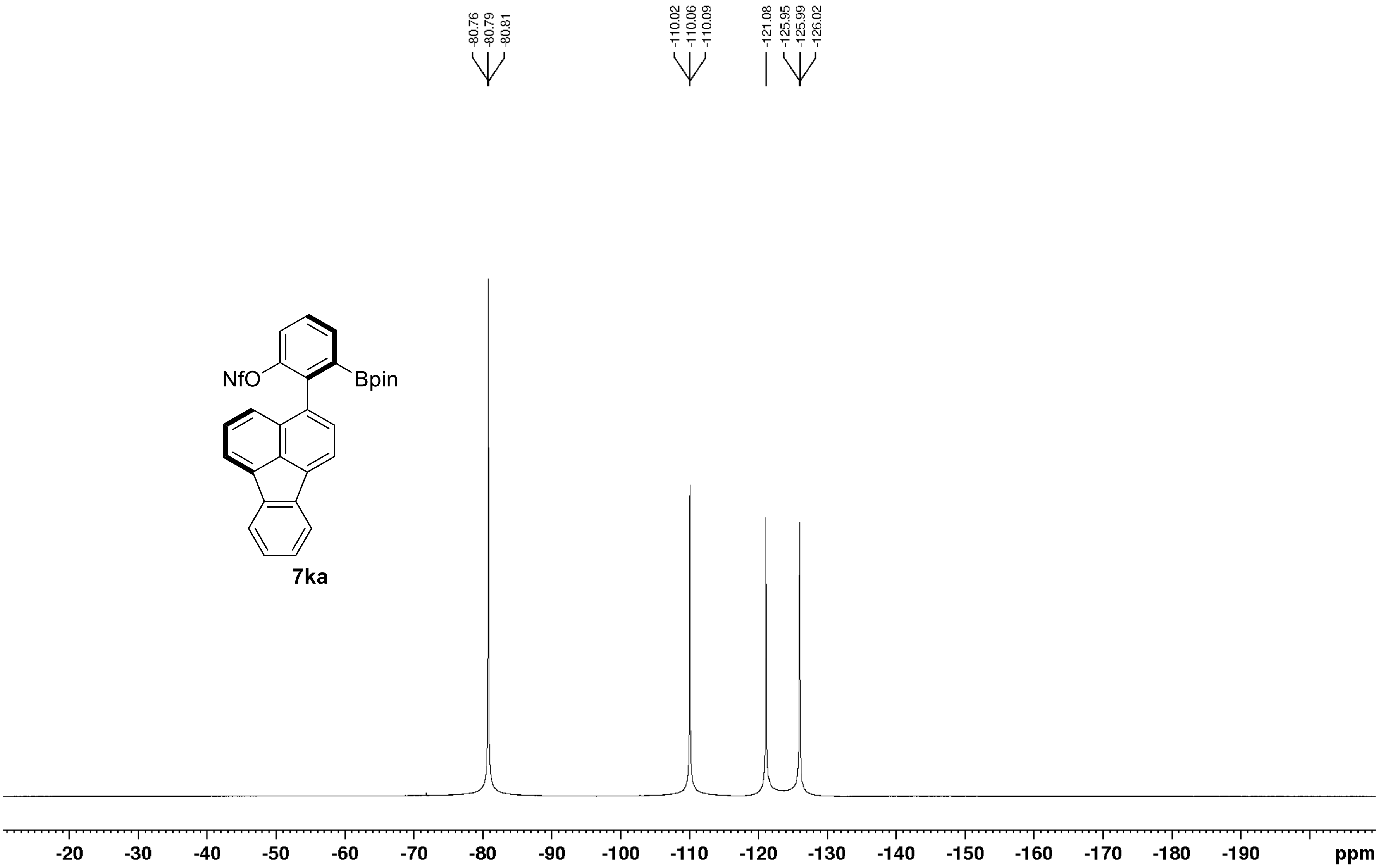

Figure S182. ^{11}**B NMR** (160 MHz, $CDCl_3$, 298 K) of **7ka**

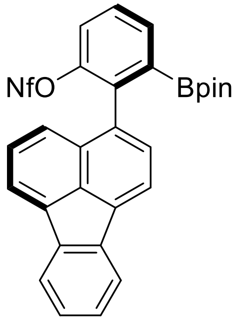

7ka

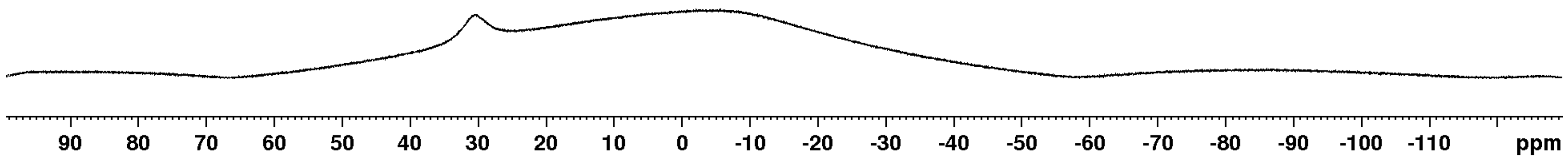

(*R*)-6-(4,4,5,5-tetramethyl-1,3,2-dioxaborolan-2-yl)-[1,1':2',1''-terphenyl]-2-yl 1,1,2,2,3,3,4,4,4-nonafluorobutane-1-sulfonate (**7la**)

Figure S183. **^{1}H NMR** (400 MHz, $CDCl_3$, 298 K) of **7la**

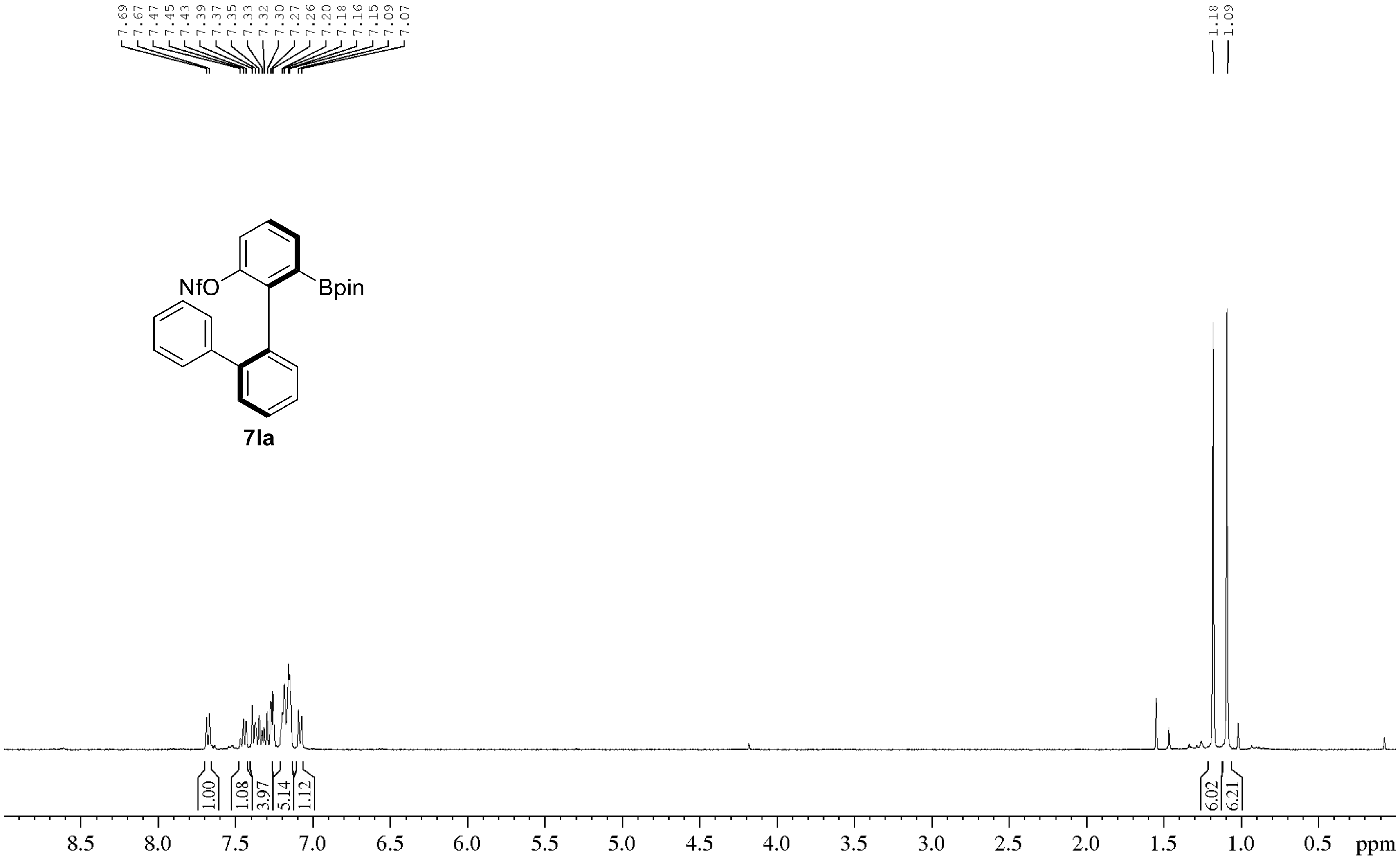

Figure S184. ^{13}**C{**^{1}**H} NMR** (101 MHz, $CDCl_3$, 298 K) of **7la**

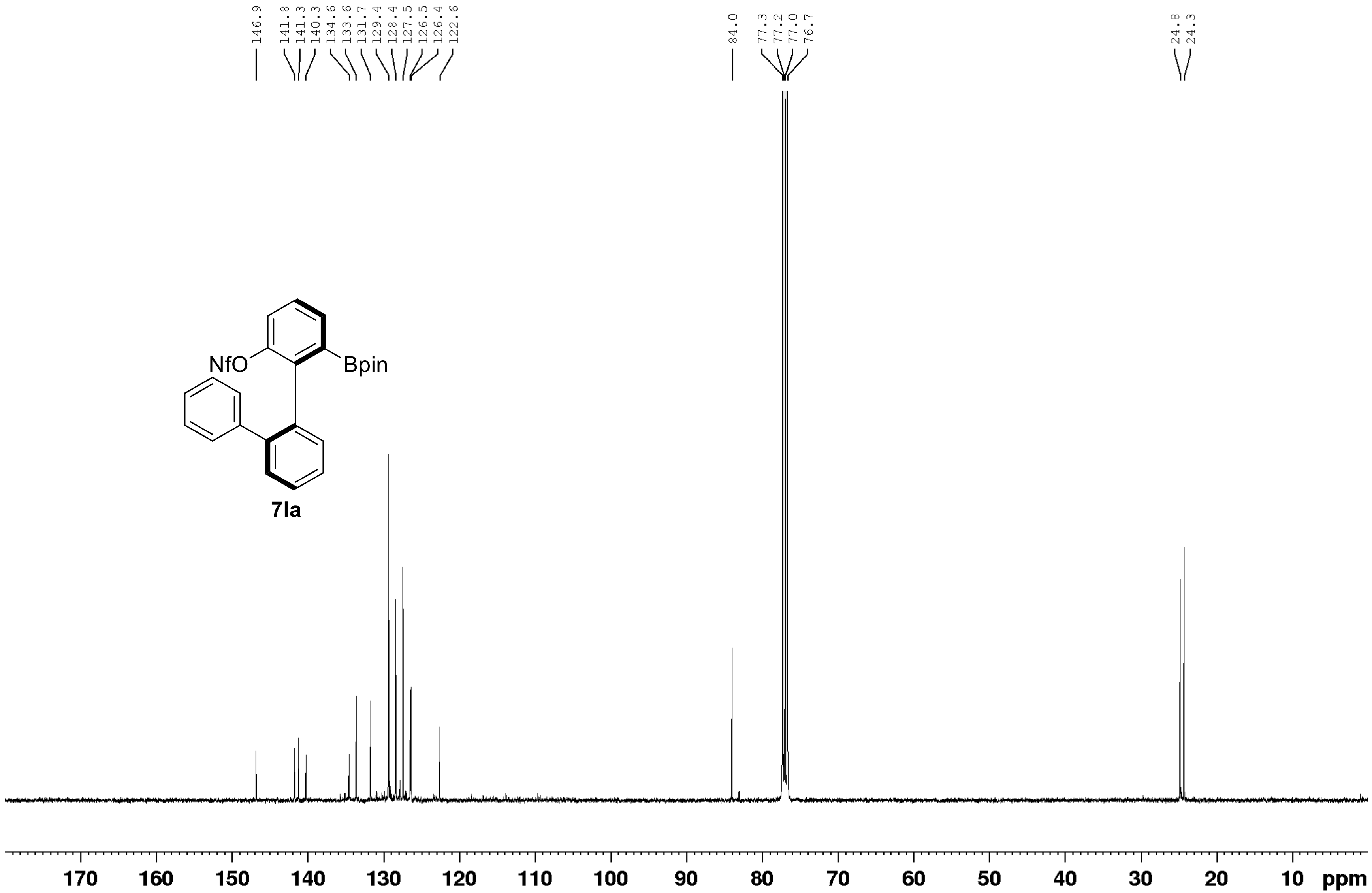

Figure S185. **^{19}F NMR** (471 MHz, $CDCl_3$, 298 K) of **7la**

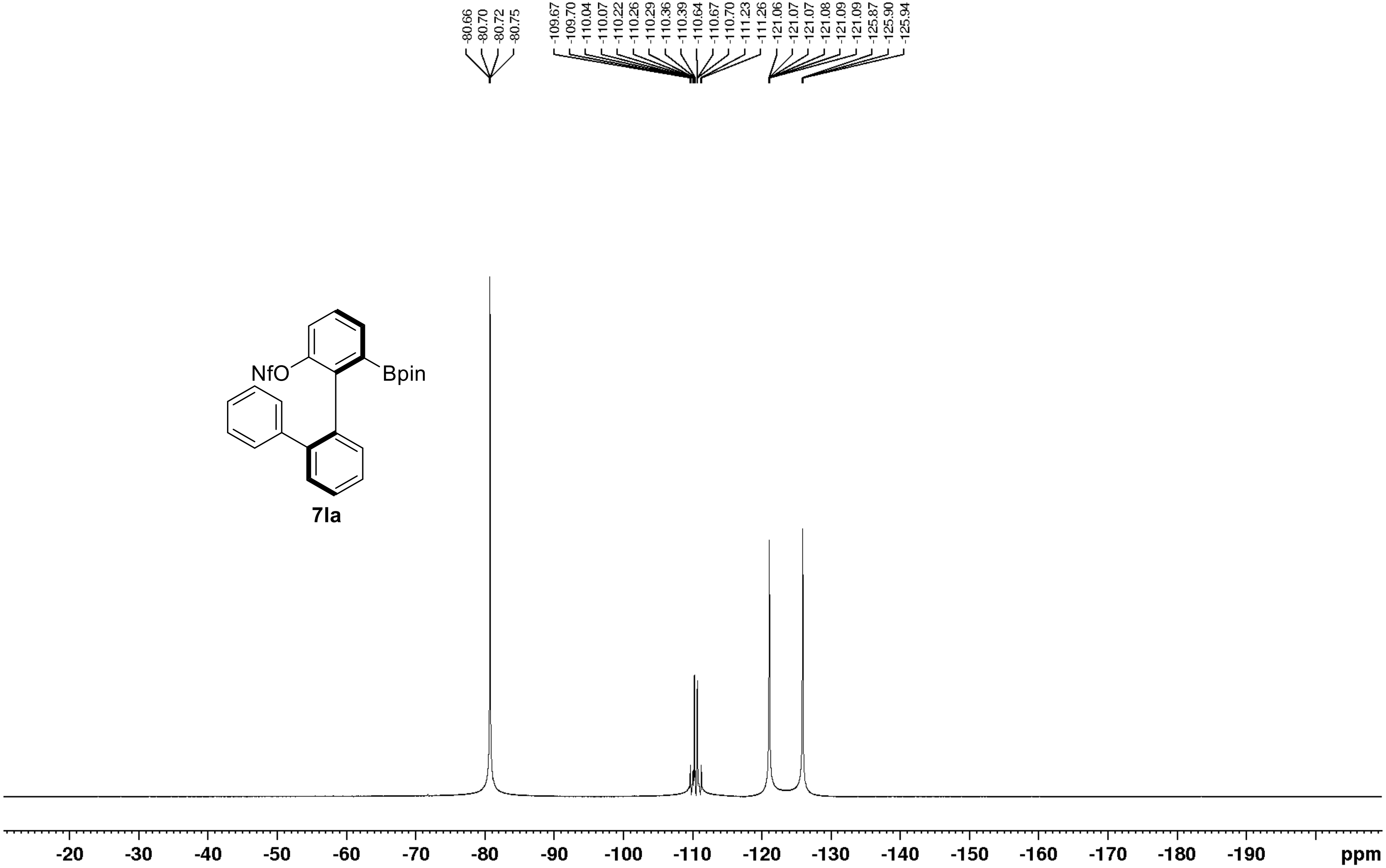

Figure S186. ^{11}B NMR (160 MHz, $CDCl_3$, 298 K) of **7la**

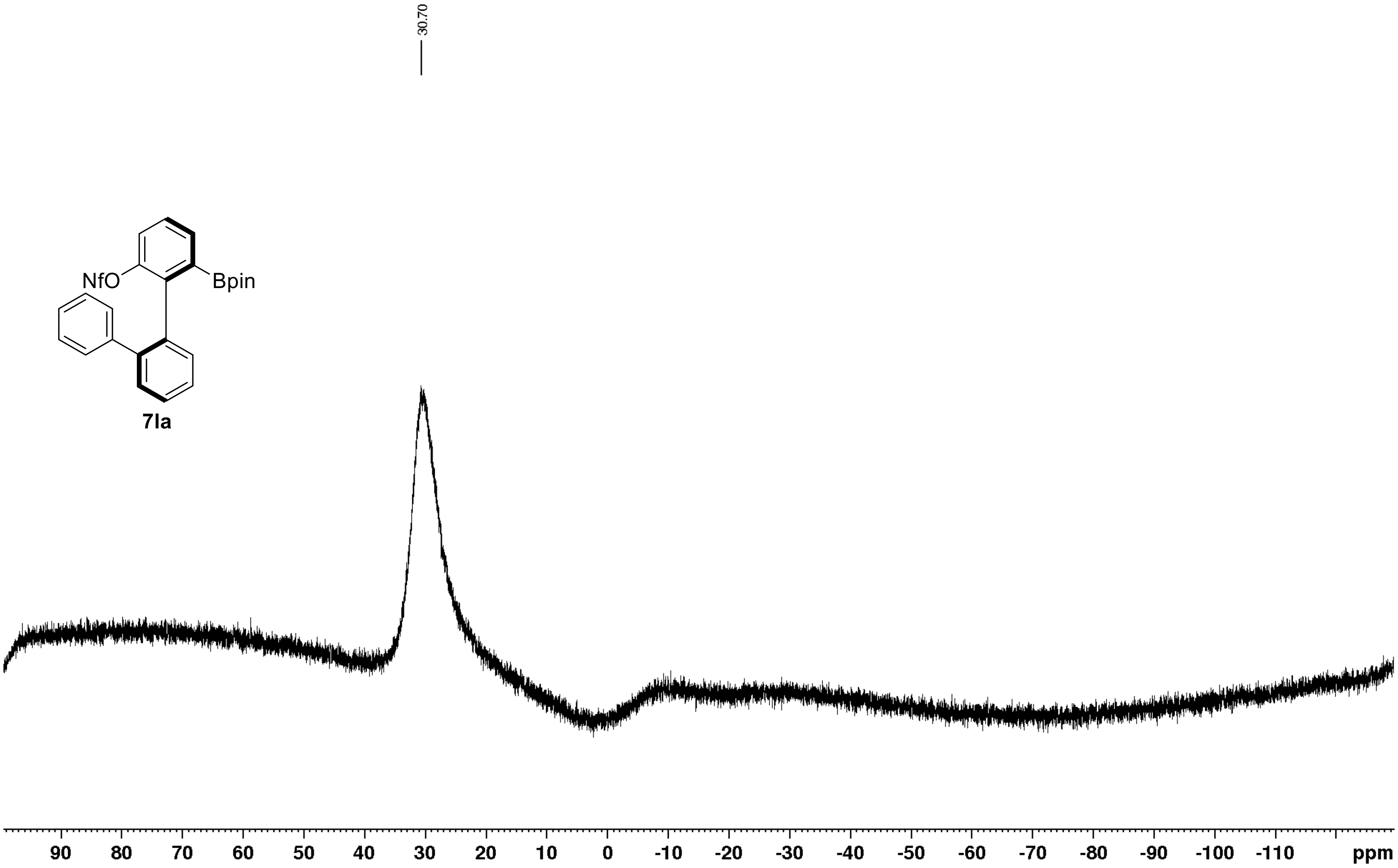

(*R*)-2''-fluoro-6-(4,4,5,5-tetramethyl-1,3,2-dioxaborolan-2-yl)-[1,1':2',1''-terphenyl]-2-yl 1,1,2,2,3,3,4,4,4-nonafluorobutane-1-sulfonate (**7ma**)

Figure S187. ^{1}H NMR (500 MHz, $CDCl_3$, 298 K) of **7ma**

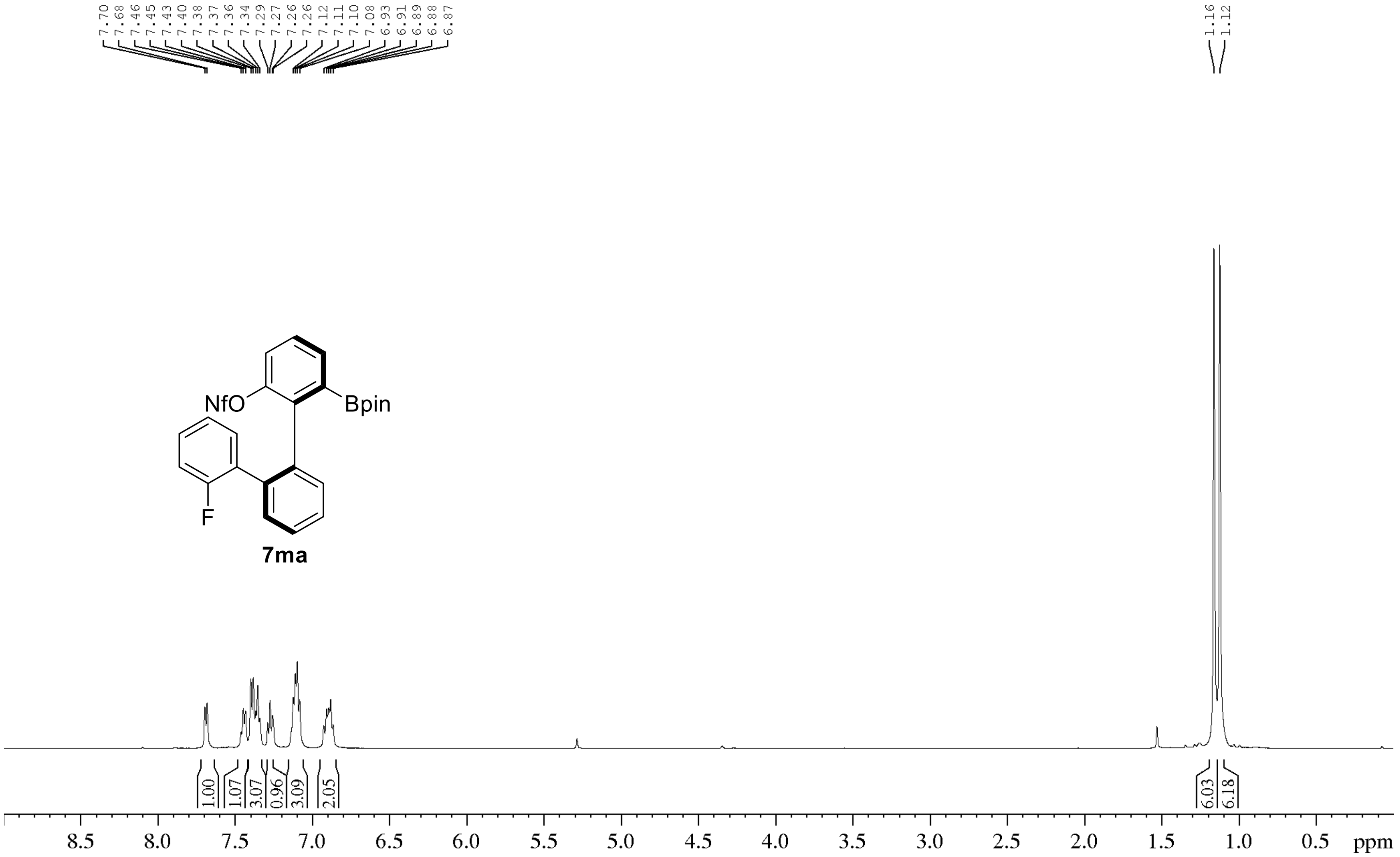

Figure S188. $^{13}C\{^1H\}$ **NMR** (126 MHz, $CDCl_3$, 298 K) of **7ma**

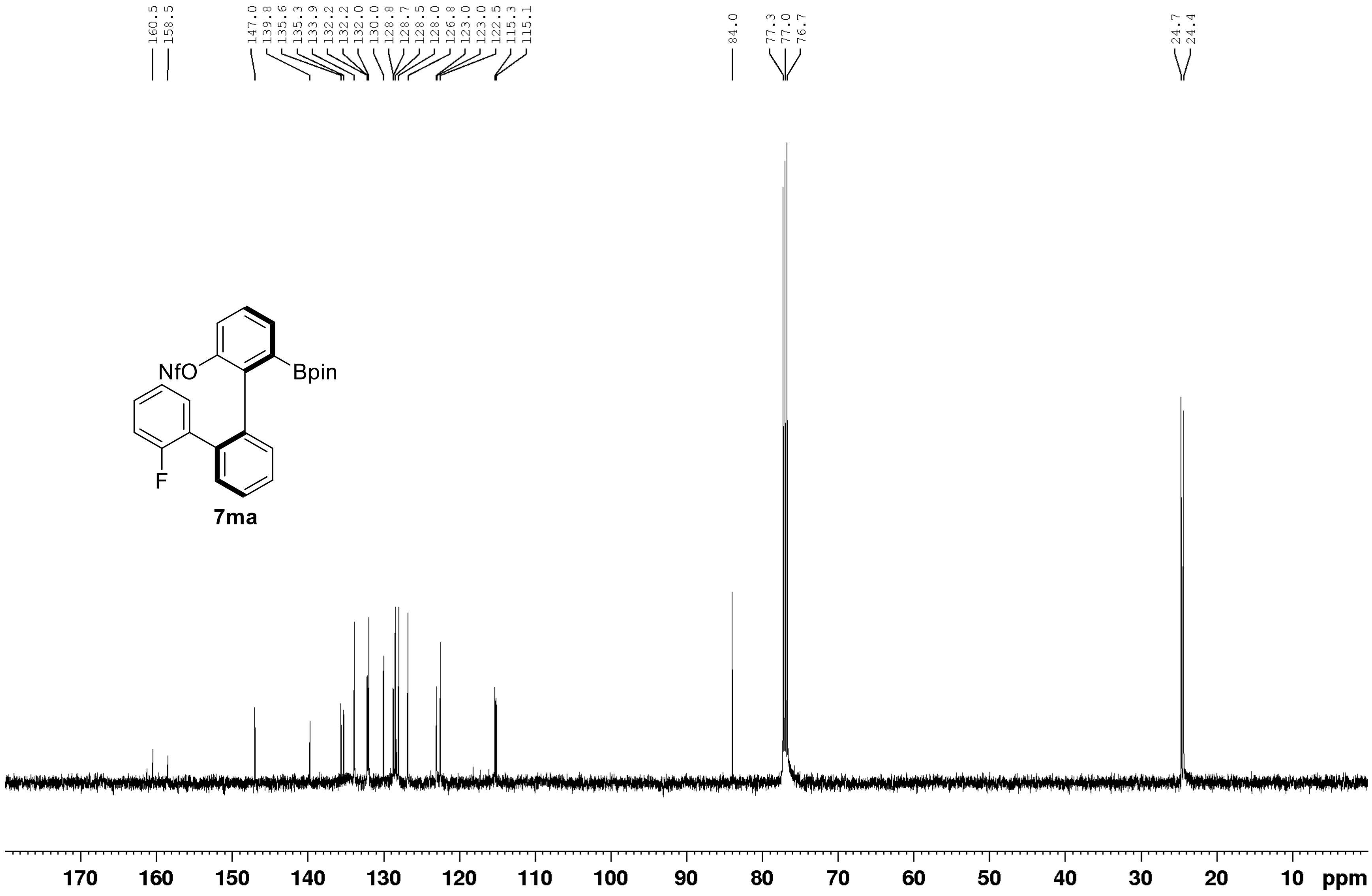

Figure S189. ^{19}**F NMR** (471 MHz, $CDCl_3$, 298 K) of **7ma**

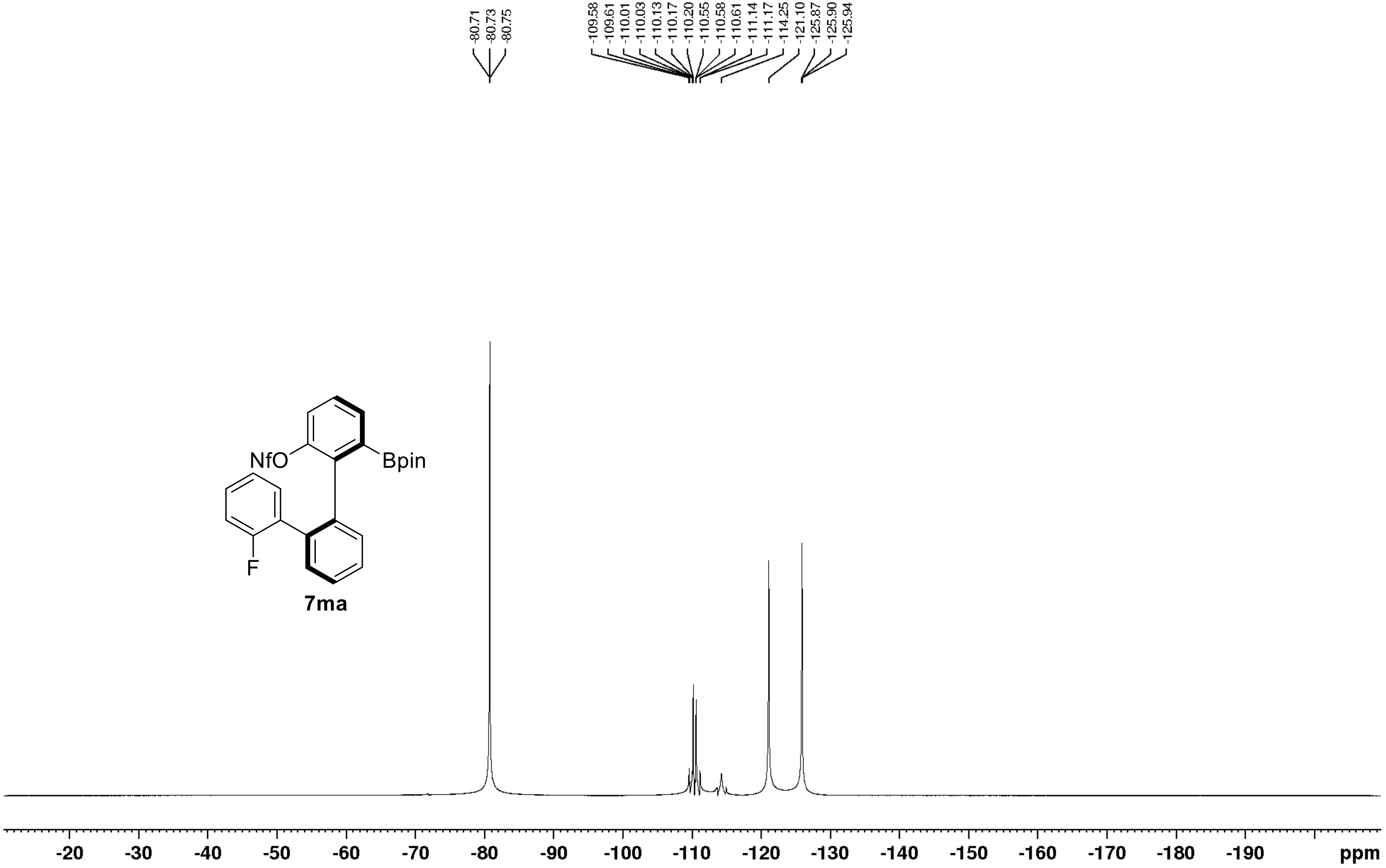

Figure S190. ^{11}**B NMR** (160 MHz, $CDCl_3$, 298 K) of **7ma**

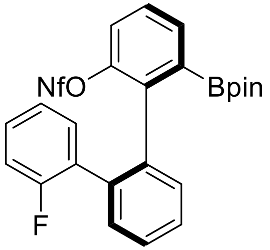

7ma

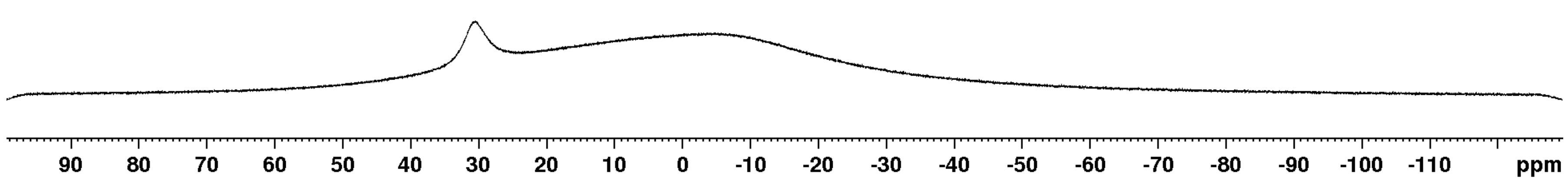

(*R*)-3''-chloro-6-(4,4,5,5-tetramethyl-1,3,2-dioxaborolan-2-yl)-[1,1':2',1''-terphenyl]-2-yl 1,1,2,2,3,3,4,4,4-nonafluorobutane-1-sulfonate (**7na**)

Figure S191. ^{1}**H NMR** (500 MHz, $CDCl_3$, 298 K) of **7na**

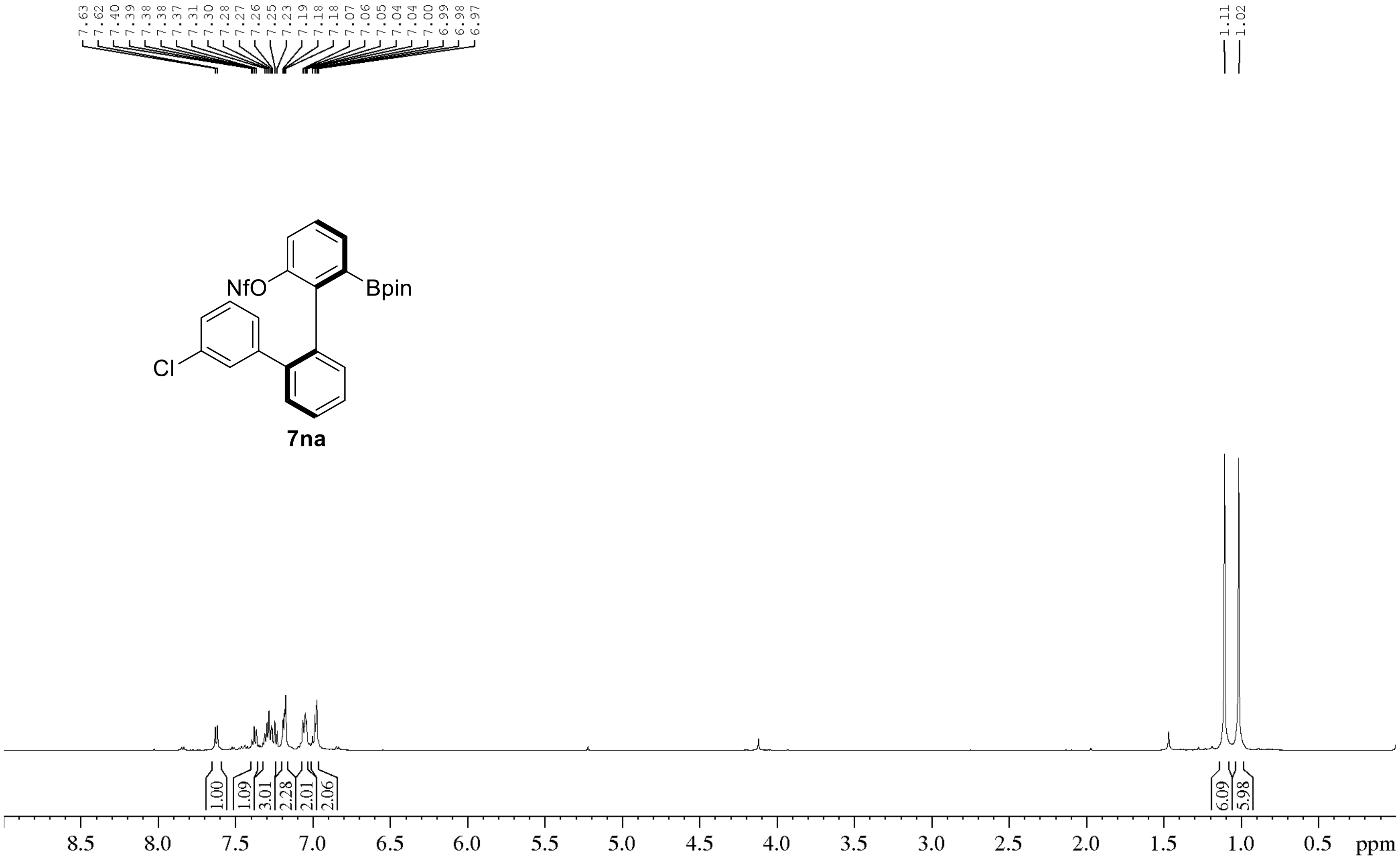

Figure S192. ^{13}C{^{1}H} NMR (101 MHz, $CDCl_3$, 298 K) of **7na**

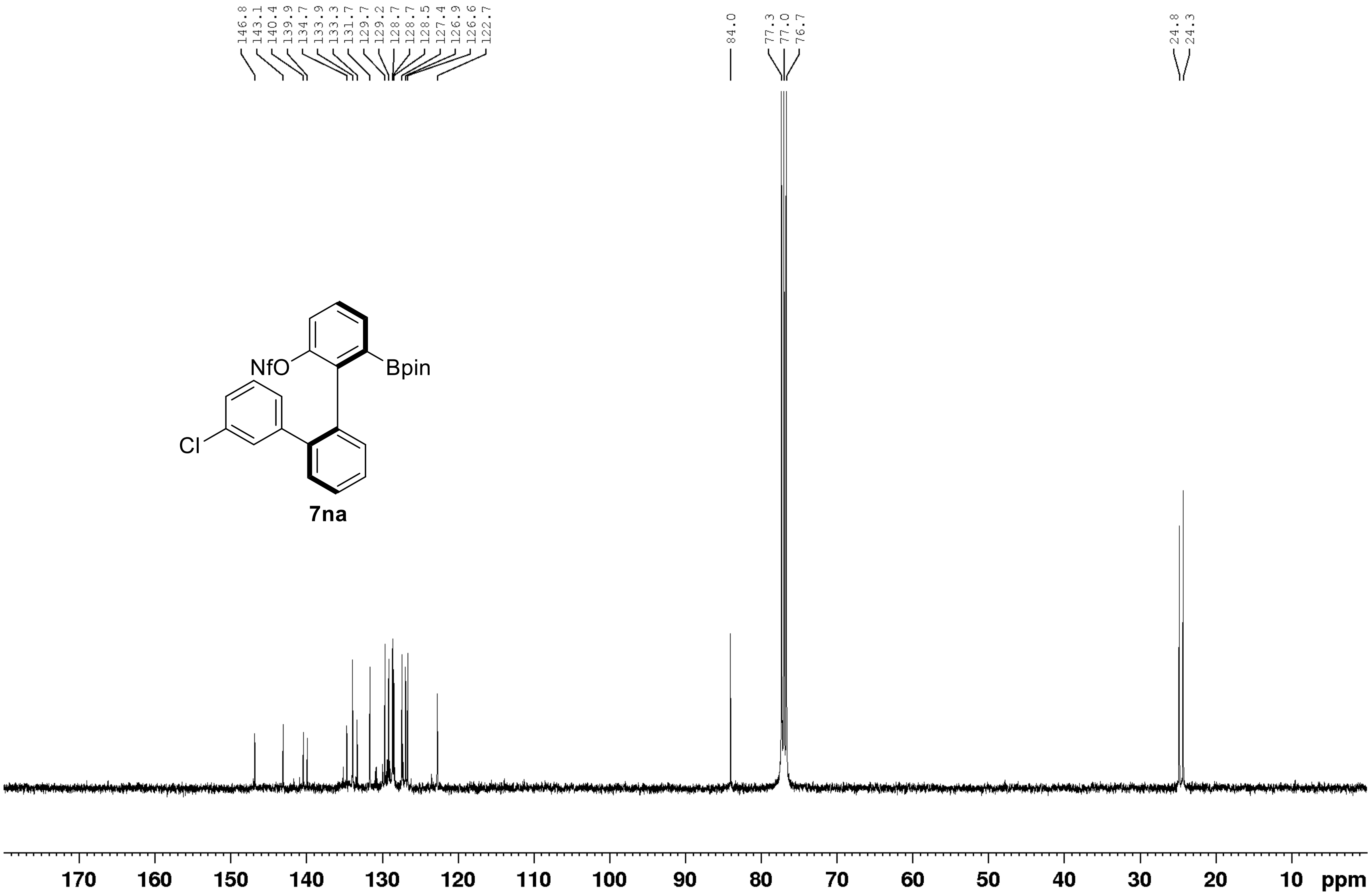

Figure S193. ^{19}F NMR (471 MHz, $CDCl_3$, 298 K) of **7na**

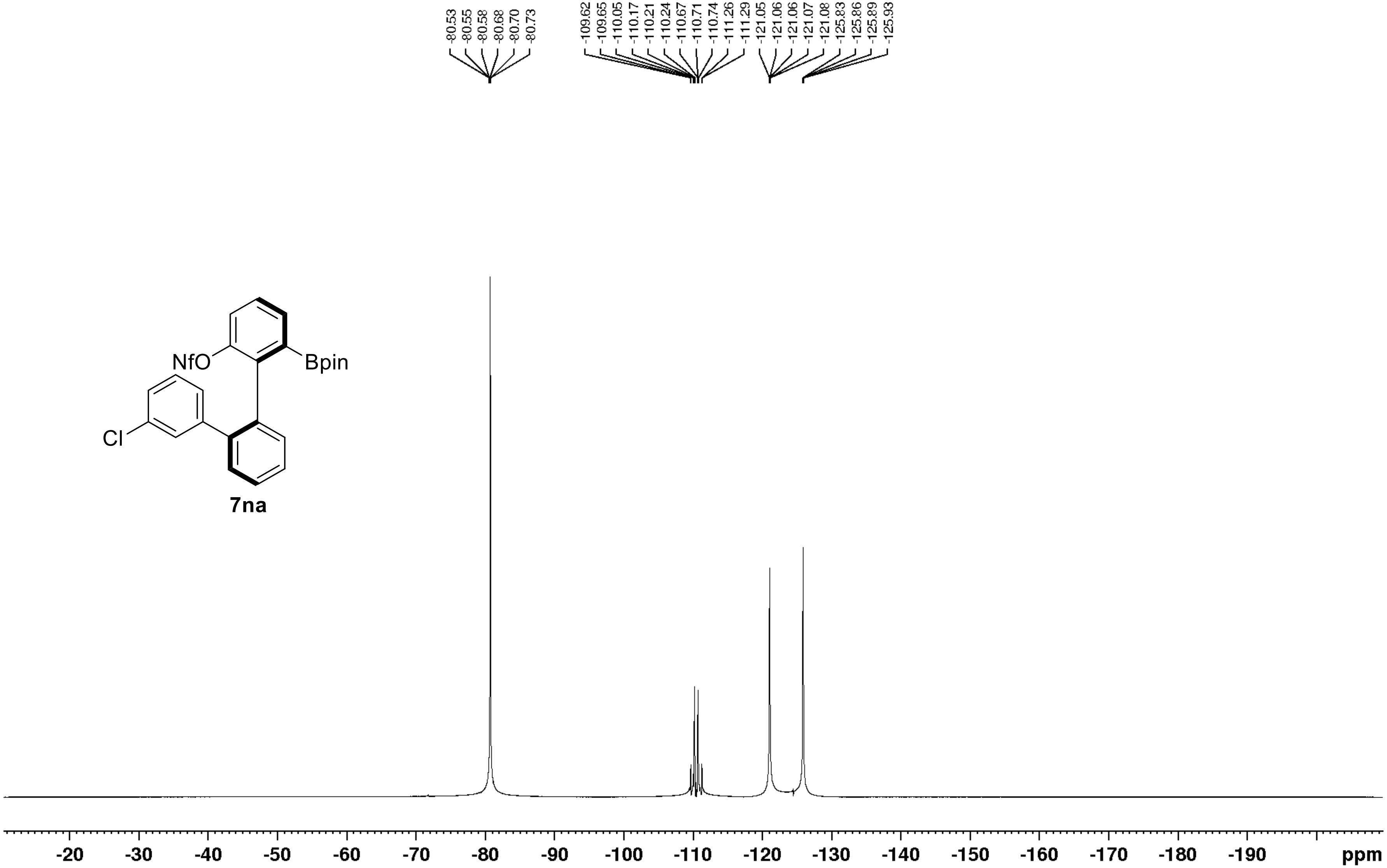

7na

Figure S194. ^{11}**B NMR** (160 MHz, $CDCl_3$, 298 K) of **7na**

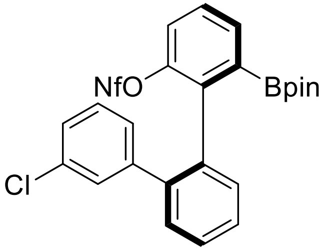

7na

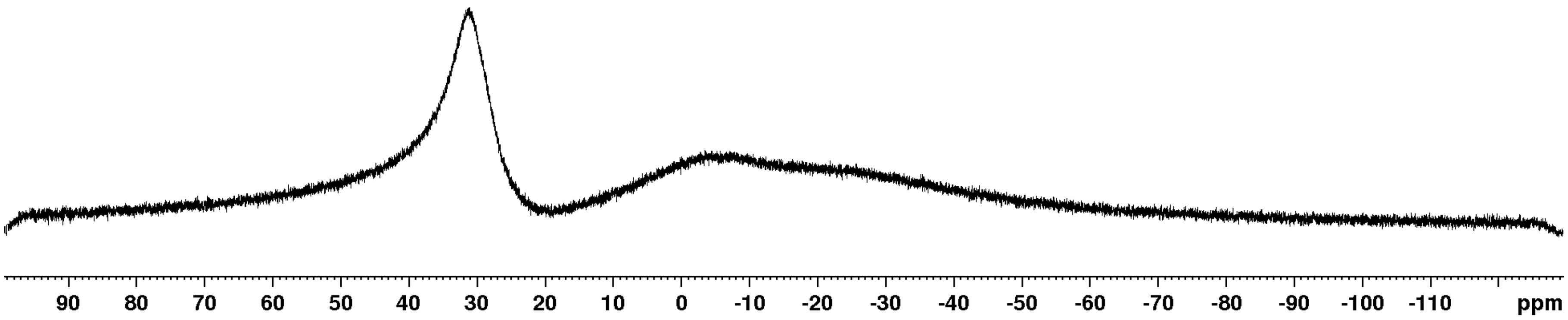

(*R*)-4''-(*tert*-butyl)-6-(4,4,5,5-tetramethyl-1,3,2-dioxaborolan-2-yl)-[1,1':2',1''-terphenyl]-2-yl 1,1,2,2,3,3,4,4,4-nonafluorobutane-1-sulfonate (**7oa**)

Figure S195. **^{1}H NMR** (500 MHz, $CDCl_3$, 298 K) of **7oa**

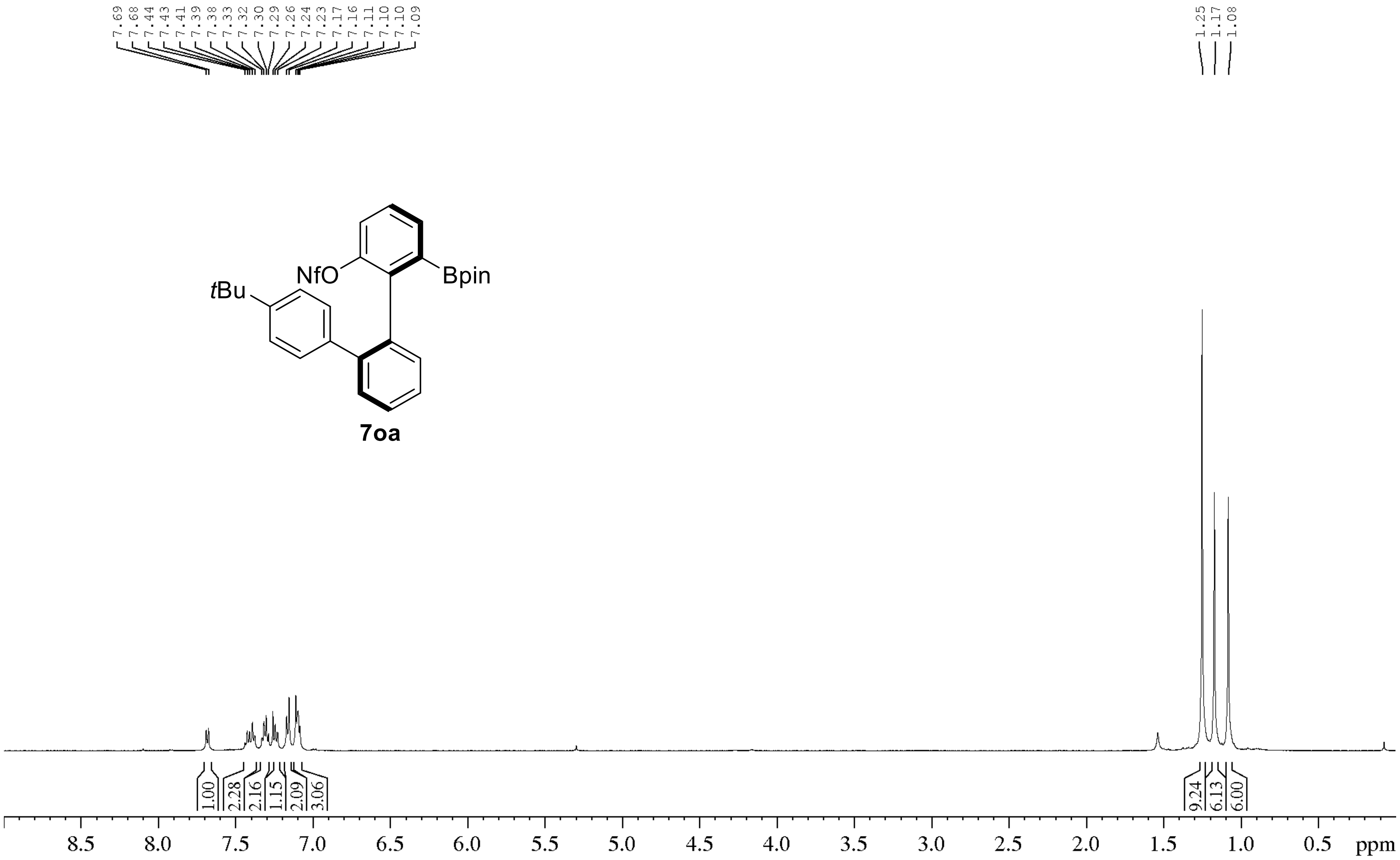

Figure S196. $^{13}C\{^1H\}$ **NMR** (126 MHz, $CDCl_3$, 298 K) of **7oa**

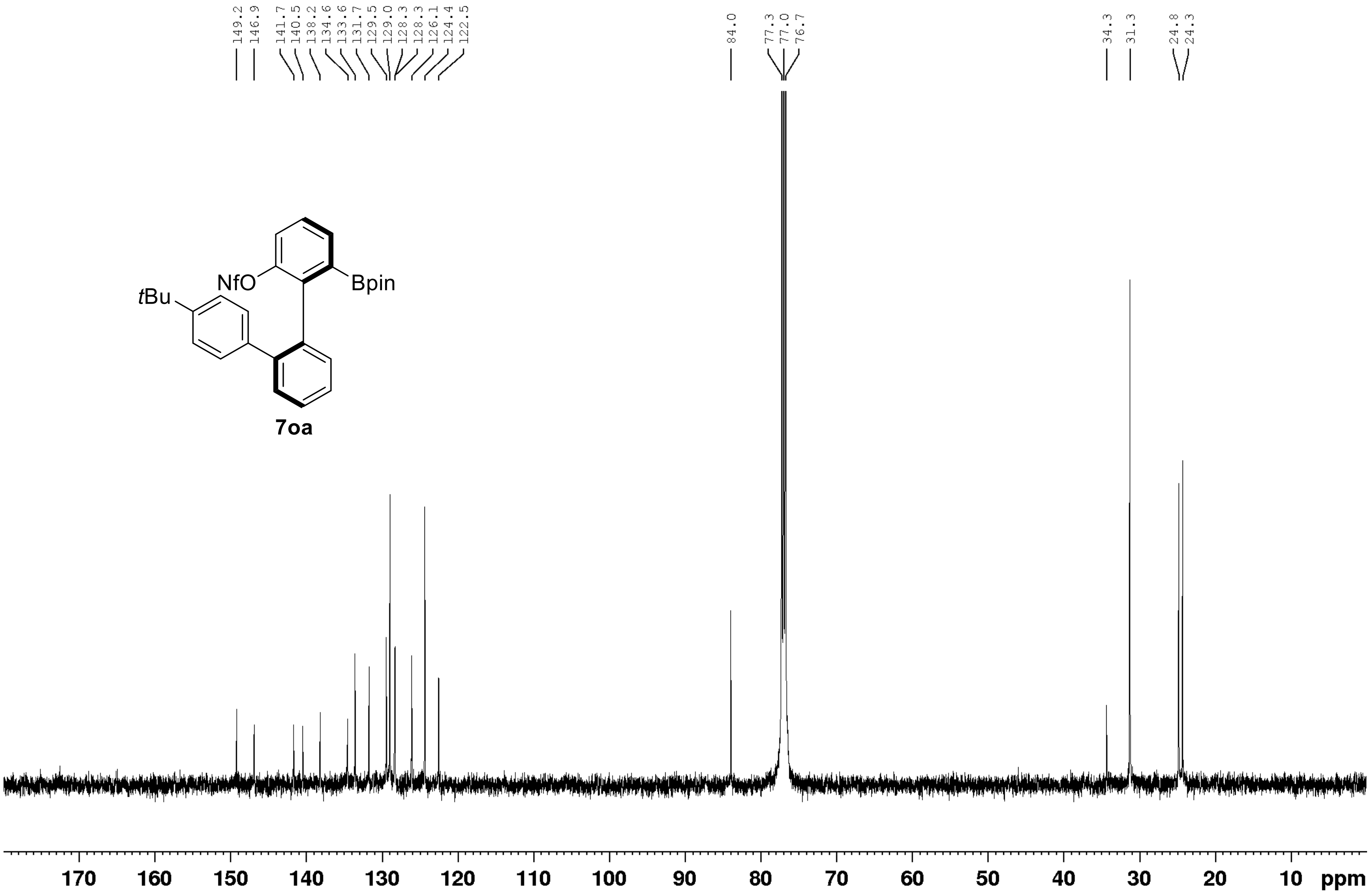

Figure S197. ^{19}**F NMR** (471 MHz, $CDCl_3$, 298 K) of **7oa**

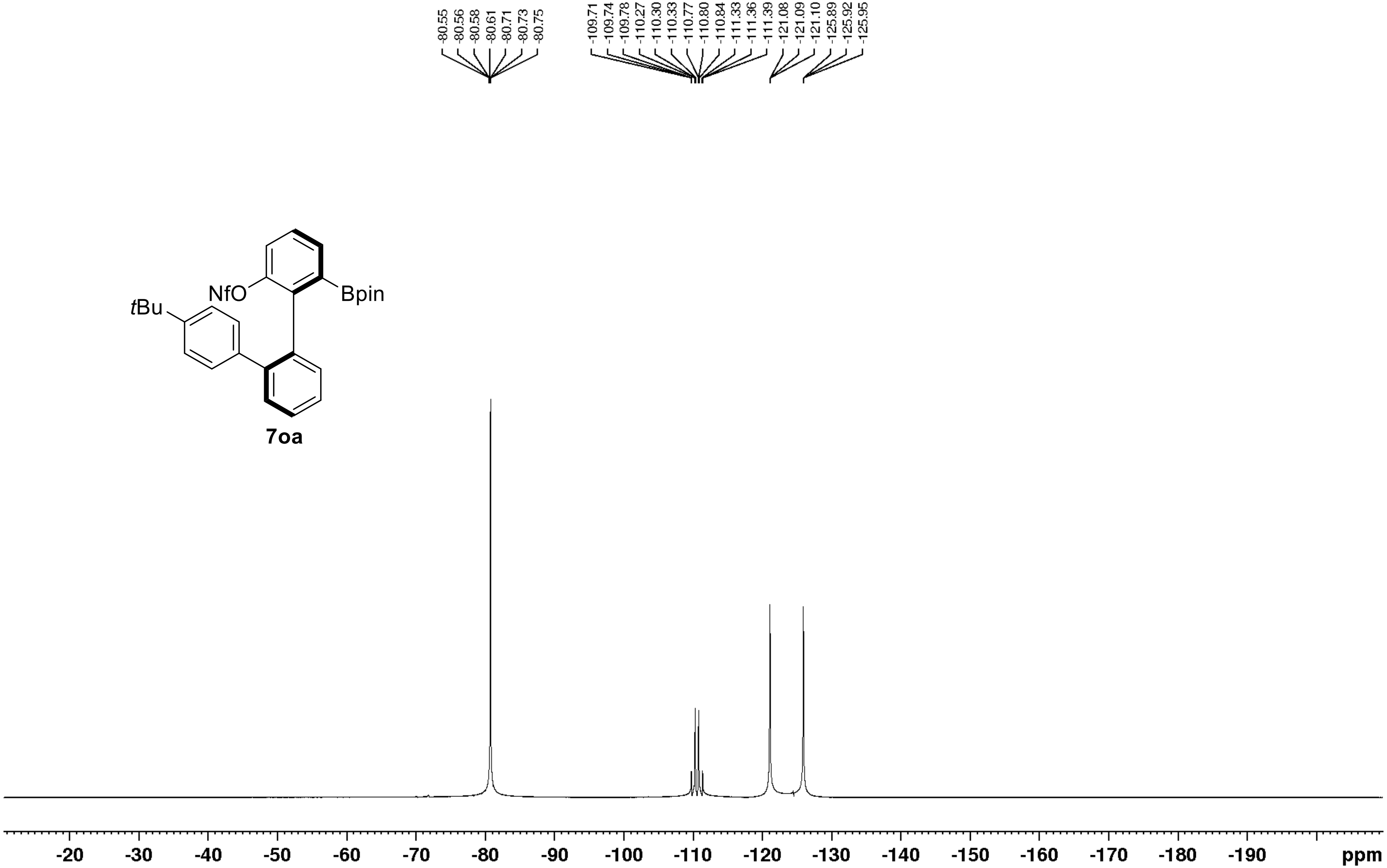

Figure S198. ^{11}B NMR (160 MHz, $CDCl_3$, 298 K) of **7oa**

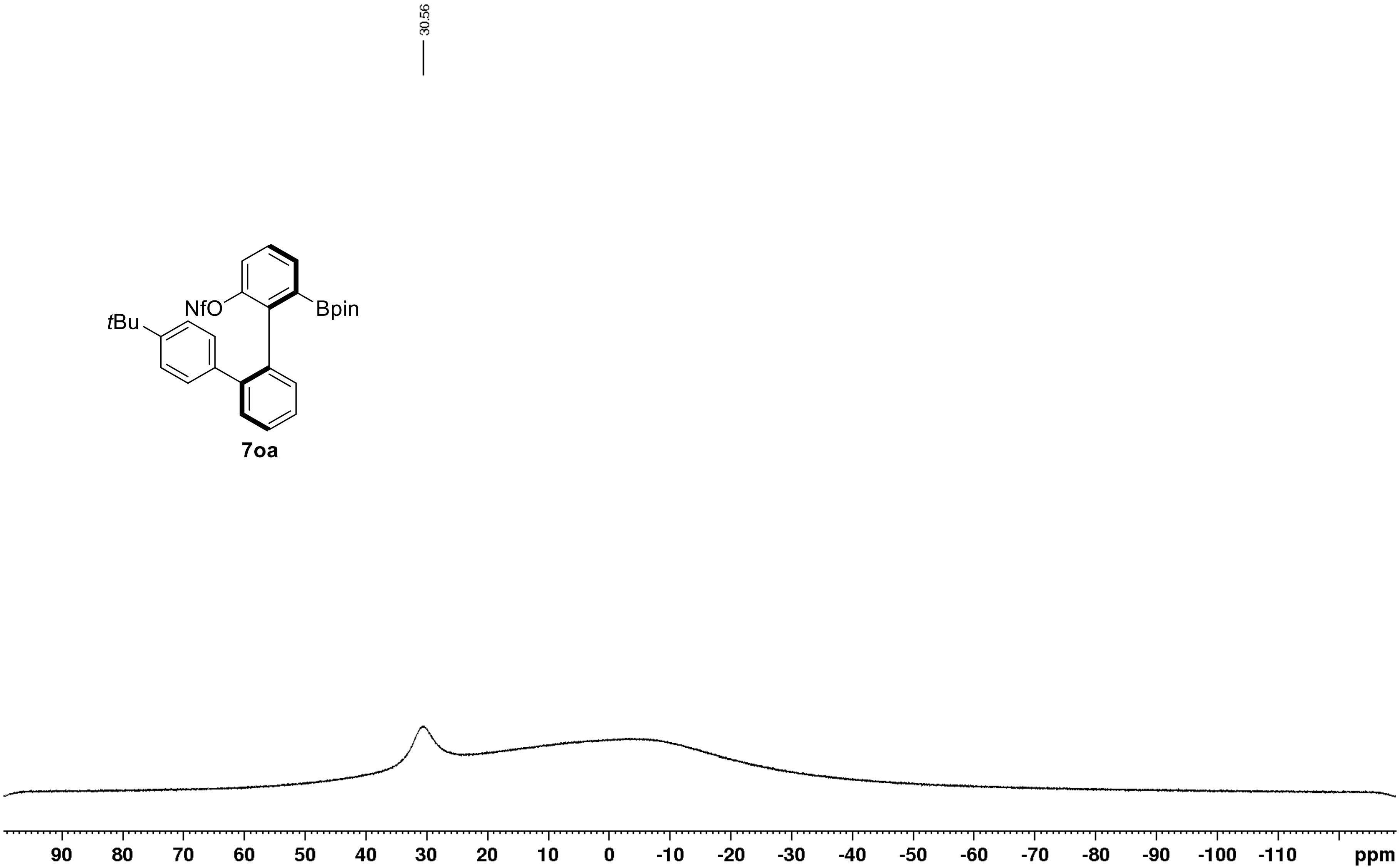

(*R*)-4''-acetyl-6-(4,4,5,5-tetramethyl-1,3,2-dioxaborolan-2-yl)-[1,1':2',1''-terphenyl]-2-yl 1,1,2,2,3,3,4,4,4-nonafluorobutane-1-sulfonate (7pa)

Figure S199. **^{1}H NMR** (500 MHz, $CDCl_3$, 298 K) of **7pa**

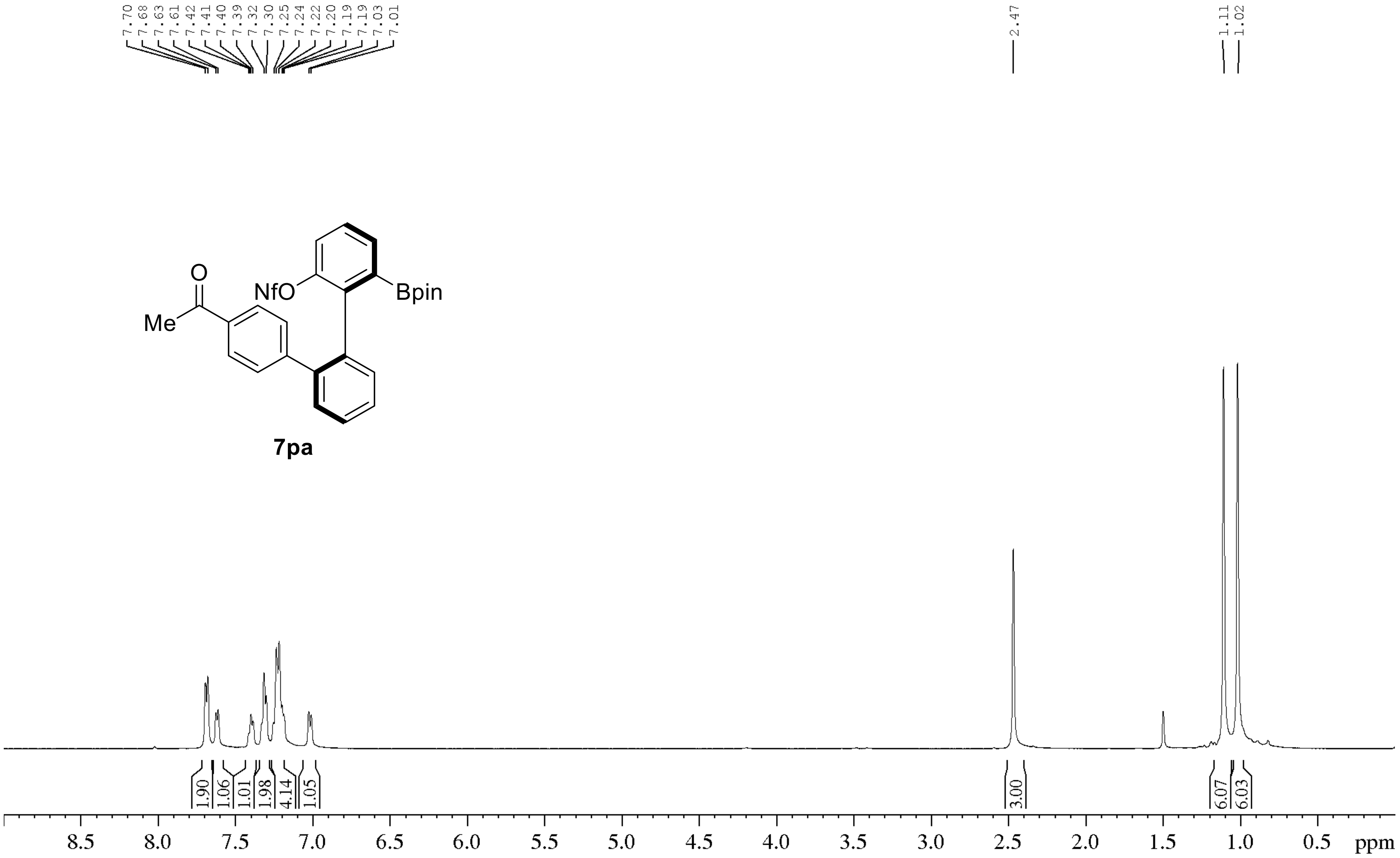

Figure S200. **$^{13}C\{^{1}H\}$ NMR** (126 MHz, $CDCl_3$, 298 K) of **7pa**

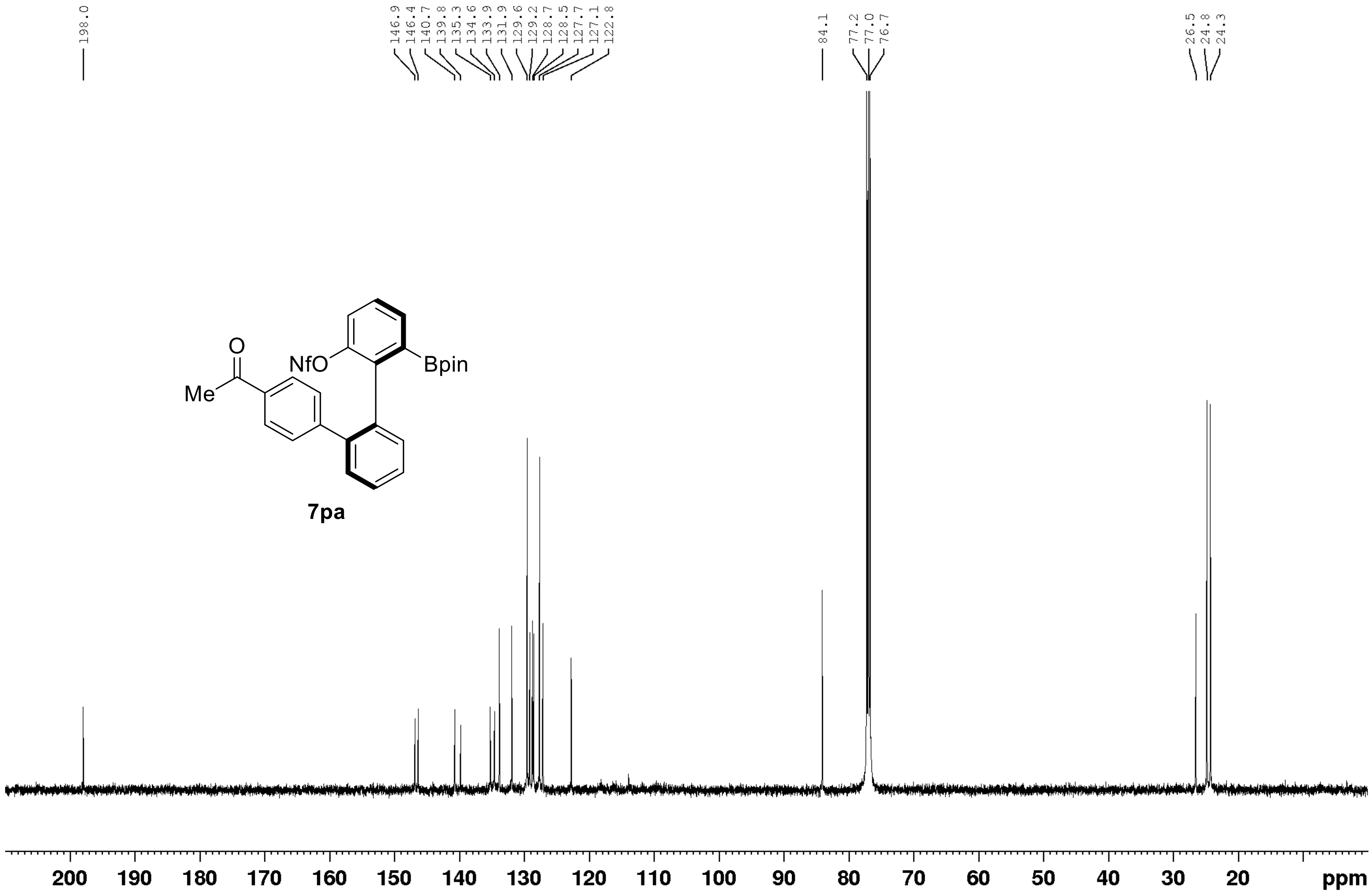

Figure S201. **^{19}F NMR** (471 MHz, $CDCl_3$, 298 K) of **7pa**

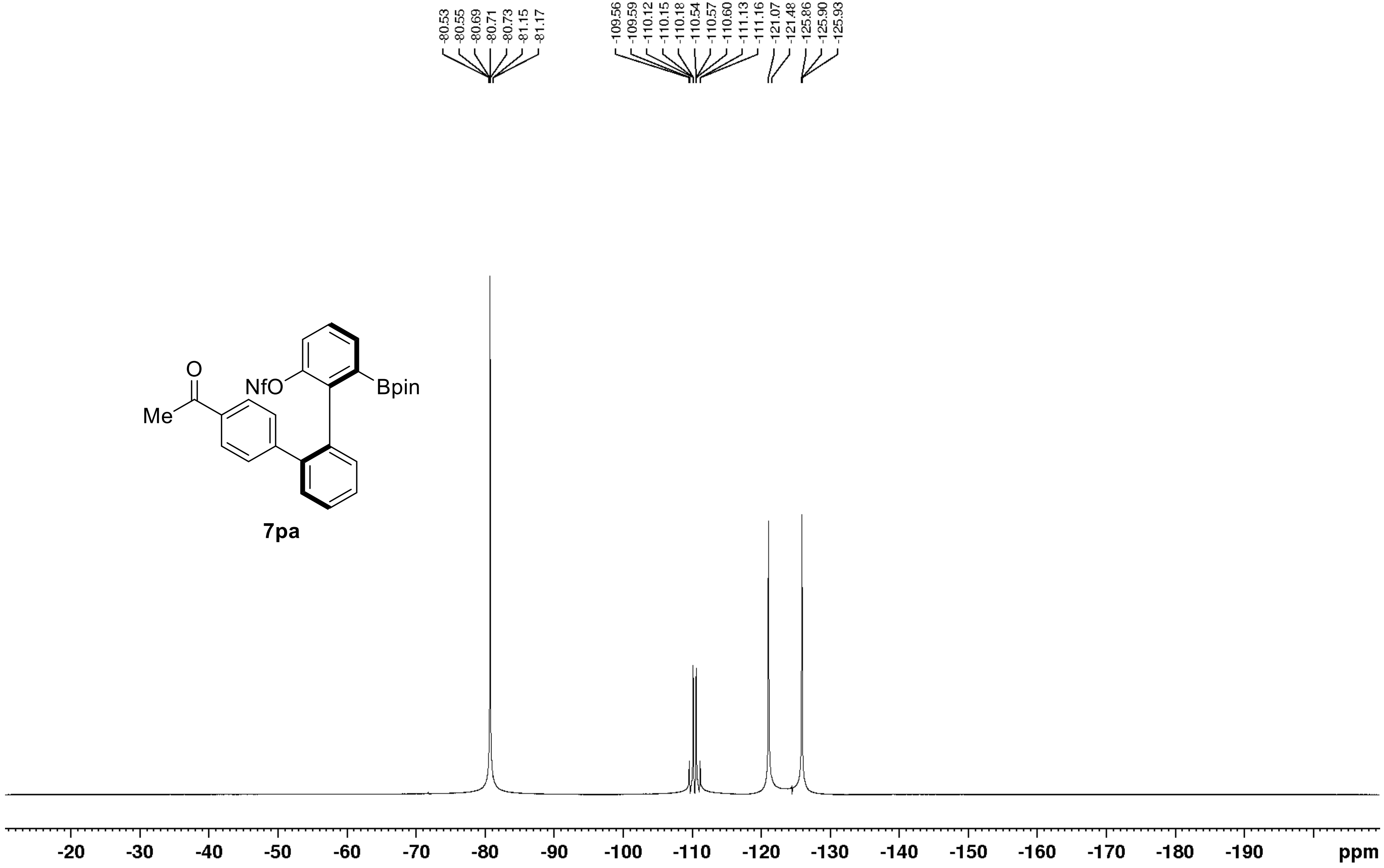

Figure S202. ^{11}B NMR (160 MHz, $CDCl_3$, 298 K) of **7pa**

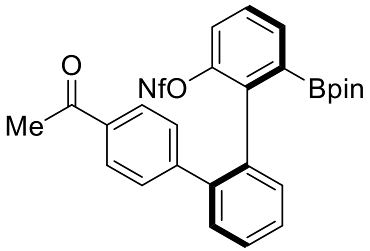

7pa

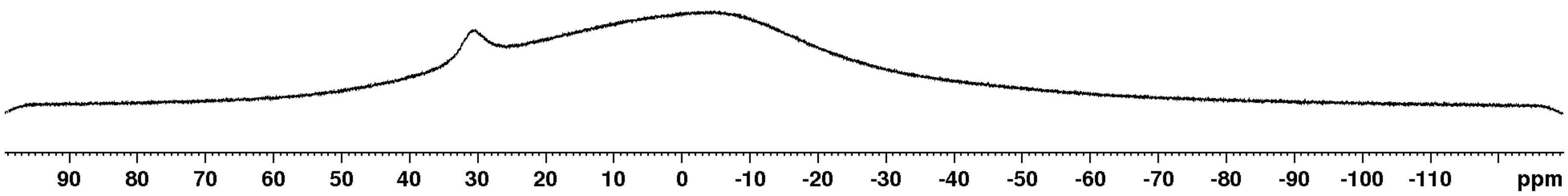

(*R*)-4''-nitro-6-(4,4,5,5-tetramethyl-1,3,2-dioxaborolan-2-yl)-[1,1':2',1''-terphenyl]-2-yl 1,1,2,2,3,3,4,4,4-nonafluorobutane-1-sulfonate (7qa)

Figure S203. ^{1}**H NMR** (500 MHz, $CDCl_3$, 298 K) of **7qa**

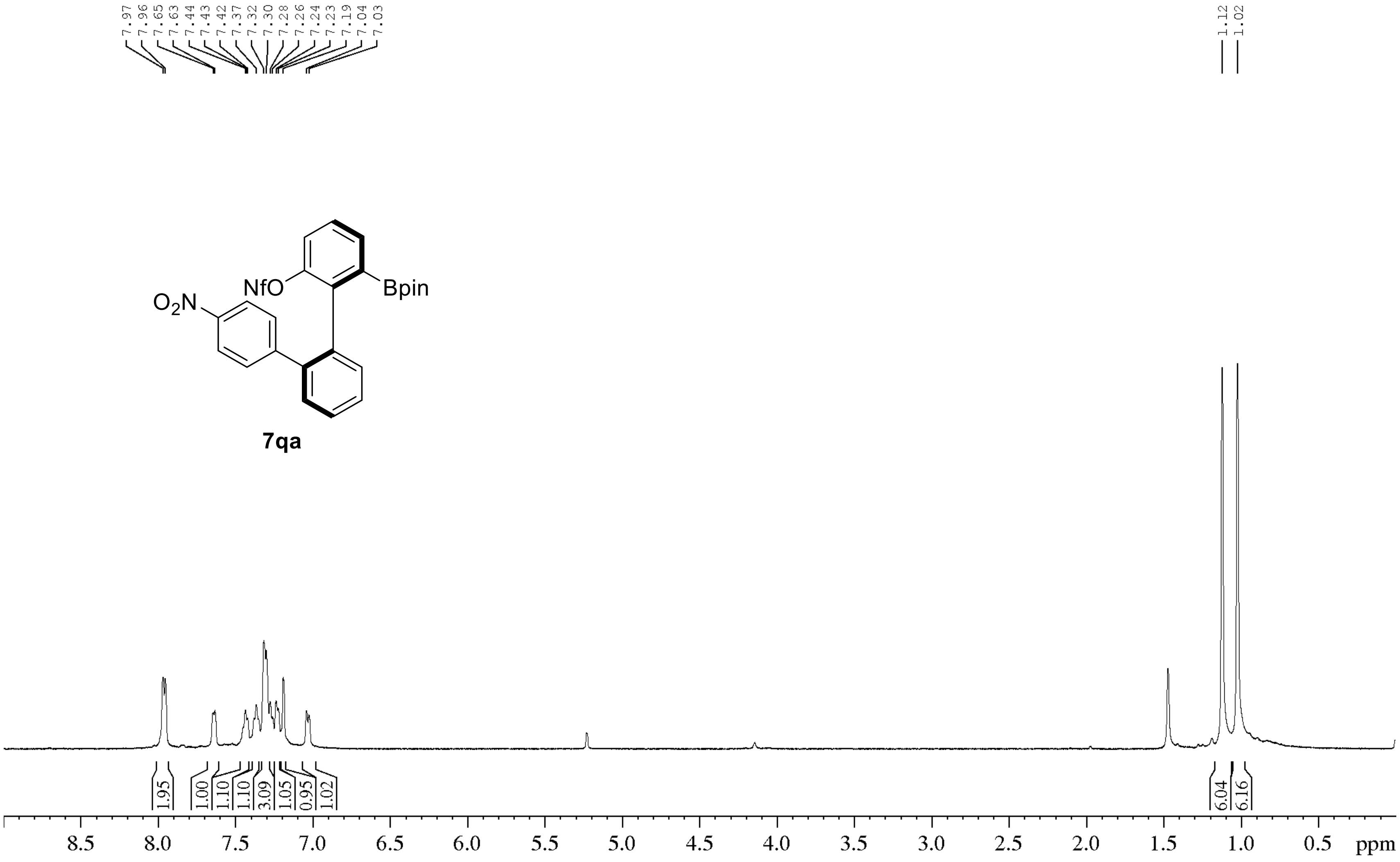

Figure S204. ^{13}**C{**^{1}**H} NMR** (101 MHz, $CDCl_3$, 298 K) of **7qa**

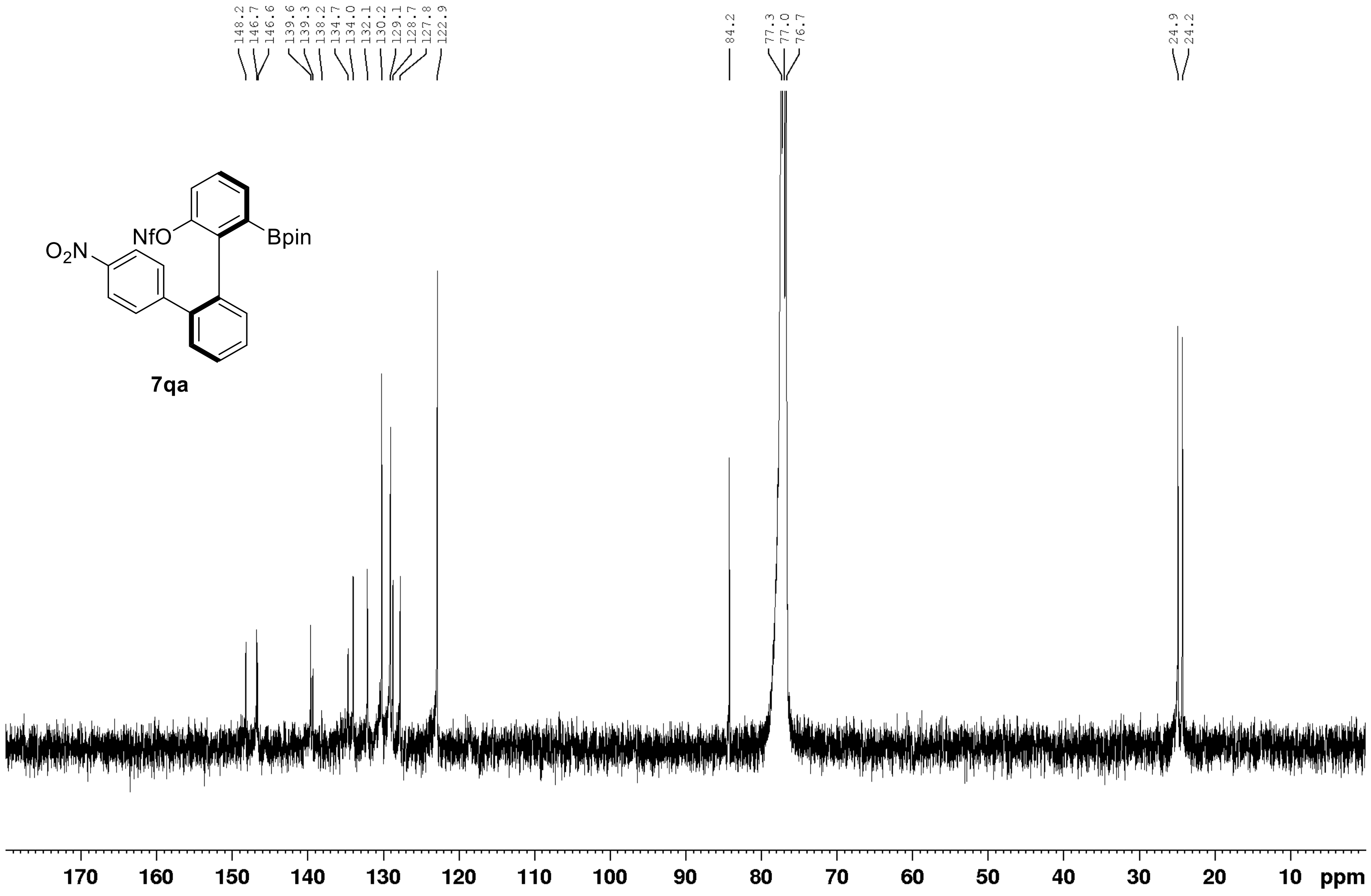

Figure S205. **^{19}F NMR** (471 MHz, $CDCl_3$, 298 K) of **7qa**

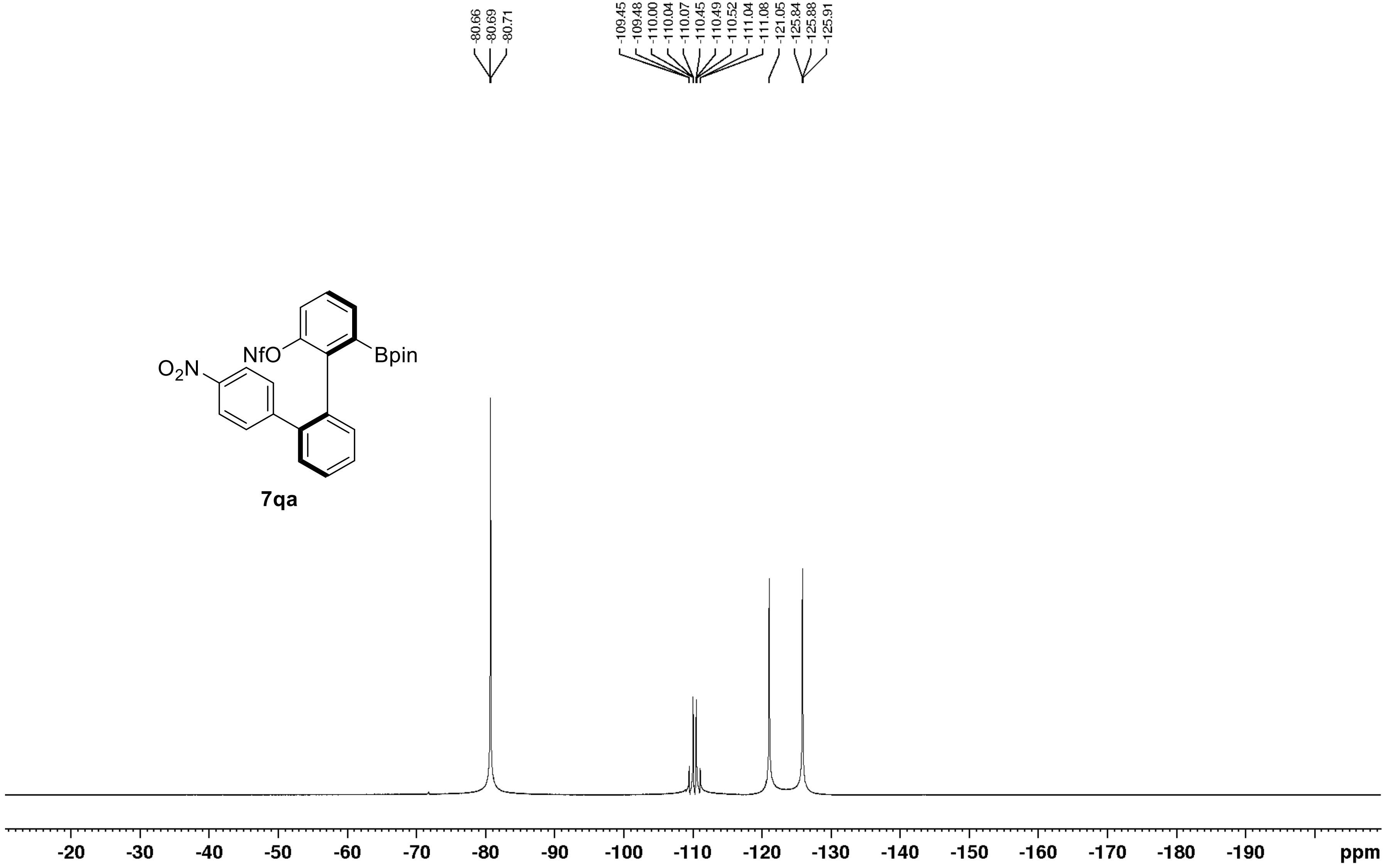

Figure S206. ^{11}**B NMR** (160 MHz, $CDCl_3$, 298 K) of **7qa**

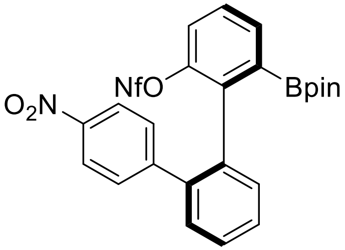

7qa

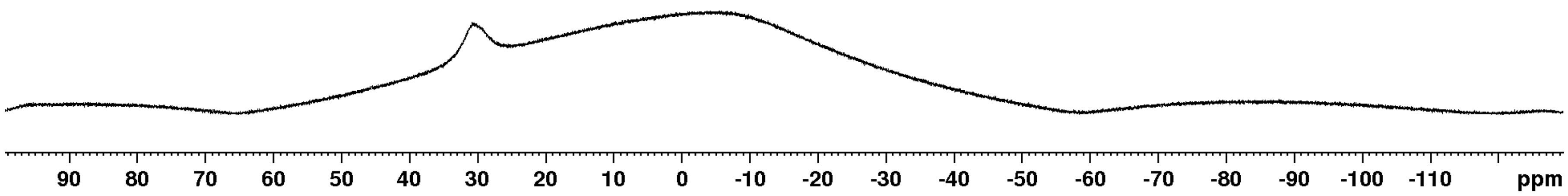

(*R*)-4'-chloro-6-(4,4,5,5-tetramethyl-1,3,2-dioxaborolan-2-yl)-[1,1':2',1''-terphenyl]-2-yl 1,1,2,2,3,3,4,4,4-nonafluorobutane-1-sulfonate (7ra)

Figure S207. ^{1}H NMR (500 MHz, $CDCl_3$, 298 K) of **7ra**

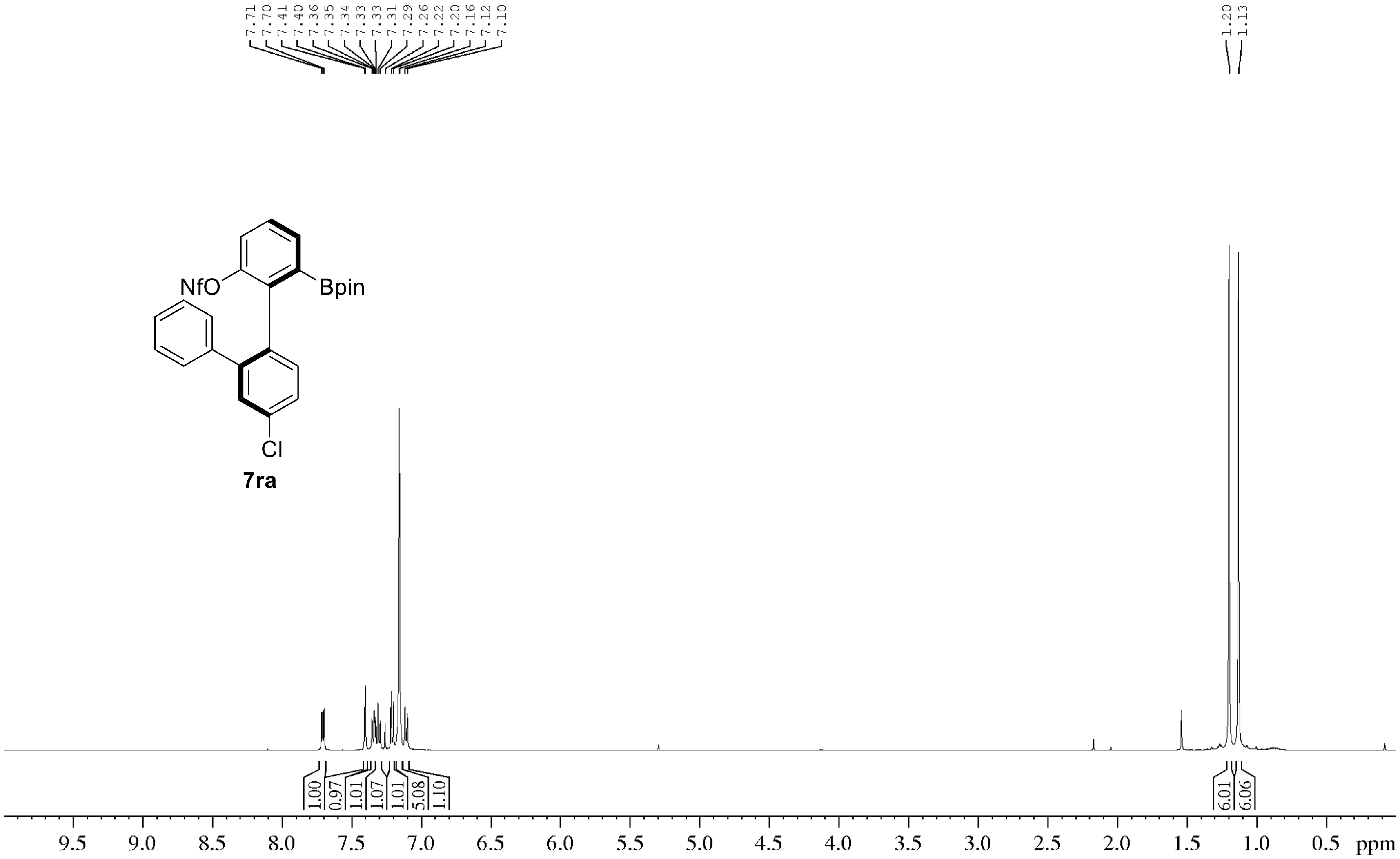

7ra

Figure S208. **$^{13}C\{^1H\}$ NMR** (101 MHz, $CDCl_3$, 298 K) of **7ra**

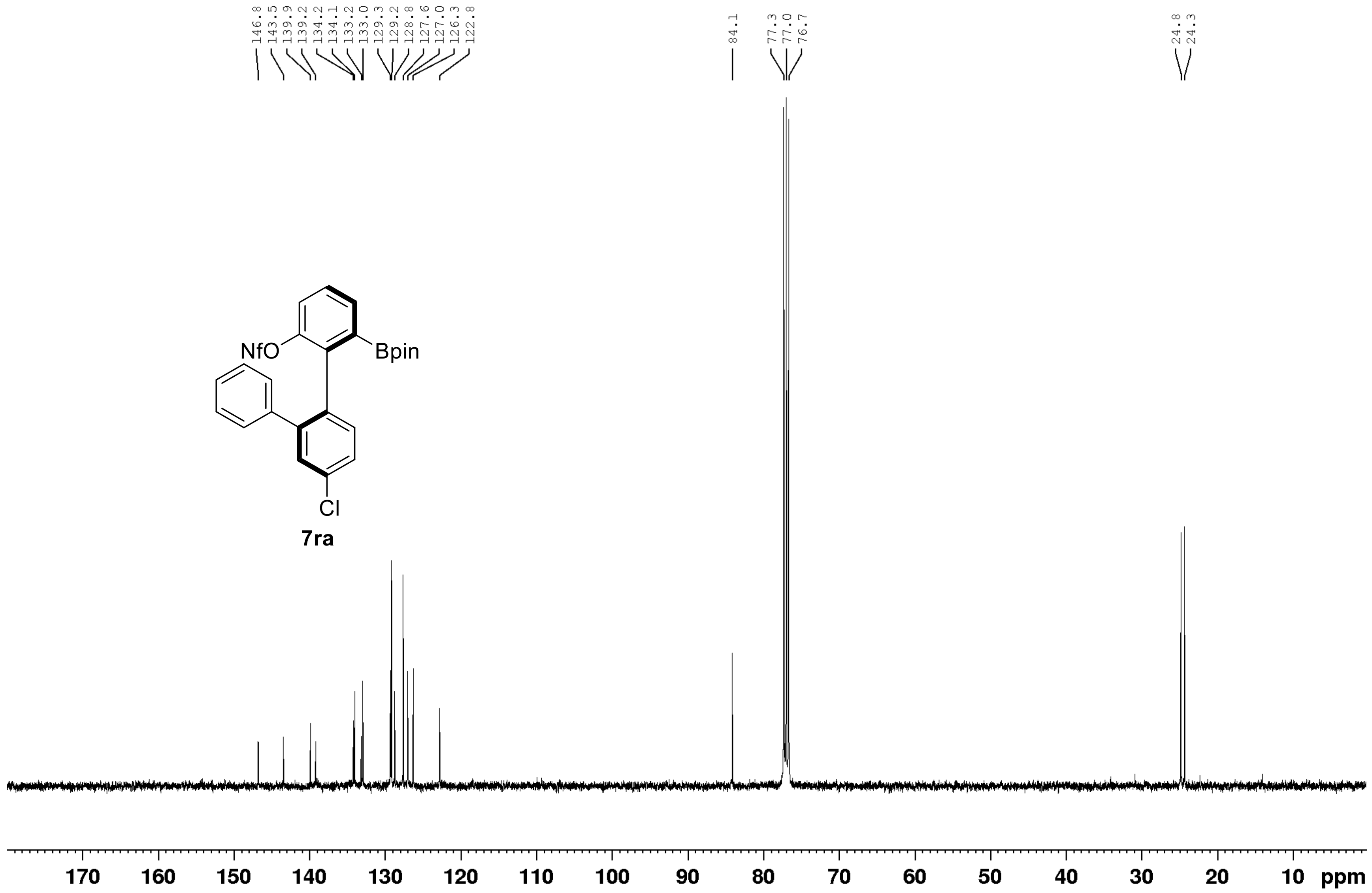

7ra

Figure S209. ^{19}F NMR (471 MHz, $CDCl_3$, 298 K) of **7ra**

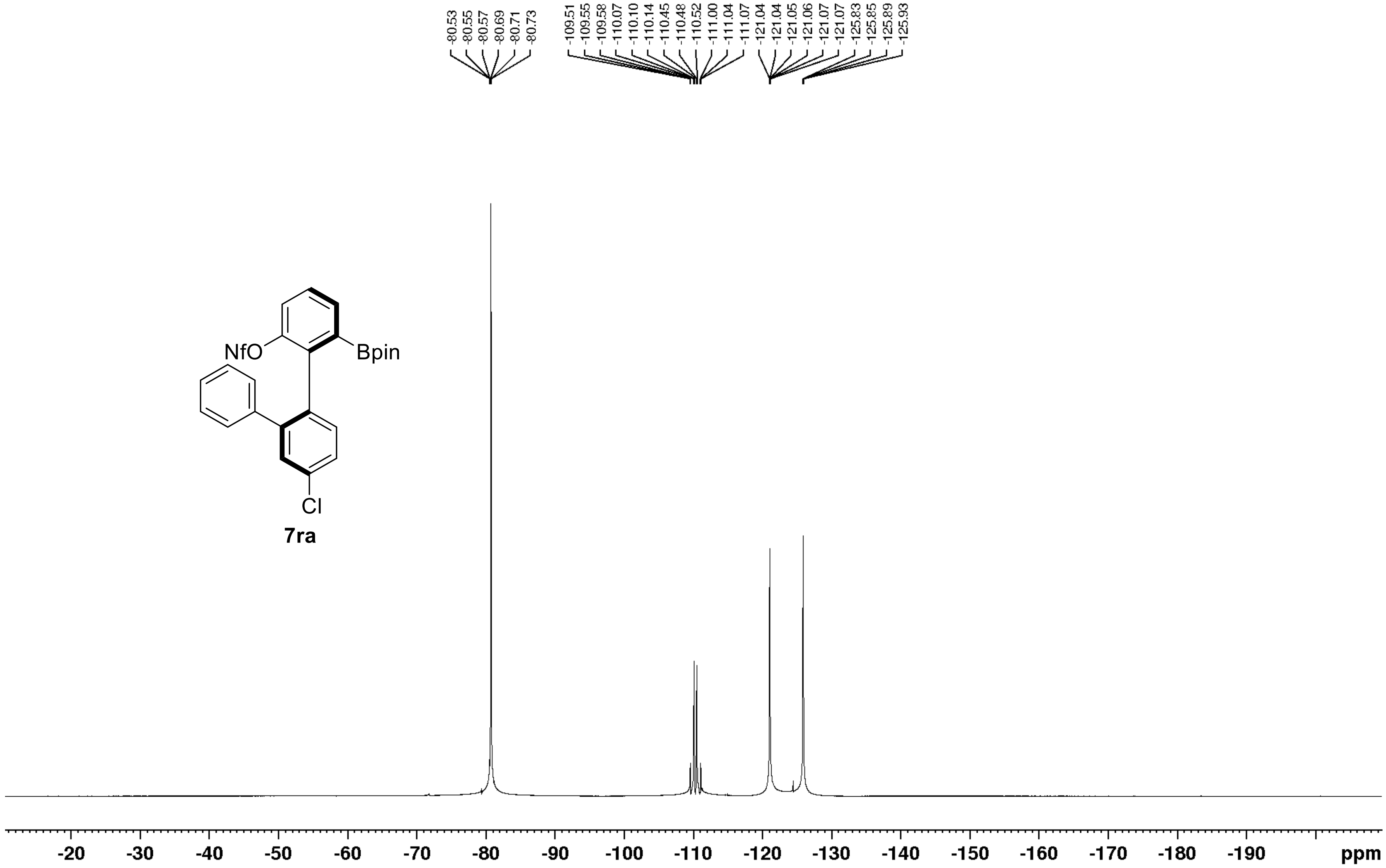

Figure S210. ^{11}B NMR (160 MHz, $CDCl_3$, 298 K) of **7ra**

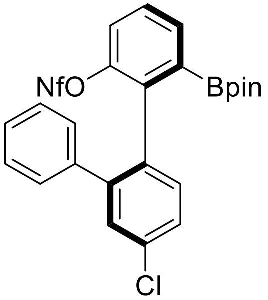

7ra

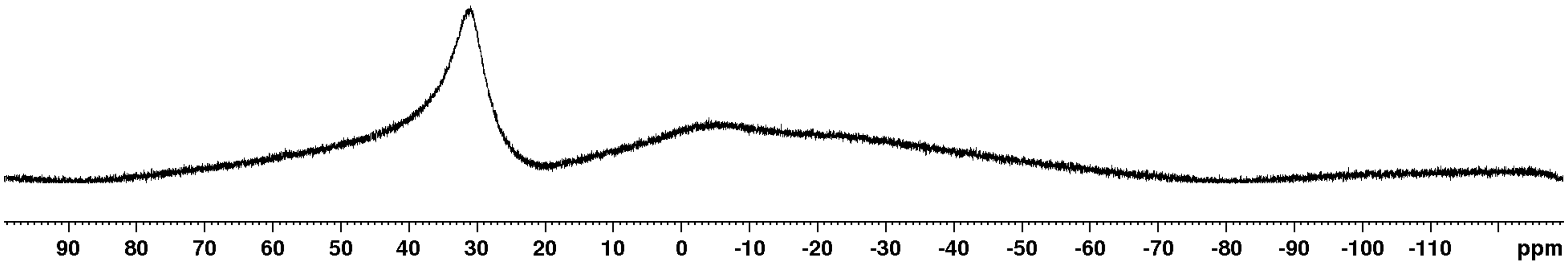

(*R*)-5'-methyl-6-(4,4,5,5-tetramethyl-1,3,2-dioxaborolan-2-yl)-[1,1':2',1''-terphenyl]-2-yl 1,1,2,2,3,3,4,4,4-nonafluorobutane-1-sulfonate (7sa)

Figure S211. ^{1}H NMR (500 MHz, $CDCl_3$, 298 K) of **7sa**

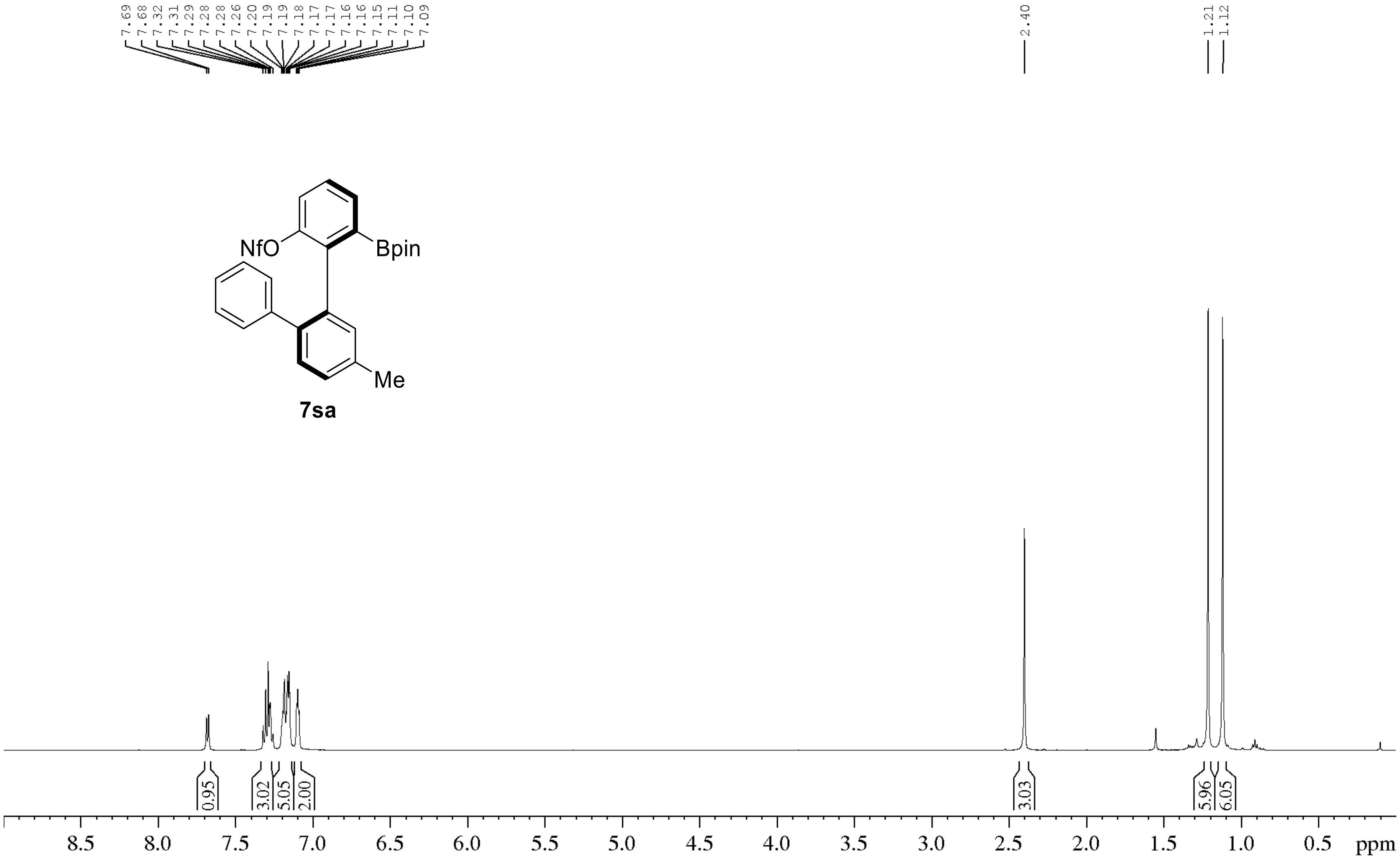

Figure S212. ^{13}**C{**^{1}**H} NMR** (126 MHz, $CDCl_3$, 298 K) of **7sa**

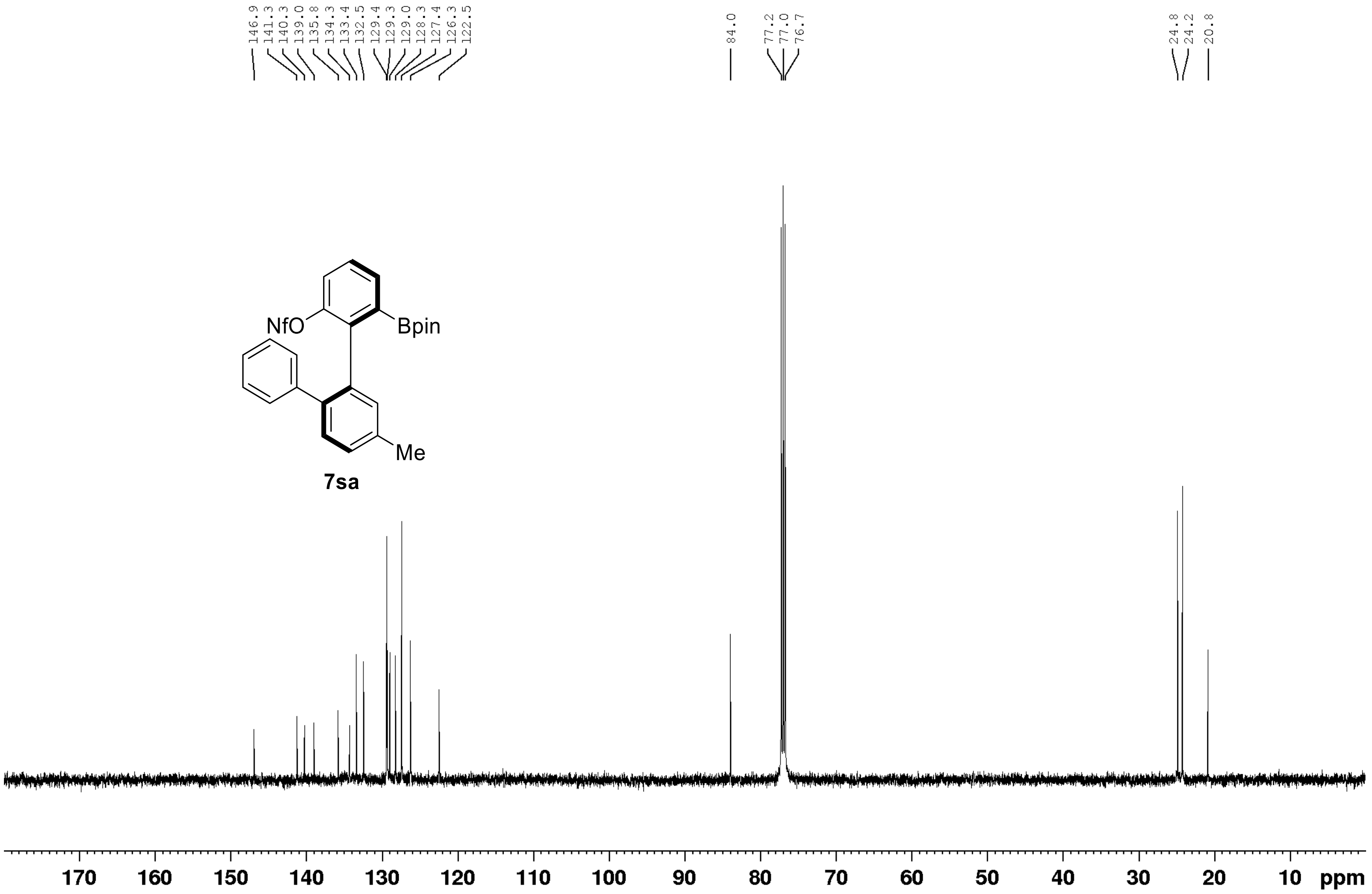

Figure S213. ^{19}**F NMR** (471 MHz, $CDCl_3$, 298 K) of **7sa**

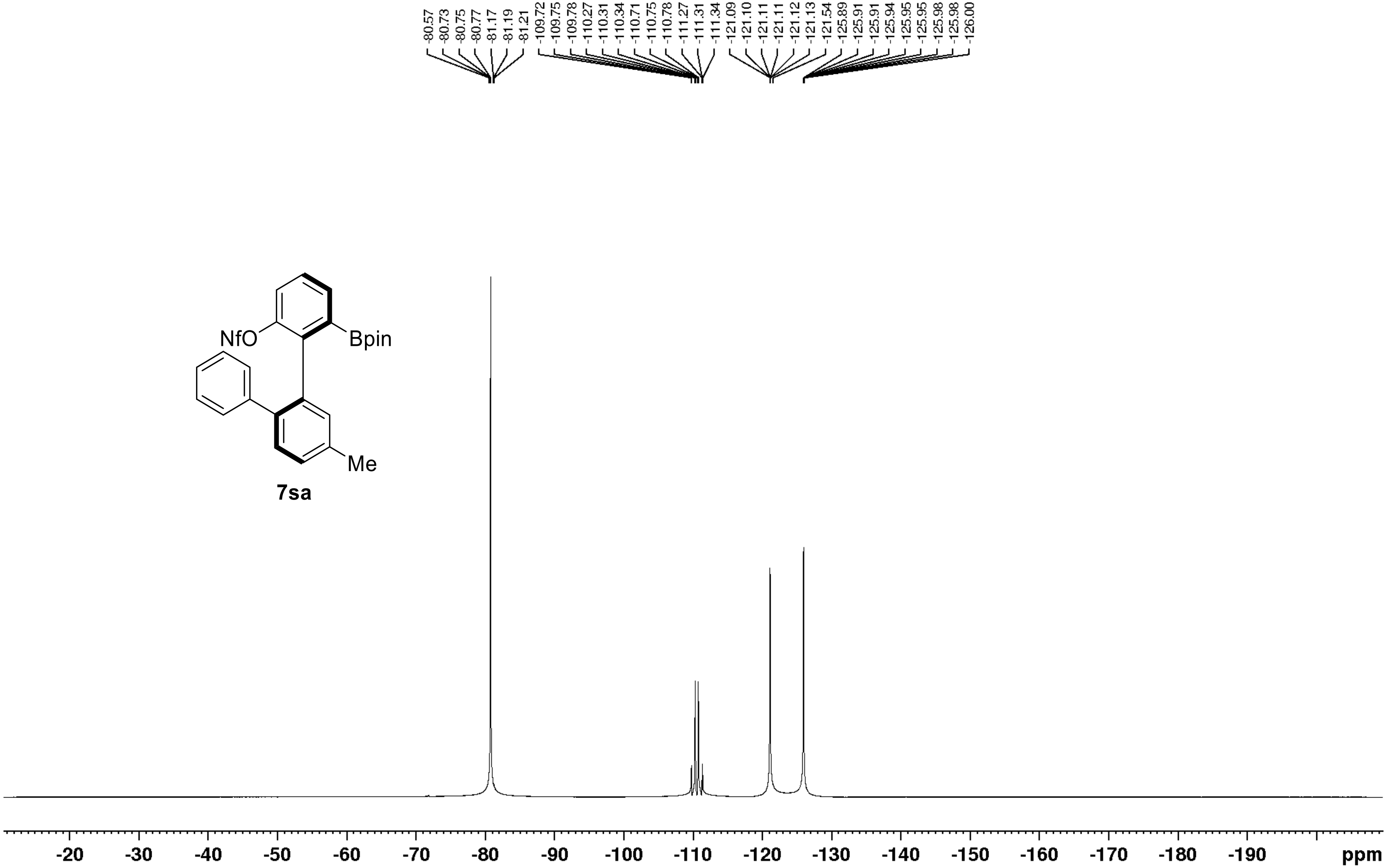

Figure S214. ^{11}**B NMR** (160 MHz, $CDCl_3$, 298 K) of **7sa**

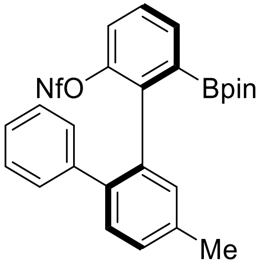

7sa

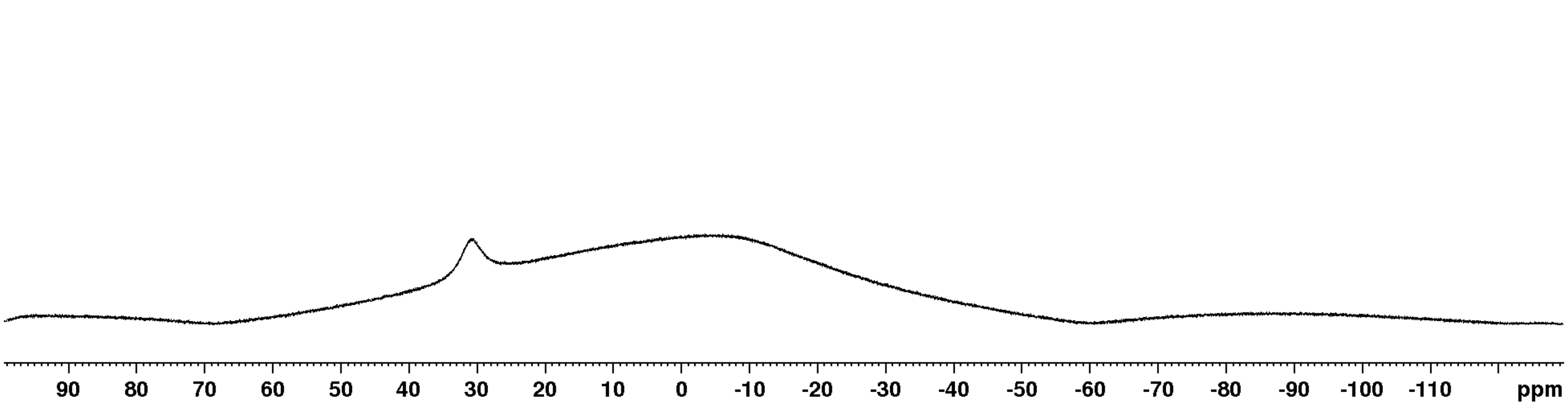

(*R*)-2'-(naphthalen-2-yl)-6-(4,4,5,5-tetramethyl-1,3,2-dioxaborolan-2-yl)-[1,1'-biphenyl]-2-yl 1,1,2,2,3,3,4,4,4-nonafluorobutane-1-sulfonate (7ta)

Figure S215. ^{1}**H NMR** (500 MHz, $CDCl_3$, 298 K) of **7ta**

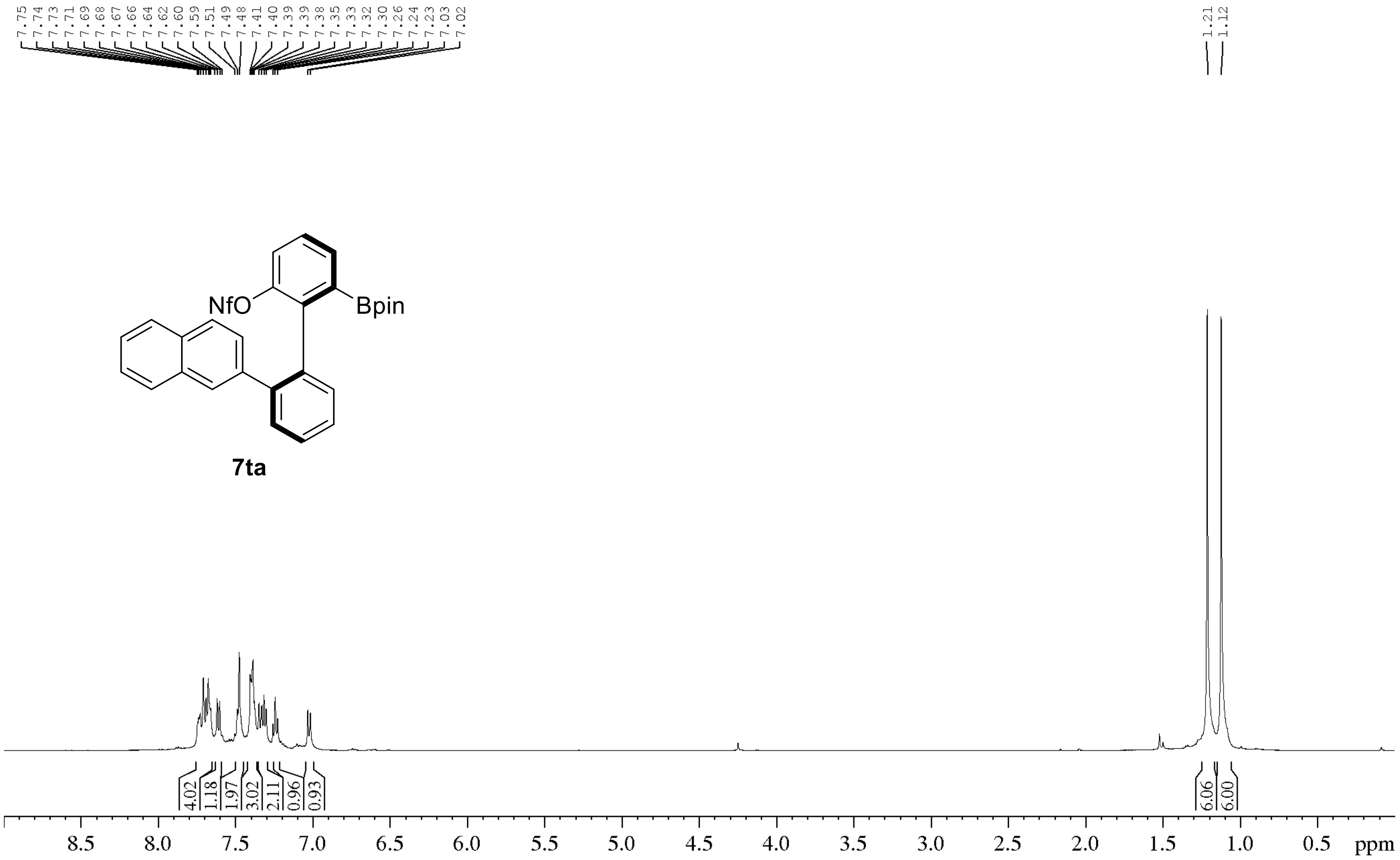

Figure S216. $^{13}C\{^{1}H\}$ NMR (101 MHz, $CDCl_3$, 298 K) of **7ta**

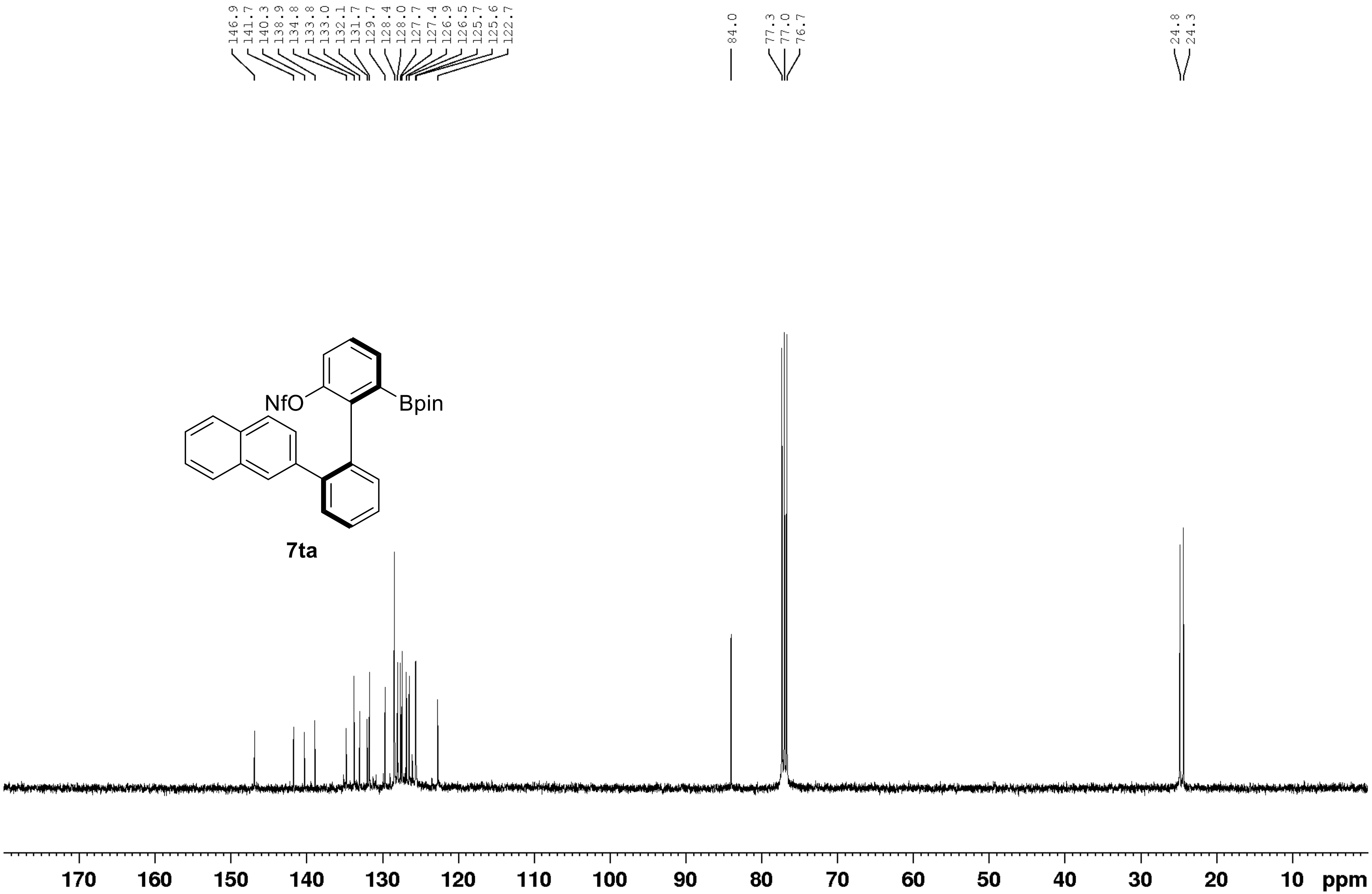

Figure S217. ^{19}F NMR (471 MHz, $CDCl_3$, 298 K) of **7ta**

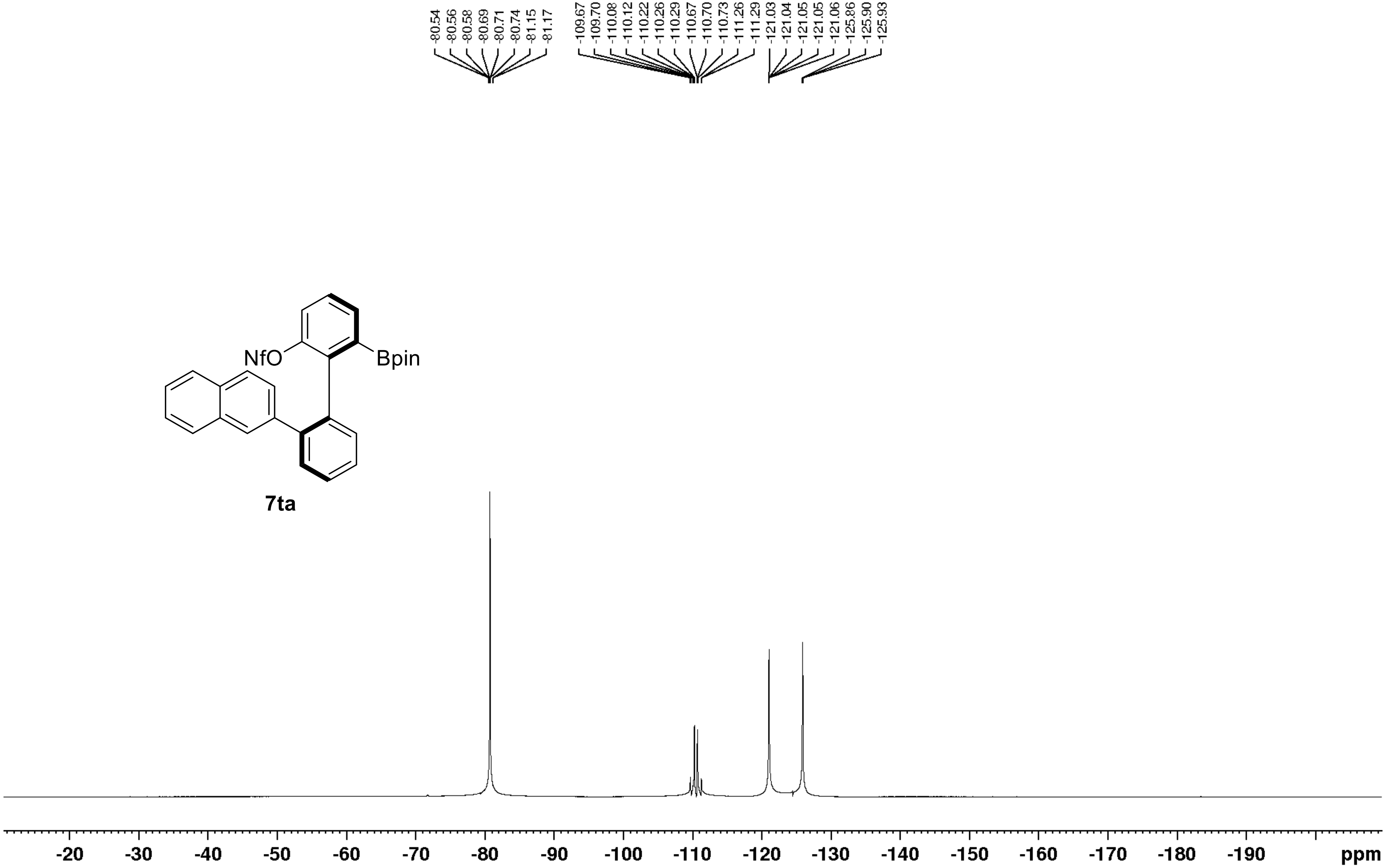

Figure S218. ^{11}B NMR (160 MHz, $CDCl_3$, 298 K) of **7ta**

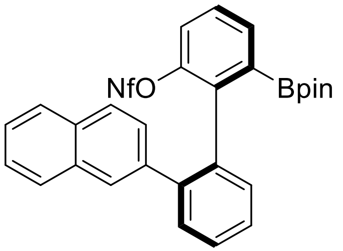

7ta

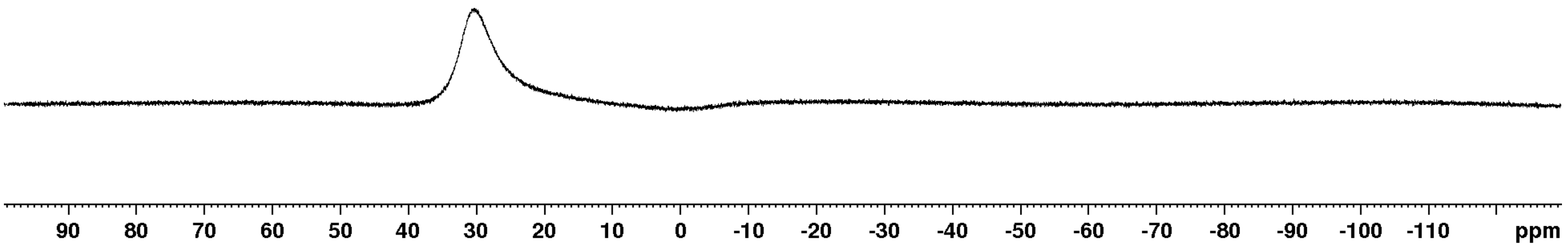

(*R*)-6-(4,4,5,5-tetramethyl-1,3,2-dioxaborolan-2-yl)-2'-(thiophen-3-yl)-[1,1'-biphenyl]-2-yl 1,1,2,2,3,3,4,4,4-nonafluorobutane-1-sulfonate (7ua)

Figure S219. ^{1}H NMR (500 MHz, $CDCl_3$, 298 K) of **7ua**

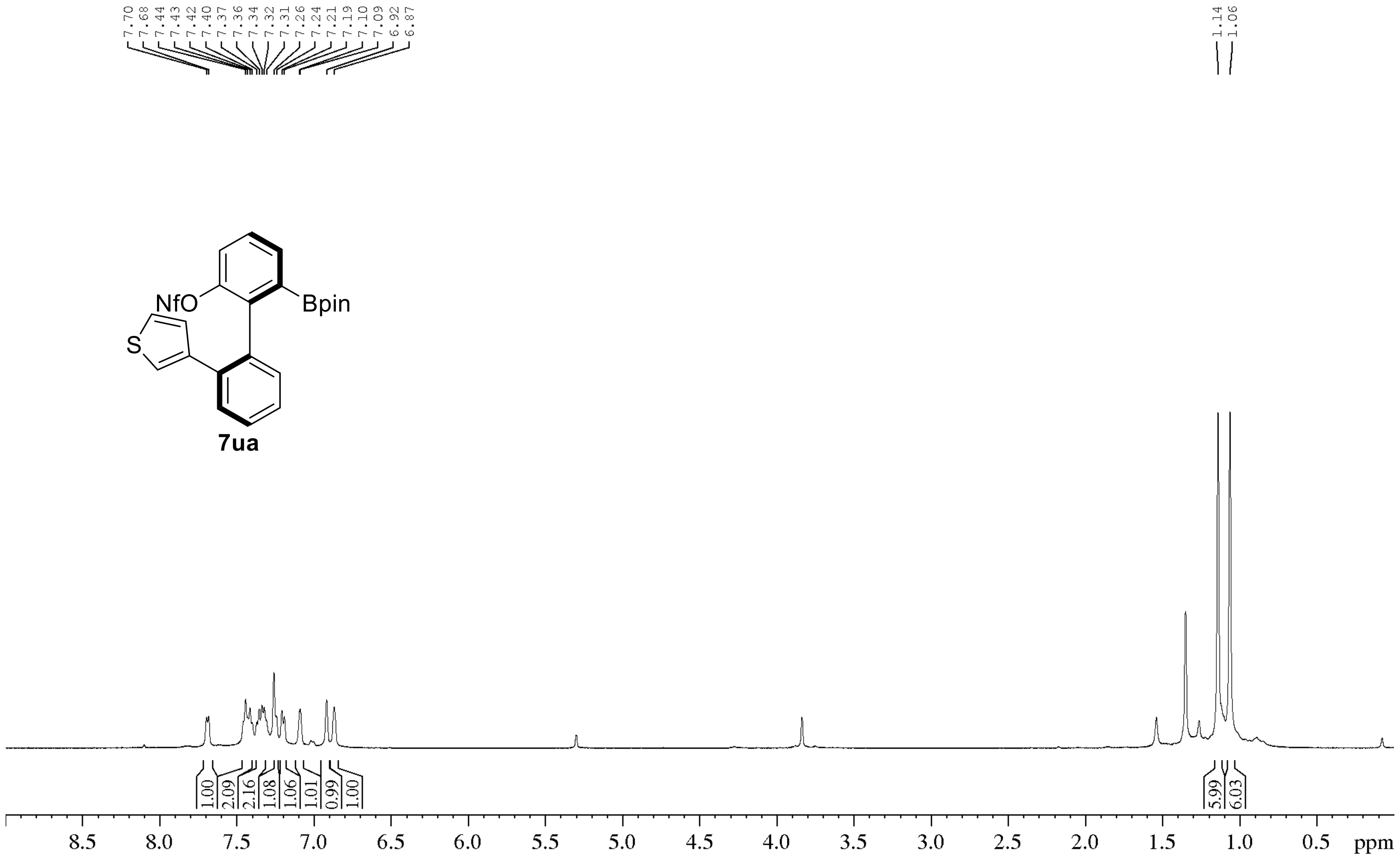

Figure S220. ^{13}**C{**^{1}**H} NMR** (126 MHz, $CDCl_3$, 298 K) of **7ua**

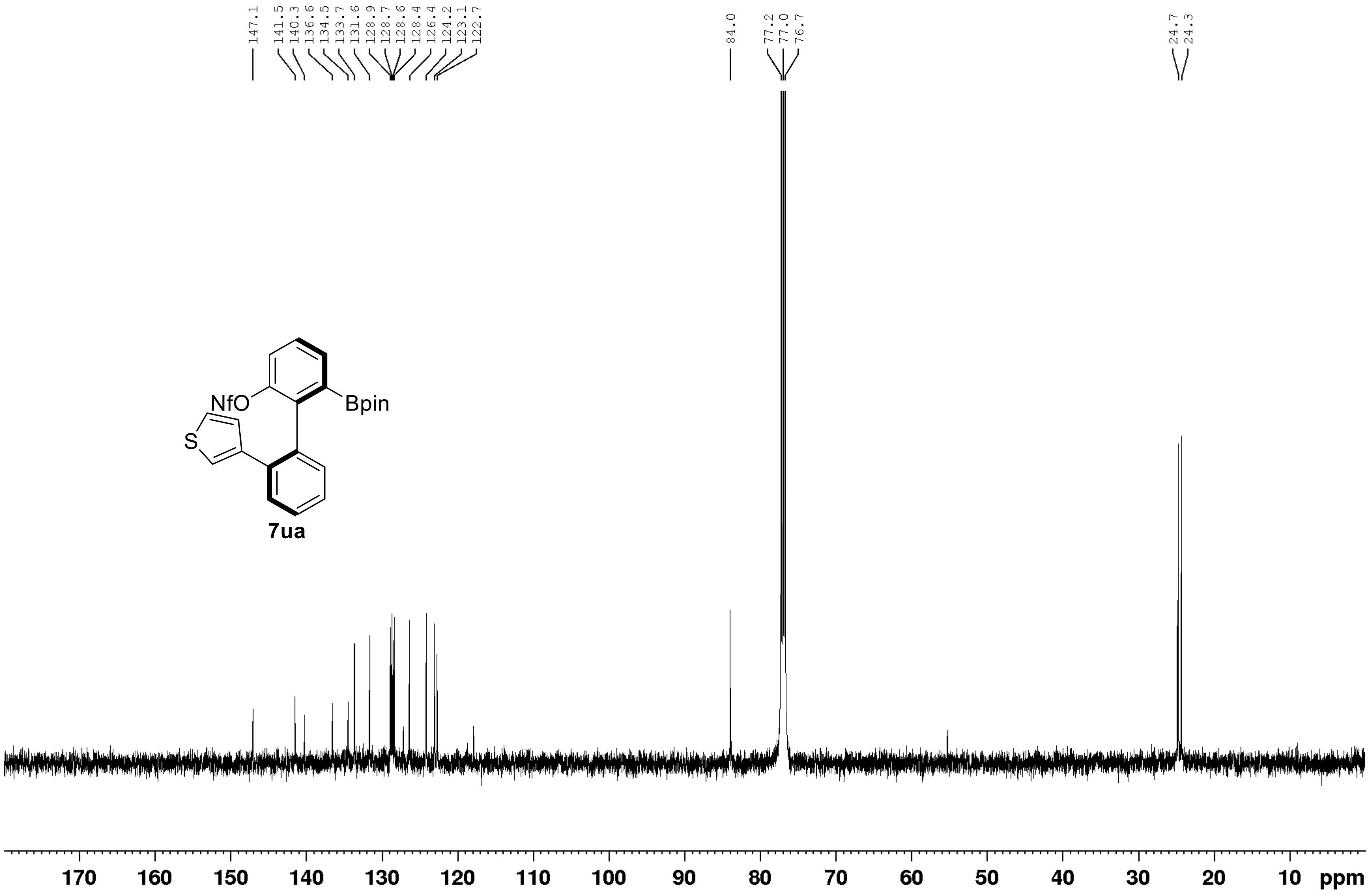

Figure S221. ^{19}**F NMR** (471 MHz, $CDCl_3$, 298 K) of **7ua**

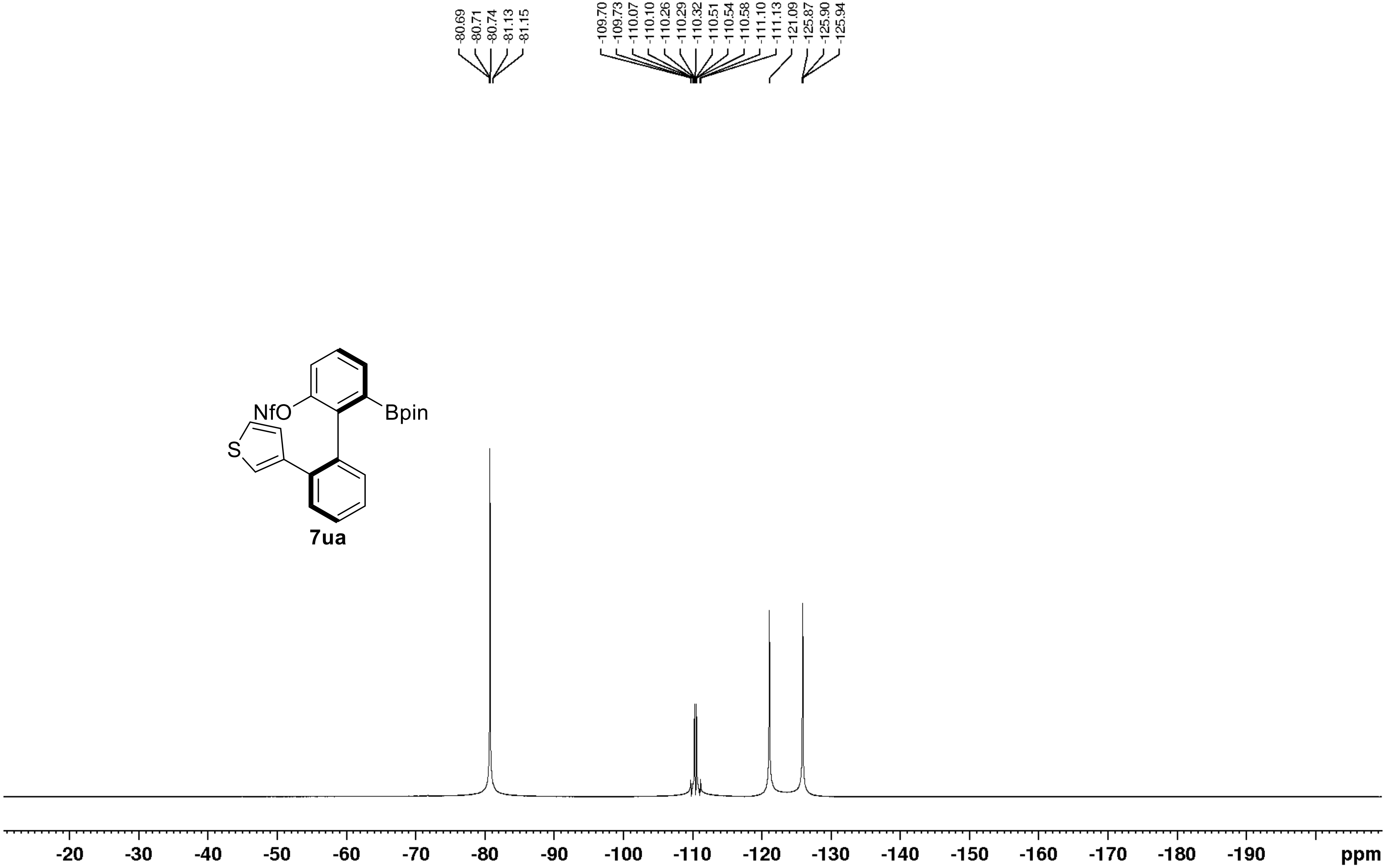

Figure S222. ^{11}B NMR (160 MHz, $CDCl_3$, 298 K) of **7ua**

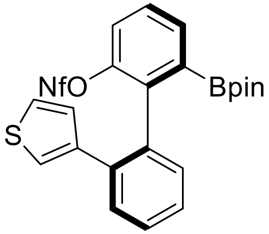

7ua

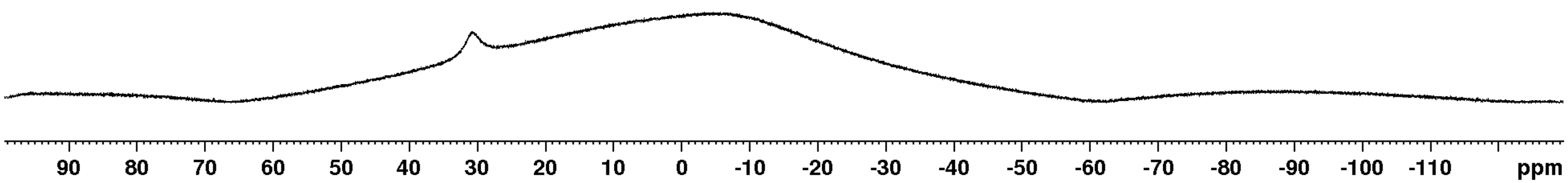

(*R*)-2'-cyano-6-(4,4,5,5-tetramethyl-1,3,2-dioxaborolan-2-yl)-[1,1'-biphenyl]-2-yl 1,1,2,2,3,3,4,4,4-nonafluorobutane-1-sulfonate (7va)

Figure S223. **^{1}H NMR** (500 MHz, $CDCl_3$, 298 K) of **7va**

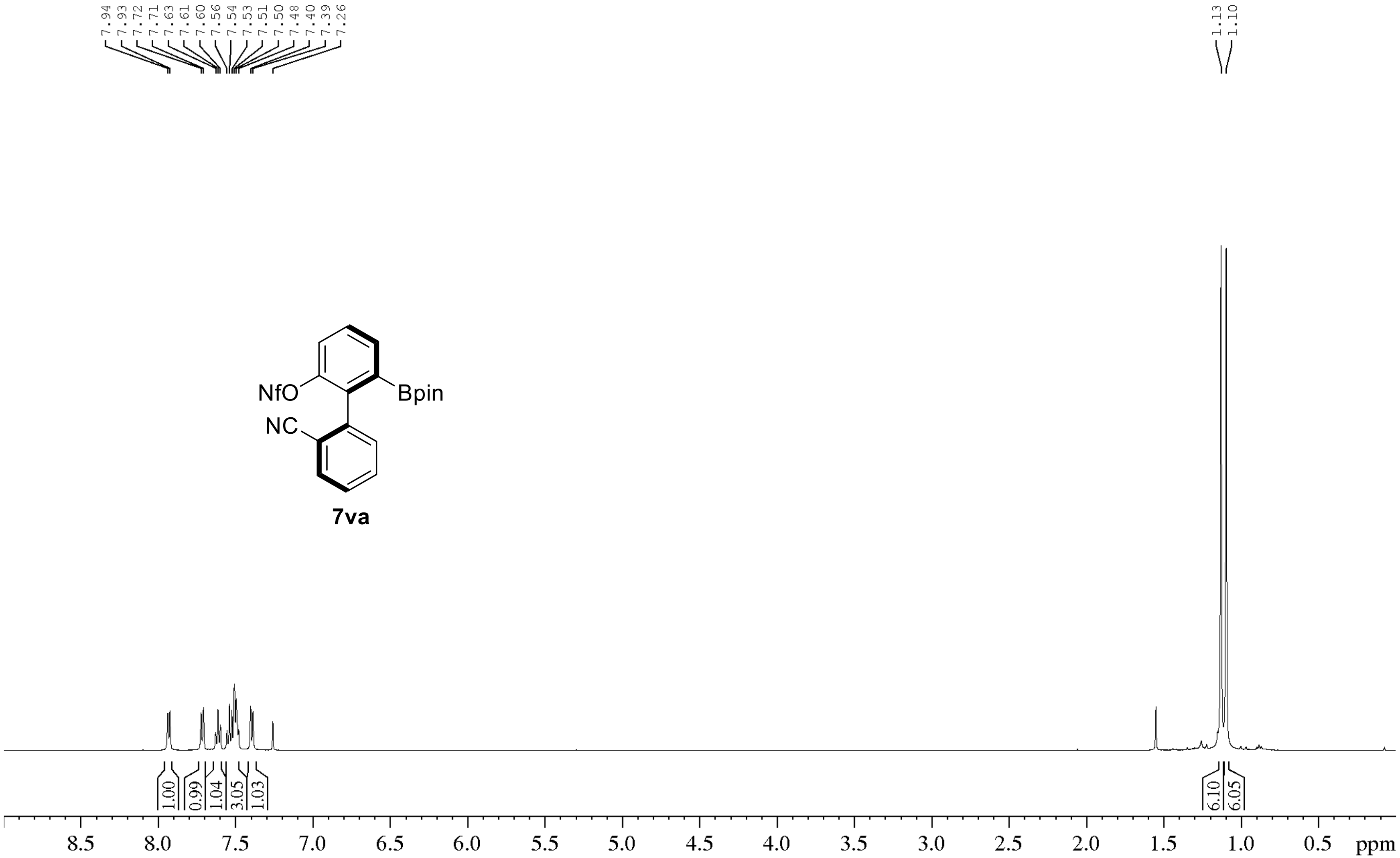

7va

Figure S224. **$^{13}C\{^1H\}$ NMR** (126 MHz, $CDCl_3$, 298 K) of **7va**

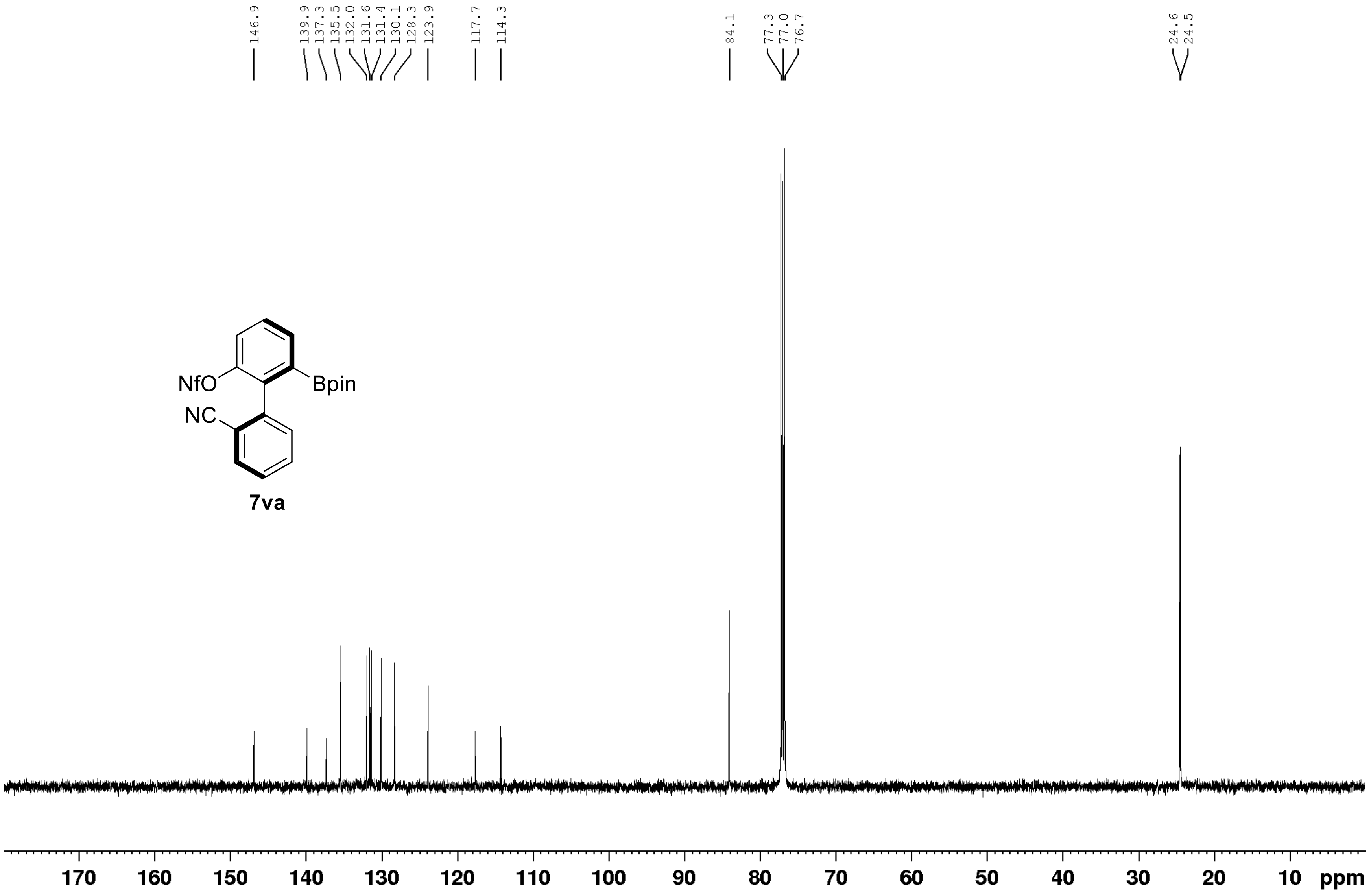

Figure S225. **^{19}F NMR** (471 MHz, $CDCl_3$, 298 K) of **7va**

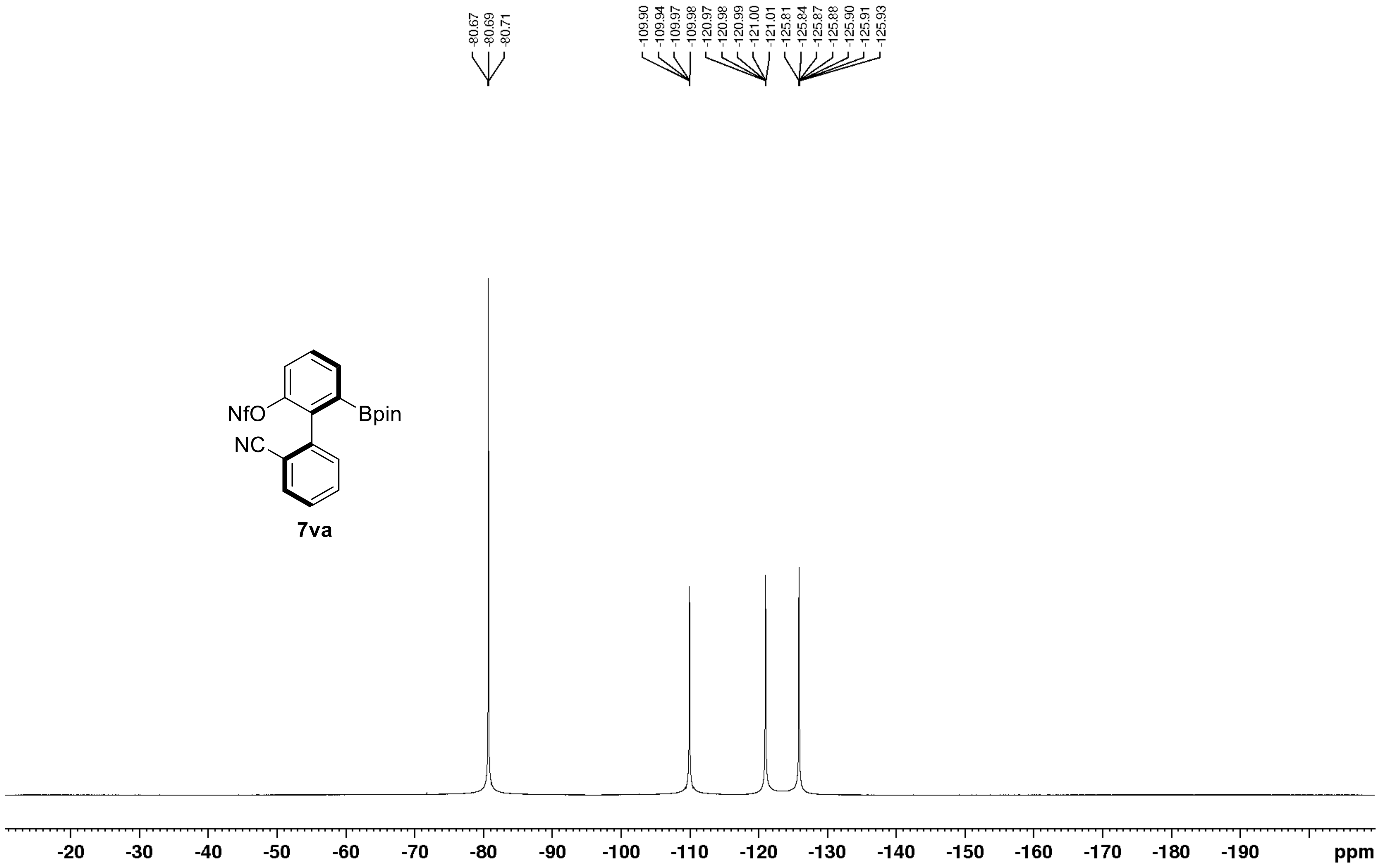

Figure S226. ^{11}B NMR (160 MHz, $CDCl_3$, 298 K) of **7va**

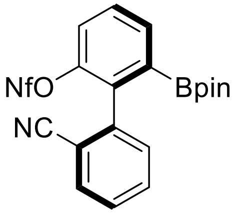

7va

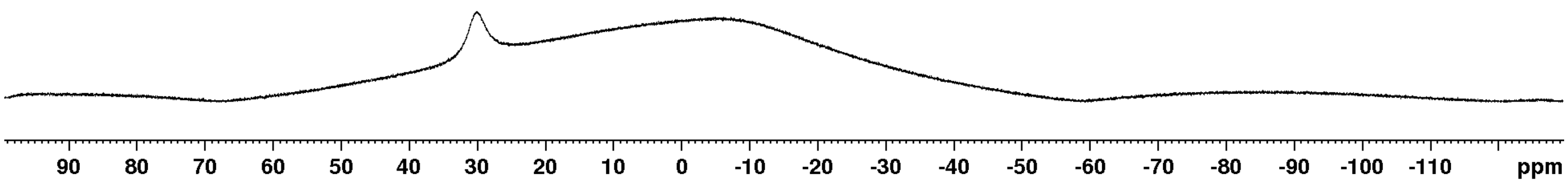

(*R*)-2'-isopropyl-6-(4,4,5,5-tetramethyl-1,3,2-dioxaborolan-2-yl)-[1,1'-biphenyl]-2-yl 1,1,2,2,3,3,4,4,4-nonafluorobutane-1-sulfonate (7wa)

Figure S227. **^{1}H NMR** (500 MHz, $CDCl_3$, 298 K) of **7wa**

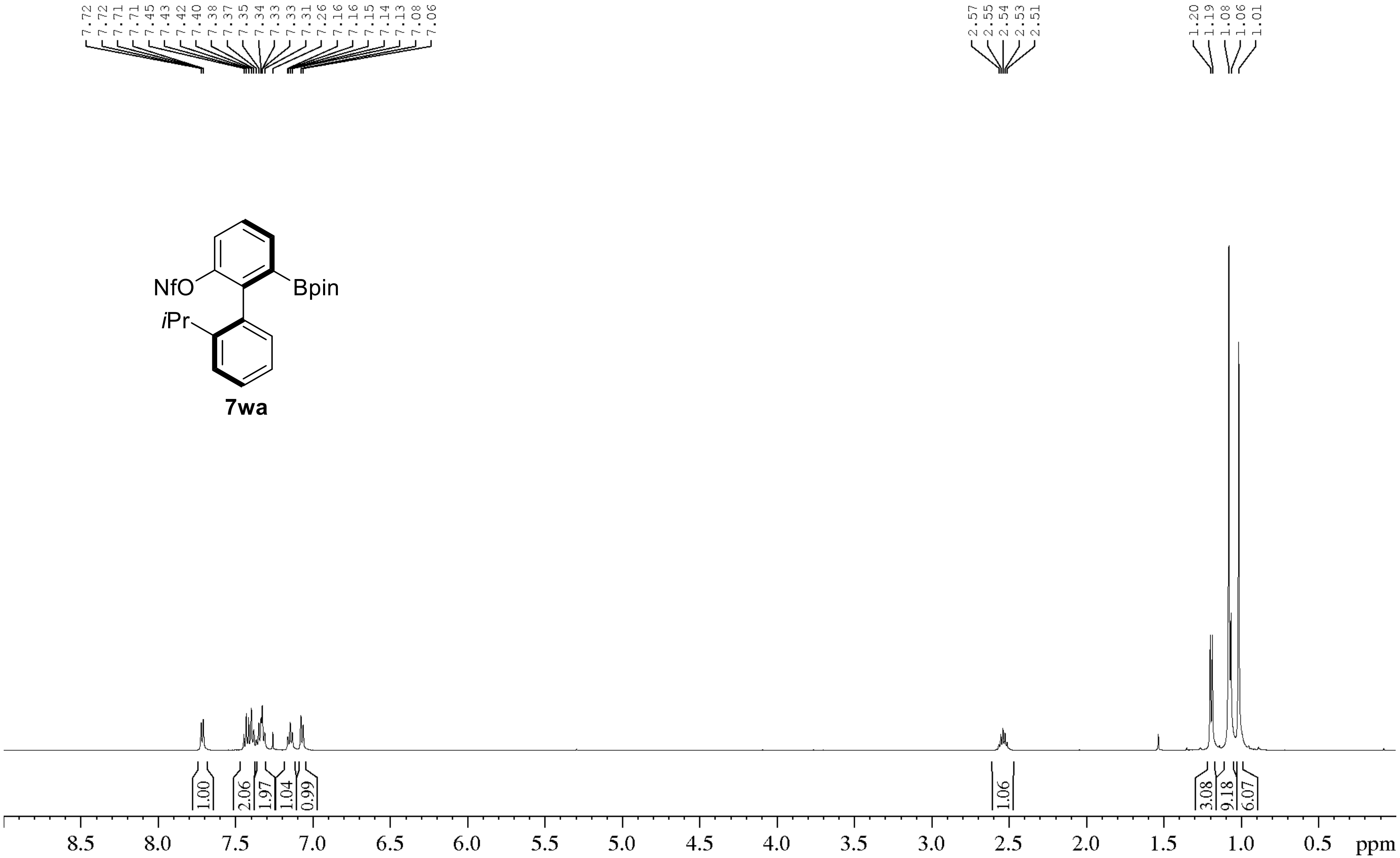

Figure S228. ^{13}**C{**^{1}**H} NMR** (126 MHz, $CDCl_3$, 298 K) of **7wa**

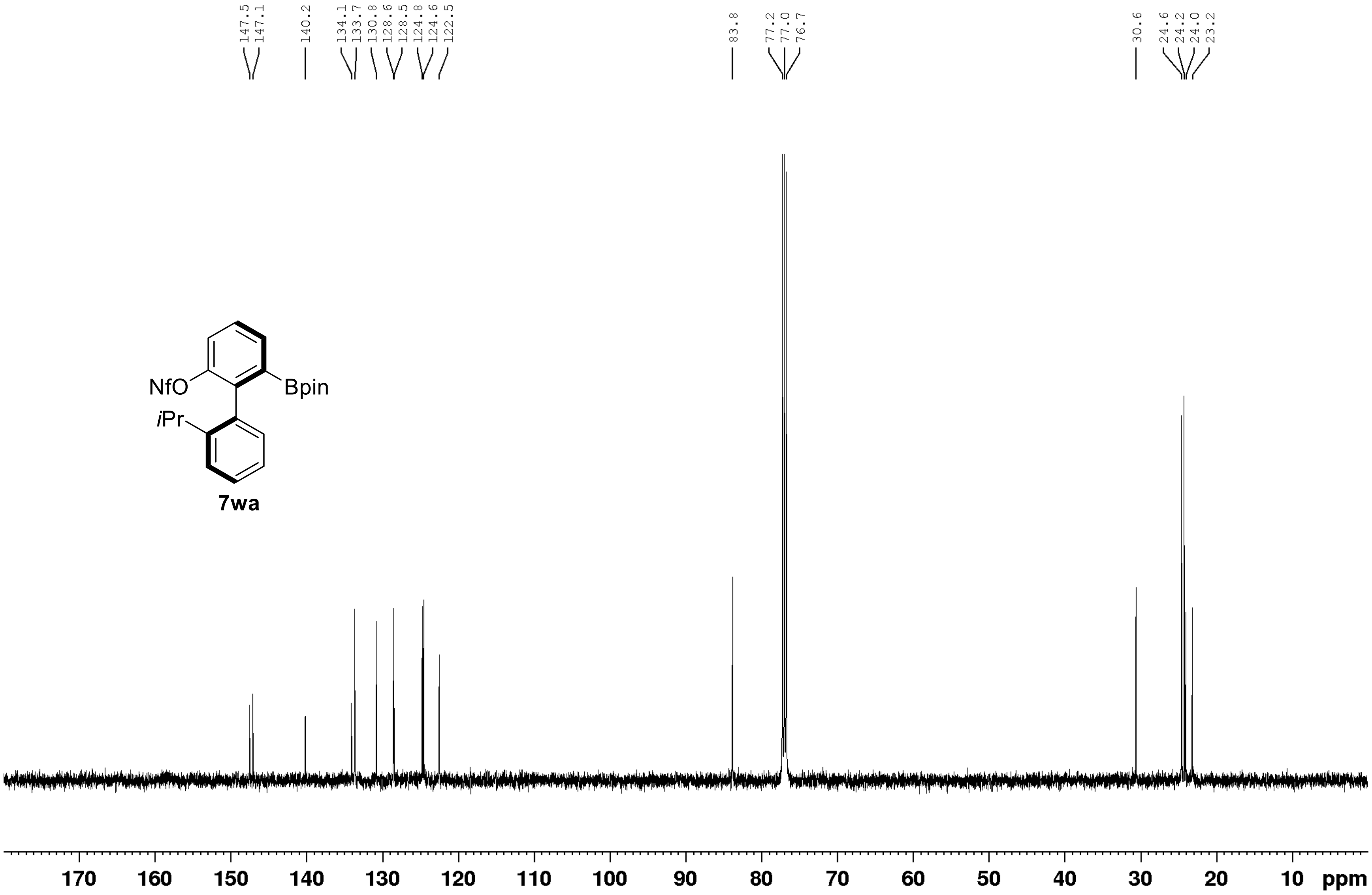

Figure S229. **^{19}F NMR** (471 MHz, $CDCl_3$, 298 K) of **7wa**

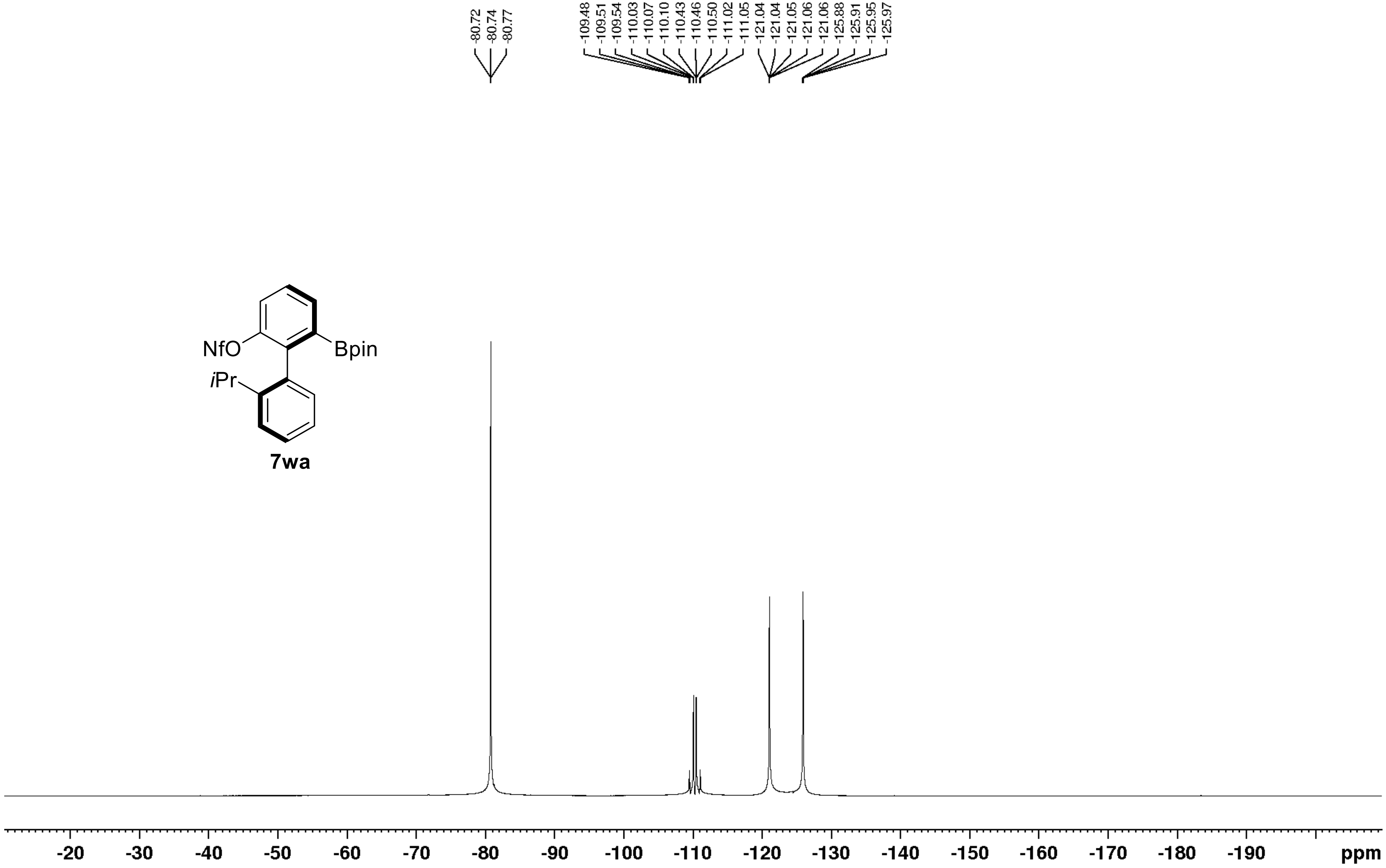

Figure S230. ^{11}**B NMR** (160 MHz, $CDCl_3$, 298 K) of **7wa**

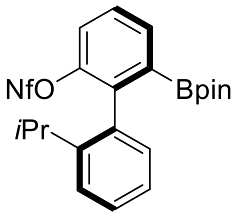

7wa

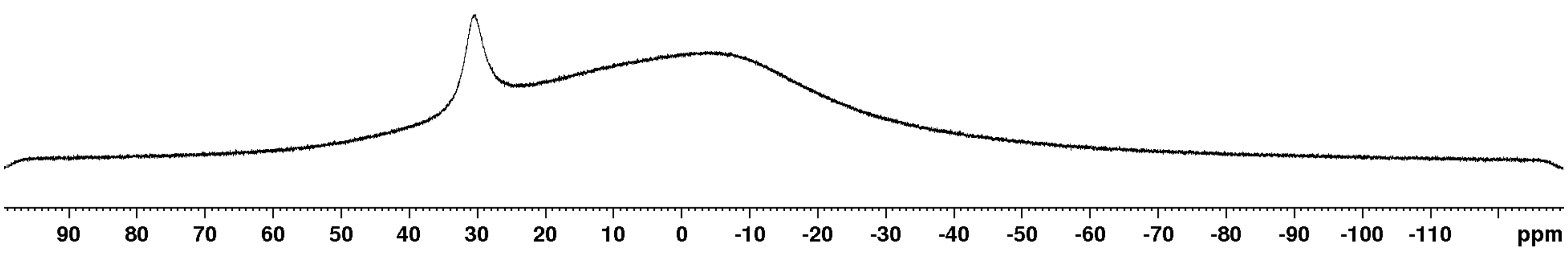

(*R*)-2'-methyl-6-(4,4,5,5-tetramethyl-1,3,2-dioxaborolan-2-yl)-[1,1'-biphenyl]-2-yl 1,1,2,2,3,3,4,4,4-nonafluorobutane-1-sulfonate (**7xa**)

Figure S231. ^{1}**H NMR** (500 MHz, $CDCl_3$, 298 K) of **7xa**

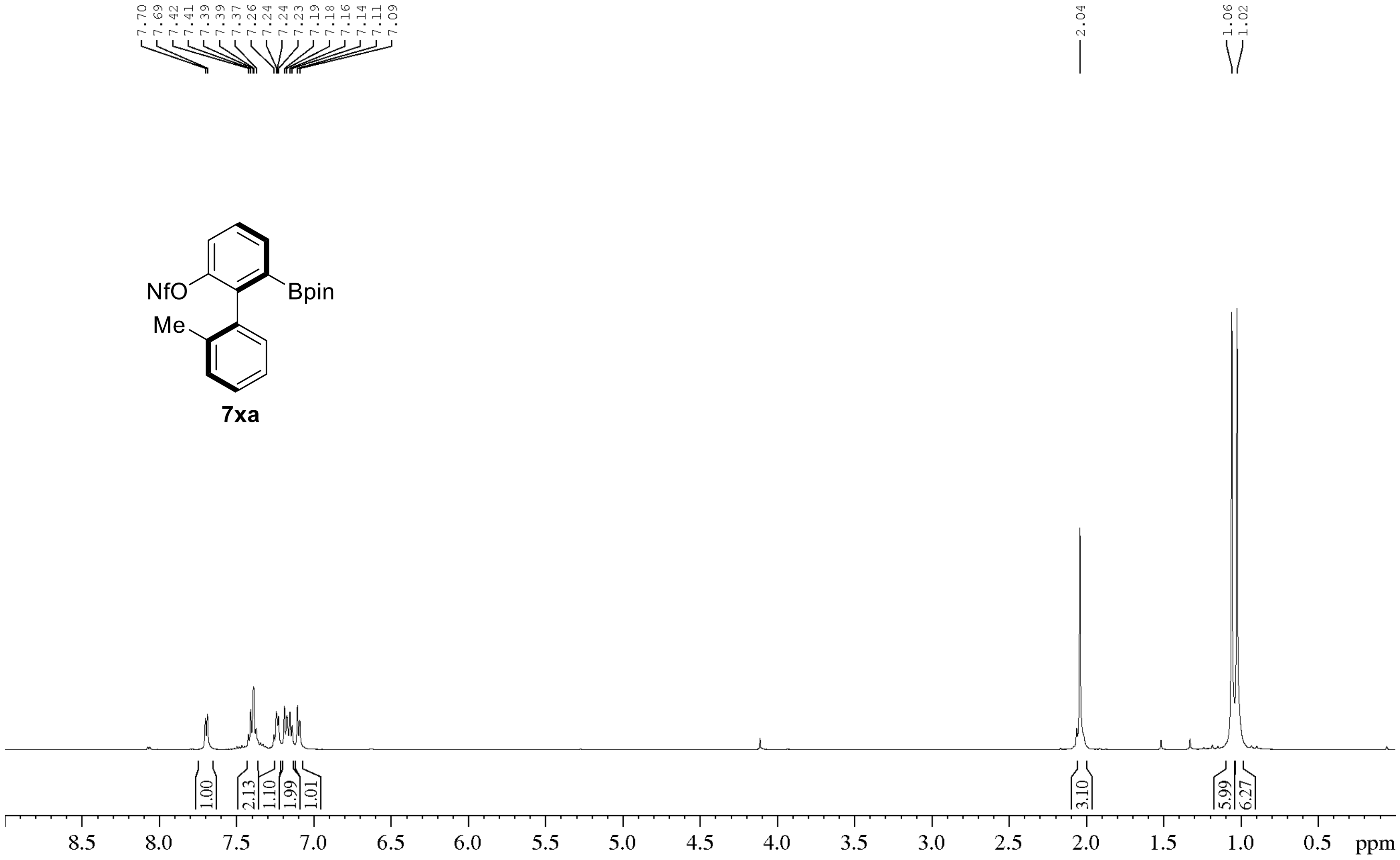

Figure S232. **^{13}C{^{1}H} NMR** (101 MHz, $CDCl_3$, 298 K) of **7xa**

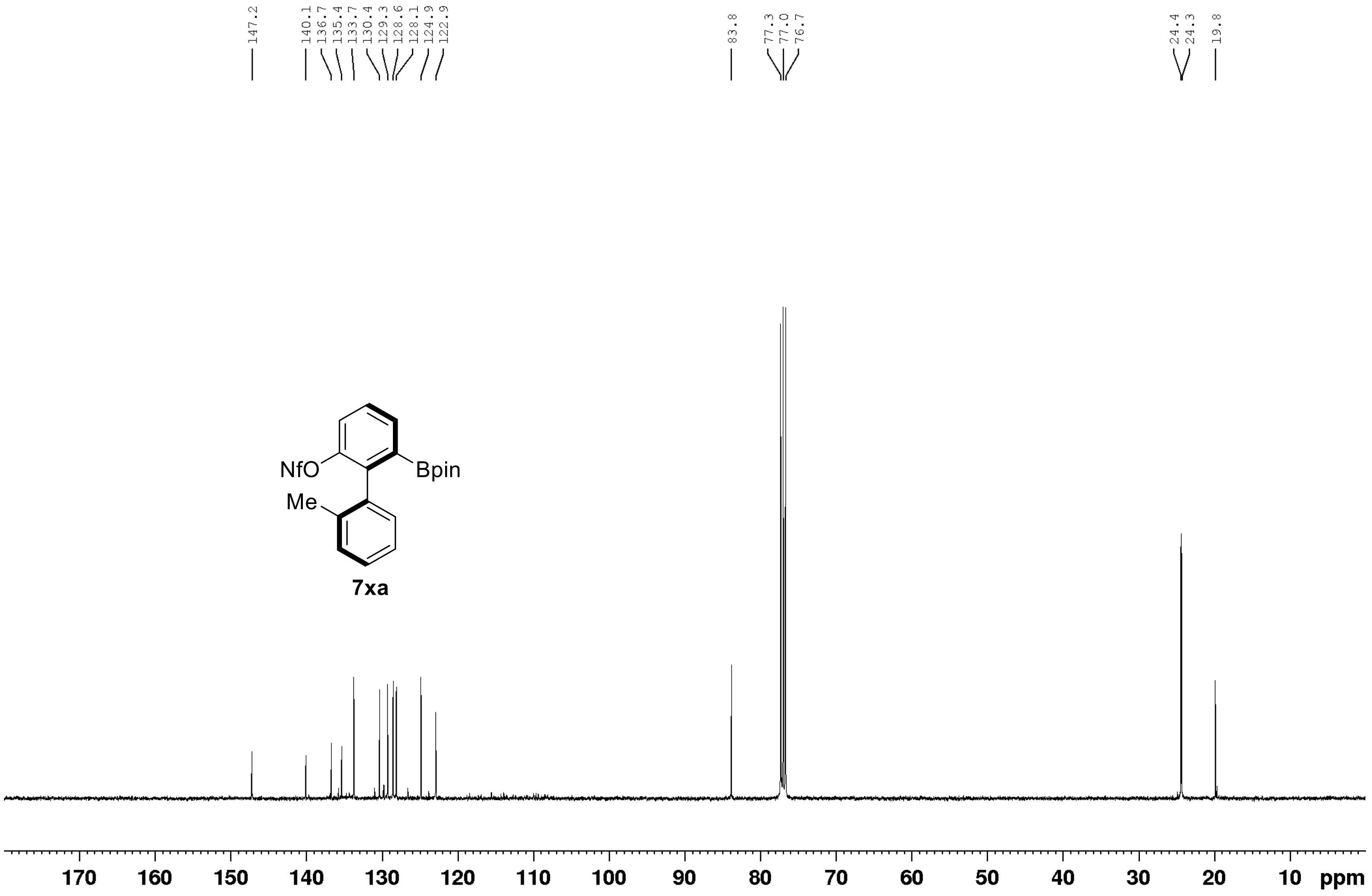

Figure S233. ^{19}F NMR (471 MHz, $CDCl_3$, 298 K) of **7xa**

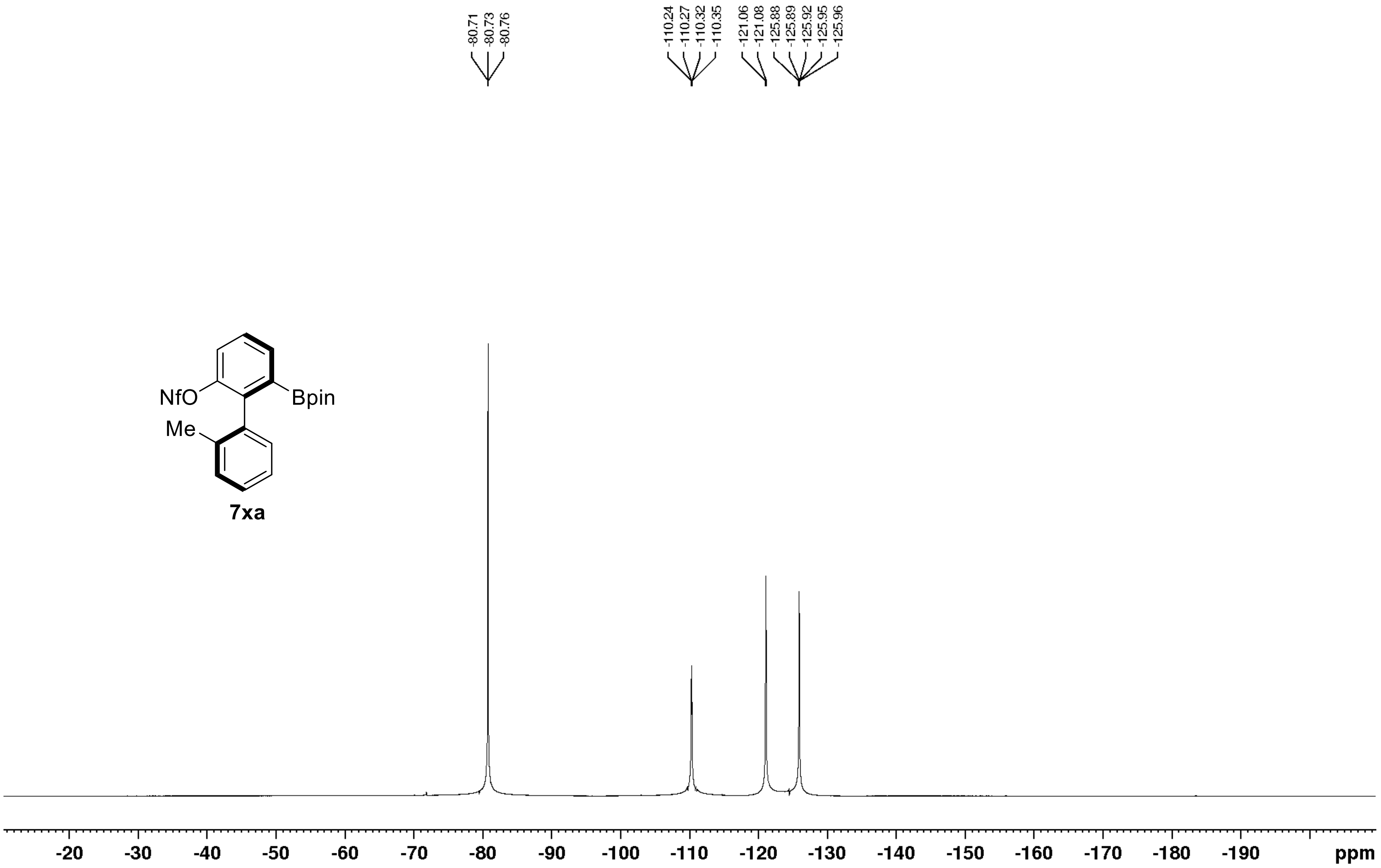

Figure S234. ^{11}**B NMR** (160 MHz, $CDCl_3$, 298 K) of **7xa**

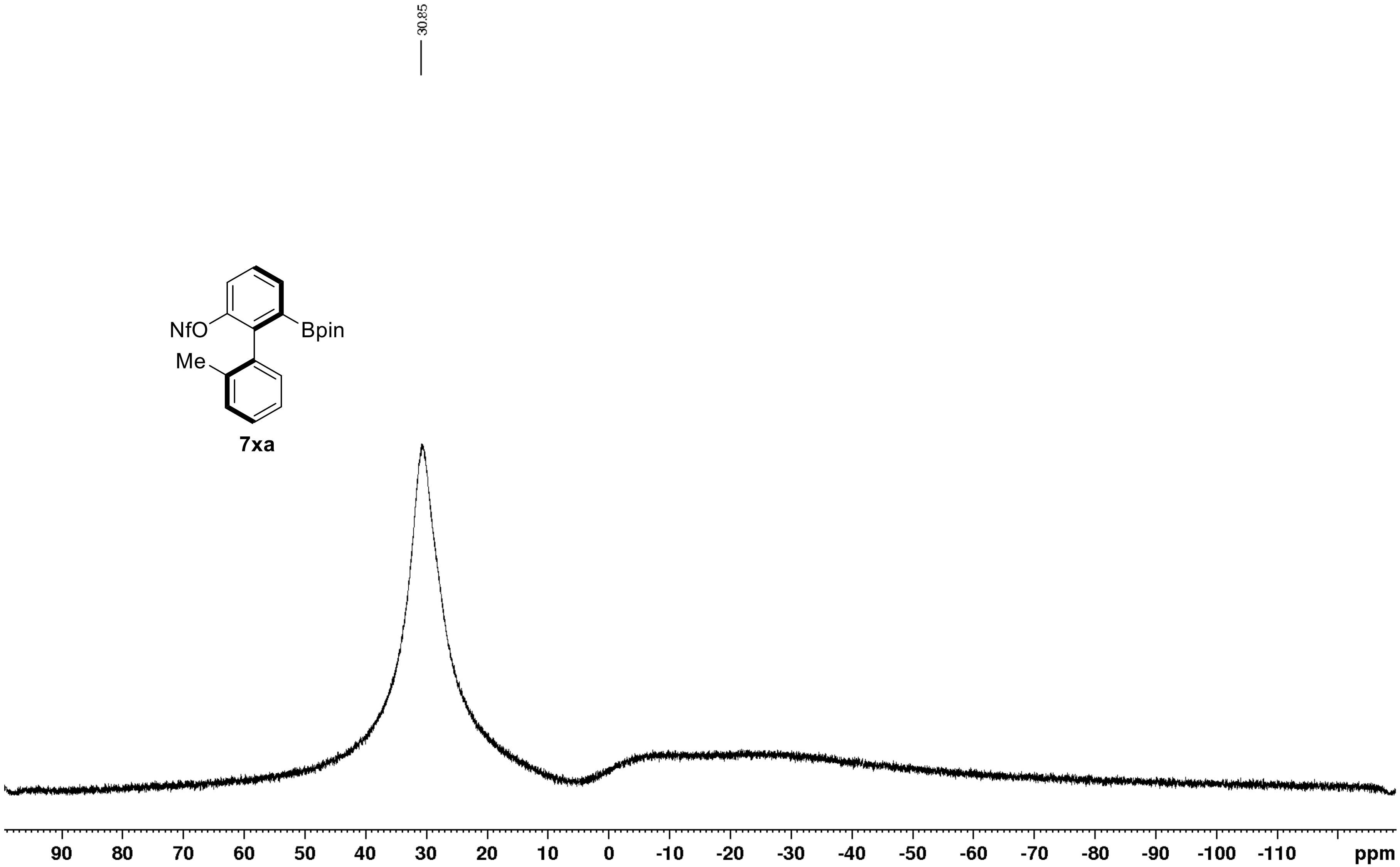

(*R*)-2'-chloro-6-(4,4,5,5-tetramethyl-1,3,2-dioxaborolan-2-yl)-[1,1'-biphenyl]-2-yl 1,1,2,2,3,3,4,4,4-nonafluorobutane-1-sulfonate (**7ya**)

Figure S235. **^{1}H NMR** (400 MHz, $CDCl_3$, 298 K) of **7ya**

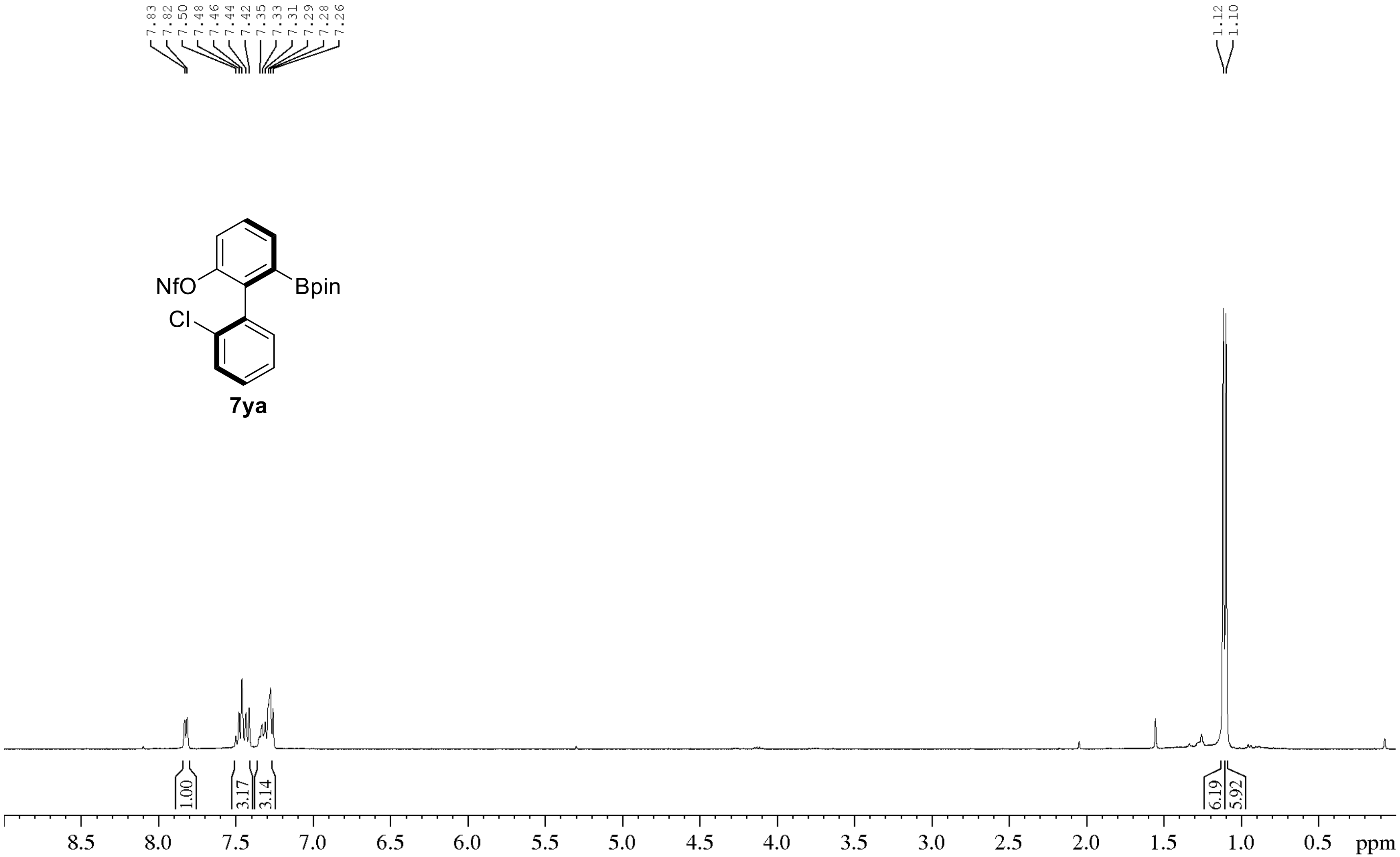

Figure S236. **$^{13}C\{^{1}H\}$ NMR** (101 MHz, $CDCl_3$, 298 K) of **7ya**

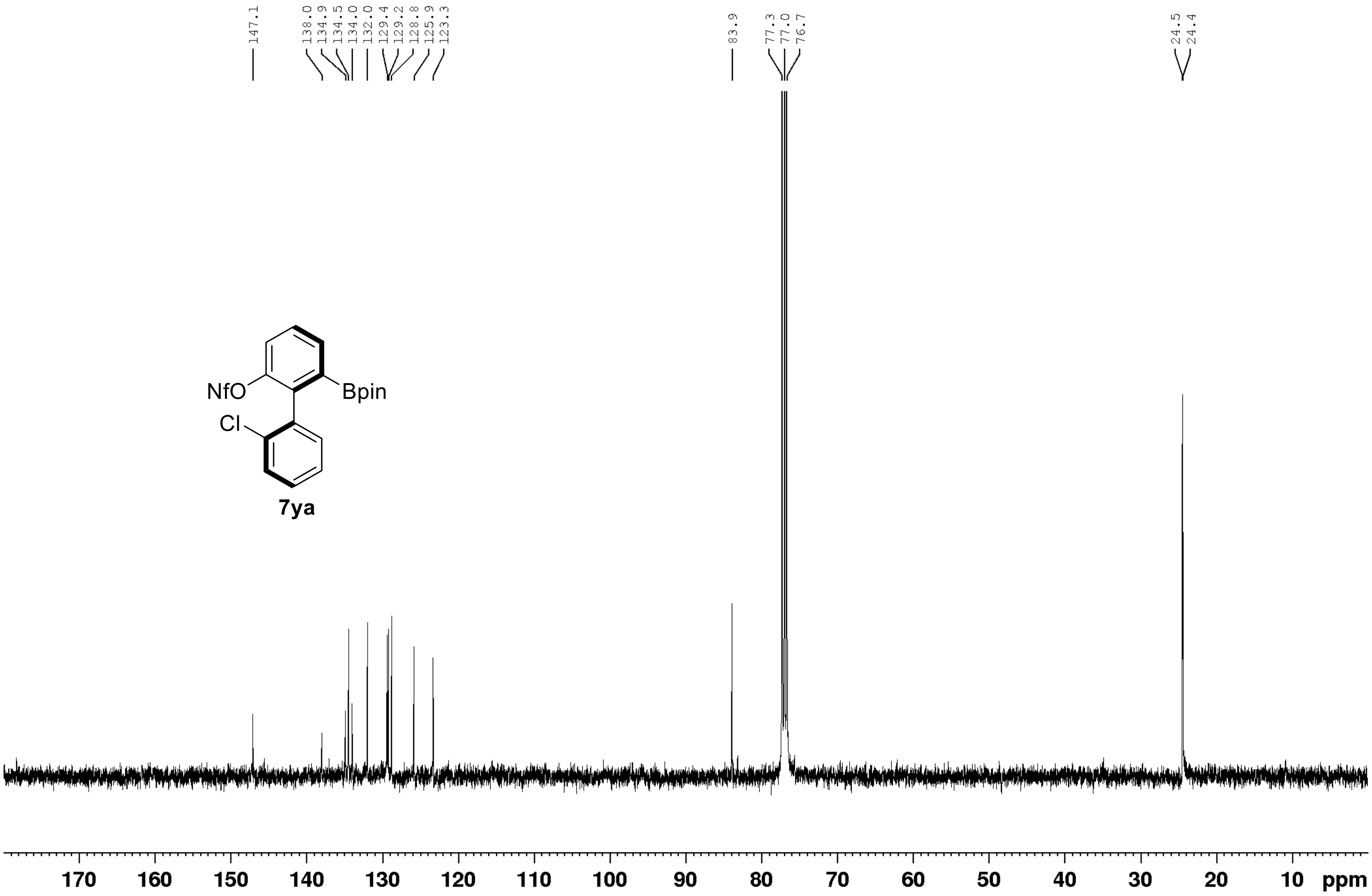

Figure S237. ^{19}**F NMR** (471 MHz, $CDCl_3$, 298 K) of **7ya**

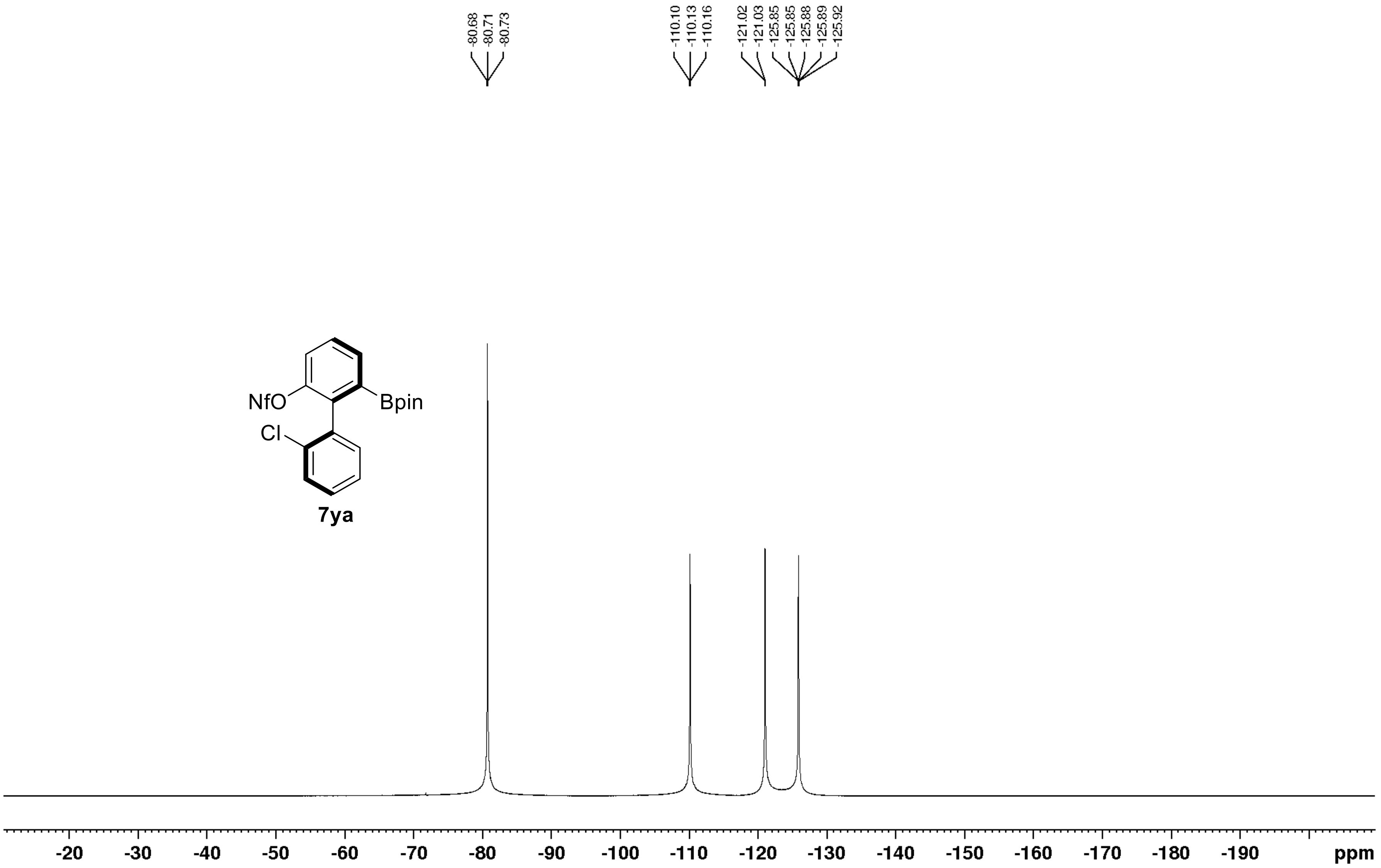

Figure S238. ^{11}**B NMR** (160 MHz, $CDCl_3$, 298 K) of **7ya**

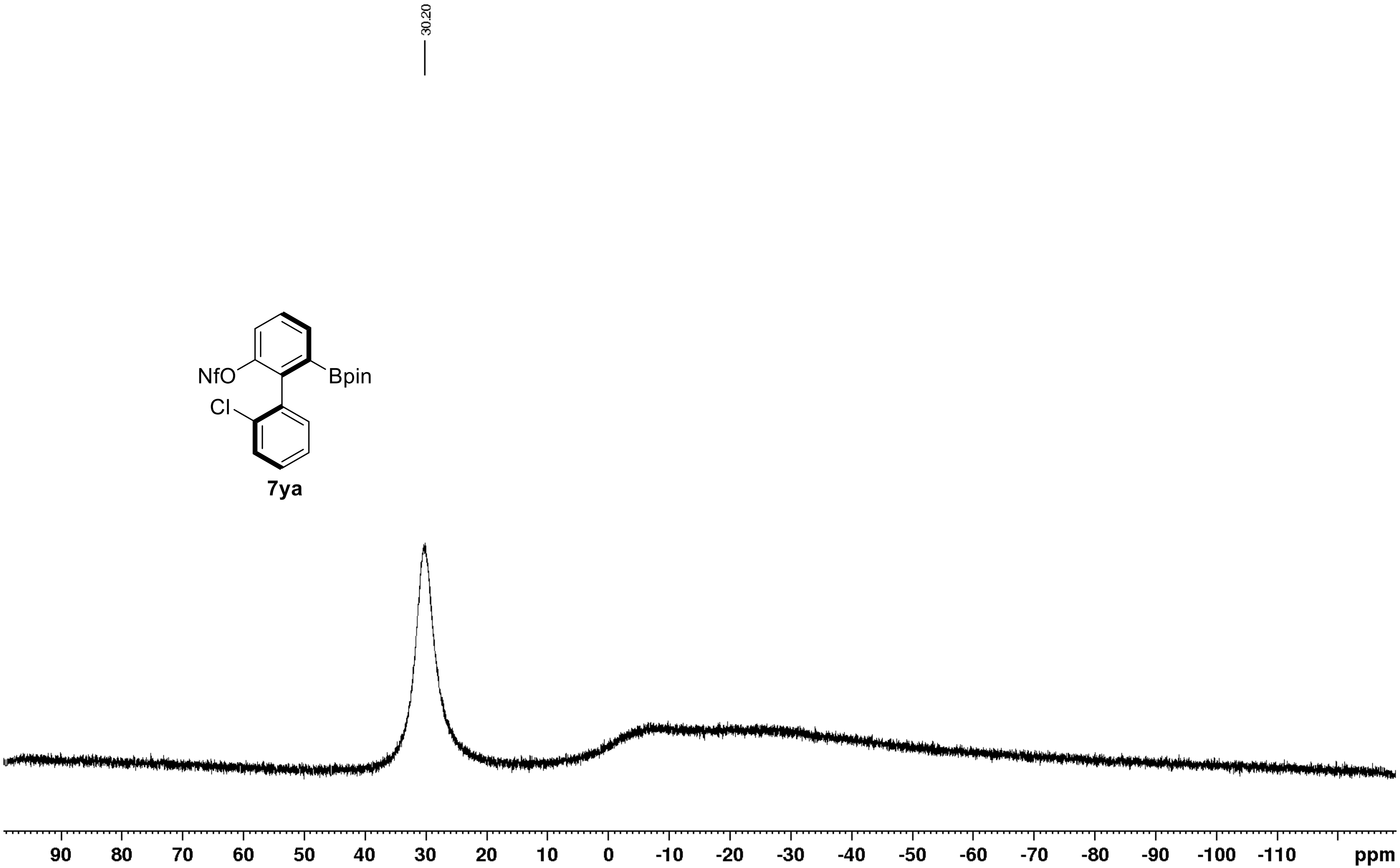

(*R*)-6-(5,5-dimethyl-1,3,2-dioxaborinan-2-yl)-[1,1':2',1''-terphenyl]-2-yl 1,1,2,2,3,3,4,4,4-nonafluorobutane-1-sulfonate (7lb)

Figure S239. **^{1}H NMR** (500 MHz, $CDCl_3$, 298 K) of **7lb**

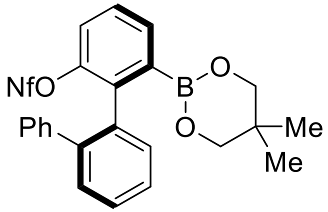

7lb

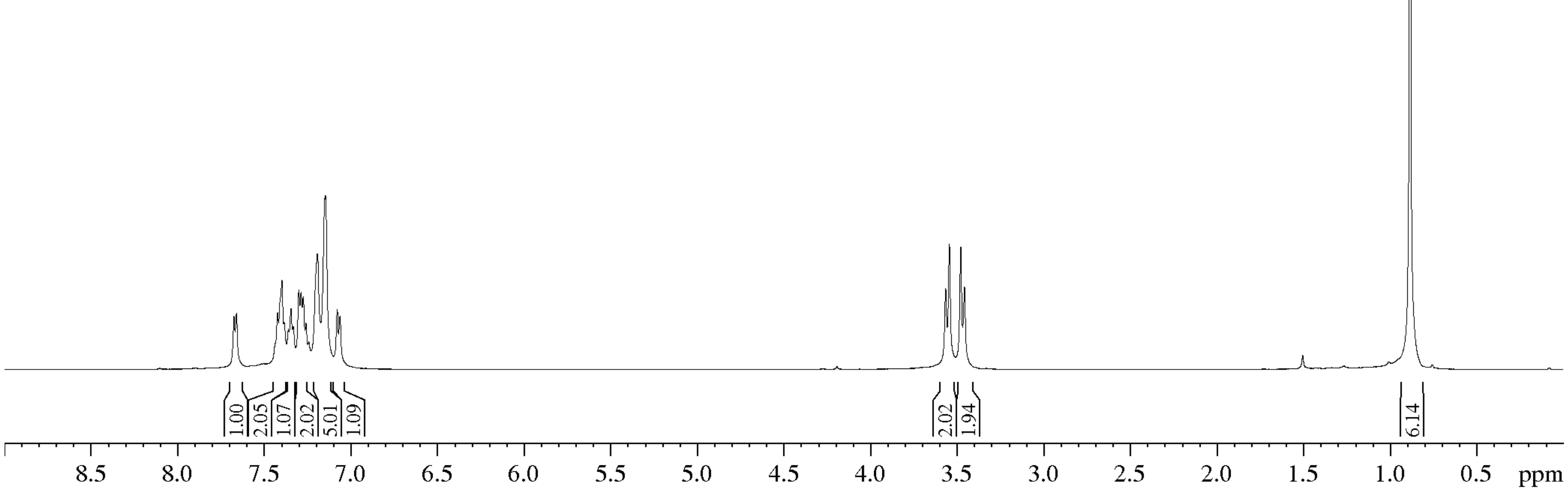

Figure S240. $^{13}C\{^{1}H\}$ NMR (101 MHz, $CDCl_3$, 298 K) of **7lb**

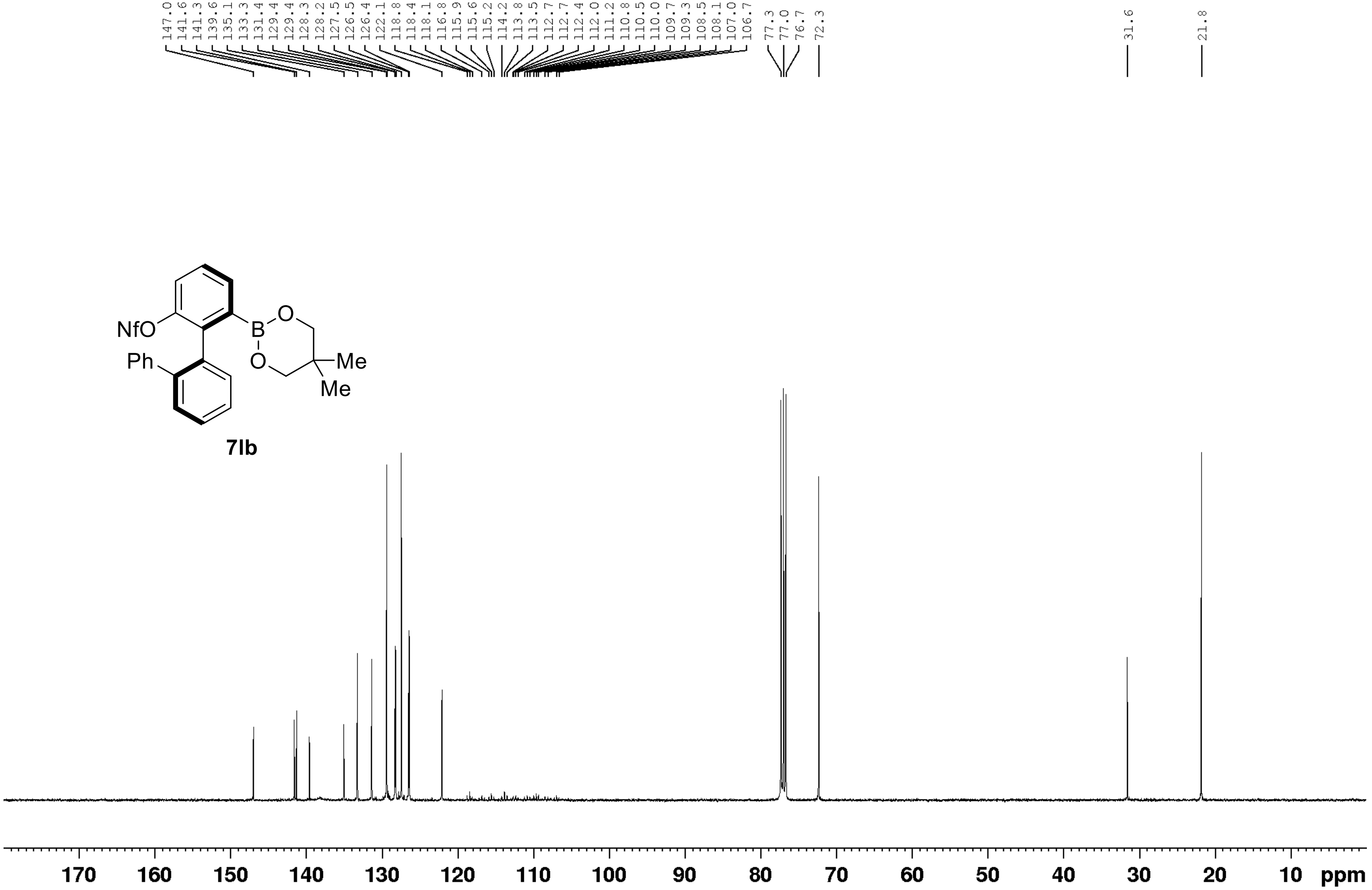

Figure S241. ^{19}F NMR (471 MHz, $CDCl_3$, 298 K) of **7lb**

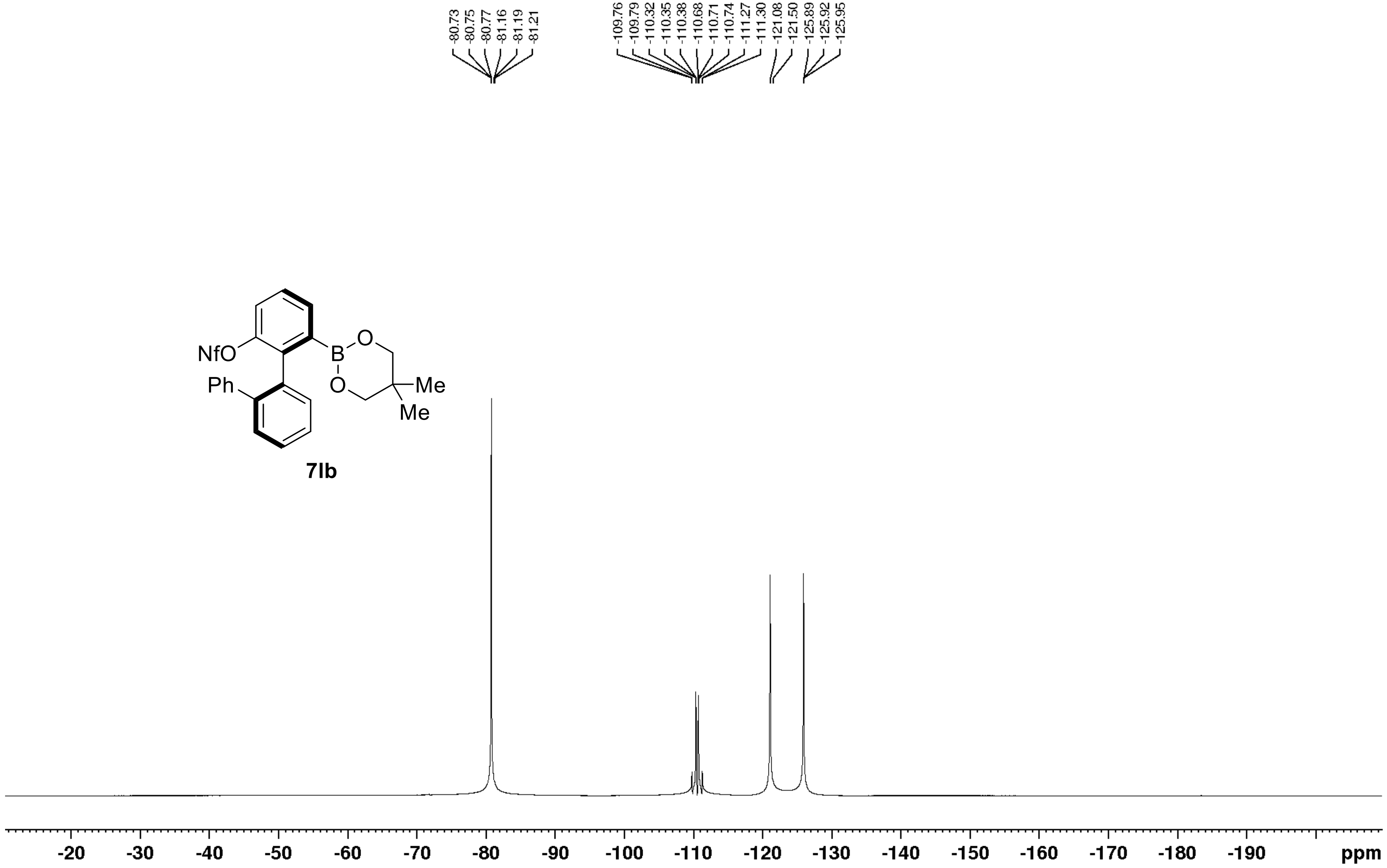

Figure S242. ^{11}**B NMR** (160 MHz, $CDCl_3$, 298 K) of **7lb**

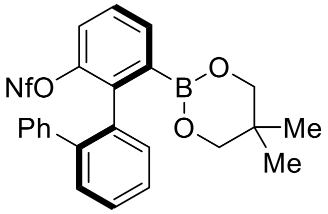

7lb

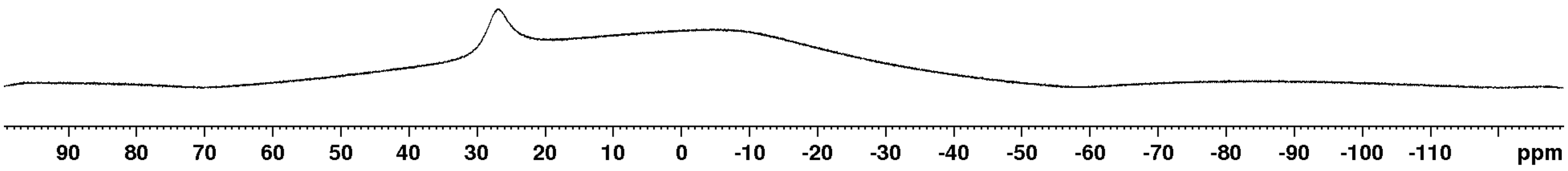

(*R*)-6-(4,4,6,6-tetramethyl-1,3,2-dioxaborinan-2-yl)-[1,1':2',1''-terphenyl]-2-yl 1,1,2,2,3,3,4,4,4-nonafluorobutane-1-sulfonate (7lc)

Figure S243. ¹H NMR (500 MHz, $CDCl_3$, 298 K) of **7lc**

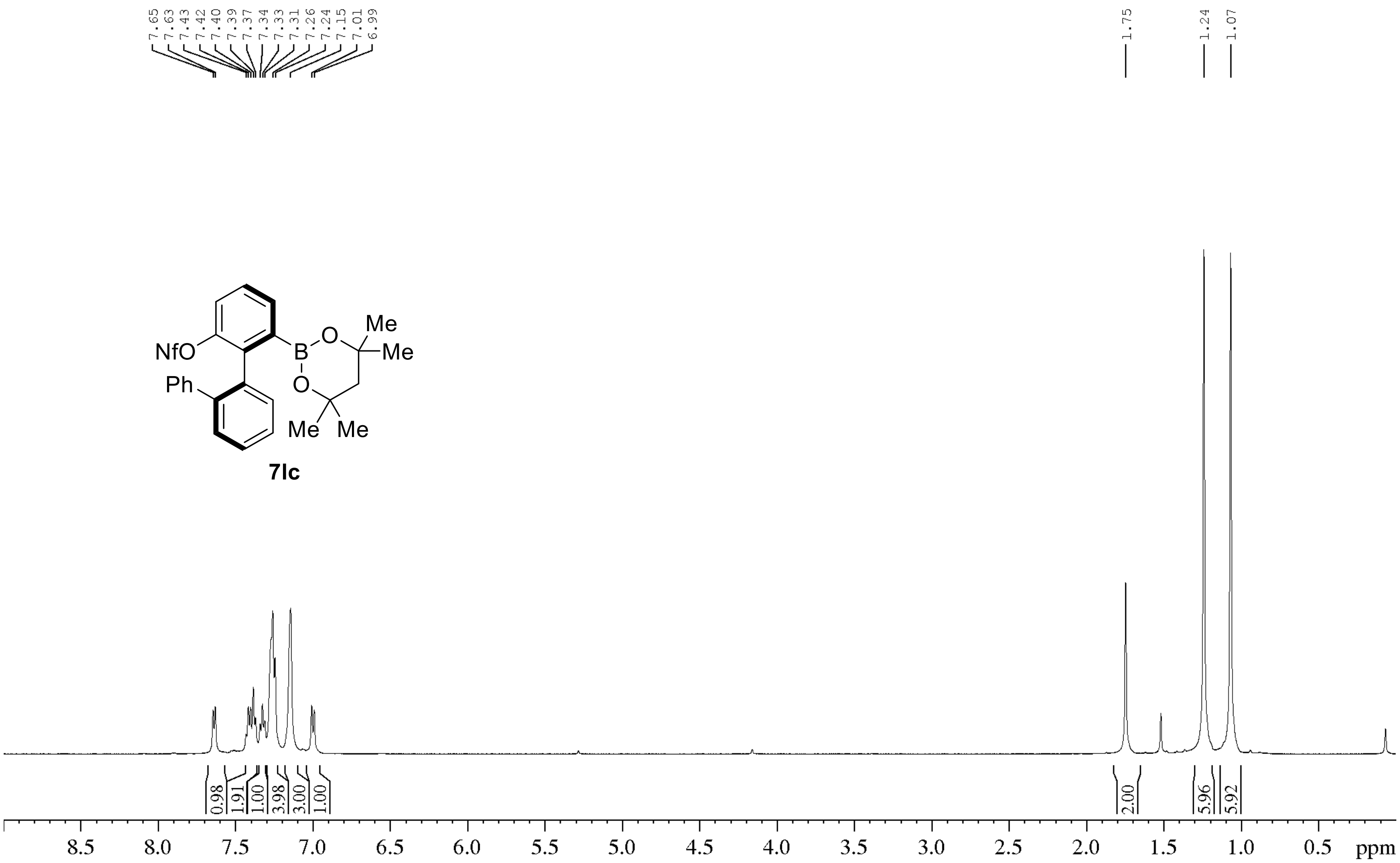

7lc

Figure S244. **$^{13}C\{^{1}H\}$ NMR** (101 MHz, $CDCl_3$, 298 K) of **7lc**

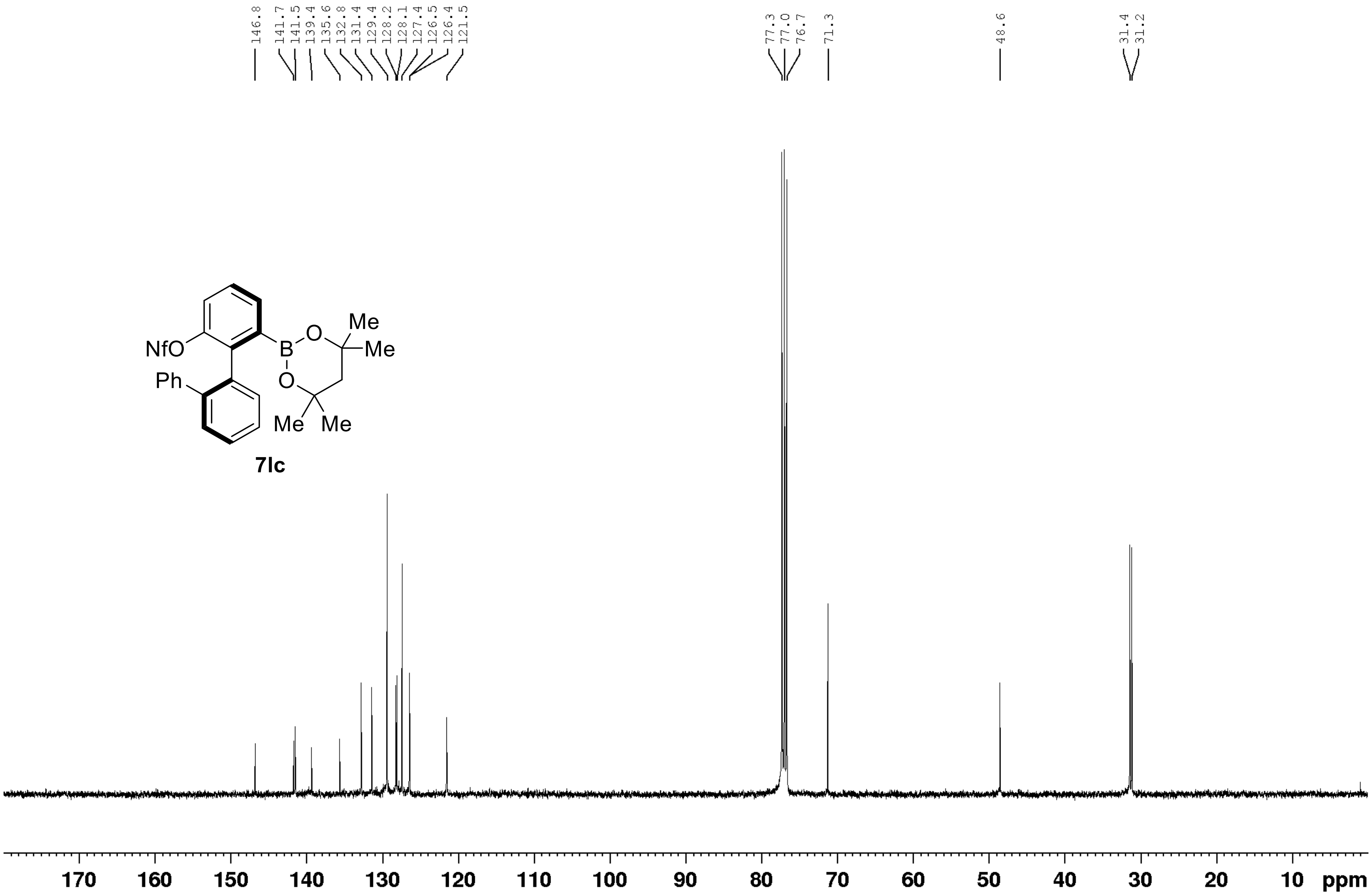

Figure S245. ^{19}F NMR (471 MHz, $CDCl_3$, 298 K) of **7lc**

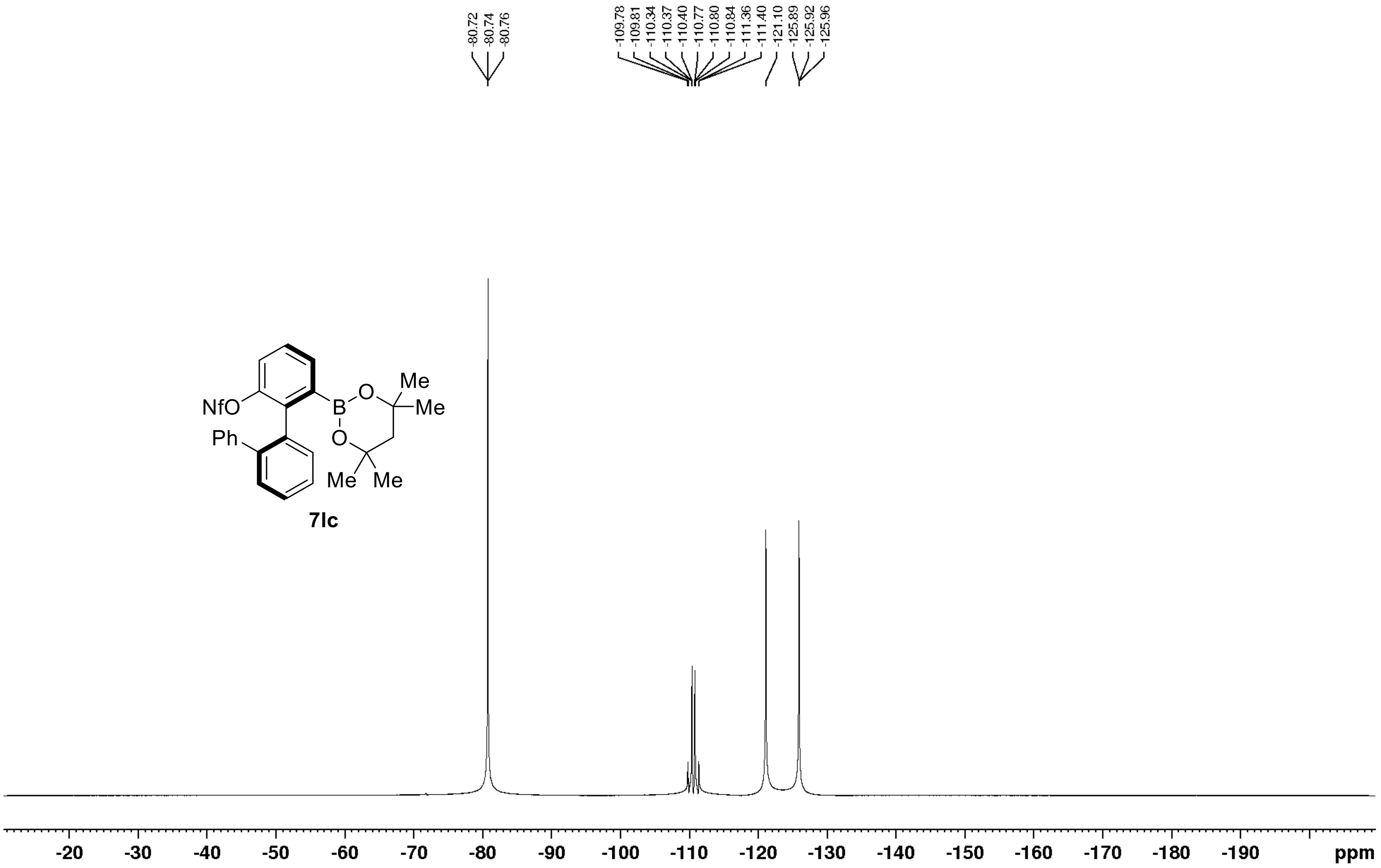

Figure S246. ^{11}B NMR (160 MHz, $CDCl_3$, 298 K) of **7lc**

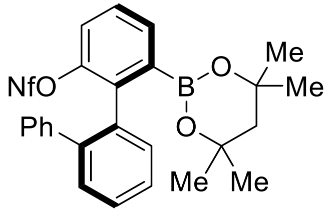

7lc

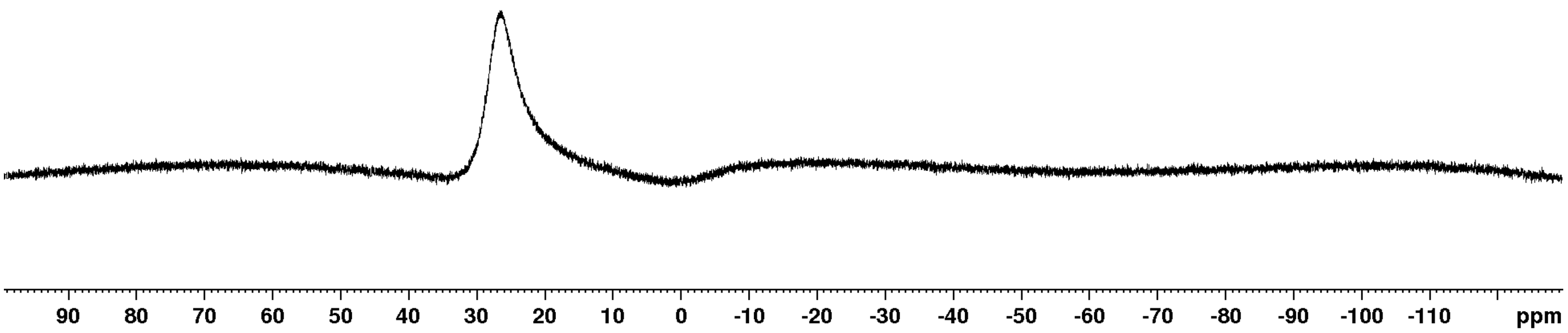

(*R*)-3-azido-2-(naphthalen-1-yl)phenyl 1,1,2,2,3,3,4,4,4-nonafluorobutane-1-sulfonate (**11**)

Figure S247. **^{1}H NMR** (500 MHz, $CDCl_3$, 298 K) of **11**

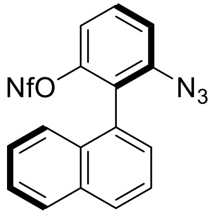

11

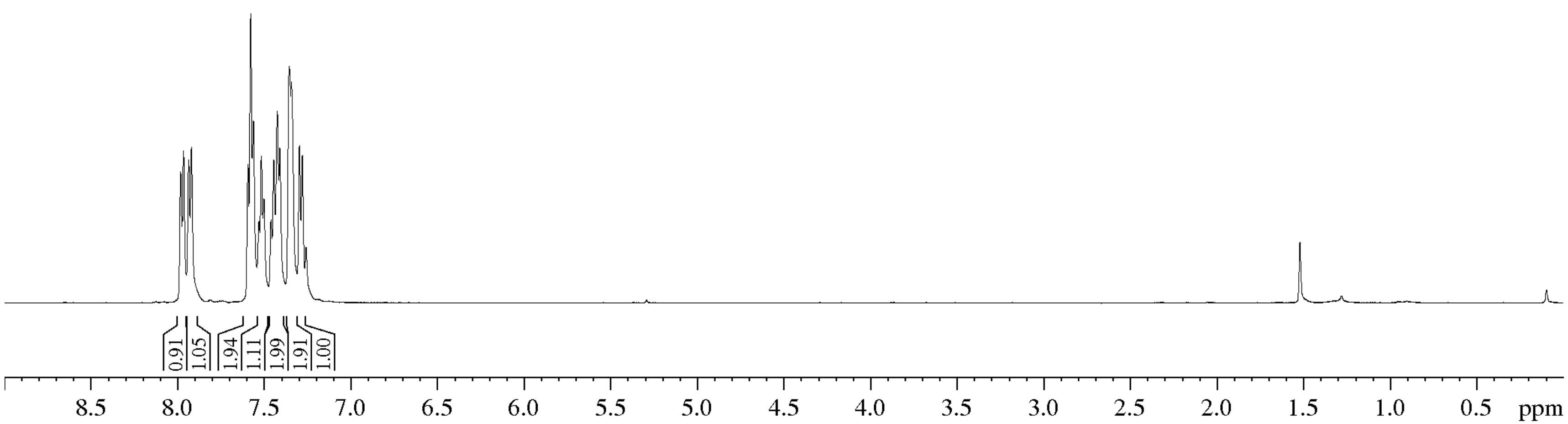

Figure S248. $^{13}C\{^{1}H\}$ **NMR** (126 MHz, $CDCl_3$, 298 K) of **11**

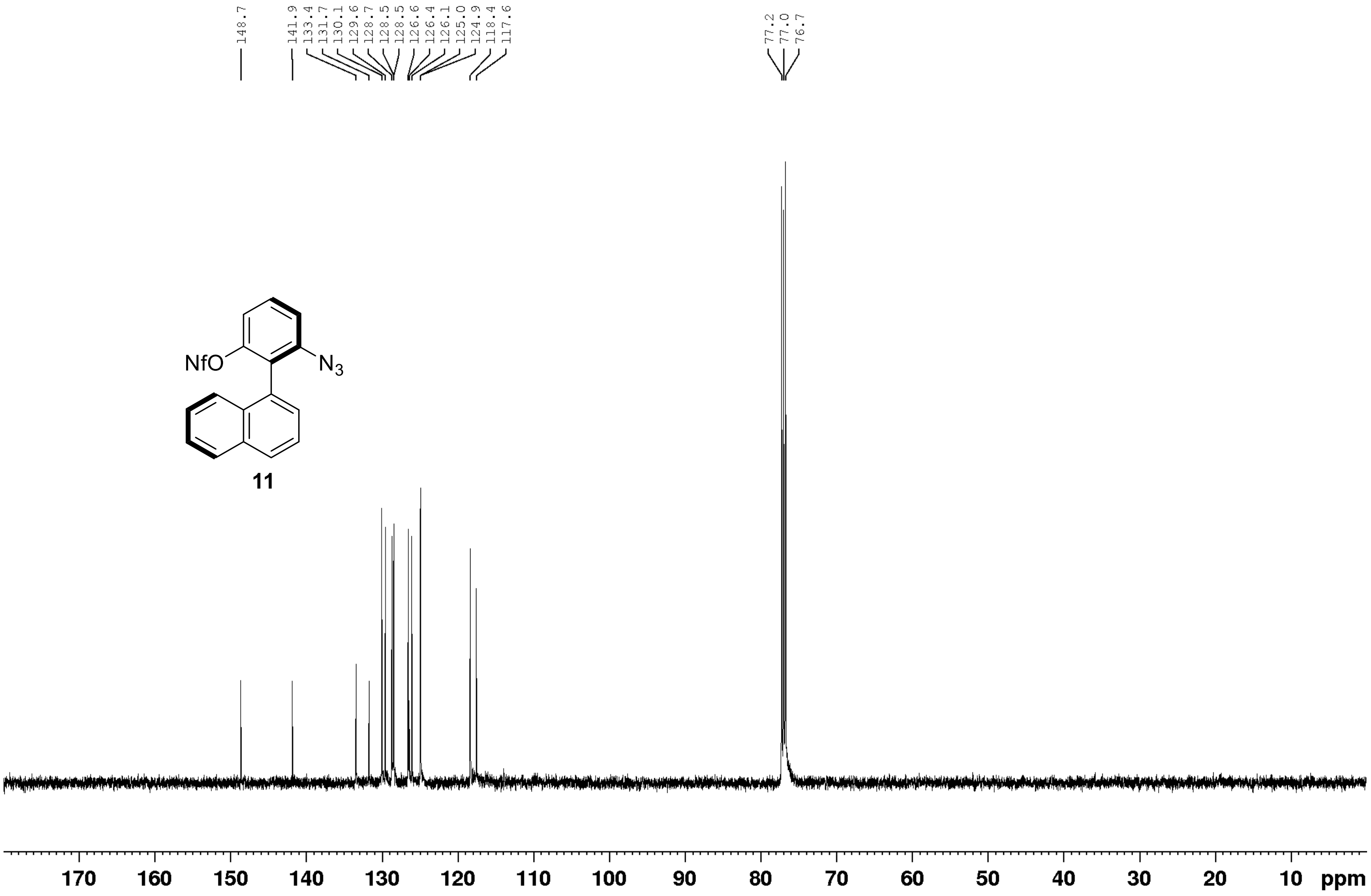

Figure S249. ^{19}**F NMR** (471 MHz, $CDCl_3$, 298 K) of **11**

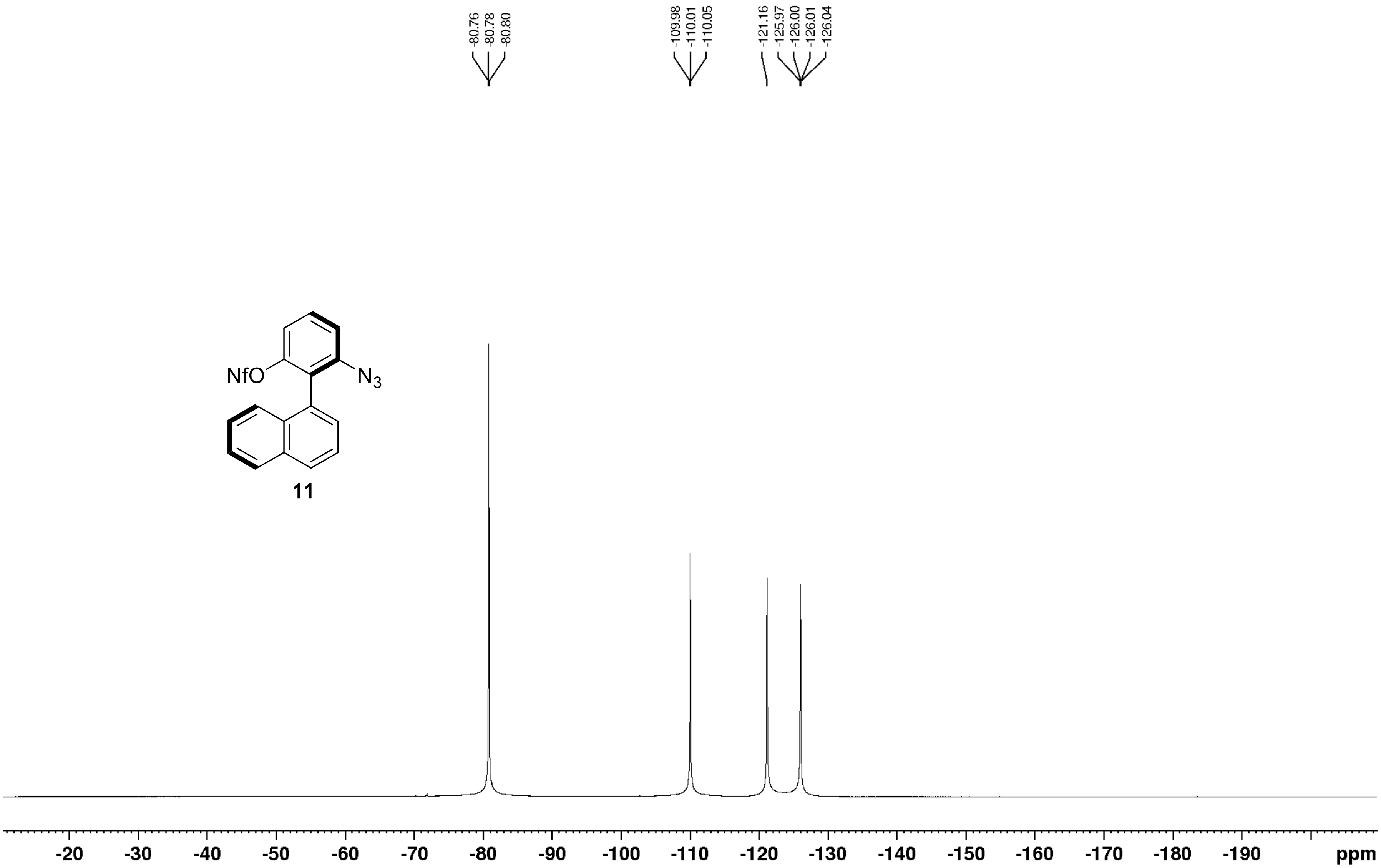

(*R*)-3-(1H-imidazol-1-yl)-2-(naphthalen-1-yl)phenyl 1,1,2,2,3,3,4,4,4-nonafluorobutane-1-sulfonate (**12**)

Figure S250. **^{1}H NMR** (500 MHz, $CDCl_3$, 298 K) of **12**

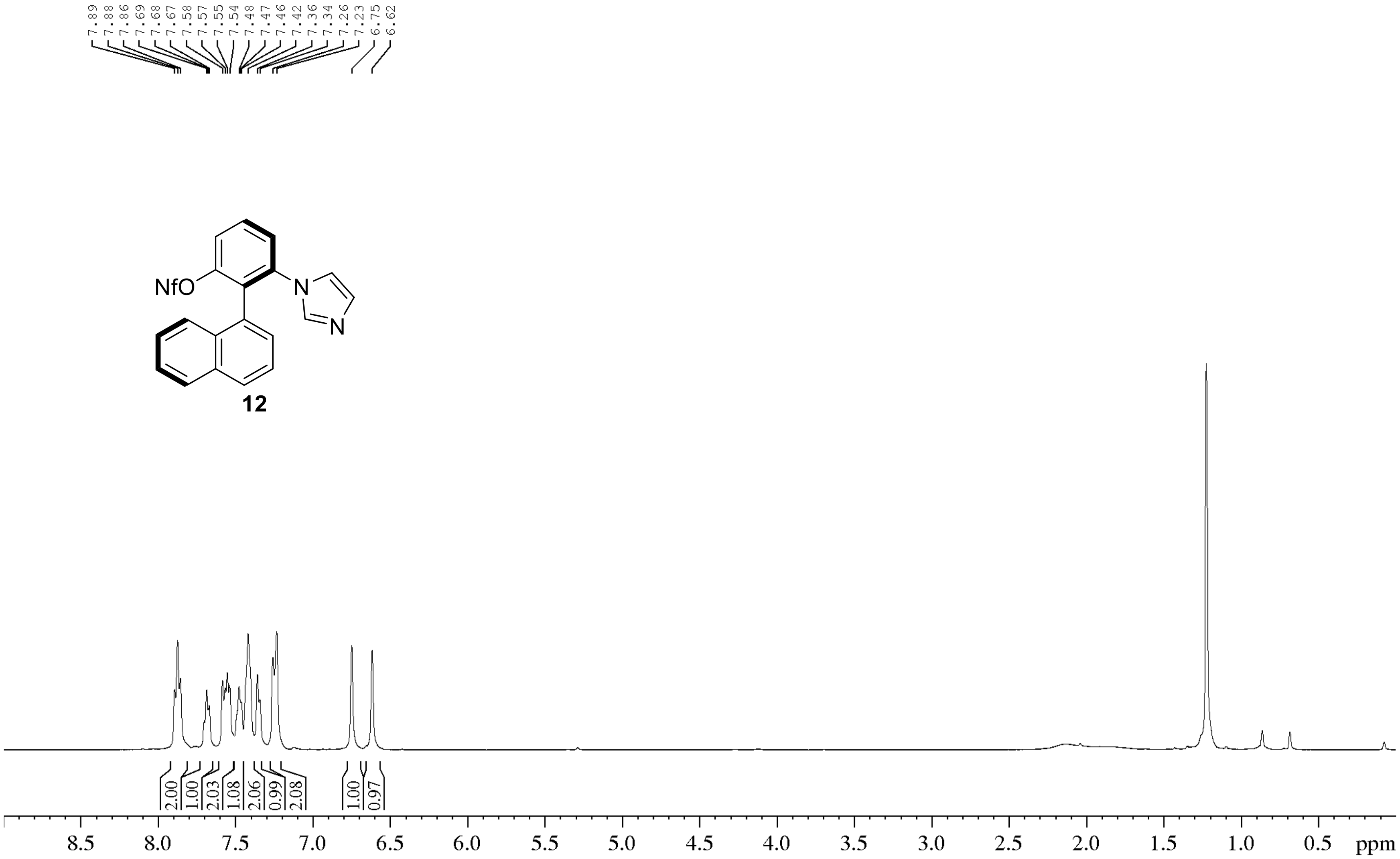

Figure S251. ^{13}**C{**^{1}**H} NMR** (126 MHz, $CDCl_3$, 298 K) of **12**

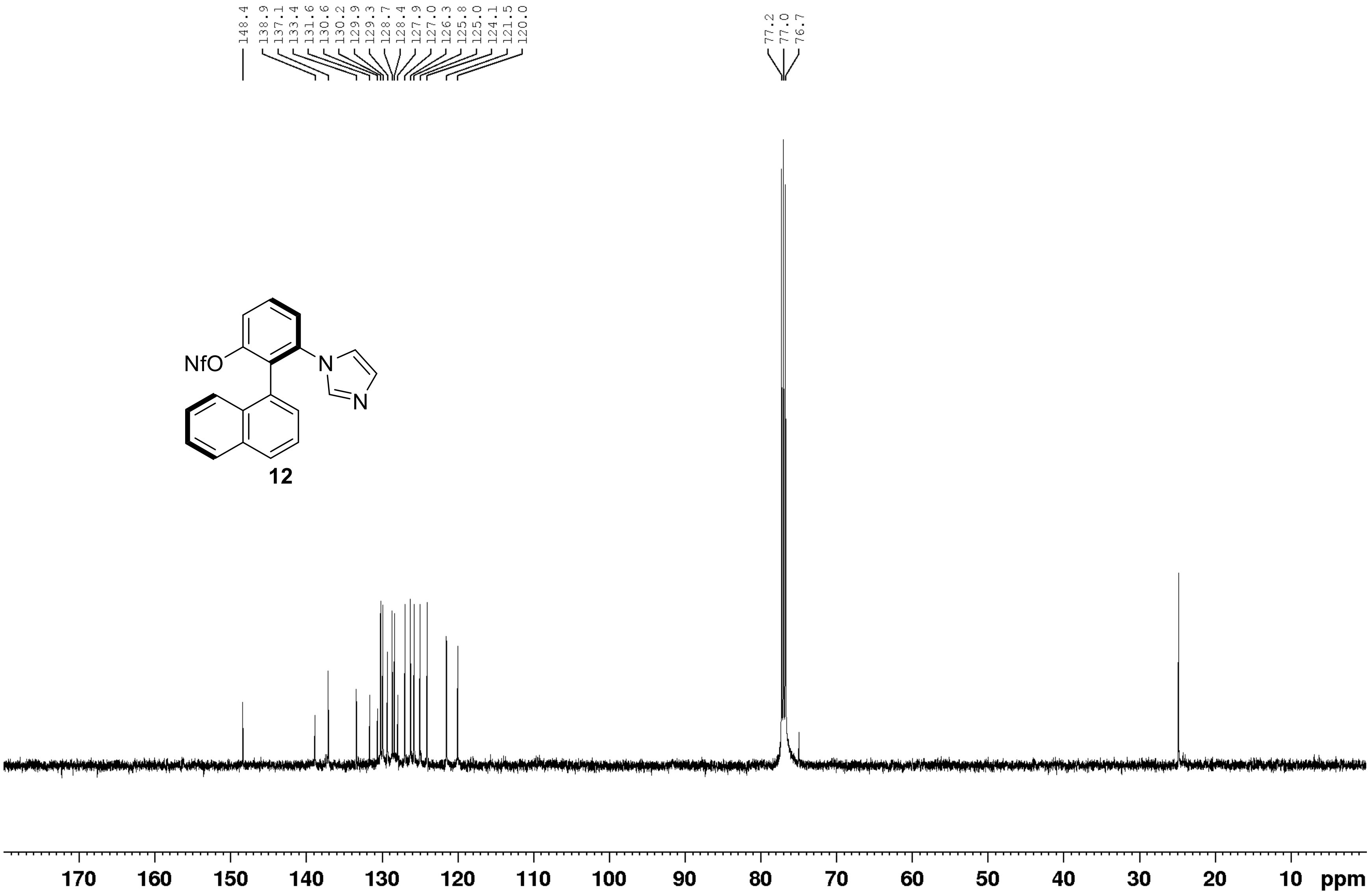

Figure S252. ^{19}F NMR (471 MHz, $CDCl_3$, 298 K) of **12**

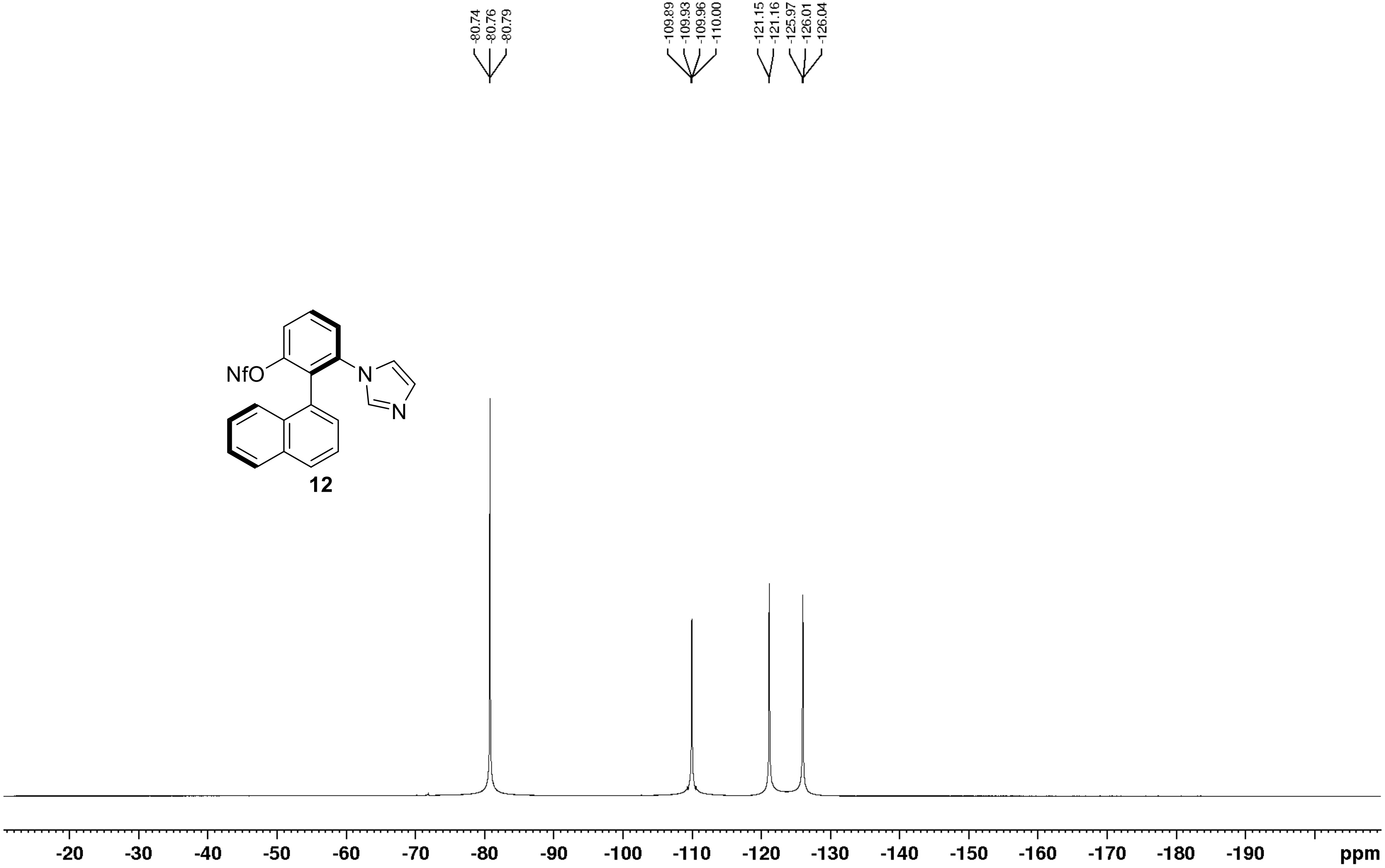

(*R*)-3-hydroxy-2-(naphthalen-1-yl)phenyl 1,1,2,2,3,3,4,4,4-nonafluorobutane-1-sulfonate (**13**)

Figure S253. ^{1}**H NMR** (500 MHz, $CDCl_3$, 298 K) of **13**

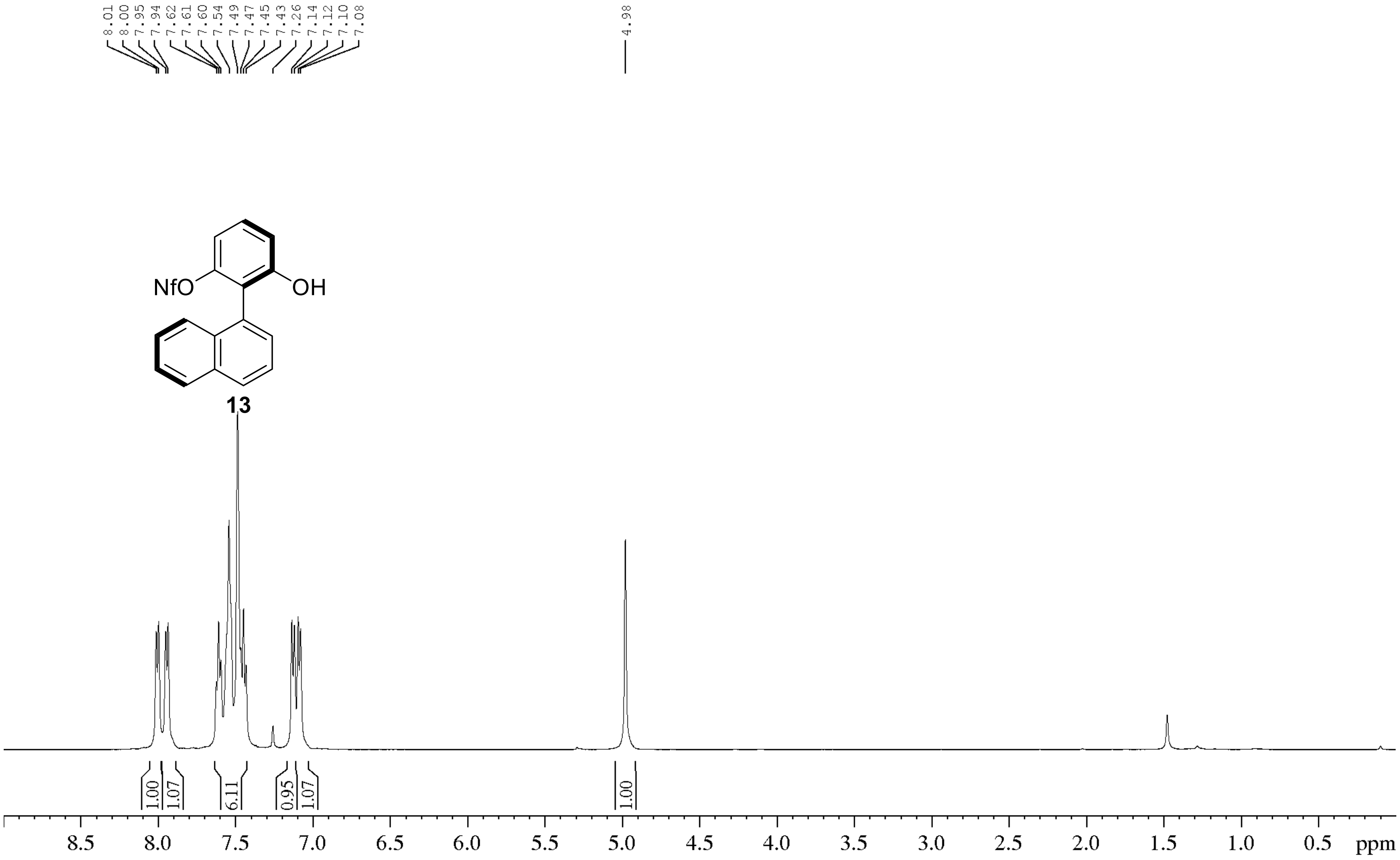

Figure S254. ^{13}**C{**^{1}**H} NMR** (126 MHz, $CDCl_3$, 298 K) of **13**

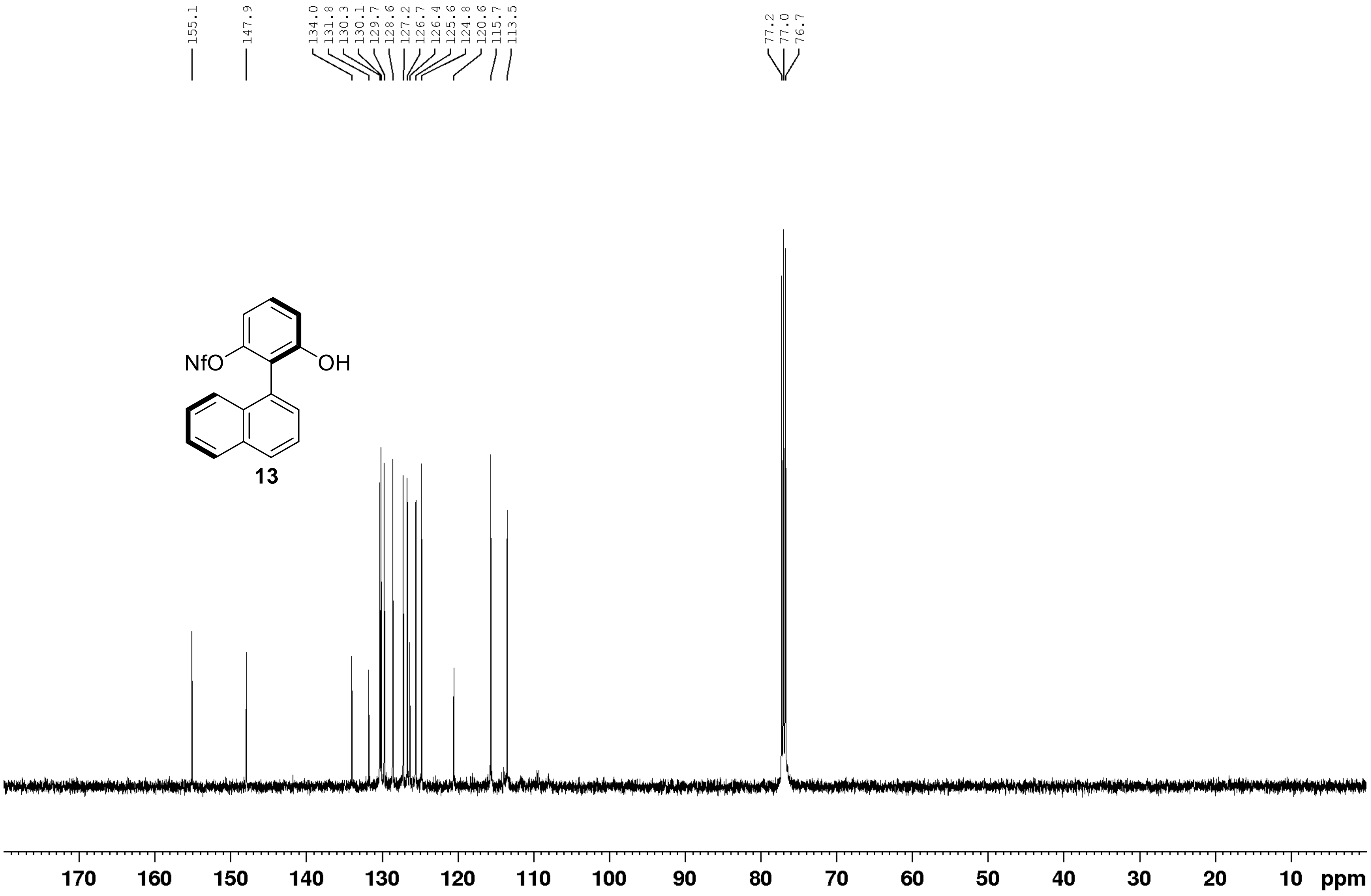

Figure S255. ^{19}F NMR (471 MHz, $CDCl_3$, 298 K) of **13**

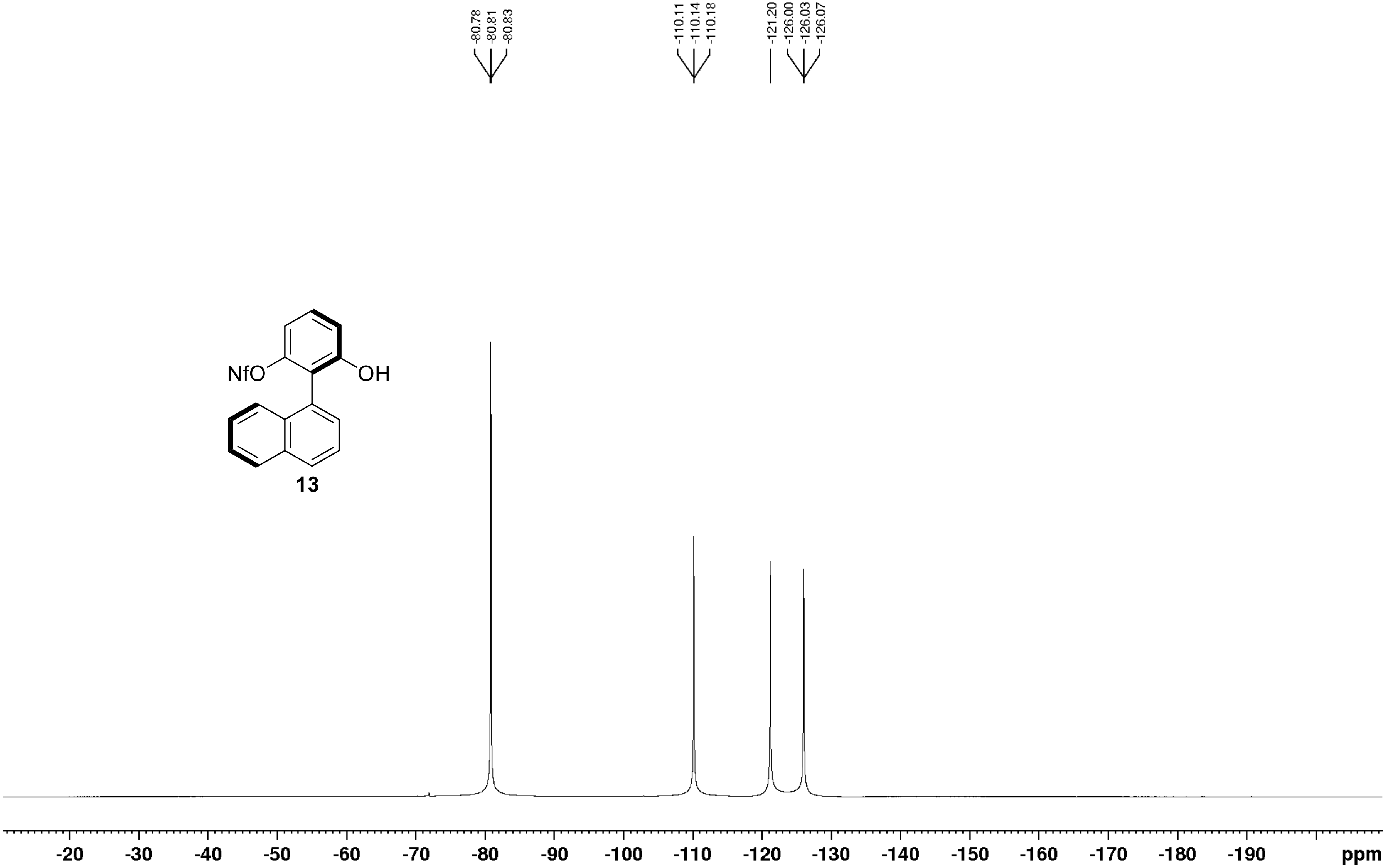

(*R*)-2-(naphthalen-1-yl)-[1,1'-biphenyl]-3-yl 1,1,2,2,3,3,4,4,4-nonafluorobutane-1-sulfonate (**14**)

Figure S256. **^{1}H NMR** (500 MHz, $CDCl_3$, 298 K) of **14**

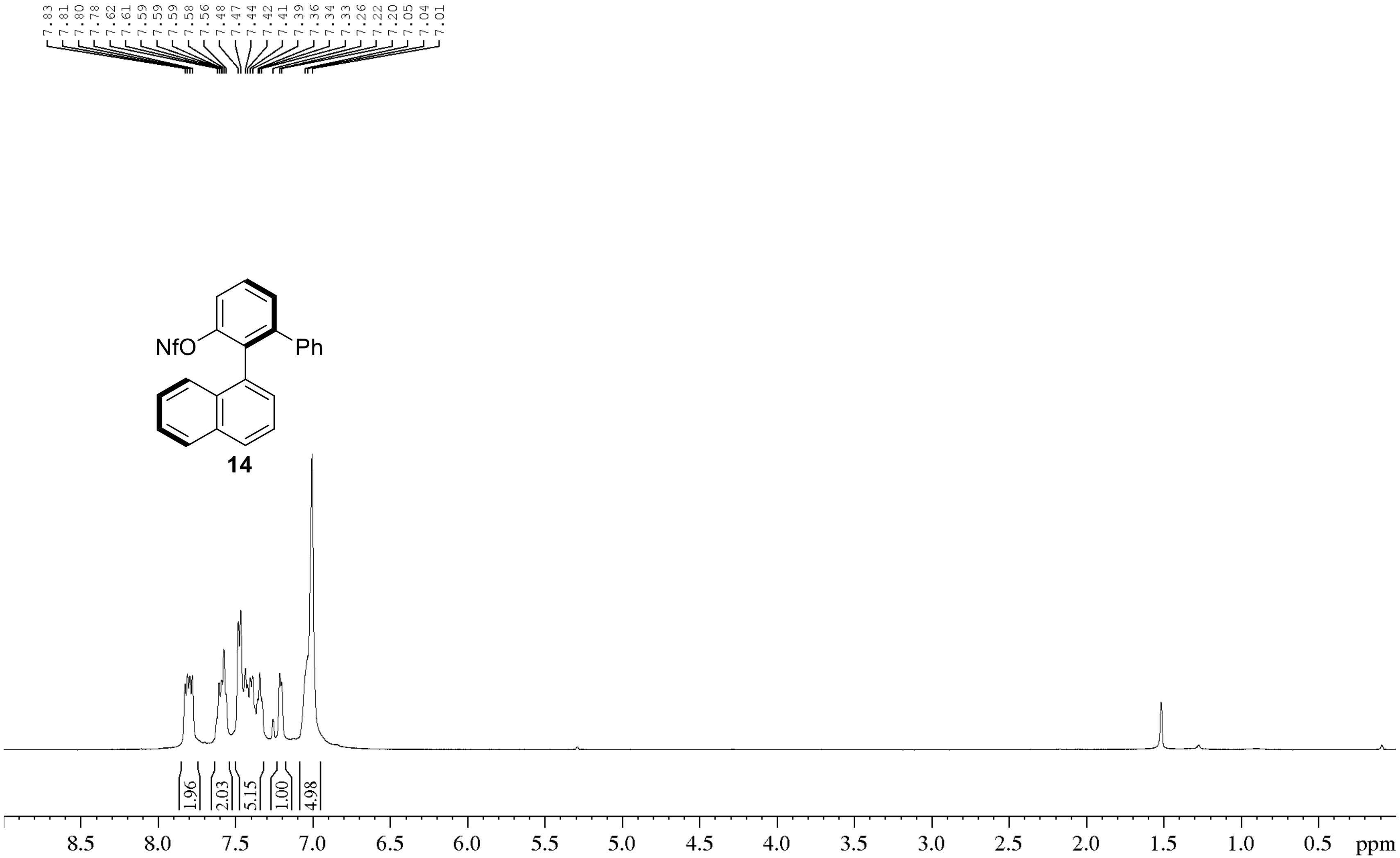

Figure S257. ^{13}C{^{1}H} NMR (126 MHz, $CDCl_3$, 298 K) of **14**

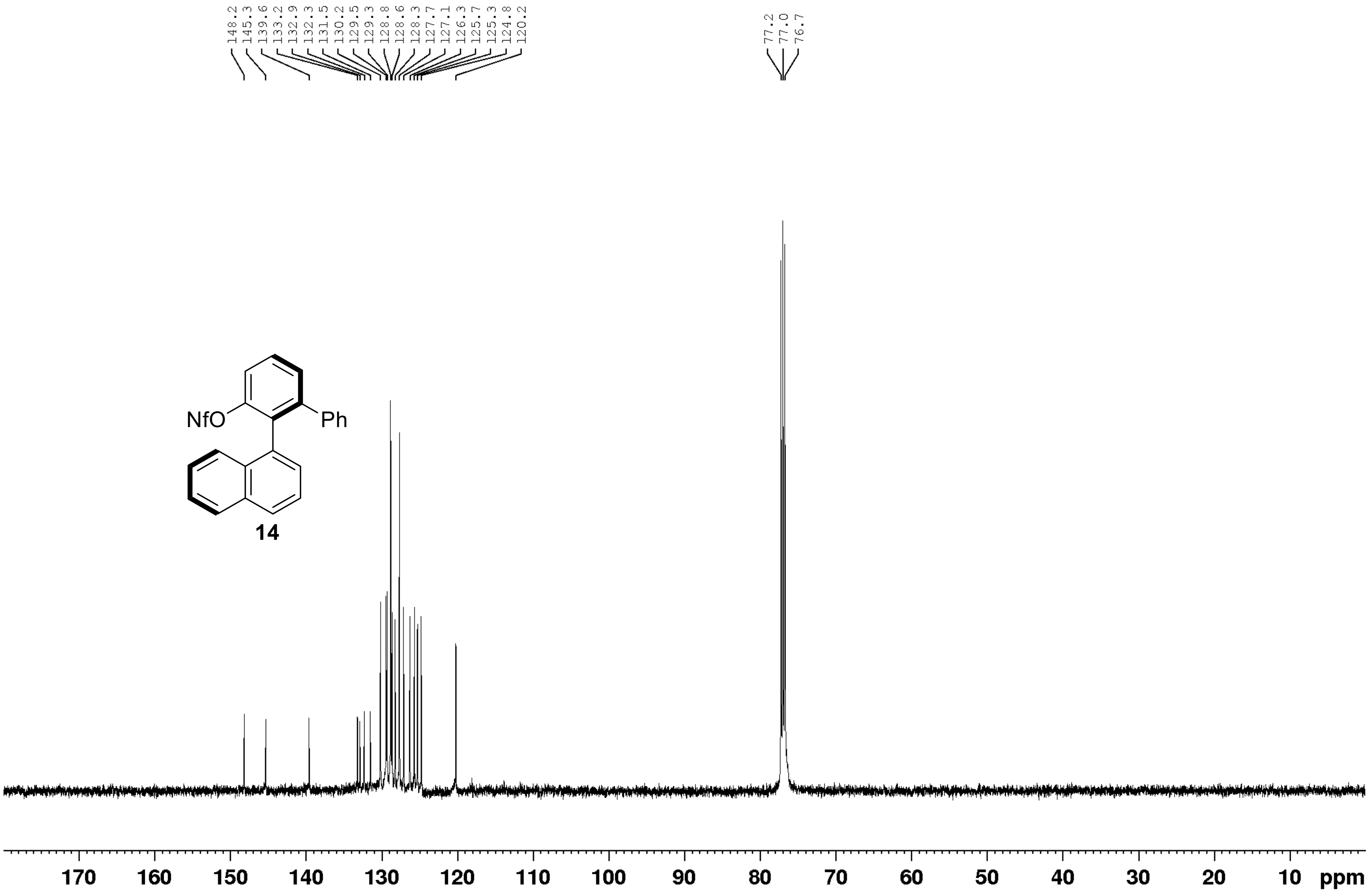

Figure S258. **^{19}F NMR** (471 MHz, $CDCl_3$, 298 K) of **14**

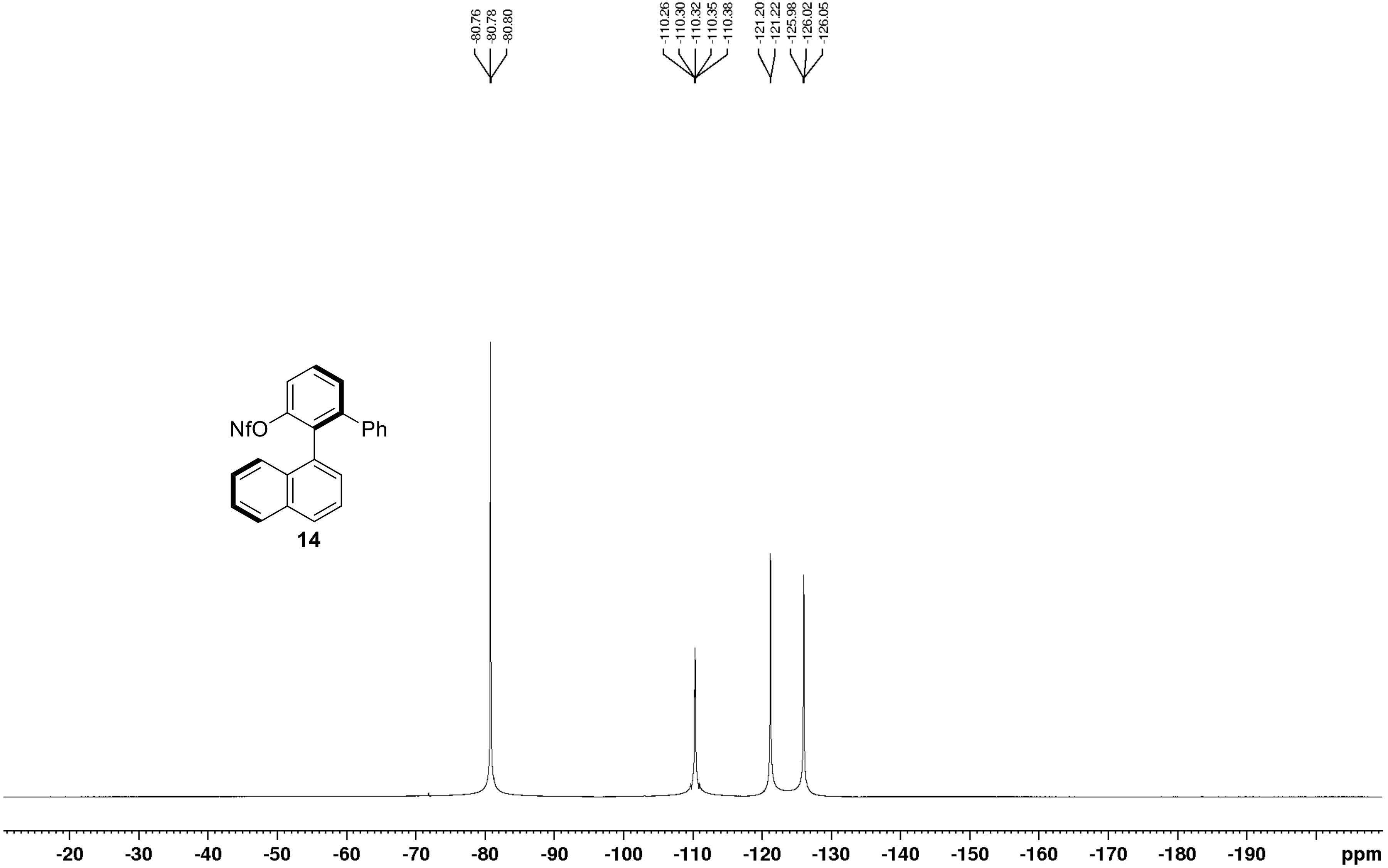

10 References

[S1] R. K. Harris, E. D. Becker, S. M. Cabral de Menezes, R. Goodfellow, P. Granger, *Pure Appl. Chem.* **2001**, *73*, 1795–1818.

[S2] Agilent CrysAlis PRO, **2012**, Agilent Technologies, Yarnton, UK.

[S3] G. M. Sheldrick, *Acta Crystallogr., Sect. A.* **1990**, *46*, 467–473.

[S4] G. M. Sheldrick, *Acta Crystallogr., Sect. A.* **2008**, *64*, 112–122.

[S5] Cambridge Crystallographic Data Centre: http://www.ccdc.cam.ac.uk/Solutions/CSDSystem/Pages/Mercury.aspx.

[S6] J. A. Carmona, V. Hornillos, P. Ramírez-López, A. Ros, J. Iglesias-Sigüenza, E. Gómez-Bengoa, R. Fernández, J. M. Lassaletta, *J. Am. Chem. Soc.* **2018**, *140*, 11067–11075.

[S7] A. Clerc, V. Bénéteau, P. Pale, S. Chassaing, *ChemCatChem*. **2020**, *12*, 2060–2065.

[S8] W.-M. Cheng, R. Shang, B. Zhao, W.-L. Xing, Y. Fu, *Org. Lett.* **2017**, *19*, 4291–4294.

[S9] B. Ramadoss, Y. Jin, S. Asako, L. Ilies, *Science* **2022,** *375*, 658–663.
